# Supplementary material for: Visible light-mediated dearomative spirocyclization/imination of nonactivated arenes through energy transfer catalysis
Source: Nat Commun. 2025 Apr 16;16:3610. doi: 10.1038/s41467-025-58808-0 (PMC12003774; doi:10.1038/s41467-025-58808-0)
Supplement: Supplementary file 1 — Supplementary Information [file 41467_2025_58808_MOESM1_ESM.pdf]

Supplementary Information

Visible Light Mediated Dearomative Spirocyclization/Imination of Nonactivated Arenes through Energy Transfer Catalysis

Chao Zhou,[1] Elena Stepanova,[1,2] Andrey Shatskiy,[1] Markus D. Kärkäs[1] and Peter Dinér[1]*

[1] Department of Chemistry, KTH Royal Institute of Technology, SE-100 44 Stockholm, Sweden

[2] Research School of Chemistry & Applied Biomedical Sciences, Tomsk Polytechnic University, 30 Lenin Avenue, 634050 Tomsk, Russia

* Corresponding author e-mail: diner@kth.se (P.D.)

Table of Contents

1. Materials and methods **8**

2. General procedure for preparation of substrates **2**

3. Optimization of reaction conditions **6**

General procedures for catalytic spirocyclization / imination **9**

4. Mechanistic studies **12**

Fluorescence quenching studies 12

Detection and trapping of intermediates 15

Light on-off experiment 16

5. Computational studies **18**

Computational details 18

Gibbs free energy diagrams 19

6. Characterization data for substrates and products **23**

2-(4-(4-chlorobenzoyl)phenoxy)-1-(((diphenylmethylene)amino)oxy)-2-methylpropan-1-one (1x) 23

Diphenylmethanone O-(2-(4-(2,2-dichlorocyclopropyl)phenoxy)-2-methylpropanoyl) oxime (1y). 23

(2S,5R)-2-((((diphenylmethylene)amino)oxy)carbonyl)-3,3-dimethyl-4-thia-1-azabicyclo[3.2.0]-heptan-7-one 4,4-dioxide (1aa). 23

(1r,4R)-*N*-((*R*)-1-(((diphenylmethylene)amino)oxy)-1-oxo-3-phenylpropan-2-yl)-4-isopropyl-cyclohexane-1-carboxamide (1ab). 24

N-(2-(((diphenylmethylene)amino)oxy)-2-oxoethyl)-4-methylbenzenesulfonamide (1ac) 24

Diphenylmethanone O-(2,2,5,5-tetramethyltetrahydro-7H-[1,3]dioxolo[4',5':4,5]furo[3,2-d][1,3]-dioxine-8a-carbonyl) oxime (1ad). 24

N-benzyl-*N*-(tert-butyl)-3-(((diphenylmethylene)amino)oxy)-3-oxopropanamide (1ae). 24

N-(*tert*-butyl)-*N*-(3,5-dimethylbenzyl)acrylamide (2c). 25

N-(*tert*-butyl)-*N*-(3,5-dichlorobenzyl)acrylamide (2d). 25

N-acryloyl-*N*-(*tert*-butyl)benzamide (2f) 25

N-acryloyl-*N*-isopropylbenzamide (2g) 25

N-(*tert*-butyl)-*N*-(thiophen-2-ylmethyl)acrylamide (2h) 26

N-(*tert*-butyl)-*N*-(dibenzo[b,d]furan-4-ylmethyl)acrylamide (2i) 26

N-([1,1'-biphenyl]-4-ylmethyl)-*N*-(*tert*-butyl)acrylamide (2j) 26

N-(*tert*-butyl)-*N*-(2-fluorobenzyl)acrylamide (2k) 26

N-(*tert*-butyl)-*N*-(4-fluorobenzyl)acrylamide (2l) 27

N-Benzyl-*N*-(*tert*-butyl)bicyclo[1.1.0]butane-1-carboxamide (2m) 27

2-(*tert*-butyl)-8-((diphenylmethylene)amino)-4-(4-phenylbutyl)-2-azaspiro[4.5]deca-6,9-dien-3-one (3a) 27

2-(*tert*-butyl)-8-((diphenylmethylene)amino)-4-(4,4,4-trifluorobutyl)-2-azaspiro[4.5]deca-6,9-dien-3-one (3b) 28

2-(*tert*-Butyl)-8-((diphenylmethylene)amino)-4-ethyl-2-azaspiro[4.5]deca-6,9-dien-3-one (3c) 28

2-(*tert*-Butyl)-4-(cyclobutylmethyl)-8-((diphenylmethylene)amino)-2-azaspiro[4.5]deca-6,9-dien-3-one (3d) 28

2-(*tert*-Butyl)-4-(cyclohexylmethyl)-8-((diphenylmethylene)amino)-2-azaspiro[4.5]deca-6,9-dien-3-one (3e, 8a) 29

2-(*tert*-Butyl)-4-((4,4-difluorocyclohexyl)methyl)-8-((diphenylmethylene)amino)-2-azaspiro[4.5]deca-6,9-dien-3-one (3f) 29

2-(*tert*-Butyl)-8-((diphenylmethylene)amino)-4-neopentyl-2-azaspiro[4.5]deca-6,9-dien-3-one (3g) 30

4-(Adamantan-1-ylmethyl)-2-(*tert*-butyl)-8-((diphenylmethylene)amino)-2-azaspiro[4.5]deca-6,9-dien-3-one (3h) 30

7-(*tert*-butyl)-2-((diphenylmethylene)amino)-9-(3-phenylpropyl)-1-thia-7-azaspiro[4.4]non-3-en-8-one (3i) 30

1'-(tert-butyl)-1-((diphenylmethylene)amino)-4'-(3-phenylpropyl)-1H-spiro[dibenzo[b,d]furan-4,3'-pyrrolidin]-5'-one (3j) 31

2-(tert-butyl)-8-((diphenylmethylene)amino)-6-fluoro-4-(3-phenylpropyl)-2-azaspiro[4.5]deca-6,9-dien-3-one (3k) 33

2-(*tert*-butyl)-8-((diphenylmethylene)amino)-6-fluoro-4-(3-phenylpropyl)-2-azaspiro[4.5]deca-6,9-dien-3-one (3l). 34

2-(*tert*-butyl)-8-((diphenylmethylene)amino)-4-(2-oxo-2-phenylethyl)-2-azaspiro[4.5]deca-6,9-dien-3-one (3m, 8e) 35

2-(*tert*-Butyl)-8-((diphenylmethylene)amino)-4-(2-mesityl-2-oxoethyl)-2-azaspiro[4.5]deca-6,9-dien-3-one (3n) 35

Methyl 2-(2-(*tert*-butyl)-8-((diphenylmethylene)amino)-3-oxo-2-azaspiro[4.5]deca-6,9-dien-4-yl)acetate (3o) 35

Ethyl 2-(2-(*tert*-butyl)-8-((diphenylmethylene)amino)-3-oxo-2-azaspiro[4.5]deca-6,9-dien-4-yl)acetate (3p) 36

Methyl 2-(2-(*tert*-butyl)-8-((diphenylmethylene)amino)-7,9-dimethoxy-3-oxo-2-azaspiro[4.5]deca-6,9-dien-4-yl)acetate (3q) 36

Methyl 2-(2-(*tert*-butyl)-8-((diphenylmethylene)amino)-7,9-dimethyl-3-oxo-2-azaspiro[4.5]deca-6,9-dien-4-yl)acetate (3r) 37

Methyl 2-(2-(*tert*-butyl)-7,9-dichloro-8-((diphenylmethylene)amino)-3-oxo-2-azaspiro[4.5]deca-6,9-dien-4-yl)acetate (3s) 37

Methyl 2-(2-(*tert*-butyl)-8-((diphenylmethylene)amino)-6,10-dimethyl-3-oxo-2-azaspiro[4.5]deca-6,9-dien-4-yl)acetate (3t) 38

Methyl 2-(2-(*tert*-butyl)-8-((diphenylmethylene)amino)-1,3-dioxo-2-azaspiro[4.5]deca-6,9-dien-4-yl)acetate (3u) 38

Methyl 2-(8-((diphenylmethylene)amino)-2-isopropyl-1,3-dioxo-2-azaspiro[4.5]deca-6,9-dien-4-yl)acetate (3v) 39

2-(*tert*-Butyl)-4-(5-(2,5-dimethylphenoxy)-2,2-dimethylpentyl)-8-((diphenylmethylene)amino)-2-azaspiro[4.5]deca-6,9-dien-3-one (3w) 39

2-(*tert*-butyl)-4-(2-(4-(4-chlorobenzoyl)phenoxy)-2-methylpropyl)-8-((diphenylmethylene)amino)-2-azaspiro[4.5]deca-6,9-dien-3-one (3x) 40

2-(tert-butyl)-4-(2-(4-(2,2-dichlorocyclopropyl)phenoxy)-2-methylpropyl)-8-((diphenylmethylene)-amino)-2-azaspiro[4.5]deca-6,9-dien-3-one (3y) 40

2-(*tert*-Butyl)-8-((diphenylmethylene)amino)-4-(3-(4,5-diphenyloxazol-2-yl)propyl)-2-azaspiro-[4.5]deca-6,9-dien-3-one (3z) 41

(5R)-2-((2-(*tert*-butyl)-8-((diphenylmethylene)amino)-3-oxo-2-azaspiro[4.5]deca-6,9-dien-4-yl)methyl)-3,3-dimethyl-4-thia-1-azabicyclo[3.2.0]heptan-7-one 4,4-dioxide (3aa) 41

(1r,4R)-*N*-(1-2-(*tert*-butyl)-8-((diphenylmethylene)amino)-3-oxo-2-azaspiro[4.5]deca-6,9-dien-4-yl)-3-phenylpropan-2-yl)-4-isopropylcyclohexane-1-carboxamide (3ab) 42

N-(2-(2-(tert-butyl)-8-((diphenylmethylene)amino)-3-oxo-2-azaspiro[4.5]deca-6,9-dien-4-yl)ethyl)-4-methylbenzenesulfonamide (3ac) 43

2-(tert-butyl)-8-((diphenylmethylene)amino)-4-((2,2,5,5-tetramethyltetrahydro-8aH-[1,3]dioxolo[4',5':4,5]furo[3,2-d][1,3]dioxin-8a-yl)methyl)-2-azaspiro[4.5]deca-6,9-dien-3-one (3ad) 43

2-(*tert*-butyl)-8-((diphenylmethylene)amino)-2-azaspiro[4.5]deca-6,9-dien-3-one (3ae). 44

Methyl(2r,4r)-12-(tert-butyl)-8-((diphenylmethylene)amino)-13-oxo-12-azadispiro[3.0.55.34]trideca-6,9-diene-2-carboxylate (3af). 44

(1-((2-(*tert*-Butyl)-8-((diphenylmethylene)amino)-3-oxo-2-azaspiro[4.5]deca-6,9-dien-4-yl)methyl)-cyclohexyl)methyl methyl carbonate (6a) 45

(1-((2-(*tert*-Butyl)-8-((diphenylmethylene)amino)-3-oxo-2-azaspiro[4.5]deca-6,9-dien-4-yl)methyl)-cyclohexyl)methyl ethyl carbonate (6b) 45

Butyl ((1-((2-(*tert*-butyl)-8-((diphenylmethylene)amino)-3-oxo-2-azaspiro[4.5]deca-6,9-dien-4-

yl)methyl)cyclohexyl)methyl) carbonate (6c) 46

(4-((2-(*tert*-Butyl)-8-((diphenylmethylene)amino)-3-oxo-2-azaspiro[4.5]deca-6,9-dien-4-yl)methyl)-tetrahydro-2H-pyran-4-yl)methyl methyl carbonate (6d) 46

(1-((2-(*tert*-Butyl)-8-((diphenylmethylene)amino)-3-oxo-2-azaspiro[4.5]deca-6,9-dien-4-yl)methyl)-cyclopentyl)methyl methyl carbonate (6e) 47

(1-((2-(*tert*-Butyl)-8-((diphenylmethylene)amino)-3-oxo-2-azaspiro[4.5]deca-6,9-dien-4-yl)methyl)cyclobutyl)methyl methyl carbonate (6f). 48

2-((2-(*tert*-Butyl)-8-((diphenylmethylene)amino)-3-oxo-2-azaspiro[4.5]deca-6,9-dien-4-yl)methyl)-2-ethylbutyl methyl carbonate (6g) 48

(1-((2-(*tert*-Butyl)-8-((diphenylmethylene)amino)-7,9-dimethyl-3-oxo-2-azaspiro[4.5]deca-6,9-dien-4-yl)methyl)cyclohexyl)methyl methyl carbonate (6h). 49

(1-((2-(*tert*-Butyl)-7,9-dichloro-8-((diphenylmethylene)amino)-3-oxo-2-azaspiro[4.5]deca-6,9-dien-4-yl)methyl)cyclohexyl)methyl methyl carbonate (6i). 49

(1-((2-(*tert*-Butyl)-8-((diphenylmethylene)amino)-6,10-dimethyl-3-oxo-2-azaspiro[4.5]deca-6,9-dien-4-yl)methyl)cyclohexyl)methyl methyl carbonate (6j) 49

2-(*tert*-Butyl)-4-(cyclopentylmethyl)-8-((diphenylmethylene)amino)-2-azaspiro[4.5]deca-6,9-dien-3-one (8b) 50

2-(*tert*-Butyl)-4-(cycloheptylmethyl)-8-((diphenylmethylene)amino)-2-azaspiro[4.5]deca-6,9-dien-3-one (8c) 50

4-(2-(*tert*-butoxy)ethyl)-2-(*tert*-butyl)-8-((diphenylmethylene)amino)-2-azaspiro[4.5]deca-6,9-dien-3-one (8d) 51

8-Amino-2-(*tert*-butyl)-4-(3-phenylpropyl)-2-azaspiro[4.5]deca-6,9-dien-3-one (9) 51

Ethyl 2-(8-amino-2-(*tert*-butyl)-3-oxo-2-azaspiro[4.5]deca-6,9-dien-4-yl)acetate (10) 51

Ethyl 2-(8-(benzhydrylamino)-2-(*tert*-butyl)-3-oxo-2-azaspiro[4.5]deca-6,9-dien-4-yl)acetate (11).......... 52

7. NMR spectra of substrates and products 53

2-(4-(4-chlorobenzoyl)phenoxy)-1-(((diphenylmethylene)amino)oxy)-2-methylpropan-1-one (1x).......... 53

Diphenylmethanone O-(2-(4-(2,2-dichlorocyclopropyl)phenoxy)-2-methylpropanoyl) oxime (1y).......... 54

(2S,5R)-2-((((diphenylmethylene)amino)oxy)carbonyl)-3,3-dimethyl-4-thia-1-azabicyclo[3.2.0]heptan-7-one 4,4-dioxide (1aa). 55

1r,4R)-*N*-((*R*)-1-(((diphenylmethylene)amino)oxy)-1-oxo-3-phenylpropan-2-yl)-4-isopropylcyclohexane-1-carboxamide (1ab) 56

N-(2-(((diphenylmethylene)amino)oxy)-2-oxoethyl)-4-methylbenzenesulfonamide (1ac).......... 57

Diphenylmethanone *O*-(2,2,5,5-tetramethyltetrahydro-7H-[1,3]dioxolo[4',5':4,5]furo[3,2-d][1,3]dioxine-

8a-carbonyl) oxime (1ad) 58

N-benzyl-*N*-(*tert*-butyl)-3-(((diphenylmethylene)amino)oxy)-3-oxopropanamide (1ae) 59

N-(tert-butyl)-N-(3, 5-dimethylbenzyl)acrylamide (2c) 60

N-(*tert*-butyl)-*N*-(3,5-dichlorobenzyl)acrylamide (2d) 61

N-acryloyl-*N*-(*tert*-butyl)benzamide (2f) 62

N-acryloyl-*N*-isopropylbenzamide (2g) 63

N-(*tert*-butyl)-*N*-(thiophen-2-ylmethyl)acrylamide (2h) 64

N-(*tert*-butyl)-*N*-(dibenzo[b,d]furan-4-ylmethyl)acrylamide (2i). 65

N-(*tert*-butyl)-*N*-(2-fluorobenzyl)acrylamide (2j). 66

N-([1,1'-biphenyl]-4-ylmethyl)-*N*-(*tert*-butyl)acrylamide (2k). 68

N-benzyl-*N*-(tert-butyl)bicyclo[1.1.0]butane-1-carboxamide (2m). 69

2-(*tert*-Butyl)-8-((diphenylmethylene)amino)-4-(4-phenylbutyl)-2-azaspiro[4.5]deca-6,9-dien-3-one (3a)70

2-(*tert*-Butyl)-8-((diphenylmethylene)amino)-4-(4,4,4-trifluorobutyl)-2-azaspiro[4.5]deca-6,9-dien-3-one (3b) 71

2-(*tert*-Butyl)-8-((diphenylmethylene)amino)-4-ethyl-2-azaspiro[4.5]deca-6,9-dien-3-one (3c) 73

2-(*tert*-Butyl)-4-(cyclobutylmethyl)-8-((diphenylmethylene)amino)-2-azaspiro[4.5]deca-6,9-dien-3-one (3d) 74

2-(*tert*-Butyl)-4-(cyclohexylmethyl)-8-((diphenylmethylene)amino)-2-azaspiro[4.5]deca-6,9-dien-3-one (3e, 8a) 75

2-(*tert*-Butyl)-4-((4,4-difluorocyclohexyl)methyl)-8-((diphenylmethylene)amino)-2-azaspiro[4.5]deca-6,9-dien-3-one (3f) 76

2-(*tert*-Butyl)-8-((diphenylmethylene)amino)-4-neopentyl-2-azaspiro[4.5]deca-6,9-dien-3-one (3g) 78

4-(Adamantan-1-ylmethyl)-2-(*tert*-butyl)-8-((diphenylmethylene)amino)-2-azaspiro[4.5]deca-6,9-dien-3-one (3h) 79

7-(*tert*-butyl)-2-((diphenylmethylene)amino)-9-(3-phenylpropyl)-1-thia-7-azaspiro[4.4]non-3-en-8-one (3i) 80

1'-(*tert*-butyl)-1-((diphenylmethylene)amino)-4'-(3-phenylpropyl)-1H-spiro[dibenzo[b,d]furan-4,3'-pyrrolidin]-5'-one (3j) 84

2-(tert-butyl)-8-((diphenylmethylene)amino)-6-fluoro-4-(3-phenylpropyl)-2-azaspiro[4.5]deca-6,9-dien-3-one (3k) 94

2-(*tert*-butyl)-8-((diphenylmethylene)amino)-6-fluoro-4-(3-phenylpropyl)-2-azaspiro[4.5]deca-6,9-dien-3-one (3l) 103

2-(*tert*-Butyl)-8-((diphenylmethylene)amino)-4-(2-oxo-2-phenylethyl)-2-azaspiro[4.5]deca-6,9-dien-3-one (3m, 8e) 104

2-(*tert*-Butyl)-8-((diphenylmethylene)amino)-4-(2-mesityl-2-oxoethyl)-2-azaspiro[4.5]deca-6,9-dien-3-one (3n) 105

Methyl 2-(2-(*tert*-butyl)-8-((diphenylmethylene)amino)-3-oxo-2-azaspiro[4.5]deca-6,9-dien-4-yl)acetate (3o) 106

Ethyl 2-(2-(*tert*-butyl)-8-((diphenylmethylene)amino)-3-oxo-2-azaspiro[4.5]deca-6,9-dien-4-yl)acetate (3p) 107

Methyl 2-(2-(*tert*-butyl)-8-((diphenylmethylene)amino)-7,9-dimethoxy-3-oxo-2-azaspiro[4.5]deca-6,9-dien-4-yl)acetate (3q) 108

Methyl 2-(2-(*tert*-butyl)-8-((diphenylmethylene)amino)-7,9-dimethyl-3-oxo-2-azaspiro[4.5]deca-6,9-dien-4-yl)acetate (3r). 109

Methyl 2-(2-(*tert*-butyl)-7,9-dichloro-8-((diphenylmethylene)amino)-3-oxo-2-azaspiro[4.5]deca-6,9-dien-4-yl)acetate (3s) 110

Methyl 2-(2-(*tert*-butyl)-8-((diphenylmethylene)amino)-6,10-dimethyl-3-oxo-2-azaspiro[4.5]deca-6,9-dien-4-yl)acetate (3t) 111

Methyl 2-(2-(*tert*-butyl)-8-((diphenylmethylene)amino)-1,3-dioxo-2-azaspiro[4.5]deca-6,9-dien-4-yl)acetate (3u) 112

Methyl 2-(8-((diphenylmethylene)amino)-2-isopropyl-1,3-dioxo-2-azaspiro[4.5]deca-6,9-dien-4-yl)acetate (3v) 114

2-(*tert*-Butyl)-4-(5-(2,5-dimethylphenoxy)-2,2-dimethylpentyl)-8-((diphenylmethylene)amino)-2-azaspiro[4.5]deca-6,9-dien-3-one (3w) 116

2-(tert-butyl)-4-(2-(4-(4-chlorobenzoyl)phenoxy)-2-methylpropyl)-8-((diphenylmethylene)amino)-2-azaspiro[4.5]deca-6,9-dien-3-one (3x) 117

2-(*tert*-butyl)-4-(2-(4-(2,2-dichlorocyclopropyl)phenoxy)-2-methylpropyl)-8-((diphenylmethylene)amino)-2-azaspiro[4.5]deca-6,9-dien-3-one (3y) 123

2-(*tert*-Butyl)-8-((diphenylmethylene)amino)-4-(3-(4,5-diphenyloxazol-2-yl)propyl)-2-azaspiro[4.5]deca-6,9-dien-3-one (3z) 124

(5*R*)-2-((2-(*tert*-butyl)-8-((diphenylmethylene)amino)-3-oxo-2-azaspiro[4.5]deca-6,9-dien-4-yl)methyl)-3,3-dimethyl-4-thia-1-azabicyclo[3.2.0]heptan-7-one 4,4-dioxide (3aa). 125

(1r,4R)-*N*-(1-((R)-2-(*tert*-butyl)-8-((diphenylmethylene)amino)-3-oxo-2-azaspiro[4.5]deca-6,9-dien-4-yl)-3-phenylpropan-2-yl)-4-isopropylcyclohexane-1-carboxamide (3ab). 126

N-(2-(2-(tert-butyl)-8-((diphenylmethylene)amino)-3-oxo-2-azaspiro[4.5]deca-6,9-dien-4-yl)ethyl)-4-methylbenzenesulfonamide (3ac) 127

2-(*tert*-butyl)-8-((diphenylmethylene)amino)-4-((2,2,5,5-tetramethyltetrahydro-8aH-[1,3]dioxolo[4',5':4,5]furo[3,2-d][1,3]dioxin-8a-yl)methyl)-2-azaspiro[4.5]deca-6,9-dien-3-one (3ad).... 133

2-(tert-butyl)-8-((diphenylmethylene)amino)-2-azaspiro[4.5]deca-6,9-dien-3-one (3ae) 134

Methyl(2r,4r)-12-(tert-butyl)-8-((diphenylmethylene)amino)-13-oxo-12-azadispiro[3.0.55.34]trideca-6,9-diene-2-carboxylate (3af).. 139

(1-((2-(tert-Butyl)-8-((diphenylmethylene)amino)-3-oxo-2-azaspiro[4.5]deca-6,9-dien-4-yl)methyl)cyclohexyl)methyl methyl carbonate (6a)... 140

(1-((2-(*tert*-Butyl)-8-((diphenylmethylene)amino)-3-oxo-2-azaspiro[4.5]deca-6,9-dien-4-yl)methyl)cyclohexyl)methyl ethyl carbonate (6b)... 141

Butyl ((1-((2-(*tert*-butyl)-8-((diphenylmethylene)amino)-3-oxo-2-azaspiro[4.5]deca-6,9-dien-4-yl)methyl)cyclohexyl)methyl) carbonate (6c)... 142

(4-((2-(*tert*-Butyl)-8-((diphenylmethylene)amino)-3-oxo-2-azaspiro[4.5]deca-6,9-dien-4-yl)methyl)tetrahydro-2H-pyran-4-yl)methyl methyl carbonate (6d) 143

(1-((2-(*tert*-Butyl)-8-((diphenylmethylene)amino)-3-oxo-2-azaspiro[4.5]deca-6,9-dien-4-yl)methyl)cyclopentyl)methyl methyl carbonate (6e) 145

(1-((2-(*tert*-Butyl)-8-((diphenylmethylene)amino)-3-oxo-2-azaspiro[4.5]deca-6,9-dien-4-yl)methyl)cyclobutyl)methyl methyl carbonate (6f)... 151

2-((2-(*tert*-Butyl)-8-((diphenylmethylene)amino)-3-oxo-2-azaspiro[4.5]deca-6,9-dien-4-yl)methyl)-2-ethylbutyl methyl carbonate (6g) 152

(1-((2-(*tert*-Butyl)-8-((diphenylmethylene)amino)-7,9-dimethyl-3-oxo-2-azaspiro[4.5]deca-6,9-dien-4-yl)methyl)cyclohexyl)methyl methyl carbonate (6h).. 153

(1-((2-(*tert*-Butyl)-7,9-dichloro-8-((diphenylmethylene)amino)-3-oxo-2-azaspiro[4.5]deca-6,9-dien-4-yl)methyl)cyclohexyl)methyl methyl carbonate (6i)... 154

(1-((2-(*tert*-Butyl)-8-((diphenylmethylene)amino)-6,10-dimethyl-3-oxo-2-azaspiro[4.5]deca-6,9-dien-4-yl)methyl)cyclohexyl)methyl methyl carbonate (6j)... 155

2-(*tert*-Butyl)-4-(cyclopentylmethyl)-8-((diphenylmethylene)amino)-2-azaspiro[4.5]deca-6,9-dien-3-one (8b).. 156

2-(*tert*-Butyl)-4-(cycloheptylmethyl)-8-((diphenylmethylene)amino)-2-azaspiro[4.5]deca-6,9-dien-3-one (8c).. 157

4-(2-(tert-Butoxy)ethyl)-2-(tert-butyl)-8-((diphenylmethylene)amino)-2-azaspiro[4.5]deca-6,9-dien-3-one (8d).. 159

8-Amino-2-(*tert*-butyl)-4-(3-phenylpropyl)-2-azaspiro[4.5]deca-6,9-dien-3-one (9)............................. 160

Ethyl 2-(8-amino-2-(tert-butyl)-3-oxo-2-azaspiro[4.5]deca-6,9-dien-4-yl)acetate (10). 161

Ethyl 2-(8-(benzhydrylamino)-2-(tert-butyl)-3-oxo-2-azaspiro[4.5]deca-6,9-dien-4-yl)acetate (11)........ 162

8. References.. 163

1. Materials and methods

All reagents were obtained from commercial sources and used without further purification. All solvents were purified and dried according to standard methods prior to use, unless stated otherwise. Other benzoic acid derivatives were prepared by using the reported procedure and purified through column chromatography respectively. Thin-layer chromatography (TLC) was performed using 60 mesh silica gel plates visualized with short-wavelength UV light (254 nm). Silica gel 60 (200–300 mesh) was used for column chromatography. A Bruker Ascend 400 spectrometer (400 MHz) or Bruker Avance DMX 500 (500 MHz) spectrometer was used for the recording of ^{1}H NMR spectra, ^{13}C NMR spectra and ^{19}F NMR spectra. Proton chemical shifts are reported as δ values (ppm) relative to tetramethylsilane with residual undeuterated $CDCl_3$ (δ 7.26), DMSO-d_6 (δ 2.50), MeCN-d_3 (δ 1.94) and CD_3OD (δ 3.31) as internal standards. ^{13}C chemical shifts are reported as δ values (ppm) relative to tetramethylsilane with $CDCl_3$ (δ 77.16 ppm), DMSO-d_6 (δ 39.52 ppm), MeCN-d_3 (δ 1.32 and 118.26 ppm) or CD_3OD (δ 49.0 ppm) as internal standards. Data for ^{1}H NMR are reported as follows: chemical shift (δ, ppm), multiplicity (s = singlet, d = doublet, t = triplet, q = quartet, m = multiplet or unresolved, br = broad singlet, *J*= coupling constants in Hz, integration). HRMS measurements were performed on methanolic solutions of the compounds with Bruker maXis impact II micrOTOF spectrometer (direct injection, electrospray ionization, ESI). The photoreactions were carried out in 8 mL vials equipped with a stirring bar unless otherwise noted, and then were illuminated with 440 nm LED (40 W, Kessil PR160) with continuous stirring.

2. General procedure for preparation of substrates

Method A

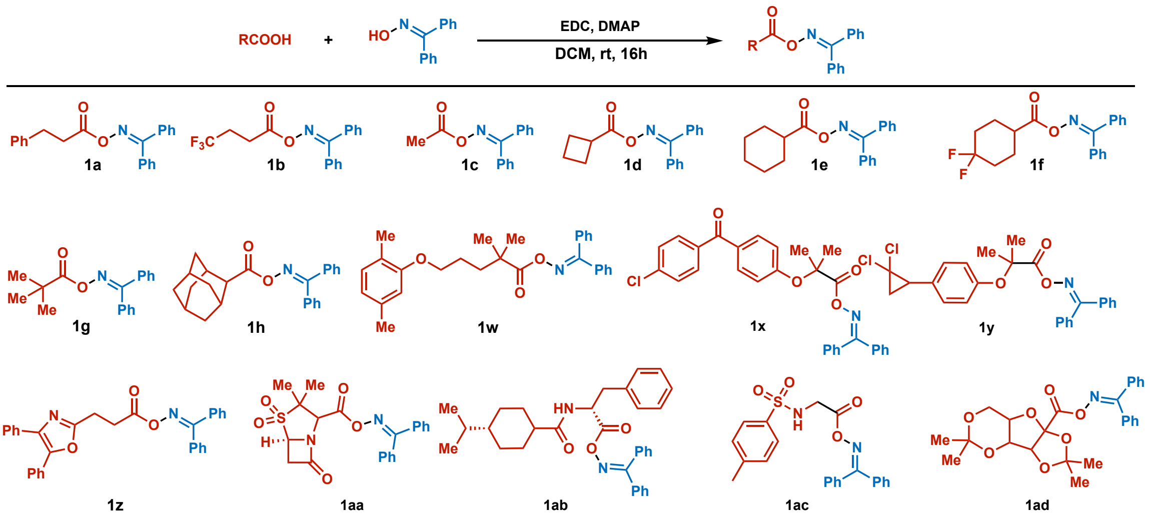

To a solution of the corresponding carboxylic acid (1.0 equiv., 2.0 mmol), benzophenone oxime (1.0 equiv., 2.0 mmol and 4-dimethylaminopyridine (DMAP, 0.1 equiv., 0.2 mmol) in CH_2Cl_2 (20.0 mL). Then, *N*-(3-dimethylaminopropyl)-*N*'-ethylcarbodiimide hydrochloride (EDC·HCl, 5.0 mmol, 2.5 equiv.) were added. Upon completion of addition, the reaction was stirred at room temperature for 16 h. Water was added and the reaction mixture was extracted 3 times with EtOAc. The combined organic layers were dried over anhydrous Na_2SO_4, filtered and the organic solvent was evaporated in vacuo and the crude product was purified by flash chromatography using silica gel to give the corresponding product. The characterization data of **1a–1h**, **1w** and **1z** were in accordance with previous reports.[1]

Method B

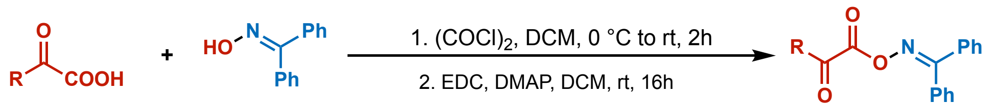

1i (R = Ph)
1j (R = Mes)

Equipped with a magnetic stirring bar in a 50 mL round bottom flask, the corresponding carboxylic acid (1.0 equiv., 4.0 mmol) was dissolved in CH_2Cl_2 (20.0 mL), and the reaction mixture was cooled at 0 °C. Next, a drop of DMF and oxalyl chloride (1.3 equiv., 5.2 mmol) were added dropwise and the resulting mixture was stirred for 2 hours. After removal of oxalyl chloride under

reduced pressure, CH_2Cl_2, (20.0 mL) and DMAP (0.1 equiv., 0.4 mmol) and EDC (2.5 equiv., 10.0 mmol) were added. Upon completion of addition, the reaction was stirred at room temperature for 16 h. Water was added, and the reaction mixture was extracted 3 times with EtOAc. The combined organic layers were dried over anhydrous Na_2SO_4, filtered and the organic solvent was evaporated in vacuo and the crude product was purified by flash chromatography using silica gel to give the corresponding product **1i** and **1j**. The characterization data of **1i** and **1j** were in accordance with previous reports.[1]

Method C

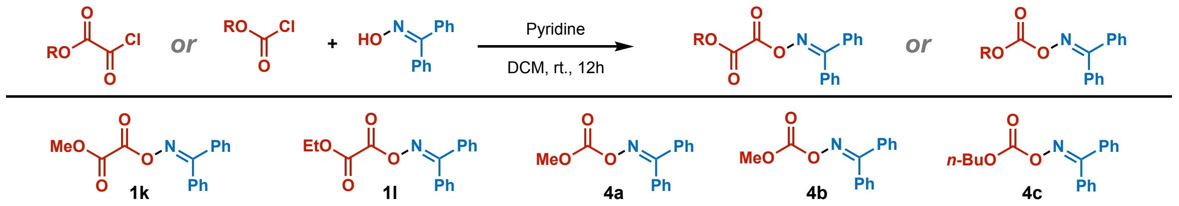

To a solution of diphenylmethanone oxime (1.97 g, 10 mmol, 1.0 equiv.) and pyridine (0.89 mL, 11 mmol, 1.1 equiv.) in dry CH_2Cl_2 (30 mL), the corresponding acyl chloride (0.92 mL, 10 mmol, 1.0 equiv.) was added dropwise at room temperature under inert atmosphere. The solution was stirred at room temperature overnight. Water was added and the reaction mixture was extracted 3 times with EtOAc. The combined organic layers were dried over anhydrous Na_2SO_4, filtered and the organic solvent was evaporated in vacuo and the crude product was sufficiently pure (as determined from NMR) and was used without further purification for the catalytic reactions. The characterization data of **1k**, **1l** and **4a–4c** were in accordance with previous reports.[2,3]

Method D

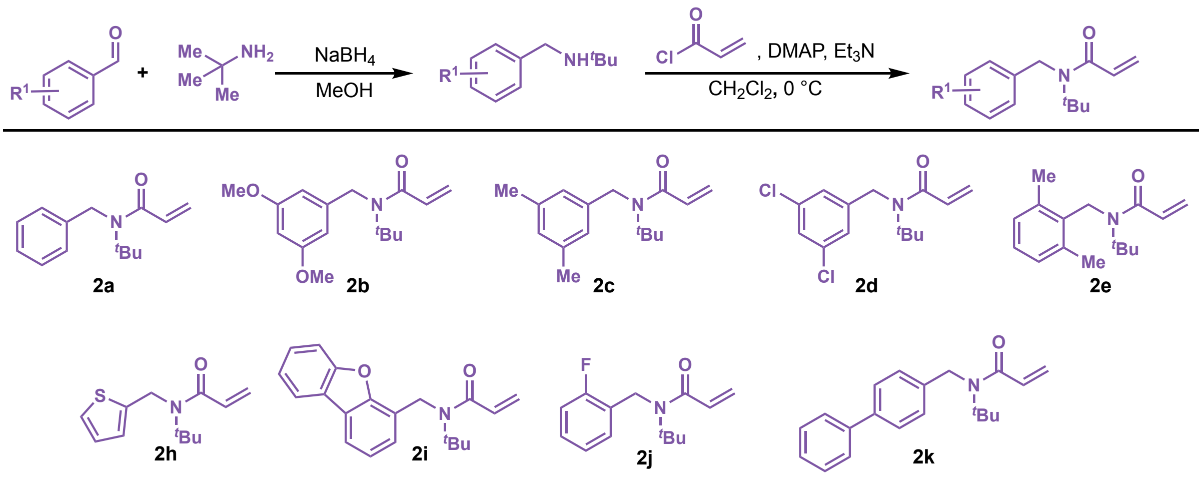

Aryl aldehydes (5.0 mmol, 1.0 equiv.) and *tert*-butyl amine (6 mmol, 1.2 equiv.) were stirred in MeOH (25 mL) for 2 hours. $NaBH_4$ (7.5 mmol, 1.5 equiv.) was added and the reaction mixture was stirred for an additional 1 hour. The organic solvent was evaporated in vacuo, and the residue was purified via flash chromatography using silica to give the corresponding product *tert*-butylamines.

To a flame dried flask equipped with a magnetic stir bar, were added *tert*-butylamine (2.2 equiv.), triethylamine (2 equiv.) and DMAP (0.1 equiv.) in CH_2Cl_2 under argon atmosphere at room temperature. After stirring at 0 °C under a nitrogen atmosphere for 15 min, a solution of acryloyl chloride (1 equiv.) in CH_2Cl_2 was slowly added dropwise at 0 °C. After stirring for 14 h at room temperature, HCl (2 M) was added, and the layers were separated. The organic layer was washed with HCl (2 M) and water, and the organic phase was dried with $MgSO_4$ and concentrated under reduced pressure to afford the target product **2a–2e**. The characterization data of **2a**, **2b** and **2e** were in accordance with previous reports.[4]

Method E

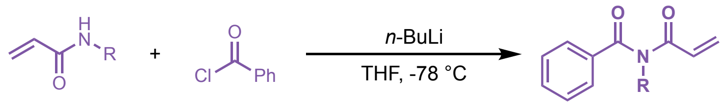

2f (R = ^{t}Bu)
2g (R = ^{i}Pr)

To a solution of acrylamide (1.0 equiv.) in THF at –78 °C was added n-BuLi (1.1 equiv.) dropwise. The resulted mixture was stirred at this temperature for 15 min. A solution of corresponding benzoyl chloride (1.3 equiv.) was added dropwise at –78 °C. The reaction was warmed to room temperature gradually and stirred for another 5 h. Water was added and the reaction mixture was extracted three times with EtOAc. The combined organic layers were dried over anhydrous Na_2SO_4, filtered and the organic solvent was evaporated in vacuo and the crude product was purified by flash chromatography using silica gel to give the corresponding product **2f** and **2g**.[5]

Method F

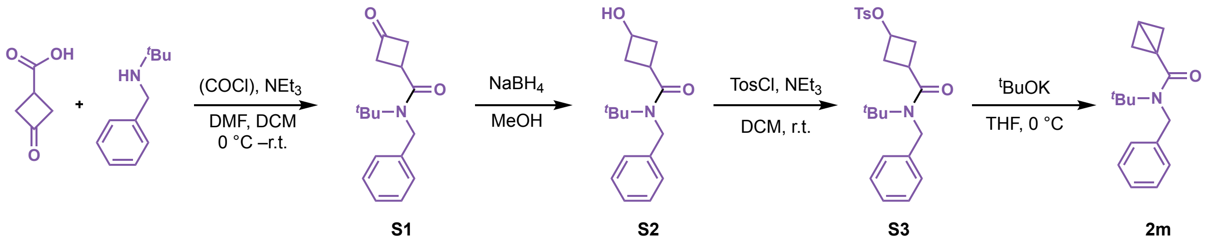

Step1: 3-Oxocyclobutane-1-carboxylic acid (1.2 equiv) was dissolved in DCM, and two drops of DMF were added. The resulting solution was cooled to 0 °C. Then $(COCl)_2$ (1.3 equiv) was slowly added, and the reaction mixture was stirred for 2 hours. The *N*-benzyl-2-methylpropan-2-amine (1.0 equiv) and NEt_3 (1.5 equiv) were dissolved in DCM, and the above acyl chloride solution was slowly added to the reaction at 0 °C. The reaction mixture was stirred for 2 hours until TLC shows complete conversion. Subsequently, the reaction mixture was extracted using DCM and water. The organic layer was dried over anhydrous Na_2SO_4, filtrated and concentrated in vacuo. The residue was purified by column chromatography to afford compound **S1**.

Step 2: Compound **S1** (1.0 equiv) was dissolved in MeOH (0.3 M) and cooled to 0 °C and NaBH4 (1.5 equiv) was carefully added. The reaction mixture was stirred for 30 min at 0 °C and reaction mixture was quenched with water, concentrated in vacuo, and extracted two times with EtOAc. The combined organic phase was dried with anhydrous Na_2SO_4, filtered, and removed under vaccum. The crude material was used directly in next step without further purification.

Step 3: The crude product **S2** (1.0 equiv) was dissolved in dry DCM (1.0 M), and TsCl (1.3 equiv) and triethylamine (1.3 equiv) were added. The reaction mixture was stirred at room temperature for 12 hours until TLC shows complete conversion of the alcohol. Subsequently, the reaction mixture was extracted using DCM and water and the organic phase was dried with anhydrous Na_2SO_4, filtrated and concentrated in vacuo. The crude tosylate product was purified by column chromatography to afford **S3**.

Step 4: To a solution of tosylate product **S3** (1.0 equiv) in dry THF (0.3 M) at 0 °C and under argon atmosphere, *t*BuOK (1.0 M in THF solution, 1.1 equiv) was added dropwise. The reaction mixture was stirred for 30 min and the reaction was quenched with saturated NH_4Cl (aq.) The aqueous phase was extracted with DCM and the combined organic fractions were dried with anhydrous Na_2SO_4, filtrated and concentrated in vacuo. The residue was purified by column chromatography to afford **2m**.[6]

3. Optimization of reaction conditions

Table S1. Optimization photocatalysts and solvents for dearomative spirocyclization/imination.[a]

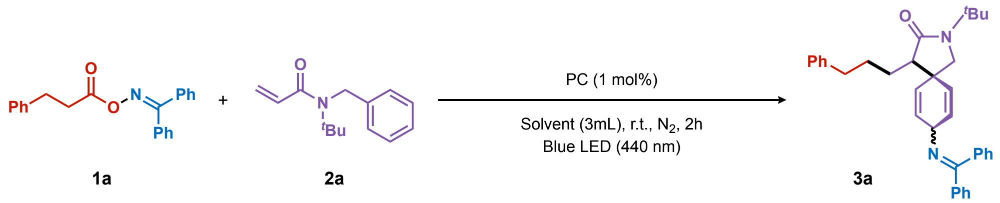

Entry	PC (1 mol%)	Solvent	Yield (%)[b]	dr[b]
1	[Ir(dFCF$_3$ppy)$_2$(dtbbpy)]PF$_6$	EtOAc	50	1.4:1
2	Ir(ppy)$_3$	EtOAc	30	1.3:1
3	4CzIPN	EtOAc	34	1.4:1
4	3DPAFIPN	EtOAc	5	1.4:1
5	4DPAIPN	EtOAc	<5	1.4:1
6[c]	Thioxanthen-9-one	EtOAc	38	1.3:1
7[c,d]	Benzophenone	EtOAc	40	1.4:1
8	[Ir(dFCF$_3$ppy)$_2$(dtbbpy)]PF$_6$	CH_3CN	38	1.3:1
9	[Ir(dFCF$_3$ppy)$_2$(dtbbpy)]PF$_6$	CH_2Cl_2	44	1.3:1
10	[Ir(dFCF$_3$ppy)$_2$(dtbbpy)]PF$_6$	$PhCF_3$	46	1.4:1
11	[Ir(dFCF$_3$ppy)$_2$(dtbbpy)]PF$_6$	acetone	47	1.4:1
12	[Ir(dFCF$_3$ppy)$_2$(dtbbpy)]PF$_6$	DMSO	34	1.4:1
13	[Ir(dFCF$_3$ppy)$_2$(dtbbpy)]PF$_6$	DMF	44	1.3:1
14[e]	[Ir(dFCF$_3$ppy)$_2$(dtbbpy)]PF$_6$	EtOAc	45	1.4:1
15[f]	[Ir(dFCF$_3$ppy)$_2$(dtbbpy)]PF$_6$	EtOAc	46	1.4:1
16[g]	[Ir(dFCF$_3$ppy)$_2$(dtbbpy)]PF$_6$	EtOAc	54	1.4:1
17[g,h]	[Ir(dFCF$_3$ppy)$_2$(dtbbpy)]PF$_6$	EtOAc	56	1.4:1
18	--	EtOAc	0	--
19[i]	[Ir(dFCF$_3$ppy)$_2$(dtbbpy)]PF$_6$	EtOAc	0	--
20[d]	- (390 nm)	EtOAc	25	1.4:1

[a] Reaction conditions: **1a** (0.3 mmol, 1.5 equiv.), **2a** (0.2 mmol, 1.0 equiv.), photocatalyst (1 mol%), solvent (3 mL), N_2, blue LEDs (440 nm), 2 h, room temperature. [b] Yields and dr were determined by ^{1}H NMR using 4-nitrobenzonitrile as the internal standard. [C] 20 mol% photocatalyst. [d] Violet LEDs (390 nm). [e] 1.5 mL EtOAc). [f] 6.0 mL EtOAc). [g] 0.5 mol% photocatalyst. [h] 10 w light intensity. [i] no light.

Table S2. The optimization of the loading of photocatalyst and reaction time.

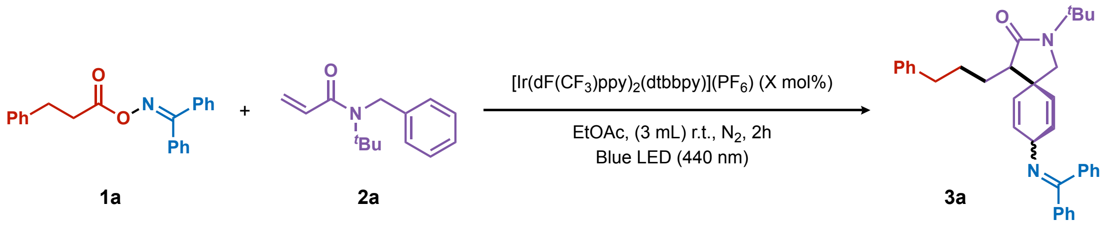

Entry	[Ir(dFCF$_3$ppy)$_2$(dtbbpy)]PF$_6$	Reaction time	Yield of 3a (%)	dr	Conversion 1a (%)	Conversion 2a (%)
1	0.5 mol%	4 h	54	1.4:1	100	>95
2	0.25 mol%	4 h	56	1.4:1	100	>95

3	0.125 mol%	4 h	42	1.4:1	54	81
4	0.25 mol%	1 h	47	1.4:1	67	91
5	0.25 mol%	2 h	53	1.4:1	77	93

Reaction conditions: **1a** (0.3 mmol, 1.5 equiv.), **2a** (0.2 mmol, 1.0 equiv.), $[Ir(dFCF_3ppy)_2(dtbbpy)]PF_6$, EtOAc (3 mL), N_2, blue LEDs (440 nm), room temperature. [b]Yields and dr were determined by ^1H NMR using 4-nitrobenzonitrile as the internal standard.

Table S3. The optimization of the ratio of **1a** and **2a**.

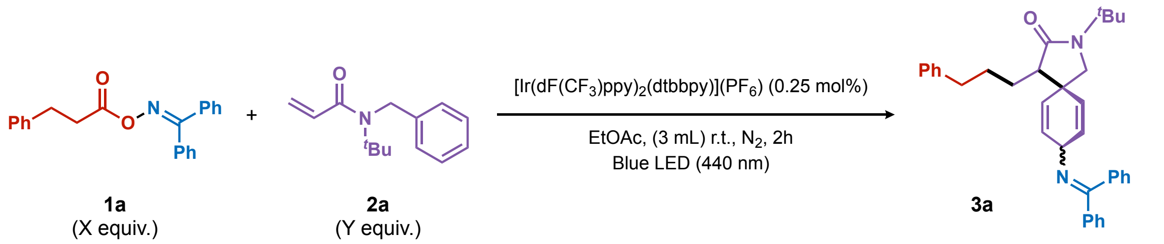

Entry	1a (mmol)	2a (mmol)	Yield of 3a (%)	dr	Conversion of 1a (%)	Conversion of 2a (%)
1	0.3	0.2	53	1.4:1	77	93
2	0.2	0.2	42	1.4:1	94	74
3	0.2	0.3	46	1.4:1	100	44
4	0.2	0.4	44	1.4:1	100	28

Reaction conditions: **1a**, **2a**, $[Ir(dFCF_3ppy)_2(dtbbpy)]PF_6$ (0.25 mol%), EtOAc (3 mL), N_2, blue LEDs (440 nm), room temperature, 2 h. Yields and dr were determined by ^1H NMR using 4-nitrobenzonitrile as the internal standard.

Table S4. The optimization of additives.

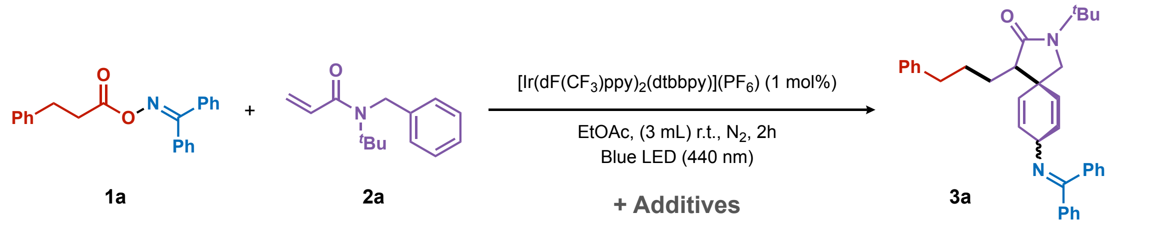

Entry	Additives (equiv.)	Reaction time	Yield of 3a (%)	dr	Conversion 1a (%)	Conversion 2a (%)
1	$NBu_4OPO(OtBu)_2$ (0.2)	1 h	49	1.4:1	73	81
2	Collidine (1.0)	1 h	49	1.4:1	100	91
3	NaOAc (1.0)	1 h	50	1.4:1	100	95
4	$Sc(OTf)_3$ (0.2)	2 h	50	1.4:1	86	>95
5	$CuBr_2$ (0.2)	2 h	11	1.4:1	42	35

Reaction conditions: **1a** (0.3 mmol, 1.5 equiv.), **2a** (0.2 mmol, 1.0 equiv.), $[Ir(dFCF_3ppy)_2(dtbbpy)]PF_6$ (1 mol%), EtOAc (3 mL), N_2, blue LEDs (440 nm), room temperature. Yields and dr were determined by ^1H NMR using 4-nitrobenzonitrile as the internal standard.

Table S5. The optimization of light intensity.

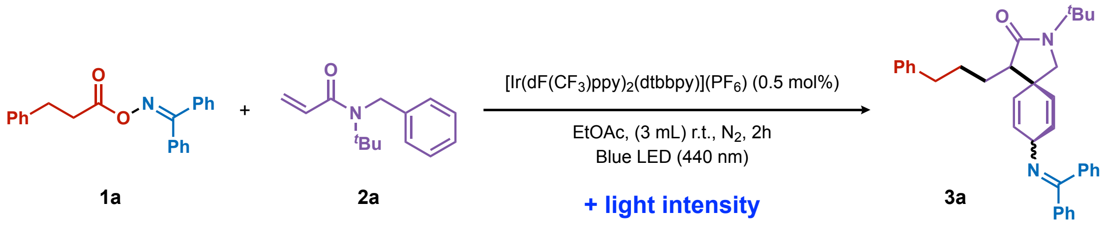

Entry	Light intensity	Reaction time	Yield 3a (%)	dr	Conversion 1a (%)	Conversion 2a (%)
1	10 w	2 h	56	1.4:1	77	>95
2	20 w	2 h	56	1.4:1	78	>95
3	30 w	2 h	53	1.4:1	83	>95
4	40 w	2 h	51	1.4:1	84	>95

Reaction conditions: **1a** (0.3 mmol, 1.5 equiv.), **2a** (0.2 mmol, 1.0 equiv.), Ir(dFCF$_3$ppy)$_2$(dtbbpy)]PF$_6$ (0.5 mol%), EtOAc (3 mL), N_2, blue LEDs (440 nm), room temperature. Yields and dr were determined by ^{1}H NMR using 4-nitrobenzonitrile as the internal standard.

Table S6. The optimization of time and temperature

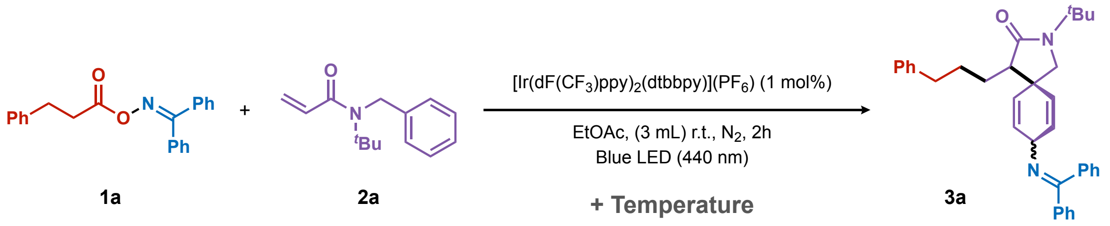

Entry	Light intensity	Reaction time	Temperature	Yield 3a (%)	dr	Conversion 1a (%)	Conversion 2a (%)
1	40 w	10 min	r.t.	37	1.4:1	54	69
2	40 w	20 min	r.t.	49	1.4:1	78	79
3	40 w	2 h	r.t.	54	1.4:1	100	>95
4	10 w	2 h	r.t.	54	1.4:1	100	>95
5	10 w	4 h	5 ℃	59	1.4:1	100	>95
6	10 w	12 h	-10℃	62	1.4:1	100	>95

Reaction conditions: **1a** (0.3 mmol, 1.5 equiv.), **2a** (0.2 mmol, 1.0 equiv.), Ir(dFCF$_3$ppy)$_2$(dtbbpy)]PF$_6$ (1 mol%), EtOAc (3 mL), N_2, blue LEDs (440 nm). Yields and dr were determined by ^{1}H NMR using 4-nitrobenzonitrile as the internal standard.

General procedures for catalytic spirocyclization / imination

General procedure A for the two-component dearomative spirocyclization/imination of nonactivated arenes via photocatalytic radical relay

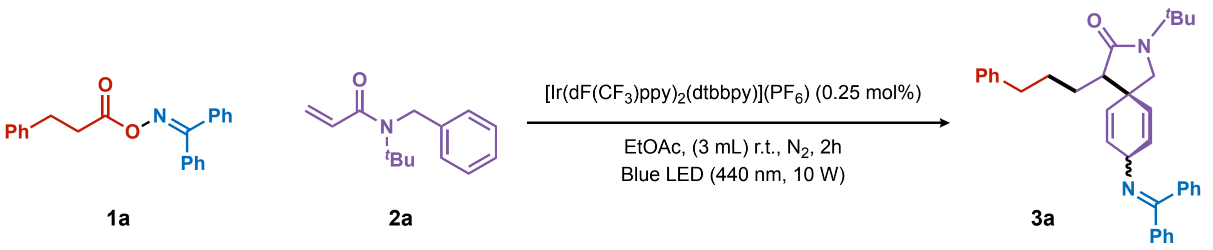

A 10 mL Pyrex tube equipped with a magnetic stir bar was charged with oxime **1a** (0.3 mmol), *N*-benzyl-*N*-(*tert*-butyl)acrylamide **2a** (0.2 mmol), $[Ir(dFCF_3ppy)_2(dtbbpy)]PF_6$ (0.001 mmol) in EtOAc (3 mL). After the degassing with N_2 for 10 mins, the mixture was irradiated by blue LEDs (λ = 440 nm, 10 W) for 2 hours at room temperature. After irradiation, the resulting homogenous solution was transferred to a 25 mL round bottom flask with aid of CH_2Cl_2 (2 x 3 mL). NEt_3 (approx. 0.5 mL) and SiO_2 were added to this solution and the volatiles were removed under reduced pressure, affording a powder which was loaded on column. Purification by column chromatography using pre-basified silica (NEt_3) with PE/EtOAc as eluent afforded the target product **3a**.

General procedure B for the three-component dearomative spirocyclization/imination of nonactivated arenes with non-activated alkenes

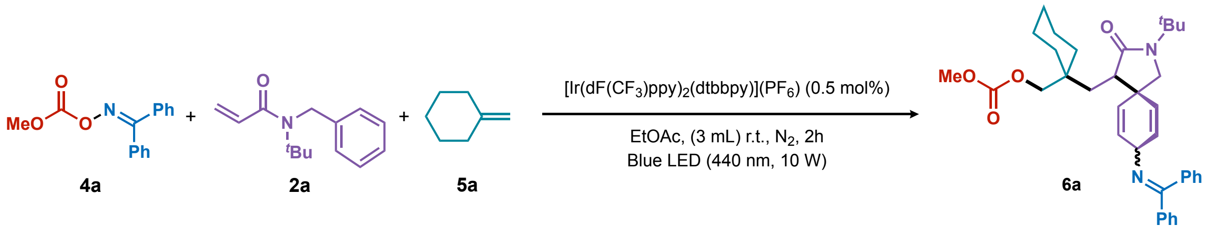

A 10 mL Pyrex tube equipped with a magnetic stir bar was charged with oxime **4a** (0.3 mmol), *N*-benzyl-*N*-(*tert*-butyl)acrylamide **2a** (0.2 mmol), $[Ir(dFCF_3ppy)_2(dtbbpy)]PF_6$ (0.001 mmol) in EtOAc (3 mL). The non-activated alkenes **5a** (0.4 mmol) was added into the mixture after the degassing with N_2 for 10 mins. Then the mixture was irradiated by blue LEDs (λ = 440 nm, 10 W) for 4 hours at room temperature. After irradiation, the resulting homogenous solution was transferred to a 25 mL round bottom flask with aid of CH_2Cl_2 (2 x 3 mL). NEt_3 (approx. 0.5 mL) and SiO_2 were added

to this solution and the volatiles were removed under reduced pressure, affording a powder which was loaded on column. Purification by column chromatography using pre-basified silica (NEt_3) with PE/EtOAc as eluent afforded the target product **6a**.

General procedure C for the three-component dearomative spirocyclization/imination of nonactivated arenes with non-activated alkanes

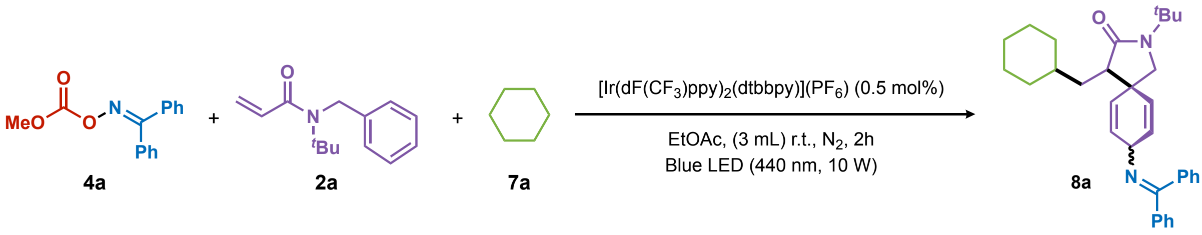

A 10 mL Pyrex tube equipped with a magnetic stir bar was charged with oxime **4a** (0.4 mmol), *N*-benzyl-*N*-(*tert*-butyl)acrylamide **2a** (0.2 mmol), cyclohexane **7a** (2 mL)., $[Ir(dFCF_3ppy)_2(dtbbpy)]PF_6$ (0.001 mmol) in EtOAc (1 mL). After the degassing with N_2 for 10 mins, the mixture was irradiated by blue LEDs (λ = 440 nm, 10 W) for 4 hours at room temperature. After irradiation, the resulting homogenous solution was transferred to a 25 mL round bottom flask with aid of CH_2Cl_2 (2 x 3 mL). NEt_3 (approx. 0.5 mL) and SiO_2 were added to this solution and the volatiles were removed under reduced pressure, affording a powder which was loaded on column. Purification by column chromatography using pre-basified silica (NEt_3) with PE/EtOAc as eluent afforded the target product **8a**.

General procedure D for the flow reactions

A 100 mL round bottom flask was loaded with the corresponding oxime (6 mmol), *N*-benzyl-*N*-(*tert*-butyl)acrylamide (4 mmol), $[Ir(dFCF_3ppy)_2(dtbbpy)]PF_6$ (0.02 mmol) in EtOAc (60 mL). The mixture was bubbled with a stream of argon for 20 minutes (for three-component reaction, 8.0 mmol methylenecyclohexane was added after the degassing), then pumped to a homemade flow reactor at a rate of 0.05 mL/min upon the irradiation of 10 W LEDs (λ = 440 nm). After irradiation, the resulting homogenous solution was transferred to a 250 mL round bottom flask with aid of CH_2Cl_2 (2 x 10 mL). NEt_3 (approx. 5 mL) and SiO_2 were added to this solution and the volatiles

were removed under reduced pressure, affording a powder which was loaded on column. Purification by column chromatography using pre-basified silica (NEt_3) with PE/EtOAc as eluent afforded the target product.

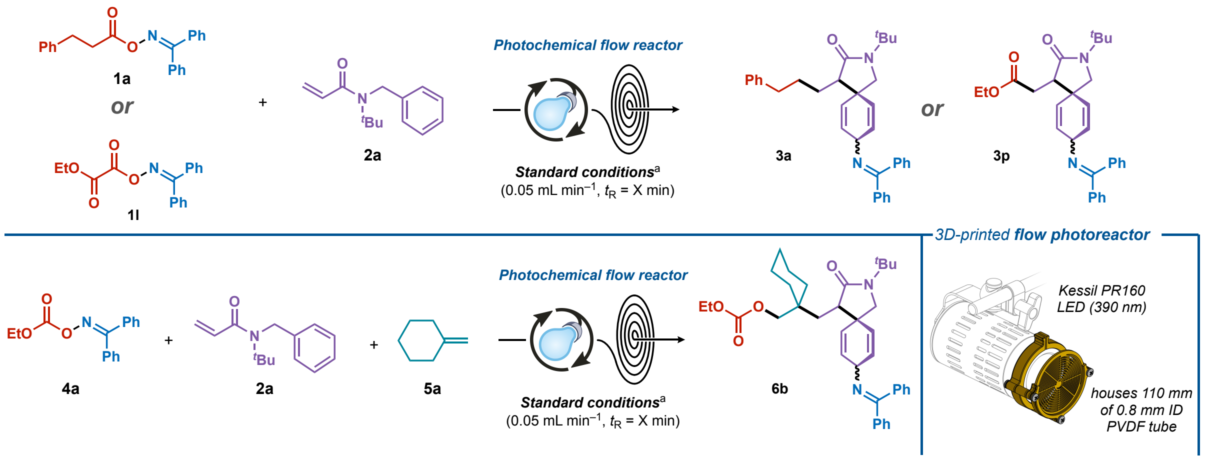

General procedure E for the hydrolysis of spiro-imines

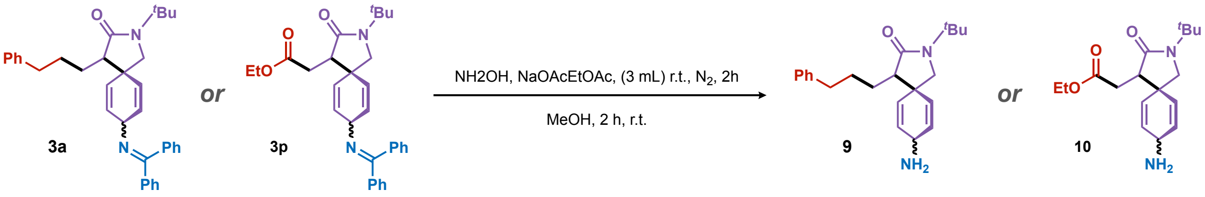

To a solution of the spiro-imines **3a** or **3p** (0.4 mmol) in MeOH (4 mL) at room temperature was added NaOAc (0.96 mmol, 2.4 equiv.) and hydroxylamine hydrochloride (0.72 mmol, 1.8 equiv.). After 2 hours, the solution was then partitioned between 0.1 M NaOH and CH_2Cl_2. The organic layer was dried over anhydrous Na_2SO_4 and evaporated in vacuo. The crude product was purified by flash chromatography using silica gel to give the corresponding product **9** and **10**.

4. Mechanistic studies

Fluorescence quenching studies

The steady-state fluorescence quenching studies were performed on FS5 spectrofluorometer (Edinburgh Instruments) using 10 × 10 mm quartz cuvettes. All measurements were carried out in ethyl acetate (HPLC grade) under Ar atmosphere at room temperature (ca. 20 °C). The emission spectra were collected at 440–750 nm with excitation at 420 nm.

The measurements were performed on a series of solutions containing quenchers **1a**, **2a**, **4a** or **5a** (0–10 mM) and the Ir-photocatalyst ([Ir(dF(CF_3)ppy)$_2$(dtbbpy)](PF_6), 15 µM) (Figure **S1–S4**). Each solution (3 mL) was prepared by mixing appropriate volumes of ethyl acetate (0–2.7 mL) with stock solutions of the Ir-photocatalyst (0.3 mL of 0.15 mM stock solution) and the quencher (0–2.7 mL of 11.1 mM stock solution). The Stern-Volmer quenching constants (K_{SV}) were calculated from linear regression in the plots of normalized emission *vs.* concentration for each of the quenchers at 477 nm (Figure **S5**): K_{SV} = 313.4 M^{-1} for **1a**, K_{SV} = 13.9 M^{-1} for **2a**, K_{SV} = 274.2 M^{-1} for **4a**, K_{SV} = 11.4 M^{-1} for **5a**.

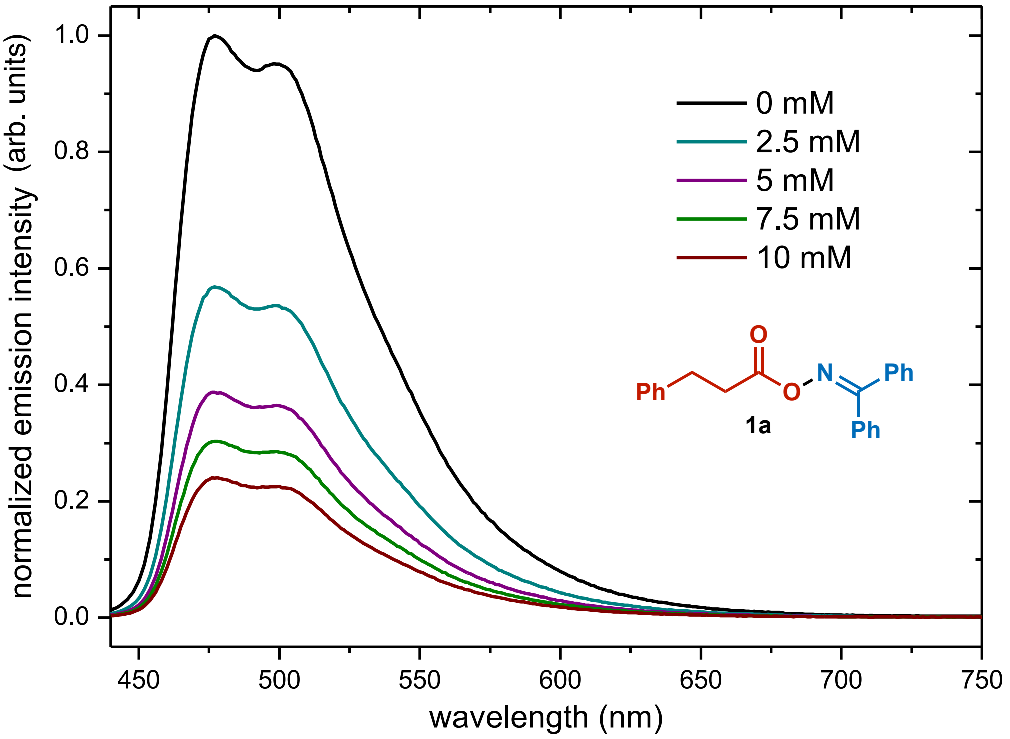

Figure S1. Fluorescence quenching measurements for 15 µM Ir-photocatalyst with **1a** (0–10 mM).

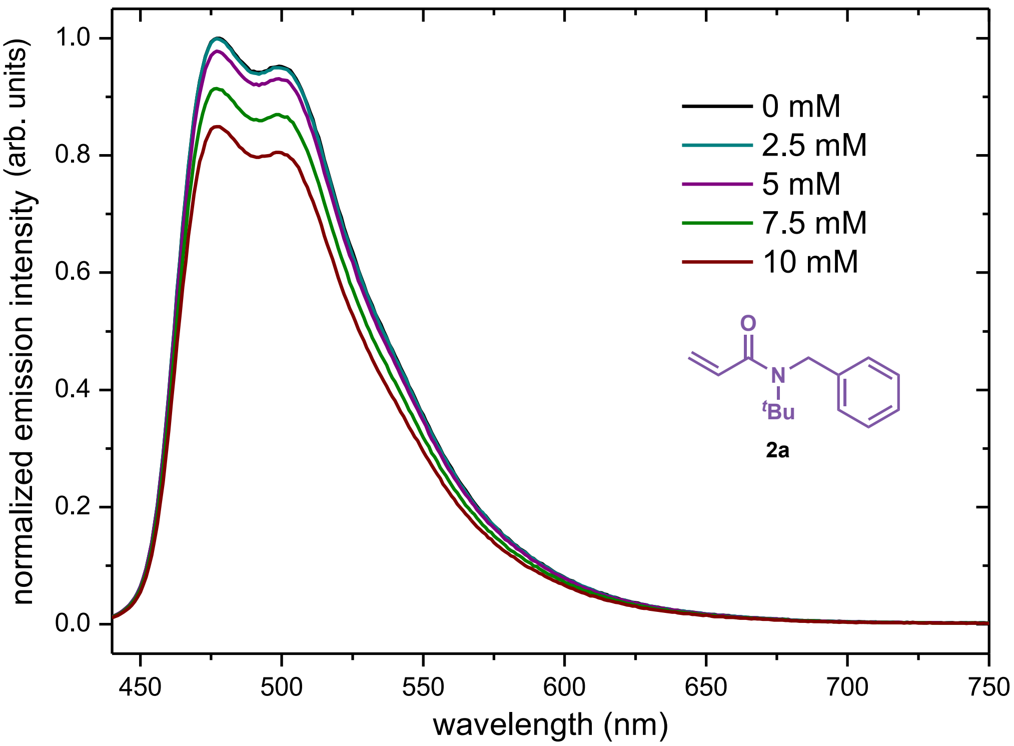

Figure S2. Fluorescence quenching measurements for 15 μM Ir-photocatalyst with **2a** (0–10 mM).

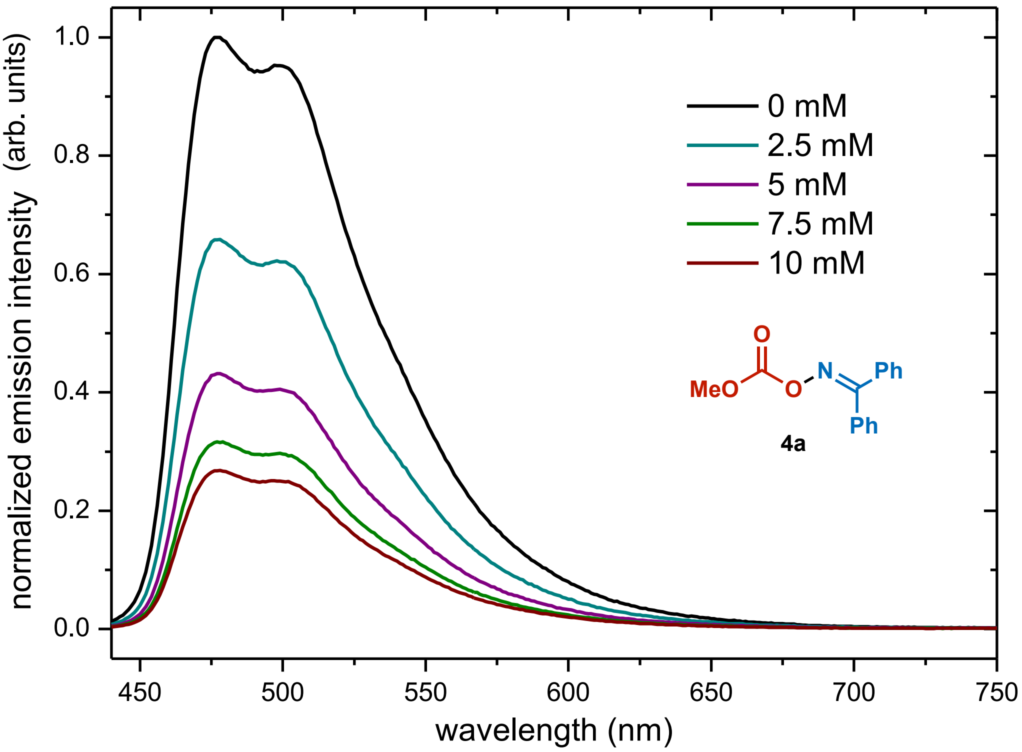

Figure S3. Fluorescence quenching measurements for 15 μM Ir-photocatalyst with **4a** (0–10 mM).

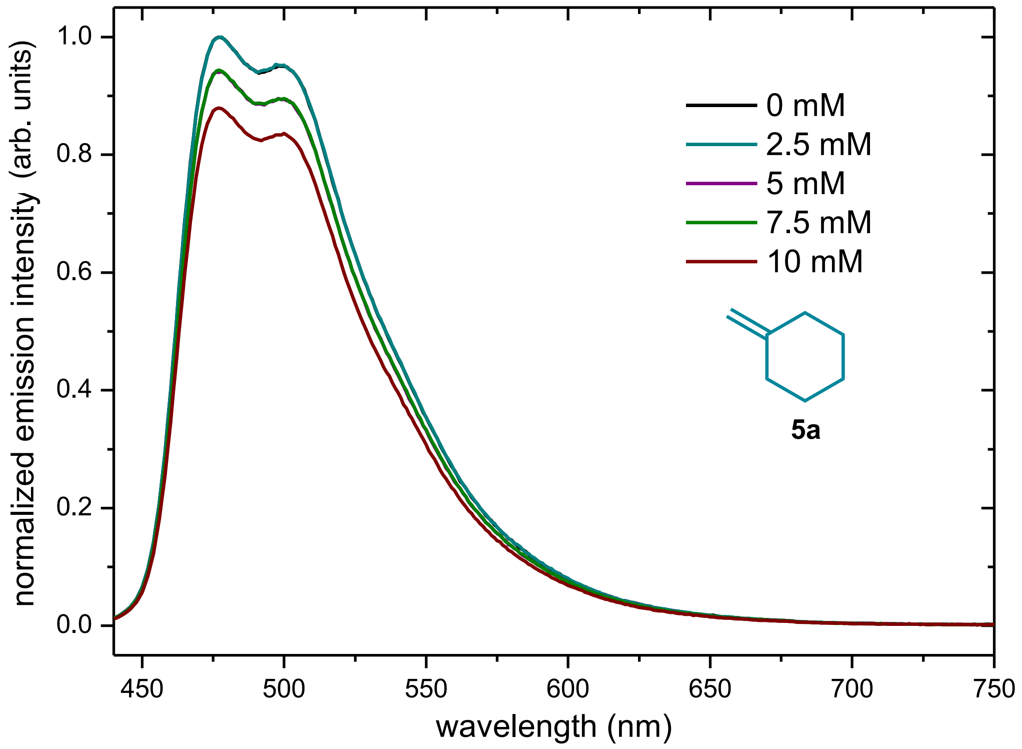

Figure S4. Fluorescence quenching measurements for 15 μM Ir-photocatalyst with **5a** (0–10 mM).

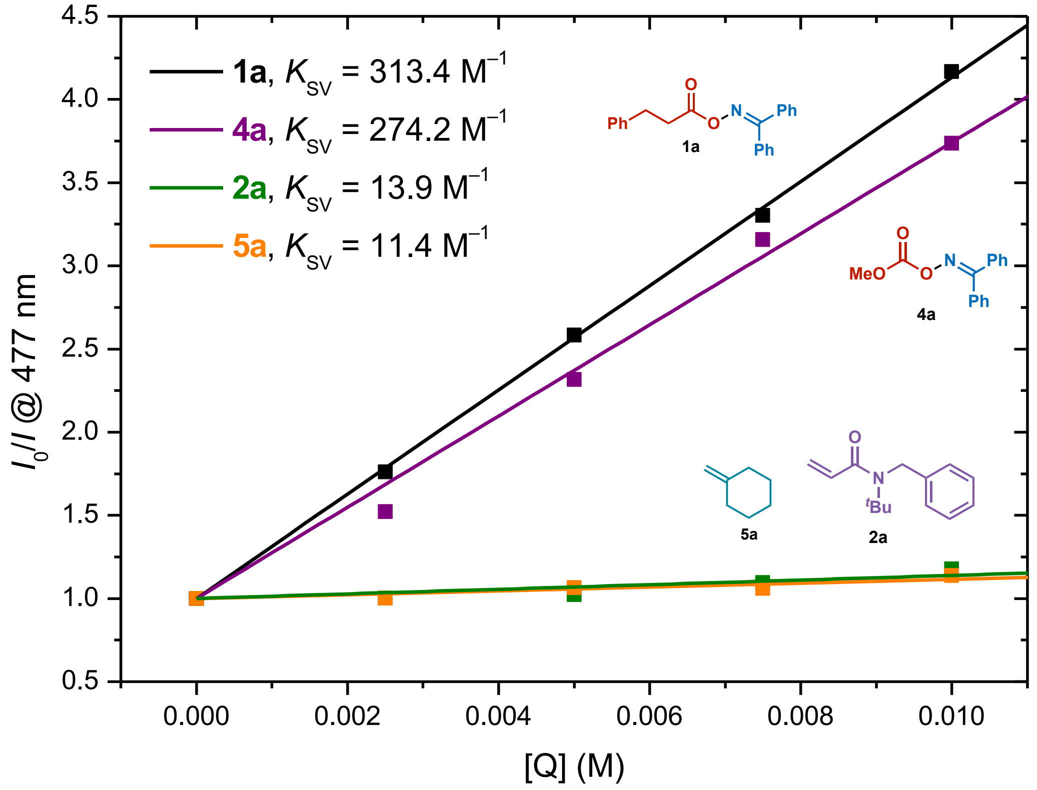

Figure S5. Stern-Volmer plot for 15 μM Ir-photocatalyst with **1a**, **2a**, **4a**, and **5a** quenchers.

Detection and trapping of intermediates

Detection of side-products using HRMS

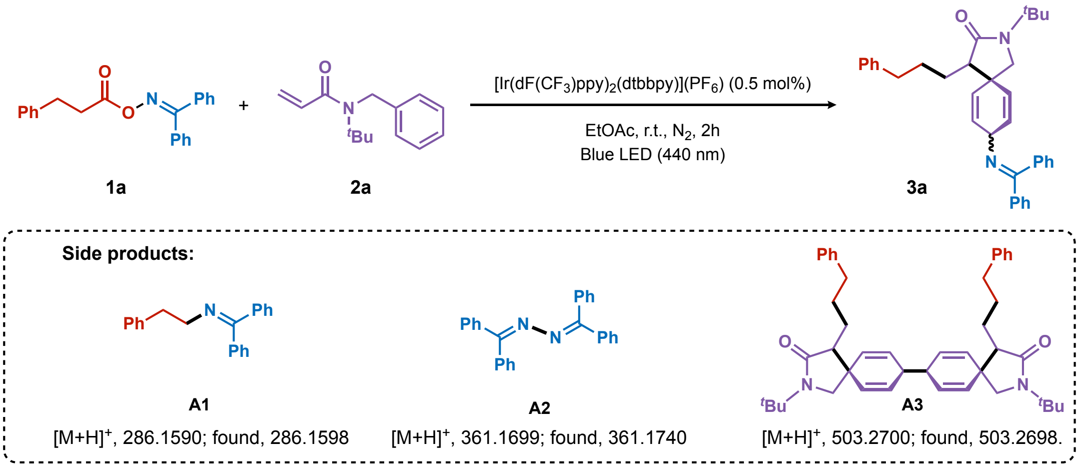

Trapping experiment using TEMPO

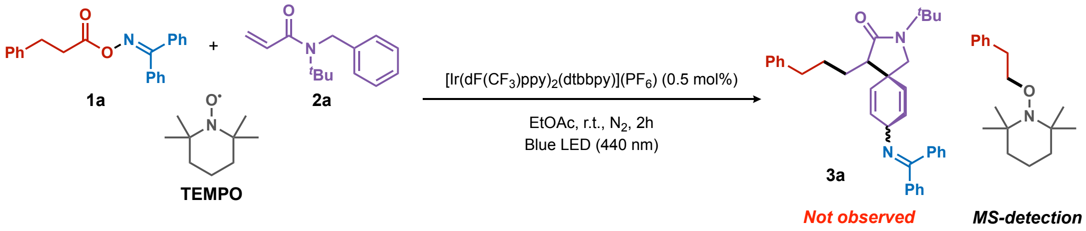

Figure S6. LC-MS spectra for the two-component dearomative spirocyclization/imination of nonactivated arenes.

Light on-off experiment

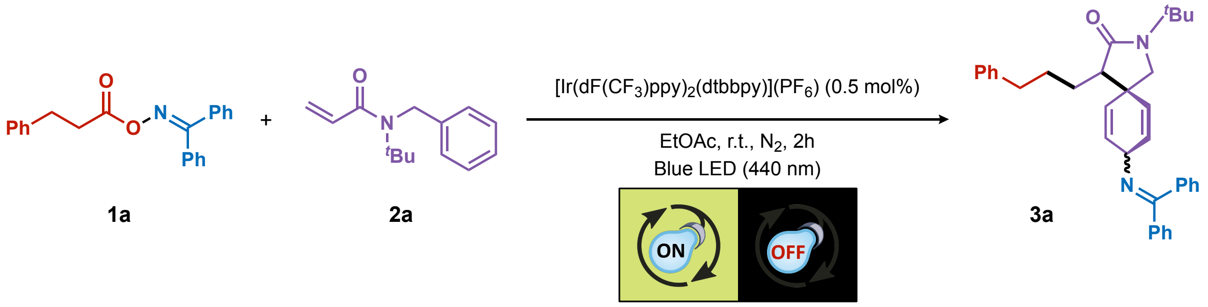

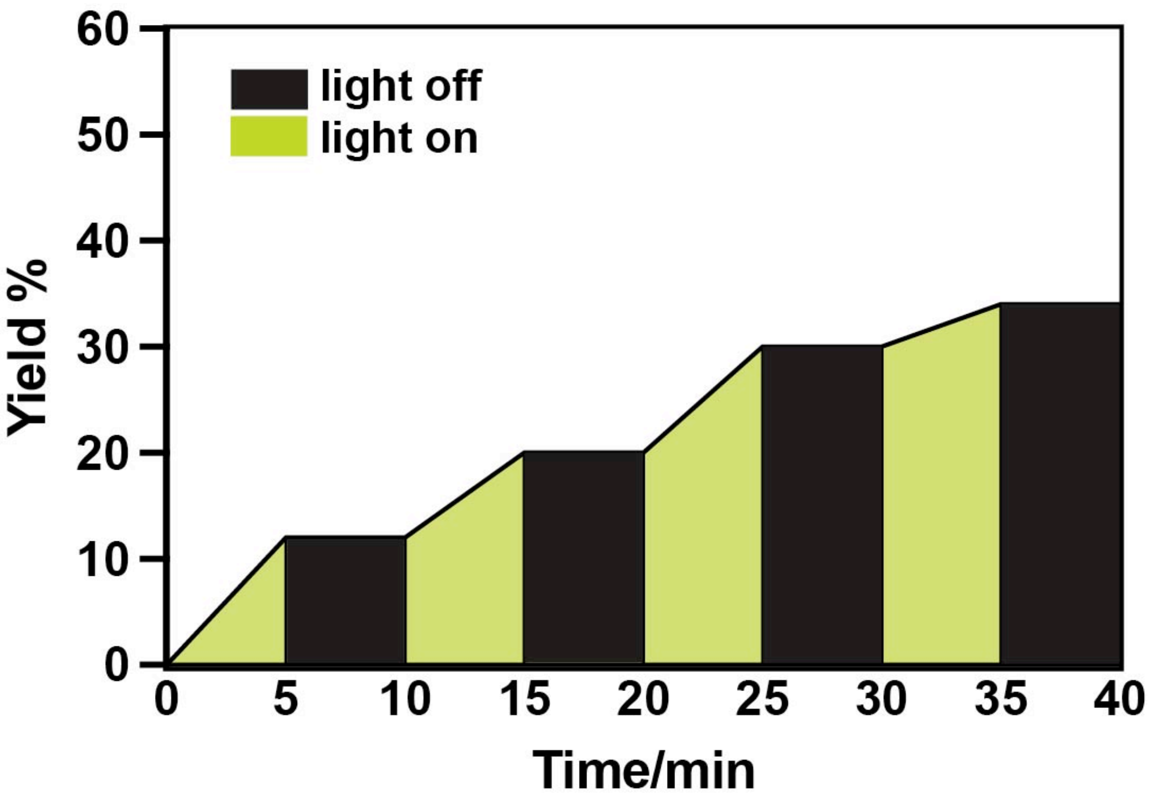

Figure S7. Light on-off experiment. General procedure: eight 10 mL Pyrex tubes equipped with a magnetic stir bar were separately charged with oxime **1a** (0.3 mmol), *N*-benzyl-*N*-(*tert*-butyl)acrylamide **2a** (0.2 mmol), [Ir(dFCF$_3$ppy)$_2$(dtbbpy)]PF$_6$ (0.001 mmol) in EtOAc (3 mL). After the degassing with N_2 for 10 mins, the reactions were placed in light and dark in every alternative 5 minutes. Then the resulting homogenous solutions were transferred to a 25 mL round bottom flask. Yields were determined by ^{1}H NMR using 4-nitrobenzonitrile as the internal standard.

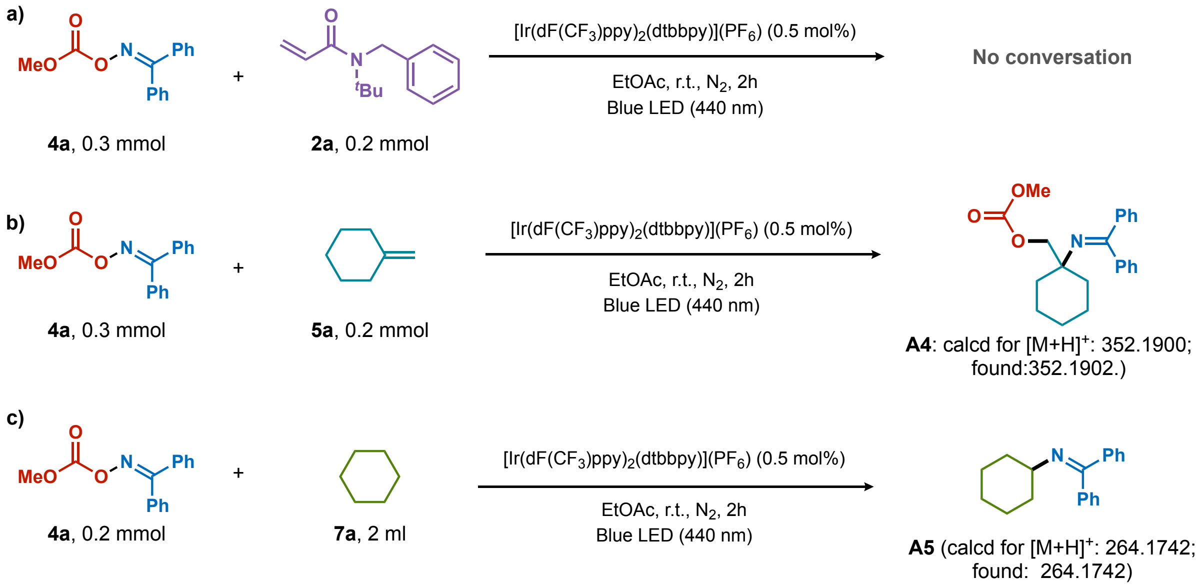

Figure S8. Control experiments for reactions with carbonate oximes.

5. Computational studies

Computational details

All stationary points were optimized at the B3LYP/6-311+G(d,p) level of theory, as implemented in Gaussian 16 Rev D.01. In the optimizations, the Grimme correction for dispersion (D3) was used in combination with the Conductor-like Polarizable Continuum Model (CPCM, Ethylacetate) using the parameters for Ethylacetate and the default Unified Force Field radii (UFF) as implemented in Gaussian 16 Rev D.01. All geometries were characterized as minima or saddle points on the potential-energy surface (PES) by using the sign of the eigenvalues of the force-constant matrix obtained from a frequency calculation. Transition states with one imaginary frequency were confirmed to describe the correct movement on the PES by mode analysis and by intrinsic reaction coordinate (IRC) calculations connecting the correct reactants and products. To examine the validity of the selected computational method, the structures obtained at the B3LYP-level of theory were subjected to a single-point calculation using M062X/6-311+G(d,p) and M06/6-311+G(d,p) with the Conductor-like Polarizable Continuum Model (CPCM, Ethylacetate) using the parameters for ethylacetate and the default Unified Force Field radii (UFF). The Gibbs free energies were obtained by adding the thermal correction to Gibbs Free Energy from the B3LYP level of theory. The comparison between relative free energies calculated with different functionals show that the major difference in the formation of the aliphatic and iminyl radicals is seen in the excitation energy from the ground state to the triplet state of the iridium photocatalyst. Both the B3LYP and the M06 functionals reproduces the experimental triplet energy (58 kcal mol^{-1} compared to 61.1 kcal mol^{-1}) while the M062X strongly overestimates excitation energy of the triplet state of the iridium photo catalyst. However, with the exception for the iridium photocatalyst excitation, both B3LYP, M062X and M06 functionals show a similar potential energy surface from the triplet **1a*** (see Figure S9A). In addition, the structures obtained at the B3LYP-level of theory for the dearomative spirocyclization/imination were subjected to a single-point calculation using M062X/6-311+G(d,p) with the Conductor-like Polarizable Continuum Model (CPCM, Ethylacetate) using the parameters for ethylacetate and the default Unified Force Field radii (UFF). The results show similar relative Gibbs free energy and barrier for the dearomative spirocyclization/imination for the two DFT methods (Figure S9B).

Gibbs free energy diagrams

A) Activation via energy transfer catalysis

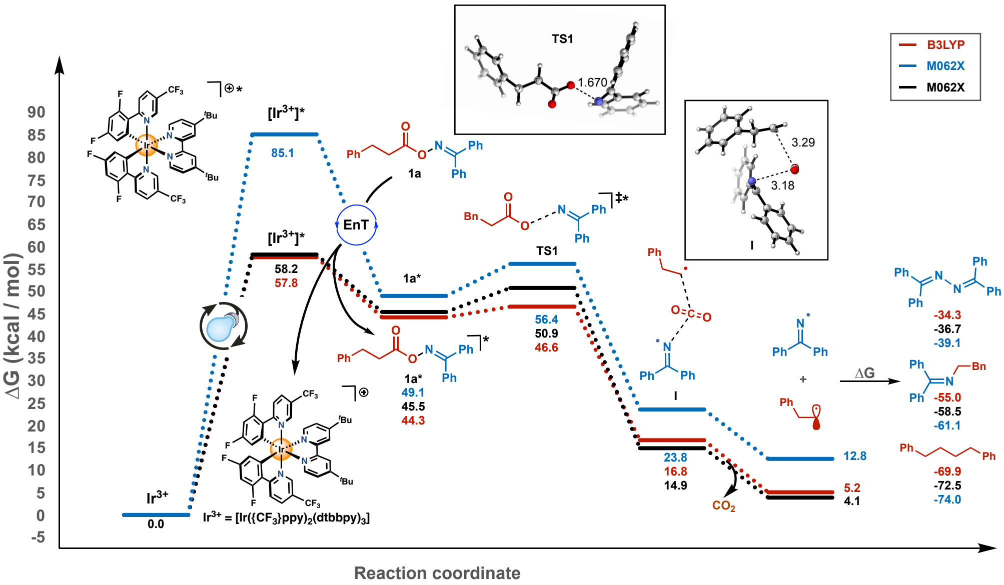

B) Gibbs free energy surface for dearomative spirocyclization/imination

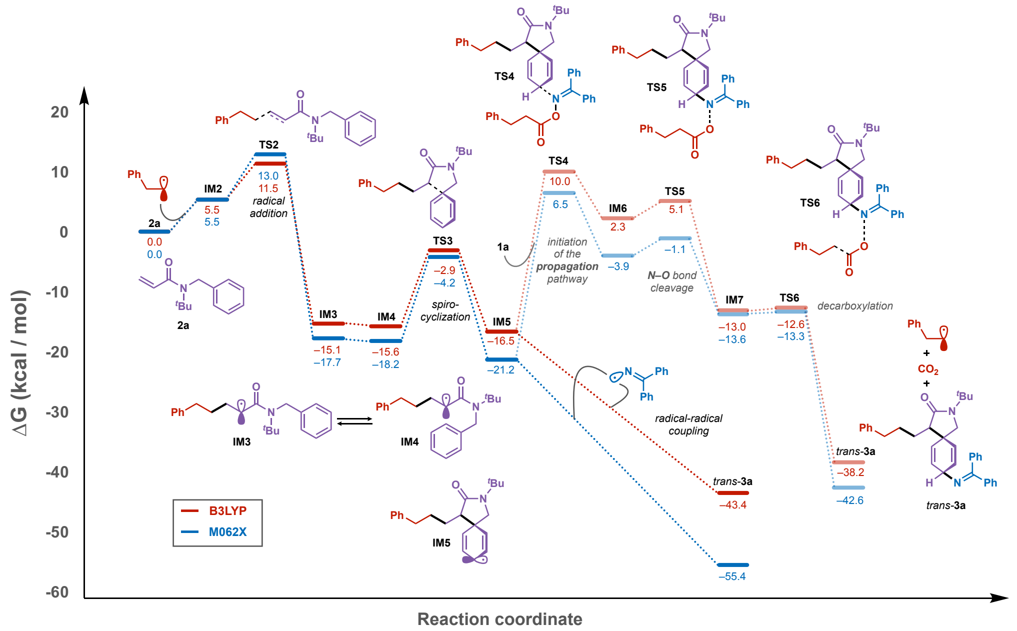

Figure S9. Gibbs free energy surface (**red**) for the formation of C- and N-centered radicals optimized at the B3LYP/6-311+G(d,p) level using the Grimme correction for dispersion (D3) and the Conductor-like Polarizable Continuum Model (CPCM, UFF, Ethylacetate). Gibbs free energies are given in kcal mol^{-1}. Values in **blue** and **black** refer to single-point energy calculations at the M062X or M06/6-311+G(d,p) level using the Conductor-like Polarizable Continuum Model (CPCM, UFF, Ethylacetate) with the addition of Gibbs free energy-corrections from the B3LYP level of theory. Gibbs free energies are given in kcal mol^{-1}.

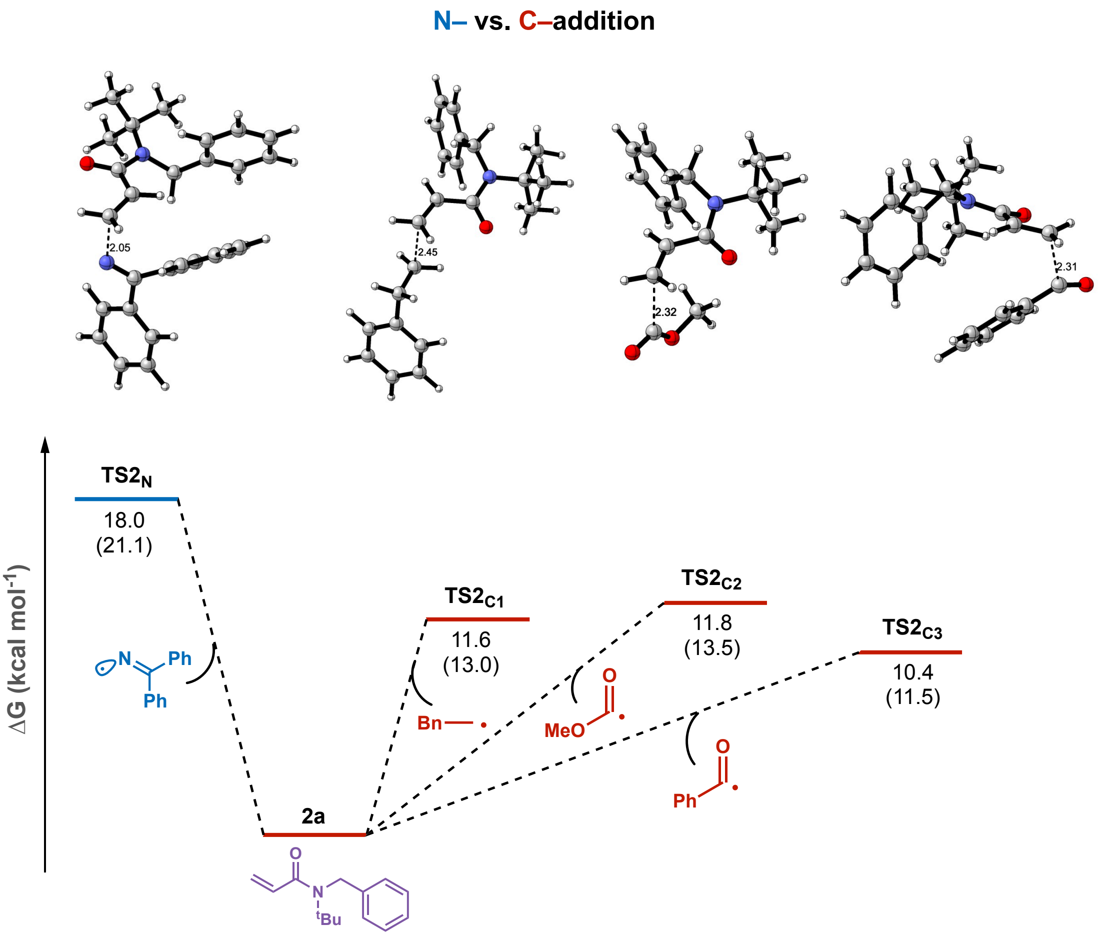

Figure S10. Comparison of Gibbs free energy for the addition of N-centered and C-centered radicals to **2a** optimized at the B3LYP/6-311+G(d,p) level using the Grimme correction for dispersion (D3) and the Conductor-like Polarizable Continuum Model (CPCM, UFF, Ethylacetate). Values in parenthesis refer to single-point energy calculations at the M062X/6-311+G(d,p) level using the Conductor-like Polarizable Continuum Model (CPCM, UFF, Ethylacetate) with the addition of Gibbs free energy-corrections from the B3LYP level of theory. Gibbs free energies are given in kcal mol^{-1}.

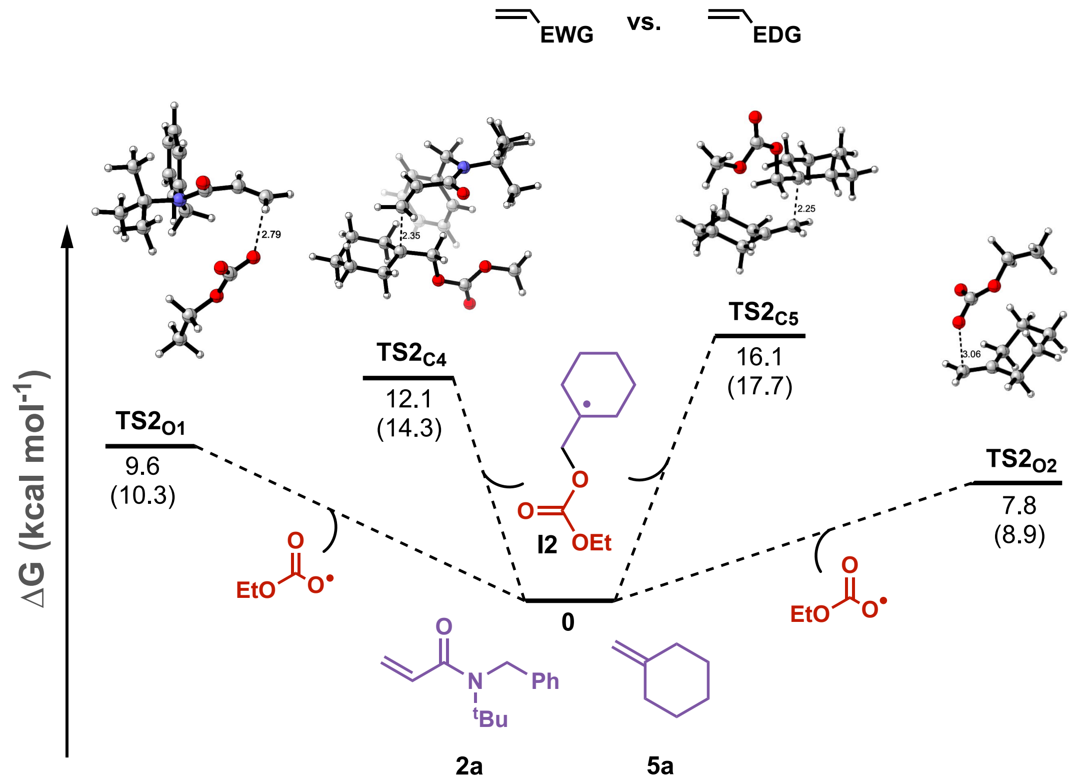

Figure S11. Comparison of Gibbs free energy for the addition of ethyl carbonate radical and C-centered radicals to **2a** and **5a** optimized at the B3LYP/6-311+G(d,p) level using the Grimme correction for dispersion (D3) and the Conductor-like Polarizable Continuum Model (CPCM, UFF, Ethylacetate). Values in parenthesis refer to single-point energy calculations at the M062X/6-311+G(d,p) level using the Conductor-like Polarizable Continuum Model (CPCM, UFF, Ethylacetate) with the addition of Gibbs free energy-corrections from the B3LYP level of theory. Gibbs free energies are given in kcal mol^{-1}.

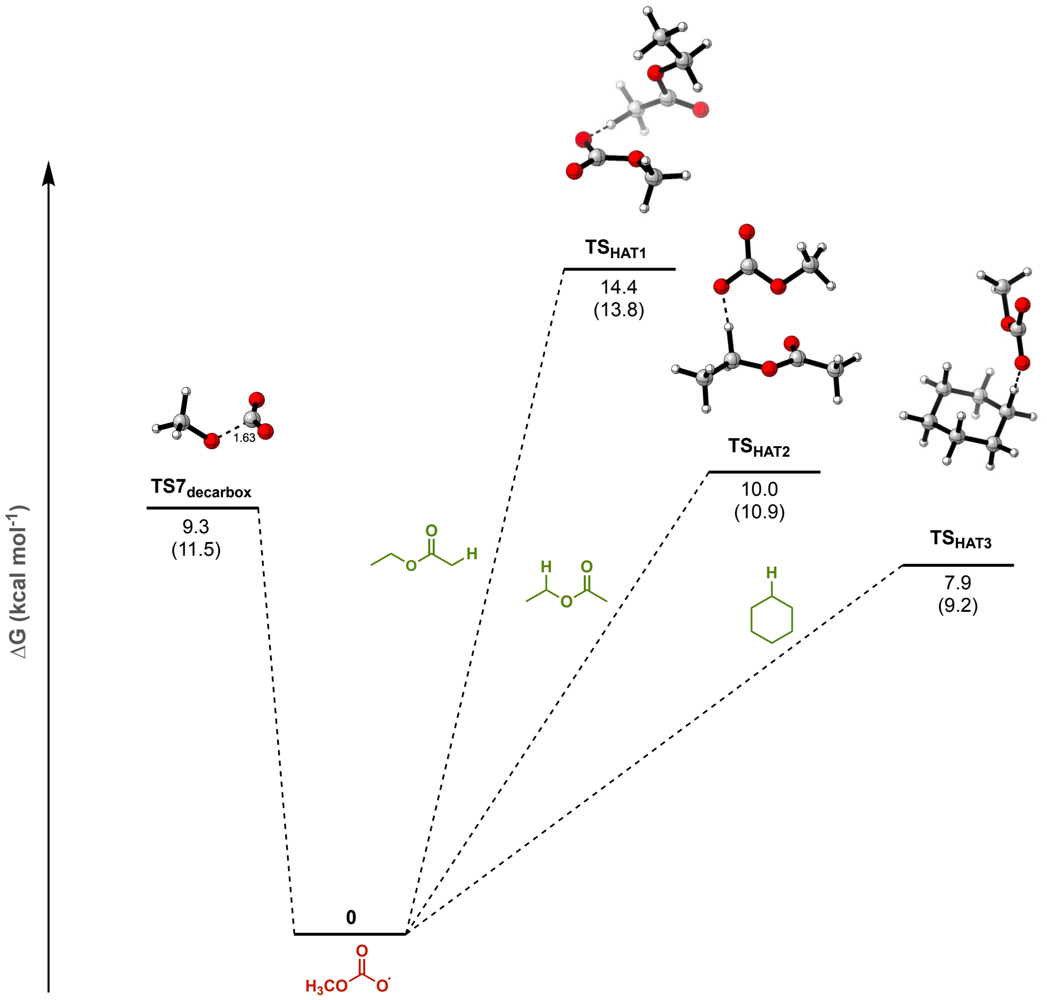

Figure S12. Comparison of Gibbs free energy for the decarboxylation and hydrogen atom transfer of cyclohexane and ethyl acetate for methyl carbonate radical optimized at the B3LYP/6-311+G(d,p) level using the Grimme correction for dispersion (D3) and the Conductor-like Polarizable Continuum Model (CPCM, UFF, Ethylacetate). Values in parenthesis refer to single-point energy calculations at the M062X/6-311+G(d,p) level using the Conductor-like Polarizable Continuum Model (CPCM, UFF, Ethylacetate) with the addition of Gibbs free energy-corrections from the B3LYP level of theory. Gibbs free energies are given in kcal mol^{-1}.

6. Characterization data for substrates and products

2-(4-(4-chlorobenzoyl)phenoxy)-1-(((diphenylmethylene)amino)oxy)-2-methylpropan-1-one (1x).

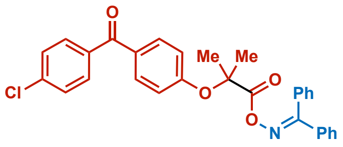

The product was obtained from *fenofibric acid* according to ***Method A*** as a white solid (isolated yield: 60%). R_f 0.47 (hexane/EtOAc 4:1). ^{1}H NMR (500 MHz, $CDCl_3$): δ 7.76 – 7.65 (m, 4H), 7.61 – 7.55 (m, 2H), 7.48 – 7.42 (m, 3H), 7.41 – 7.30 (m, 5H), 7.19 – 7.10 (m, 2H), 6.83 – 6.74 (m, 2H), 1.58 (s, 6H). ^{13}C NMR (126 MHz, $CDCl_3$) δ 194.3, 170.8, 167.1, 159.5, 136.5, 134.3, 132.6, 132.1, 131.4, 131.3, 130.6, 130.2, 129.8, 129.3, 128.7, 128.6, 128.4, 128.2, 117.7, 79.2, 25.5. HRMS (ESI-TOF, *m/z*): calcd for $C_{30}H_{24}ClNO_4$ [M + Na]$^+$, 520.1286; found, 520.1293.

Diphenylmethanone O-(2-(4-(2,2-dichlorocyclopropyl)phenoxy)-2-methylpropanoyl) oxime (1y).

The product was obtained from *ciprofibrate* according to ***Method A*** as colorless oil (isolated yield: 80%). R_f 0.40 (hexane/EtOAc 4:1). ^{1}H NMR (500 MHz, $CDCl_3$): δ 7.66 – 7.52 (m, 2H), 7.48 – 7.43 (m, 1H), 7.42 – 7.28 (m, 5H), 7.15 – 7.02 (m, 4H), 6.84 – 6.74 (m, 2H), 2.83 (dd, *J* = 10.7, 8.3 Hz, 1H), 1.94 (dd, *J* = 10.7, 7.4 Hz, 1H), 1.78 (dd, *J* = 8.3, 7.4 Hz, 1H), 1.50 (s, 6H). ^{13}C NMR (126 MHz, $CDCl_3$) δ 171.3, 166.7, 154.9, 134.5, 132.3, 131.3, 129.8, 129.7, 129.3, 128.8, 128.6, 128.4, 128.2, 119.0, 79.0, 61.0, 35.0, 26.0, 25.5, 25.4. HRMS (ESI-TOF, *m/z*): calcd for $C_{26}H_{23}Cl_2NO_3$ [M + Na]$^+$, 490,0947; found, 490.0952.

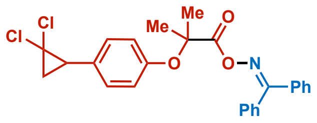

(2S,5R)-2-((((diphenylmethylene)amino)oxy)carbonyl)-3,3-dimethyl-4-thia-1-azabicyclo[3.2.0]-heptan-7-one 4,4-dioxide (1aa).

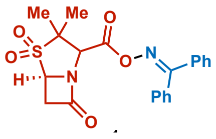

The product was obtained from *sulbactam* according to ***Method A*** as colorless foam (isolated yield: 78%). R_f 0.21 (hexane/EtOAc 3:1). ^{1}H NMR (500 MHz, $CDCl_3$): δ 7.65 – 7.58 (m, 2H), 7.54 – 7.46 (m, 4H), 7.43 – 7.35 (m, 2H), 7.32 – 7.27 (m, 2H), 4.46 (dd, *J* = 4.2, 2.2 Hz, 1H), 4.33 (s, 1H), 3.43 (dd, *J* = 16.2, 4.2 Hz, 1H), 3.38 (dd, *J* = 16.2, 2.2 Hz, 1H), 1.34 (s, 3H), 1.17 (s, 3H). ^{13}C NMR (126 MHz, $CDCl_3$) δ 170.5, 167.3, 164.2, 133.5, 132.5, 131.8, 130.0, 129.1, 128.7, 128.74, 128.2, 62.71, 62.6, 61.1, 38.5, 20.5, 18.0. HRMS (ESI-TOF, *m/z*): calcd for $C_{21}H_{20}N_2O_5S$ [M + Na]$^+$, 435.0985; found, 435.0992.

(1r,4R)-*N*-((*R*)-1-(((diphenylmethylene)amino)oxy)-1-oxo-3-phenylpropan-2-yl)-4-isopropyl-cyclohexane-1-carboxamide (1ab).

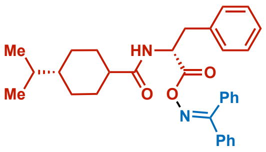

The product was obtained from *nateglinide* according to ***Method A*** as a white solid (isolated yield: 60%). R_f 0.34 (hexane/EtOAc 4:1). ^{1}H NMR (500 MHz, $CDCl_3$): δ 7.86 – 7.72 (m, 4H), 7.63 – 7.53 (m, 2H), 7.49 (t, *J* = 7.7 Hz, 4H), 7.32 (dd, *J* = 8.0, 6.4 Hz, 3H), 7.21 – 7.11 (m, 2H), 5.81 (d, *J* = 6.7 Hz, 1H), 4.79 (q, *J* = 6.9 Hz, 1H), 3.27 (dd, *J* = 14.2, 5.6 Hz, 1H), 3.14 (dd, *J* = 14.2, 7.0 Hz, 1H), 2.00 (ddd~tt, *J* = 12.1, 3.5 Hz, 1H), 1.91 – 1.71 (m, 4H), 1.43 – 1.28 (m, 3H), 1.05 – 0.92 (m, 3H), 0.84 (d, *J* = 6.9 Hz, 6H). HRMS (ESI-TOF, *m/z*): calcd for $C_{32}H_{36}N_2O_3$ $[M + Na]^+$, 519.2618; found, 519.2620.

***N*-(2-(((diphenylmethylene)amino)oxy)-2-oxoethyl)-4-methylbenzenesulfonamide (1ac).**

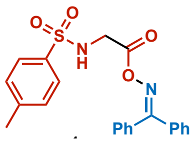

The product was obtained from *N-p-tosylglycine* according to ***Method A*** as colorless oil (isolated yield: 90%). R_f 0.60 (hexane/EtOAc 2:1). ^{1}H NMR (500 MHz, $CDCl_3$): δ 7.78 – 7.65 (m, 2H), 7.57 – 7.41 (m, 6H), 7.37 (t, *J* = 7.7 Hz, 2H), 7.30 (d, *J* = 7.8 Hz, 2H), 7.26 – 7.20 (m, 2H), 5.06 (t, *J* = 5.3 Hz, 1H), 3.91 (d, *J* = 5.3 Hz, 2H), 2.42 (s, 3H). ^{13}C NMR (126 MHz, $CDCl_3$) δ 167.7, 166.1, 144.1, 136.2, 134.2, 131.9, 131.5, 130.2, 130.0, 129.2, 128.9, 128.7, 128.5, 127.4, 43.6, 21.7. HRMS (ESI-TOF, *m/z*): calcd for $C_{22}H_{20}N_2O_4S$ $[M + Na]^+$, 431,1036; found, 431.1043.

Diphenylmethanone O-(2,2,5,5-tetramethyltetrahydro-7H-[1,3]dioxolo[4',5':4,5]furo[3,2-d][1,3]-dioxine-8a-carbonyl) oxime (1ad).

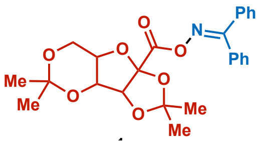

The product was obtained from *diprogulic acid* according to ***Method A*** as colorless foam (isolated yield: 21%). R_f 0.41 (hexane/EtOAc 2:1). ^{1}H NMR (500 MHz, $CDCl_3$): δ 7.65 – 7.57 (m, 2H), 7.51 – 7.40 (m, 6H), 7.40 – 7.31 (m, 2H), 4.77 (s, 1H), 4.29 (d, *J* = 2.3 Hz, 1H), 4.14 (q, *J* = 2.0 Hz, 1H), 4.09 – 3.96 (m, 2H), 1.47 (s, 3H), 1.41 (s, 3H), 1.33 (s, 3H), 1.26 (s, 3H). ^{13}C NMR (126 MHz, $CDCl_3$) δ 166.8, 164.3, 134.9, 132.2, 131.1, 130.0, 129.8, 129.6, 128.5, 128.2, 114.5, 110.3, 97.7, 88.0, 74.2, 72.6, 60.0, 28.8, 27.1, 25.7, 19.1. HRMS (ESI-TOF, *m/z*): calcd for $C_{25}H_{27}NO_7$ $[M + H]^+$, 476.1680; found, 476.1688.

***N*-benzyl-*N*-(tert-butyl)-3-(((diphenylmethylene)amino)oxy)-3-oxopropanamide (1ae).**

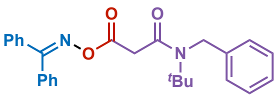

The product was obtained according to ***Method A*** as colorless oil (isolated yield: 90%). R_f 0.47 (hexane/EtOAc 2:1). ^{1}H NMR (500 MHz,

$CDCl_3$): δ 7.60 – 7.53 (m, 2H), 7.52 – 7.43 (m, 4H), 7.42 – 7.32 (m, 4H), 7.31 – 7.21 (m, 3H), 7.15 – 7.05 (m, 2H), 4.47 (s, 2H), 3.45 (s, 2H), 1.40 (s, 9H). ^{13}C NMR (126 MHz, $CDCl_3$) δ 166.8, 165.9, 164.9, 138.5, 134.8, 132.5, 130.9, 129.6, 129.2, 129.1, 128.9, 128.3, 128.2, 127.2, 125.4, 58.4, 49.1, 42.9, 28.5. HRMS (ESI-TOF, *m/z*): calcd for $C_{27}H_{28}N_2O_3$ $[M + Na]^+$, 451.1992; found, 451.1999.

***N*-(*tert*-butyl)-*N*-(3,5-dimethylbenzyl)acrylamide (2c).**

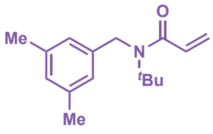

^1H NMR (500 MHz, $CDCl_3$): δ 6.90 (s, 1H), 6.83 (s, 2 H), 6.38 (dd, *J* = 16.6, 10.0 Hz, 1H), 6.30 (dd, *J* = 16.7, 2.4 Hz, 1H), 5.52 (dd, *J* = 10.0, 2.4 Hz, 1H), 4.56 (s, 2H), 2.30 (s, 6H), 1.46 (s, 9H); ^{13}C NMR (126 MHz, $CDCl_3$): δ 168.7, 139.6, 138.5, 131.9, 128.8, 127.1, 123.6, 57.8, 49.0, 28.7, 21.5; HRMS (ESI-TOF, *m/z*): calcd for $C_{16}H_{23}NO$ $[M + Na]^+$, 268,1672; found, 268.1676.

***N*-(*tert*-butyl)-*N*-(3,5-dichlorobenzyl)acrylamide (2d).**

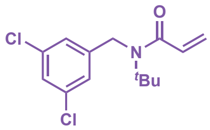

^1H NMR (500 MHz, $CDCl_3$): δ 7.27 (m, 1H), 7.12 (dd, *J* = 1.9, 0.9 Hz, 2H), 6.37–6.23 (m, 2H), 5.59 (dd, *J* = 9.5, 2.8 Hz, 1H), 4.57 (s, 2H), 1.44 (s, 9H); ^{13}C NMR (126 MHz, $CDCl_3$): δ 168.6, 143.5, 135.7, 131.0, 128.2, 127.6, 124.4, 58.0, 48.4, 28.7; HRMS (ESI-TOF, *m/z*): calcd for $C_{14}H_{17}ClNO$ $[M + H]^+$, 286,0754; found, 286.0750.

***N*-acryloyl-*N*-(*tert*-butyl)benzamide (2f)**

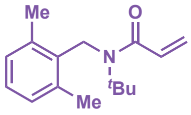

^1H NMR (400 MHz, CD_3OD): δ 7.96 (dd, *J* = 8.4, 1.4 Hz, 2H), 7.78–7.66 (m, 1H), 7.64–7.53 (m, 2H), 6.18 (dd, *J* = 16.4, 1.7 Hz, 1H), 5.96 (dd, *J* = 16.5, 10.3 Hz, 1H), 5.48 (dd, *J* = 10.3, 1.7 Hz, 1H), 1.53 (s, 9H); ^{13}C NMR (101 MHz, CD_3OD): δ 175.2, 165.3, 135.8, 134.5, 131.0, 130.3, 129.1, 127.2, 58.4, 27.3; HRMS (ESI-TOF, *m/z*): calcd for $C_{14}H_{17}NO_2$ $[M + Na]^+$, 254.1151; found, 254.1146.

***N*-acryloyl-*N*-isopropylbenzamide (2g)**

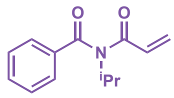

^1H NMR (400 MHz, $CDCl_3$): δ 7.63–7.33 (m, 5H), 6.08 (ddt, *J* = 16.8, 3.1, 1.7 Hz, 1H), 5.82 (ddt, *J* = 16.8, 10.3, 1.8 Hz, 1H), 5.32 (dtd, *J* = 10.5, 3.9, 3.2, 1.7 Hz, 1H), 4.78 (dddd, *J* = 13.7, 6.9, 5.5, 2.2 Hz, 1H), 1.39 (ddd, *J* = 7.0, 3.5, 1.6 Hz, 6H); ^{13}C NMR (101 MHz, $CDCl_3$): δ 174.0, 168.8, 137.1, 132.9, 132.2, 129.2, 128.9, 127.6, 49.8, 20.4; HRMS (ESI-TOF, *m/z*): calcd for $C_{13}H_{15}NO_2$ $[M + H]^+$, 218.1176; found, 218.1164.

N-(tert-butyl)-N-(thiophen-2-ylmethyl)acrylamide (2h)

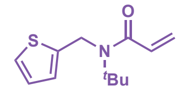

The product was obtained from 2-methyl-*N*-(thiophen-2-ylmethyl)propan-2-amine according to ***Method D*** as colorless solid (isolated yield: 69%). R_f 0.46 (hexane/EtOAc 4:1). ^{1}H NMR (500 MHz, $CDCl_3$): δ 7.22 (dd, *J* = 5.0, 1.2 Hz, 1H), 6.98 (dd, *J* = 5.0, 3.5 Hz, 1H), 6.91 (dd, *J* = 3.5, 1.2 Hz, 1H), 6.55 (dd, *J* = 16.6, 10.4 Hz, 1H), 6.31 (dd, *J* = 16.6, 2.0 Hz, 1H), 5.59 (dd, *J* = 10.4, 2.0 Hz, 1H), 4.75 (d, *J* = 1.2 Hz, 2H), 1.48 (s, 9H). ^{13}C NMR (126 MHz, $CDCl_3$) δ 168.3, 144.0, 131.5, 127.5, 127.2, 124.4, 124.1, 58.0, 45.0, 28.7. HRMS (ESI-TOF, *m/z*): calcd for $C_{12}H_{17}NOS$ $[M + Na]^+$, 246,0923; found, 246.0939.

N-(tert-butyl)-N-(dibenzo[b,d]furan-4-ylmethyl)acrylamide (2i)

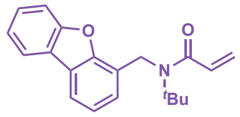

The product was obtained from 2-*N*-(dibenzo[b,d]furan-4-ylmethyl)-2-methylpropan-2-amine according to ***Method D*** as colorless solid (isolated yield: 69%). R_f 0.49 (hexane/EtOAc 4:1). ^{1}H NMR (500 MHz, $CDCl_3$): δ 7.98 (d, *J* = 7.7 Hz, 1H), 7.88 (dd, *J* = 7.1, 1.2 Hz, 1H), 7.60 (d, *J* = 8.2 Hz, 1H), 7.54 – 7.43 (m, 1H), 7.42 – 7.33 (m, 3H), 6.44 (dd, *J* = 16.6, 10.1 Hz, 1H), 6.34 (dd, *J* = 16.6, 2.3 Hz, 1H), 5.54 (dd, *J* = 10.1, 2.3 Hz, 1H), 5.01 (s, 2H), 1.51 (s, 9H). ^{13}C NMR (126 MHz, $CDCl_3$) δ 168.8, 156.3, 152.7, 131.6, 127.52, 127.49, 124.7, 124.3, 124.2, 123.8, 123.3, 123.1, 121.0, 119.5, 111.9, 58.0, 44.0, 28.6. HRMS (ESI-TOF, *m/z*): calcd for $C_{20}H_{21}NO_2$ $[M + Na]^+$, 330,1465; found, 330.1493.

N-([1,1'-biphenyl]-4-ylmethyl)-N-(tert-butyl)acrylamide (2j)

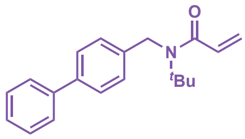

The product was obtained from *N*-([1,1'-biphenyl]-4-ylmethyl)-2-methylpropan-2-amine according to ***Method D*** as colorless solid (isolated yield: 69%). R_f 0.40 (hexane/EtOAc 4:1). ^{1}H NMR (500 MHz, $CDCl_3$): δ 7 7.72 – 7.55 (m, 4H), 7.45 (t, *J* = 7.7 Hz, 2H), 7.38 – 7.33 (m, 1H), 7.31 (d, *J* = 8.0 Hz, 2H), 6.42 (dd, *J* = 16.6, 10.1 Hz, 1H), 6.33 (dd, *J* = 16.6, 2.3 Hz, 1H), 5.56 (dd, *J* = 10.1, 2.3 Hz, 1H), 4.68 (s, 2H), 1.49 (s, 9H). ^{13}C NMR (126 MHz, $CDCl_3$) δ 168.7, 140.7, 140.3, 138.7, 131.7, 129.0, 127.6, 127.5, 127.4, 127.2, 126.3, 57.9, 48.9, 28.7. HRMS (ESI-TOF, *m/z*): calcd for $C_{20}H_{23}NO$ $[M + Na]^+$, 316,1672; found, 316.1700.

N-(tert-butyl)-N-(2-fluorobenzyl)acrylamide (2k)

The product was obtained from *N*-(2-fluorobenzyl)- 2-methylpropan-2-amine according to

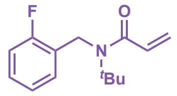

Method D as colorless solid (isolated yield: 69%). R_f 0.54 (hexane/EtOAc 4:1). ^{1}H

NMR (500 MHz, $CDCl_3$): δ 7.34 – 7.22 (m, 2H), 7.17 (td, *J* = 7.5, 1.2 Hz, 1H), 7.05 (ddd, *J* = 10.4, 8.1, 1.2 Hz, 1H), 6.34 – 6.29 (m, 2H), 5.56 (ddt, *J* = 6.2 Hz, 1H), 4.65 (s, 2H), 1.46 (s, 9H). ^{13}C NMR (126 MHz, $CDCl_3$) δ 168.7, 131.3, 128.8 (d, *J* = 8.0 Hz), 127.9 (d, *J* = 4.1 Hz), 127.7, 126.8 (d, *J* = 14.0 Hz), 124.6 (d, *J* = 3.6 Hz), 115.4 (d, *J* = 21.0 Hz), 57.9, 43.1 (d, *J* = 5.8 Hz), 28.5. ^{19}F NMR (377 MHz, $CDCl_3$): δ -118.9. HRMS (ESI-TOF, *m/z*): calcd for $C_{14}H_{18}FNO$ $[M + Na]^+$, 258.1265; found, 258.1286.

N-(*tert*-butyl)-*N*-(4-fluorobenzyl)acrylamide (2l)

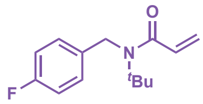

The product was obtained from *N*-(4-fluorobenzyl)-2-methylpropan-2-amine according to ***Method D*** as colorless solid (isolated yield: 70%). R_f 0.60 (hexane/EtOAc 4:1). ^1H NMR (500 MHz, $CDCl_3$): δ 7.20 (dd, *J* = 8.4, 5.3 Hz, 2H), 7.13 – 6.98 (m, 2H), 6.39 – 6.28 (m, 2H), 5.56 (dd, *J* = 9.1, 3.2 Hz, 1H), 4.60 (s, 2H), 1.44 (s, 9H). ^{13}C NMR (126 MHz, $CDCl_3$) δ 168.6, 162.1 (d, *J* = 245.6 Hz), 135.3 (d, *J* = 3.2 Hz), 131.5, 127.6, 127.4 (d, *J* = 8.0 Hz), 115.8 (d, *J* = 21.6 Hz), 57.9, 48.5, 28.7. ^{19}F NMR (377 MHz, $CDCl_3$) δ -115.8. HRMS (ESI-TOF, *m/z*): calcd for $C_{14}H_{18}FNO$ $[M + Na]^+$, 258.1265; found, 258.1278.

N-Benzyl-*N*-(*tert*-butyl)bicyclo[1.1.0]butane-1-carboxamide (2m)

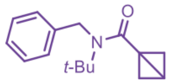

The product was obtained according to ***Method F*** as colorless solid. ^1H NMR (500 MHz, $CDCl_3$): δ 7.37 – 7.31 (m, 2H), 7.26 – 7.21 (m, 3H), 5.05 (s, 2H), 2.12 (d, J = 3.3 Hz, 2H), 1.97 (dq, J = 4.4, 1.7, 1.2 Hz, 1H), 1.40 (s, 9H), 0.89 (d, J = 2.3 Hz, 2H). ^{13}C NMR (101 MHz, $CDCl_3$) δ 173.0, 141.1, 128.7, 127.0, 126.3, 58.1, 50.5, 36.5, 28.8, 12.7, 10.9. HRMS (ESI-TOF, *m/z*): calcd for $C_{16}H_{21}NO$ $[M + H]^+$ 244.1696; found, 244.1698.

2-(*tert*-butyl)-8-((diphenylmethylene)amino)-4-(4-phenylbutyl)-2-azaspiro[4.5]deca-6,9-dien-3-one (3a)

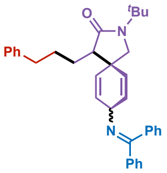

The product was obtained as a white solid (isolated yield: 60%, 1.4:1 dr). ^1H NMR (400 MHz, CD_3OD): δ 7.59–7.03 (m, 13.78H), 6.96–6.86 (m, 1.24H), 5.88–5.62 (m, 4H), 4.46 (m, 1H), 3.48–3.34 (m, 1.58H), 3.12 (d, *J* = 9.9 Hz, 0.36H), 2.70–2.56 (m, 0.67H), 2.53–2.28 (m, 2.35H), 2.05–1.09 (m, 13H); ^{13}C NMR (101 MHz, CD_3OD): δ 176.2, 175.7, 171.3, 169.8, 142.4, 142.0, 139.5, 139.4, 136.7, 136.4, 132.2, 131.6, 130.3, 130.0, 129.6, 128.8, 128.6, 128.5, 128.4, 128.3, 128.3, 128.1, 128.0, 127.9, 127.9, 127.8, 127.8, 127.7, 127.7, 127.4, 127.4, 127.4, 125.2, 56.5, 55.9, 55.5, 54.9, 53.9, 53.8, 52.5, 52.3, 47.0, 41.8, 41.7, 35.6, 35.5, 29.3, 29.0, 26.5, 26.5, 26.0, 25.8; HRMS (ESI-TOF, m/z): calcd for $C_{35}H_{38}N_2O$ $[M + H]^+$,

503.3056; found, 503.3061.

2-(*tert*-butyl)-8-((diphenylmethylene)amino)-4-(4,4,4-trifluorobutyl)-2-azaspiro[4.5]deca-6,9-dien-3-one (3b)

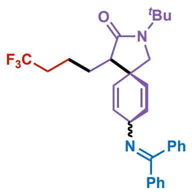

The product was obtained as a white solid (isolated yield: 54%, 1.6:1 dr). ^{1}H NMR (400 MHz, CD_3OD): δ 7.61–7.21 (m, 10H), 5.92–5.66 (m, 4H), 4.47 (dtt, *J* = 12.3, 3.4, 1.6 Hz, 1H), 3.56–3.34 (m, 1.72H), 3.15 (d, *J* = 10.0 Hz, 0.39H), 2.44–2.32 (m, 1H), 2.24–1.77 (m, 3H), 1.74–1.14 (m, 12H); ^{13}C NMR (126 MHz, CD_3OD): δ 177.2, 176.7, 172.9, 171.5, 140.9, 140.8, 138.0, 137.8, 133.2, 132.6, 131.7, 131.5, 130.1, 130.1, 129.9, 129.9, 129.9, 129.8, 129.7, 129.6, 129.5, 129.4, 129.4, 129.2, 129.1, 129.1, 128.7, 128.7, 57.8, 57.3, 56.7, 56.3, 55.3, 55.2, 53.6, 53.5, 43.3, 43.2, 34.7 (q, *J* = 28.3 Hz), 34.4 (q, *J* = 28.2 Hz)27.9, 27.9, 26.7, 26.5, 21.6 (q, *J* = 2.9 Hz), 21.2 (q, *J* = 3.1 Hz); ^{19}F NMR (377 MHz, CD_3OD): δ 67.6 (t, *J* = 11.2 Hz), –68.0 (t, *J* = 11.1 Hz); HRMS (ESI-TOF, *m/z*): calcd for $C_{30}H_{33}F_3N_2O$ $[M + H]^+$, 495.2618; found, 495.2612.

2-(*tert*-Butyl)-8-((diphenylmethylene)amino)-4-ethyl-2-azaspiro[4.5]deca-6,9-dien-3-one (3c)

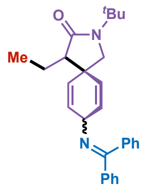

The product was obtained as a white solid (isolated yield: 46%, 1.3:1 dr). ^{1}H NMR (400 MHz, CD_3OD): δ 7.58–7.20 (m, 10H), 5.91–5.63 (m, 4H), 4.49–4.44 (m, 1H), 3.48–3.32 (m, 1.57H), 3.14 (d, *J* = 10.0 Hz, 0.42H), 2.28 (dd, *J* = 7.8, 6.0 Hz, 0.40H), 2.21 (dd, *J* = 8.3, 5.8 Hz, 0.60H), 1.75–1.48 (m, 2H), 1.41 (s, 5.40H), 1.36 (s, 3.60H), 1.10 (t, *J* = 7.5 Hz, 1.20H), 0.81 (t, *J* = 7.5 Hz, 1.86H); ^{13}C NMR (126 MHz, CD_3OD): δ 177.7, 177.3, 172.8, 171.6, 140.9, 140.8, 138.1, 137.9, 133.7, 133.2, 131.7, 131.5, 130.2, 130.0, 129.9, 129.8, 129.7, 129.6, 129.4, 129.3, 129.1, 129.1, 128.9, 128.8, 128.8, 128.7, 57.8, 57.3, 56.9, 56.4, 56.1, 55.9, 55.3, 55.2, 43.2, 43.1, 27.9, 27.9, 20.7, 20.7, 13.4, 13.0; HRMS (ESI-TOF, *m/z*): calcd for $C_{28}H_{32}N_2O$ $[M + H]^+$, 413.2587; found, 413.2584.

2-(*tert*-Butyl)-4-(cyclobutylmethyl)-8-((diphenylmethylene)amino)-2-azaspiro[4.5]deca-6,9-dien-3-one (3d)

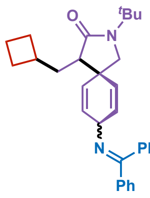

The product was obtained as a white solid (isolated yield: 56%, 1.4:1 dr). ^{1}H NMR (400 MHz, CD_3OD): δ 7.66–7.23 (m, 10H), 5.85–5.69 (m, 4H), 4.54–4.44 (m, 1H), 3.53–3.38 (m, 1H), 3.35 (d, *J* = 8.6 Hz, 0.58H), 3.14 (d, *J* = 9.9 Hz, 0.42H), 2.33–2.05 (m, 2H), 1.98–1.81 (m, 2H), 1.75–1.59 (m, 3H), 1.54–1.21 (m, 12H); ^{13}C NMR (101

MHz, CD_3OD): δ 176.4, 176.0, 171.4, 169.7, 139.6, 139.4, 136.6, 136.5, 132.0, 131.4, 130.3, 130.1, 128.8, 128.6, 128.6, 128.5, 128.5, 128.4, 128.3, 128.3, 127.9, 127.8, 127.7, 127.6, 127.4, 127.4, 56.5, 55.8, 55.4, 54.9, 53.9, 53.8, 50.5, 49.9, 41.9, 41.7, 33.3, 33.1, 33.1, 33.0, 28.0, 27.9, 27.2, 26.5, 26.5, 17.5, 17.3; HRMS (ESI-TOF, *m/z*): calcd for $C_{31}H_{36}N_2O$ $[M + H]^+$, 453.2900; found, 453.2899.

2-(*tert*-Butyl)-4-(cyclohexylmethyl)-8-((diphenylmethylene)amino)-2-azaspiro[4.5]deca-6,9-dien-3-one (3e, 8a)

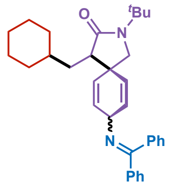

The product was obtained as a white solid (isolated yield: 60%, 1.5:1 dr). ^1H NMR (400 MHz, CD_3OD): δ 7.63–7.24 (m, 10H), 5.91–5.65 (m, 4H), 4.54–4.44 (m, 1H), 3.52–3.35 (m, 1.61H), 3.15 (d, J = 9.9 Hz, 0.40H), 2.56–2.47 (m, 1H), 1.89–1.24 (m, 18H), 1.18–0.62 (m, 4H); ^{13}C NMR (101 MHz, CD_3OD): δ 176.7, 176.3, 171.5, 169.5, 139.6, 139.2, 136.7, 136.4, 132.1, 131.2, 130.3, 130.0, 129.6, 128.7, 128.6, 128.5, 128.4, 128.3, 128.2, 128.2, 128.1, 127.9, 127.8, 127.7, 127.6, 127.5, 127.4, 127.3, 56.8, 55.9, 55.4, 55.0, 53.9, 53.7, 49.2, 42.0, 41.9, 34.0, 34.0, 33.8, 33.5, 33.4, 32.6, 31.9, 26.5, 26.5, 26.2, 26.1, 26.0, 25.9; HRMS (ESI-TOF, *m/z*): calcd for $C_{33}H_{40}N_2O$ $[M + H]^+$, 481.3213; found, 481.3211.

2-(*tert*-Butyl)-4-((4,4-difluorocyclohexyl)methyl)-8-((diphenylmethylene)amino)-2-azaspiro[4.5]deca-6,9-dien-3-one (3f)

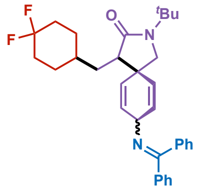

The product was obtained as a white solid (isolated yield: 61%, 1.3: 1 dr). ^1H NMR (400 MHz, CD_3CN): δ 7.61–7.22 (m, 10H), 5.83–5.59 (m, 4H), 4.41–4.34 (m, 1H), 3.46–3.03 (m, 2H), 2.44–2.31 (m, 1H), 2.084–1.98 (m, 1H), 1.91–1.64 (m, 4H), 1.53–1.12 (m, 13H), 1.05–0.91 (m, 2H); ^{13}C NMR (101 MHz, CD_3CN): δ 175.8, 175.4, 169.7, 169.3, 140.7, 140.5, 137.6, 137.5, 132.6, 132.5, 131.2, 131.2, 129.8, 129.8, 129.7, 129.7, 129.6, 129.5, 129.4, 129.2, 129.2, 129.1, 129.1, 129.1, 128.5, 128.5, 128.3, 57.6, 57.0, 56.0, 56.0, 54.3, 54.2, 50.2, 49.8, 42.9, 42.8, 34.14 (m), 33.98 (m), 33.4, 33.4, 33.1, 33.1, 33.1, 33.1, 30.3 (dd, J = 9.7, 4.2 Hz), 29.0 (dd, J = 35.1, 9.7 Hz), 27.8, 27.8. ^{19}F NMR (377 MHz, CD_3CN) δ -91.2 (d, J = 233.6 Hz), -101.9 (t, J = 226.4 Hz); HRMS (ESI-TOF, *m/z*): calcd for $C_{33}H_{38}F_2N_2O$ $[M + H]^+$, 517.3025; found, 517.3025.

2-(*tert*-Butyl)-8-((diphenylmethylene)amino)-4-neopentyl-2-azaspiro[4.5]deca-6,9-dien-3-one (3g)

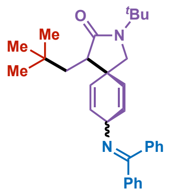

The product was obtained as a white solid (isolated yield: 50%, 1.5:1 dr). ^{1}H NMR (500 MHz, CD_3OD): δ 7.60–7.22 (m, 10H), 5.82–5.67 (m, 4H), 4.48–4.40 (m, 1H), 3.53–3.40 (m, 1.61H), 3.16 (d, *J* = 9.8 Hz, 0.39H), 1.44–1.33 (m, 1H), 1.55–1.35 (m, 11H), 0.94 (s, 3.55H), 0.76 (s, 5.41H); ^{13}C NMR (126 MHz, CD_3OD): δ 178.1, 177.7, 172.7, 171.1, 141.0, 140.8, 138.1, 137.8, 133.0, 132.6, 131.7, 131.4, 130.4, 130.2, 130.1, 130.1, 130.0, 129.9, 129.9, 129.9, 129.8, 129.7, 129.5, 129.4, 129.2, 129.1, 128.7, 128.7, 57.9, 57.3, 56.4, 55.9, 55.2, 55.1, 51.0, 50.5, 45.0, 45.0, 39.4, 38.8, 31.1, 30.8, 30.2, 30.1, 28.0, 27.9; HRMS (ESI-TOF, *m/z*): calcd for $C_{31}H_{38}N_2O$ $[M + H]^+$, 455.3057; found, 455.3052.

4-(Adamantan-1-ylmethyl)-2-(*tert*-butyl)-8-((diphenylmethylene)amino)-2-azaspiro[4.5]deca-6,9-dien-3-one (3h)

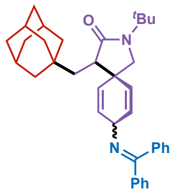

The product was obtained as a white solid (isolated yield: 44%, 1.4:1 dr). ^{1}H NMR (400 MHz, CD_3OD): δ 7.65–7.25 (m, 10H), 5.86–5.67 (m, 4H), 4.50–4.41 (m, 1H), 3.58–3.41 (m, 1.6H), 3.19 (d, *J* = 10.0 Hz, 0.42H), 2.52–2.41 (m, 1H), 1.99–1.13 (m, 25H), 0.93 (dd, *J* = 12.6, 7.1 Hz, 0.43H), 0.72 (dd, *J* = 14.4, 3.0 Hz, 0.60H); ^{13}C NMR (101 MHz, CD_3OD): δ 176.9, 176.5, 171.4, 169.4, 139.6, 139.3, 136.7, 136.5, 131.6, 131.3, 130.3, 130.0, 129.6, 128.9, 128.8, 128.8, 128.6, 128.6, 128.5, 128.4, 128.4, 128.3, 128.1, 128.1, 128.1, 127.9, 127.6, 127.3, 56.6, 55.9, 55.0, 54.5, 53.8, 53.7, 43.6, 43.5, 42.3, 42.2, 38.8, 38.2, 36.9, 36.7, 31.7, 31.3, 28.8, 28.7, 26.6, 26.6; HRMS (ESI-TOF, *m/z*): calcd for $C_{37}H_{44}N_2O$ $[M + H]^+$, 533.3526; found, 533.3526.

7-(*tert*-butyl)-2-((diphenylmethylene)amino)-9-(3-phenylpropyl)-1-thia-7-azaspiro[4.4]non-3-en-8-one (3i)

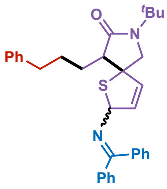

The target compounds (**A:B:C**) were obtained with total yield 52% and in a diastereomeric ratio of 4.5:2.3:1 and was isolated a major **isomer A** (30%) and a **mixture of two isomers B and C** (22%, ratio 1:2.2). The mixture was purified again to obtain enriched fraction **B:C** 1:3.2, as yellowish foam R_f 0.45 (hexane/EtOAc 4:1).

Major isomer (isolated yield: 30%). The product was obtained as yellowish foam (isolated yield: 30%). R_f 0.32 (hexane/EtOAc 4:1). ^{1}H NMR (500 MHz, CD_3OD): δ 7.62 – 7.50 (m, 5H), 7.48 – 7.43 (m, 1H), 7.39 – 7.34 (m, 2H), 7.23 – 7.19 (m, 2H), 7.16 – 7.11 (m, 3H), 7.04 – 6.98 (m, 2H), 5.98 (dd, *J* = 6.1, 1.4 Hz,

1H), 5.80 (dd, J = 6.1, 2.6 Hz, 1H), 5.72 (dd, J = 2.6, 1.4 Hz, 1H), 3.97 (d, J = 10.5 Hz, 1H), 3.86 (d, J = 10.5 Hz, 1H), 2.59 (dd, J = 7.4, 5.3 Hz, 1H), 2.58 – 2.50 (m, 1H), 2.51 – 2.41 (m, 1H), 1.62 – 1.48 (m, 4H), 1.39 (s, 9H). ^{13}C NMR (126 MHz, CD_3OD) δ 176.9, 172.9, 143.4, 140.5, 137.4, 137.2, 133.3, 132.0, 130.5, 130.1, 129.9, 129.33, 129.26, 129.19, 129.12, 126.6, 75.2, 71.7, 59.9, 55.5, 53.3, 36.9, 30.7, 30.5, 28.0. HRMS (ESI-TOF, *m/z*): calcd for $C_{33}H_{36}N_2OS$ $[M + H]^+$, 509.2621; found, 509.2627.

Mixture of two minor isomers B and C (isolated yield: 22%, ratio 1:2.2). The mixture was purified again to obtain enriched fraction 1:3.2, obtained as yellowish foam R_f 0.45 (hexane/EtOAc 4:1). ^1H NMR (500 MHz, CD_3OD): δ 7.59 – 7.50 (m, 5H), 7.43 – 7.35 (m, 2H), 7.32 – 7.26 (m, 2H), 7.22 – 7.16 (m, 6H), 5.95 (dd, J = 6.1, 1.3 Hz, 1H), 5.86 (dd, J = 6.1, 2.6 Hz, 1H), 5.83 (dd, J = 2.6, 1.3 Hz, 1H), 3.73 (d, J = 10.3 Hz, 1H), 3.58 (d, J = 10.3 Hz, 1H), 2.73 – 2.60 (m, 3H), 2.08 – 1.99 (m, 1H), 1.97 – 1.89 (m, 1H), 1.88 – 1.71 (m, 1H), 1.64 – 1.53 (m, 1H), 1.35 (s, 9H).^{13}C NMR (126 MHz, CD_3OD) δ 177.2, 171.5, 143.7, 140.6, 137.5, 136.7, 133.6, 131.7, 130.1, 129.92, 129.86, 129.5, 129.2, 129.11, 129.05, 126.6, 74.6, 71.3, 59.8, 55.5, 54.1, 37.2, 30.4, 28.9, 28.0. HRMS (ESI-TOF, *m/z*): calcd for $C_{33}H_{36}N_2OS$ $[M + H]^+$, 509.2621; found, 509.2634.

1'-(tert-butyl)-1-((diphenylmethylene)amino)-4'-(3-phenylpropyl)-1H-spiro[dibenzo[b,d]furan-4,3'-pyrrolidin]-5'-one (3j)

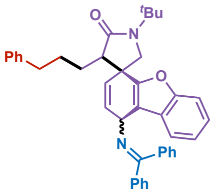

The target compound was obtained as mixture of isomers (A:B:C:D) with a total yield of 71% and a diastereomeric ratio of 4.6:2.8:2.8:1.

Major Isomer A (isolated yield: 22%). The product was obtained as yellowish foam. R_f 0.48 (hexane/EtOAc 4:1). ^1H NMR (500 MHz, CD_3OD): δ 7.65 (dd, J = 8.1, 6.8 Hz, 2H), 7.62 – 7.56 (m, 1H), 7.56 – 7.47 (m, 5H), 7.45 – 7.40 (m, 1H), 7.39 – 7.28 (m, 4H), 7.27 – 7.21 (m, 1H), 6.96 – 6.84 (m, 3H), 6.60 (dd, J = 6.8, 1.6 Hz, 2H), 5.94 (dd, J = 10.0, 1.5 Hz, 1H), 5.89 (dd, J = 10.0, 3.1 Hz, 1H), 5.28 (dd, J = 3.1, 1.5 Hz, 1H), 4.03 (d, J = 10.1 Hz, 1H), 3.59 (d, J = 10.1 Hz, 1H), 3.05 (dd, J = 8.6, 4.6 Hz, 1H), 2.31 – 2.16 (m, 2H), 1.73 – 1.64 (m, 1H), 1.45 (s, 9H), 1.26 – 1.14 (m, 3H). ^{13}C NMR (126 MHz, CD_3OD) δ 176.0, 173.5, 156.0, 153.7, 142.9, 140.8, 137.7, 131.9, 130.6, 130.2, 129.9, 129.7, 129.3, 129.05, 129.04, 128.98, 128.4, 126.5, 125.6, 124.3, 120.4, 116.5, 112.4, 57.5, 55.6, 54.5, 51.1, 43.4, 36.0, 29.7, 27.9, 26.5. HRMS (ESI-TOF, *m/z*): calcd for $C_{41}H_{41}N_2O_2$ $[M + H]^+$, 594.3246; found, 594.3234.

Minor isomer B (isolated yield: 17%). R_f 0.55 (hexane/EtOAc 4:1). ^1H NMR (500 MHz, CD_3OD): δ 7.64 – 7.59 (m, 2H), 7.58 – 7.50 (m, 3H), 7.49 – 7.45 (m, 2H), 7.44 – 7.40 (m, 2H), 7.38 – 7.26 (m, 3H), 7.26 –

7.20 (m, 1H), 7.19 – 7.13 (m, 2H), 7.05 – 6.94 (m, 2H), 6.82 – 6.70 (m, 2H), 5.96 (dd, *J* = 10.0, 1.6 Hz, 1H), 5.89 (dd, *J* = 10.0, 3.6 Hz, 1H), 5.28 (dd, *J* = 3.6, 1.6 Hz, 1H), 3.95 (d, *J* = 10.0 Hz, 1H), 3.34 (d, *J* = 10.0 Hz, 1H), 3.17 (dd, *J* = 9.6, 4.9 Hz, 1H), 2.39 (td, *J* = 7.9, 3.7 Hz, 2H), 1.84 – 1.76 (m, 1H), 1.73 – 1.64 (m, 2H), 1.63 – 1.54 (m, 1H), 1.42 (s, 9H). ^{13}C NMR (126 MHz, CD_3OD) δ 176.4, 171.4, 156.0, 153.4, 143.4, 140.8, 138.0, 133.8, 131.4, 131.0, 130.2, 130.1, 129.9, 129.5, 129.4, 129.05, 129.00, 128.7, 126.3, 125.4, 124.1, 120.6, 117.2, 112.3, 57.2, 55.6, 54.8, 50.8, 43.3, 36.2, 29.4, 27.9, 27.1. HRMS (ESI-TOF, *m/z*): calcd for $C_{41}H_{41}N_2O_2$ [M + H]$^+$, 594.3246; found, 594.3230.

Minor isomer C (isolated yield: 17%). R_f 0.34 (hexane/EtOAc 4:1). ^{1}H NMR (500 MHz, CD_3OD): δ 7.71 – 7.59 (m, 3H), 7.55 – 7.46 (m, 4H), 7.46 – 7.41 (m, 1H), 7.38 – 7.29 (m, 4H), 7.29 – 7.16 (m, 2H), 6.82 – 6.75 (m, 3H), 6.66 (dd, *J* = 6.5, 3.0 Hz, 2H), 6.05 (dd, *J* = 9.8, 1.7 Hz, 1H), 5.91 (dd, *J* = 9.8, 3.4 Hz, 1H), 5.26 (dd, *J* = 3.4, 1.7 Hz, 1H), 3.78 (d, *J* = 10.4 Hz, 1H), 3.70 (d, *J* = 10.4 Hz, 1H), 2.67 (dd, *J* = 9.5, 4.5 Hz, 1H), 2.40 (ddd, *J* = 13.4, 7.4, 6.0 Hz, 1H), 2.14 (dt, *J* = 13.5, 7.7 Hz, 1H), 1.64 – 1.54 (m, 2H), 1.49 (s, 9H), 1.42 – 1.33 (m, 2H). ^{13}C NMR (126 MHz, CD_3OD) δ 176.6, 173.4, 155.8, 155.0, 142.9, 140.8, 137.8, 133.9, 131.9, 130.7, 130.2, 129.9, 129.32, 129.29, 129.1, 128.98, 128.9, 127.7, 126.4, 125.3, 124.0, 120.6, 115.7, 112.2, 57.6, 55.8, 55.0, 53.4, 44.7, 36.6, 30.4, 28.0, 27.1. HRMS (ESI-TOF, *m/z*): calcd for $C_{41}H_{41}N_2O_2$ [M + H]$^+$, 594.3246; found, 594.3276.

Mixture of isomers A and D (isolated yield: 15%, the ratio 1:1.5). R_f 0.48, 0.41 (hexane/EtOAc 4:1). ^{1}H NMR (500 MHz, CD_3OD): δ 7.71 – 7.20 (m, 36H), 7.05 – 6.98 (m, 3H), 6.93 – 6.82 (m, 7H), 6.64 – 6.55 (m, 2H), 6.01 (dd, *J* = 9.8, 1.6 Hz, 1H), 5.94 (dd, *J* = 10.0, 1.4 Hz, 1.5H), 5.90 (t, *J* = 3.3 Hz, 1.5H), 5.88 (t, *J* = 3.2 Hz, 1H), 5.30 (dd, *J* = 3.6, 1.6 Hz, 1H), 5.28 (dd, *J* = 3.2, 1.5 Hz, 1.5H), 4.03 (d, *J* = 10.1 Hz, 1.5H), 3.63 – 3.56 (m, 2.5H), 3.53 (d, *J* = 10.3 Hz, 1H), 3.05 (dd, *J* = 8.5, 4.7 Hz, 1.5H), 2.76 (dd, *J* = 9.1, 4.8 Hz, 1H), 2.60 (ddd, *J* = 14.1, 9.1, 5.2 Hz, 1H), 2.45 (dt, *J* = 14.8, 7.9 Hz, 1.5H), 2.30 – 2.16 (m, 3H), 1.76 – 1.63 (m, 4H), 1.45 (s, 13.5H), 1.45 (s, 9H). ^{13}C NMR (126 MHz, CD_3OD) δ 177.0, 176.0, 173.5, 171.6, 156.0, 155.9, 155.2, 153.7, 143.3, 142.9, 140.9, 140.8, 138.1, 137.7, 133.2, 131.9, 131.4, 131.0, 130.6, 130.24, 130.17, 130.1, 129.94, 129.87, 129.78, 129.7, 129.3, 129.1, 129.05, 129.01, 128.98 128.92, 128.4, 128.2, 120.5, 120.4, 116.5, 116.3, 112.4, 112.2, 88.6, 82.0, 57.5, 57.3, 55.7, 55.6, 55.45, 54.35, 53.3, 51.1, 49.0, 44.6, 43.5, 37.6, 36.8, 36.0, 29.9, 29.7, 28.0, 27.9, 27.6, 26.5. HRMS (ESI-TOF, *m/z*): calcd for $C_{41}H_{41}N_2O_2$ [M + H]$^+$, 594.3246; found, 594.3233.

2-(tert-butyl)-8-((diphenylmethylene)amino)-6-fluoro-4-(3-phenylpropyl)-2-azaspiro[4.5]deca-6,9-dien-3-one (3k)

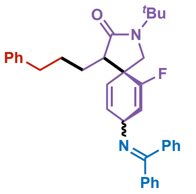

The target compounds were obtained as mixture of isomers (**A:B:C:D**), with total yield of 73% and a diastereomeric ratio of 3.3:2.5:2.0:1.

Major isomer A (isolated yield: 22%). R_f 0.41 (hexane/EtOAc 4:1). 7.64 – 7.50 (m, 5H), 7.46 – 7.40 (m, 1H), 7.36 (dd, *J* = 8.4, 6.9 Hz, 2H), 7.28 (dd, *J* = 7.8, 1.7 Hz, 2H), 7.11 – 7.04 (m, 3H), 6.90 (dd, *J* = 7.7, 1.8 Hz, 2H), 5.81 (td, *J* = 9.4, 1.5 Hz, 1H), 5.61 (dp, *J* = 9.8, 2.0 Hz, 1H), 5.19 (ddd, *J* = 18.4, 3.7, 1.6 Hz, 1H), 4.65 (dtd, *J* = 5.0, 3.5, 1.5 Hz, 1H), 3.73 (d, *J* = 10.7 Hz, 1H), 3.56 (d, *J* = 10.7 Hz, 1H), 2.52 (ddd, *J* = 14.1, 8.5, 5.9 Hz, 1H), 2.46 (dd, *J* = 9.2, 4.9 Hz, 1H), 2.37 (dt, *J* = 13.5, 7.8 Hz, 1H), 1.75 (ddd, *J* = 17.7, 10.8, 5.3 Hz, 1H), 1.62 – 1.43 (m, 2H), 1.40 (s, 9H), 1.28 – 1.21 (m, 1H). ^{13}C NMR (126 MHz, CD_3OD) δ 176.4, 172.9, 162.7 (d, *J* = 262.1 Hz), 143.3, 140.6, 137.7, 133.8, 133.0 (d, *J* = 7.9 Hz), 131.8, 131.0, 130.3, 130.1, 129.8, 129.5, 129.27, 129.25, 129.19, 128.7, 128.3 (d, *J* = 2.8 Hz), 126.6, 106.0 (d, *J* = 15.9 Hz), 59.2 (d, *J* = 10.0 Hz), 55.5, 54.4, 52.0, 45.0 (d, *J* = 20.6 Hz), 36.8, 30.8, 27.8, 27.2. ^{19}F NMR (377 MHz, CD_3OD) δ -117.0. HRMS (ESI-TOF, *m/z*): calcd for $C_{35}H_{37}FN_2O$ $[M + H]^+$, 521.2963; found, 521.2979.

Minor isomer C (isolated yield: 13.5%). R_f 0.54 (hexane/EtOAc 4:1). ^{1}H NMR (500 MHz, CD_3OD): δ 7.80 – 7.75 (m, 1H), 7.58 – 7.45 (m, 5H), 7.40 – 7.33 (m, 1H), 7.33 – 7.14 (m, 7H), 7.14 – 7.05 (m, 2H), 5.73 (ddd, *J* = 10.0, 8.6, 1.3 Hz, 1H), 5.66 (ddt, *J* = 10.0, 3.7, 1.8 Hz, 1H), 5.36 (ddd, *J* = 17.1, 3.9, 1.8 Hz, 1H), 4.69 (dd, *J* = 3.9, 1.3 Hz, 1H), 3.68 (d, *J* = 9.9 Hz, 1H), 3.16 (d, *J* = 9.9 Hz, 1H), 2.81 (dd, *J* = 9.4, 5.0 Hz, 1H), 2.72 – 2.58 (m, 2H), 2.11 – 1.99 (m, 1H), 1.86 (dddd, *J* = 13.0, 9.3, 6.5, 3.4 Hz, 1H), 1.78 (ddt, *J* = 13.9, 10.7, 5.5 Hz, 1H), 1.60 (dtd, *J* = 14.6, 9.6, 5.1 Hz, 1H), 1.37 (s, 9H). ^{13}C NMR (126 MHz, CD_3OD) δ 176.3, 171.2, 160.2 (d, *J* = 257.1 Hz), 143.6, 140.7, 137.9, 133.8, 131.5, 131.0, 130.0, 129.9, 129.7, 129.5, 129.5, 129.2, 129.1, 128.6, 128.1 (d, *J* = 5.1 Hz), 126.6, 107.6 (d, *J* = 16.9 Hz), 58.6 (d, *J* = 10.1 Hz), 55.5, 53.3, 44.4 (d, *J* = 22.4 Hz), 36.8, 29.8, 27.8, 27.3. ^{19}F NMR (377 MHz, CD_3OD) δ -120.2. HRMS (ESI-TOF, *m/z*): calcd for $C_{35}H_{37}FN_2O$ $[M + H]^+$, 521.2963; found, 521.2980.

Mixture of isomers B and D (isolated yield: 30.7%, ratio 1:2). R_f 0.48 (hexane/EtOAc 4:1). ^{1}H NMR (500 MHz, CD_3OD): δ 7.84 – 7.74 (m, 2H), 7.70 – 7.64 (m, 1H), 7.63 – 7.07 (m, 38H), 6.97 – 6.90 (m, 4H), 5.84 – 5.77 (m, 1H), 5.71 (ddd, *J* = 10.1, 8.6, 1.5 Hz, 2H), 5.67 – 5.63 (m, 1H), 5.62 – 5.56 (m, 2H), 5.38 (ddd, *J* = 16.8, 3.9, 1.7 Hz, 2H), 5.28 (ddd, *J* = 18.5, 4.1, 1.6 Hz, 1H), 4.68 (dq, *J* = 4.5, 3.1, 2.4 Hz, 3H), 3.77 (d, *J* = 10.0 Hz, 2H), 3.53 (d, *J* = 10.5 Hz, 1H), 3.47 (d, *J* = 11.0 Hz, 1H), 3.44 (d, *J* = 10.4 Hz, 2H), 2.75 (dd, *J*

= 9.6, 4.7 Hz, 2H), 2.70 (ddd, *J* = 11.7, 9.3, 6.3 Hz, 2H), 2.61 (dd, *J* = 8.8, 5.1 Hz, 1H), 2.53 (ddd, *J* = 14.0, 8.3, 6.0 Hz, 2H), 2.39 (dt, *J* = 13.6, 7.8 Hz, 2H), 2.15 – 2.05 (m, 1H), 1.95 – 1.82 (m, 1H), 1.69 (ddt, *J* = 13.7, 10.7, 5.4 Hz, 3H), 1.59 – 1.48 (m, 7H), 1.43 (s, 18H), 1.39 (s, 9H). ^{13}C NMR (126 MHz, CD_3OD) δ 176.7, 175.9, 172.1 (d, *J* = 217.2 Hz), 160.6 (d, *J* = 258.2 Hz), 143.7, 143.2, 140.7 (d, *J* = 14.3 Hz), 137.7, 133.8, 132.2 (d, *J* = 7.7 Hz), 131.8, 131.4, 131.0, 130.3, 130.1, 130.0, 129.8, 129.7, 129.5, 129.5, 129.3, 129.2, 129.1, 128.7, 128.6, 128.4 (d, *J* = 5.2 Hz), 128.1 (d, *J* = 2.5 Hz), 126.6, 126.6, 107.1 (d, *J* = 17.4 Hz), 106.3 (d, *J* = 15.3 Hz), 59.0 (d, *J* = 10.0 Hz), 58.7 (d, *J* = 10.1 Hz), 55.6, 54.9, 53.1, 51.7, 45.1, 44.4 (d, *J* = 22.3 Hz), 37.1, 36.6, 30.4, 30.3, 27.8, 26.9, 24.4. ^{19}F NMR (377 MHz, CD_3OD) δ -117.5, -118.6. HRMS (ESI-TOF, *m/z*): calcd for $C_{35}H_{37}FN_2O$ $[M + H]^+$, 521.2963; found, 521.2999.

2-(*tert*-butyl)-8-((diphenylmethylene)amino)-6-fluoro-4-(3-phenylpropyl)-2-azaspiro[4.5]deca-6,9-dien-3-one (3l).

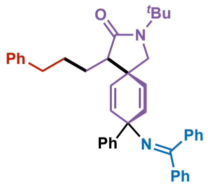

The target compound was obtained as mixture of isomers with a total yield of 33% and diastereomeric ratio of 1:1.25. R_f 0.47 (hexane/EtOAc 4:1). ^{1}H NMR (500 MHz, CD_3OD): δ 7.67 – 7.03 (m, 32H), 6.98 – 6.92 (m, 4H), 6.41 (dd, *J* = 9.7, 1.6 Hz, 1H), 5.98 – 5.90 (m, 3H), 5.70 (dd, *J* = 4.9, 1.5 Hz, 1H), 5.52 – 5.43 (m, 1H), 5.40 (dd, *J* = 10.0, 2.2 Hz, 1H), 4.33 (d, *J* = 4.9 Hz, 1H), 4.05 (d, *J* = 10.0 Hz, 1H), 3.83 (dt, *J* = 9.0, 4.7 Hz, 2H), 3.31 (d, *J* = 10.0 Hz, 4H), 2.92 (d, *J* = 10.1 Hz, 1H), 2.69 (d, *J* = 10.1 Hz, 1H), 2.45 – 2.32 (m, 4H), 2.27 (dd, *J* = 8.1, 4.3 Hz, 1H), 2.17 (t, *J* = 6.3 Hz, 1H), 1.58 – 1.49 (m, 4H), 1.45 (s, 9H), 1.40 (s, 9H), 1.29 – 1.19 (m, 2H). ^{13}C NMR (126 MHz, CD_3OD) δ 177.9, 177.3, 171.5, 170.4, 149.5, 143.4, 143.2, 142.8, 141.3, 140.3, 140.0, 138.8, 138.2, 137.9, 137.5, 136.0, 134.1, 133.8, 133.5, 132.9, 131.8, 131.4, 131.2, 131.0, 130.23, 130.05, 129.96, 129.83, 129.81, 129.63, 129.52, 129.50, 129.40, 129.37, 129.33, 129.29, 129.23, 129.18, 129.15, 129.05, 129.04, 128.95, 128.78, 128.53, 128.4, 127.9, 127.6, 127.4, 127.2, 126.7, 126.6, 126.5, 123.9, 83.7, 65.4, 61.2, 55.9, 55.6, 55.4, 55.1, 54.8, 50.5, 44.9, 43.1, 37.0, 36.8, 31.4, 28.1, 28.0, 26.9, 24.7. HRMS (ESI-TOF, *m/z*): calcd for $C_{41}H_{42}N_2O$ $[M + Na]^+$, 601,3189; found, 601.3206.

2-(*tert*-butyl)-8-((diphenylmethylene)amino)-4-(2-oxo-2-phenylethyl)-2-azaspiro[4.5]deca-6,9-dien-3-one (3m, 8e)

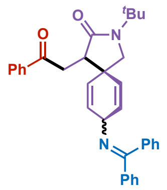

The product was obtained as a white solid (isolated yield: 57%, 1.3:1 dr). ^{1}H NMR (400 MHz, CD_3OD): δ 8.10–8.02 (m, 0.78H), 7.81–7.72 (m, 1.22H), 7.57–7.09 (m, 13H), 5.89–5.51 (m, 4H), 4.42 (d, *J* = 3.8 Hz, 0.41H), 4.16 (qd, *J* = 3.2, 2.1, 1.6 Hz, 0.61H), 3.60–3.43 (m, 2.62H), 3.31–3.25 (m, 1.28H), 3.04 (dd, *J* = 17.0, 5.0 Hz, 0.62H), 2.92–2.83 (m, 0.63H), 1.43–1.40 (m, 9H); ^{13}C NMR (101 MHz, CD_3OD): δ 199.0, 198.4, 175.2, 174.6, 171.3, 170.1, 139.3, 139.3, 137.1, 136.9, 136.6, 136.3, 132.9, 132.8, 131.0, 130.8, 130.3, 130.0, 129.6, 129.0, 128.7, 128.6, 128.6, 128.5, 128.5, 128.4, 128.4, 128.3, 128.2, 128.2, 128.1, 128.0, 127.9, 127.8, 127.7, 127.7, 127.3, 127.2, 56.1, 55.9, 55.3, 54.9, 54.1, 54.0, 52.1, 49.9, 42.3, 41.9, 34.4, 33.9, 26.5; HRMS (ESI-TOF, *m/z*): calcd for $C_{34}H_{34}N_2O_2$ [M + H]$^+$, 503.2700; found, 503.2698.

2-(*tert*-Butyl)-8-((diphenylmethylene)amino)-4-(2-mesityl-2-oxoethyl)-2-azaspiro[4.5]deca-6,9-dien-3-one (3n)

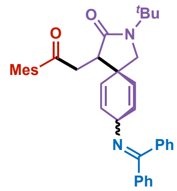

The product was obtained as a white solid (isolated yield: 63%, 1.3:1 dr). ^{1}H NMR (400 MHz, CD_3OD): δ 7.58–7.20 (m, 10H), 6.87 (s, 0.76H), 6.77 (s, 1.25H), 5.97–5.67 (m, 4H), 4.45–4.39 (m, 1H), 3.67–3.48 (m, 1.64H), 3.41–3.36 (m, 0.42H), 3.33–3.26 (m, 0.82H), 3.12–2.94 (m, 1.60H), 2.32–2.24 (m, 6H), 1.92 (s, 3H), 1.45–1.40 (m, 9H); ^{13}C NMR (101 MHz, CD_3OD): δ 209.1, 208.1, 174.8, 174.4, 171.6, 169.5, 139.3, 139.2, 138.7, 138.2, 138.1, 136.7, 136.5, 132.6, 132.1, 131.3, 130.5, 130.4, 129.9, 129.6, 129.5, 128.8, 128.7, 128.7, 128.6, 128.5, 128.4, 128.2, 128.2, 128.1, 128.1, 128.0, 127.9, 127.9, 127.7, 127.3, 56.4, 56.0, 55.2, 54.9, 54.1, 53.9, 42.3, 42.3, 40.5, 40.5, 26.6, 26.5, 19.8, 19.7, 18.3, 17.7; HRMS (ESI-TOF, *m/z*): calcd for $C_{37}H_{40}N_2O_2$ [M + H]$^+$, 567.2982; found, 567.2969.

Methyl 2-(2-(*tert*-butyl)-8-((diphenylmethylene)amino)-3-oxo-2-azaspiro[4.5]deca-6,9-dien-4-yl)acetate (3o)

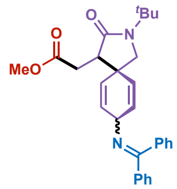

The product was obtained as a white solid (isolated yield: 61%, 1.4:1 dr). ^{1}H NMR (400 MHz, CD_3OD): δ 7.56–7.22 (m, 10H), 5.83–5.67 (m, 4H), 4.43 (dq, *J* = 4.7, 1.9, 1.3 Hz, 1H), 3.70 (s, 1.20H), 3.55 (d, *J* = 9.9 Hz, 0.62H), 3.50–3.41 (m, 1H), 3.37 (s, 1.79H), 3.21 (d, *J* = 10.1 Hz, 0.42H), 3.07 (dd, *J* = 8.5, 4.6 Hz, 0.40H), 2.95 (dd, *J* = 8.2, 5.9 Hz, 0.62H), 2.67 (dd, *J* = 17.0, 4.6 Hz, 0.42H), 2.51–2.39 (m, 1H), 2.04 (dd, *J* = 15.8,

8.2 Hz, 0.62H), 1.43–1.35 (m, 9H); ^{13}C NMR (101 MHz, CD_3OD): δ 174.5, 173.7, 173.4, 172.9, 171.4, 170.4, 139.4, 139.3, 136.6, 136.5, 130.6, 130.5, 130.4, 130.2, 129.6, 129.3, 128.8, 128.7, 128.6, 128.6, 128.5, 128.5, 128.4, 128.3, 128.1, 127.9, 127.8, 127.4, 127.3, 127.2, 56.2, 55.9, 55.2, 54.8, 54.1, 54.0, 51.0, 50.7, 50.4, 49.3, 41.9, 41.7, 30.0, 29.8, 26.5; HRMS (ESI-TOF, *m/z*): calcd for $C_{29}H_{32}N_2O_3$ [M + H]$^+$, 457.2476; found, 457.2476.

Ethyl 2-(2-(*tert*-butyl)-8-((diphenylmethylene)amino)-3-oxo-2-azaspiro[4.5]deca-6,9-dien-4-yl)acetate (3p)

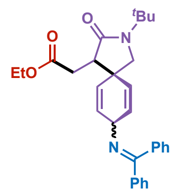

The product was obtained as a white solid (isolated yield: 62%, 1.3:1 dr). ^{1}H NMR (500 MHz, CD_3CN): δ 7.57–7.46 (m, 5H), 7.44–7.39 (m, 1H), 7.38–7.32 (m, 2H), 7.27–7.22 (m, 2H), 5.80–5.61 (m, 4H), 4.37–4.31 (m, 1H), 4.13 (q, *J* = 7.1 Hz, 0.80H), 3.87 (dq, *J* = 10.7, 7.1 Hz, 0.62H), 3.76 (dq, *J* = 10.8, 7.1 Hz, 0.63H), 3.49 (d, *J* = 9.9 Hz, 0.62H), 3.41 (d, *J* = 9.8 Hz, 0.39H), 3.25 (d, *J* = 9.9 Hz, 0.61H), 3.14 (d, *J* = 9.8 Hz, 0.40H), 2.96 (dd, *J* = 8.3, 4.9 Hz, 0.39H), 2.83 (dd, *J* = 8.0, 6.0 Hz, 0.61H), 2.48 (dd, *J* = 16.9, 4.9 Hz, 0.39H), 2.41–2.32 (m, 1H), 1.97–1.90 (m, 0.59H), 1.36–1.33 (m, 9H), 1.24 (t, *J* = 7.1 Hz, 1.71H), 0.99 (t, *J* = 7.1 Hz, 1.81H); ^{13}C NMR (126 MHz, CD_3CN): δ 173.0, 172.3, 172.0, 171.8, 168.3, 168.1, 139.4, 139.3, 136.3, 136.3, 130.6, 129.9, 129.9, 129.1, 129.0, 128.8, 128.4, 128.4, 128.3, 128.2, 128.2, 127.9, 127.9, 127.8, 127.8, 127.2, 127.1, 59.9, 59.7, 55.7, 55.5, 54.6, 54.6, 53.3, 53.2, 49.9, 48.7, 41.5, 41.2, 30.3, 30.2, 26.5, 26.5, 13.3, 13.1; HRMS (ESI-TOF, *m/z*): calcd for $C_{30}H_{34}N_2O_3$ [M + H]$^+$, 471.2642; found, 471.2644.

Methyl 2-(2-(*tert*-butyl)-8-((diphenylmethylene)amino)-7,9-dimethoxy-3-oxo-2-azaspiro[4.5]deca-6,9-dien-4-yl)acetate (3q)

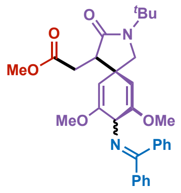

The product was obtained as a white solid (isolated yield: 45%, 1.6:1 dr). ^{1}H NMR (400 MHz, CD_3OD): δ 7.58–7.23 (m, 10H), 4.81–4.48 (m, 3H), 3.72 (s, 1.17H), 3.65–3.49 (m, 7.65H), 3.40 (s, 1.83H), 3.26 (d, *J* = 9.8 Hz, 0.36H), 3.09 (dd, *J* = 8.6, 4.2 Hz, 0.38H), 2.99 (dd, *J* = 8.3, 5.9 Hz, 0.62H), 2.77 (dd, *J* = 17.2, 4.2 Hz, 0.35H), 2.51–2.38 (m, 1H), 2.07 (dd, *J* = 15.5, 8.3 Hz, 0.61H), 1.44–1.40 (m, 9H); ^{13}C NMR (101 MHz, CD_3OD): δ 173.6, 172.8, 172.3, 172.0, 171.8, 170.3, 153.5, 153.4, 152.7, 152.5, 138.3, 138.2, 134.9, 134.9, 128.7, 128.5, 127.2, 127.1, 127.0, 126.9, 126.9, 126.7, 126.4, 126.3, 126.2, 126.1, 96.9, 96.6, 93.6, 93.2, 58.6, 58.5, 55.8, 55.2, 52.5, 52.4, 52.4, 52.3, 52.2, 50.3, 49.4, 49.2, 49.0, 42.5, 42.3, 28.4, 28.4, 25.1; HRMS (ESI-TOF, *m/z*): calcd for $C_{31}H_{36}N_2O_5$ [M + H]$^+$, 517.2697;

found, 517.2698.

Methyl 2-(2-(*tert*-butyl)-8-((diphenylmethylene)amino)-7,9-dimethyl-3-oxo-2-azaspiro[4.5]deca-6,9-dien-4-yl)acetate (3r)

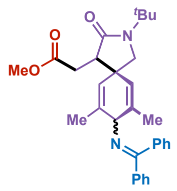

The product was obtained as a white solid (isolated yield: 58%, 1.5:1 dr). ^{1}H NMR (500 MHz, CD_3OD): δ 7.57–7.26 (m, 10H), 5.54–5.42 (m, 2H), 4.31–4.23 (m, 1H), 3.71 (s, 1.26H), 3.52–3.39 (m, 3.40H), 3.19 (d, *J* = 9.9 Hz, 0.42H), 3.02 (dd, *J* = 8.7, 4.1 Hz, 0.42H), 2.90 (dd, *J* = 8.1, 6.1 Hz, 0.58H), 2.77 (dd, *J* = 17.2, 4.1 Hz, 0.42H), 2.44–2.36 (m, 1H), 2.05–1.96 (m, 0.61H), 1.71–1.62 (m, 6H), 1.41–1.38 (m, 9H); ^{13}C NMR (126 MHz, CD_3OD): δ 176.3, 175.5, 175.0, 174.4, 173.1, 171.3, 141.1, 140.9, 137.9, 137.3, 136.6, 136.3, 136.2, 131.7, 131.4, 130.0, 129.8, 129.8, 129.7, 129.7, 129.6, 129.6, 129.5, 129.3, 129.1, 127.8, 127.7, 124.8, 124.4, 64.6, 64.4, 56.9, 56.4, 55.4, 55.3, 52.3, 52.2, 52.1, 50.7, 45.0, 44.6, 31.3, 27.9, 27.9, 21.9, 21.9, 21.9; HRMS (ESI-TOF, *m/z*): calcd for $C_{31}H_{36}N_2O_3$ $[M + H]^+$, 485.2799; found, 485.2798.

Methyl 2-(2-(*tert*-butyl)-7,9-dichloro-8-((diphenylmethylene)amino)-3-oxo-2-azaspiro[4.5]deca-6,9-dien-4-yl)acetate (3s)

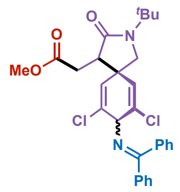

The product was obtained as a white solid (isolated yield: 39%, 1.4:1 dr). ^{1}H NMR (400 MHz, CD_3OD): δ 7.62–7.33 (m, 10H), 6.13–5.91 (m, 2H), 4.72–4.66 (m, 1H), 3.73 (s, 0.62H), 3.67–3.51 (m, 1.83H), 3.45 (s, 2.43H), 3.36 (d, *J* = 10.1 Hz, 0.22H), 3.20 (dd, *J* = 7.7, 5.2 Hz, 0.23H), 3.08 (dd, *J* = 8.9, 5.5 Hz, 0.82H), 2.72 (dd, *J* = 17.2, 5.2 Hz, 0.22H), 2.59–2.49 (m, 1H), 2.10 (dd, *J* = 15.7, 8.9 Hz, 0.79H), 1.42–1.39 (m, 9H); ^{13}C NMR (101 MHz, CD_3OD): δ 175.2, 173.6, 173.5, 172.7, 172.7, 172.4, 139.3, 139.2, 135.4, 135.3, 132.3, 132.0, 132.0, 131.4, 130.8, 130.6, 128.9, 128.7, 128.7, 128.5, 128.5, 128.3, 128.2, 128.0, 127.9, 127.8, 125.8, 125.5, 64.4, 63.8, 54.4, 54.3, 53.9, 53.4, 51.2, 51.0, 50.1, 48.6, 47.0, 46.7, 30.1, 29.8, 26.5; HRMS (ESI-TOF, *m/z*): calcd for $C_{29}H_{30}Cl_2N_2O_3$ $[M + H]^+$, 525.1706; found, 525.1702.

Methyl 2-(2-(*tert*-butyl)-8-((diphenylmethylene)amino)-6,10-dimethyl-3-oxo-2-azaspiro[4.5]deca-6,9-dien-4-yl)acetate (3t)

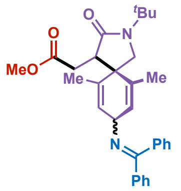

The product was obtained as a white solid (isolated yield: 45%, 1.4:1 dr). ^{1}H NMR (400 MHz, CD_3OD): δ 7.56–7.20 (m, 10H), 5.52–5.39 (m, 2H), 4.35–4.29 (m, 1H), 3.87–3.72 (m, 2.53H), 3.49–3.43 (m, 1H), 3.29–3.27 (s, 2.43H), 2.80 (dd, *J* = 17.4, 4.1 Hz, 0.35H), 2.58–2.45 (m, 1H), 1.99–1.89 (m, 3.64H), 1.75–1.71 (m, 3H), 1.45–1.41 (m, 9H); ^{13}C NMR (101 MHz, CD_3OD): δ 175.2, 174.3, 173.7, 172.8, 170.7, 169.6, 139.5, 139.3, 136.8, 136.6, 136.0, 134.8, 134.6, 134.5, 130.2, 130.0, 128.6, 128.6, 128.5, 128.4, 128.2, 127.8, 127.7, 127.4, 127.3, 126.8, 126.6, 126.3, 126.1, 56.8, 56.8, 54.5, 54.4, 51.4, 51.1, 50.9, 50.6, 46.0, 45.9, 45.6, 44.9, 30.0, 29.6, 26.4, 26.4, 20.1, 20.0, 18.4, 18.2; HRMS (ESI-TOF, *m/z*): calcd for $C_{31}H_{36}N_2O_3$ $[M + H]^+$, 485.2799; found, 485.2798.

Methyl 2-(2-(*tert*-butyl)-8-((diphenylmethylene)amino)-1,3-dioxo-2-azaspiro[4.5]deca-6,9-dien-4-yl)acetate (3u)

The product was obtained as a white solid (isolated yield: 43%, 3:1 dr). ***Major product.*** ^{1}H NMR

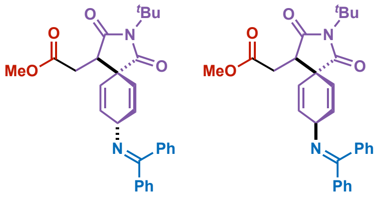

major

minor

(500 MHz, CD_2Cl_2): δ 7.65–7.19 (m, 10H), 5.87 (ddd, *J* = 9.8, 5.6, 2.6 Hz, 2H), 5.72 (dt, *J* = 9.9, 1.9 Hz, 1H), 5.47 (d, *J* = 9.9 Hz, 1H), 4.54 (s, 1H), 3.72 (s, 3H), 3.27 (dd, *J* = 7.4, 5.6 Hz, 1H), 2.69 (qd, J = 17.1, 6.4 Hz, 2H), 1.53 (s, 9H); ^{13}C NMR (126 MHz, CD_2Cl_2): δ 178.2, 178.1, 172.3, 169.9, 139.8, 136.8, 132.7, 131.6, 130.7, 129.2, 129.0, 128.9, 128.4, 127.9, 126.6, 123.5, 58.9, 55.5, 52.3, 51.3, 47.9, 30.5, 28.4. HRMS (ESI-TOF, *m/z*): calcd for $C_{29}H_{30}N_2O_4$ $[M + H]^+$, 471.2278; found, 471.2266.

Minor product. ^{1}H NMR (500 MHz, CD_3OD): δ 7.60–7.24 (m, 10H), 5.96 (dt, *J* = 10.3, 2.2 Hz, 1H), 5.87–5.77 (m, 2H), 5.59 (dt, *J* = 10.2, 2.2 Hz, 1H), 4.42 (p, *J* = 2.5 Hz, 1H), 3.37 (s, 3H), 3.20 (dd, *J* = 8.3, 5.5 Hz, 1H), 2.63 (dd, *J* = 16.5, 5.5 Hz, 1H), 2.33 (dd, *J* = 16.5, 8.3 Hz, 1H), 1.58 (s, 9H); ^{13}C NMR (126 MHz, CD_3OD): δ 179.8, 178.9, 173.4, 172.8, 140.7, 138.0, 133.9, 131.7, 131.4, 130.0, 130.0, 129.9, 129.2, 128.8, 127.8, 124.6, 59.6, 56.7, 52.2, 51.6, 31.3, 28.6, 28.5; HRMS (ESI-TOF, *m/z*): calcd for $C_{29}H_{30}N_2O_4$ $[M + H]^+$, 471.2278; found, 471.2268.

Methyl 2-(8-((diphenylmethylene)amino)-2-isopropyl-1,3-dioxo-2-azaspiro[4.5]deca-6,9-dien-4-yl)acetate (3v)

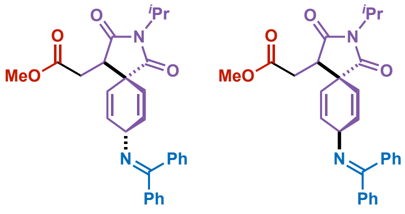

The product was obtained as a white solid (isolated yield: 29%, 4:1 dr).

Major isomer A: ^{1}H NMR (400 MHz, CD_3OD): δ 7.66–7.23 (m, 10H), 5.95–5.77 (m, 3H), 5.62 (dt, *J* = 9.8, 1.9 Hz, 1H), 4.56 (dq, *J* = 3.3, 1.8 Hz, 1H), 4.31 (p, *J* = 6.9 Hz, 1H), 3.72 (s, 3H), 3.39 (dd, *J* = 7.0, 5.7 Hz, 1H), 2.93–2.73 (m, 2H), 1.35 (dd, *J* = 7.0, 2.8 Hz, 6H); ^{13}C NMR (101 MHz, CD_3OD): δ 177.7, 177.2, 172.4, 171.4, 139.3, 136.4, 131.5, 130.3, 130.2, 128.8, 128.6, 128.4, 127.8, 127.3, 126.4, 123.1, 55.2, 51.2, 50.3, 44.0, 29.5, 18.2, 17.7. HRMS (ESI-TOF, *m/z*): calcd for $C_{28}H_{28}N_2O_4$ $[M + H]^+$, 457.2122; found, 457.2111.

Minor isomer B: ^{1}H NMR (400 MHz, CD_3OD): δ 7.49–7.14 (m, 10 H), 5.86 (dt, *J* = 10.1, 2.2 Hz, 1H), 5.74 (qt, *J* = 10.0, 2.2 Hz, 2H), 5.50 (dt, *J* = 10.2, 2.2 Hz, 1H), 4.32 (p, *J* = 2.4 Hz, 1H), 4.26 (p, *J* = 6.9 Hz, 1H), 3.26 (s, 3H), 3.13 (dd, *J* = 8.7, 5.1 Hz, 1H), 2.57 (dd, *J* = 16.7, 5.1 Hz, 1H), 2.32 (dd, *J* = 16.7, 8.7 Hz, 1H), 1.29 (dd, *J* = 6.9, 2.0 Hz, 6H); ^{13}C NMR (101 MHz, CD_3OD): δ 177.5, 176.7, 171.9, 171.4, 139.3, 136.5, 132.5, 130.3, 130.0, 128.6, 128.6, 128.5, 127.8, 127.4, 126.1, 123.0, 55.2, 50.8, 49.7, 44.0, 29.8, 18.2, 17.7; HRMS (ESI-TOF, *m/z*): calcd for $C_{28}H_{28}N_2O_4$ $[M + H]^+$, 457.2122; found, 457.2111.

2-(*tert*-Butyl)-4-(5-(2,5-dimethylphenoxy)-2,2-dimethylpentyl)-8-((diphenylmethylene)amino)-2-azaspiro[4.5]deca-6,9-dien-3-one (3w)

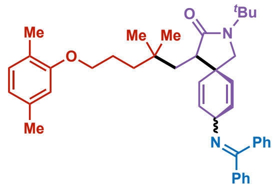

The product was obtained as a white solid (isolated yield: 50%, 1.6:1 dr). ^{1}H NMR (500 MHz, CD_3OD): δ 7.61–7.16 (m, 10H), 6.95–6.91 (m, 1H), 6.66–6.57 (m, 2H), 5.83–5.61 (m, 4H), 4.47–4.40 (m, 1H), 3.90–3.77 (m, 2H), 3.52–3.37 (m, 1.46H), 3.15 (d, *J* = 9.8 Hz, 0.53H), 2.45–2.38 (m, 1H), 2.28–2.24 (m, 3H), 2.10–2.07 (m, 3H), 1.84 (ddt, *J* = 23.1, 10.0, 6.3 Hz, 1H), 1.67–1.17 (m, 14H), 0.95–0.93 (d, *J* = 7.9 Hz, 3H), 0.79–0.75 (m, 3H); ^{13}C NMR (126 MHz, CD_3OD): δ 178.0, 177.6, 172.7, 170.8, 158.5, 158.4, 140.9, 140.7, 138.1, 137.8, 137.6, 137.5, 133.1, 132.6, 131.7, 131.4, 131.2, 131.2, 130.5, 130.2, 130.1, 129.9, 129.9, 129.8, 129.8, 129.8, 129.7, 129.5, 129.2, 129.0, 128.7, 128.7, 124.4, 124.4, 121.7, 121.6, 113.1, 113.0, 69.9, 69.6, 57.9, 57.3, 56.4, 55.9, 55.2, 55.1, 50.6, 50.1, 45.1, 45.0, 39.8, 39.7, 37.6, 37.3, 33.5, 33.1, 28.0, 28.0, 27.7, 27.7, 27.6, 27.5, 25.5, 25.3,

21.5, 16.1; HRMS (ESI-TOF, *m/z*): calcd for $C_{41}H_{50}N_2O_2$ [M + H]$^+$, 603.3945; found, 603.3938.

2-(*tert*-butyl)-4-(2-(4-(4-chlorobenzoyl)phenoxy)-2-methylpropyl)-8-((diphenylmethylene)amino)-2-azaspiro[4.5]deca-6,9-dien-3-one (3x)

Major isomer. The product was obtained as yellowish solid (isolated yield: 22.8%). R_f 0.20 (hexane/EtOAc 4:1). ^{1}H NMR (500 MHz, CD_3OD): δ 7.74 (d, *J* = 8.5 Hz, 2H), 7.58 (d, *J* = 8.7 Hz, 2H), 7.56 (d, *J* = 8.6 Hz, 2H), 7.53 – 7.49 (m, 2H), 7.48 – 7.38 (m, 4H), 7.34 (t, *J* = 7.6 Hz, 2H), 7.24 – 7.15 (m, 2H), 6.95 (d, *J* = 8.7 Hz, 2H), 5.80 – 5.72 (m, 3H), 5.61 – 5.52 (m, 1H), 4.43 (dt, *J* = 3.1, 1.5 Hz, 1H), 3.54 (d, *J* = 10.0 Hz, 1H), 3.45 (d, *J* = 10.0 Hz, 1H), 2.71 (dd, *J* = 5.4, 3.6 Hz, 1H), 1.94 (dd, *J* = 14.6, 5.4 Hz, 1H), 1.65 (dd, *J* = 14.6, 3.6 Hz, 1H), 1.42 (s, 9H), 1.32 (s, 6H). ^{13}C NMR (126 MHz, CD_3OD) δ 196.2, 177.0, 172.7, 161.9, 140.7, 139.6, 137.9, 137.8, 132.9, 132.7, 132.4, 131.8, 131.7, 130.1, 129.9, 129.8, 129.74, 129.67, 129.2, 128.7, 122.4, 82.2, 57.9, 56.1, 55.3, 50.7, 44.8, 38.4, 28.0, 27.7, 27.3. HRMS (ESI-TOF, *m/z*): calcd for $C_{43}H_{43}ClN_2O_3$ [M + H]$^+$, 671.3035; found, 671.3042.

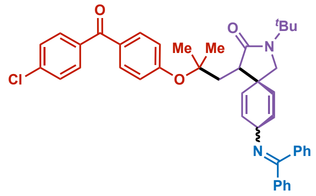

Minor isomer. The product was obtained as yellowish solid (isolated yield: 17.3%). R_f 0.32 (hexane/EtOAc 4:1). ^{1}H NMR (500 MHz, CD_3OD): δ 7.75 – 7.68 (m, 4H), 7.68 – 7.58 (m, 4H), 7.57 – 7.43 (m, 6H), 7.29 – 7.23 (m, 2H), 7.23 – 7.14 (m, 2H), 6.95 – 6.85 (m, 2H), 5.80 – 5.70 (m, 2H), 5.70 – 5.63 (m, 1H), 5.55 (ddd, *J* = 10.0, 3.7, 1.7 Hz, 1H), 4.42 (q, *J* = 2.7, 1.9 Hz, 1H), 3.45 (d, *J* = 9.9 Hz, 1H), 3.18 (d, *J* = 9.9 Hz, 1H), 2.76 (dd, *J* = 6.1, 2.5 Hz, 1H), 2.28 (dd, *J* = 14.7, 2.5 Hz, 1H), 2.01 (dd, *J* = 14.7, 6.1 Hz, 1H), 1.48 (s, 3H), 1.46 (s, 3H), 1.39 (s, 9H). ^{13}C NMR (126 MHz, CD_3OD) δ 196.34, 196.25, 177.3, 171.0, 164.2, 162.2, 140.8, 139.4, 139.2, 138.3, 138.0, 137.9, 133.9, 132.8, 132.4, 132.2, 131.4, 130.5, 122.6, 116.4, 82.6, 57.3, 56.4, 55.2, 50.0, 45.1, 37.9, 28.4, 28.1, 27.9, 27.5. HRMS (ESI-TOF, *m/z*): calcd for $C_{43}H_{43}ClN_2O_3$ [M + H]$^+$, 671.3035; found, 671.3040.

2-(tert-butyl)-4-(2-(4-(2,2-dichlorocyclopropyl)phenoxy)-2-methylpropyl)-8-((diphenylmethylene)-amino)-2-azaspiro[4.5]deca-6,9-dien-3-one (3y)

The target compounds were obtained as colorless oil with a total isolated yield of 38% and with a diastereomeric ratio of 1:1. R_f 0.35 (hexane/EtOAc 4:1). ^{1}H NMR (500 MHz, CD_3OD): δ 7.59 – 7.49 (m, 5H), 7.49 – 7.38 (m, 2H), 7.35 (s, 2H), 7.28 – 7.22 (m, 2H), 7.04 – 6.98 (m, 2H), 6.84 – 6.77 (m, 2H), 5.83 – 5.72 (m, 3H), 5.67 – 5.59 (m, 1H), 4.52 – 4.45 (m, 1H), 3.53 (d, *J* = 10.0 Hz, 1H), 3.45 (dd, *J* = 10.0, 1.9 Hz, 1H), 2.95 – 2.85 (m, 1H), 2.73 – 2.65 (m, 1H), 2.00 – 1.94 (m, 1H), 1.92 –

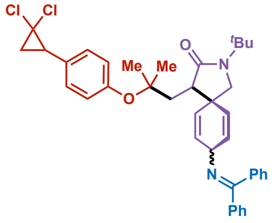

1.83 (m, 2H), 1.57 – 1.48 (m, 1H), 1.42 (s, 9H), 1.18 (s, 3H), 1.16 (s, 3H). ^{13}C NMR (126 MHz, CD_3OD) δ 180.7, 177.1, 172.7, 172.6, 156.21, 156.16, 140.8, 140.1, 138.8, 137.9, 137.9, 133.8, 133.02, 132.97, 131.8, 131.6, 131.0, 130.5, 130.3, 130.2, 130.03, 129.98, 129.8, 129.74, 129.73, 129.68, 129.64, 129.62, 129.52, 129.50, 129.2, 128.8, 124.2, 124.1, 80.91, 80.88, 62.4, 62.3, 57.91, 57.90, 56.1, 55.3, 50.7, 44.8, 38.8, 38.7, 36.0, 28.0, 27.83, 27.82, 27.09, 27.00, 26.3. HRMS (ESI-TOF, *m/z*): calcd for $C_{39}H_{42}Cl_2N_2O_2$ [M + H]$^+$, 641,2696; found, 641.2701.

2-(*tert*-Butyl)-8-((diphenylmethylene)amino)-4-(3-(4,5-diphenyloxazol-2-yl)propyl)-2-azaspiro-[4.5]deca-6,9-dien-3-one (3z)

The product was obtained as a white solid (isolated yield: 40%, 1.6:1 dr).

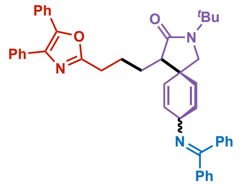

^{1}H NMR (500 MHz, CD_3OD): δ 7.55–7.15 (m, 20H), 5.88–5.64 (m, 4H), 4.47–4.42 (m, 1H), 3.49–3.35 (m, 1.62H), 3.13 (d, *J* = 10.0 Hz, 0.40H), 2.92 (t, *J* = 7.6 Hz, 0.81H), 2.72 (tdd, *J* = 15.2, 11.6, 6.9 Hz, 1.23H), 2.51 (dd, *J* = 8.7, 5.5 Hz, 0.42H), 2.41 (dd, *J* = 7.8, 6.0 Hz, 0.60H), 2.25 (ddt, *J* = 15.2, 13.1, 6.6 Hz, 0.41H), 2.10 (ddt, *J* = 17.4, 13.7, 7.4 Hz, 0.42H), 1.90–1.63 (m, 2,27H), 1.44–1.23 (d, *J* = 21.3 Hz, 10H); ^{13}C NMR (126 MHz, CD_3OD): δ 177.2, 176.9, 172.7, 171.0, 165.6, 165.2, 146.8, 146.7, 140.8, 138.0, 137.7, 135.8, 135.8, 133.5, 133.4, 133.3, 132.8, 131.7, 131.3, 131.0, 130.1, 130.0, 130.0, 129.9, 129.8, 129.8, 129.7, 129.7, 129.7, 129.6, 129.5, 129.5, 129.4, 129.4, 129.4, 129.3, 129.2, 129.1, 129.1, 129.1, 128.7, 128.7, 127.5, 127.5, 57.7, 57.3, 56.9, 56.3, 55.3, 55.2, 53.6, 53.1, 43.3, 43.1, 28.8, 28.6, 27.9, 27.9, 27.0, 26.9, 26.1, 25.9; HRMS (ESI-TOF, *m/z*): calcd for $C_{44}H_{43}N_3O_2$ [M + H]$^+$, 646.3428; found, 646.3423.

(5R)-2-((2-(*tert*-butyl)-8-((diphenylmethylene)amino)-3-oxo-2-azaspiro[4.5]deca-6,9-dien-4-yl)methyl)-3,3-dimethyl-4-thia-1-azabicyclo[3.2.0]heptan-7-one 4,4-dioxide (3aa)

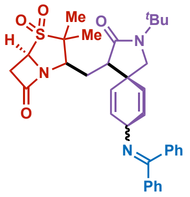

The product was obtained as colorless oil with a total yield of 19% and a mixture of four diastereomers (two major isomers (1:2.1) and minor indistinguishable isomers). R_f 0.13 (hexane/EtOAc 2:1). ^{1}H NMR (500 MHz, CD_3OD): δ 7.60 – 7.18 (m, 31H), 5.89 – 5.67 (m, 13H), 5.65 (s, 2H), 5.56 (s, 1H), 5.20 (dd, *J* = 5.3, 2.2 Hz, 2H), 5.10 (dd, *J* = 5.2, 2.1 Hz, 1H), 4.57 – 4.53 (m, 1H), 4.53 – 4.51 (m, 2H), 3.69 – 3.60 (m, 3H), 3.51 (d, *J* = 10.1 Hz, 3H), 3.29 – 3.20 (m, 6H), 3.17 – 3.10 (m, 6H), 2.69 (dd, *J* = 15.1, 5.1 Hz, 1H), 2.61 (dd, J = 14.8, 4.7 Hz, 2H), 1.63 (s, 9H), 1.57 (s, 9H), 1.43 (s, 19H), 1.43 (s, 9H). ^{13}C NMR (126 MHz,

CD$_3$OD) δ 173.16, 173.06, 165.72, 165.67, 140.73, 137.80, 137.77, 136.80, 136.44, 133.80, 132.06, 131.74, 131.45, 131.27, 131.19, 131.04, 130.99, 130.90, 130.25, 130.23, 130.07, 130.02, 129.90, 129.80, 129.59, 129.51, 129.23, 129.21, 128.73, 128.70, 128.22, 128.15, 117.62, 117.48, 69.17, 68.67, 57.57, 57.51, 56.08, 56.04, 55.76, 50.39, 50.14, 43.84, 43.79, 40.20, 38.92, 27.88, 27.84, 22.35, 22.30, 19.05, 19.00. HRMS (ESI-TOF, *m/z*): calcd for $C_{34}H_{39}N_3O_4S$ [M + H]$^+$, 586,2734; found, 586.2739.

(1r,4R)-*N*-(1-2-(*tert*-butyl)-8-((diphenylmethylene)amino)-3-oxo-2-azaspiro[4.5]deca-6,9-dien-4-yl)-3-phenylpropan-2-yl)-4-isopropylcyclohexane-1-carboxamide (3ab)

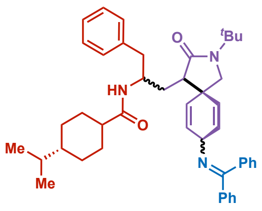

The product was obtained as colorless oil (mixture of three isomers 1.0:1.3:3.0, isolated yield: 33%). R$_f$ 0.17 (hexane/EtOAc 4:1). ^{1}H NMR (500 MHz, CD$_3$OD): δ 7.51 – 7.28 (m, 33H), 7.27 – 7.21 (m, 6H), 7.20 – 6.88 (m, 47H), 6.83 – 6.65 (m, 15H), 5.63 – 5.44 (m, 15H), 5.34 (dt, *J* = 10.2, 1.9 Hz, 3H), 5.26 (ddd, J = 10.2, 3.3, 1.9 Hz, 3H), 4.33 – 4.30 (m, 1.3H), 4.28 (dt, *J* = 3.3, 1.8 Hz, 1H), 4.24 (tt, *J* = 3.2, 1.4 Hz, 3H), 4.12 – 4.00 (m, 2.3H), 3.67 – 3.56 (m, 3H), 3.31 (d, *J* = 10.1 Hz, 1.3H), 3.24 (d, *J* = 10.1 Hz, 4.3H), 3.18 (d, *J* = 10.1 Hz, 4H), 2.95 (d, *J* = 10.0 Hz, 1H), 2.80 (dd, *J* = 13.7, 5.5 Hz, 1H), 2.61 – 2.50 (m, 6H), 2.43 (dd, *J* = 13.8, 8.9 Hz, 1.3H), 2.30 – 2.23 (m, 3H), 2.16 (td, *J* = 8.8, 4.6 Hz, 6H), 1.94 – 1.81 (m, 5H), 1.67 – 1.31 (m, 38H), 1.24 (s, 12H), 1.20 (s, 36H), 1.17 (s, 9H), 1.06 – 0.94 (m, 9H), 0.88 – 0.75 (m, 21H), 0.70 (d, *J* = 6.9 Hz, 18H), 0.68 (d, *J* = 6.7 Hz, 14H). ^{13}C NMR (126 MHz, CD$_3$OD) δ 178.84, 178.82, 178.62, 177.28, 177.15, 176.86, 172.63, 172.57, 171.17, 140.96, 140.93, 140.83, 139.97, 139.66, 139.64, 138.83, 138.13, 137.97, 137.87, 133.79, 132.66, 132.31, 132.05, 131.66, 131.61, 131.37, 130.99, 130.95, 130.84, 130.61, 130.35, 130.28, 130.24, 130.10, 130.08, 130.00, 129.98, 129.88, 129.80, 129.76, 129.70, 129.51, 129.35, 129.31, 129.25, 129.21, 129.19, 129.10, 129.07, 129.04, 128.98, 128.78, 128.74, 127.10, 127.07, 127.05, 125.62, 57.67, 57.58, 57.41, 56.72, 56.21, 55.36, 55.33, 55.30, 51.64, 51.49, 50.94, 50.60, 50.55, 50.29, 47.02, 46.87, 46.85, 44.83, 44.81, 44.76, 43.88, 43.81, 43.49, 42.99, 42.12, 41.05, 34.08, 34.06, 32.04, 31.13, 31.03, 30.89, 30.82, 30.78, 30.22, 30.18, 30.16, 27.96, 27.83, 20.13. HRMS (ESI-TOF, *m/z*): calcd for $C_{45}H_{55}N_3O_2$ [M + H]$^+$, 670.4367; found, 670.4370.

N-(2-(2-(tert-butyl)-8-((diphenylmethylene)amino)-3-oxo-2-azaspiro[4.5]deca-6,9-dien-4-yl)ethyl)-4-methylbenzenesulfonamide (3ac)

The target compounds were obtained with total yield 49% and in a diastereomeric ratio of 1.3: 1 and was isolated a major isomer (28%) and minor isomer (21%).

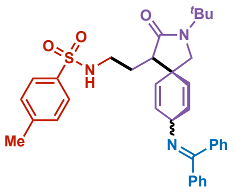

Major isomer: The product was obtained as colorless oil (isolated yield: 28%). R_f 0.33 (hexane/EtOAc 4:1). ^{1}H NMR (500 MHz, CD_3OD): δ 7.60 – 7.47 (m, 6H), 7.46 – 7.39 (m, 1H), 7.39 – 7.32 (m, 3H), 7.31 – 7.19 (m, 4H), 5.77 – 5.61 (m, 4H), 4.49 (s, 1H), 3.43 (d, *J* = 10.1 Hz, 1H), 3.39 (d, *J* = 10.1 Hz, 1H), 2.90 – 2.79 (m, 1H), 2.74 (ddd, *J* = 12.7, 7.9, 6.9 Hz, 1H), 2.41 (s, 3H), 2.28 (t, *J* = 6.9 Hz, 1H), 1.58 (dq, *J* = 14.8, 6.8 Hz, 1H), 1.39 (s, 9H), 1.20 (dq, *J* = 13.8, 6.5 Hz, 1H). ^{13}C NMR (126 MHz, CD_3OD) δ 176.5, 172.7, 144.4, 140.9, 138.9, 137.8, 132.6, 131.7, 130.6, 130.5, 130.2, 130.0, 129.8, 129.25, 129.21, 128.8, 128.0, 127.2, 57.6, 56.3, 55.4, 51.9, 43.2, 42.5, 27.9, 27.4, 21.5. HRMS (ESI-TOF, *m/z*): calcd for $C_{34}H_{37}N_3O_3S$ $[M + H]^+$, 582.2785; found, 582.2801.

Minor isomer: The product was obtained as colorless oil (isolated yield: 21%). R_f 0.41 (hexane/EtOAc 4:1). ^{1}H NMR (500 MHz, CD_3OD): δ 7.78 (d, *J* = 8.3 Hz, 1H), 7.72 (d, *J* = 8.3 Hz, 1H), 7.59 – 7.50 (m, 2H), 7.38 – 7.27 (m, 4H), 7.26 – 7.21 (m, 1H), 5.75 – 5.62 (m, 4H), 4.42 (tt, *J* = 3.2, 1.3 Hz, 1H), 3.33 (d, *J* = 10.0 Hz, 1H), 3.13 (d, *J* = 10.0 Hz, 1H), 3.09 (t, *J* = 6.7 Hz, 2H), 2.45 – 2.40 (m, 2H), 2.39 (s, 3H), 1.70 (tq, *J* = 14.3, 6.8 Hz, 2H), 1.36 (s, 9H). ^{13}C NMR (126 MHz, CD_3OD) δ 177.0, 171.7, 144.4, 144.1, 142.2, 140.8, 138.9, 138.0, 133.8, 132.4, 131.5, 131.0, 130.7, 130.5, 130.2, 129.9, 129.8, 129.51, 129.46, 129.36, 129.2, 128.7, 128.2, 127.2, 57.3, 56.7, 55.3, 51.4, 43.3, 42.7, 27.9, 27.1, 21.5. HRMS (ESI-TOF, *m/z*): calcd for $C_{34}H_{37}N_3O_3S$ $[M + H]^+$, 582.2785; found, 582.2801.

2-(tert-butyl)-8-((diphenylmethylene)amino)-4-((2,2,5,5-tetramethyltetrahydro-8aH-[1,3]dioxolo[4',5':4,5]furo[3,2-d][1,3]dioxin-8a-yl)methyl)-2-azaspiro[4.5]deca-6,9-dien-3-one (3ad)

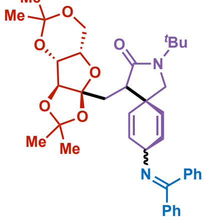

The product was obtained as yellowish oil with a total isolated yield of 38% and with a diastereomeric ratio of 1:2. R_f 0.43 (hexane/EtOAc 2:1). ^{1}H NMR (500 MHz, CD3OD): δ 7.64 –7.58 (m, 2H), 7.58 – 7.49 (m, 13H), 7.45 – 7.29 (m, 10H), 7.28 – 7.22 (m, 6H), 5.90 – 5.80 (m, 3H), 5.80 – 5.66 (m, 9H), 4.83 (s, 1H), 4.63 (s, 2H), 4.51 (tt, J = 3.0, 1.3 Hz, 2H), 4.48 – 4.43 (m, 1H), 4.24 (d, J = 2.4 Hz, 1H), 4.20 (d, J = 2.4 Hz, 2H), 4.16 (dd, J = 13.5, 2.4 Hz, 1H), 4.11 (dd, J = 13.6, 2.4 Hz, 2H), 4.01 – 3.92 (m, 2H), 3.91 – 3.80 (m, 4H), 3.49

(d, J = 10.2 Hz, 2H), 3.46 (d, J = 10.2 Hz, 3H), 3.36 (d, J = 10.0 Hz, 1H), 3.24 – 3.14 (m, 2H), 3.07 (dd, J = 6.0, 3.2 Hz, 2H), 2.38 (dd, J = 14.8, 2.0 Hz, 1H), 2.12 – 1.94 (m, 3H), 1.71 (dd, J = 14.8, 3.3 Hz, 2H), 1.46 (s, 3H), 1.44 (s, 3H), 1.41 (s, 28H), 1.36 (s, 9H), 1.33 (s, 9H), 1.29 (s, 13H), 1.28 (s, 9H). 13C NMR (126 MHz, CD3OD) δ 177.7, 177.1, 172.6, 171.1, 140.85, 140.81, 138.1, 137.9, 133.8, 132.6, 132.0, 131.6, 131.4, 131.0, 130.6, 130.4, 130.1, 130.0, 129.9, 129.80, 129.79, 129.71, 129.6, 129.5, 129.4, 129.2, 129.0, 128.7, 116.5, 116.1, 111.4, 111.1, 98.5, 87.5, 75.7, 75.4, 72.9, 72.6, 61.5, 61.4, 57.9, 57.2, 56.8, 56.3, 55.2, 55.1, 49.9, 49.5, 45.0, 44.6, 33.9, 33.8, 29.7, 29.6, 28.07, 28.06, 27.4, 27.3, 26.8, 26.7, 18.98, 18.96. HRMS (ESI-TOF, m/z): calcd for C38HN2O6 [M + H]+, 627.3429; found, 627.3430.

2-(*tert*-butyl)-8-((diphenylmethylene)amino)-2-azaspiro[4.5]deca-6,9-dien-3-one (3ae).

The products were obtained with a total isolated yield of 41% and with a diastereomeric ratio of 1:1.

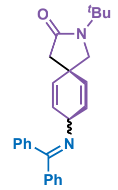

Isomer A: The product was obtained as yellowish foam (isolated yield: 21%). R_f 0.29 (hexane/EtOAc 2:1). ^{1}H NMR (500 MHz, CD_3OD): δ 9.13 – 9.02 (m, 5H), 9.00 – 8.94 (m, 1H), 8.94 – 8.87 (m, 2H), 8.85 – 8.79 (m, 2H), 7.48 (dd, J = 10.1, 1.9 Hz, 2H), 7.22 (dd, J = 10.1, 3.2 Hz, 2H), 6.00 (tt, J = 3.2, 1.9 Hz, 1H), 4.93 (s, 2H), 4.03 (s, 2H), 2.94 (s, 9H). ^{13}C NMR (126 MHz, CD_3OD) δ 175.8, 172.6, 140.7, 137.9, 132.7, 131.6, 130.1, 129.9, 129.8, 129.2, 128.7, 127.8, 58.7, 57.2, 55.4, 47.3, 38.1, 27.9. HRMS (ESI-TOF, *m/z*): calcd for $C_{26}H_{28}N_2O$ [M + H]$^+$, 385,2274; found, 385.2279.

Isomer B: The product was obtained as yellowish foam (isolated yield: 20%). R_f 0.38 (hexane/EtOAc 2:1). ^{1}H NMR (500 MHz, CD_3OD): δ 7.58 – 7.46 (m, 5H), 7.46 – 7.40 (m, 1H), 7.40 – 7.32 (m, 2H), 7.28 – 7.21 (m, 2H), 5.93 (dd, J = 10.1, 1.8 Hz, 2H), 5.66 (dd, J = 10.1, 3.4 Hz, 2H), 4.43 (tt, J = 3.4, 1.8 Hz, 1H), 3.55 (s, 2H), 2.29 (s, 2H), 1.42 (s, 9H). ^{13}C NMR (126 MHz, CD_3OD) δ 175.5, 172.8, 140.8, 137.8, 132.7, 131.7, 130.1, 129.9, 129.8, 129.2, 128.7, 127.7, 58.3, 57.3, 55.5, 47.7, 38.1, 27.9. HRMS (ESI-TOF, *m/z*): calcd for $C_{26}H_{28}N_2O$ [M + H]$^+$, 385,2274; found, 385.2282.

Methyl(2r,4r)-12-(*tert*-butyl)-8-((diphenylmethylene)amino)-13-oxo-12-azadispiro[3.0.55.34]trideca-6,9-diene-2-carboxylate (3af).

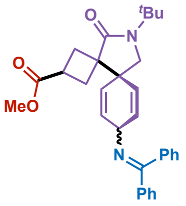

The target compounds were obtained as mixture of three isomers with total yield 36% and diastereomeric ratio of 2.3:1.3 :1. ^{1}H NMR (400 MHz, CD_3OD) δ 7.63 – 7.23 (m, 10H), 5.91 – 5.67 (m, 4H), 4.57 – 4.42 (m, 1H), 3.71 (s, 0.64H), 3.66 (s, 0.83H), 3.55 (s, 1.51H), 3.42 (s, 1H), 3.31 – 3.28(m, 1H), 3.26 – 3.10 (m, 1H), 2.71 – 2.56 (m, 1H), 2.45 (td, J = 9.7, 2.6 Hz, 0.31H), 2.25 – 2.10 (m, 2.74H), 1.44 (s, 4.39H), 1.42

(s, 2.52H), 1.38 (s, 1.94H). ^{13}C NMR (101 MHz, CD_3OD) δ 178.5, 178.0, 176.8, 175.3, 175.3, 175.2, 171.2, 170.1, 169.7, 139.5, 139.5, 139.4, 136.8, 136.7, 136.4, 130.3, 130.1, 130.0, 129.5, 129.2, 129.1, 128.8, 128.7, 128.6, 128.5, 128.5, 128.4, 128.4, 128.4, 128.3, 128.3, 128.2, 127.8, 127.8, 127.8, 127.7, 127.7, 127.4, 127.3, 127.3, 56.2, 56.0, 56.0, 53.9, 53.8, 53.7, 53.7, 53.3, 53.2, 51.3, 50.8, 50.8, 50.8, 50.8, 50.7, 48.0, 42.3, 42.2, 42.0, 31.8, 31.6, 31.5, 28.6, 28.4, 27.5, 26.5. HRMS (ESI-TOF, *m/z*): calcd for $C_{31}H_{34}N_2O_3$ $[M + H]^+$, 483.2642; found, 483.2645 .

(1-((2-(*tert*-Butyl)-8-((diphenylmethylene)amino)-3-oxo-2-azaspiro[4.5]deca-6,9-dien-4-yl)methyl)-cyclohexyl)methyl methyl carbonate (6a)

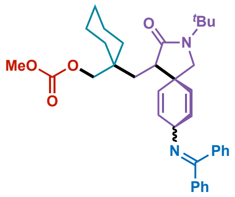

The product was obtained as a white solid (isolated yield: 50%, 1.4:1 dr). ^{1}H NMR (500 MHz, CD_3OD): δ 7.62–7.23 (m, 10H), 5.84–5.64 (m, 4H), 4.52 (dt, *J* = 3.1, 1.5 Hz, 0.61H), 4.46–4.41 (m, 0.39H), 3.92–3.71 (m, 2H), 3.70 (s, 1.1H), 3.58 (s, 1.84H), 3.52–3.39 (m, 1.62H), 3.15 (d, *J* = 10.0 Hz, 0.38H), 2.60 (dd, *J* = 6.5, 2.4 Hz, 0.38H), 2.48 (dd, *J* = 5.8, 3.1 Hz, 0.61H), 1.55–1.16 (m, 21H); ^{13}C NMR (126 MHz, CD_3OD): δ 177.8, 177.4, 172.8, 170.8, 157.6, 157.3, 141.0, 140.9, 138.1, 137.9, 132.6, 132.2, 131.7, 131.4, 130.8, 130.4, 130.2, 130.1, 130.0, 129.9, 129.9, 129.8, 129.8, 129.7, 129.7, 129.6, 129.3, 129.0, 128.8, 128.8, 74.9, 73.9, 57.9, 57.4, 56.4, 56.0, 55.2, 55.1, 50.3, 49.7, 49.3, 45.1, 44.9, 37.0, 36.7, 34.6, 33.8, 33.1, 32.4, 28.0, 28.0, 27.4, 27.1, 22.7, 22.6, 22.5, 22.5; HRMS (ESI-TOF, *m/z*): calcd for $C_{36}H_{44}N_2O_4$ $[M + H]^+$, 569.3374; found, 569.3373.

(1-((2-(*tert*-Butyl)-8-((diphenylmethylene)amino)-3-oxo-2-azaspiro[4.5]deca-6,9-dien-4-yl)methyl)-cyclohexyl)methyl ethyl carbonate (6b)

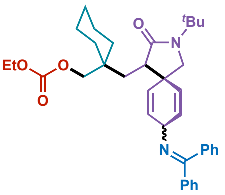

The product was obtained as a white solid (isolated yield: 54%, 1.3:1 dr). ^{1}H NMR (400 MHz, CD_3OD): δ 7.64–7.24 (m, 10H), 5.89–5.68 (m, 4H), 4.55 (tt, *J* = 3.1, 1.6 Hz, 0.62H), 4.45 (qd, *J* = 2.6, 1.7, 1.1 Hz, 0.37H), 4.20–3.82 (m, 4H), 3.55–3.39 (m, 1.63H), 3.17 (d, *J* = 9.9 Hz, 0.36H), 2.62 (dd, *J* = 6.5, 2.4 Hz, 0.37H), 2.52 (dd, *J* = 5.9, 3.0 Hz, 0.63H), 1.54–1.16 (m, 24H); ^{13}C NMR (101 MHz, CD_3OD): δ 176.4, 176.0, 171.4, 169.4, 155.6, 155.3, 139.6, 139.4, 136.7, 136.4, 131.3, 130.8, 130.3, 130.0, 129.4, 129.0, 128.8, 128.7, 128.6, 128.5, 128.4, 128.4, 128.3, 128.3, 127.9, 127.6, 127.4, 127.3, 73.3, 72.4, 63.3, 63.3, 56.5, 56.0, 54.9, 54.5, 53.8, 53.6, 48.4, 47.9, 43.7, 43.6, 35.6, 35.3, 33.2, 32.5, 31.5, 30.9, 26.6, 26.6, 26.0, 25.7, 21.3, 21.2, 21.1, 21.1, 13.3, 13.3. HRMS (ESI-TOF, *m/z*): calcd for $C_{37}H_{46}N_2O_4$ $[M + H]^+$, 583.3530; found, 583.3531.

Butyl ((1-((2-(*tert*-butyl)-8-((diphenylmethylene)amino)-3-oxo-2-azaspiro[4.5]deca-6,9-dien-4-yl)methyl)cyclohexyl)methyl) carbonate (6c)

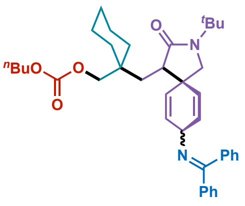

The product was obtained as a white solid (isolated yield: 51%, 1.5:1 dr). ^{1}H NMR (400 MHz, CD_3OD): δ 7.64–7.17 (m, 10H), 5.85–5.61 (m, 4H), 4.53 (tt, *J* = 3.0, 1.6 Hz, 0.65H), 4.43 (dd, *J* = 3.0, 1.8 Hz, 0.33H), 4.13–3.80 (m, 4H), 3.52–3.38 (m, 1.69H), 3.15 (d, *J* = 9.9 Hz, 0.34H), 2.60 (dd, *J* = 6.5, 2.4 Hz, 0.32H), 2.50 (dd, *J* = 5.9, 3.0 Hz, 0.67H), 1.63–1.18 (m, 28H); ^{13}C NMR (101 MHz, CD_3OD): δ 176.3, 175.9, 171.4, 169.3, 155.7, 155.5, 139.6, 139.4, 136.7, 136.4, 131.3, 130.8, 130.3, 130.0, 129.4, 129.0, 128.8, 128.7, 128.6, 128.5, 128.4, 128.4, 128.3, 128.3, 127.9, 127.6, 127.4, 127.3, 73.2, 72.6, 67.2, 67.2, 56.5, 56.0, 54.9, 54.5, 53.8, 53.6, 48.4, 47.9, 43.7, 43.6, 35.6, 35.3, 33.2, 32.6, 31.4, 31.0, 30.5, 26.6, 26.0, 25.7, 21.3, 21.2, 21.1, 21.1, 18.6, 18.6. HRMS (ESI-TOF, *m/z*): calcd for $C_{39}H_{50}N_2O_4$ $[M + H]^+$, 6112.3843; found, 611.3844.

(4-((2-(*tert*-Butyl)-8-((diphenylmethylene)amino)-3-oxo-2-azaspiro[4.5]deca-6,9-dien-4-yl)methyl)-tetrahydro-2H-pyran-4-yl)methyl methyl carbonate (6d)

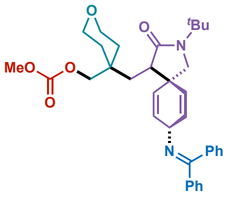

6d (major)

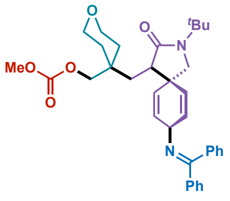

6d (minor)

The product was obtained as a white solid (isolated yield: 38%, 1.5:1 dr).

Major isomer: ^{1}H NMR (500 MHz, CD_3OD): δ 7.59–7.27 (m, 10H), 5.86–5.68 (m, 4H), 4.55 (dt, *J* = 3.3, 1.6 Hz, 1H), 3.96–3.85 (m, 2H), 3.75–3.65 (m, 2H), 3.62–3.45 (m, 7H), 2.53 (dd, *J* = 5.9, 2.9 Hz, 1H), 1.74 (dd, *J* = 14.9, 5.8 Hz, 1H), 1.53 (ddt, *J* = 14.0, 7.7, 3.4 Hz, 1H), 1.46–1.24 (m, 13H); ^{13}C NMR (126 MHz, CD_3OD): δ 177.1, 172.7, 157.1, 140.9, 137.8, 132.4, 131.7, 130.5, 130.2, 130.0, 129.8, 129.7, 129.3, 128.8, 73.2, 64.5, 57.8, 56.0, 55.2, 55.2, 49.6, 44.9, 34.9, 33.7, 33.0, 31.1, 28.0. HRMS (ESI-TOF, *m/z*): calcd for $C_{35}H_{42}N_2O_5$ $[M + H]^+$, 571.3166; found, 571.3161.

Minor isomer. ^{1}H NMR (500 MHz, acetone-d_6): δ 7.55 – 7.16 (m, 10H), 5.69 – 5.56 (m, 4H), 4.29 (dt, *J* = 3.4, 1.9 Hz, 1H), 3.94 (s, 2H), 3.73 (ddt, *J* = 15.4, 9.3, 3.5 Hz, 2H), 3.57 (s, 3H), 3.49 (ddt, *J* = 21.8, 11.7, 4.7 Hz, 2H), 3.31 (d, *J* = 9.7 Hz, 1H), 3.03 (d, *J* = 9.7 Hz, 1H), 2.42 (dd, *J* = 6.8, 2.3 Hz, 1H), 1.78 (dd, *J* = 14.8, 2.3 Hz, 1H), 1.57 – 1.45 (m, 3H), 1.30 (ddtd, *J* = 19.4, 12.6, 3.5, 1.9 Hz, 2H), 1.22 (s, 9H). ^{13}C NMR (126 MHz, acetone-d_6): δ 175.6, 169.0, 156.5, 140.6, 137.8, 131.9, 131.0, 130.5, 129.7, 129.6, 129.4, 129.2, 128.8, 128.4, 74.3, 63.8, 63.8, 57.1, 55.4, 54.7, 54.1, 48.4, 44.5, 35.0, 34.1, 31.7, 27.8. HRMS (ESI-TOF, *m/z*): calcd for $C_{35}H_{42}N_2O_4$ $[M + H]^+$, 571.3166; found, 571.3168.

(1-((2-(*tert*-Butyl)-8-((diphenylmethylene)amino)-3-oxo-2-azaspiro[4.5]deca-6,9-dien-4-yl)methyl)-cyclopentyl)methyl methyl carbonate (6e)

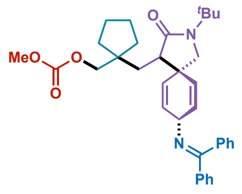

6e (major)

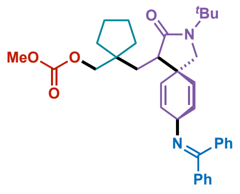

6e (minor)

Two stereoisomers of the desired product were isolated as white solids (**6e**[1], 23%; **6e**[2], 17%; combined isolated yield of **6e**, 40%, 1.3:1 dr).

Major isomer: (**6e**[1]). ^{1}H NMR (500 MHz, acetone-d_6): δ 7.68–7.27 (m, 10H), 5.86–5.63 (m, 4H), 4.43–4.36 (m, 1H), 4.10–3.97 (m, 2H), 3.68 (s, 3H), 3.42 (d, *J* = 9.7 Hz, 1H), 3.15 (d, *J* = 9.6 Hz, 1H), 2.43 (t, *J* = 4.4 Hz, 1H), 1.82–1.49 (m, 10H), 1.35 (s, 9H); ^{13}C NMR (126 MHz, acetone-d_6): δ 175.7, 168.7, 156.7, 140.6, 137.8, 132.0, 131.0, 130.3, 129.6, 129.4, 129.3, 129.1, 128.8, 128.5, 74.9, 57.0, 55.4, 54.6, 54.1, 50.1, 46.4, 44.5, 35.8, 34.4, 33.5, 27.8, 25.8, 25.8. HRMS (ESI-TOF, *m/z*): calcd for $C_{35}H_{42}N_2O_4$ [M + H]$^+$, 555.3217; found, 555.3219.

Minor isomer (**6e**[2]): ^{1}H NMR (500 MHz, CD_3OD): δ 7.57–7.25 (m, 10H), 5.82–5.66 (m, 4H), 4.50 (dt, *J* = 3.2, 1.6 Hz, 1H), 3.85 (d, *J* = 10.6 Hz, 1H), 3.76 (d, *J* = 10.6 Hz, 1H), 3.57 (s, 3H), 3.50 (d, *J* = 10.1 Hz, 1H), 3.43 (d, *J* = 10.0 Hz, 1H), 2.40 (dd, *J* = 5.7, 2.9 Hz, 1H), 1.61–1.17 (m, 19H); ^{13}C NMR (126 MHz, CD_3OD): δ 177.3, 172.7, 157.3, 140.8, 137.8, 132.4, 131.7, 130.3, 130.2, 130.0, 130.0, 129.8, 129.6, 129.2, 128.8, 74.4, 57.8, 55.9, 55.2, 55.0, 51.3, 49.5, 49.5, 49.3, 49.3, 49.2, 49.1, 49.0, 49.0, 48.8, 48.7, 48.7, 48.5, 48.5, 46.5, 44.8, 35.8, 35.2, 33.2, 28.0, 25.9, 25.7. HRMS (ESI-TOF, *m/z*): calcd for $C_{35}H_{42}N_2O_4$ [M + H]$^+$, 555.3217; found, 555.3224.

The configuration of stereoisomers **6e** was determined to be *trans* and *cis*, respectively, based on 2D NMR experiments. The major stereoisomer **6e** demonstrated NOE correlation δ_H 4.49/1.16 ppm between H-19 and H-6a and HMBC correlation from H-19 δ_H 4.49 ppm to C-5 δ_C 79.0 ppm. The minor stereoisomer **6e** demonstrated NOE correlation between protons of one of the benzene rings δ_H 7.64 ppm and protons of the cyclopentyl moiety δ_H 1.51–1.80 ppm.

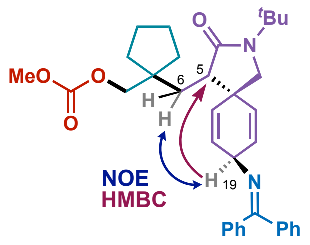

major **(22.6%)**

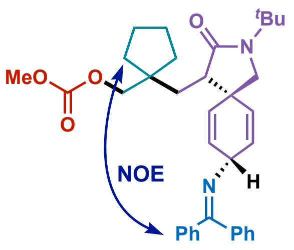

minor **(17.4%)**

(1-((2-(*tert*-Butyl)-8-((diphenylmethylene)amino)-3-oxo-2-azaspiro[4.5]deca-6,9-dien-4-yl)methyl)cyclobutyl)methyl methyl carbonate (6f).

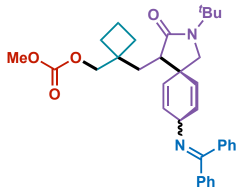

The product was obtained as a white solid (isolated yield: 41%, 1.3:1 dr). ^{1}H NMR (400 MHz, CD_3OD): δ 7.63–7.25 (m, 10H), 5.85–5.70 (m, 4H), 4.53 (tt, *J* = 2.7, 1.3 Hz, 0.6H), 4.46 (t, *J* = 2.1 Hz, 0.4H), 4.19–4.09 (m, 0.84H), 4.03–3.91 (m, 1.22H), 3.74 (s, 1.23H), 3.61 (s, 1.79H), 3.55–3.40 (m, 1.64H), 3.20 (d, *J* = 9.9 Hz, 0.40H), 2.39 (dd, *J* = 6.5, 2.7 Hz, 0.41H), 2.29 (dd, *J* = 5.8, 3.3 Hz, 0.62H), 2.02–1.71 (m, 7H), 1.42–1.39 (m, 9H), 1.34–1.28 (m, 1H); ^{13}C NMR (101 MHz, CD_3OD): δ 176.2, 175.8, 171.3, 169.7, 156.3, 156.0, 139.5, 139.5, 136.7, 136.4, 131.0, 130.8, 130.3, 130.0, 129.4, 128.8, 128.8, 128.6, 128.6, 128.5, 128.5, 128.4, 128.3, 128.1, 127.8, 127.7, 127.4, 127.3, 73.3, 72.3, 56.3, 55.9, 55.0, 54.6, 53.8, 53.7, 49.6, 49.2, 43.4, 43.1, 41.0, 40.6, 32.0, 31.7, 28.1, 28.0, 27.5, 27.4, 26.6, 26.5, 14.1, 14.0. HRMS (ESI-TOF, *m/z*): calcd for $C_{34}H_{40}N_2O_4$ [M + H]$^+$, 541.3061; found, 541.3064.

2-((2-(*tert*-Butyl)-8-((diphenylmethylene)amino)-3-oxo-2-azaspiro[4.5]deca-6,9-dien-4-yl)methyl)-2-ethylbutyl methyl carbonate (6g)

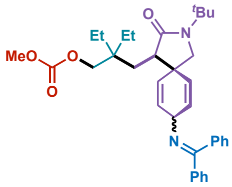

The product was obtained as a white solid (isolated yield: 42%, 1.4:1 dr). ^{1}H NMR (400 MHz, CD_3OD): δ 7.61–7.22 (m, 10H), 5.83–5.66 (m, 4H), 4.51 (tt, *J* = 3.1, 1.5 Hz, 0.64H), 4.43 (dt, *J* = 3.7, 2.2 Hz, 0.36H), 4.10–3.96 (m, 0.74H), 3.80 (s, 1.16H), 3.74–3.69 (m, 1.27H), 3.59 (s, 1.83H), 3.51–3.38 (m, 1.63H), 3.14 (d, *J* = 9.9 Hz, 0.37H), 2.63 (dd, *J* = 6.0, 3.1 Hz, 0.38H), 2.52 (dd, *J* = 5.5, 3.5 Hz, 0. 64H), 1.65–1.52 (m, 1.26H), 1.40–1.36 (m, 9H), 1.30–1.03 (m, 4.77H), 0.93–0.85 (m, 2H), 0.69 (dt, *J* = 17.8, 7.5 Hz, 4H); ^{13}C NMR (101 MHz, CD_3OD): δ 176.3, 175.9, 171.4, 169.5, 156.0, 155.8, 139.5, 139.4, 136.7, 136.4, 131.4, 130.8, 130.3, 130.0, 129.4, 128.8, 128.8, 128.7, 128.6, 128.5, 128.4, 128.3, 128.2, 128.2, 127.8, 127.6, 127.4, 127.3, 72.7, 71.5, 56.5, 55.9, 55.0, 54.6, 53.8, 53.7, 53.7, 53.6, 43.7, 43.6, 38.1, 37.8, 29.9, 29.5, 26.5, 26.5, 26.5, 25.5, 25.4, 25.2, 6.8, 6.7, 6.4, 6.3. HRMS (ESI-TOF, *m/z*): calcd for $C_{35}H_{44}N_2O_4$ [M + H]$^+$, 557.3374; found, 557.3374.

(1-((2-(*tert*-Butyl)-8-((diphenylmethylene)amino)-7,9-dimethyl-3-oxo-2-azaspiro[4.5]deca-6,9-dien-4-yl)methyl)cyclohexyl)methyl methyl carbonate (6h).

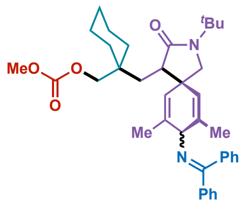

The product was obtained as a white solid (isolated yield: 45%, 1.5:1 dr). ^{1}H NMR (400 MHz, CD_3OD): δ 7.65–7.25 (m, 10H), 5.56–5.33 (m, 2H), 4.38 (s, 0.57H), 4.24 (s, 0.43H), 4.04 (s, 0.82H), 3.90 (d, *J* = 10.7 Hz, 0.60H), 3.75–3.70 (m, 2.03H), 3.55 (s, 1.73H), 3.49–3.35 (m, 1.66H), 3.12 (d, *J* = 9.8 Hz, 0.42H), 2.55 (dd, *J* = 6.4, 2.4 Hz, 0.43H), 2.43 (dd, *J* = 5.2, 3.7 Hz, 0.58H), 1.74–1.60 (m, 6H), 1.53–1.17 (m, 21H); ^{13}C NMR (101 MHz, CD_3OD): δ 176.7, 176.3, 171.8, 169.1, 156.2, 155.9, 139.9, 139.7, 136.7, 136.6, 135.7, 134.9, 134.9, 134.1, 130.2, 129.9, 128.6, 128.4, 128.4, 128.3, 128.3, 128.2, 128.2, 128.1, 127.9, 127.6, 127.5, 126.5, 124.5, 123.6, 73.9, 72.6, 63.4, 63.3, 55.2, 54.8, 53.7, 53.7, 53.6, 48.8, 48.2, 45.4, 44.9, 35.6, 35.3, 33.2, 32.1, 31.9, 30.9, 26.6, 26.0, 25.8, 21.3, 21.0, 20.8, 20.7, 20.6, 20.5. HRMS (ESI-TOF, *m/z*): calcd for $C_{38}H_{48}N_2O_4$ $[M + H]^+$, 597.3687; found, 597.3687.

(1-((2-(*tert*-Butyl)-7,9-dichloro-8-((diphenylmethylene)amino)-3-oxo-2-azaspiro[4.5]deca-6,9-dien-4-yl)methyl)cyclohexyl)methyl methyl carbonate (6i).

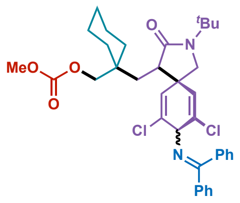

The product was obtained as a white solid (isolated yield: 31%, 1.5:1 dr). ^{1}H NMR (500 MHz, CD_3OD): δ 7.62–7.31 (m, 10H), 6.29–5.67 (m, 2H), 4.80 (d, *J* = 1.1 Hz, 0.7H), 4.22 (s, 0.3H), 4.06–3.94 (m, 1.34H), 3.71 (d, *J* = 9.9 Hz, 1.66H), 3.62–3.37 (m, 4.11H), 2.64 (t, *J* = 4.4 Hz, 0.70H), 2.22 (dd, *J* = 7.0, 2.3 Hz, 0.29H), 1.67 (dd, *J* = 14.8, 4.9 Hz, 0.72H), 1.57–1.15 (m, 21.33H); ^{13}C NMR (126 MHz, CD_3OD): δ 176.9, 176.5, 176.5, 173.3, 157.3, 157.3, 140.9, 140.5, 136.9, 136.0, 132.9, 132.9, 132.2, 132.1, 130.4, 130.4, 130.3, 130.1, 130.1, 130.1, 129.9, 129.8, 129.6, 129.3, 129.0, 128.3, 126.9, 125.3, 73.3, 73.0, 68.9, 65.6, 55.5, 55.4, 55.2, 55.2, 54.8, 51.6, 50.2, 50.1, 49.8, 49.4, 37.0, 36.9, 34.1, 33.9, 33.1, 32.6, 28.0, 27.9, 27.2, 27.1, 22.6, 22.5, 22.5, 22.4. HRMS (ESI-TOF, *m/z*): calcd for $C_{36}H_{42}$ $Cl_2N_2O_4$ $[M + H]^+$, 637.2594; found, 637.2593.

(1-((2-(*tert*-Butyl)-8-((diphenylmethylene)amino)-6,10-dimethyl-3-oxo-2-azaspiro[4.5]deca-6,9-dien-4-yl)methyl)cyclohexyl)methyl methyl carbonate (6j)

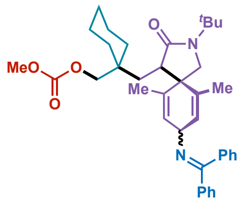

The product was obtained as a white solid (isolated yield: 37%, 1.6:1 dr). ^{1}H NMR (400 MHz, CD_3OD): δ 7.66–7.15 (m, 10H), 5.58–5.36 (m, 2H), 4.45 (td, *J* = 3.1, 1.5 Hz, 0.64H), 4.38–4.30 (m, 0.37H), 4.14–4.06 (m, 1.29H), 3.91 (d, *J* = 10.8 Hz, 0.64H), 3.81–3.73 (m, 1H), 3.70 (s, 1.04H), 3.59 (s, 1.84H), 3.41 (d, *J* = 11.0

Hz, 0.62H), 3.22 (d, *J* = 10.8 Hz, 0.37H), 2.93 (dd, *J* = 6.0, 2.5 Hz, 0.35H), 2.76 (dd, *J* = 5.5, 2.9 Hz, 0.61H), 1.94–1.86 (m, 3H), 1.72–1.65 (m, 3H), 1.50–1.19 (m, 21H); ^{13}C NMR (101 MHz, CD_3OD): δ 177.1, 176.6, 170.7, 168.3, 156.1, 155.9, 139.8, 139.5, 136.9, 136.6, 136.6, 136.5, 134.6, 134.2, 130.2, 129.8, 128.7, 128.5, 128.4, 128.4, 128.2, 128.2, 127.8, 127.5, 127.5, 127.4, 127.3, 126.9, 125.8, 125.6, 73.4, 73.0, 60.1, 57.0, 56.9, 54.2, 54.1, 53.7, 53.6, 51.2, 51.0, 43.7, 43.4, 35.7, 35.5, 33.6, 32.9, 31.3, 30.8, 26.5, 25.9, 25.7, 21.4, 21.2, 21.1, 21.0, 20.2, 20.2, 19.5, 18.5, 18.3, 13.1. HRMS (ESI-TOF, *m/z*): calcd for $C_{38}H_{48}N_2O_4$ $[M + H]^+$, 597.3687; found, 597.3685.

2-(*tert*-Butyl)-4-(cyclopentylmethyl)-8-((diphenylmethylene)amino)-2-azaspiro[4.5]deca-6,9-dien-3-one (8b)

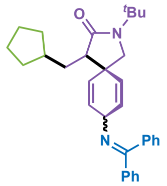

The product was obtained as a white solid (isolated yield: 30%, 1.4:1 dr). ^{1}H NMR (400 MHz, CD_3OD): δ 7.64–7.16 (m, 10H), 5.96–5.62 (m, 4H), 4.45 (ddd, *J* = 5.3, 3.2, 1.6 Hz, 1H), 3.50–3.33 (m, 2H), 3.13 (d, *J* = 9.9 Hz, 0.6H), 2.51–2.31 (m, 1.4H), 1.89–0.83 (m, 20H); ^{13}C NMR (101 MHz, CD_3OD): δ 176.6, 176.1, 171.5, 169.6, 139.6, 139.3, 136.6, 136.4, 132.1, 131.4, 130.3, 130.0, 128.7, 128.6, 128.5, 128.5, 128.4, 128.4, 128.3, 128.3, 128.2, 127.9, 127.9, 127.6, 127.6, 127.5, 127.3, 127.3, 56.6, 55.8, 55.4, 54.9, 53.8, 53.8, 51.6, 51.2, 42.0, 41.9, 37.0, 36.7, 32.7, 32.6, 32.3, 32.2, 31.6, 31.5, 26.5, 26.5, 24.9, 24.7, 24.5, 24.4. HRMS (ESI-TOF, *m/z*): calcd for $C_{32}H_{38}N_2O$ $[M + H]^+$, 467.3057; found, 467.3057.

2-(*tert*-Butyl)-4-(cycloheptylmethyl)-8-((diphenylmethylene)amino)-2-azaspiro[4.5]deca-6,9-dien-3-one (8c)

The product was obtained as a white solid (isolated yield: 32%, 1.5:1 dr).

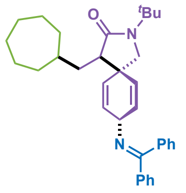

9c (major)

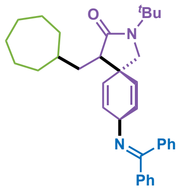

9c (minor)

Major isomer: ^{1}H NMR (500 MHz, CD_3OD): δ 7.60–7.23 (m, 10H), 5.99–5.63 (m, 4H), 4.50 (t, *J* = 2.5 Hz, 1H), 3.49–3.34 (m, 2H), 2.44 (dd, *J* = 10.1, 4.7 Hz, 1H), 1.67–0.90 (m, 24H); ^{13}C NMR (126 MHz, CD_3OD): δ 177.6, 172.8, 140.7, 137.9, 133.6, 131.7, 130.1, 130.0, 129.9, 129.8, 129.2, 129.2, 128.8, 128.7, 58.3, 56.5, 55.3, 51.3, 43.2, 37.0, 36.3, 36.1, 33.8, 29.3, 29.2, 28.1, 27.9, 27.7. HRMS (ESI-TOF, *m/z*): calcd for $C_{34}H_{42}N_2O$ $[M + H]^+$, 495.3370; found, 495.3369.

Minor isomer: ^{1}H NMR (400 MHz, CD_3OD): δ 7.74–7.18 (m, 10H), 5.84–5.60 (m, 4H), 4.43 (dq, *J* = 3.4, 1.7 Hz, 1H), 3.35 (d, *J* = 10.0 Hz, 1H), 3.13 (d, *J* = 9.9 Hz, 1H), 2.48 (dd, *J* = 7.7, 6.0 Hz, 1H), 2.06–1.91 (m, 1H), 1.84–1.13 (m, 23H); ^{13}C NMR (101 MHz, CD_3OD): δ 176.8, 169.5, 139.6, 136.7, 131.3, 130.0, 128.5, 128.5, 128.4, 128.3, 128.2, 127.7, 127.6, 127.4, 55.9, 55.4, 53.8, 49.9, 42.0, 35.3, 34.8, 33.9,

33.3, 28.4, 28.4, 26.5, 26.2, 26.0. HRMS (ESI-TOF, *m/z*): calcd for $C_{34}H_{42}N_2O$ $[M + H]^+$, 495.3370; found, 495.3370.

4-(2-(*tert*-butoxy)ethyl)-2-(*tert*-butyl)-8-((diphenylmethylene)amino)-2-azaspiro[4.5]deca-6,9-dien-3-one (8d)

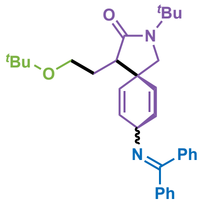

The product was obtained as a white solid (isolated yield: 33%, 1.4:1 dr). ^{1}H NMR (400 MHz, CD_3OD): δ 7.65–7.16 (m, 10H), 5.87–5.64 (m, 4H), 4.49 (tt, *J* = 3.1, 1.8 Hz, 0.62H), 4.44 (tt, *J* = 3.4, 1.6 Hz, 0.37H), 3.70–3.54 (m, 0.78H), 3.50–3.34 (m, 1.64H), 3.23 (ddd, *J* = 8.3, 6.5, 2.4 Hz, 1.23H), 3.14 (d, *J* = 9.9 Hz, 0.39H), 2.59 (dd, *J* = 8.5, 5.6 Hz, 0.37H), 2.46 (dd, *J* = 8.7, 5.5 Hz, 0.62H), 1.95–1.65 (m, 1H), 1.39 (d, *J* = 16.7 Hz, 10H), 1.21 (s, 3.35H), 1.01 (s, 5.61H); ^{13}C NMR (101 MHz, CD_3OD): δ 176.0, 175.5, 171.4, 169.7, 139.5, 139.3, 136.7, 136.5, 132.0, 131.4, 130.4, 130.0, 128.7, 128.6, 128.5, 128.4, 128.4, 128.3, 128.3, 128.1, 127.9, 127.9, 127.6, 127.6, 127.3, 127.3, 72.7, 72.6, 59.1, 59.0, 56.5, 55.9, 54.9, 53.9, 53.8, 49.4, 48.9, 41.7, 41.6, 27.3, 27.2, 26.7, 26.5, 26.5, 26.4. HRMS (ESI-TOF, *m/z*): calcd for $C_{32}H_{40}N_2O_2$ $[M + H]^+$, 485.3163; found, 485.3162.

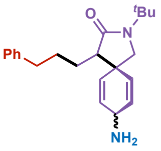

8-Amino-2-(*tert*-butyl)-4-(3-phenylpropyl)-2-azaspiro[4.5]deca-6,9-dien-3-one (9)

The product was obtained as a white solid (isolated yield: 75%, 1.4:1 dr). ^{1}H NMR (400 MHz, CD_3OD): δ 7.31–7.07 (m, 5H), 5.93–5.63 (m, 4H), 3.66 (qd, *J* = 3.0, 1.5 Hz, 1H), 3.37 (dd, *J* = 17.6, 10.0 Hz, 1H), 3.17 (dd, *J* = 19.2, 9.9 Hz, 1H), 2.71–2.43 (m, 2H), 2.39–2.26 (m, 1H), 1.81–1.18 (m, 13H); ^{13}C NMR (101 MHz, CD_3OD): δ 176.0, 175.8, 142.2, 142.2, 131.3, 131.0, 130.5, 130.3, 130.0, 130.0, 128.1, 128.0, 127.9, 127.9, 127.6, 127.5, 125.4, 125.3, 55.3, 55.1, 53.8, 53.8, 52.5, 51.9, 45.0, 44.7, 41.9, 41.8, 35.5, 35.4, 29.8, 29.3, 26.5, 25.8, 25.5. HRMS (ESI-TOF, *m/z*): calcd for $C_{22}H_{30}N_2O$ $[M + Na]^+$, 361.2248; found, 361.225.

Ethyl 2-(8-amino-2-(*tert*-butyl)-3-oxo-2-azaspiro[4.5]deca-6,9-dien-4-yl)acetate (10)

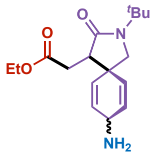

The product was obtained as a white solid (isolated yield: 73%, 1.5:1 dr). ^{1}H NMR (400 MHz, CD_3OD): δ 6.03–5.87 (m, 2H), 5.71–5.63 (m, 2H), 4.15–4.03 (m, 2H), 3.68 (dtt, *J* = 13.2, 3.4, 1.8 Hz, 1H), 3.49 (dd, *J* = 17.2, 10.1 Hz, 1H), 3.25 (dd, *J* = 20.6, 10.0 Hz, 1H), 2.97 (ddd, *J* = 7.5, 6.0, 2.1 Hz, 1H), 2.51 (ddd, *J* = 30.0, 15.9, 6.0 Hz, 1H), 2.15 (ddd, *J* = 29.2, 15.8, 7.9 Hz, 1H), 1.40–1.39 (dm, 9H), 1.24 (q, *J* = 7.1 Hz, 3H); ^{13}C NMR (101 MHz, CD_3OD): δ 173.9, 173.9, 172.5, 172.5, 131.3, 131.2, 130.8, 130.4, 129.7, 129.7, 126.6, 126.6, 60.4, 60.4, 55.2, 54.8, 54.0, 50.2,

49.0, 44.8, 44.6, 41.7, 41.6, 30.6, 30.3, 26.5, 26.5, 13.0. HRMS (ESI-TOF, *m/z*): calcd for $C_{17}H_{26}N_2O_3$ [M + Na]$^+$, 329.1836; found, 329.1821.

Ethyl 2-(8-(benzhydrylamino)-2-(*tert*-butyl)-3-oxo-2-azaspiro[4.5]deca-6,9-dien-4-yl)acetate (11)

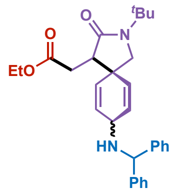

The product was obtained as a white solid (isolated yield: 81%, 1.5:1 dr). ^{1}H NMR (400 MHz, CD_3OD): δ 7.49–7.18 (m, 10H), 6.24–5.98 (m, 2H), 5.74–5.55 (m, 2H), 5.21 (s, 0.47H), 5.17 (s, 0.54H), 4.09–3.84 (m, 2H), 3.61–3.40 (m, 2H), 3.21 (dd, *J* = 28.2, 10.0 Hz, 1H), 2.94 (ddd, *J* = 19.0, 8.4, 5.4 Hz, 1H), 2.57 (dd, *J* = 16.0, 5.1 Hz, 0.46H), 2.44 (dd, *J* = 15.9, 5.8 Hz, 0.57H), 2.23 (dd, *J* = 16.0, 8.8 Hz, 0.47H), 2.05 (dd, *J* = 15.9, 8.1 Hz, 0.57H), 1.39 (d, *J* = 4.2 Hz, 9H), 1.09 (dt, *J* = 13.3, 7.1 Hz, 3H); ^{13}C NMR (101 MHz, CD_3OD): δ 174.0, 173.8, 172.6, 172.5, 143.9, 143.9, 143.9, 143.8, 130.1, 130.1, 129.8, 129.7, 129.5, 129.3, 128.1, 128.1, 127.2, 127.2, 127.1, 127.0, 126.8, 126.8, 126.7, 126.6, 63.7, 60.4, 60.3, 55.3, 54.7, 54.0, 50.4, 49.1, 48.9, 48.3, 41.9, 41.8, 30.5, 30.2, 26.5, 26.4, 13.0, 13.0. HRMS (ESI-TOF, *m/z*): calcd for $C_{30}H_{36}N_2O_3$ [M + H]$^+$, 473.2799; found, 473.2795.

7. NMR spectra of substrates and products

2-(4-(4-chlorobenzoyl)phenoxy)-1-(((diphenylmethylene)amino)oxy)-2-methylpropan-1-one (1x)

^{1}H NMR (500 MHz, $CDCl_3$)

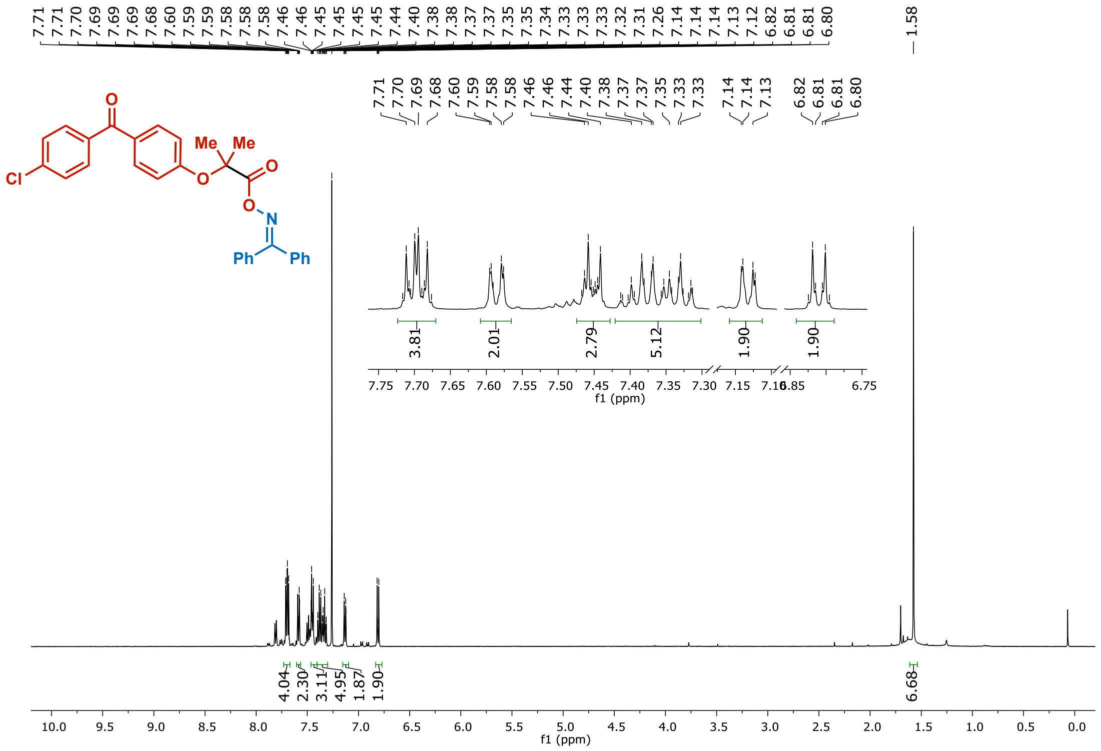

^{13}C NMR (126 MHz, $CDCl_3$)

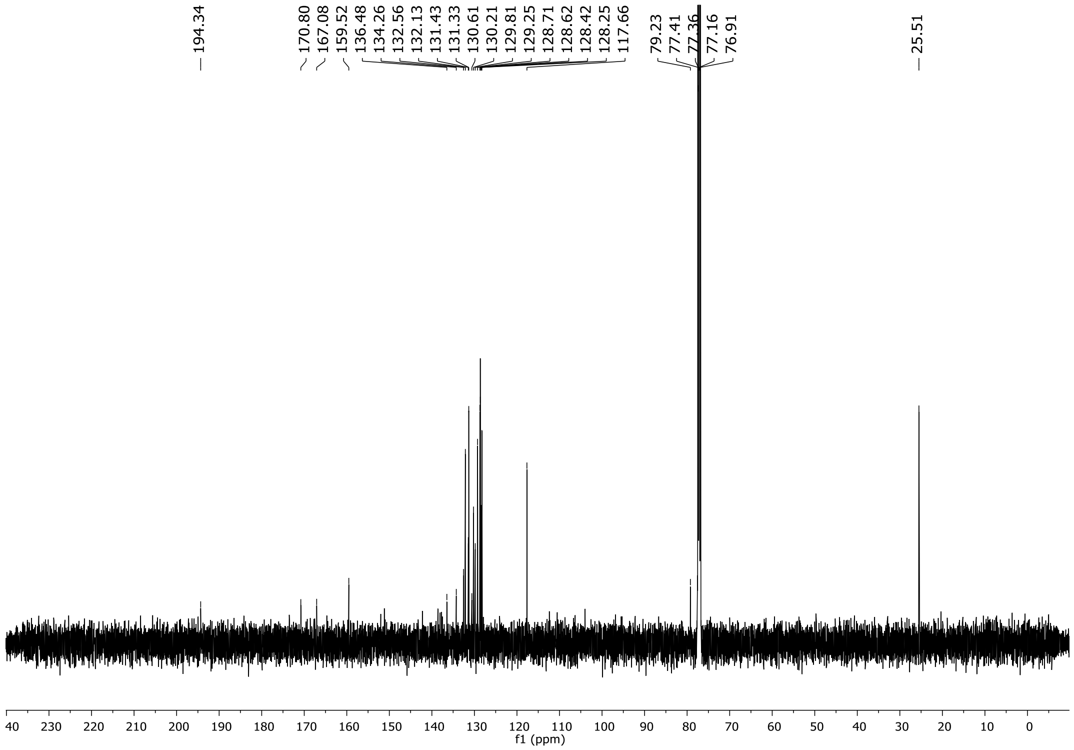

Diphenylmethanone O-(2-(4-(2,2-dichlorocyclopropyl)phenoxy)-2-methylpropanoyl) oxime (1y)

^{1}H NMR (500 MHz, CDCl$_3$)

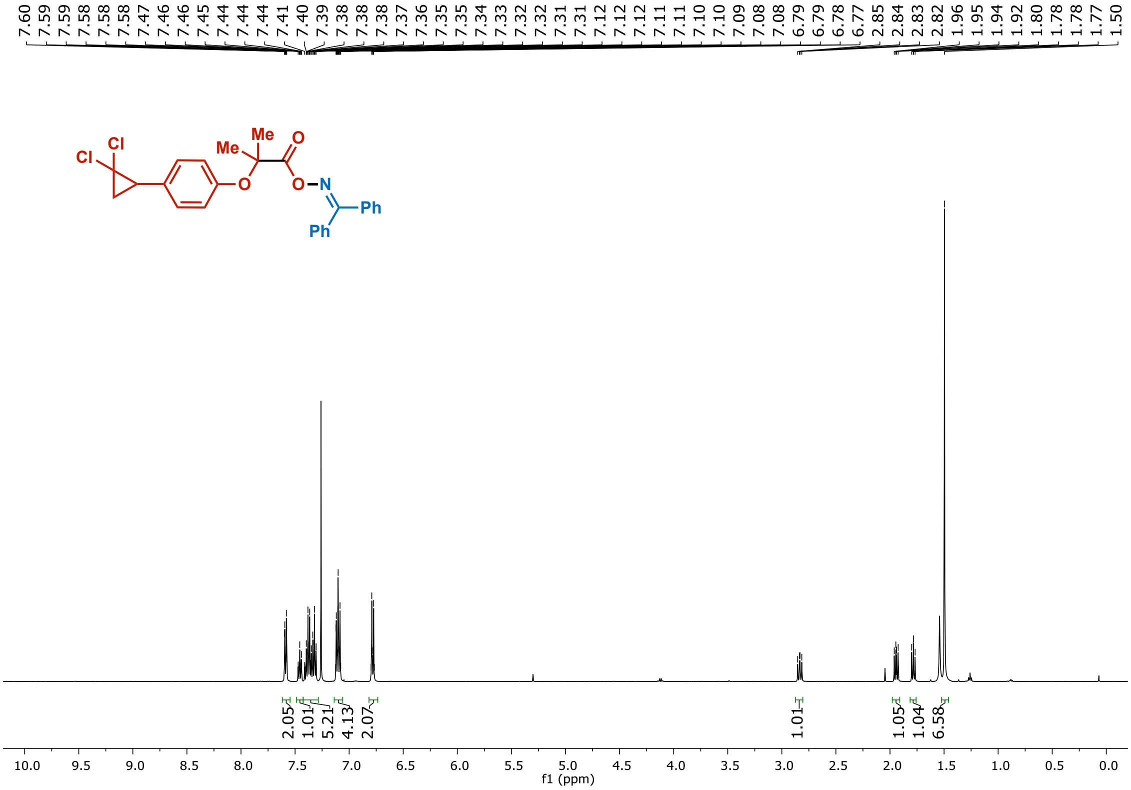

^{13}C NMR (126 MHz, CDCl$_3$)

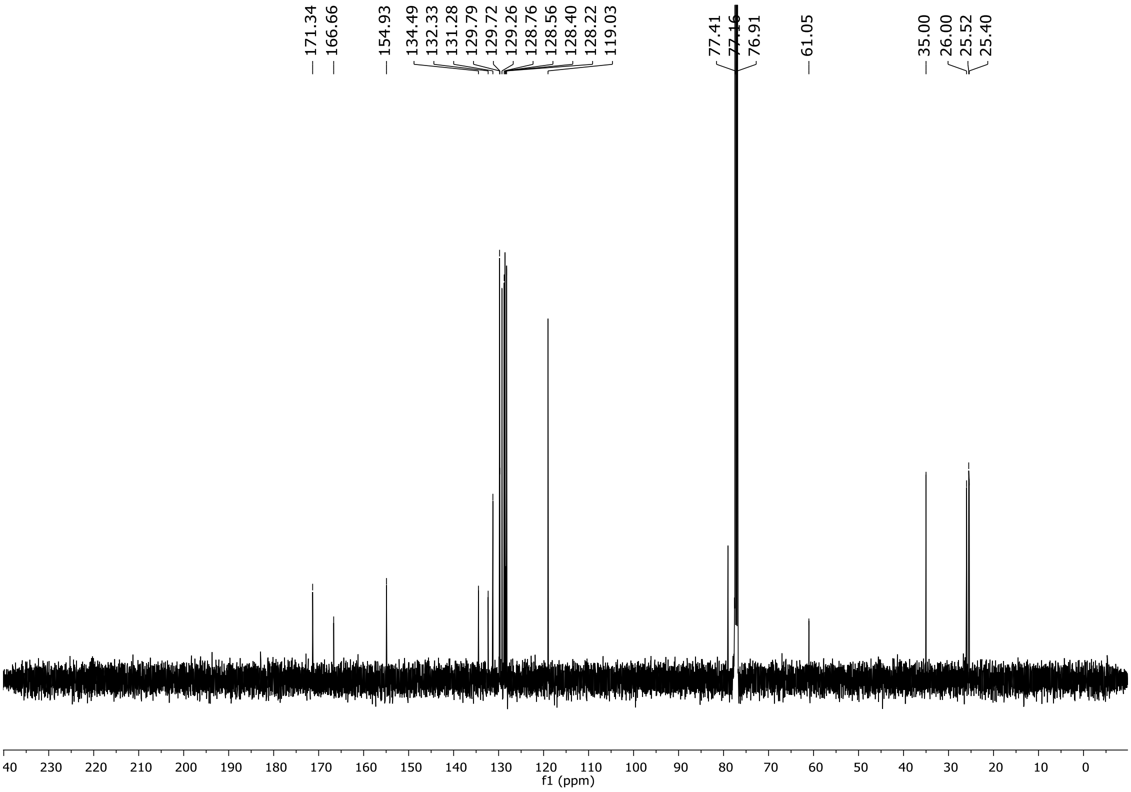

(2S,5R)-2-((((diphenylmethylene)amino)oxy)carbonyl)-3,3-dimethyl-4-thia-1-azabicyclo[3.2.0]heptan-7-one 4,4-dioxide (1aa).

^{1}H NMR (500 MHz, $CDCl_3$)

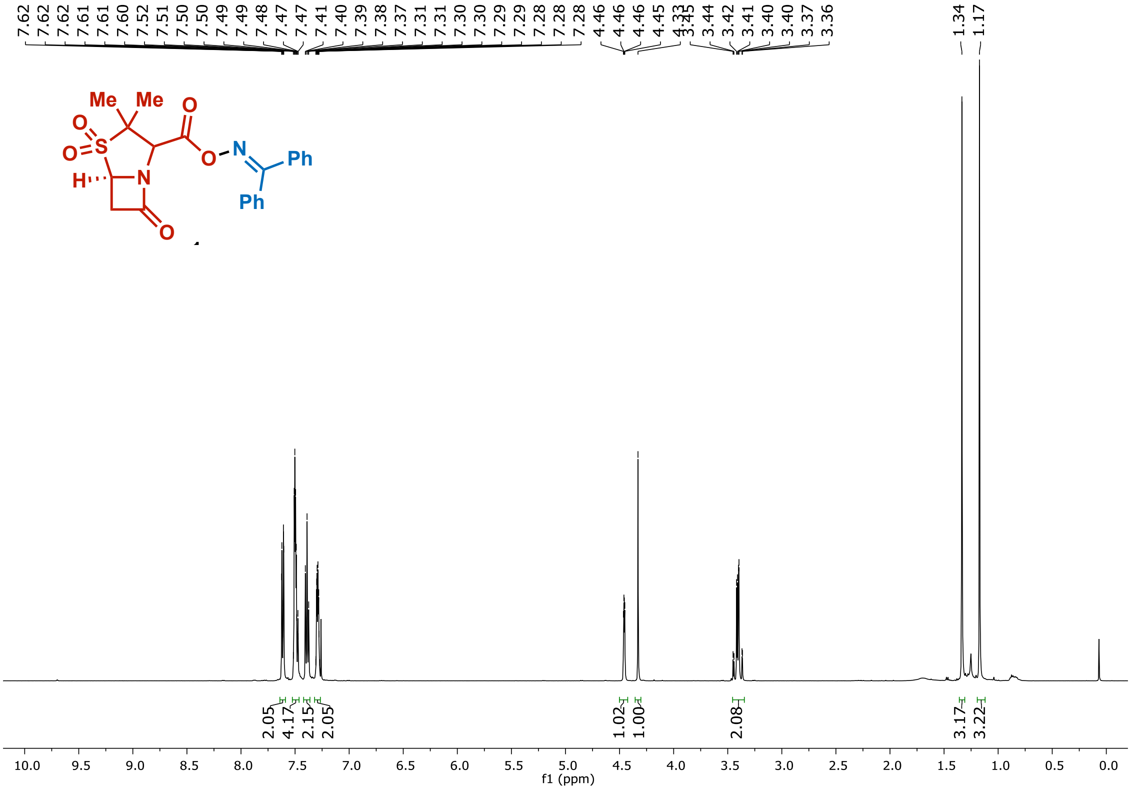

^{13}C NMR (126 MHz, $CDCl_3$)

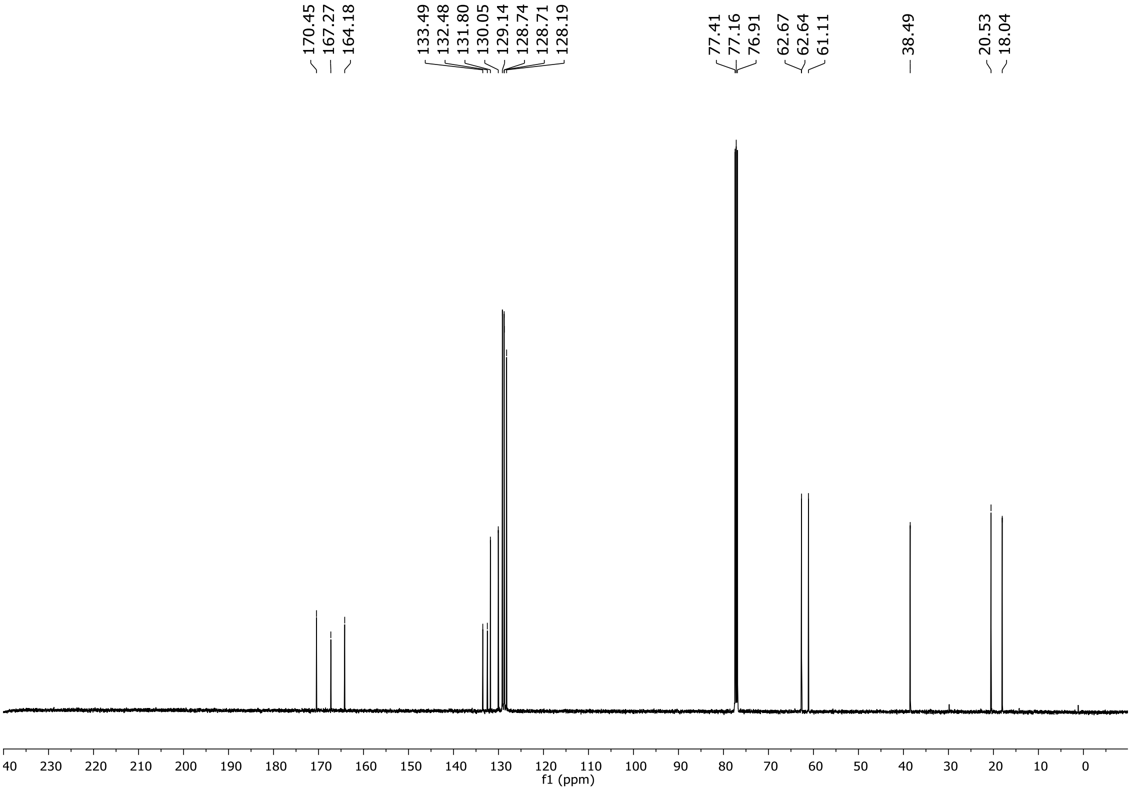

1r,4R)-*N*-((*R*)-1-(((diphenylmethylene)amino)oxy)-1-oxo-3-phenylpropan-2-yl)-4-isopropylcyclohexane-1-carboxamide (1ab)

^{1}H NMR (500 MHz, $CDCl_3$)

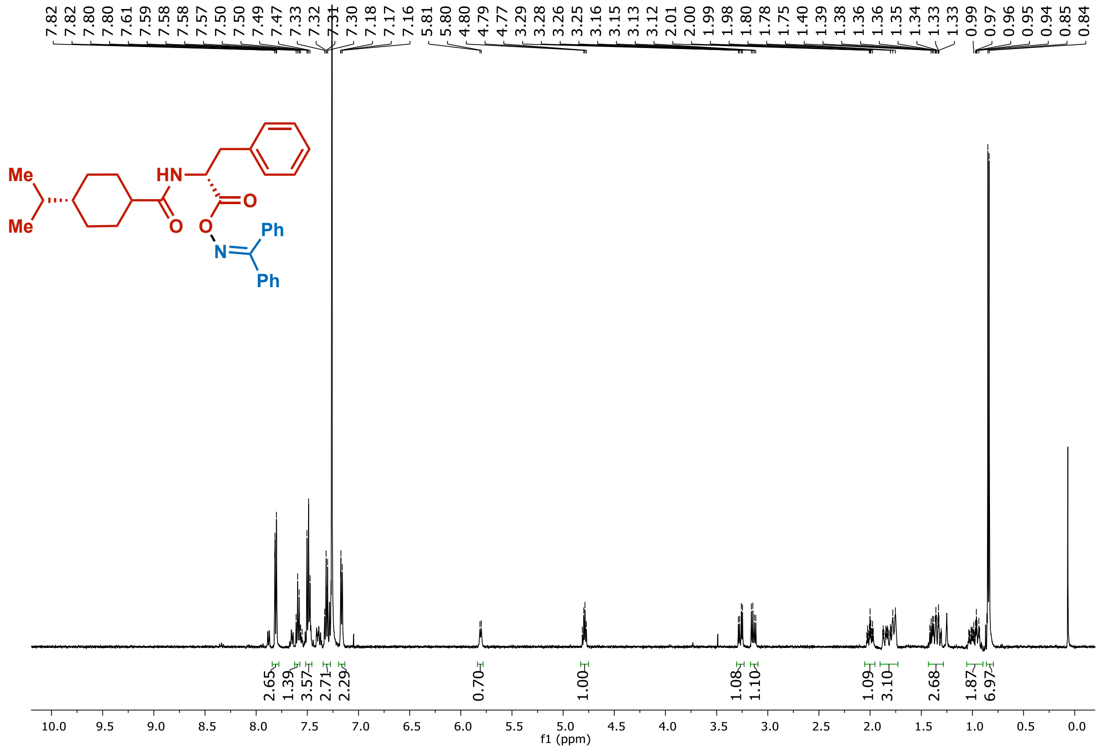

N-(2-(((diphenylmethylene)amino)oxy)-2-oxoethyl)-4-methylbenzenesulfonamide (1ac)

^{1}H NMR (500 MHz, $CDCl_3$)

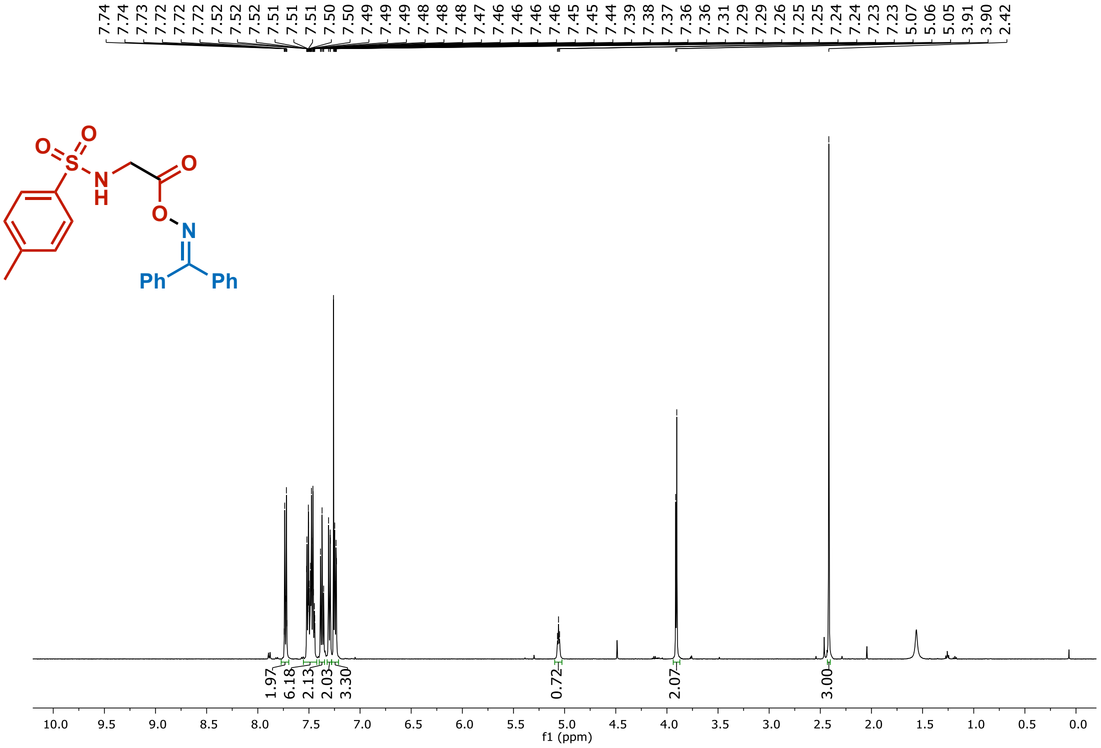

^{13}C NMR (126 MHz, $CDCl_3$)

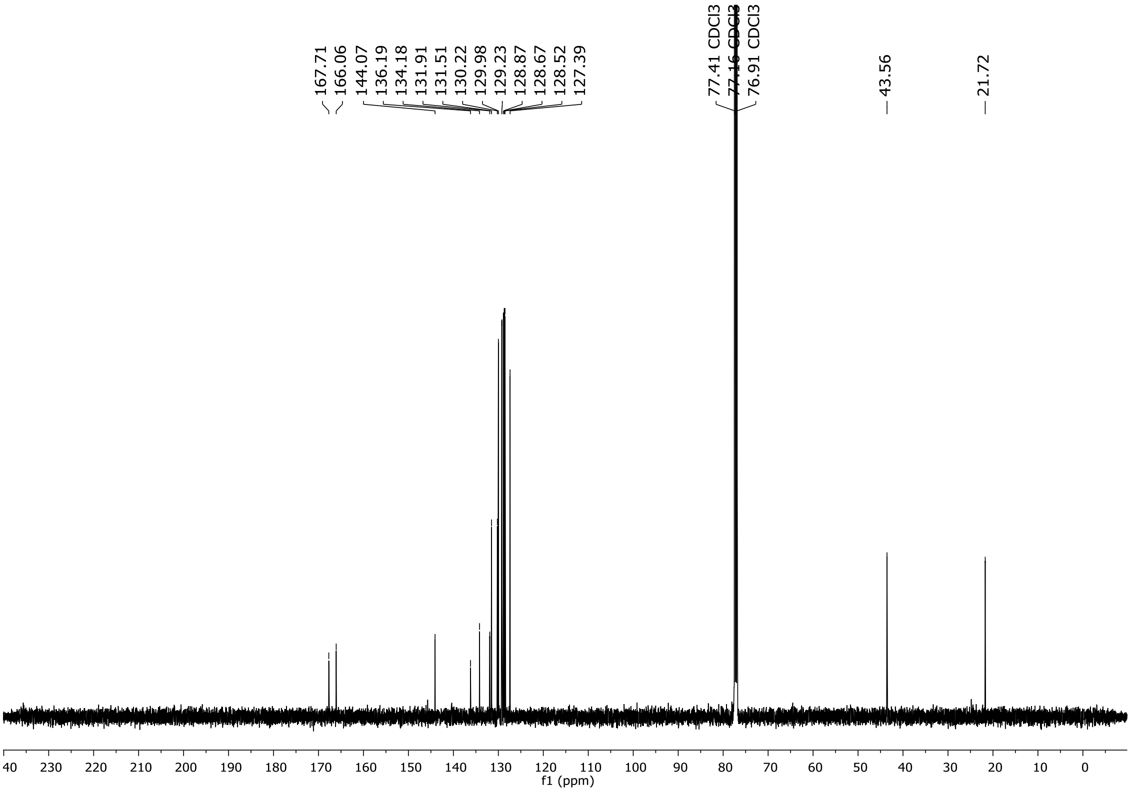

Diphenylmethanone *O*-(2,2,5,5-tetramethyltetrahydro-7H-[1,3]dioxolo[4',5':4,5]furo[3,2-d][1,3]dioxine-8a-carbonyl) oxime (1ad)

^{1}H NMR (500 MHz, CDCl$_3$)

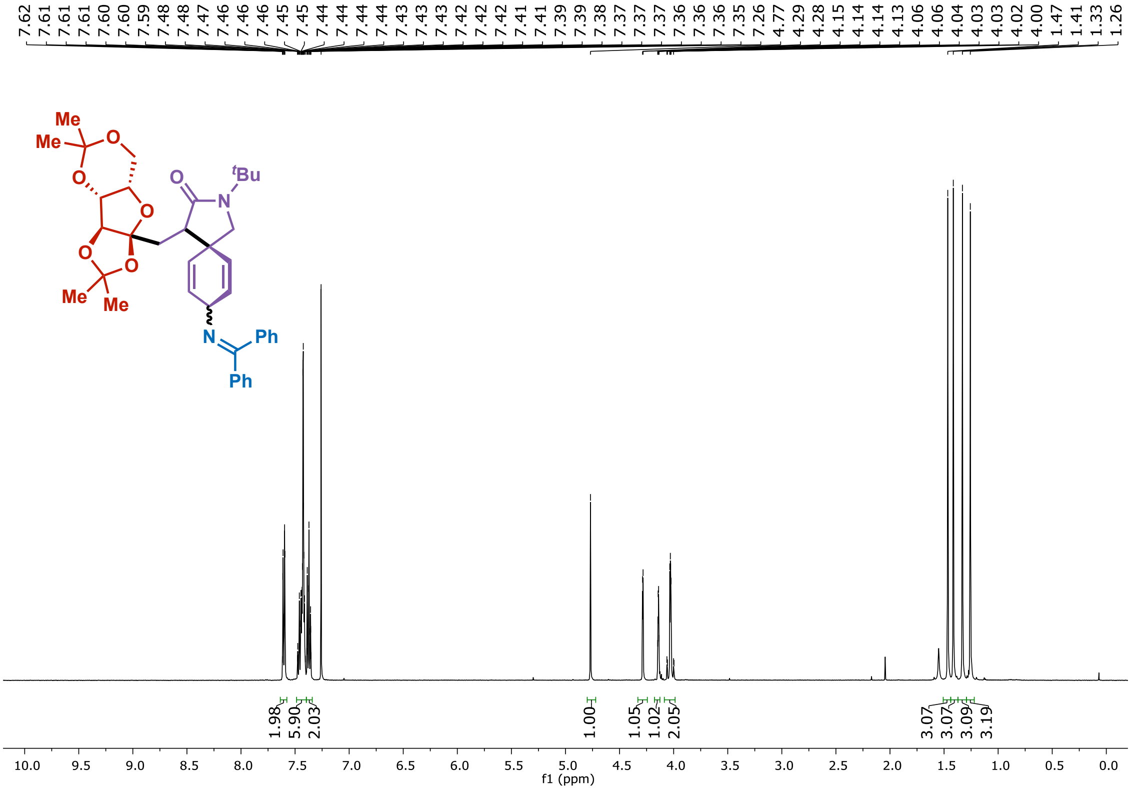

^{13}C NMR (126 MHz, CDCl$_3$)

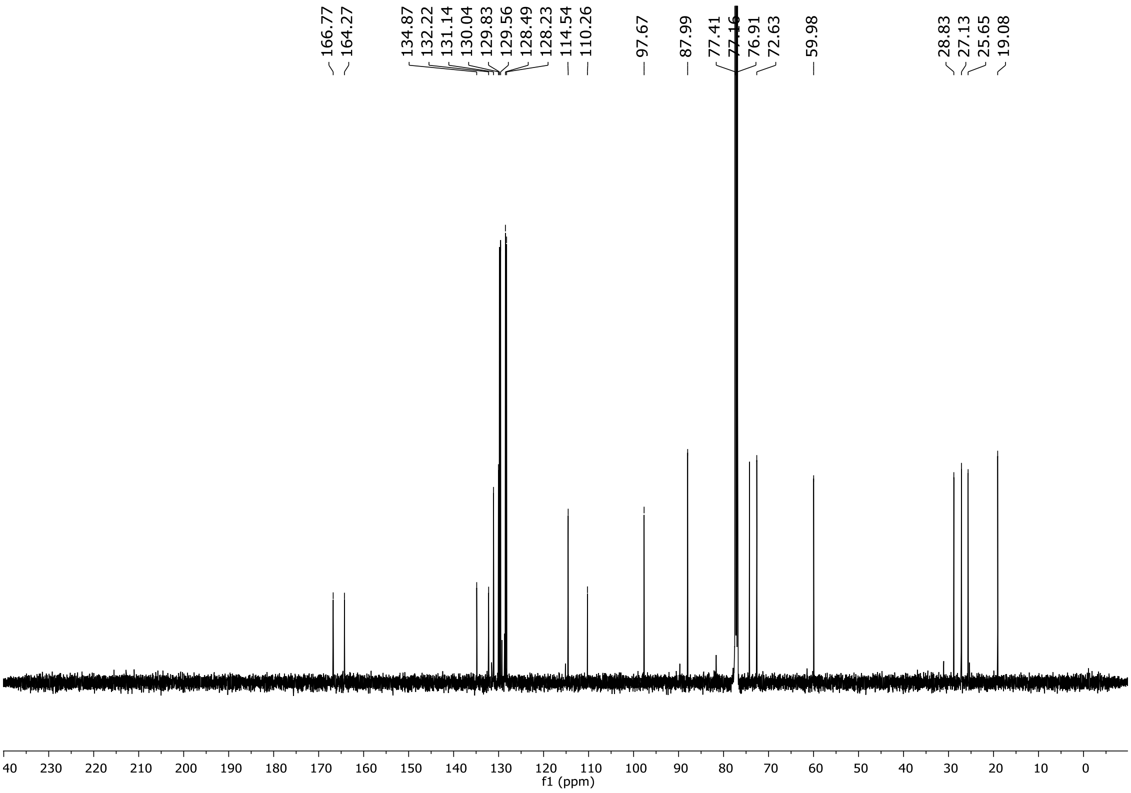

***N*-benzyl-*N*-(*tert*-butyl)-3-(((diphenylmethylene)amino)oxy)-3-oxopropanamide (1ae)**

^{1}H NMR (500 MHz, $CDCl_3$)

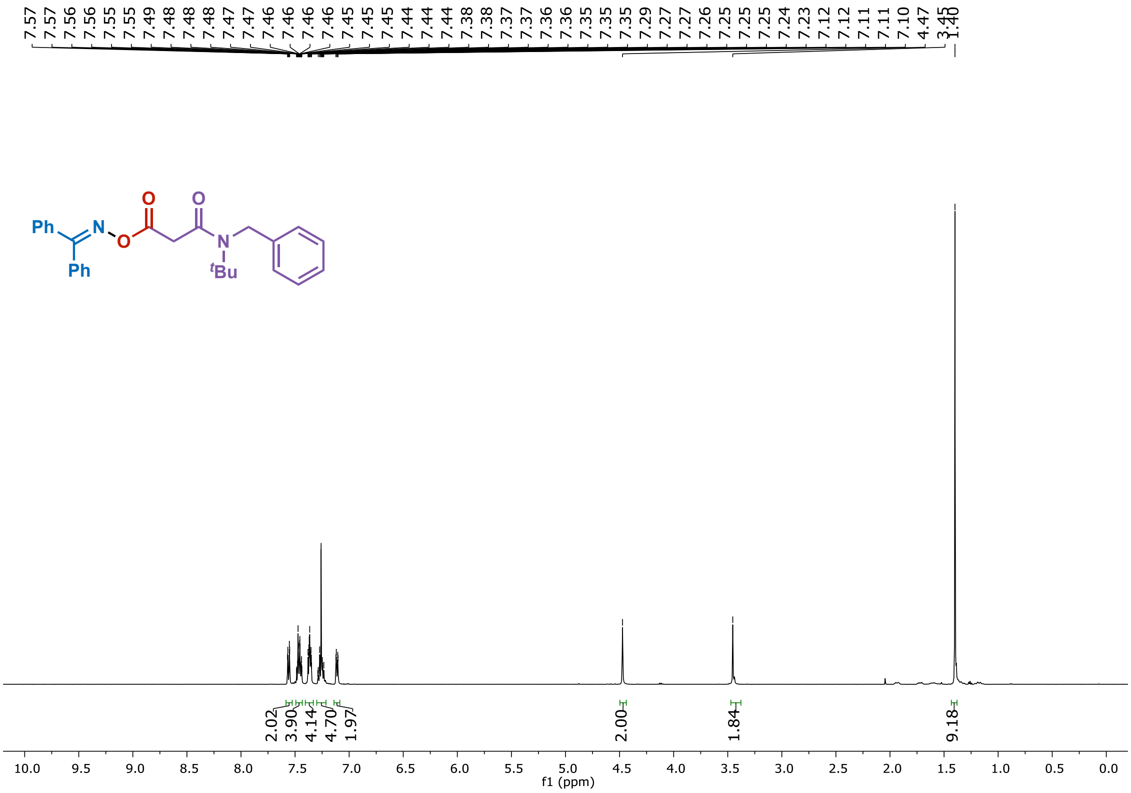

^{13}C NMR (126 MHz, $CDCl_3$)

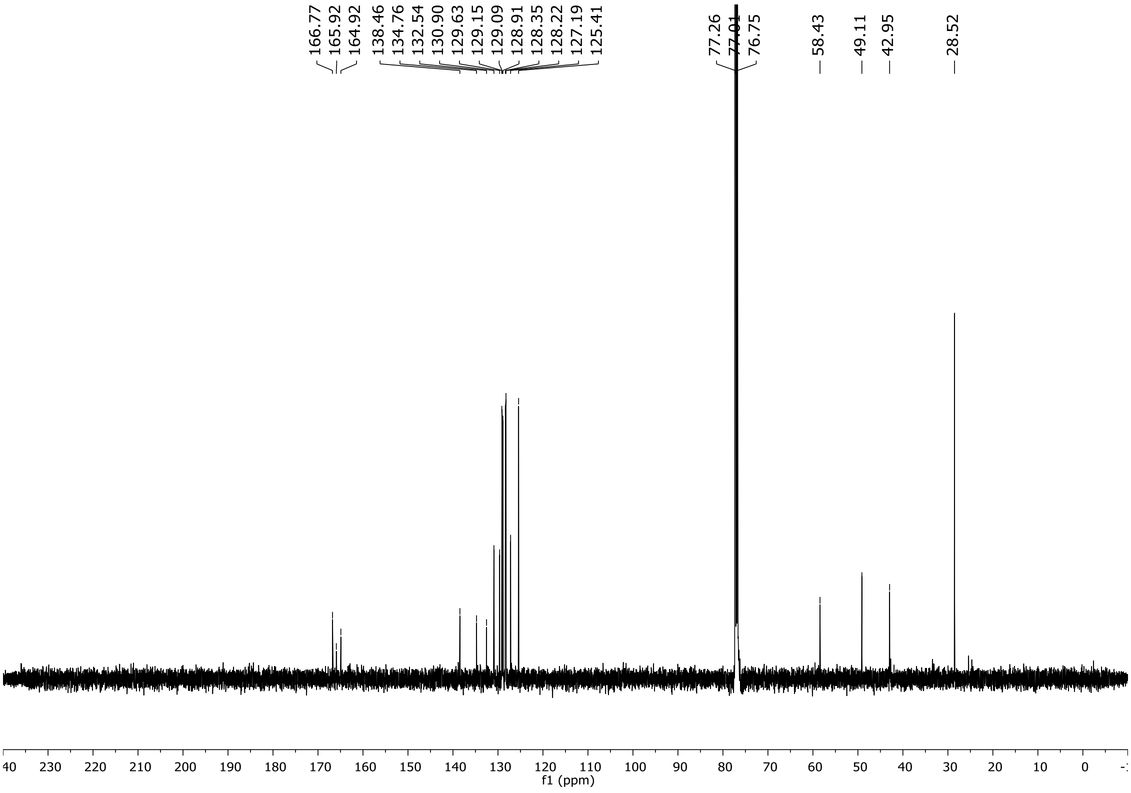

_N_-(_tert_-butyl)-N-(3, 5-dimethylbenzyl)acrylamide (2c)

^{1}H NMR (500 MHz, $CDCl_3$)

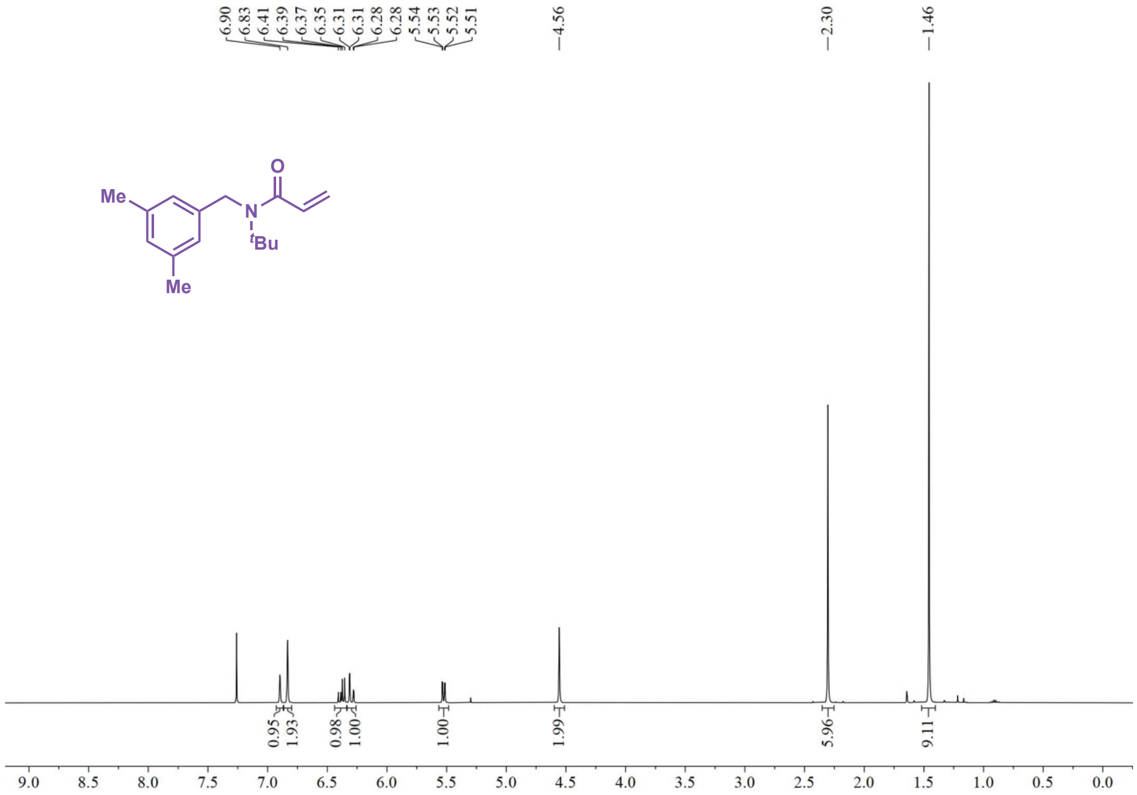

^{13}C NMR (126 MHz, $CDCl_3$)

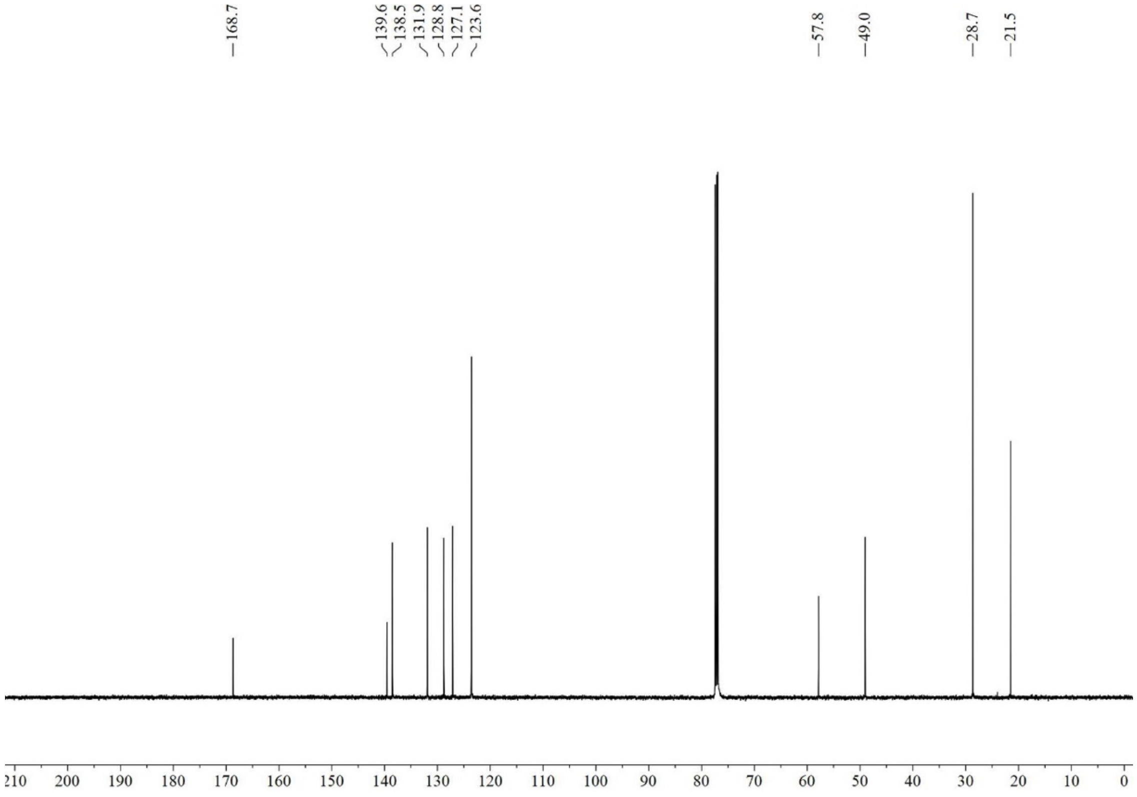

***N*-(*tert*-butyl)-*N*-(3,5-dichlorobenzyl)acrylamide (2d)**

^{1}H NMR (500 MHz, $CDCl_3$)

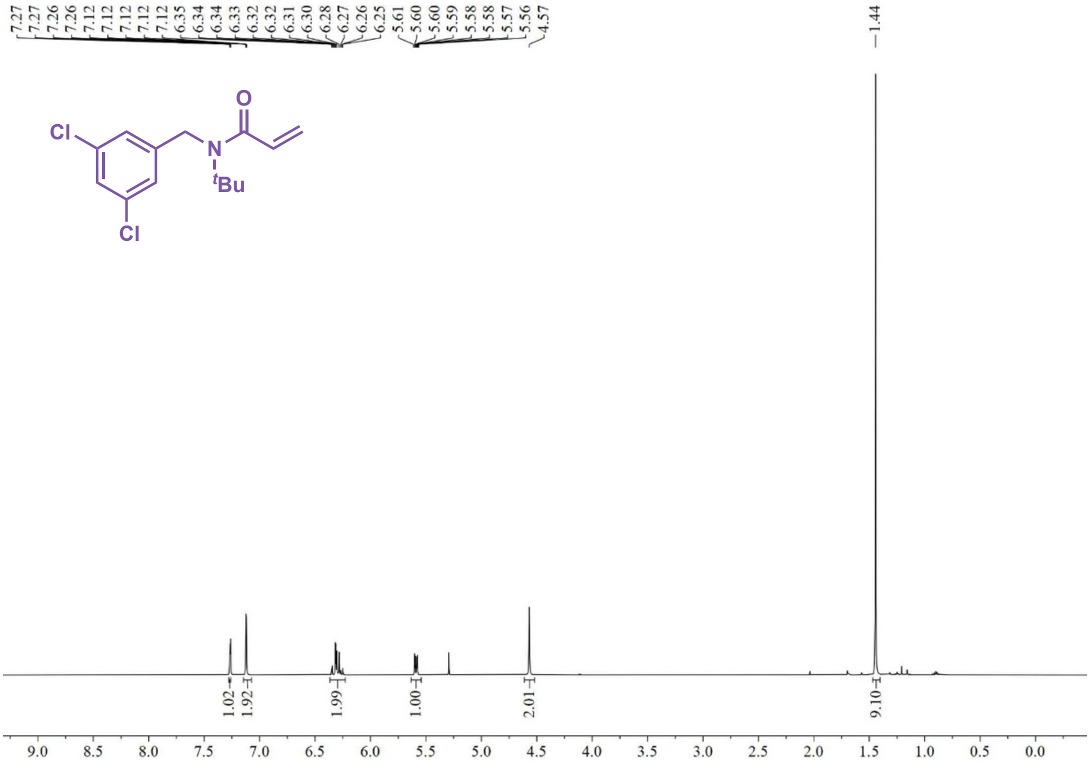

^{13}C NMR (126 MHz, $CDCl_3$)

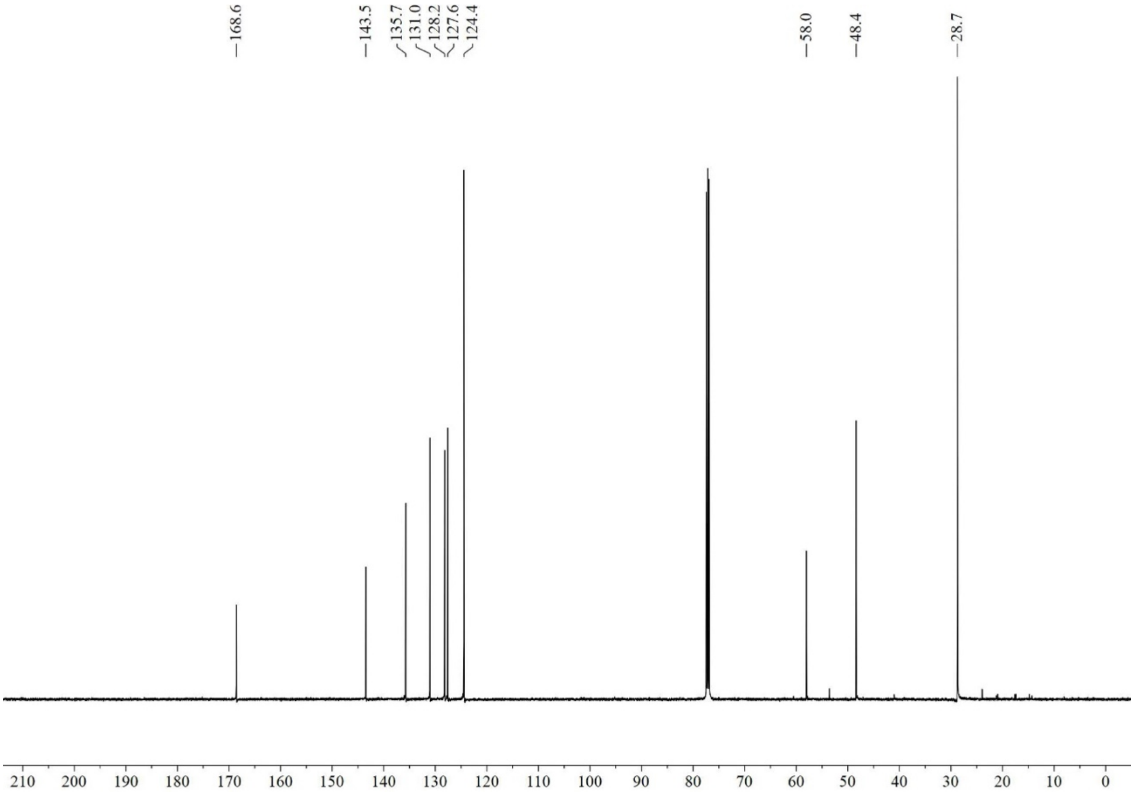

***N*-acryloyl-*N*-(*tert*-butyl)benzamide (2f)**

^{1}H NMR (500 MHz, $CDCl_3$)

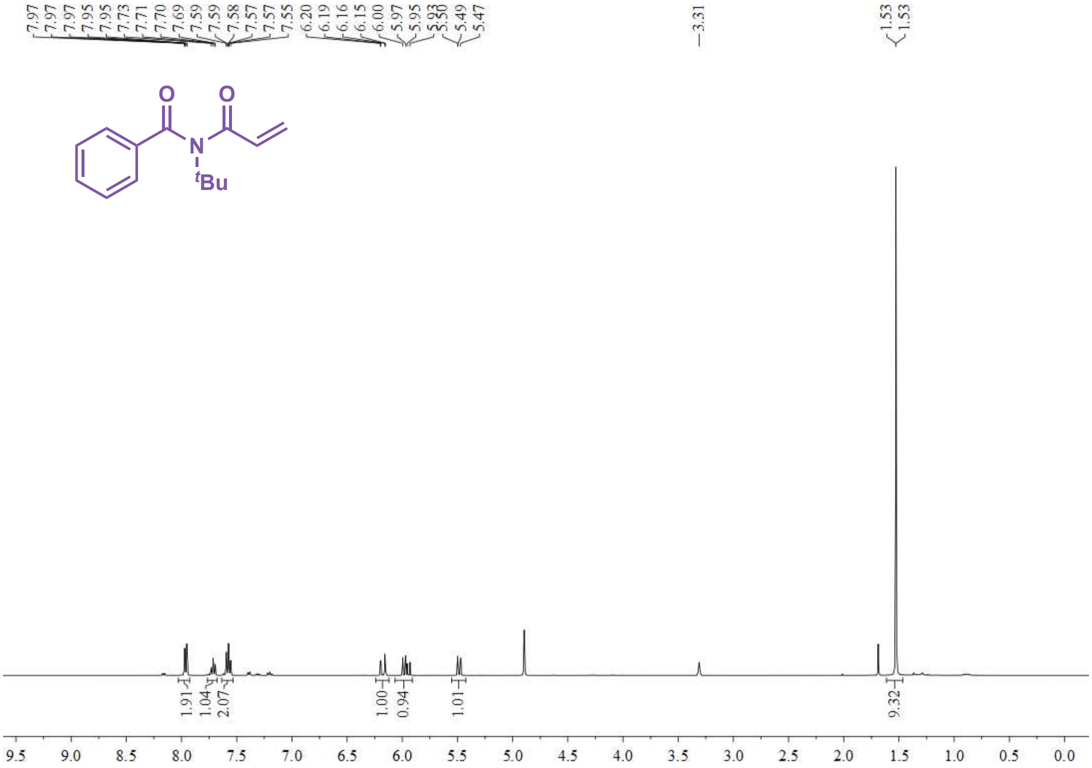

^{13}C NMR (126 MHz, $CDCl_3$)

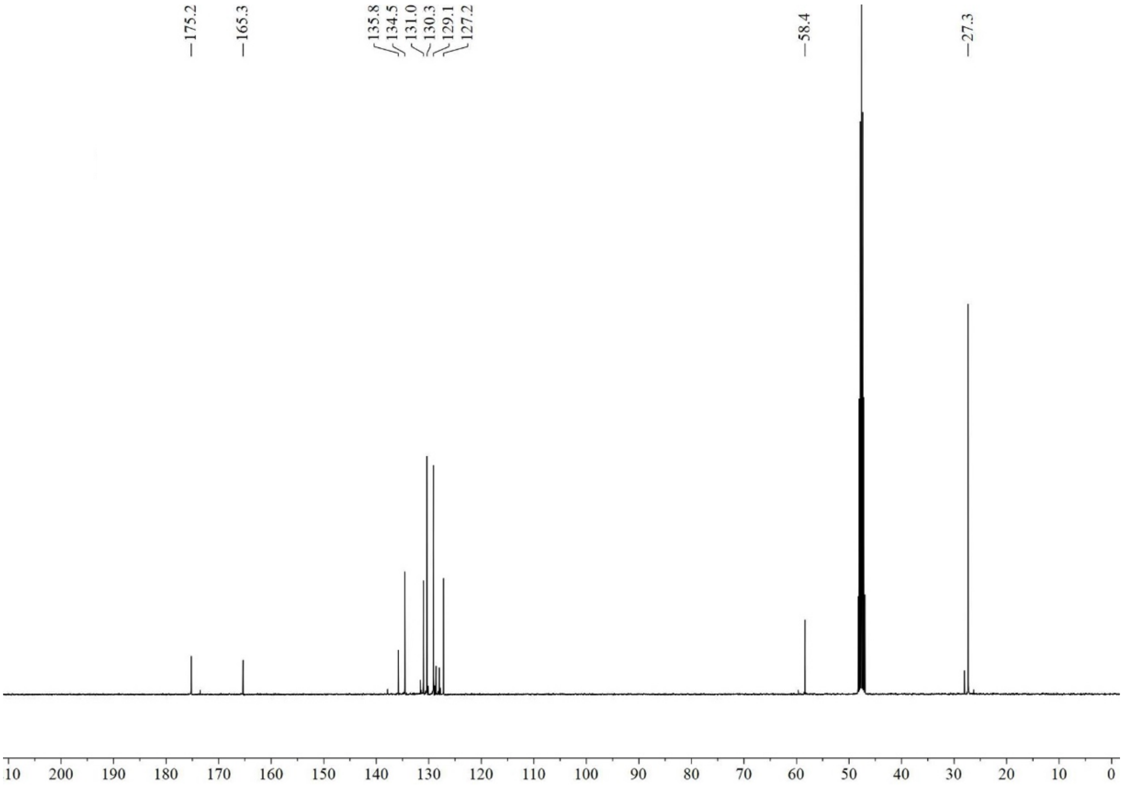

***N*-acryloyl-*N*-isopropylbenzamide (2g)**

^{1}H NMR (400 MHz, $CDCl_3$)

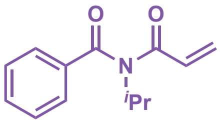

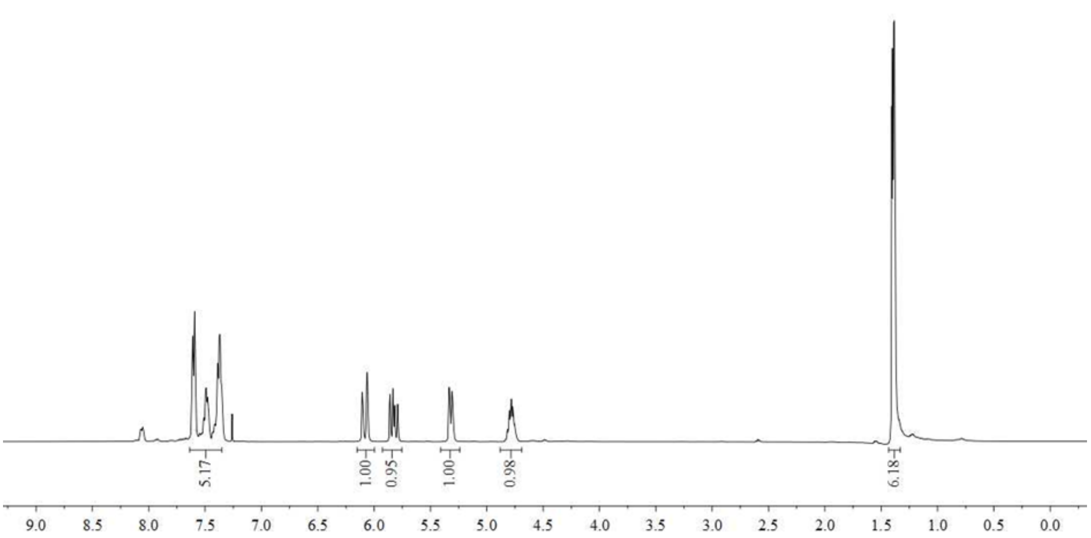

^{13}C NMR (101 MHz, $CDCl_3$)

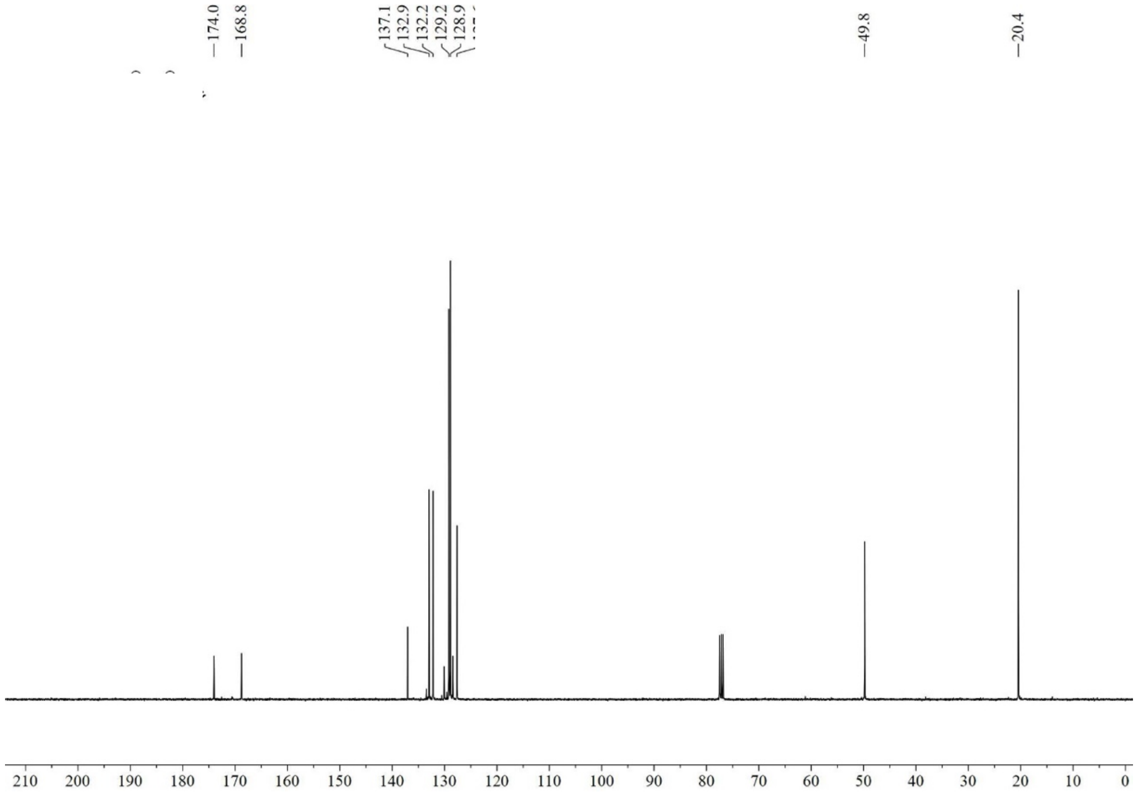

***N*-(*tert*-butyl)-*N*-(thiophen-2-ylmethyl)acrylamide (2h)**

^{1}H NMR (500 MHz, $CDCl_3$)

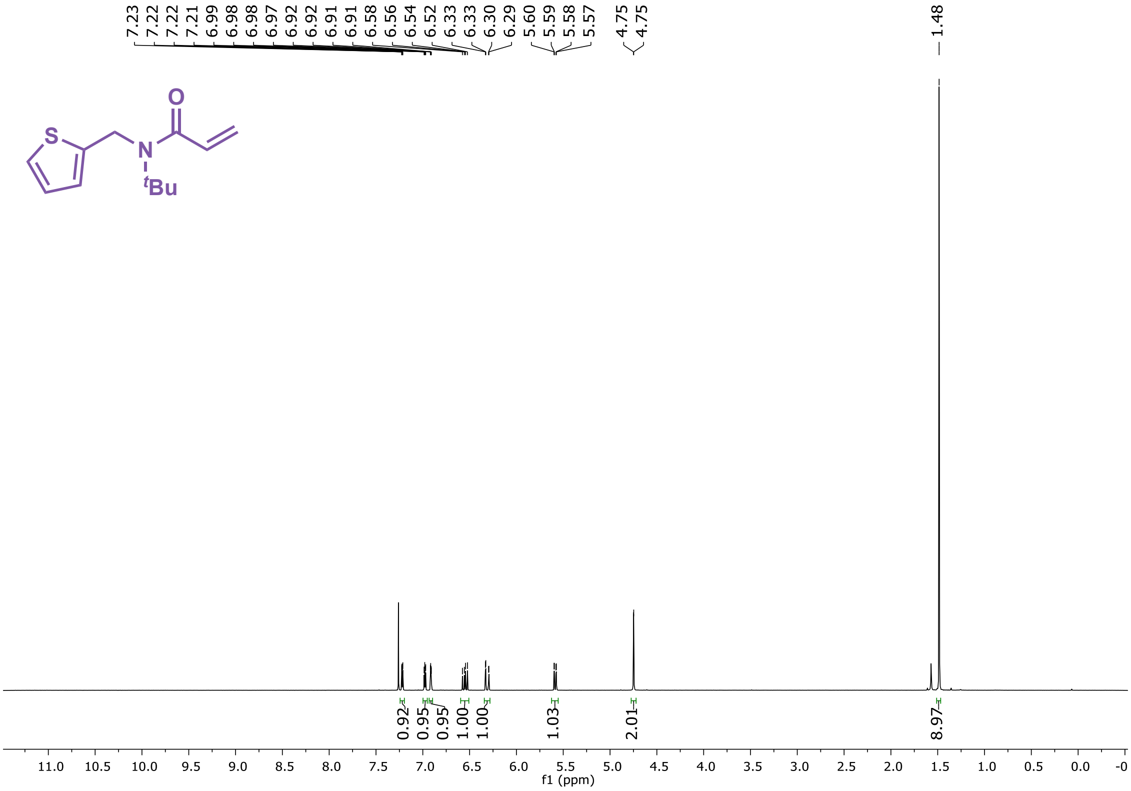

^{13}C NMR (126 MHz, $CDCl_3$)

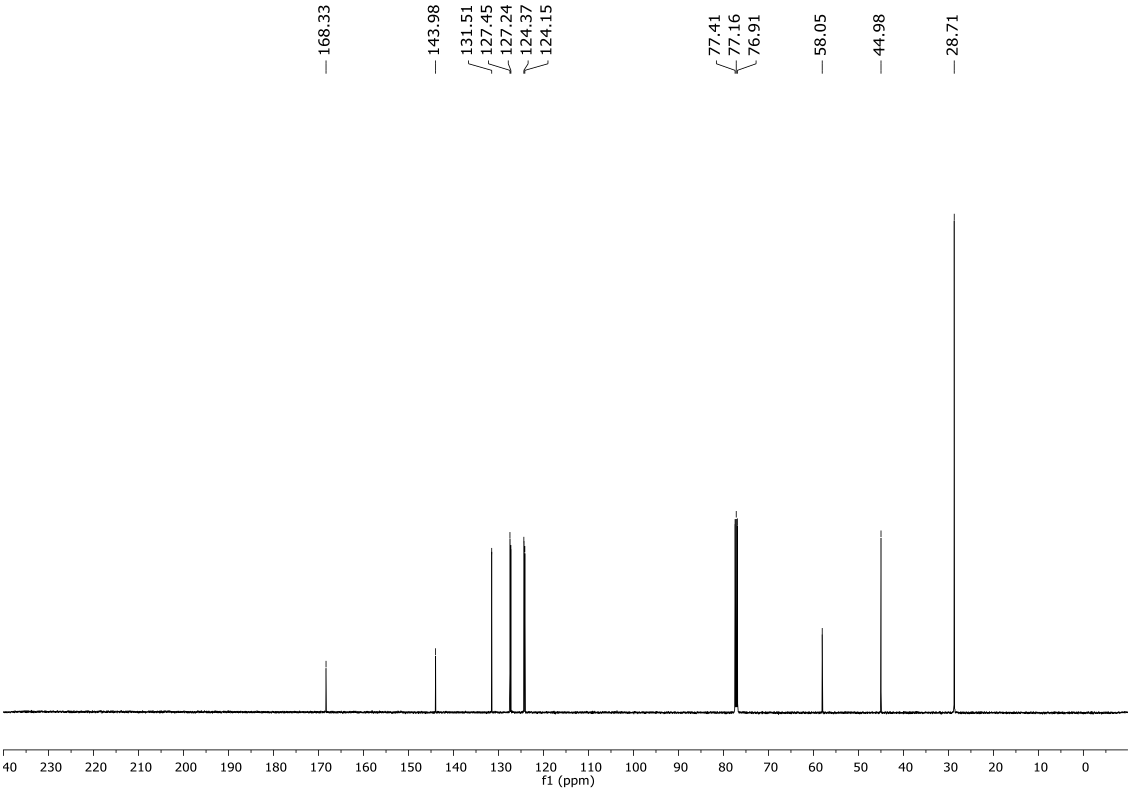

***N*-(*tert*-butyl)-*N*-(dibenzo[b,d]furan-4-ylmethyl)acrylamide (2i).**

^{1}H NMR (500 MHz, $CDCl_3$)

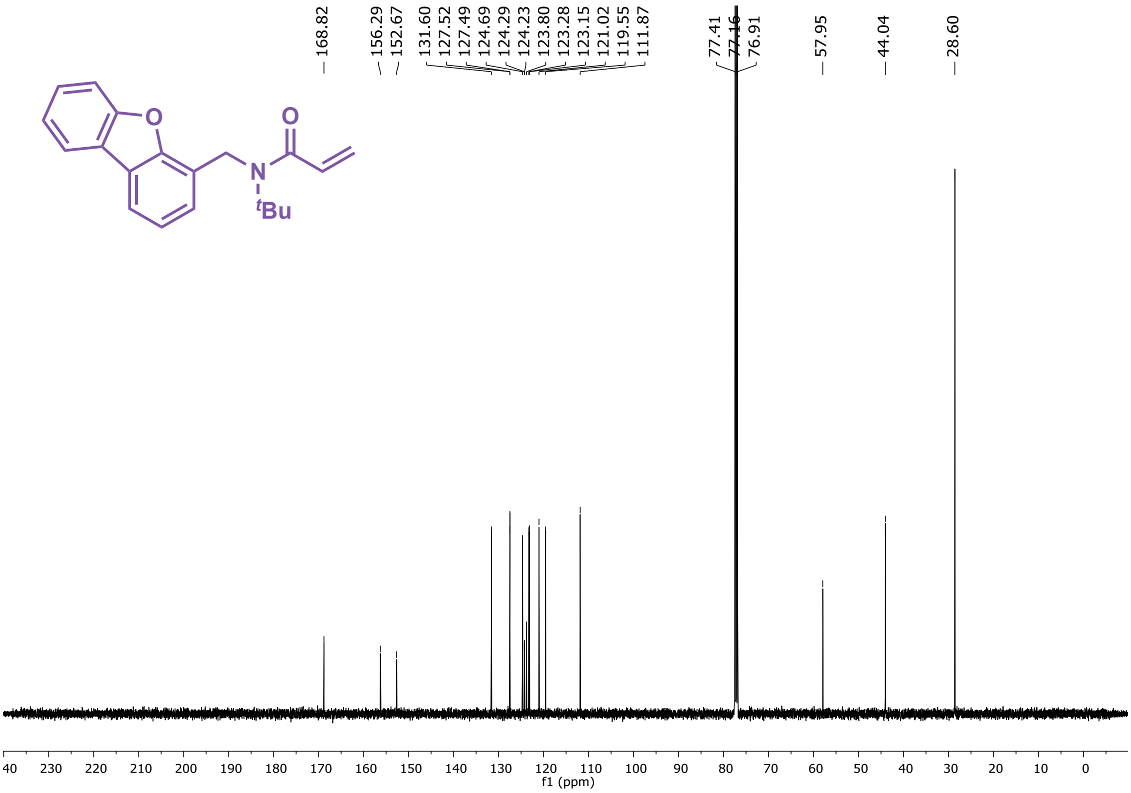

^{13}C NMR (126 MHz, $CDCl_3$)

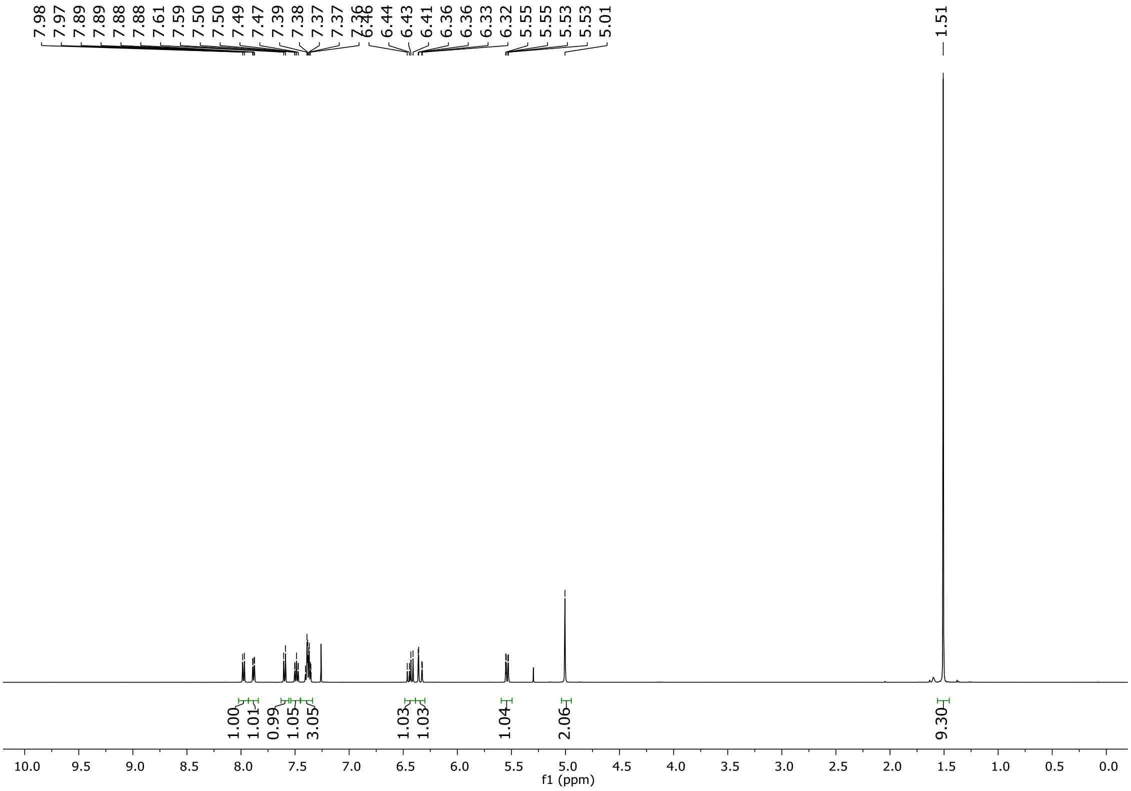

***N*-(*tert*-butyl)-*N*-(2-fluorobenzyl)acrylamide (2j).**

^{1}H NMR (500 MHz, $CDCl_3$)

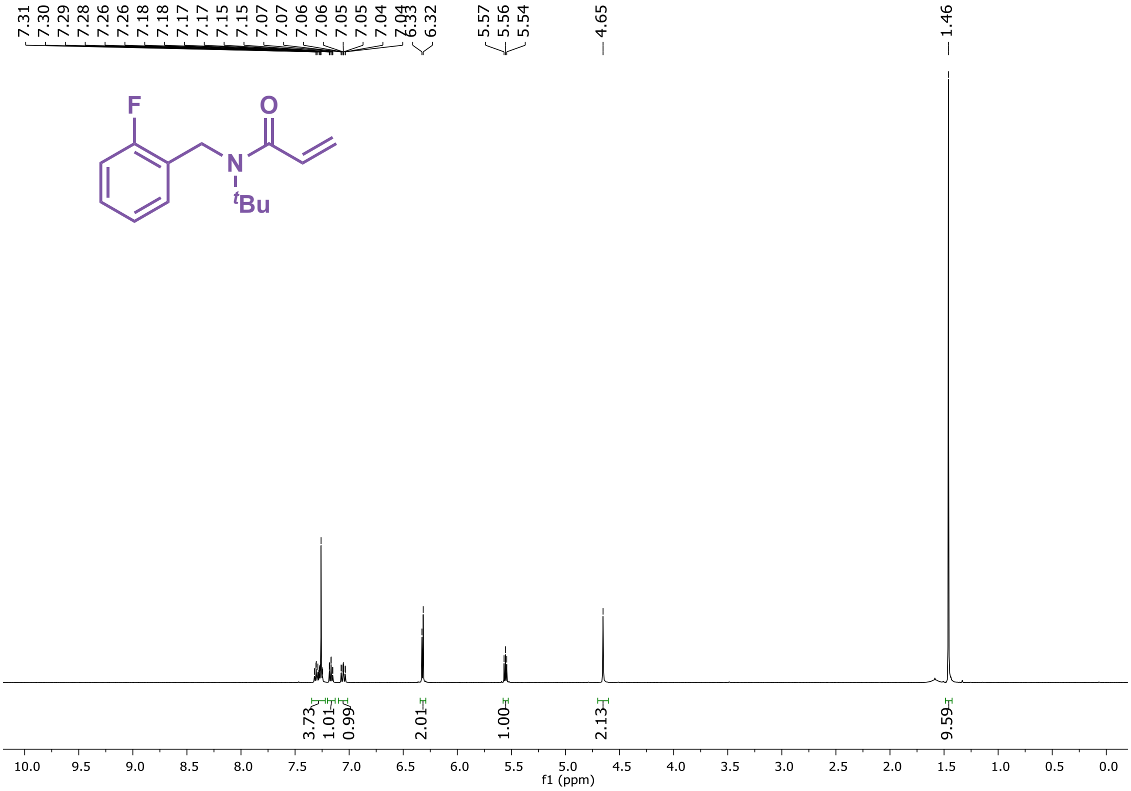

^{13}C NMR (126 MHz, $CDCl_3$)

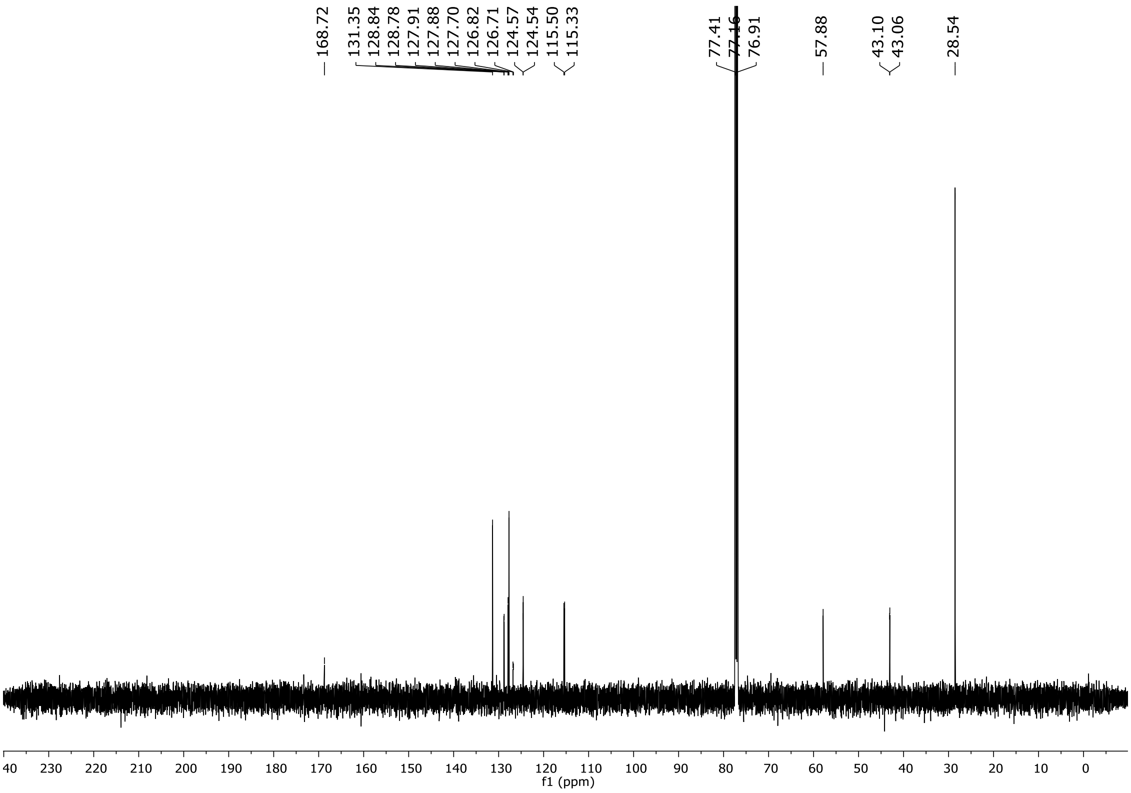

^{19}F NMR (377 MHz, $CDCl_3$)

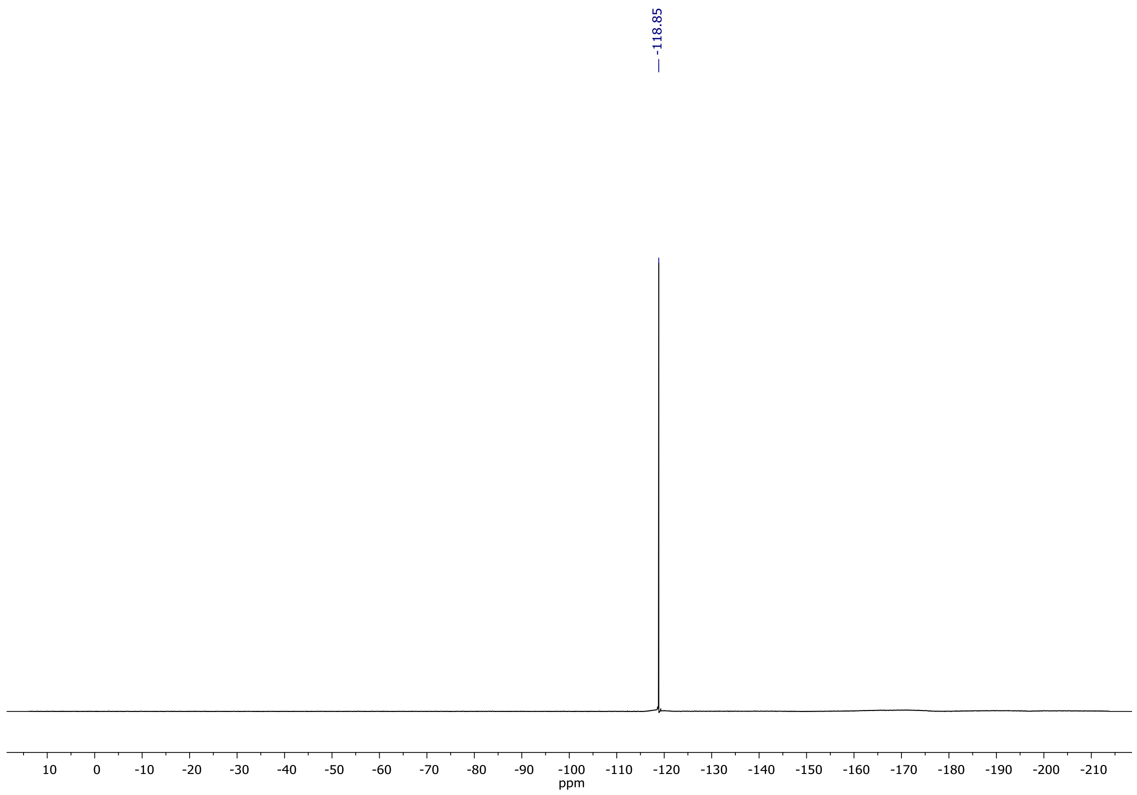

***N*-([1,1'-biphenyl]-4-ylmethyl)-*N*-(*tert*-butyl)acrylamide (2k).**

^{1}H NMR (500 MHz, CDCl$_3$)

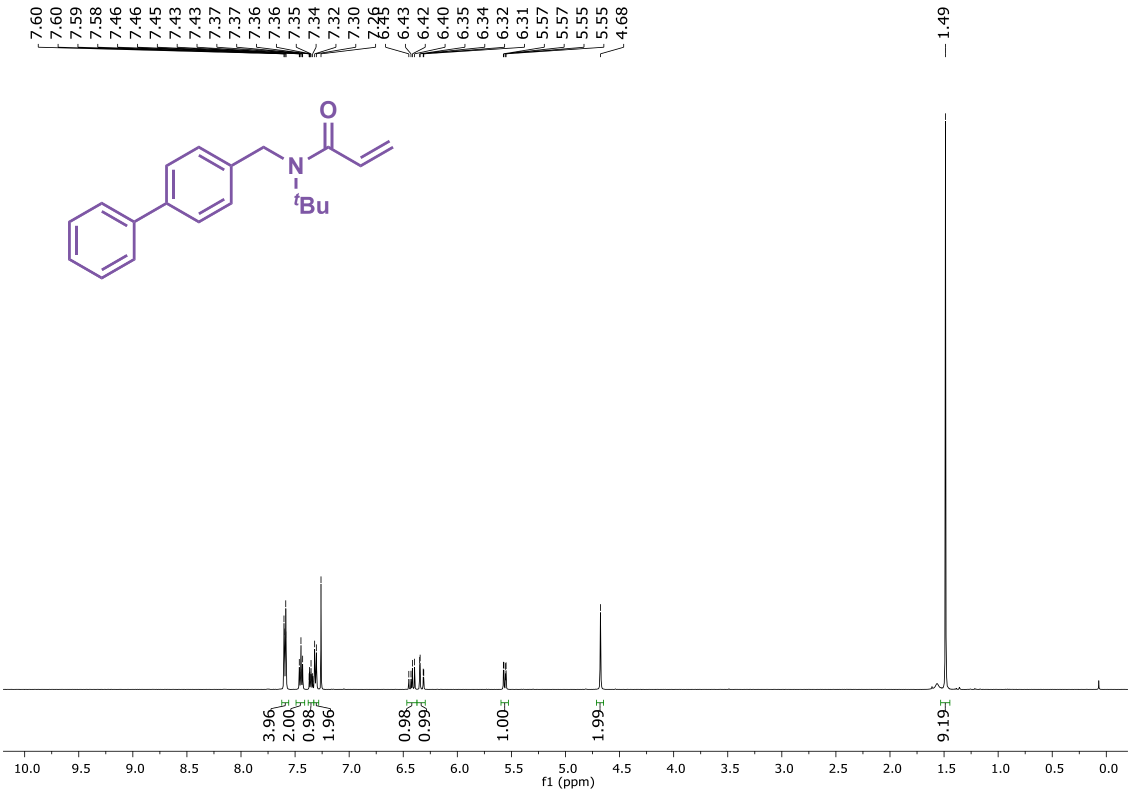

^{13}C NMR (126 MHz, CDCl$_3$)

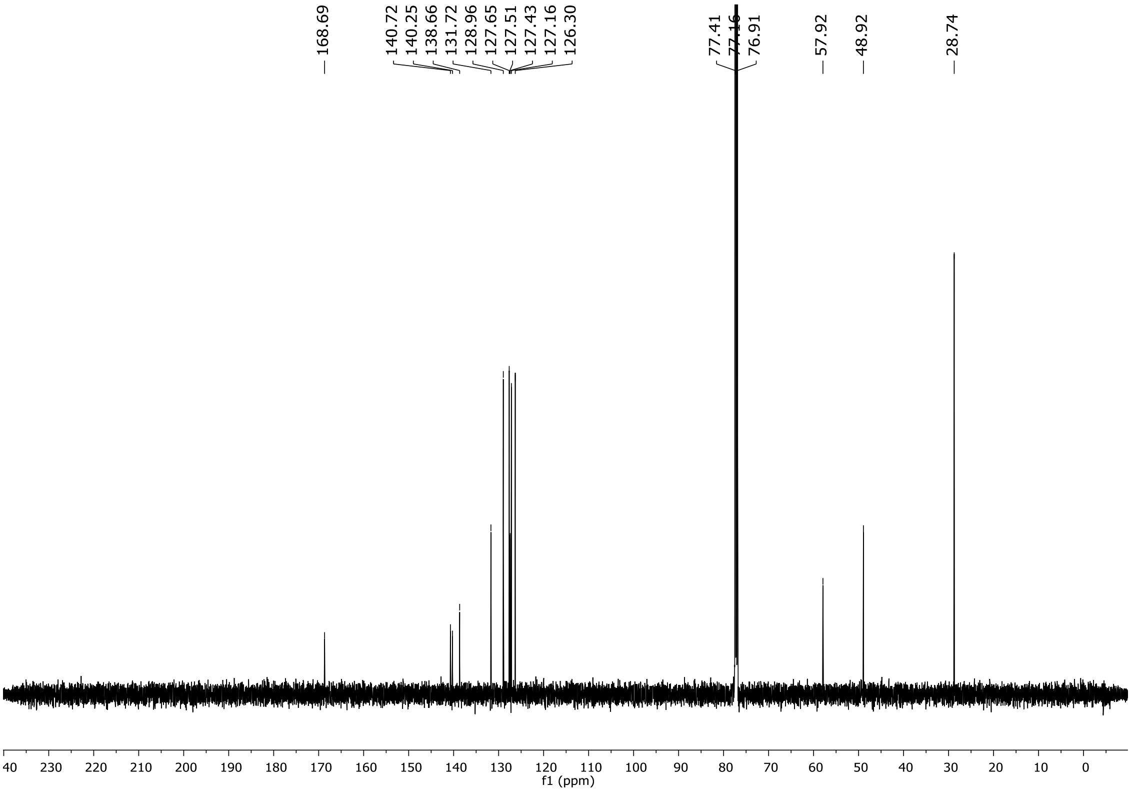

***N*-benzyl-*N*-(tert-butyl)bicyclo[1.1.0]butane-1-carboxamide (2m).**

^{1}H NMR (500 MHz, $CDCl_3$)

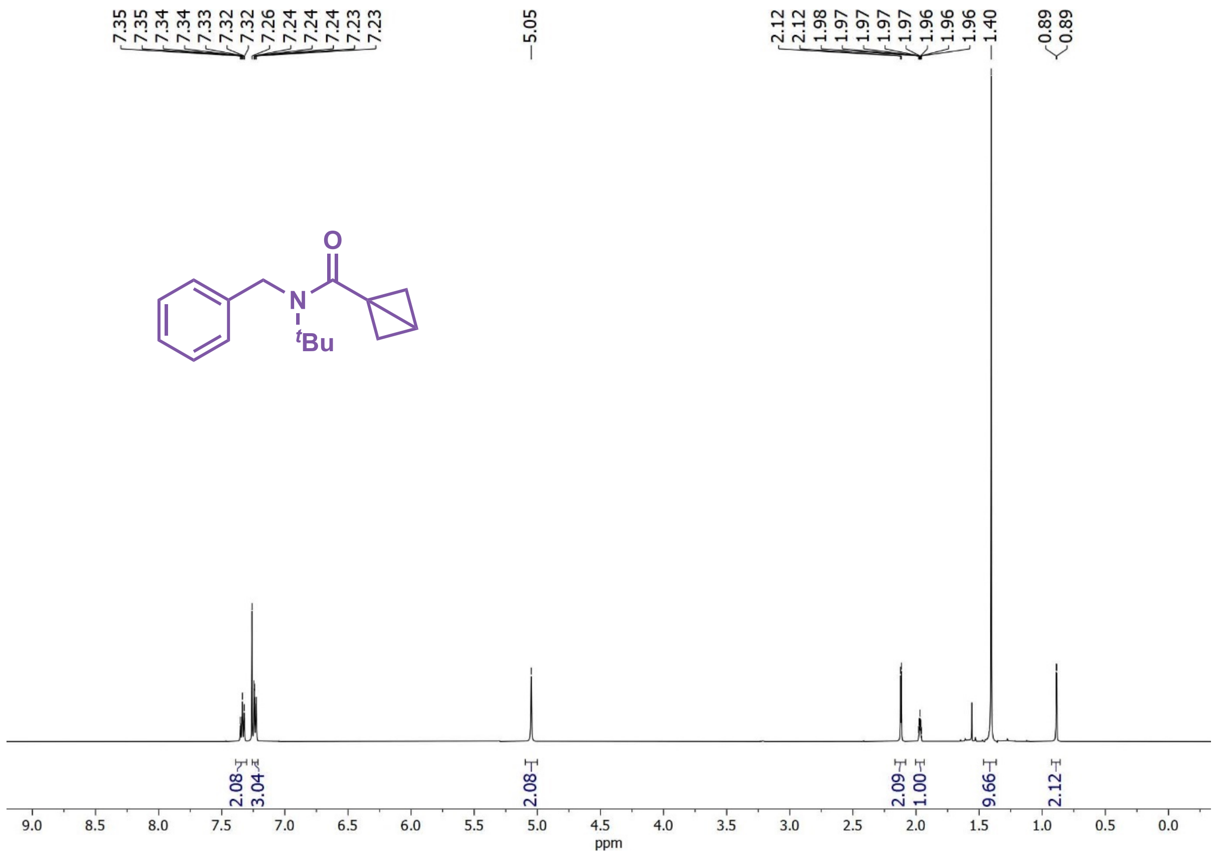

^{13}C NMR (101 MHz, $CDCl_3$)

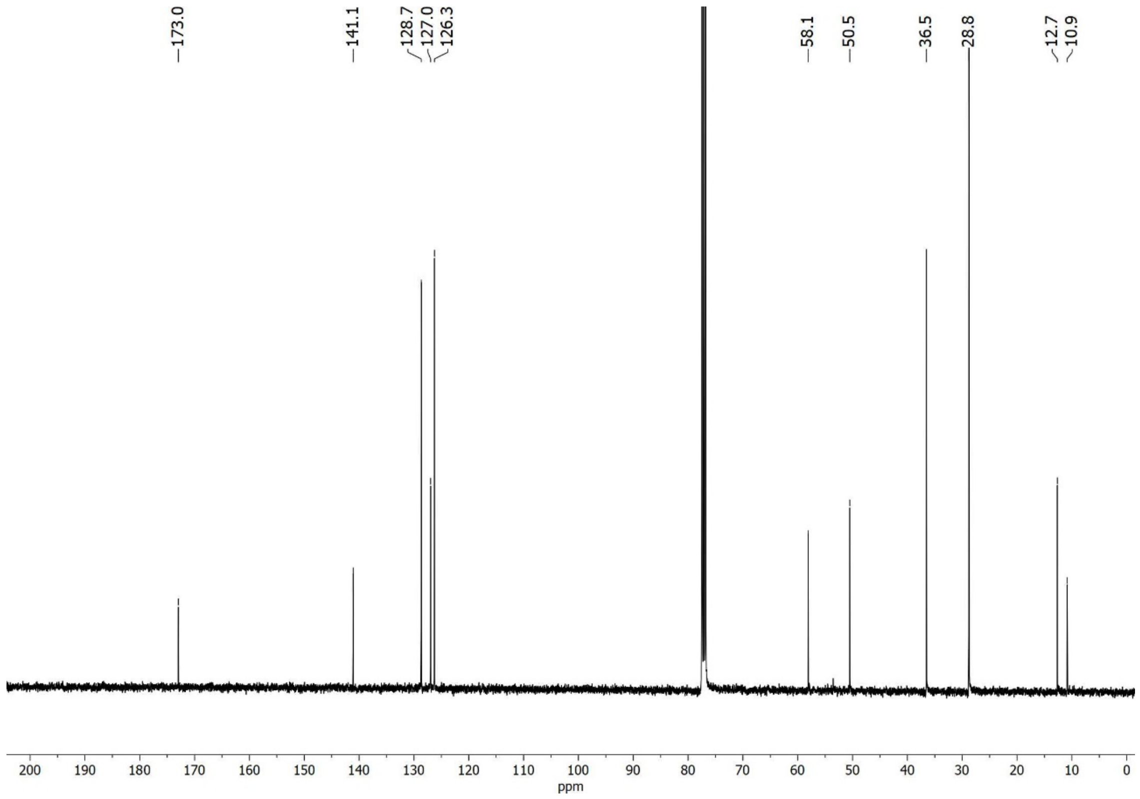

2-(*tert*-Butyl)-8-((diphenylmethylene)amino)-4-(4-phenylbutyl)-2-azaspiro[4.5]deca-6,9-dien-3-one (3a)

^{1}H NMR (400 MHz, CD_3OD)

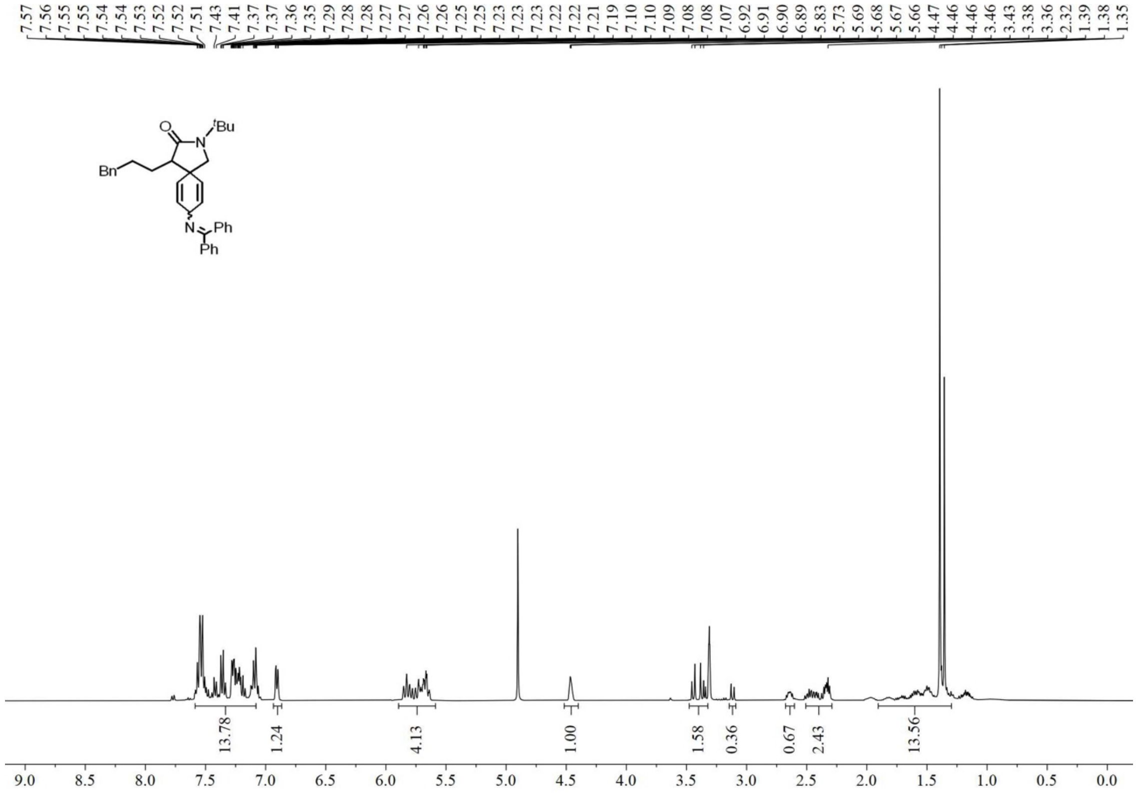

^{13}C NMR (101 MHz, CD_3OD)

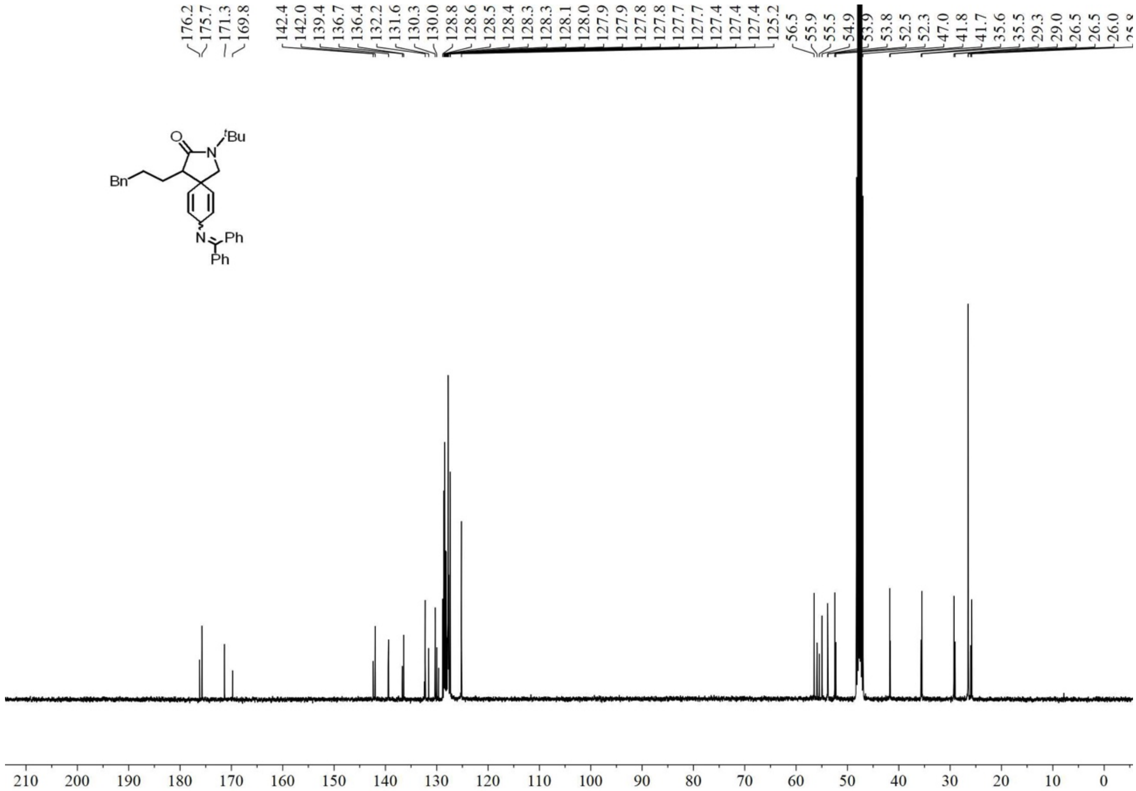

2-(*tert*-Butyl)-8-((diphenylmethylene)amino)-4-(4,4,4-trifluorobutyl)-2-azaspiro[4.5]deca-6,9-dien-3-one (3b)

^{1}H NMR (400 MHz, CD_3OD)

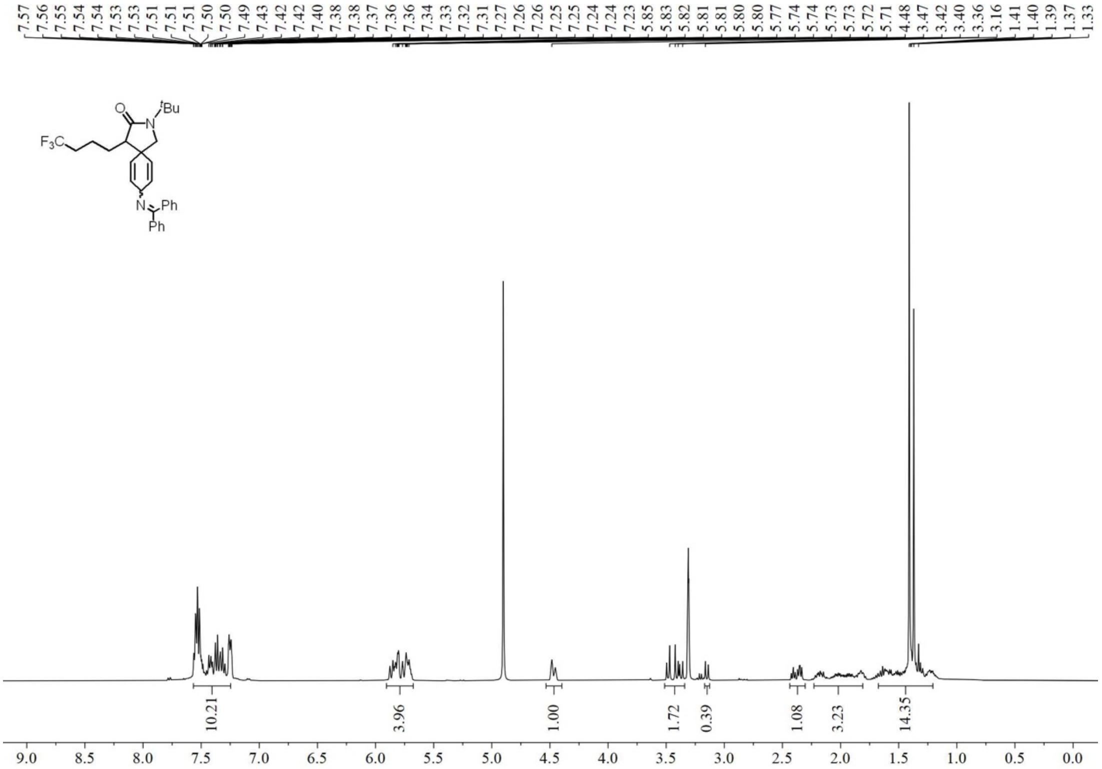

^{13}C NMR (101 MHz, CD_3OD)

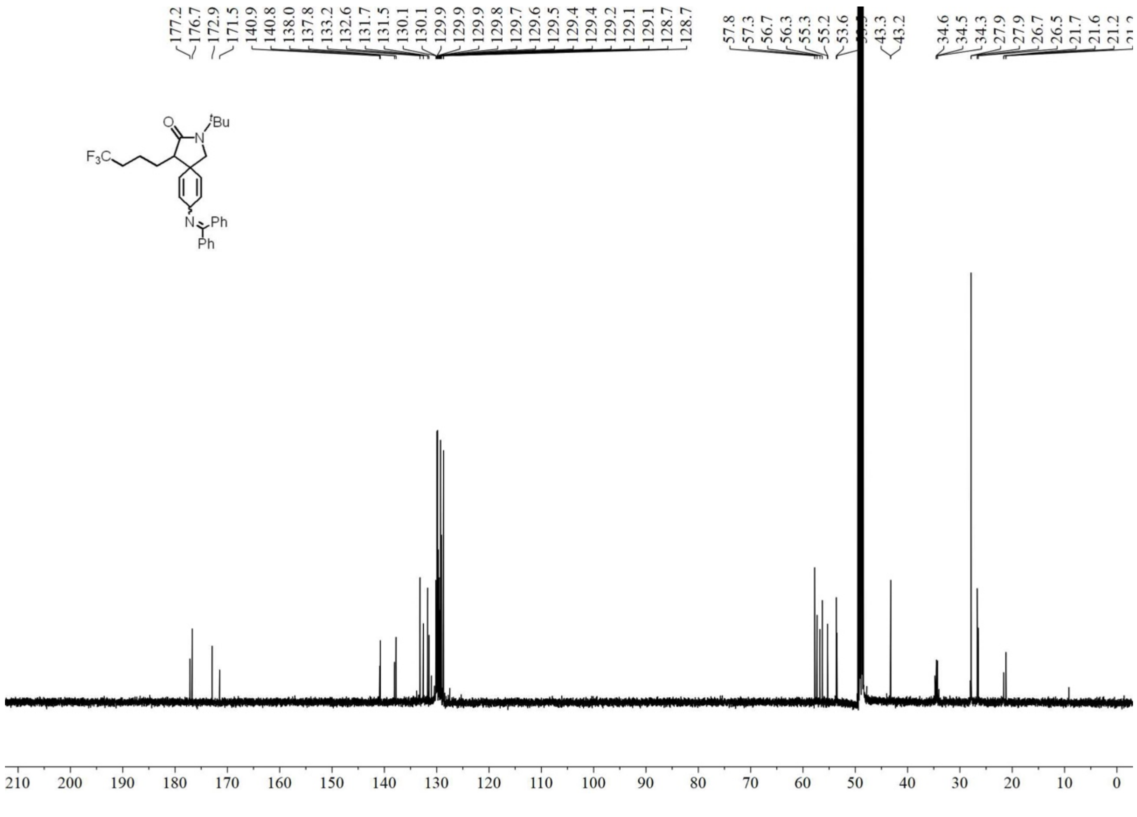

^{19}F NMR (377 MHz, CD_3OD)

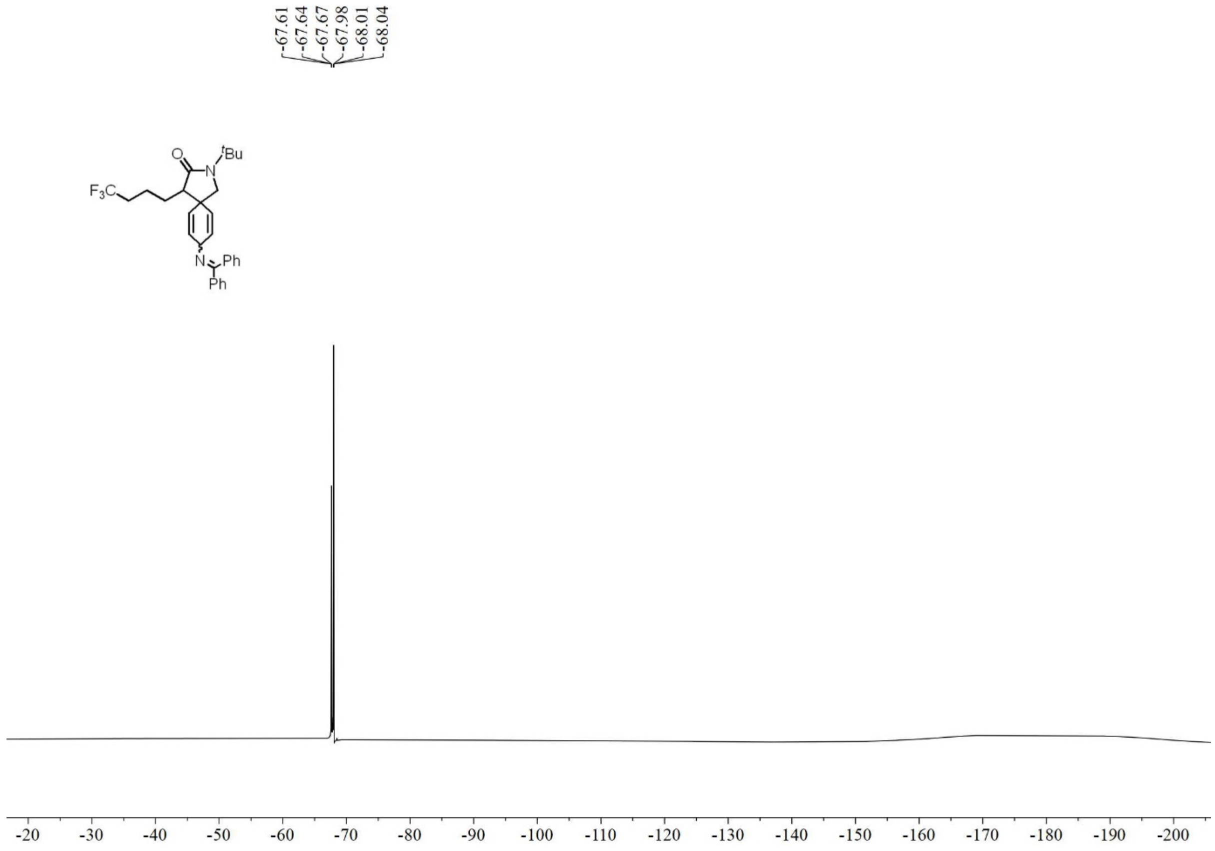

2-(*tert*-Butyl)-8-((diphenylmethylene)amino)-4-ethyl-2-azaspiro[4.5]deca-6,9-dien-3-one (3c)

^{1}H NMR (400 MHz, CD_3OD)

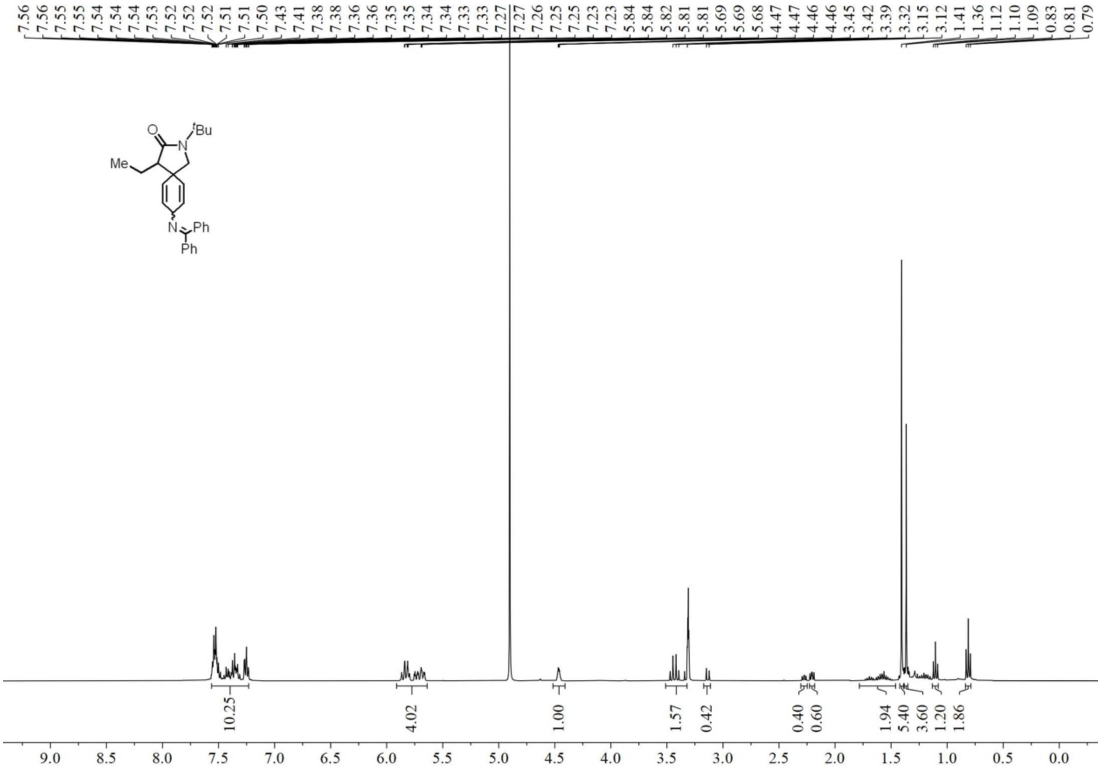

^{13}C NMR (126 MHz, CD_3OD)

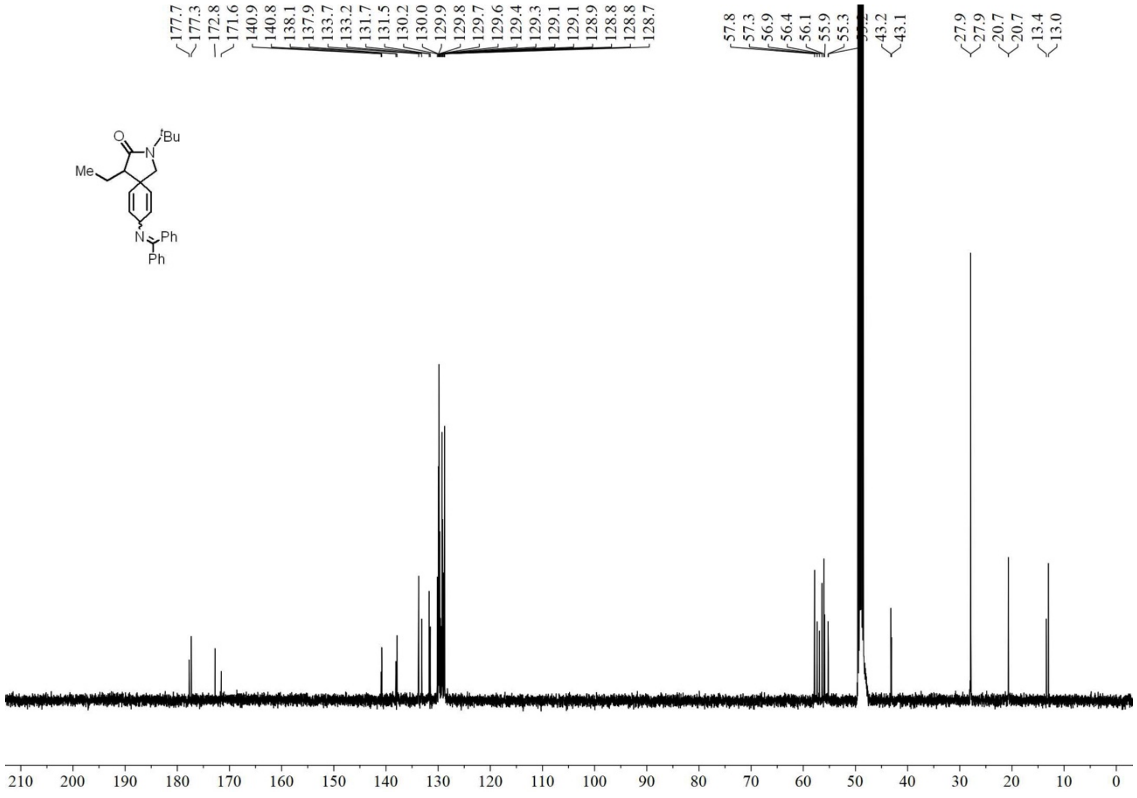

2-(*tert*-Butyl)-4-(cyclobutylmethyl)-8-((diphenylmethylene)amino)-2-azaspiro[4.5]deca-6,9-dien-3-one (3d)

^{1}H NMR (400 MHz, CD_3OD)

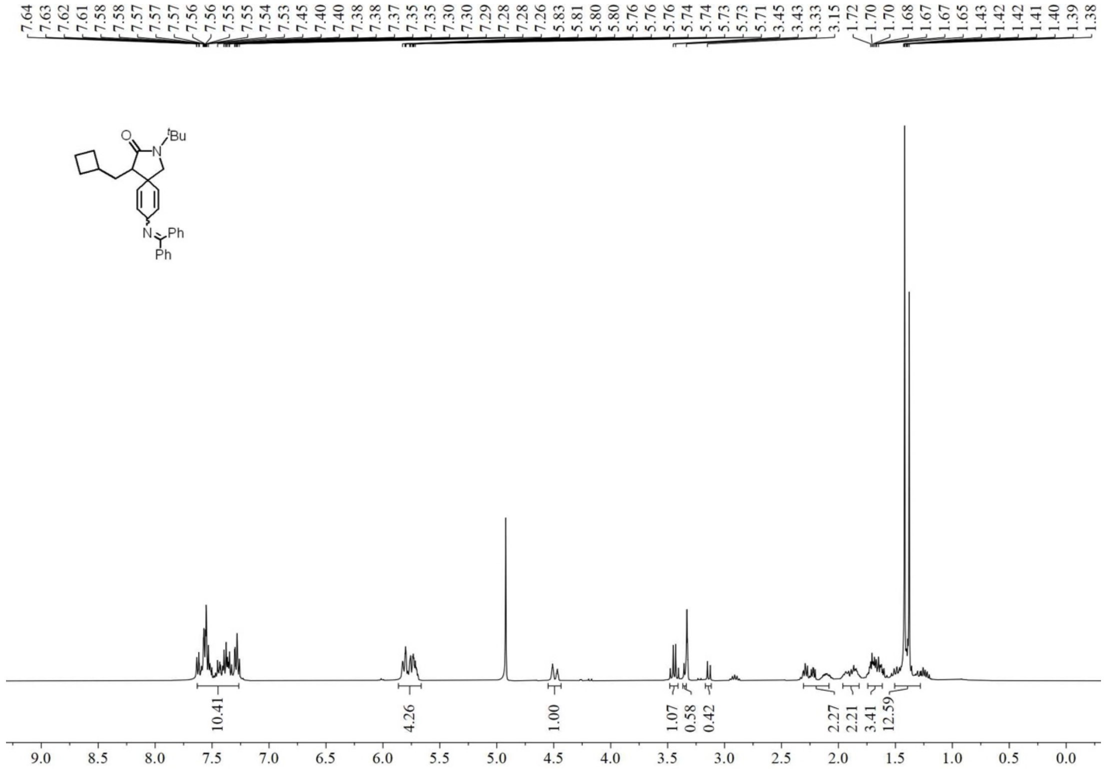

^{13}C NMR (101 MHz, CD_3OD)

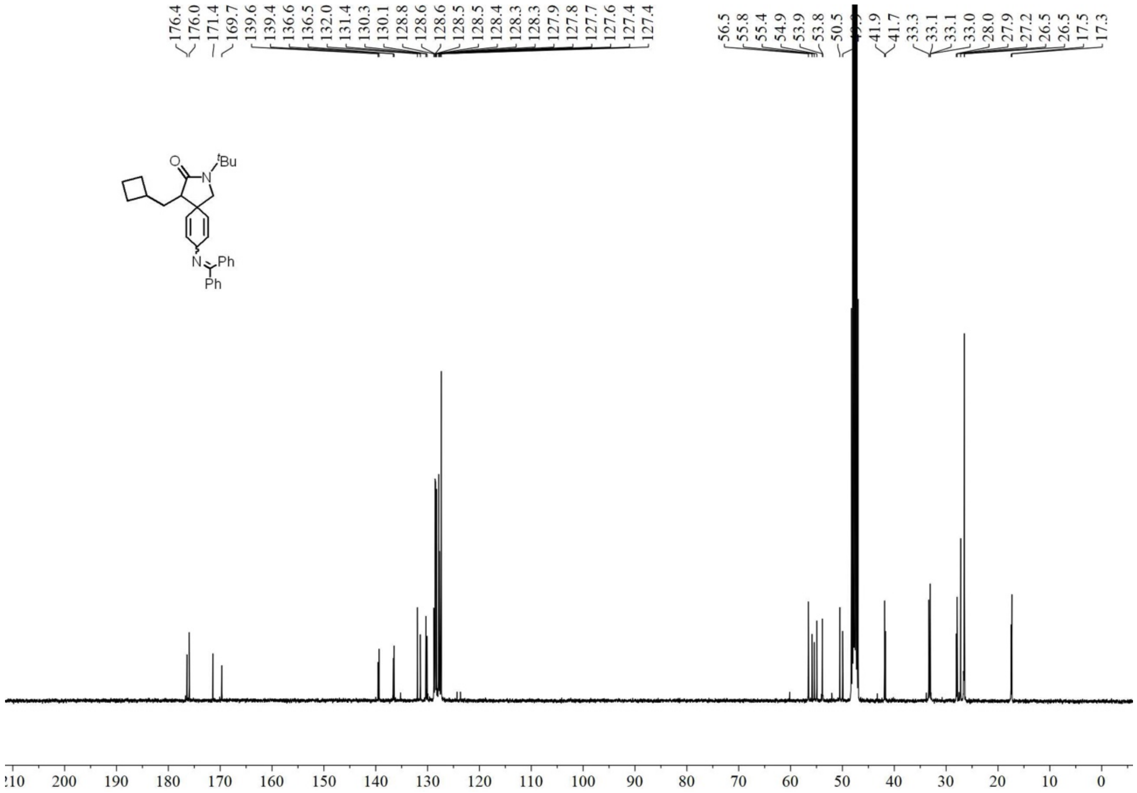

2-(*tert*-Butyl)-4-(cyclohexylmethyl)-8-((diphenylmethylene)amino)-2-azaspiro[4.5]deca-6,9-dien-3-one (3e, 8a)

^{1}H NMR (400 MHz, CD_3OD)

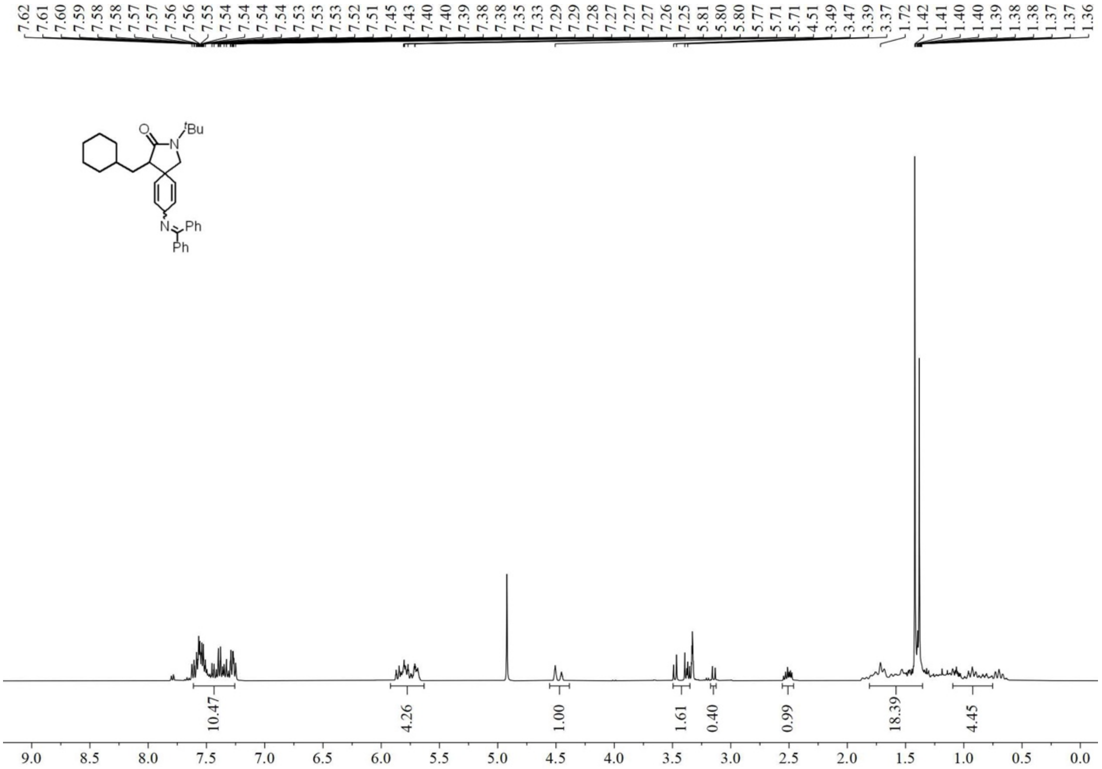

^{13}C NMR (101 MHz, CD_3OD)

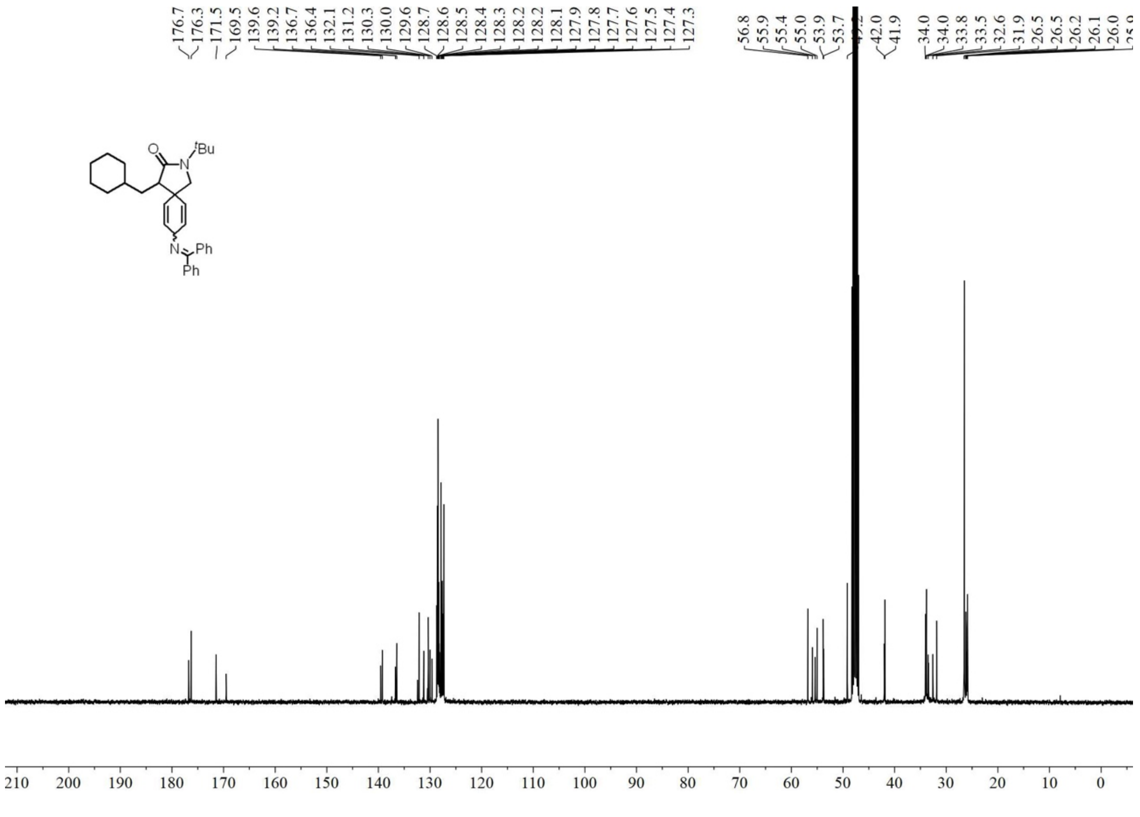

2-(*tert*-Butyl)-4-((4,4-difluorocyclohexyl)methyl)-8-((diphenylmethylene)amino)-2-azaspiro[4.5]deca-6,9-dien-3-one (3f)

^{1}H NMR (400 MHz, CD_3CN)

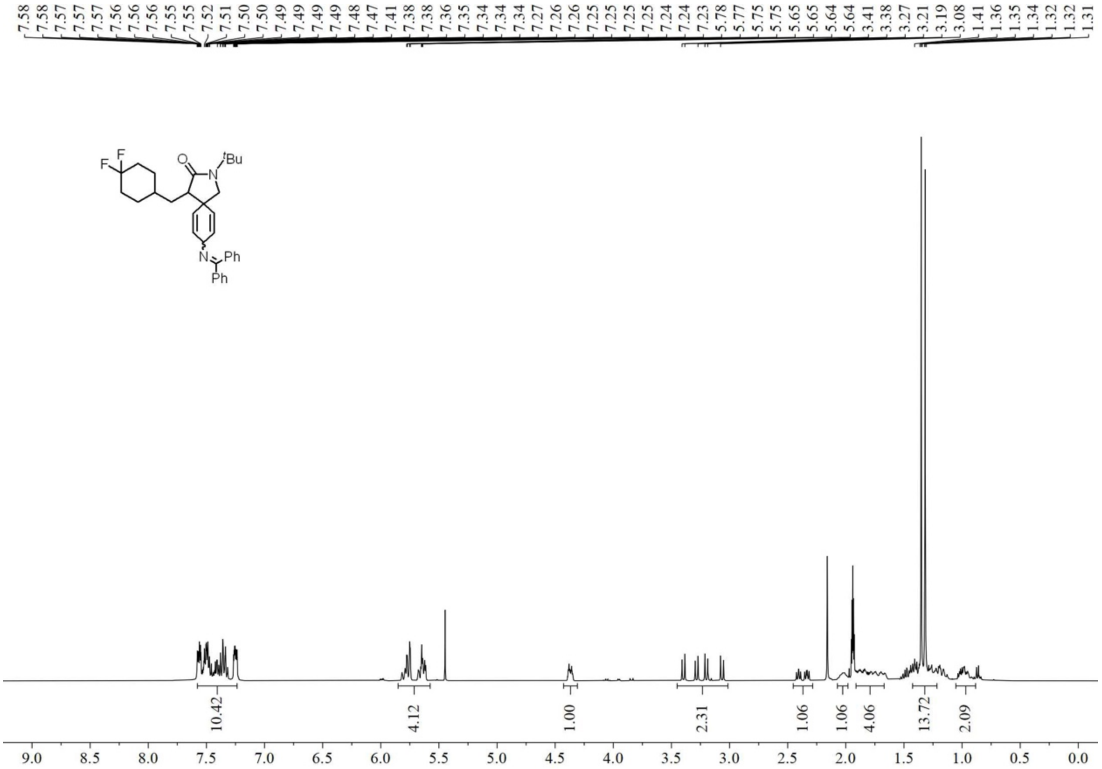

^{13}C NMR (101 MHz, CD_3CN)

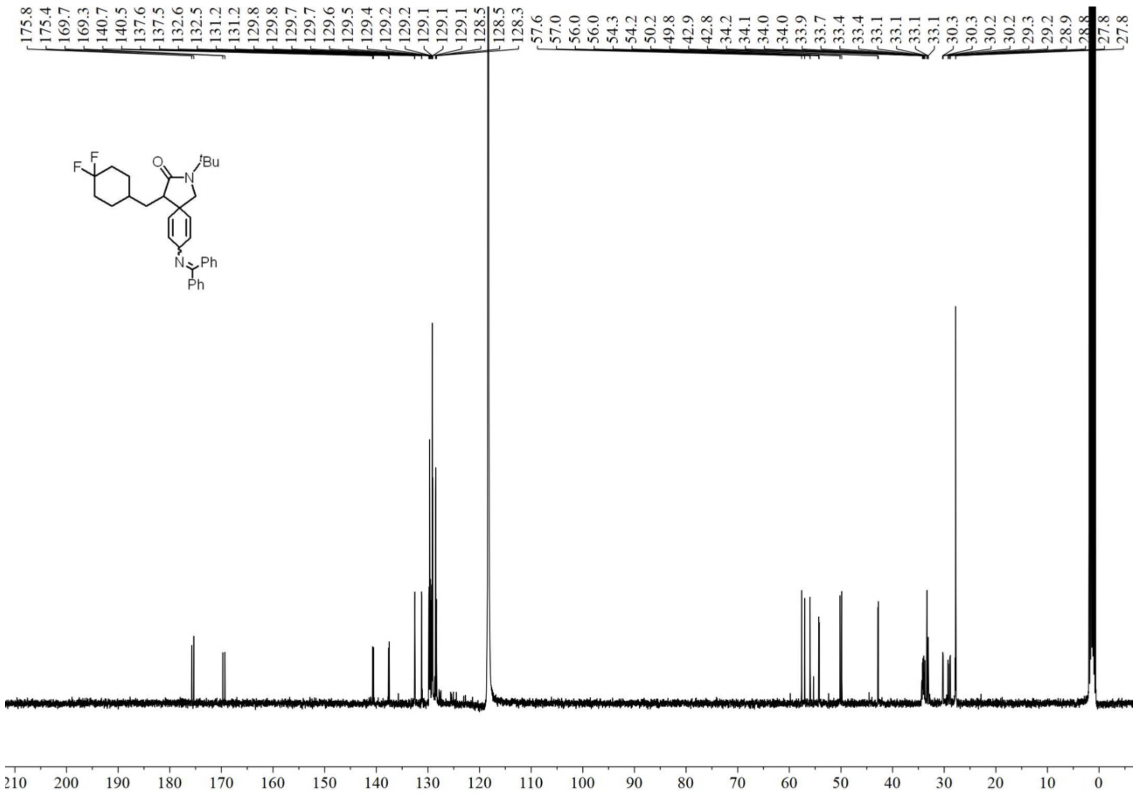

^{19}F NMR (377 MHz, CD_3CN)

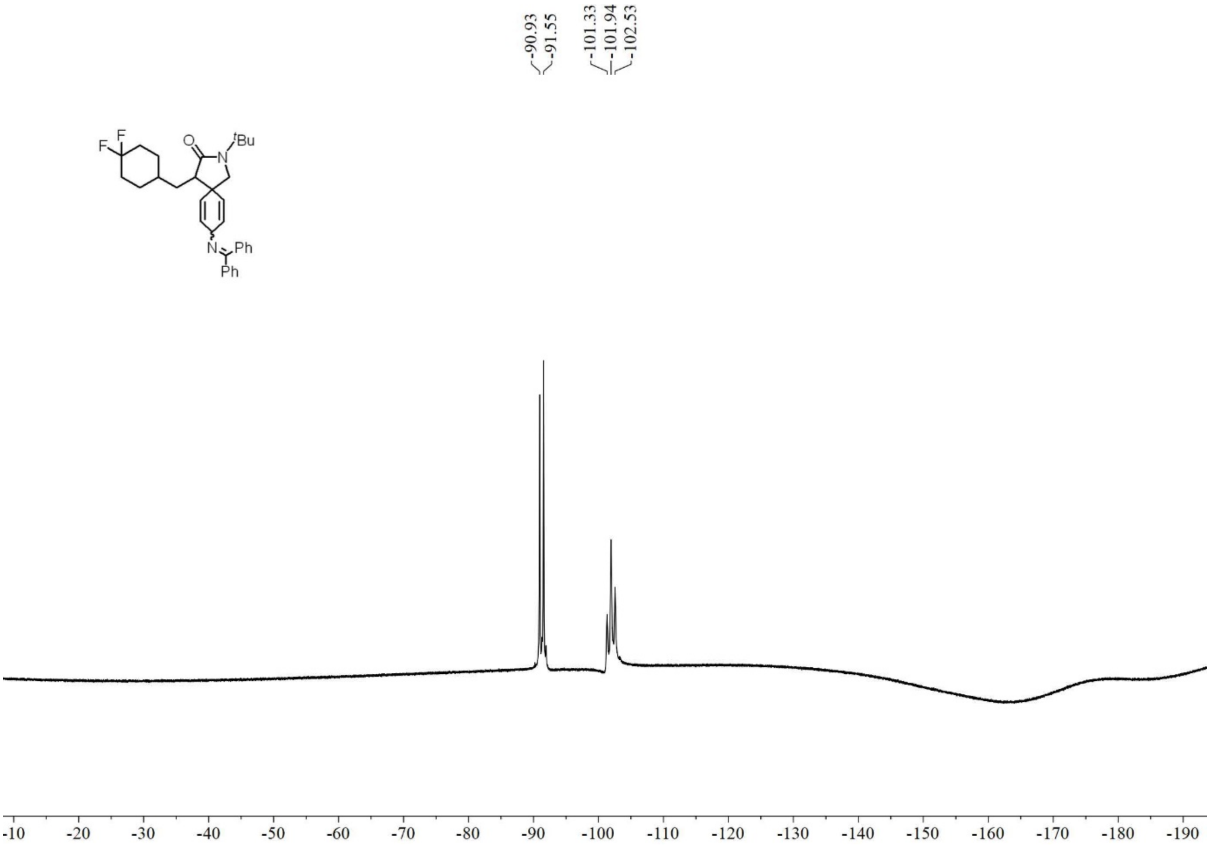

2-(*tert*-Butyl)-8-((diphenylmethylene)amino)-4-neopentyl-2-azaspiro[4.5]deca-6,9-dien-3-one (3g)

^{1}H NMR (500 MHz, CD_3OD)

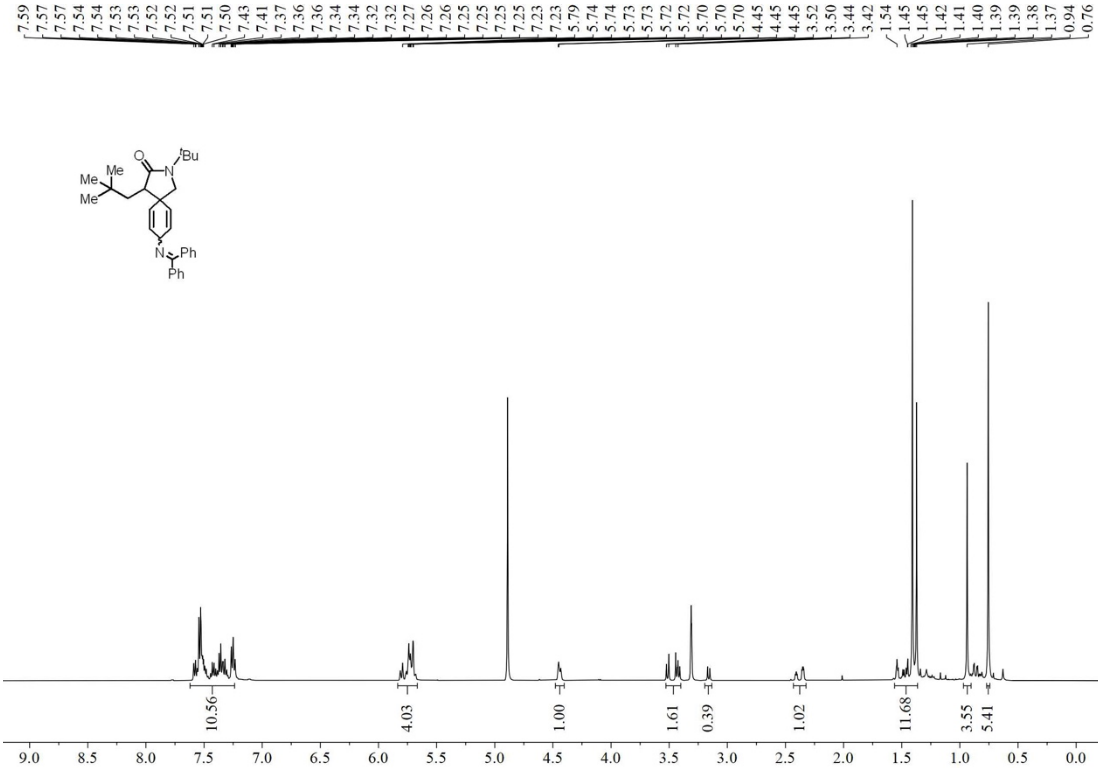

^{13}C NMR (126 MHz, CD_3OD)

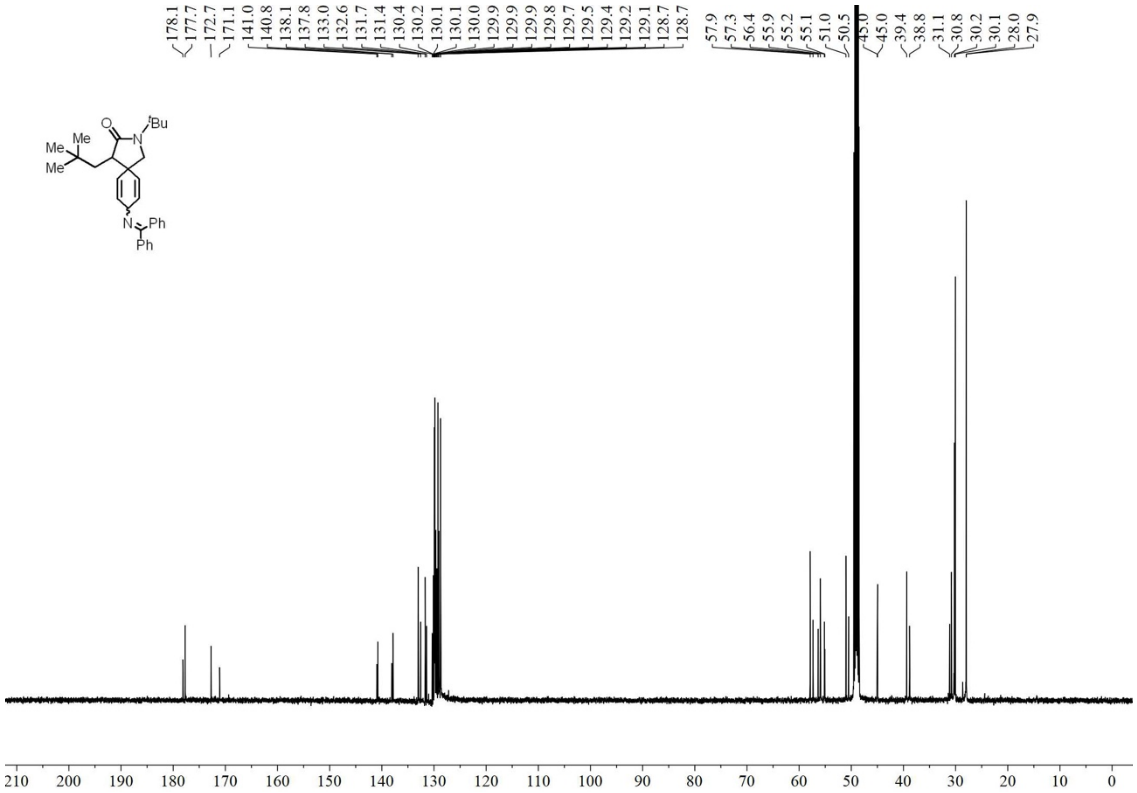

4-(Adamantan-1-ylmethyl)-2-(*tert*-butyl)-8-((diphenylmethylene)amino)-2-azaspiro[4.5]deca-6,9-dien-3-one (3h)

^{1}H NMR (400 MHz, CD_3OD)

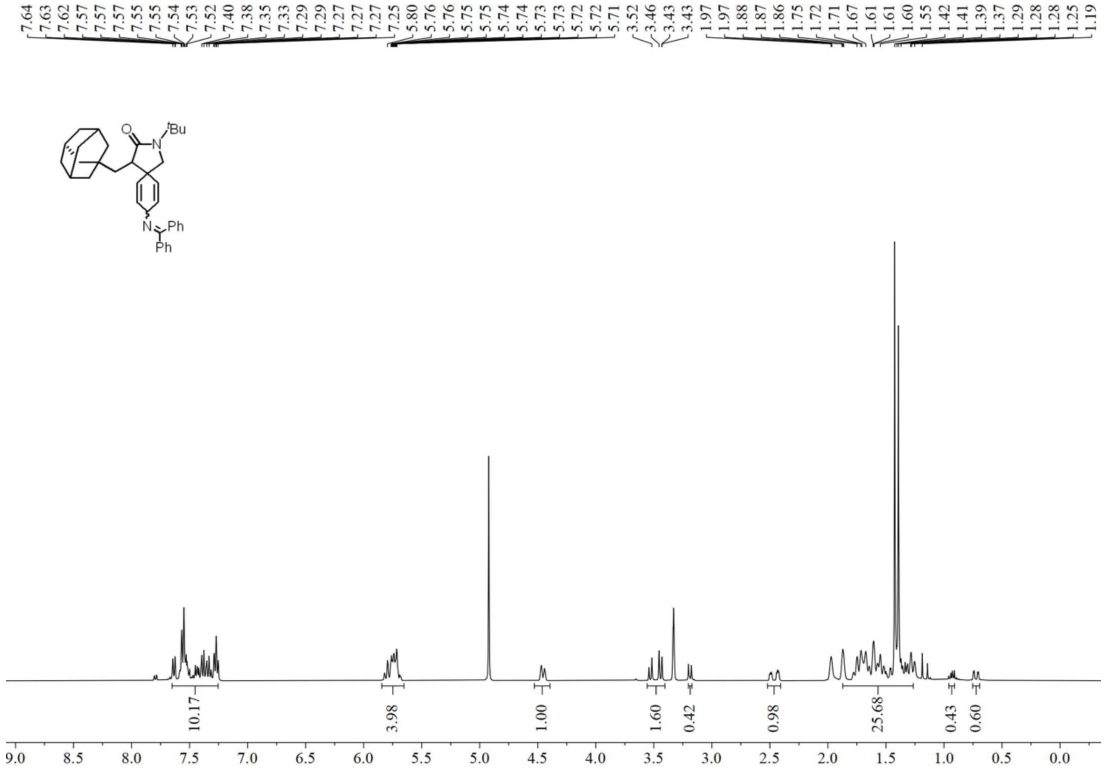

^{13}C NMR (101 MHz, CD_3OD)

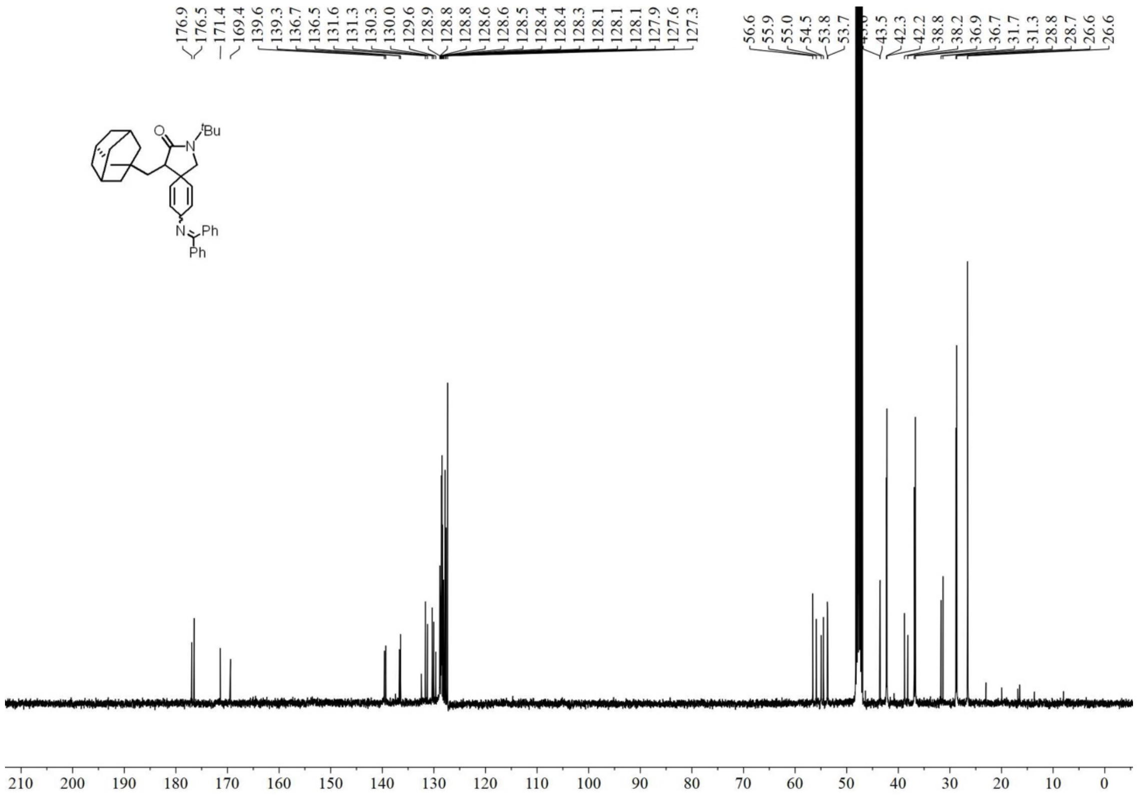

7-(*tert*-butyl)-2-((diphenylmethylene)amino)-9-(3-phenylpropyl)-1-thia-7-azaspiro[4.4]non-3-en-8-one (3i)

Major isomer A

^{1}H NMR (500 MHz, CD_3OD)

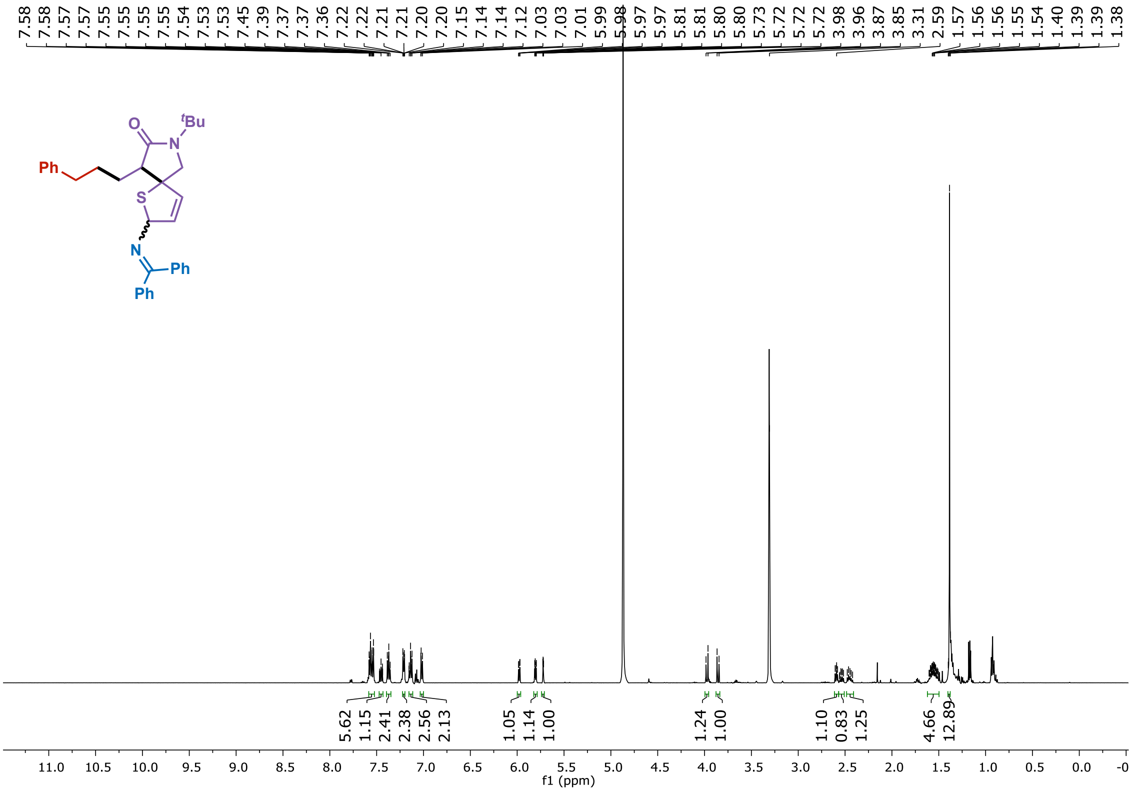

^{13}C NMR (126 MHz, CD_3OD)

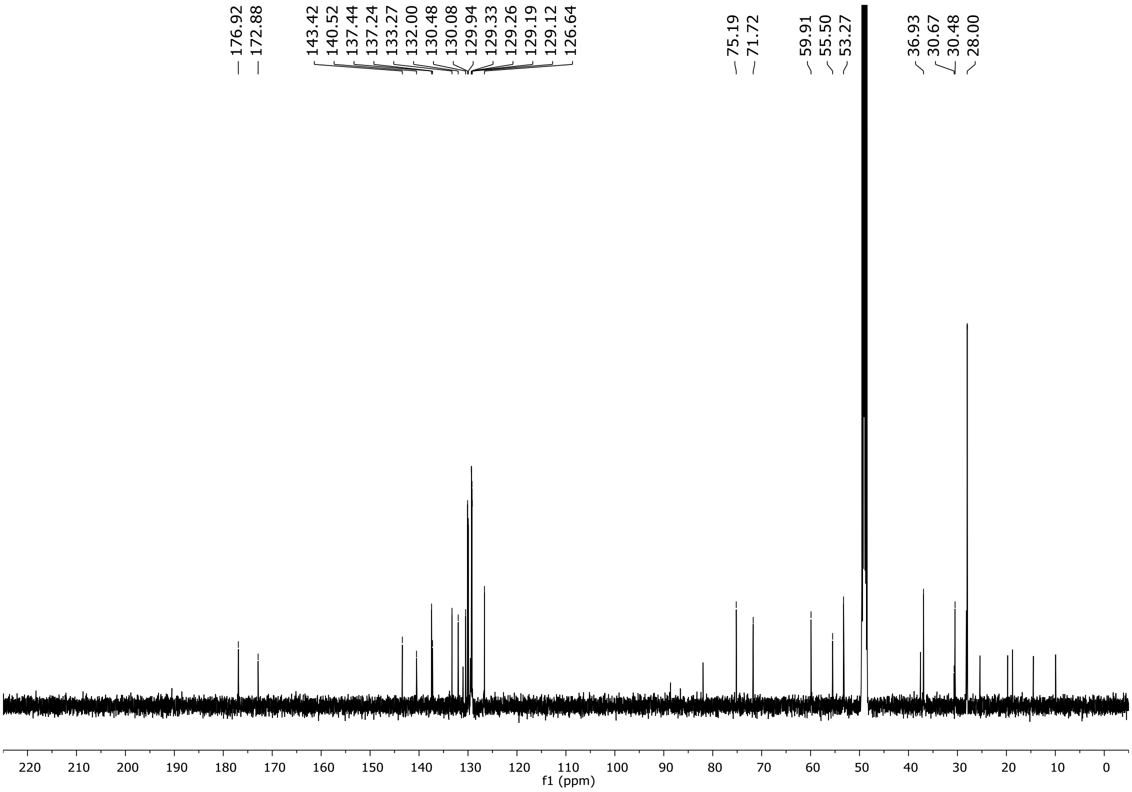

COSY

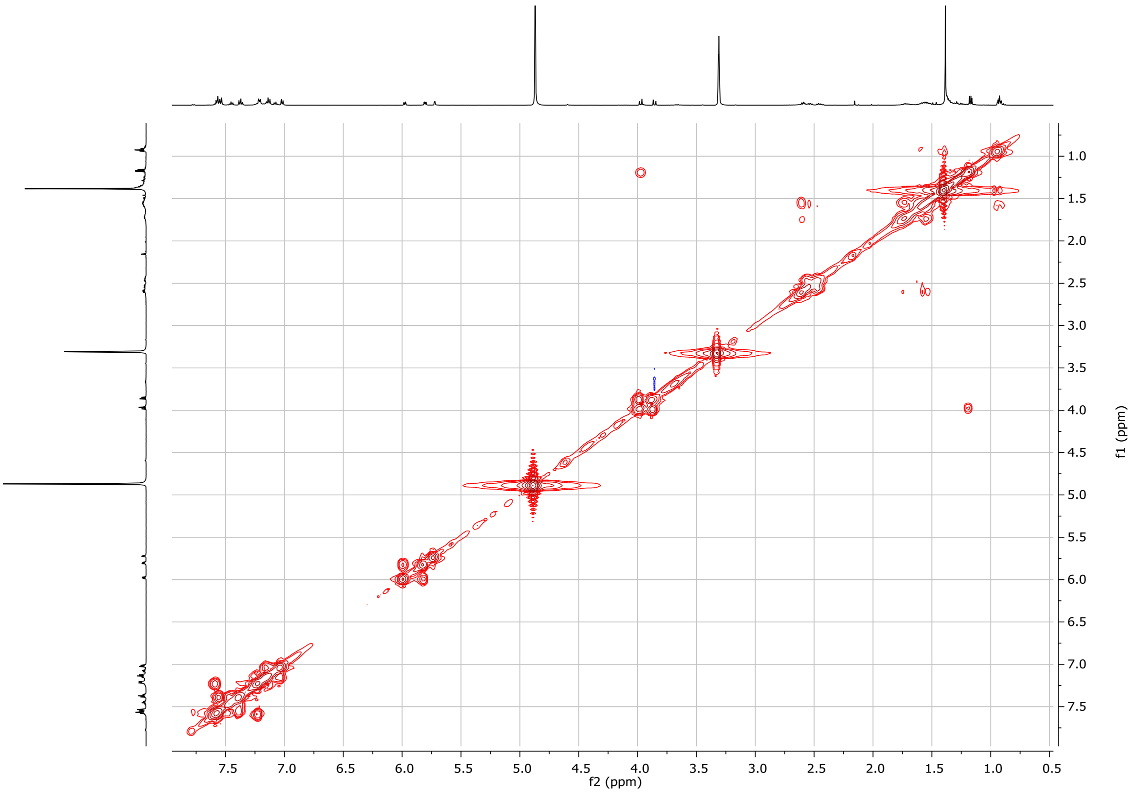

NOE

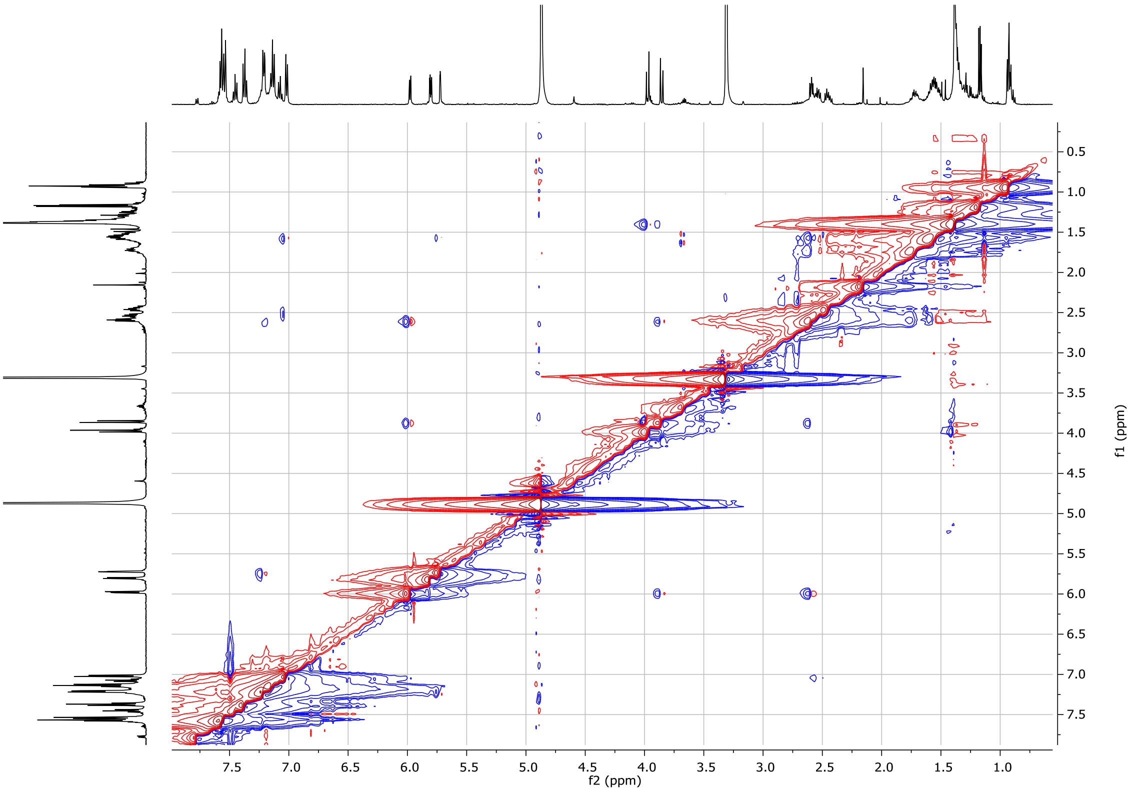

HSQC

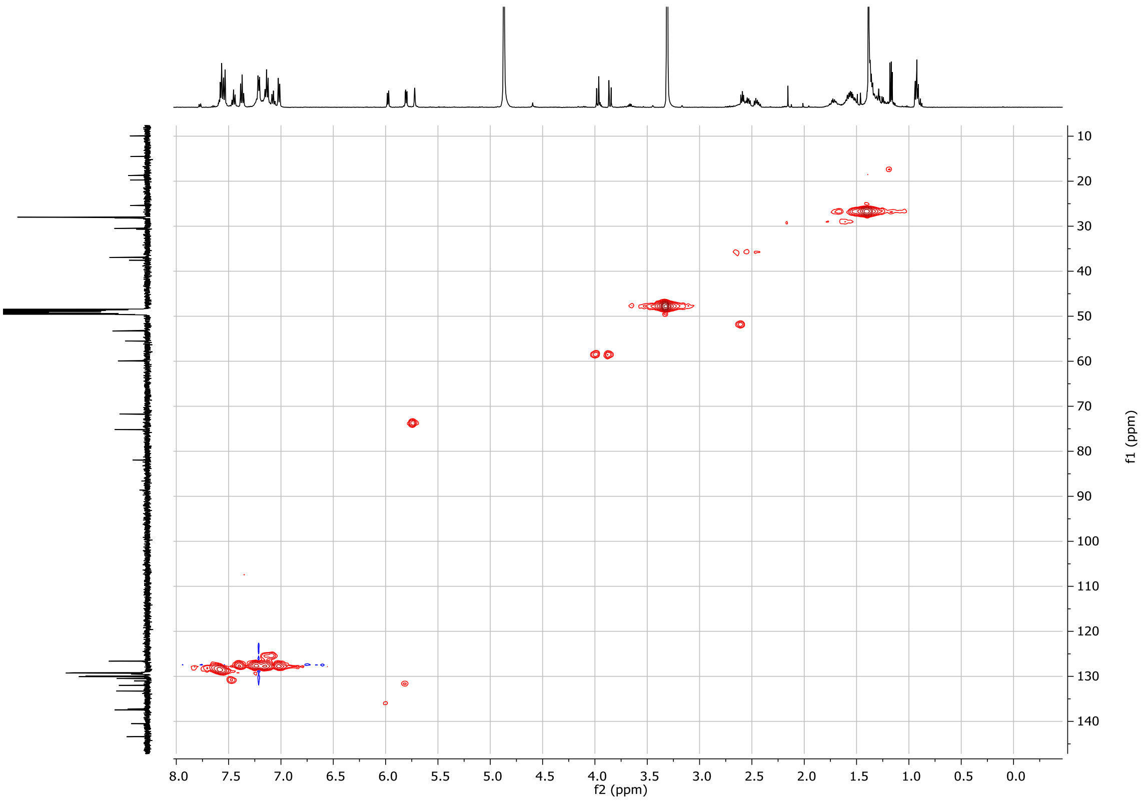

8.0 7.5 7.0 6.5 6.0 5.5 5.0 4.5 4.0 3.5 3.0 2.5 2.0 1.5 1.0 0.5 0.0
f2 (ppm)
10 20 30 40 50 60 70 80 90 100 110 120 130 140
f1 (ppm)

Mixture of two minor isomers B and C

^1H NMR (500 MHz, CD_3OD)

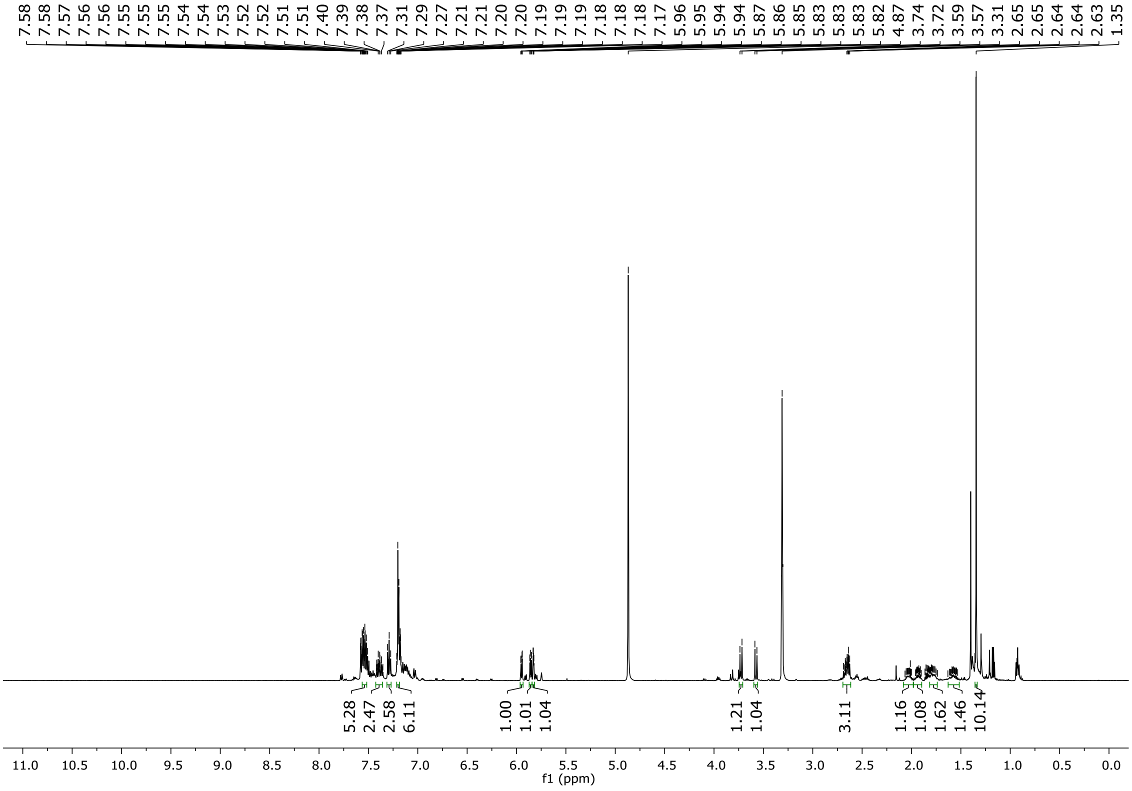

^{13}C NMR (126 MHz, CD_3OD)

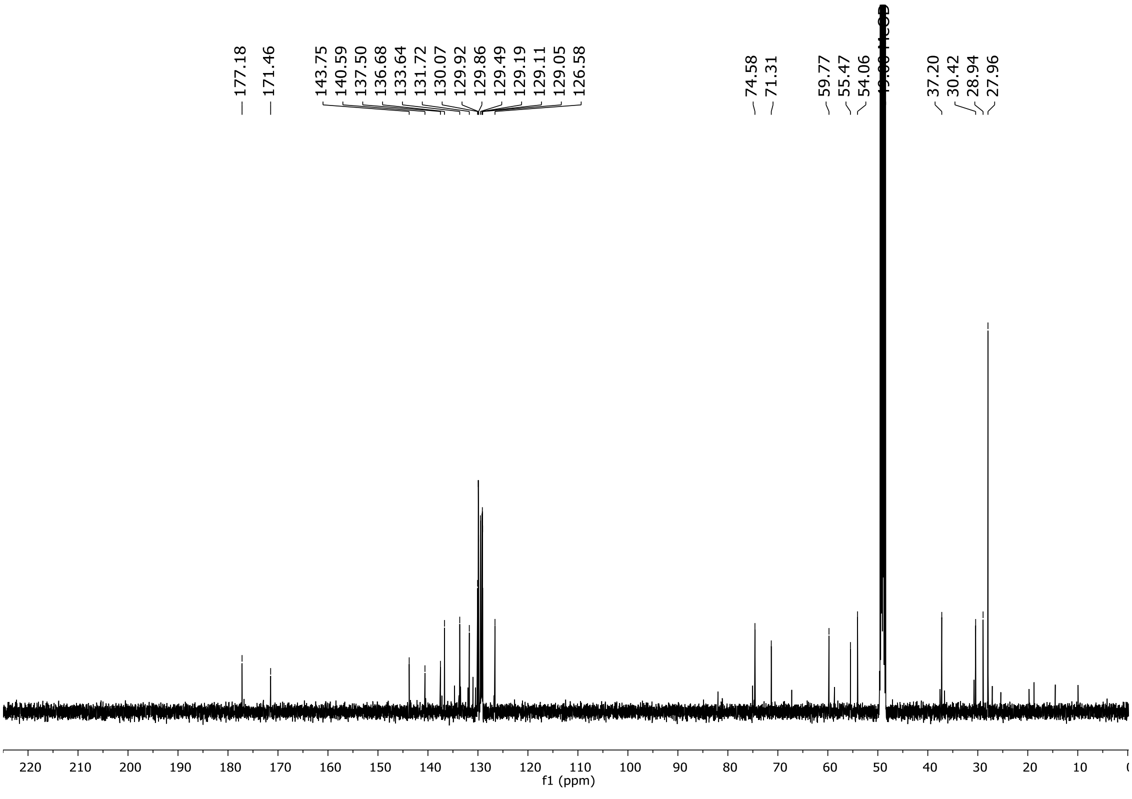

1'-(*tert*-butyl)-1-((diphenylmethylene)amino)-4'-(3-phenylpropyl)-1H-spiro[dibenzo[b,d]furan-4,3'-pyrrolidin]-5'-one (3j)

Major isomer A

^{1}H NMR (500 MHz, CD_3OD)

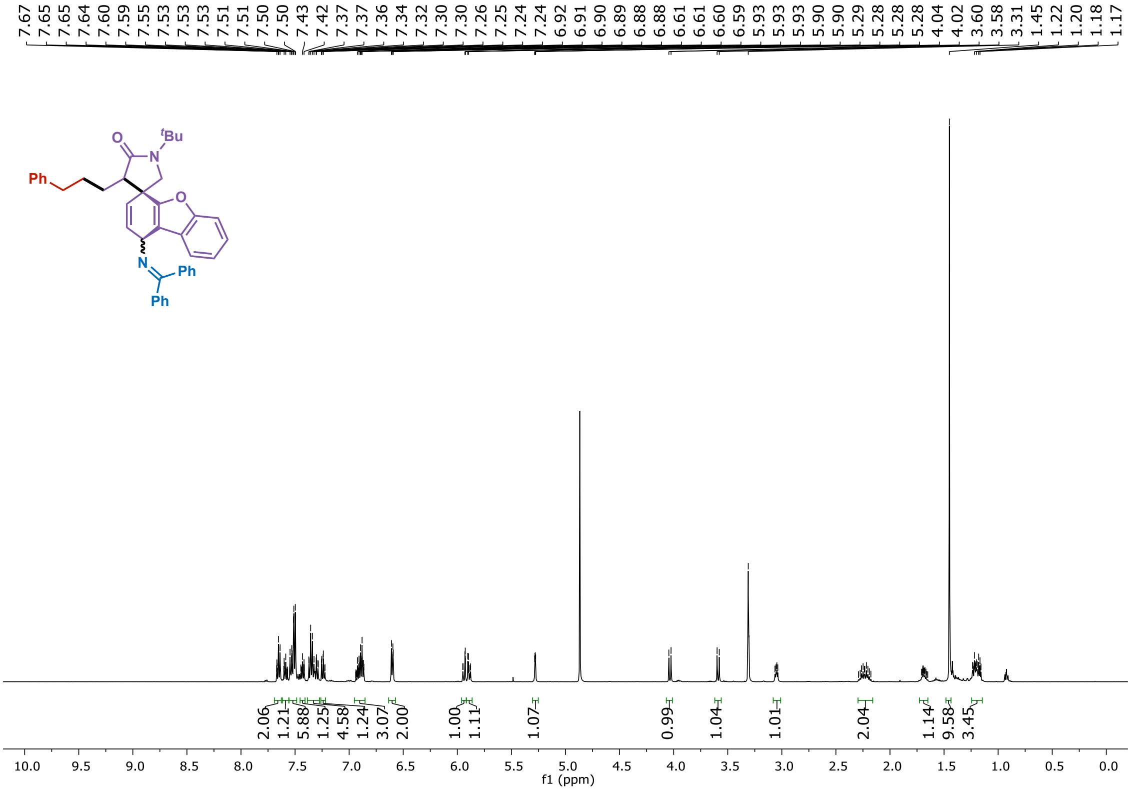

^{13}C NMR (126 MHz, CD_3OD)

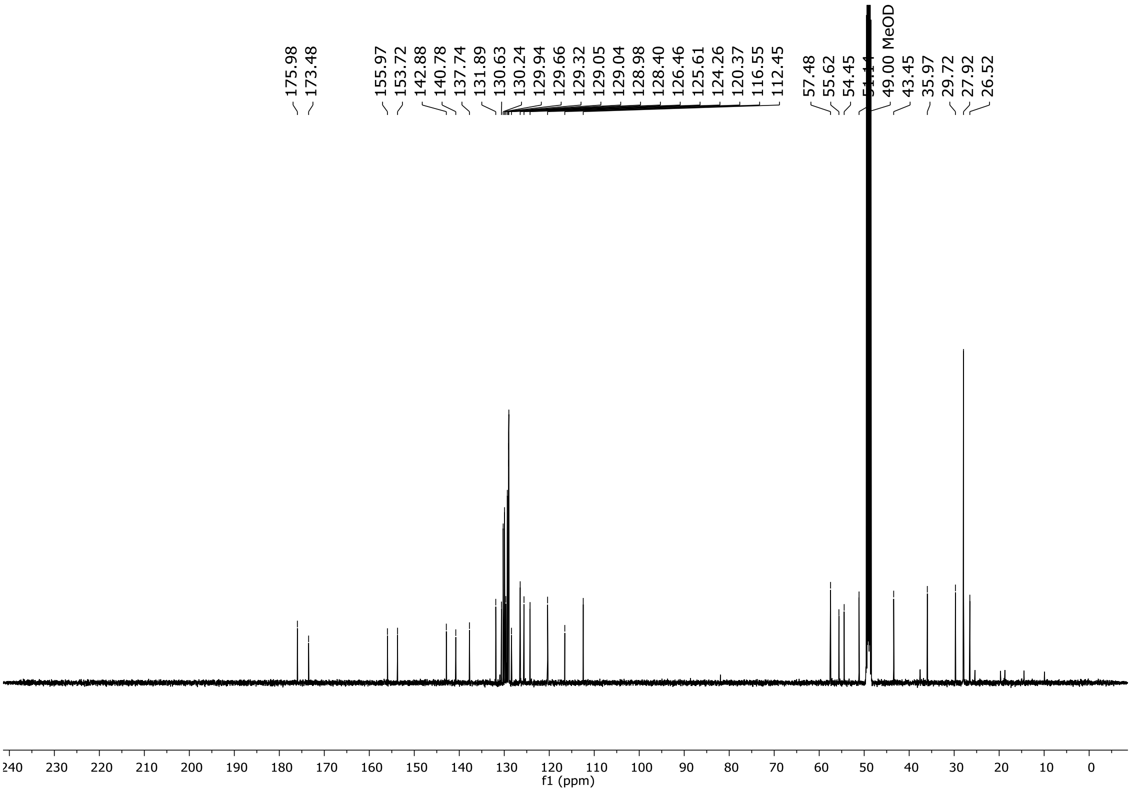

COSY

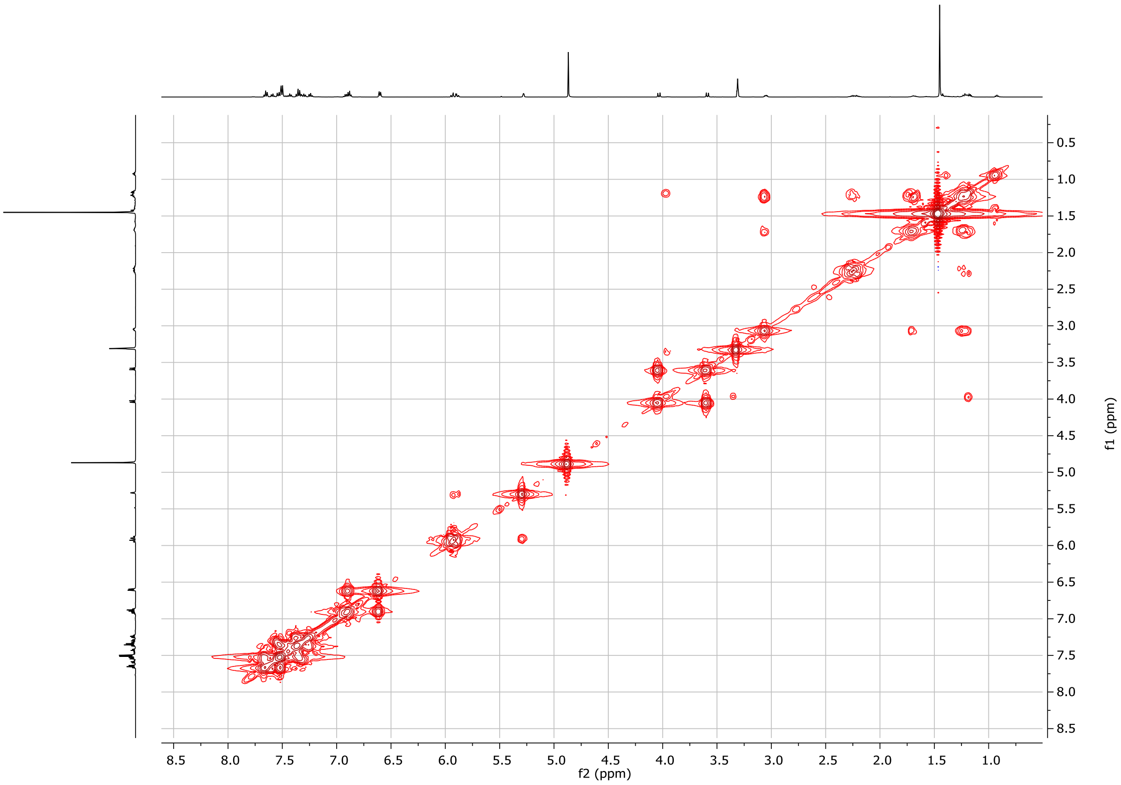

NOESY

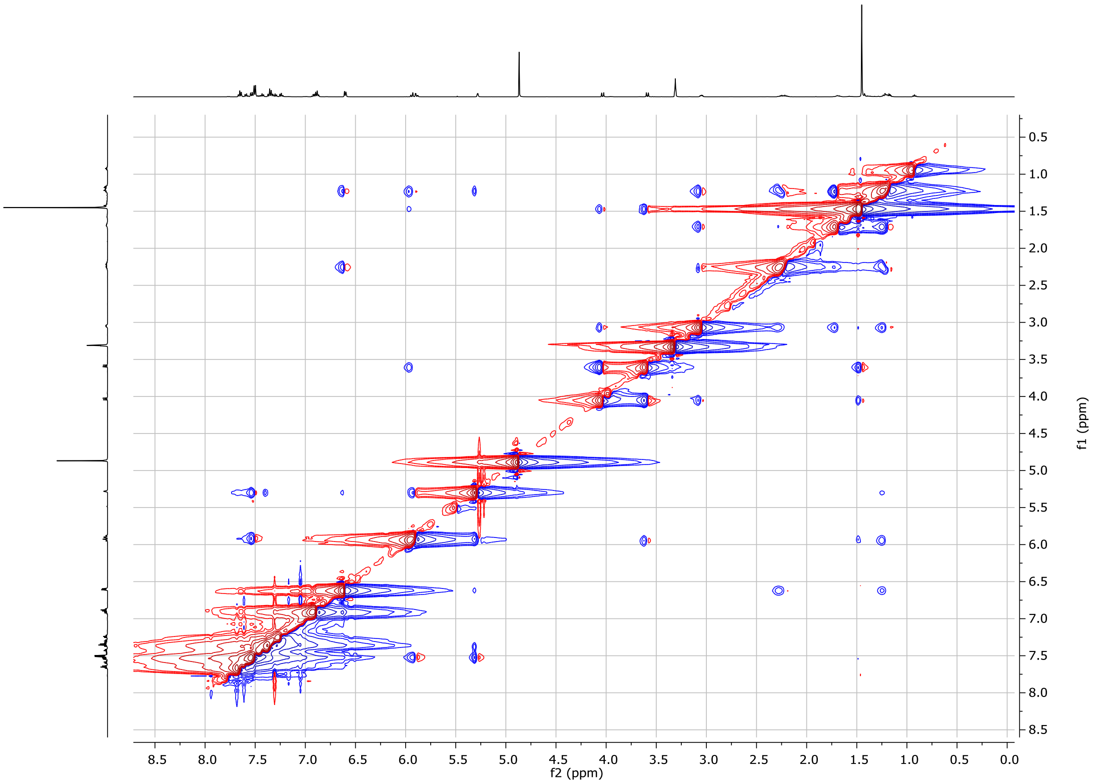

HSQC

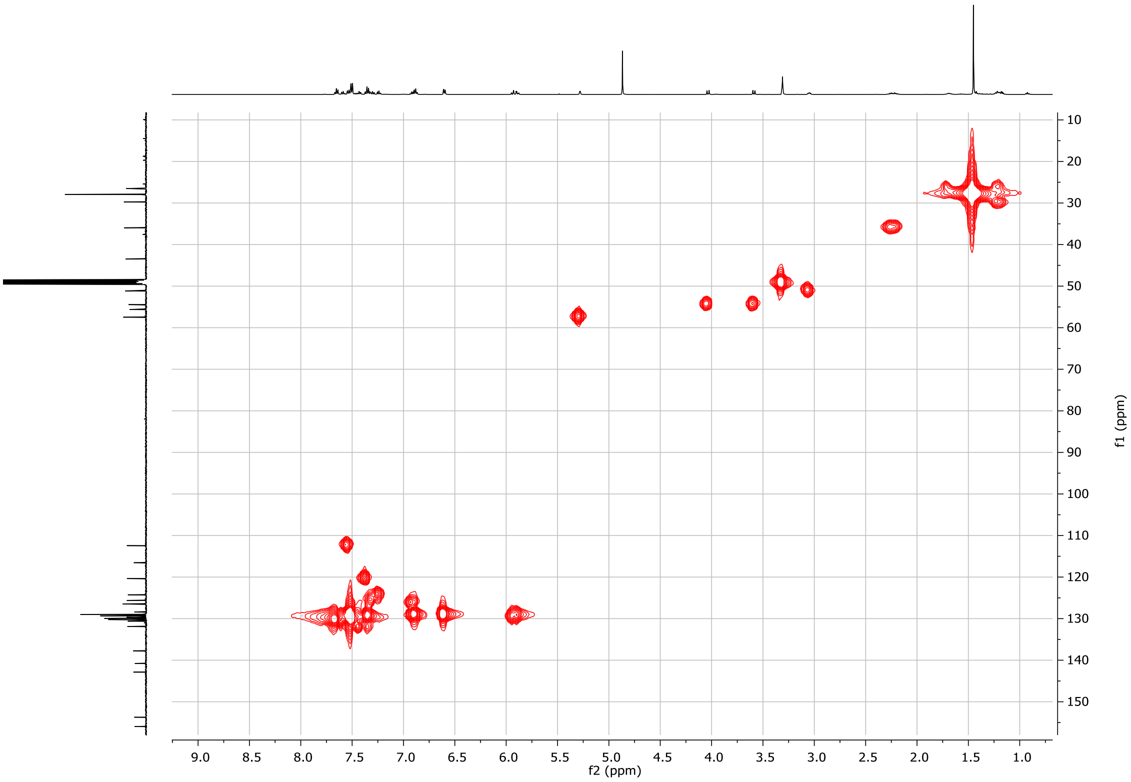

9.0 8.5 8.0 7.5 7.0 6.5 6.0 5.5 5.0 4.5 4.0 3.5 3.0 2.5 2.0 1.5 1.0
f2 (ppm)
10 20 30 40 50 60 70 80 90 100 110 120 130 140 150
f1 (ppm)

HMBC

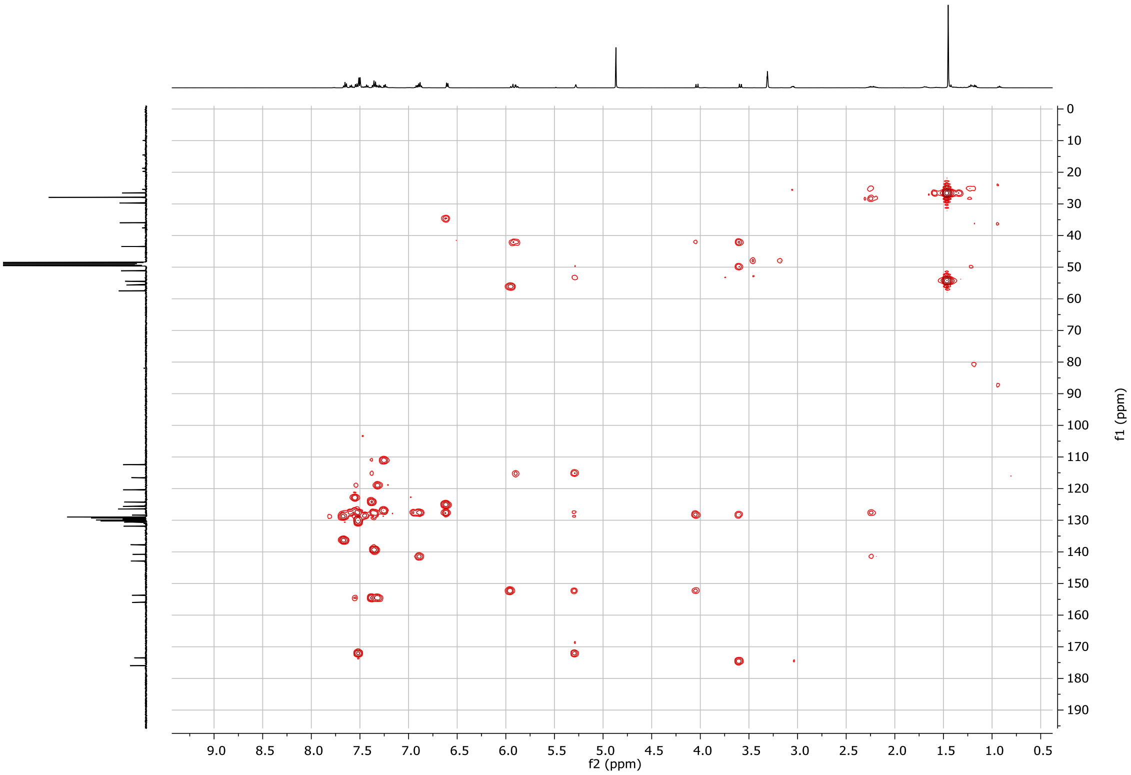

9.0 8.5 8.0 7.5 7.0 6.5 6.0 5.5 5.0 4.5 4.0 3.5 3.0 2.5 2.0 1.5 1.0 0.5
f2 (ppm)
0 10 20 30 40 50 60 70 80 90 100 110 120 130 140 150 160 170 180 190
f1 (ppm)

Minor isomer B

^1H NMR (500 MHz, CD_3OD)

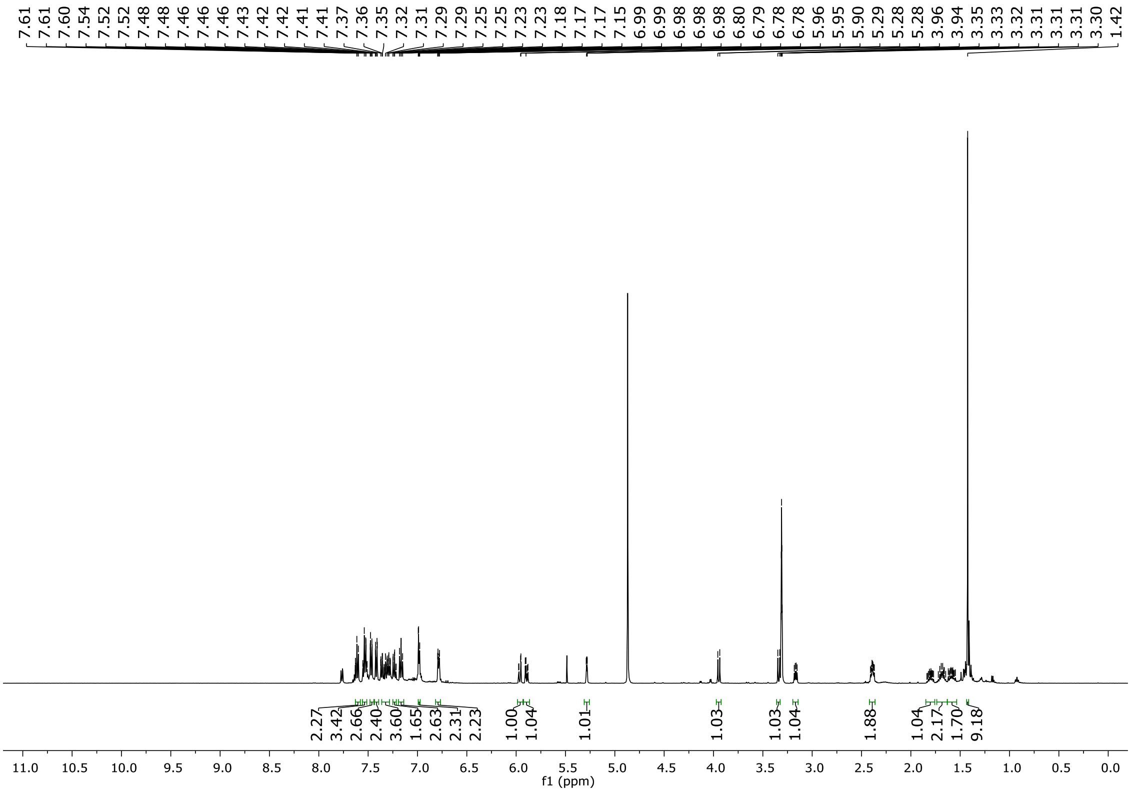

^{13}C NMR (126 MHz, CD_3OD)

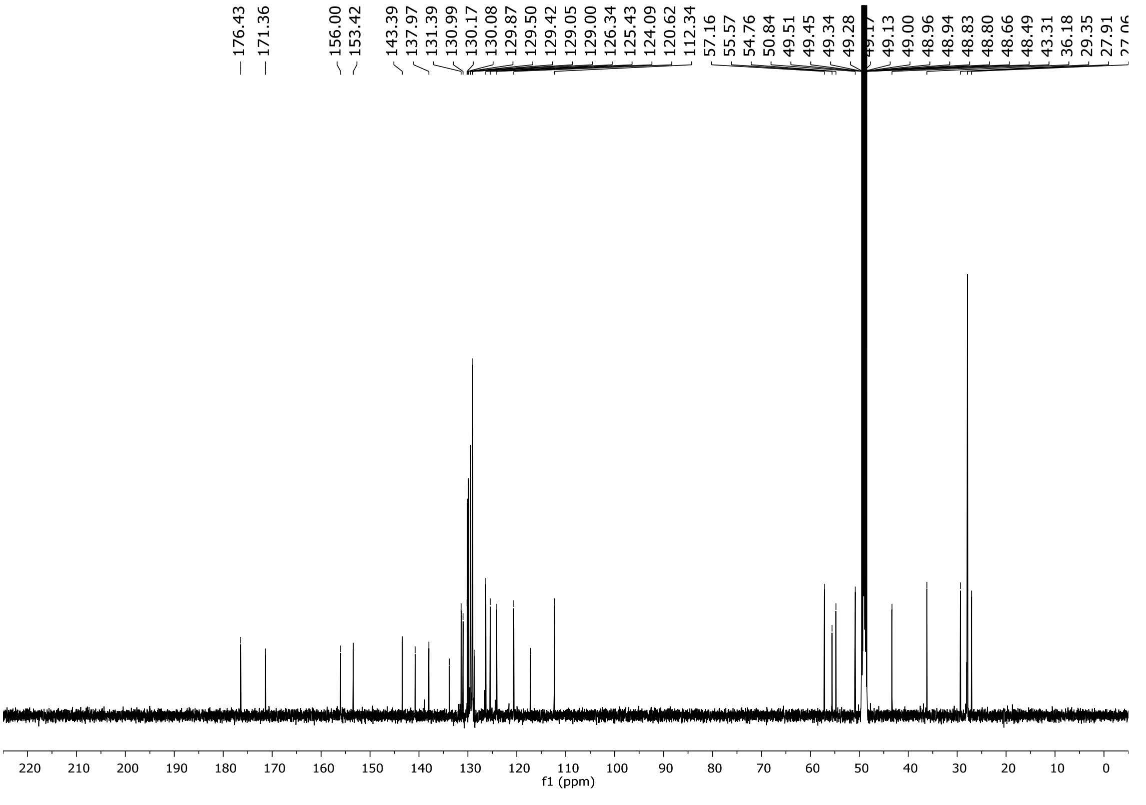

COSY

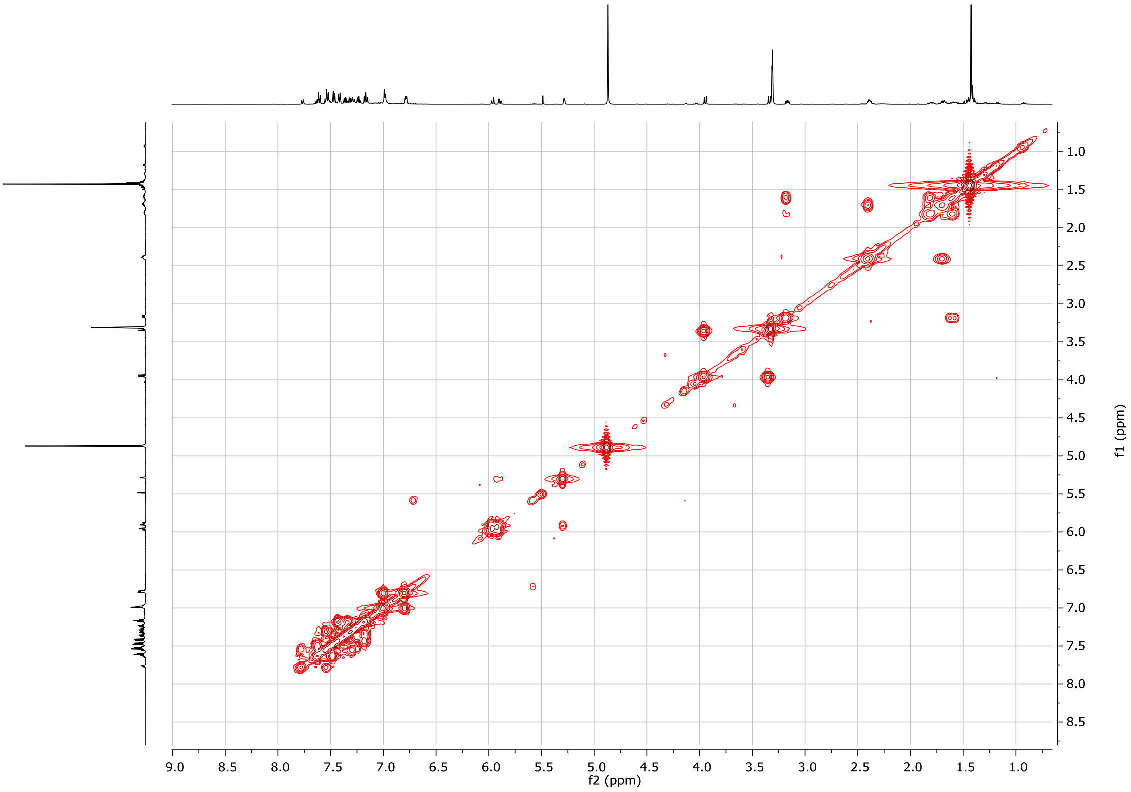

NOESY

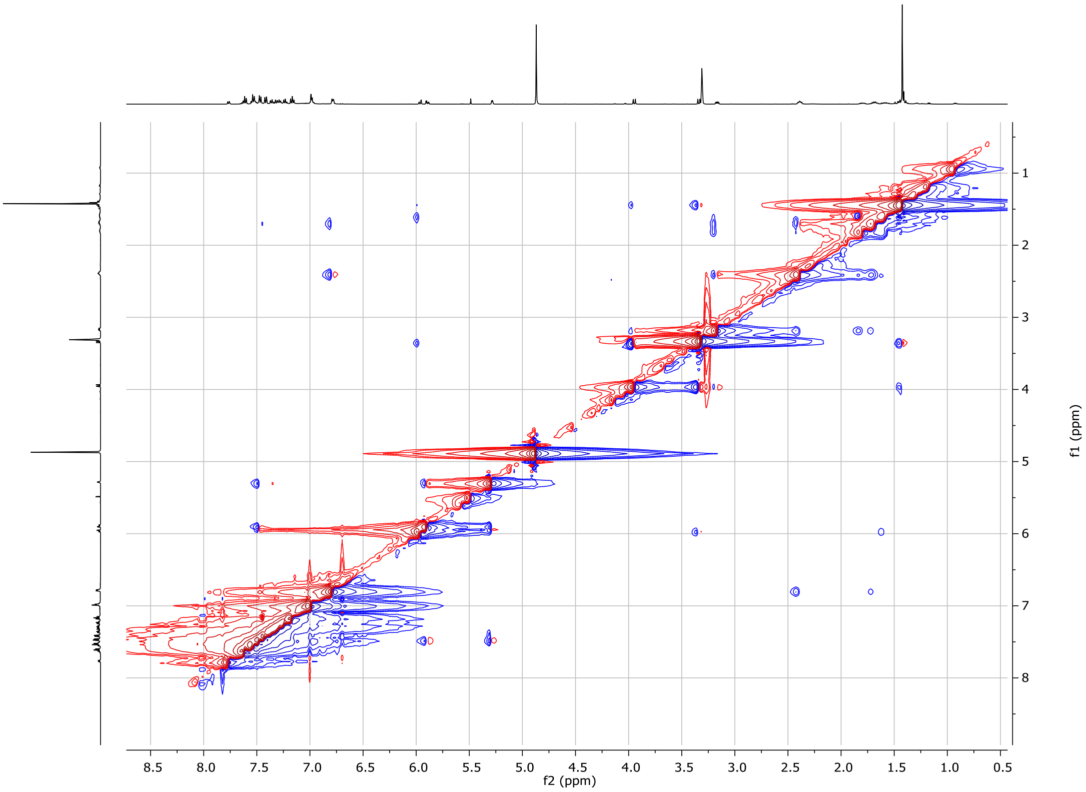

HSQC

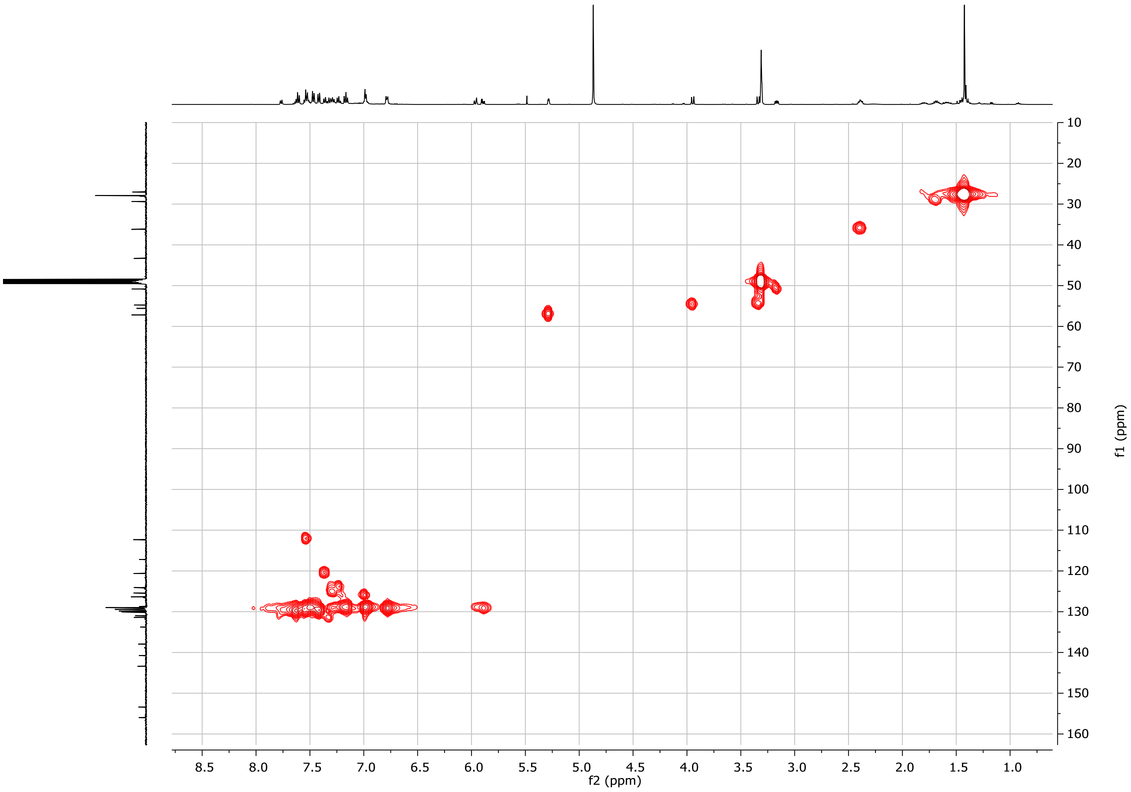
f1 (ppm)
f2 (ppm)
8.5 8.0 7.5 7.0 6.5 6.0 5.5 5.0 4.5 4.0 3.5 3.0 2.5 2.0 1.5 1.0
10 20 30 40 50 60 70 80 90 100 110 120 130 140 150 160

HMBC

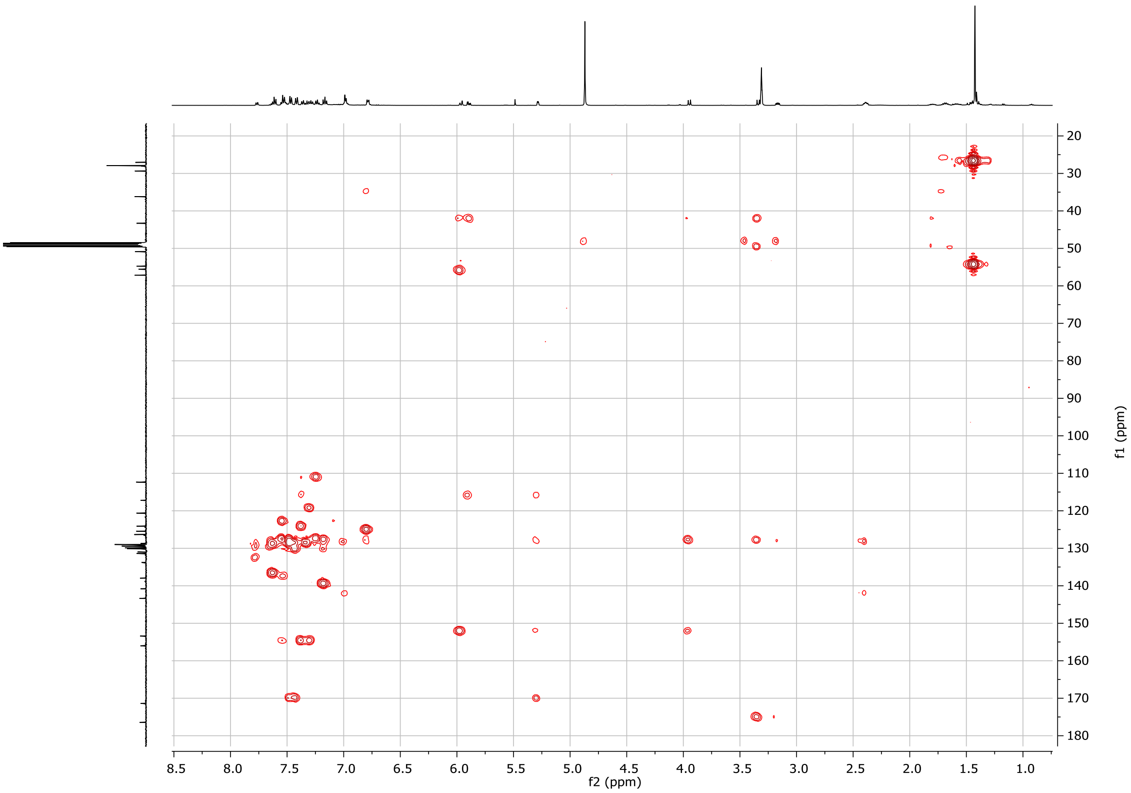
f1 (ppm)
f2 (ppm)
8.5 8.0 7.5 7.0 6.5 6.0 5.5 5.0 4.5 4.0 3.5 3.0 2.5 2.0 1.5 1.0
20 30 40 50 60 70 80 90 100 110 120 130 140 150 160 170 180

Minor isomer C

^{1}H NMR (500 MHz, CD_3OD)

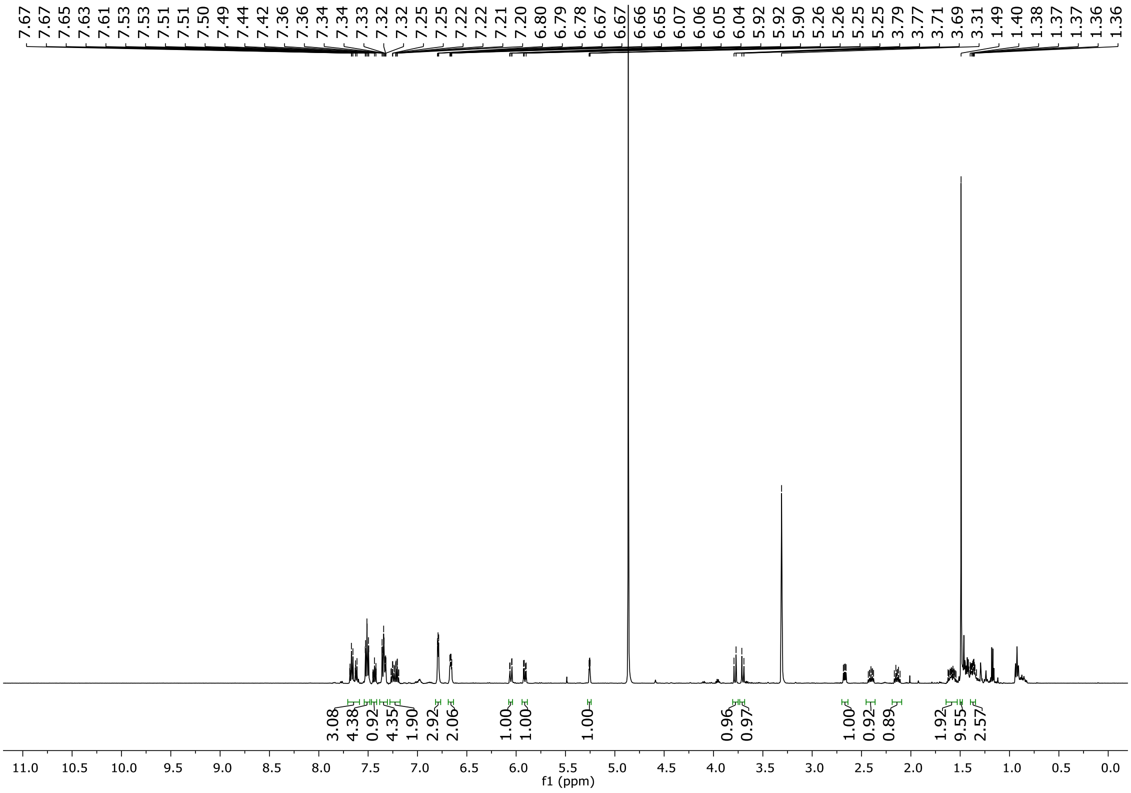

^{13}C NMR (126 MHz, CD_3OD)

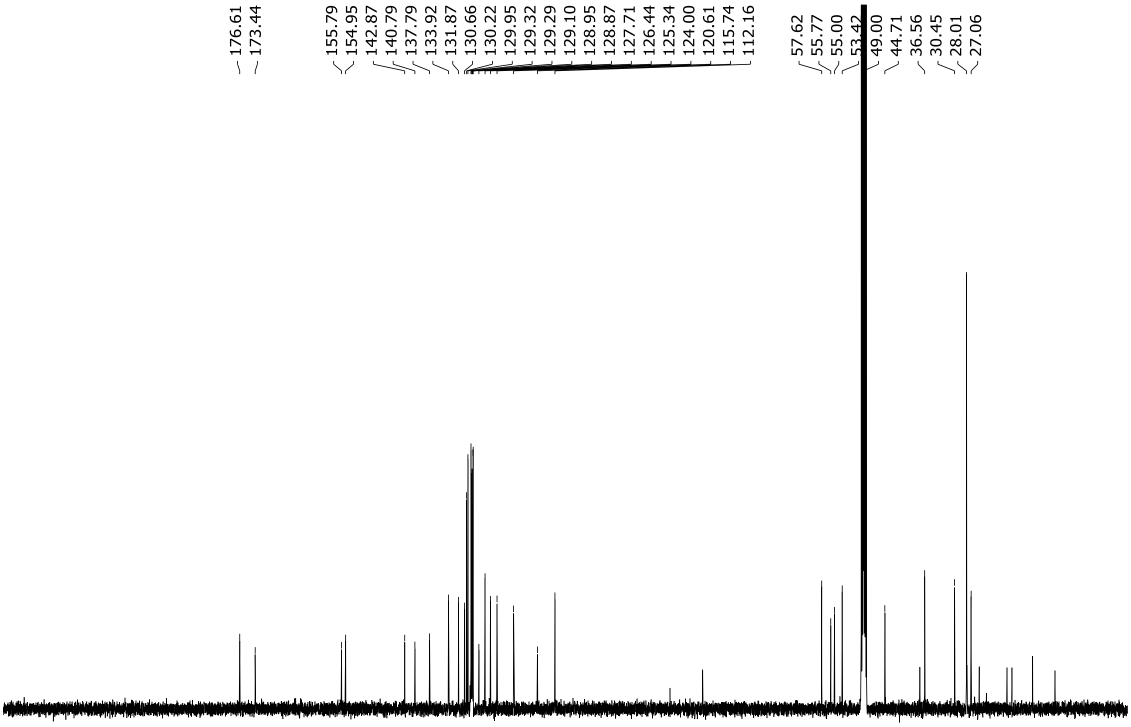

COSY

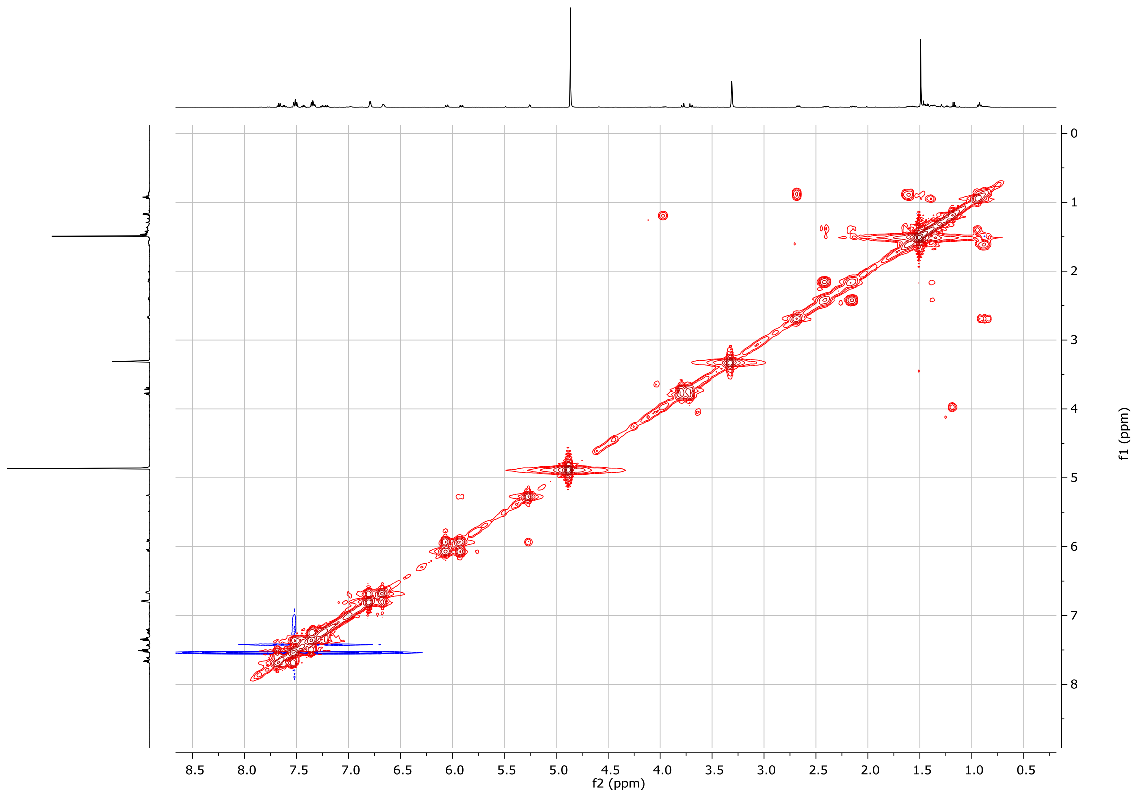

8.5
8.0
7.5
7.0
6.5
6.0
5.5
5.0
4.5
4.0
3.5
3.0
2.5
2.0
1.5
1.0
0.5
f2 (ppm)
0
1
2
3
4
5
6
7
8
f1 (ppm)

NOESY

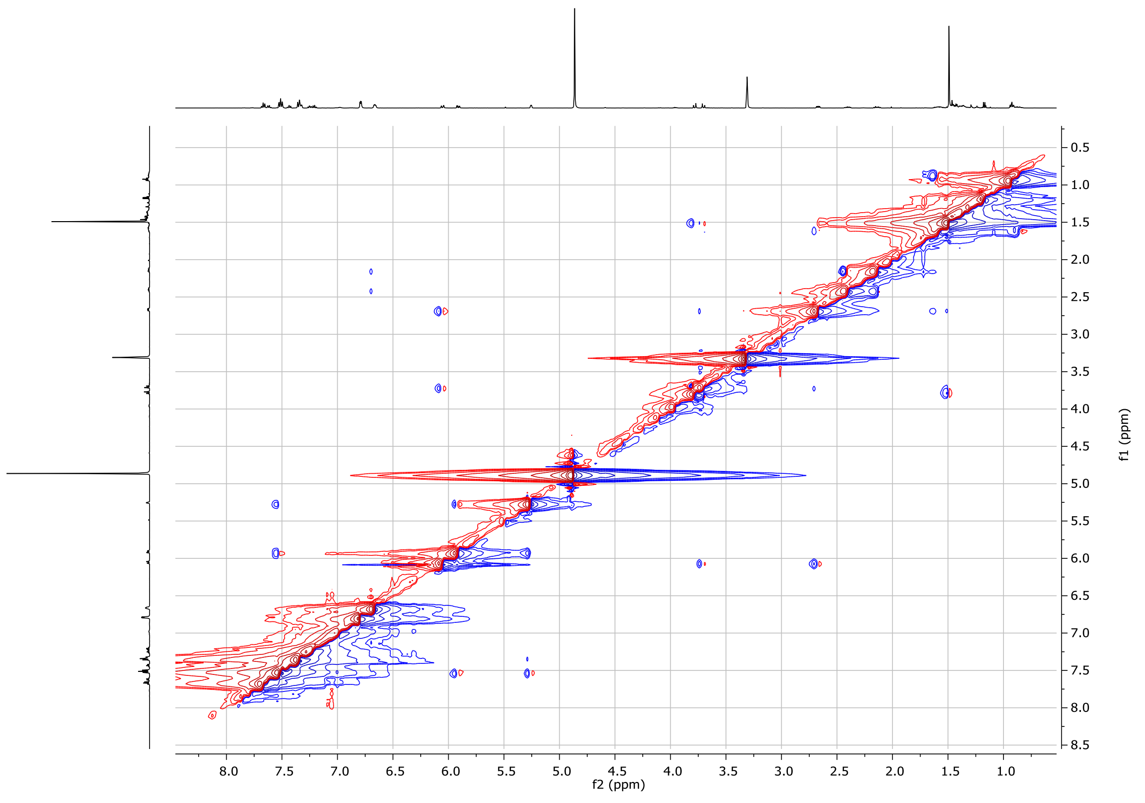

8.0
7.5
7.0
6.5
6.0
5.5
5.0
4.5
4.0
3.5
3.0
2.5
2.0
1.5
1.0
f2 (ppm)
0.5
1.0
1.5
2.0
2.5
3.0
3.5
4.0
4.5
5.0
5.5
6.0
6.5
7.0
7.5
8.0
8.5
f1 (ppm)

HSQC

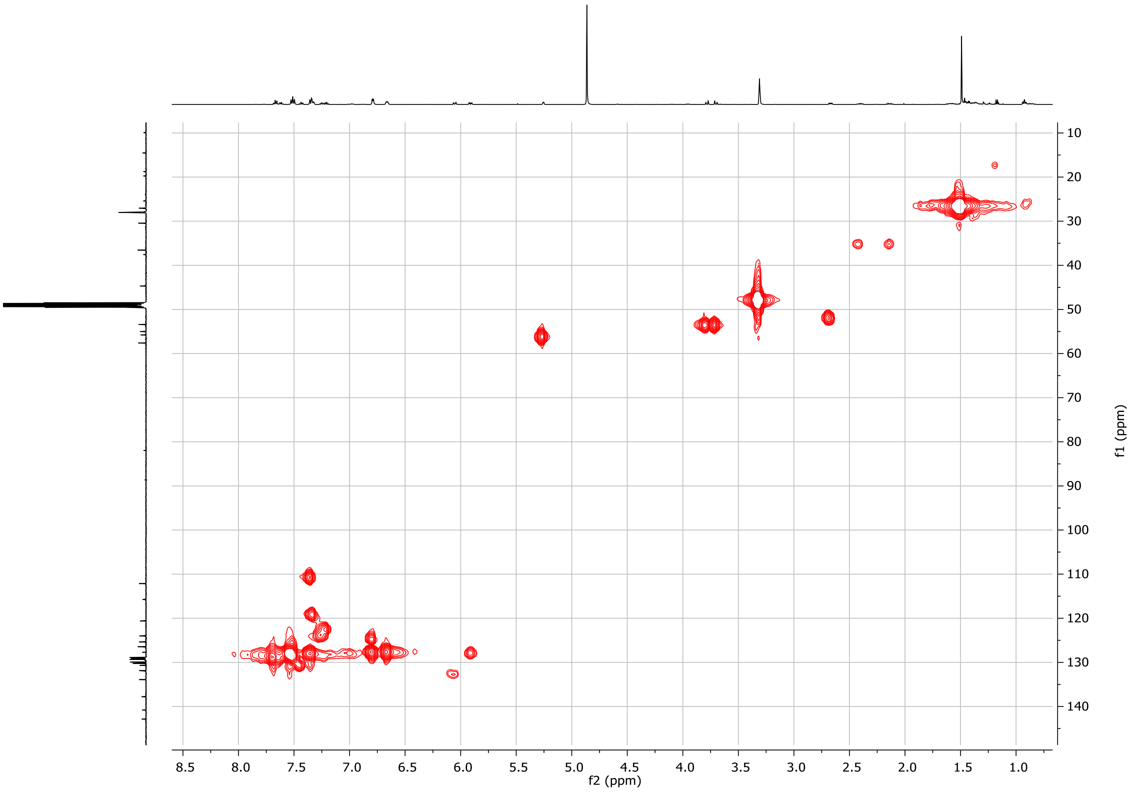

HMBC

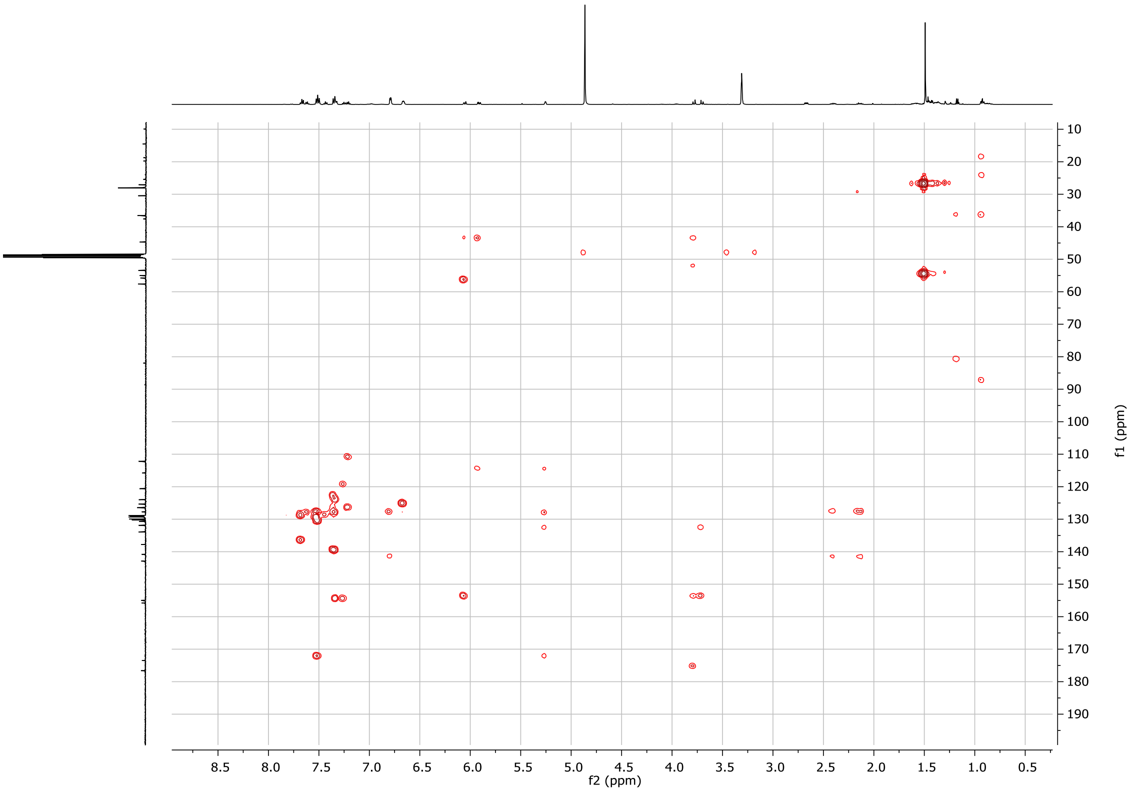

Mixture of isomers A and D

^{1}H NMR (500 MHz, CD_3OD)

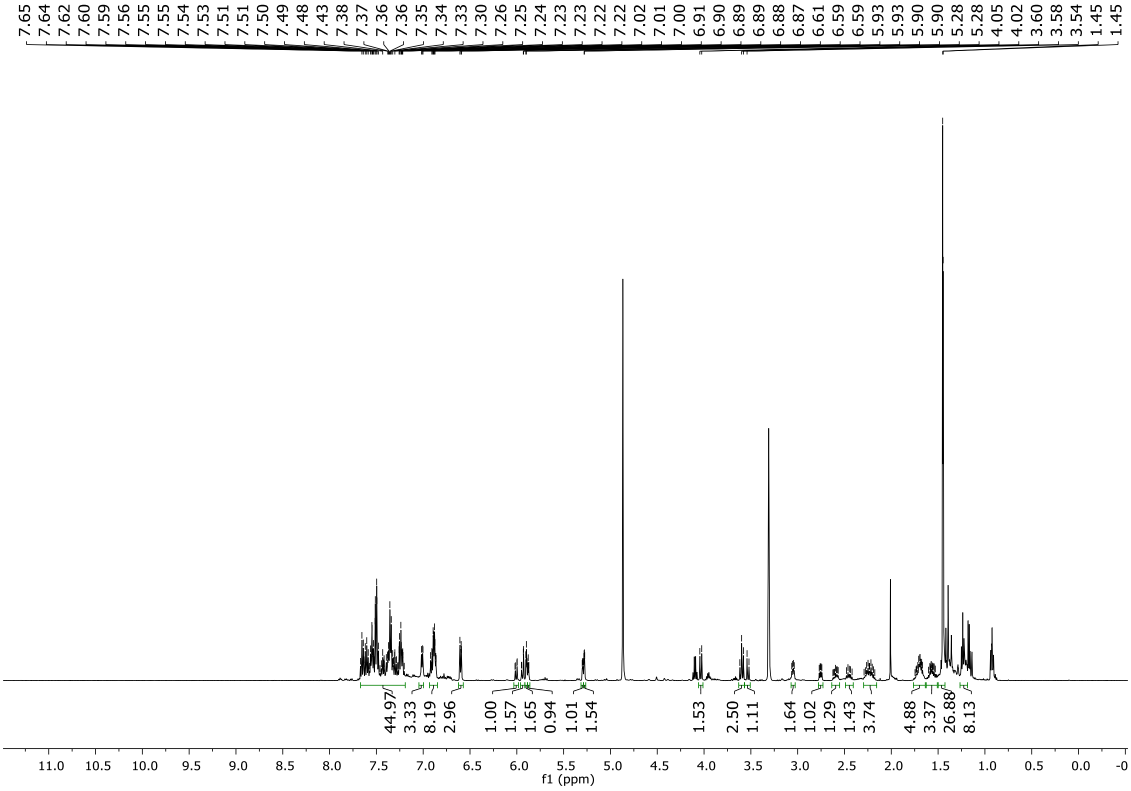

^{13}C NMR (126 MHz, CD_3OD)

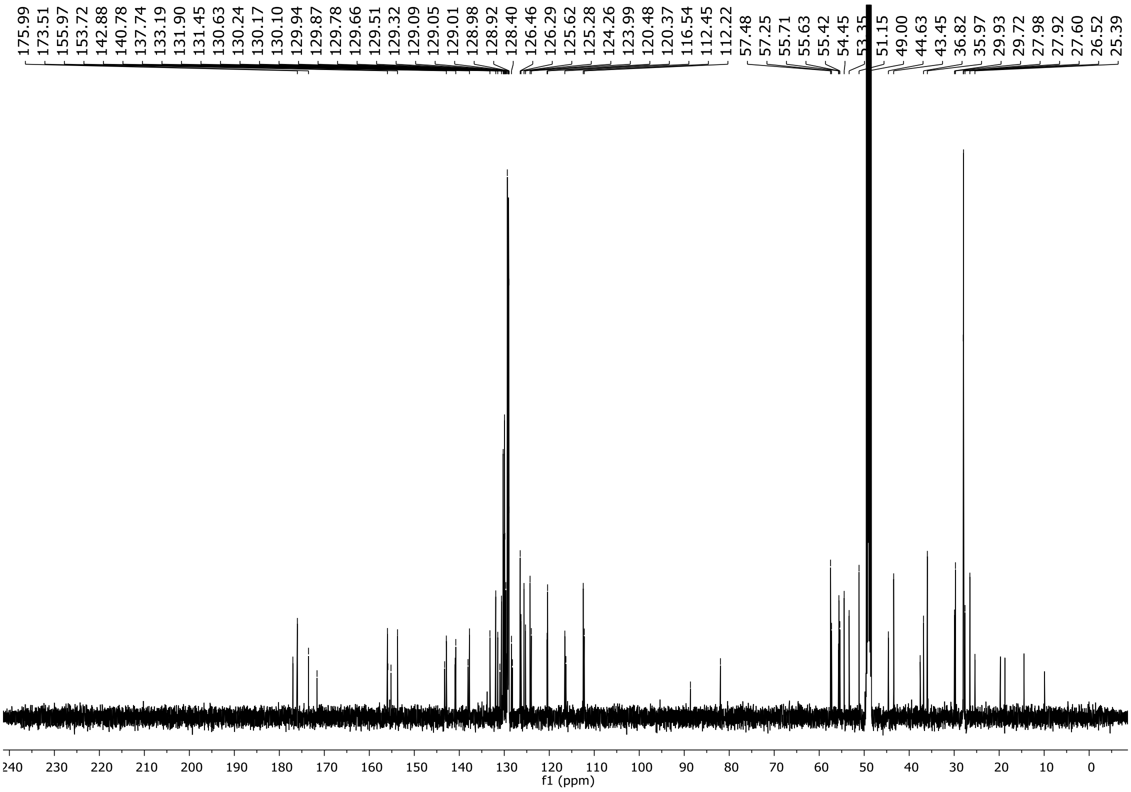

2-(tert-butyl)-8-((diphenylmethylene)amino)-6-fluoro-4-(3-phenylpropyl)-2-azaspiro[4.5]deca-6,9-dien-3-one (3k)

Major isomer A

^{1}H NMR (500 MHz, CD_3OD)

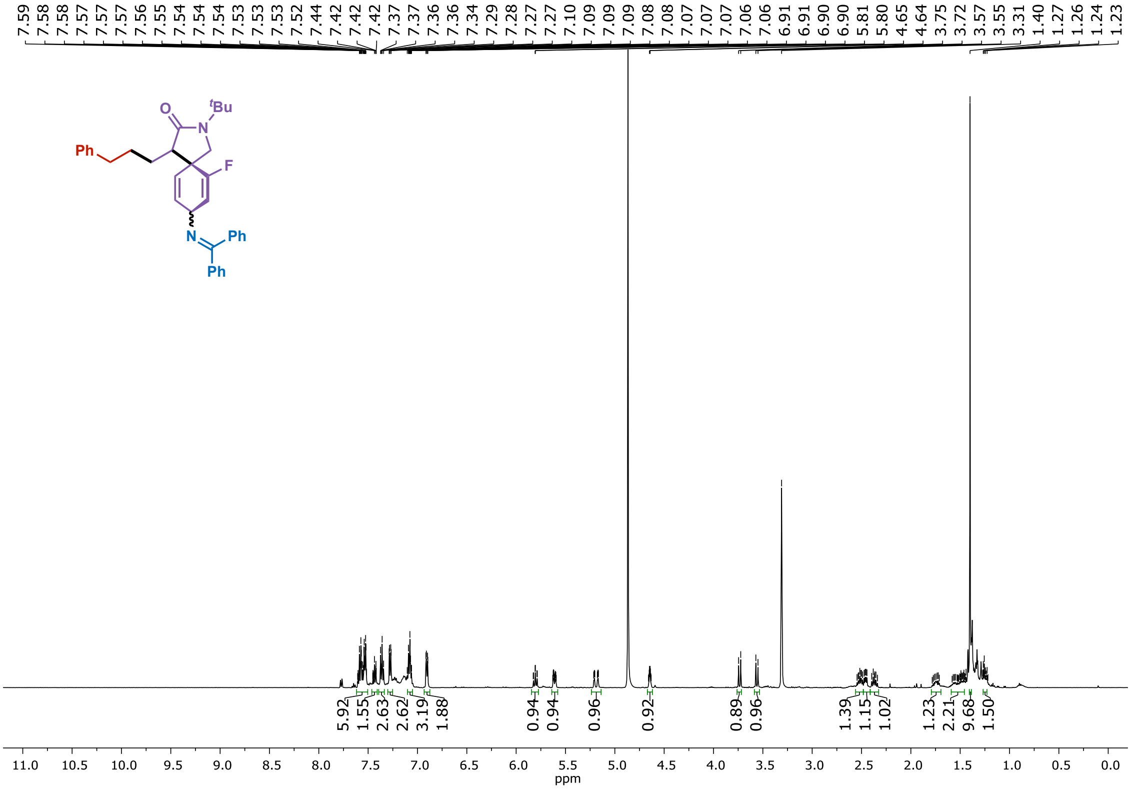

^{13}C NMR (126 MHz, CD_3OD)

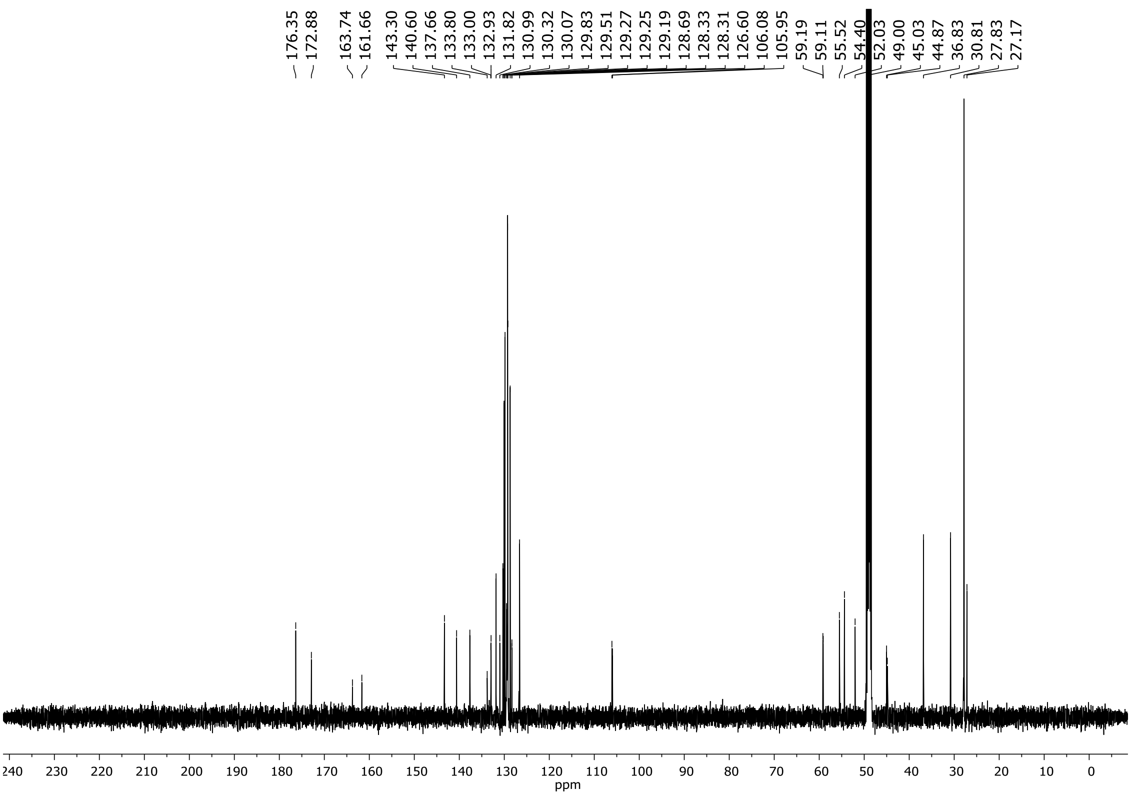

COSY

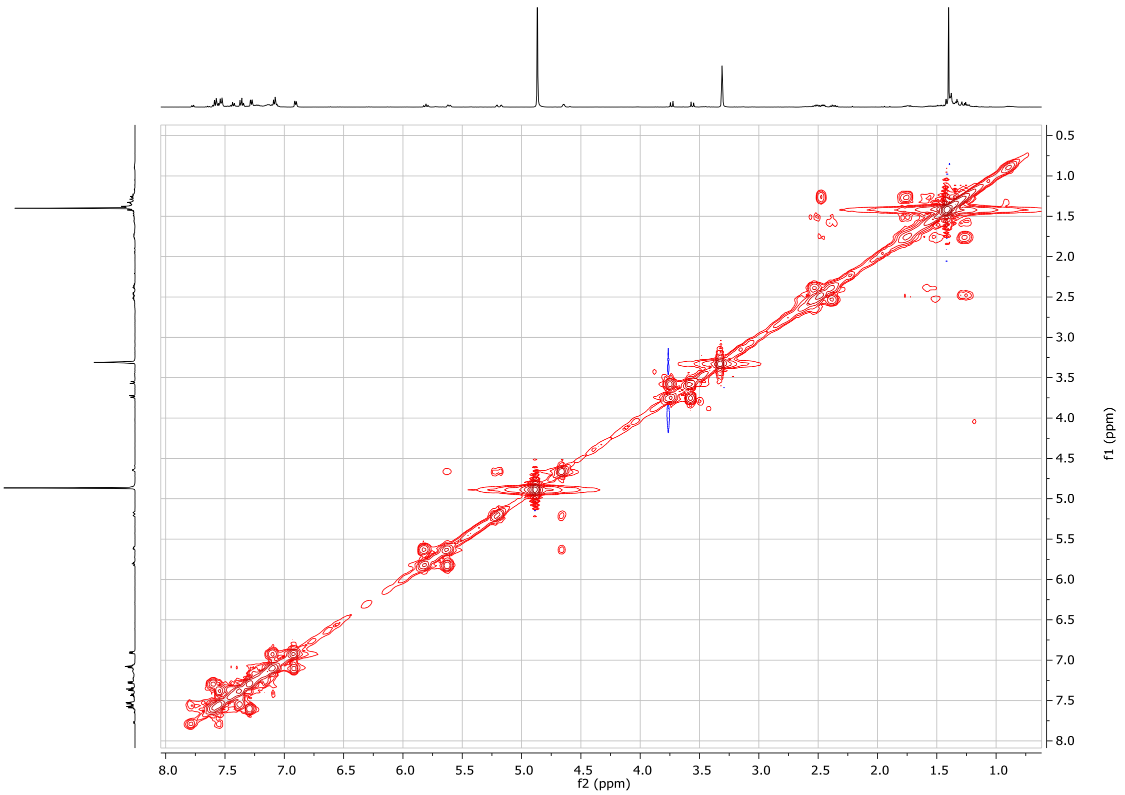

NOESY

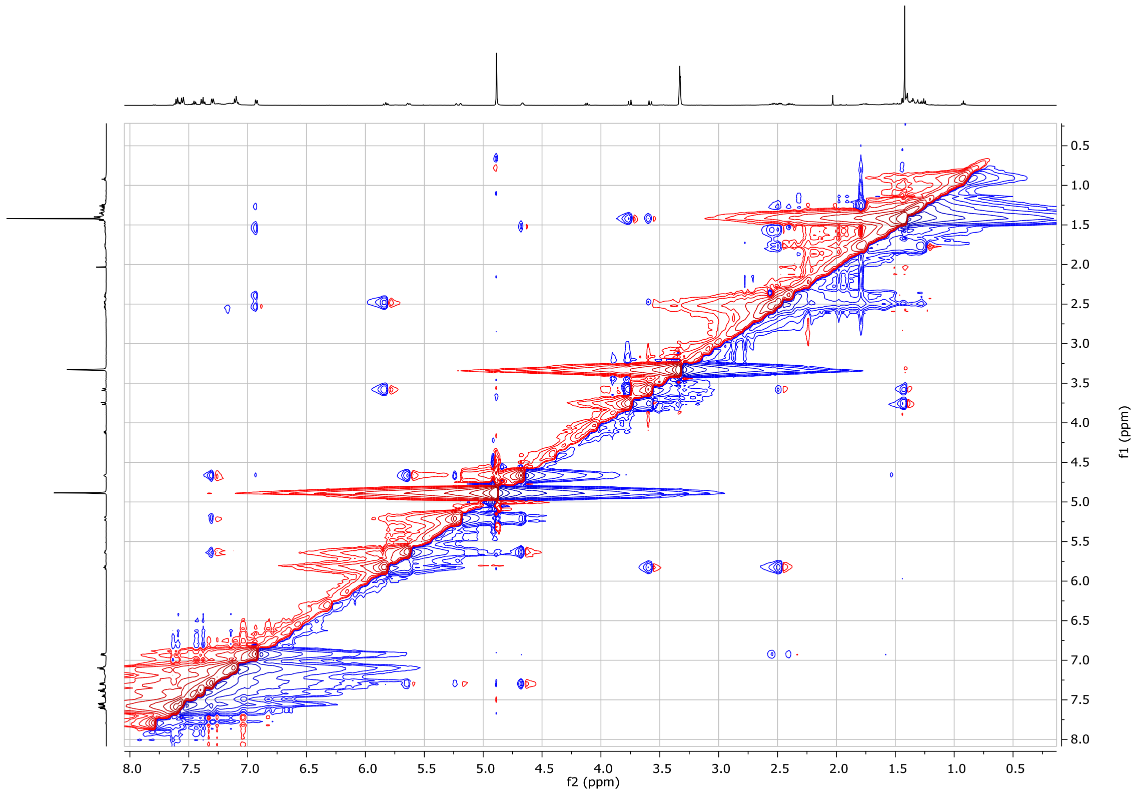

HSQC

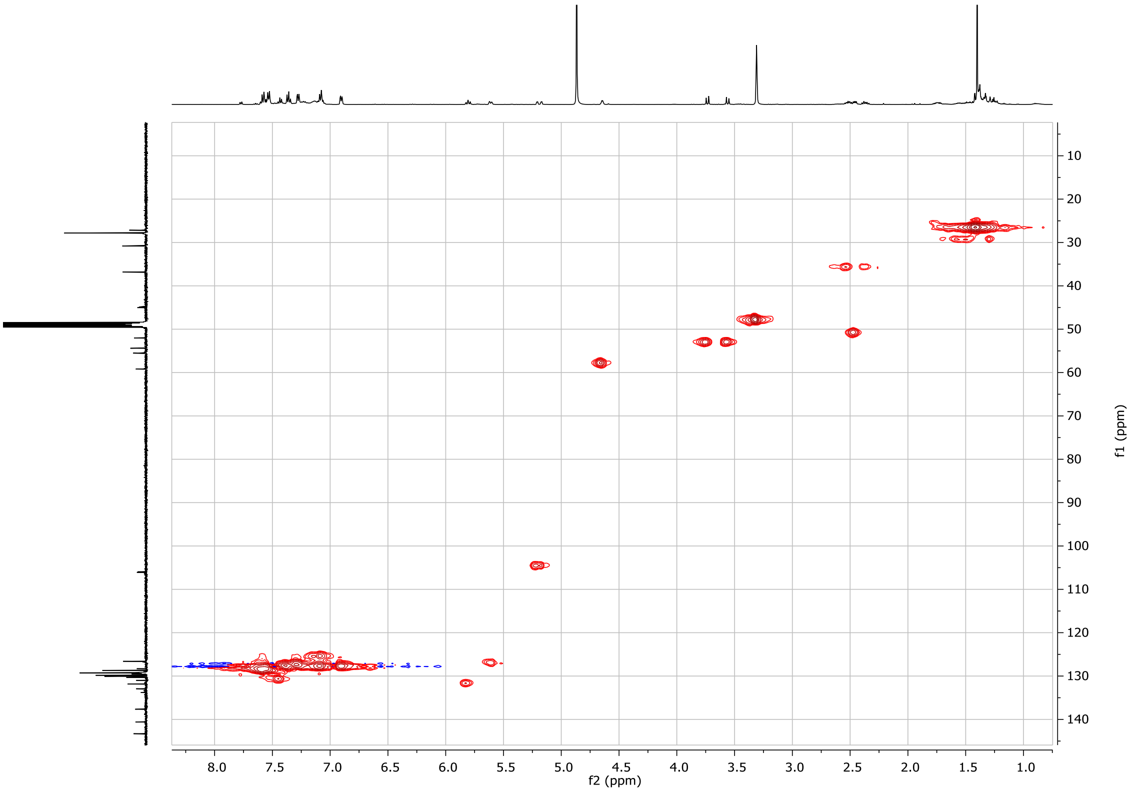

^{19}F NMR (377 MHz, CD_3OD)

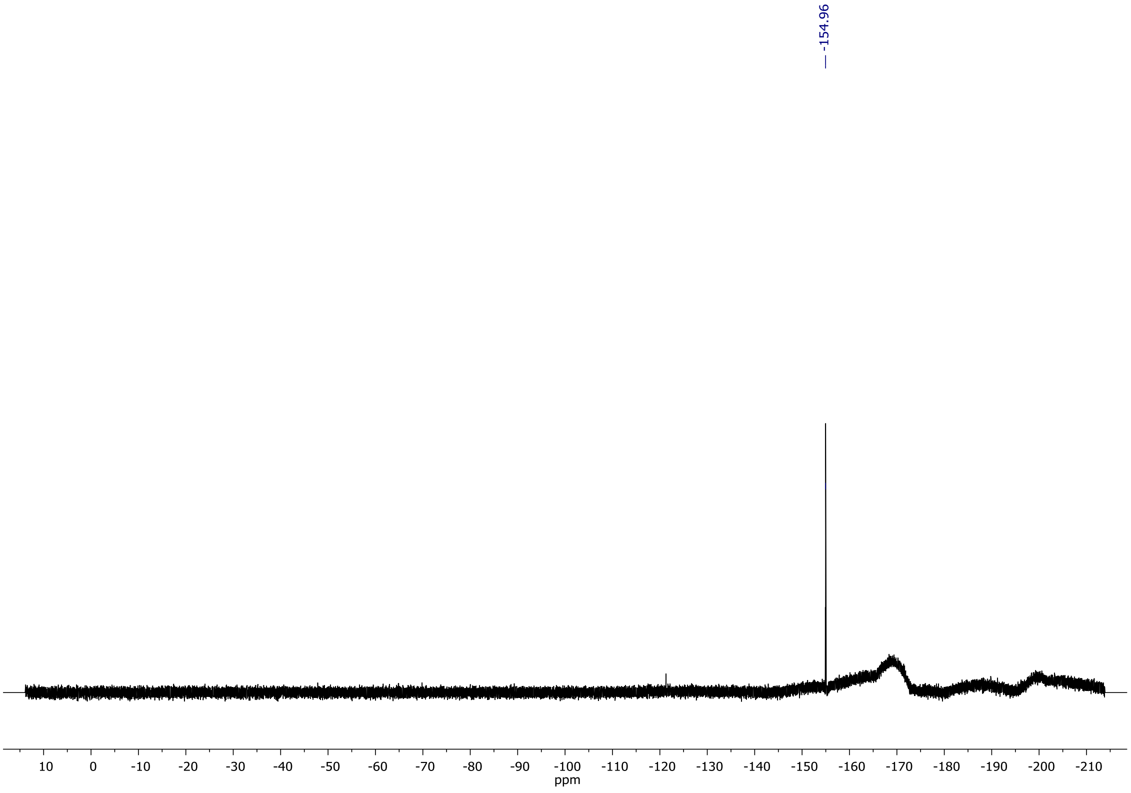

Minor isomer C

^{1}H NMR (500 MHz, CD_3OD)

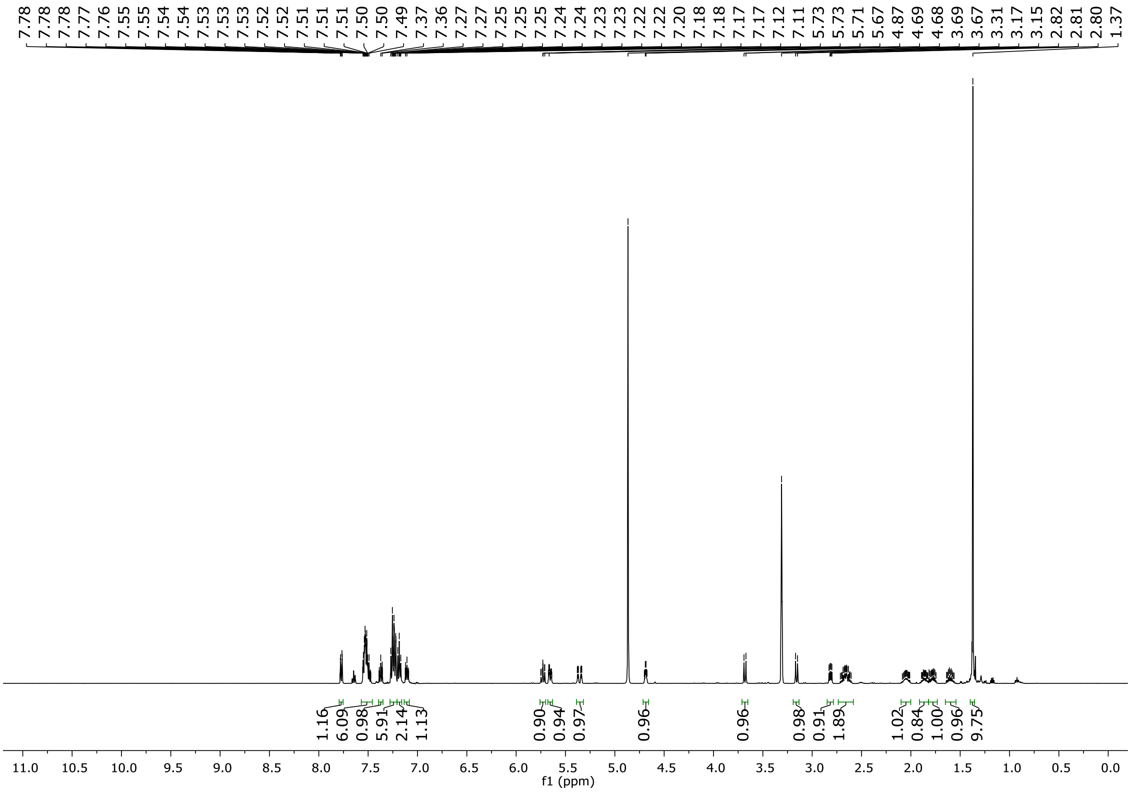

^{13}C NMR (126 MHz, CD_3OD)

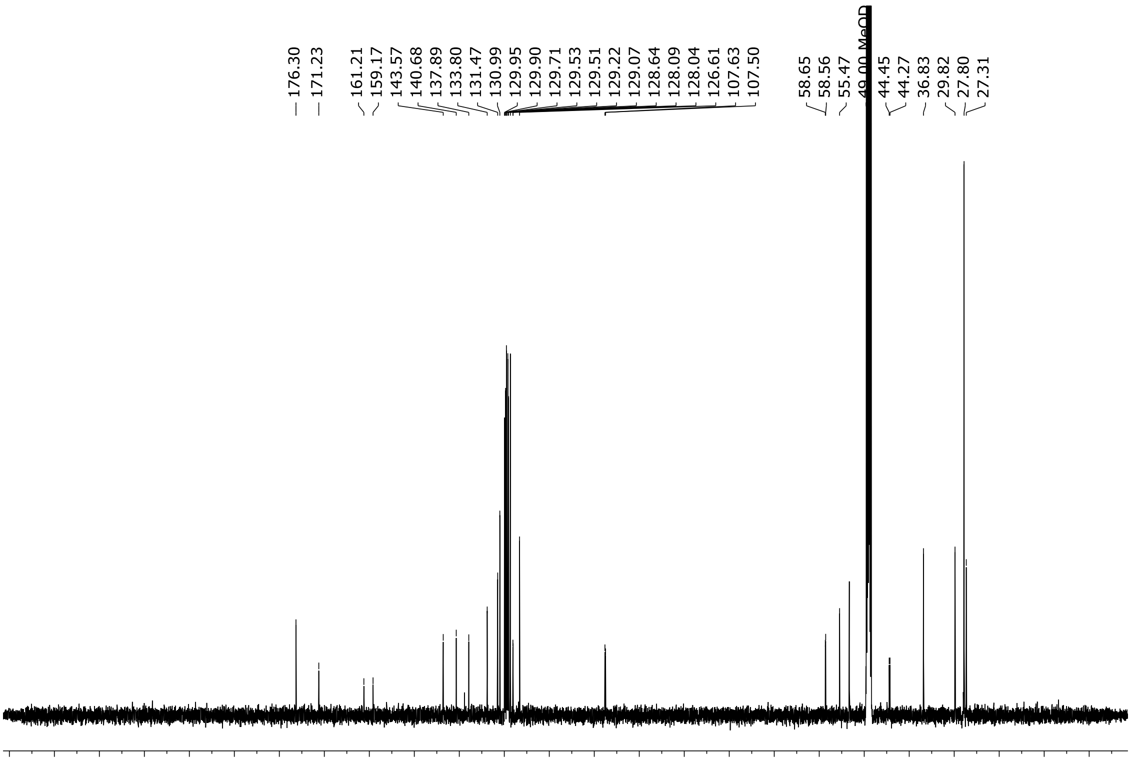

COSY

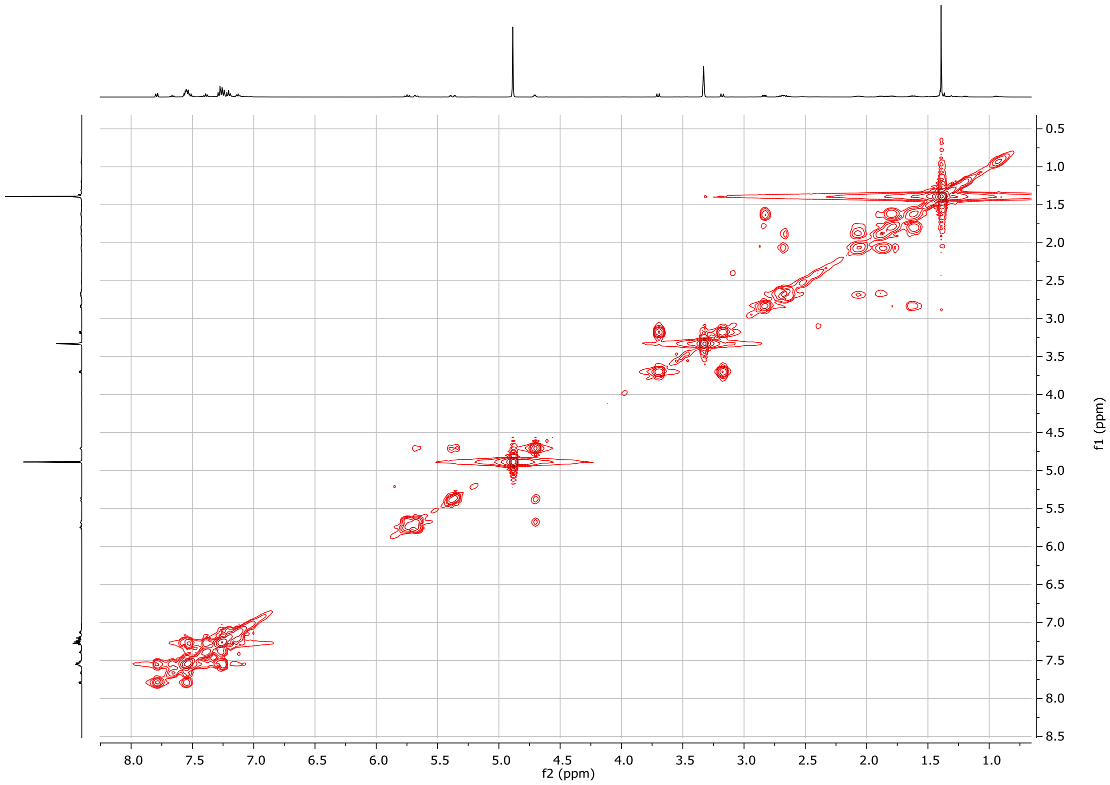

NOESY

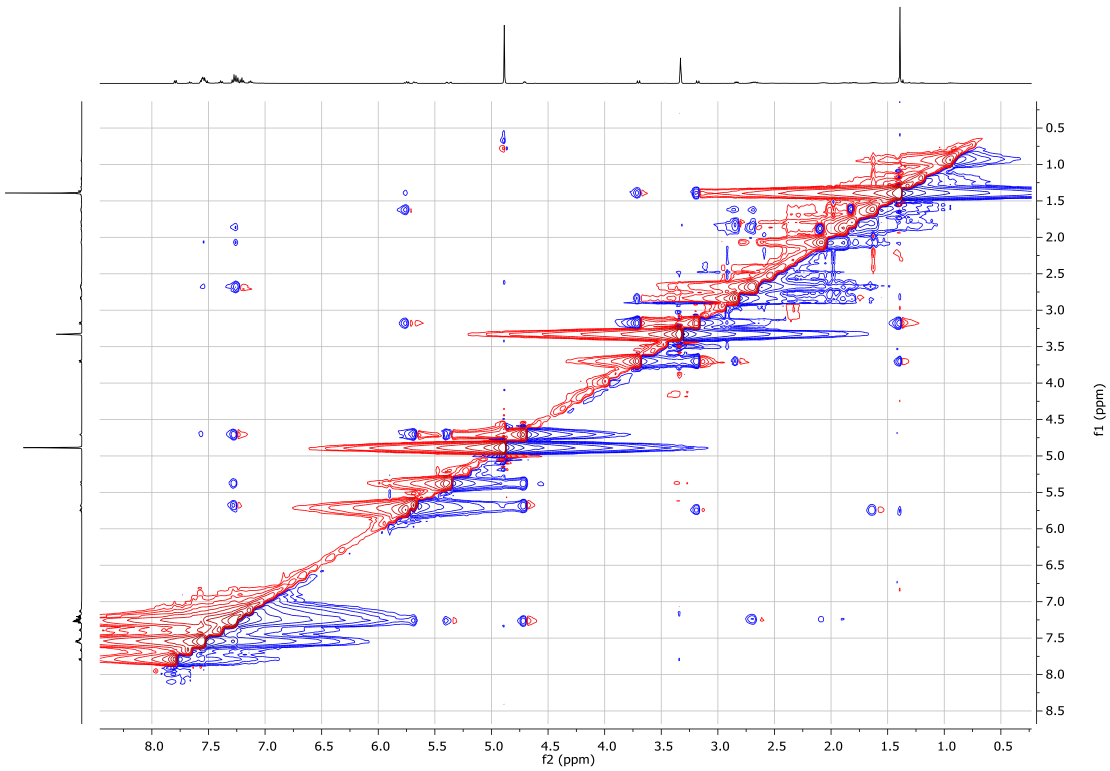

HSQC

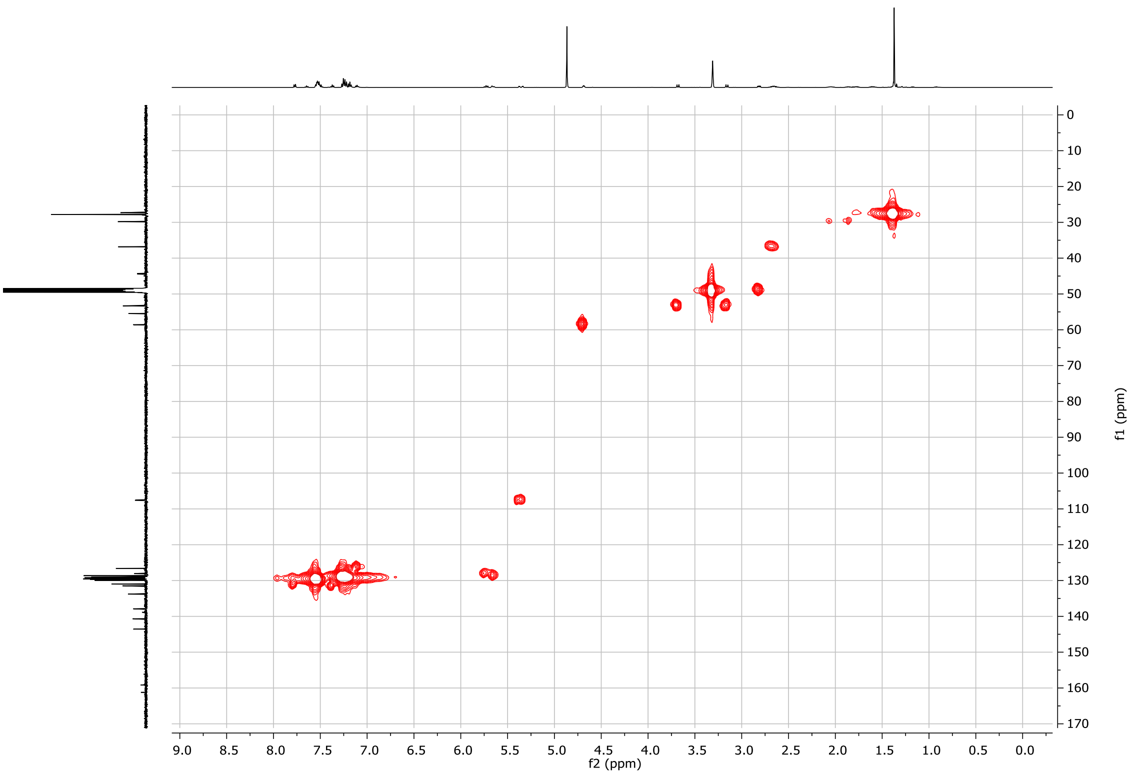

HMBC

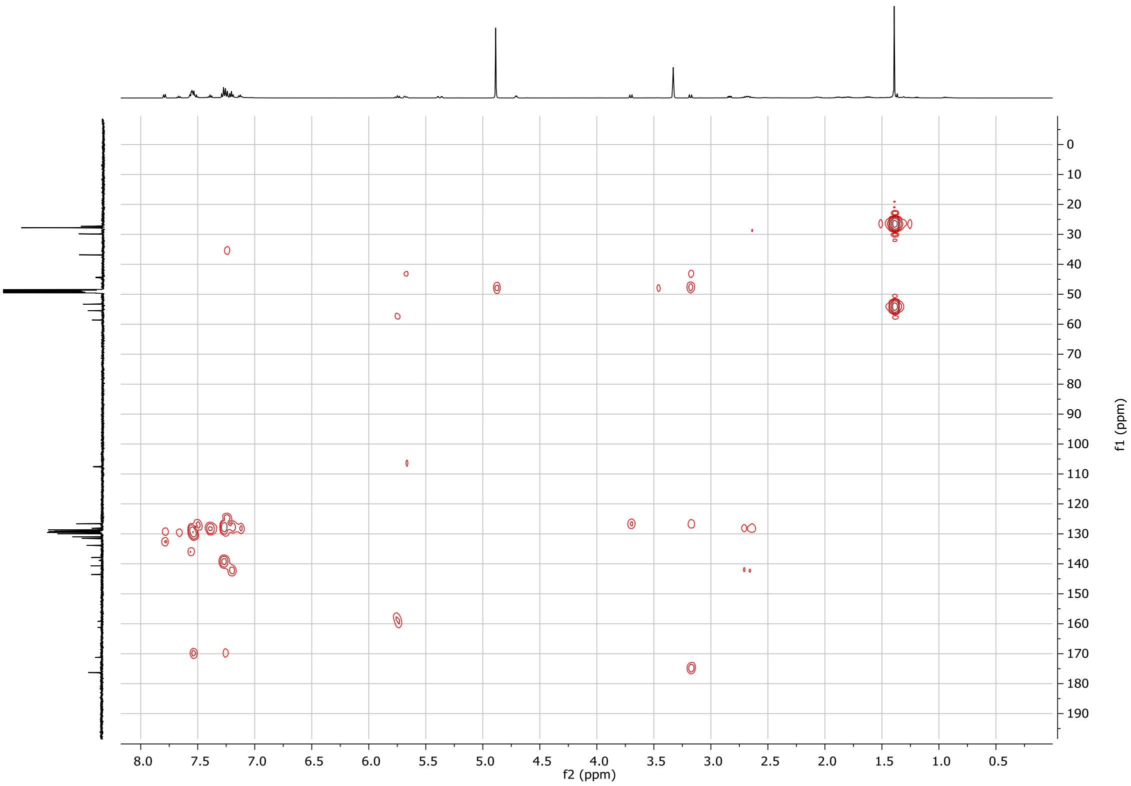

^{19}F NMR (377 MHz, CD_3OD)

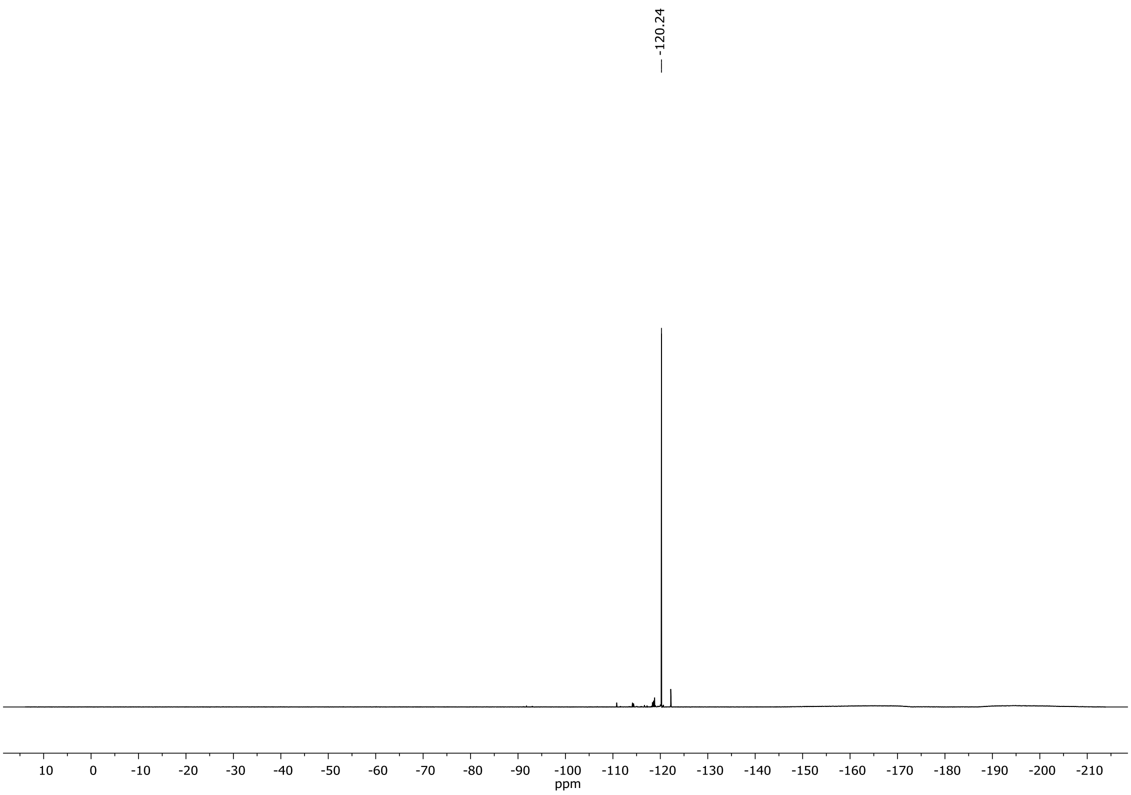

Mixture of minor isomers B and C

^{1}H NMR (500 MHz, CD_3OD)

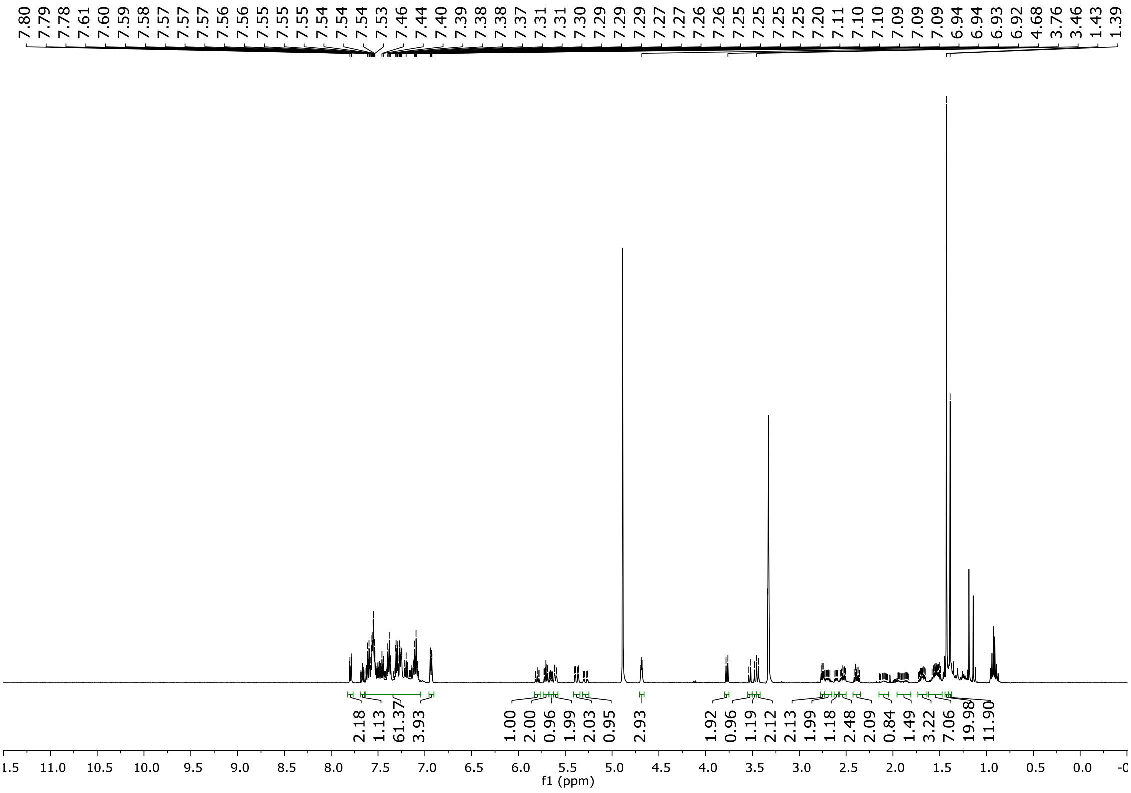

^{13}C NMR (126 MHz, CD_3OD)

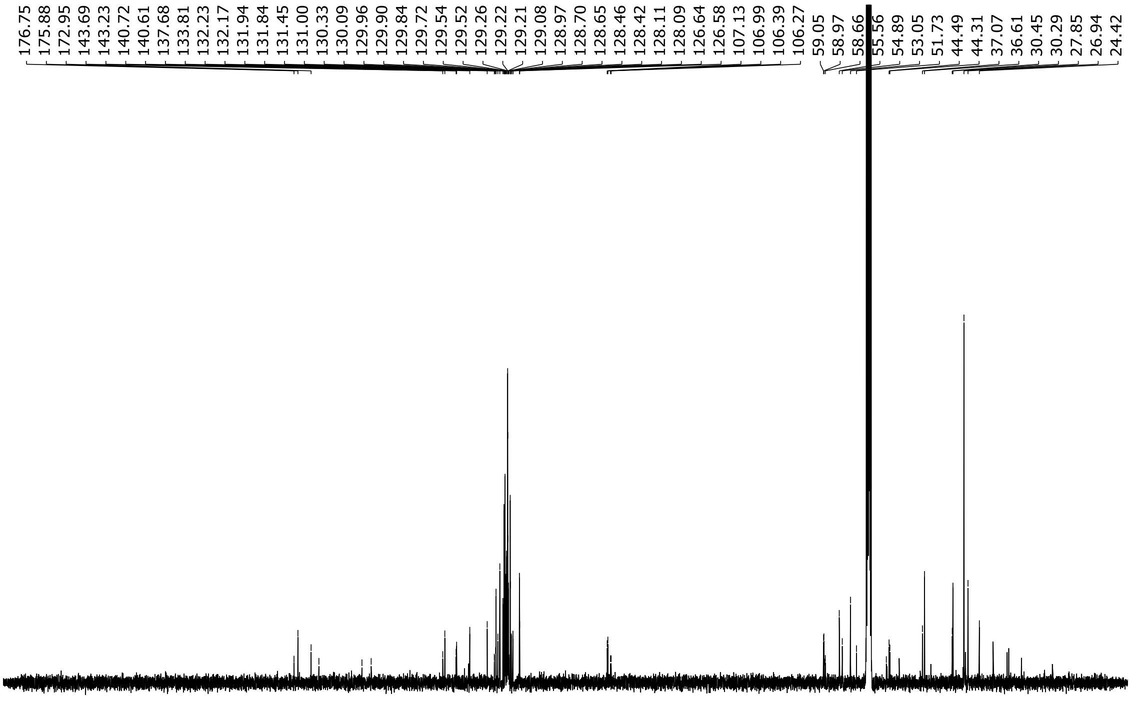

^{19}F NMR (377 MHz, CD_3OD)

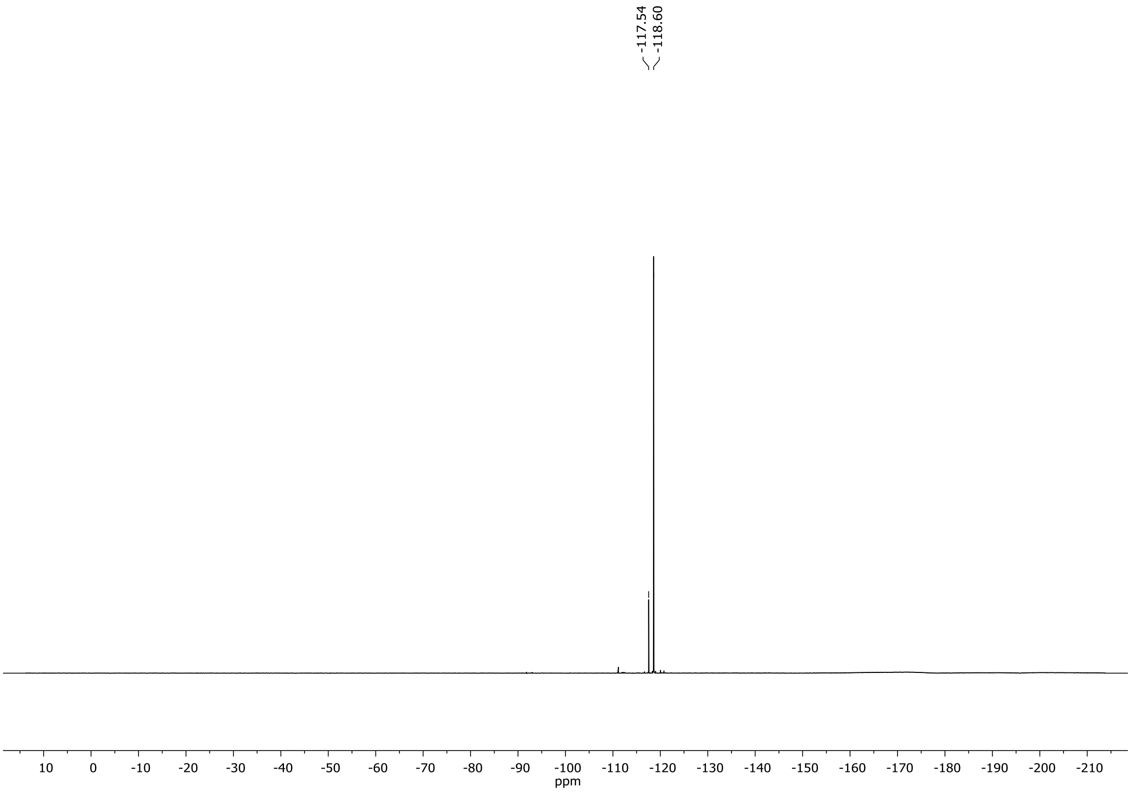

2-(*tert*-butyl)-8-((diphenylmethylene)amino)-6-fluoro-4-(3-phenylpropyl)-2-azaspiro[4.5]deca-6,9-dien-3-one (3l)

^{1}H NMR (500 MHz, CD_3OD)

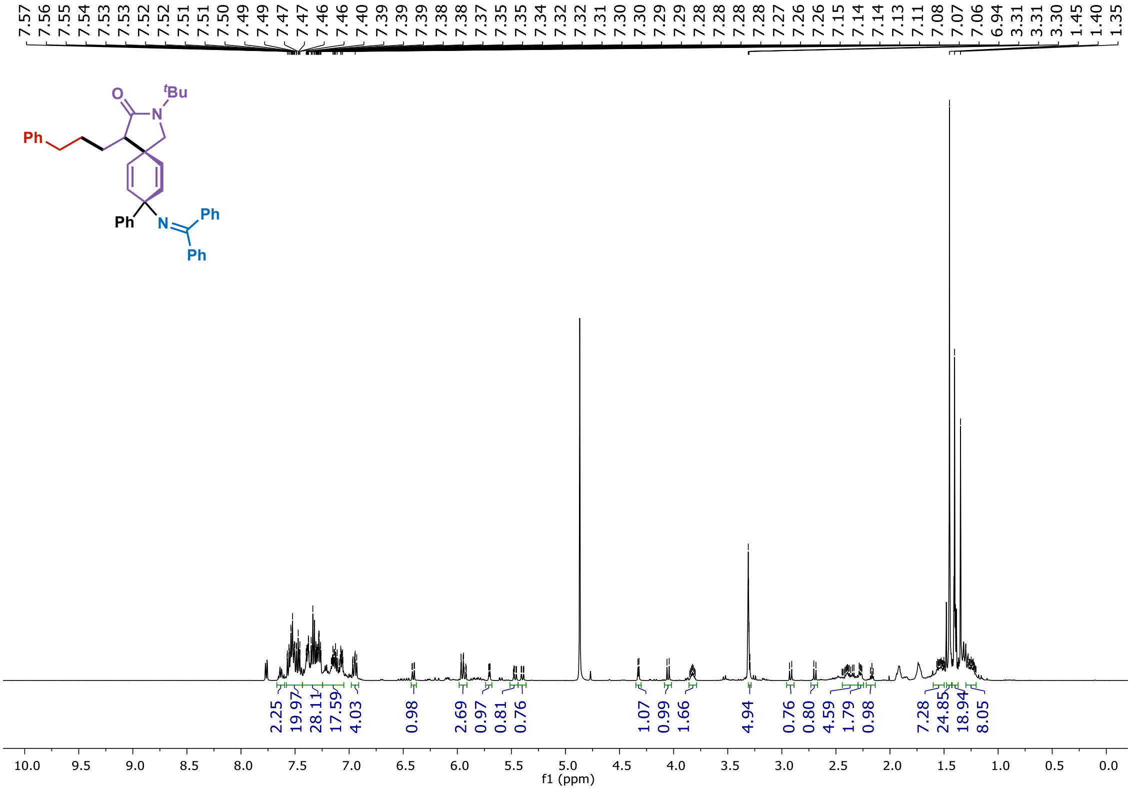

^{13}C NMR (126 MHz, CD_3OD)

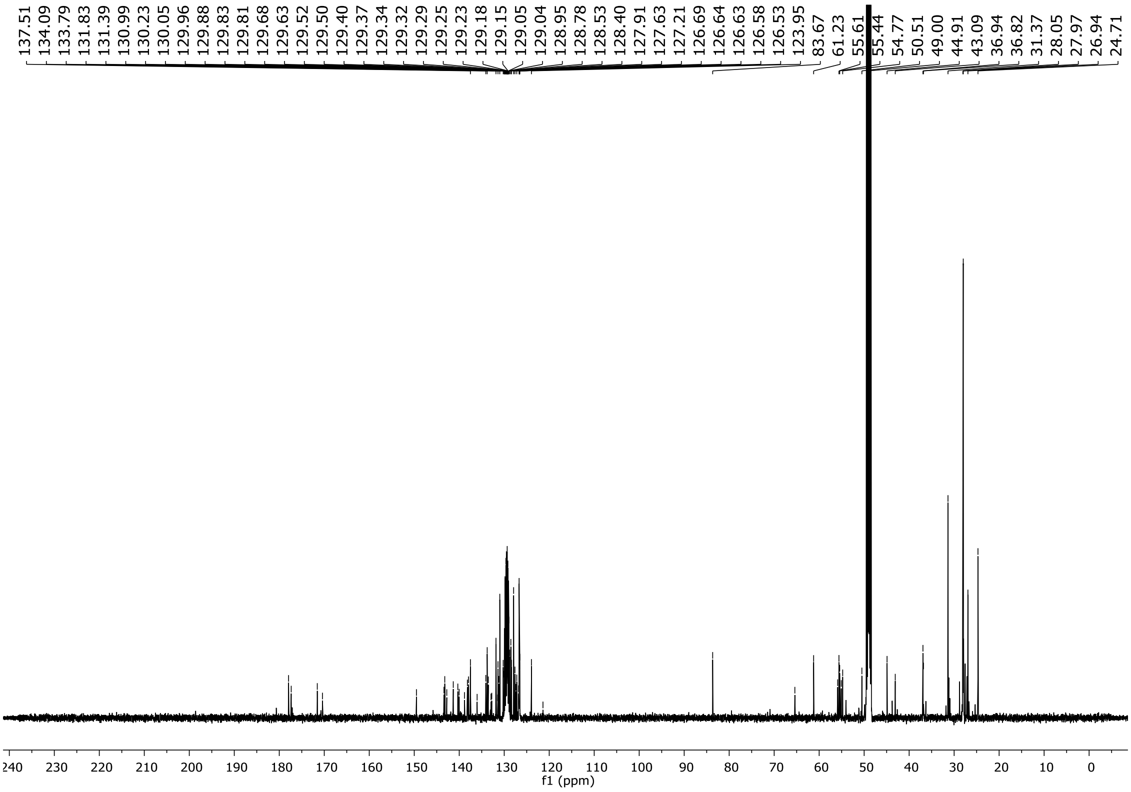

2-(*tert*-Butyl)-8-((diphenylmethylene)amino)-4-(2-oxo-2-phenylethyl)-2-azaspiro[4.5]deca-6,9-dien-3-one (3m, 8e)

^{1}H NMR (400 MHz, CD_3OD)

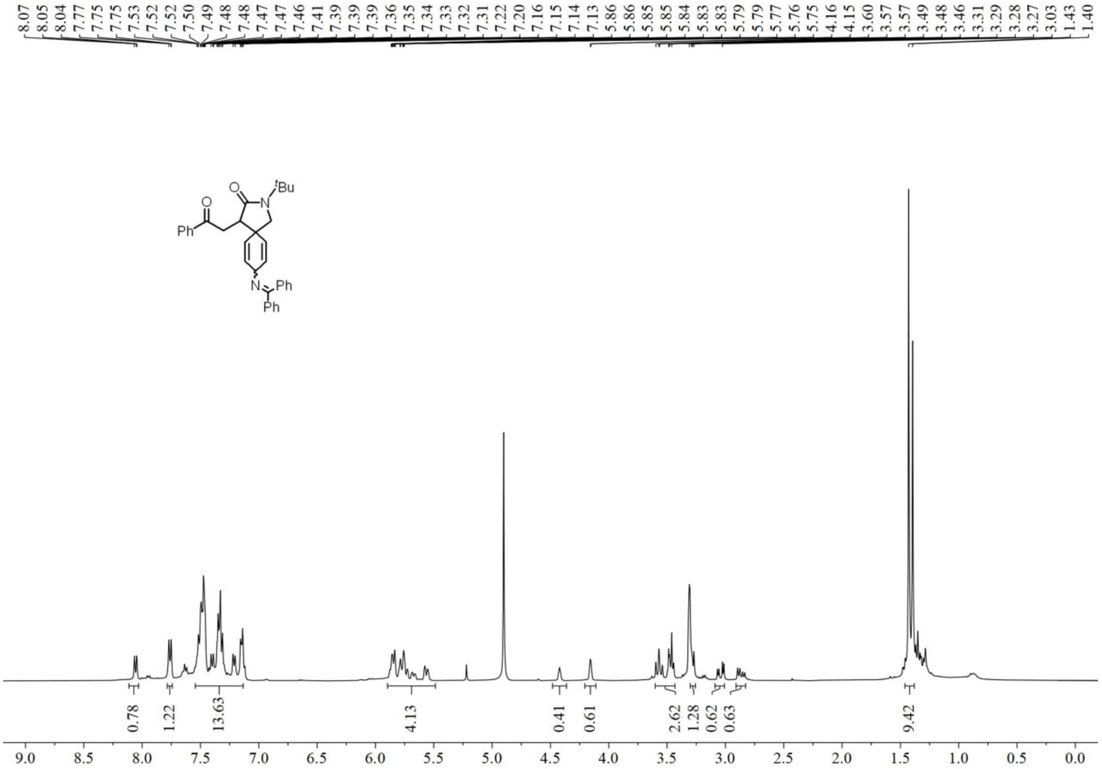

^{13}C NMR (101 MHz, CD_3OD)

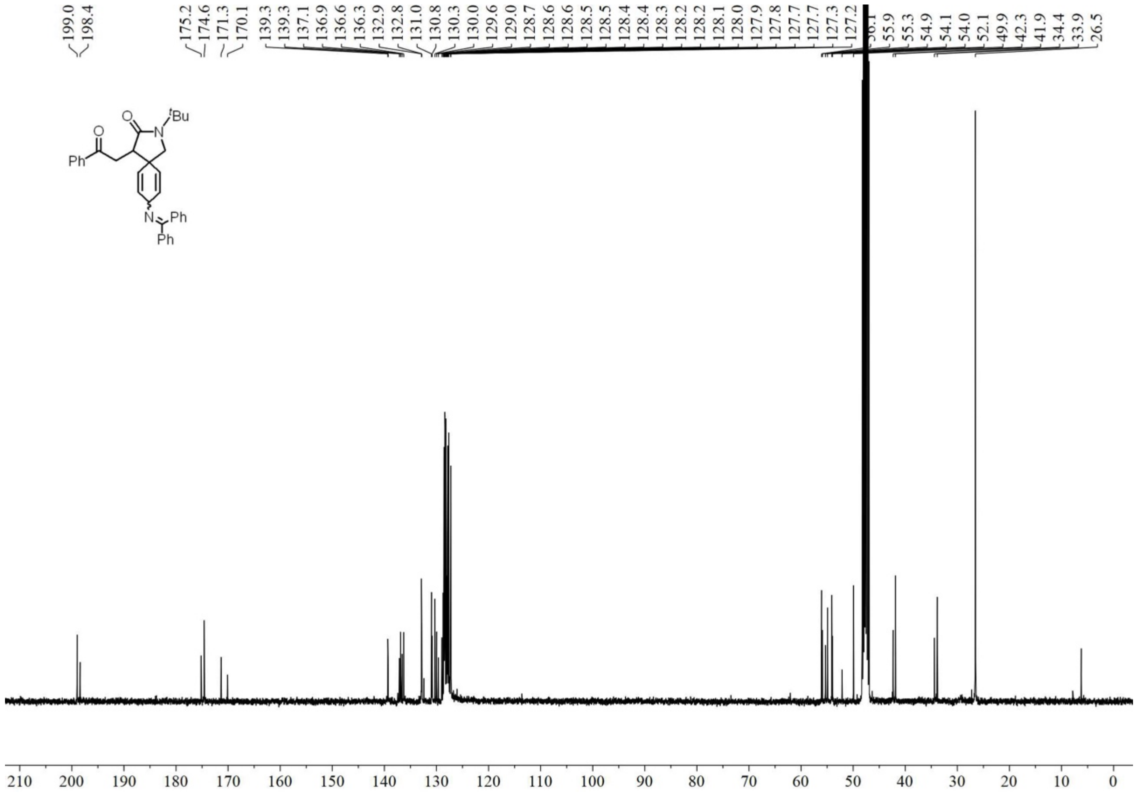

2-(*tert*-Butyl)-8-((diphenylmethylene)amino)-4-(2-mesityl-2-oxoethyl)-2-azaspiro[4.5]deca-6,9-dien-3-one (3n)

^{1}H NMR (400 MHz, CD_3OD)

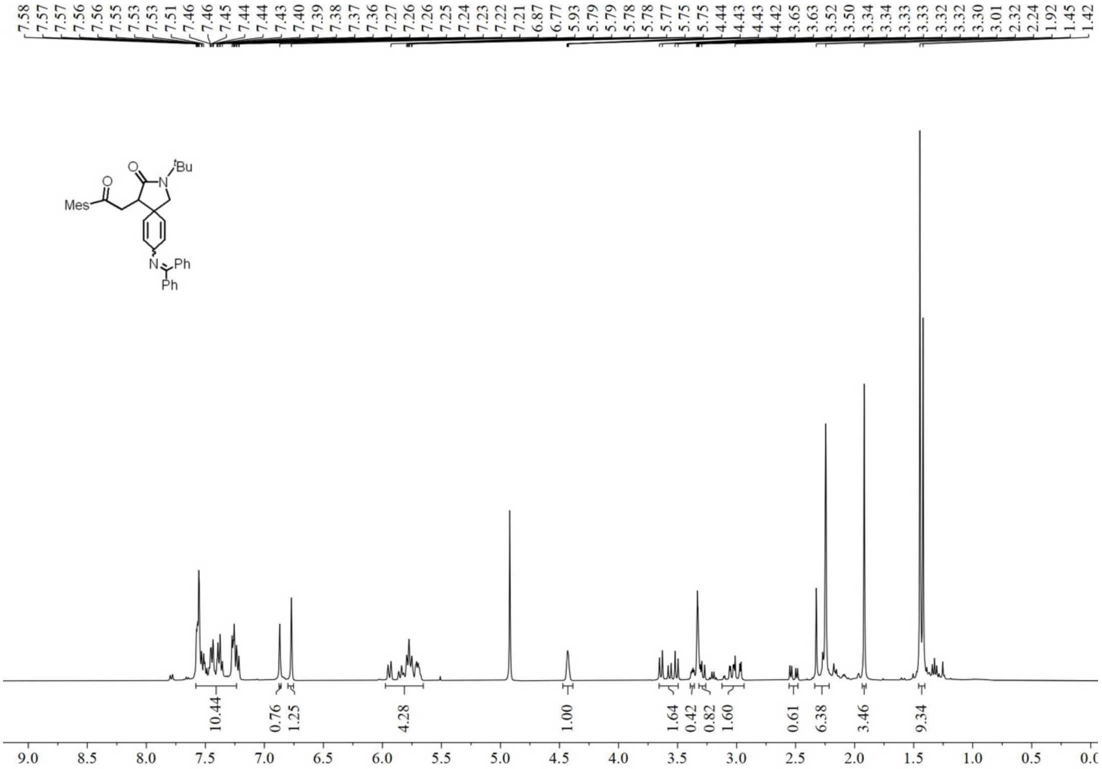

^{13}C NMR (101 MHz, CD_3OD)

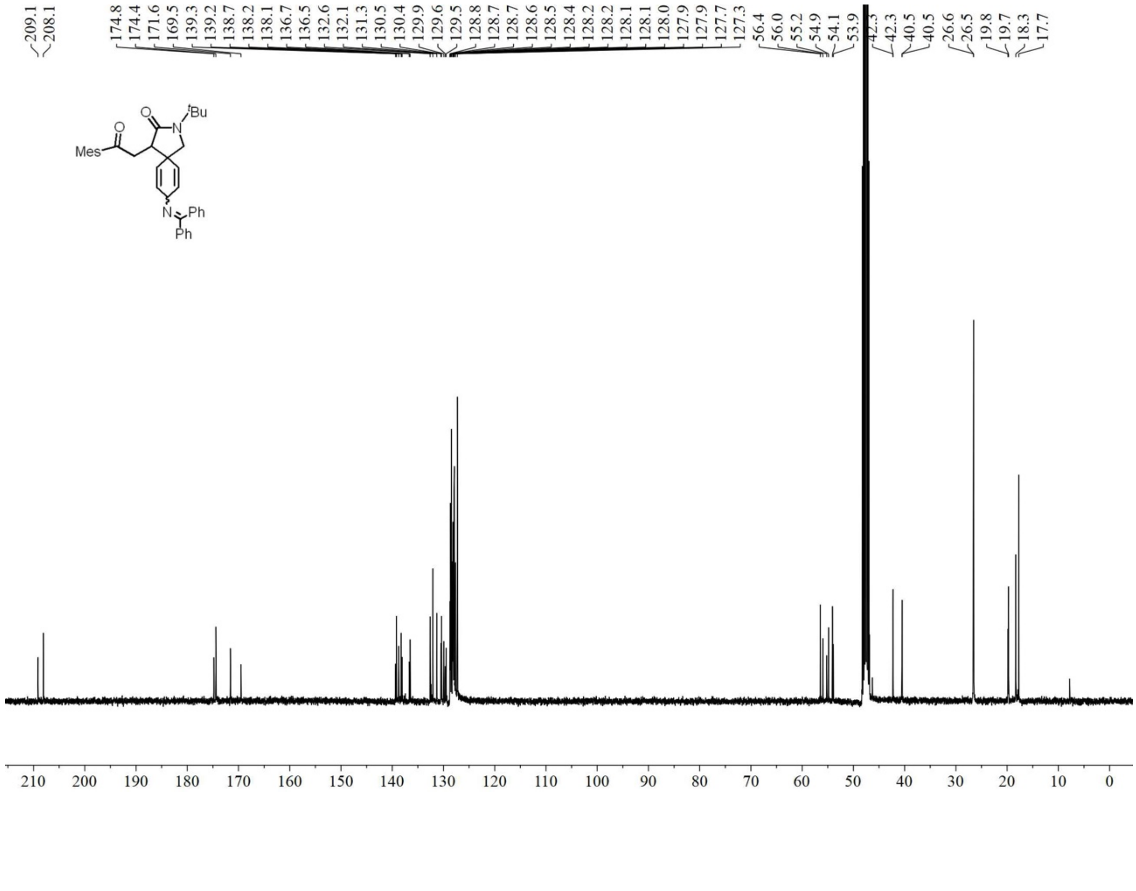

Methyl 2-(2-(*tert*-butyl)-8-((diphenylmethylene)amino)-3-oxo-2-azaspiro[4.5]deca-6,9-dien-4-yl)acetate (3o)

^{1}H NMR (400 MHz, CD_3OD)

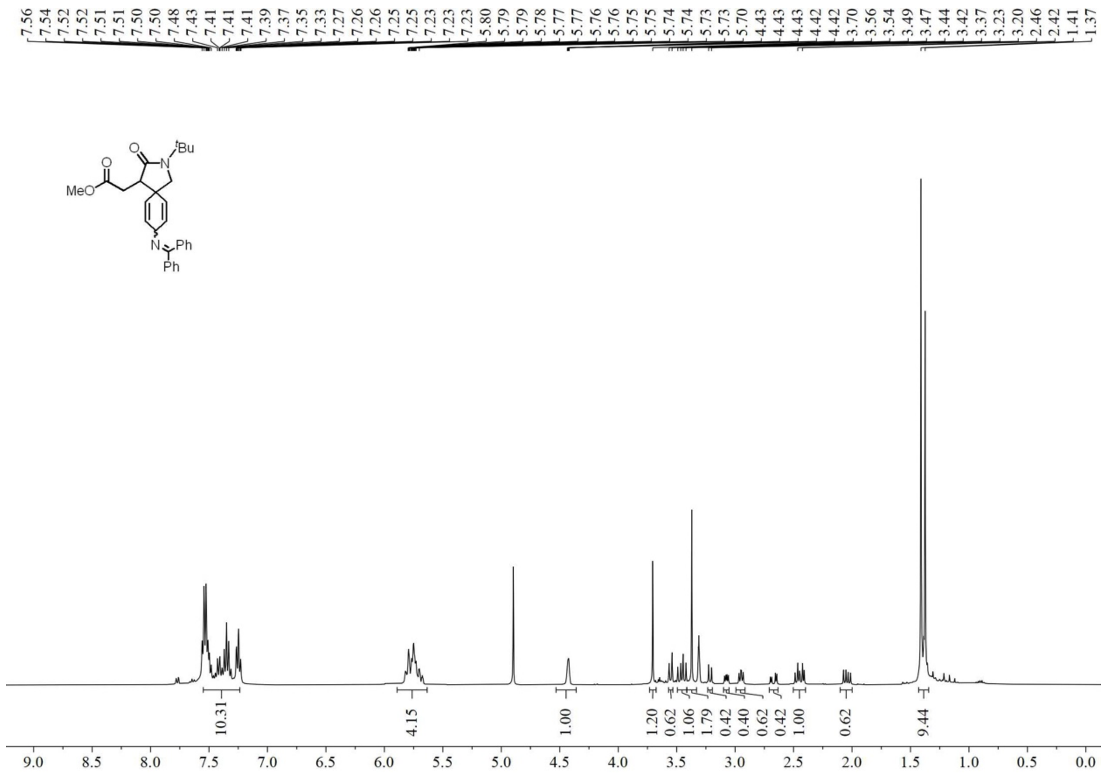

^{13}C NMR (101 MHz, CD_3OD)

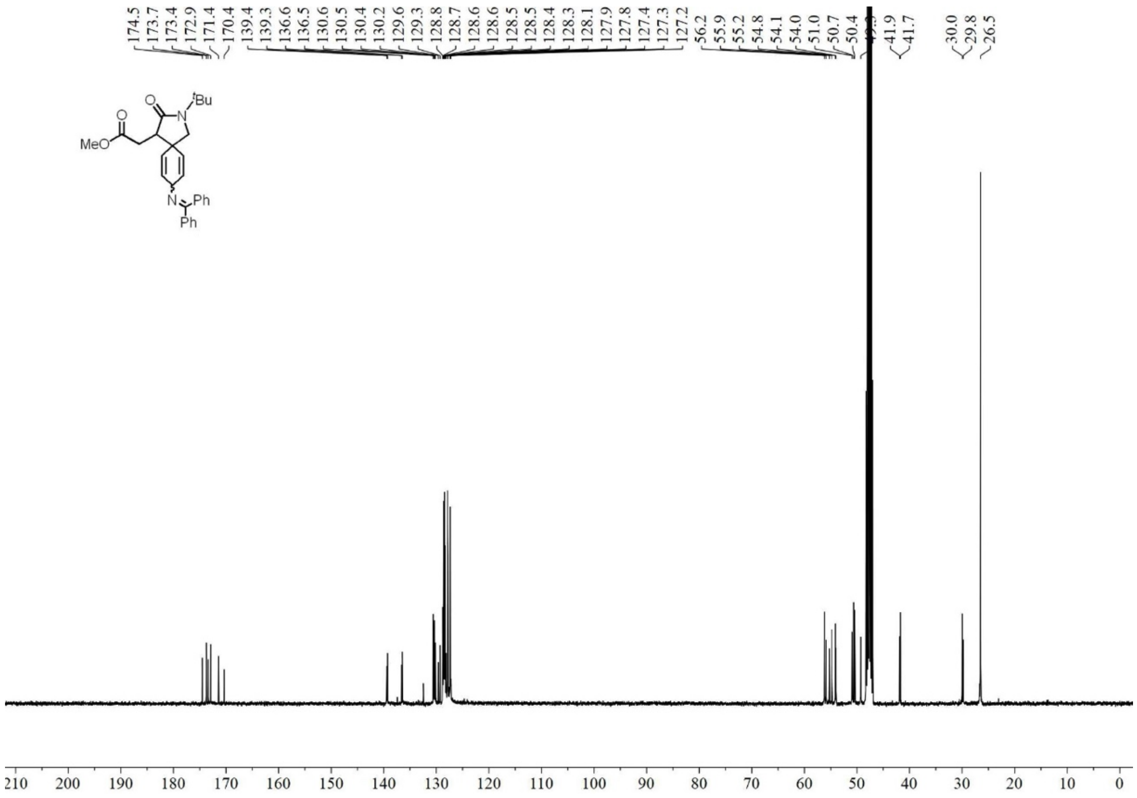

Ethyl 2-(2-(*tert*-butyl)-8-((diphenylmethylene)amino)-3-oxo-2-azaspiro[4.5]deca-6,9-dien-4-yl)acetate (3p)

^{1}H NMR (500 MHz CD_3CN)

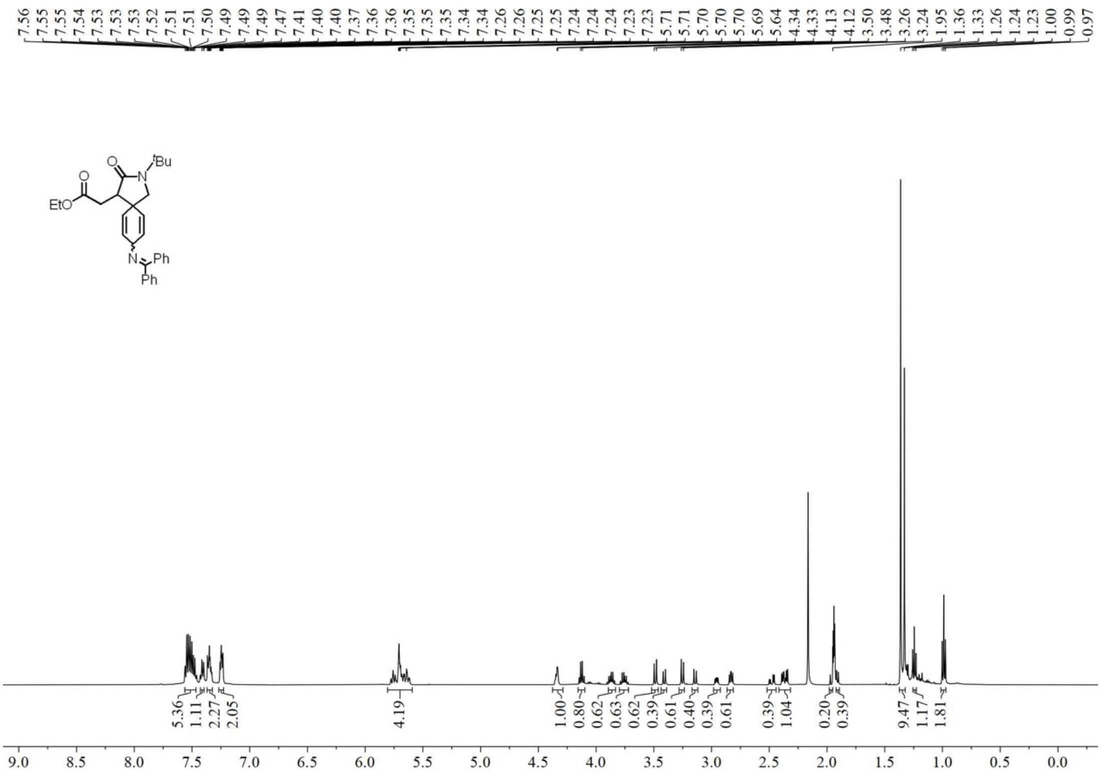

^{13}C NMR (126 MHz, CD_3CN)

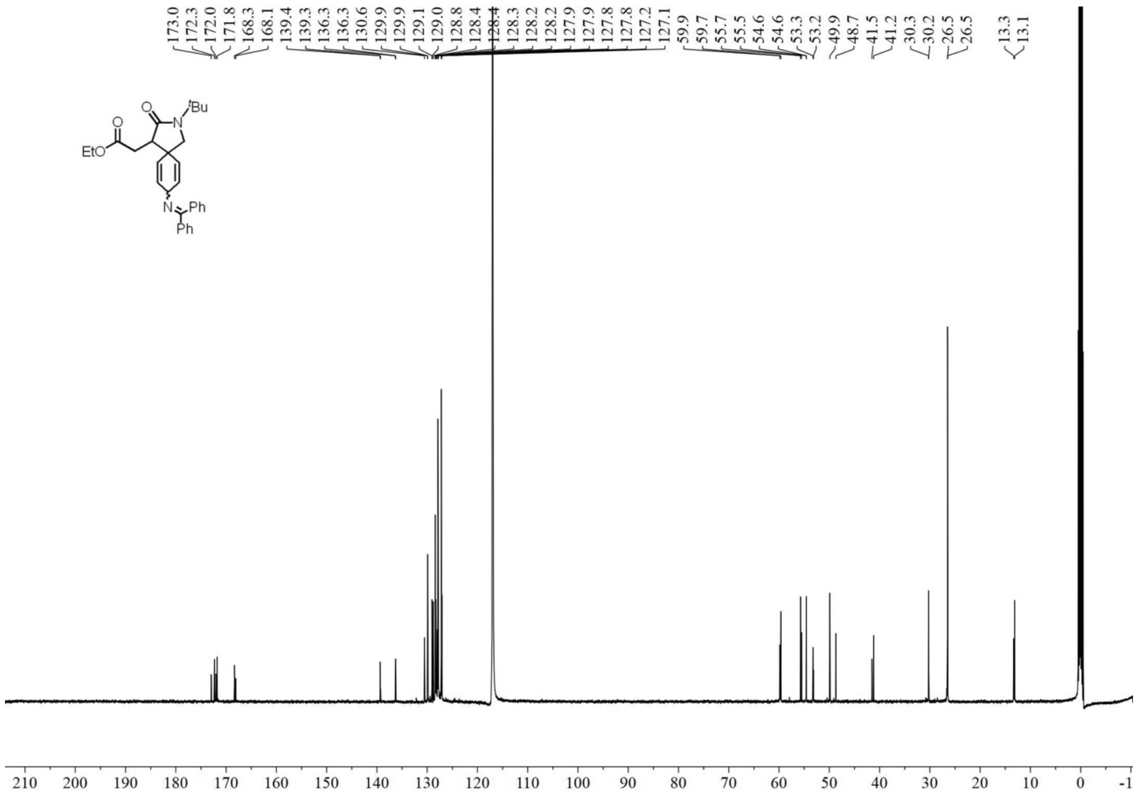

Methyl 2-(2-(*tert*-butyl)-8-((diphenylmethylene)amino)-7,9-dimethoxy-3-oxo-2-azaspiro[4.5]deca-6,9-dien-4-yl)acetate (3q)

^{1}H NMR (400 MHz, CD_3OD)

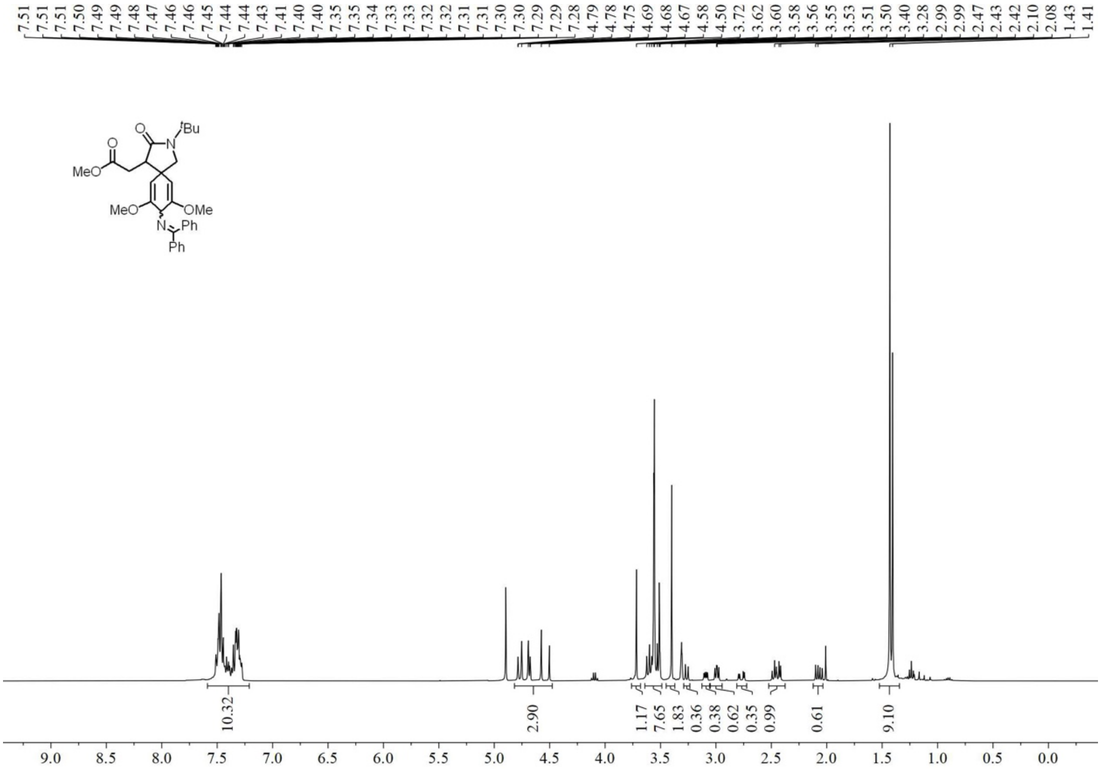

^{13}C NMR (101 MHz, CD_3OD)

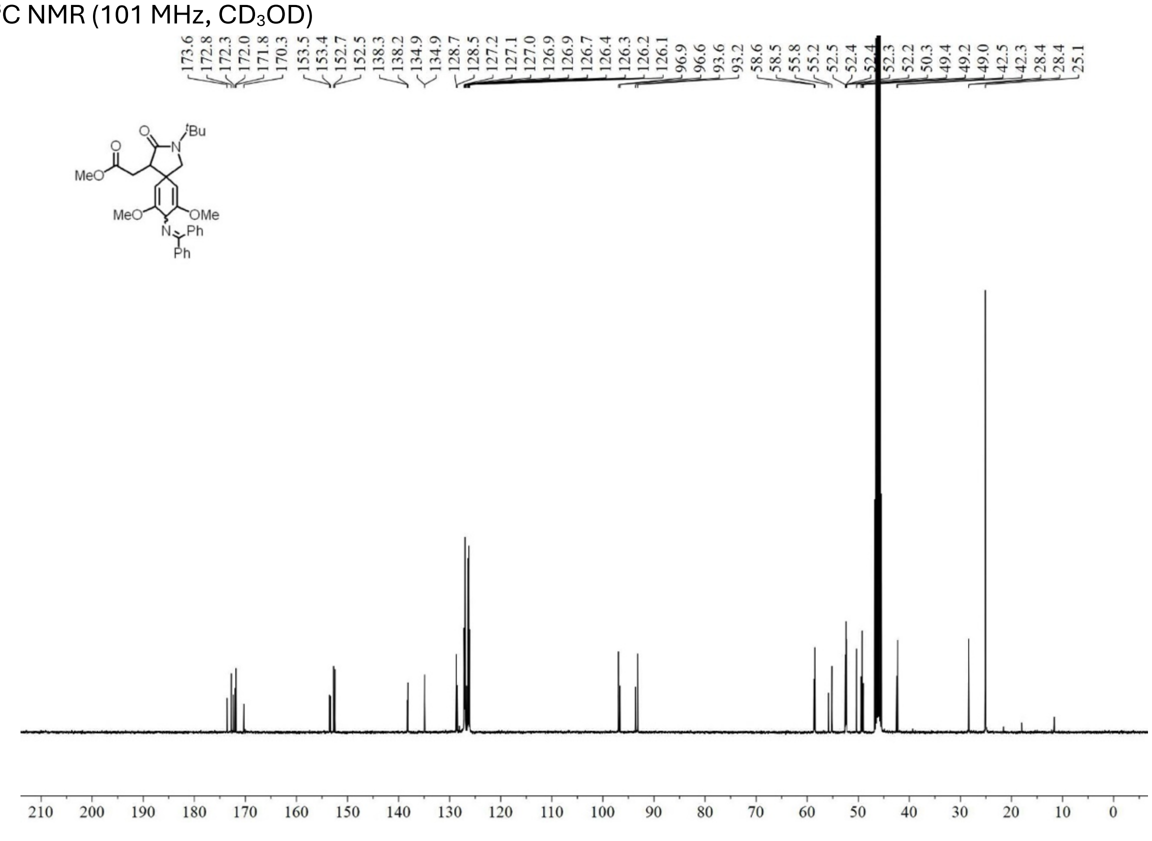

Methyl 2-(2-(*tert*-butyl)-8-((diphenylmethylene)amino)-7,9-dimethyl-3-oxo-2-azaspiro[4.5]deca-6,9-dien-4-yl)acetate (3r).

^{1}H NMR (500 MHz, CD_3OD)

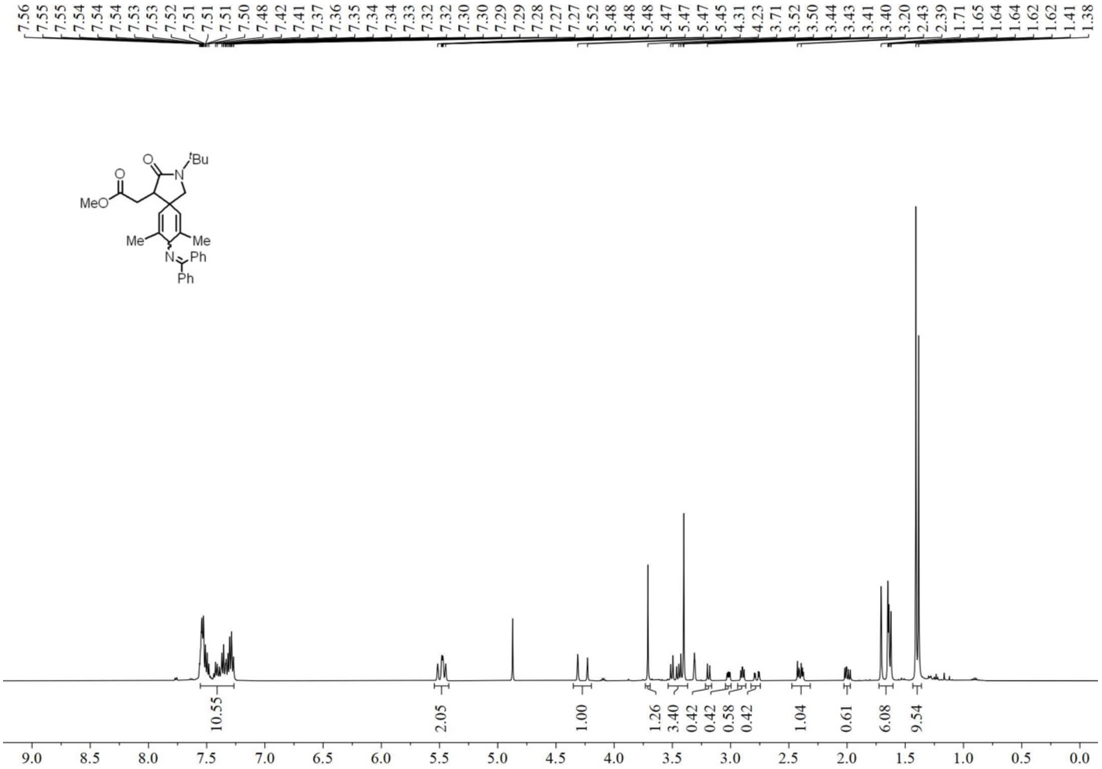

^{13}C NMR (126 MHz, CD_3OD)

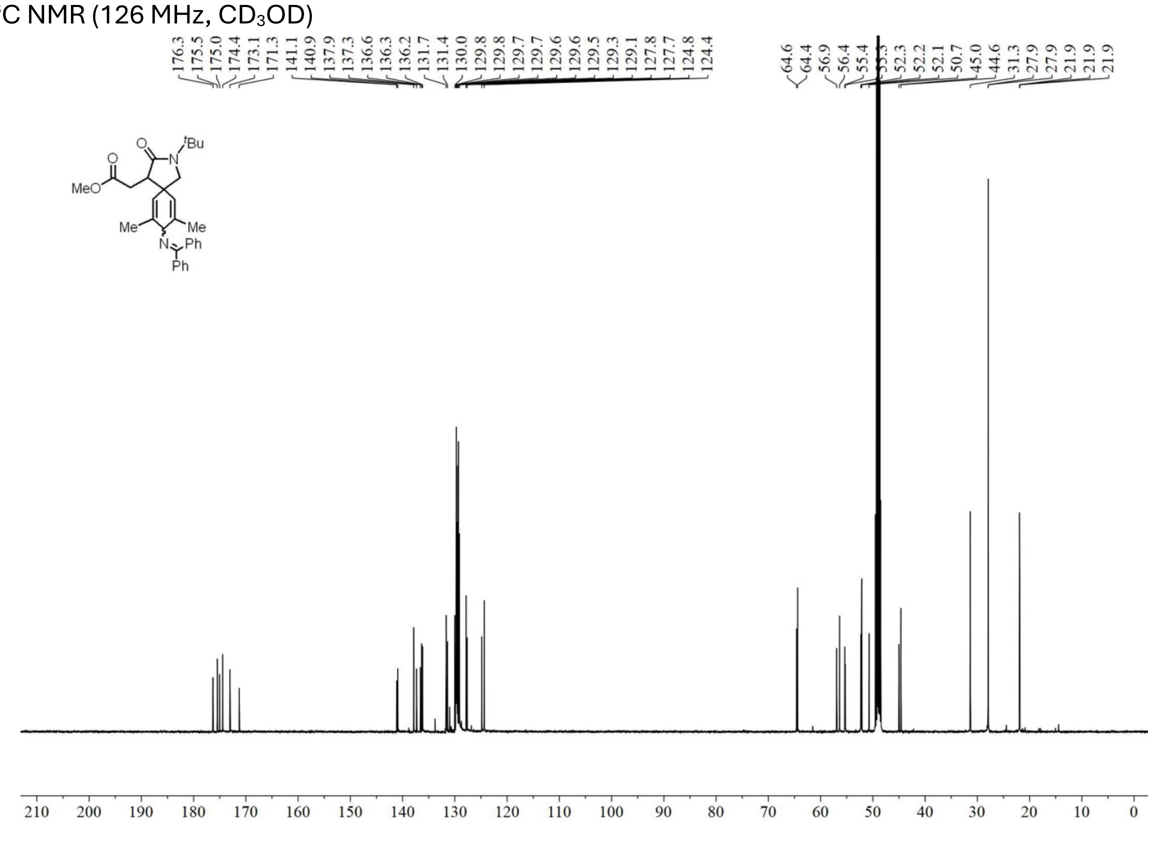

Methyl 2-(2-(*tert*-butyl)-7,9-dichloro-8-((diphenylmethylene)amino)-3-oxo-2-azaspiro[4.5]deca-6,9-dien-4-yl)acetate (3s)

^{1}H NMR (400 MHz, CD_3OD)

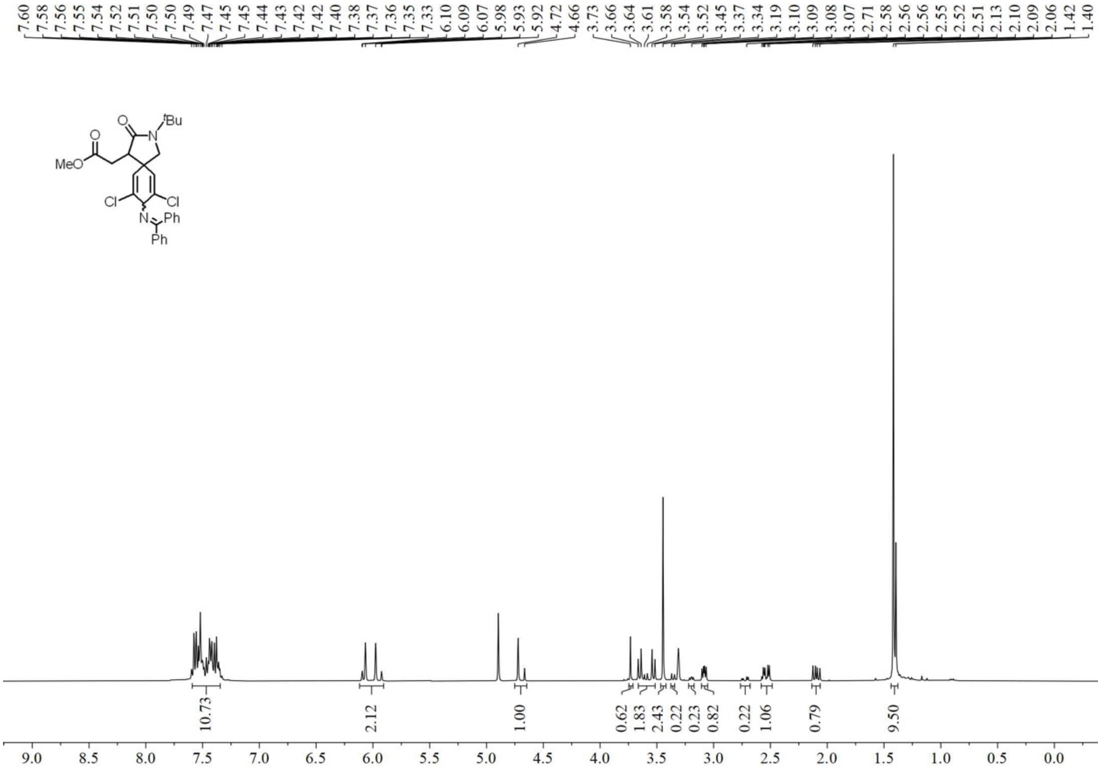

^{13}C NMR (101 MHz, CD_3OD)

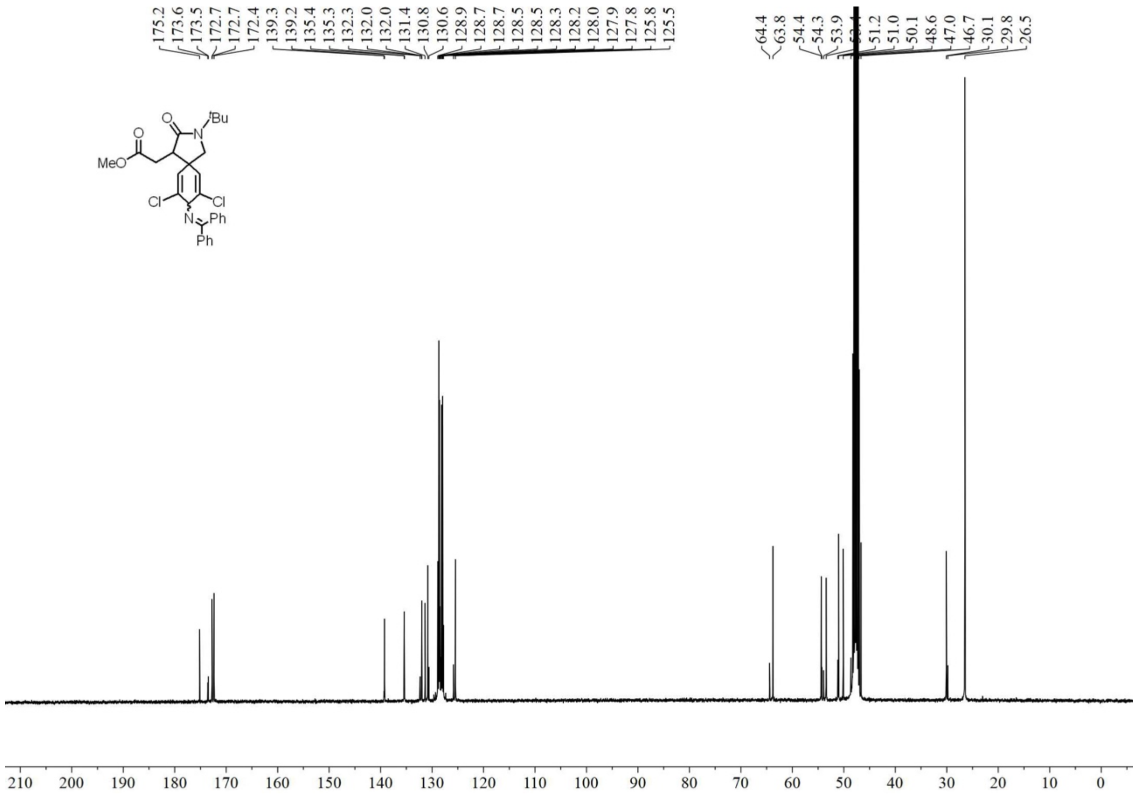

Methyl 2-(2-(*tert*-butyl)-8-((diphenylmethylene)amino)-6,10-dimethyl-3-oxo-2-azaspiro[4.5]deca-6,9-dien-4-yl)acetate (3t)

^{1}H NMR (400 MHz, CD_3OD)

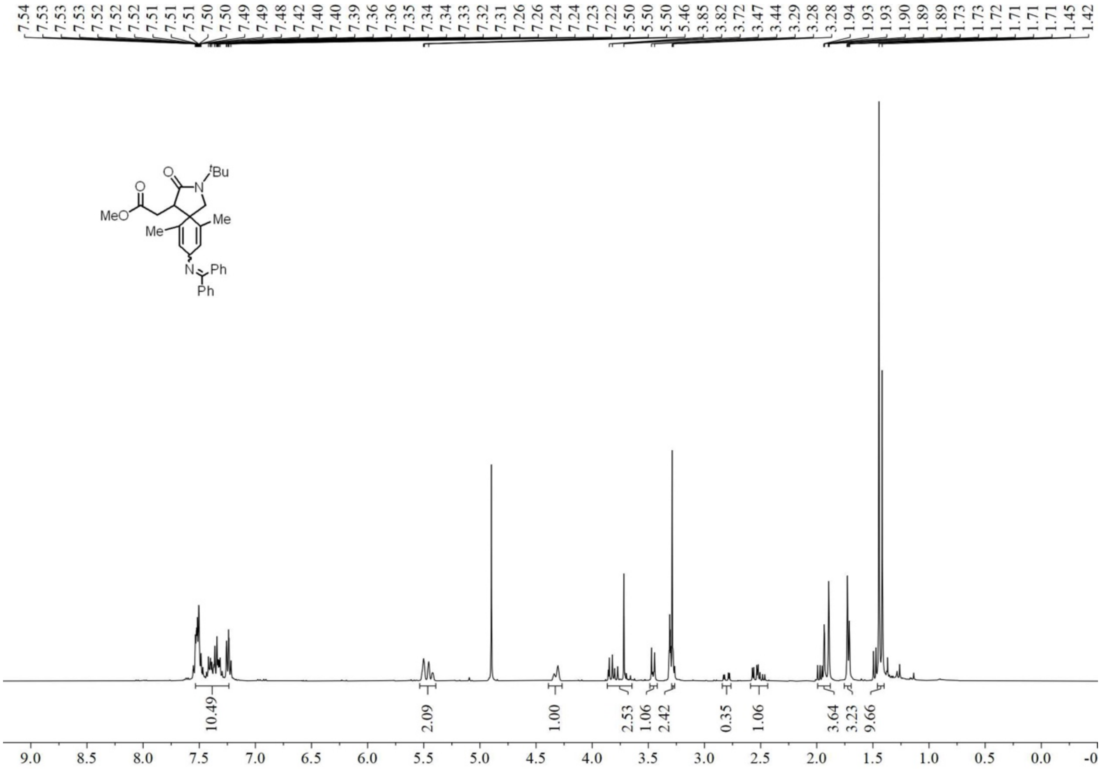

^{13}C NMR (101 MHz, CD_3OD)

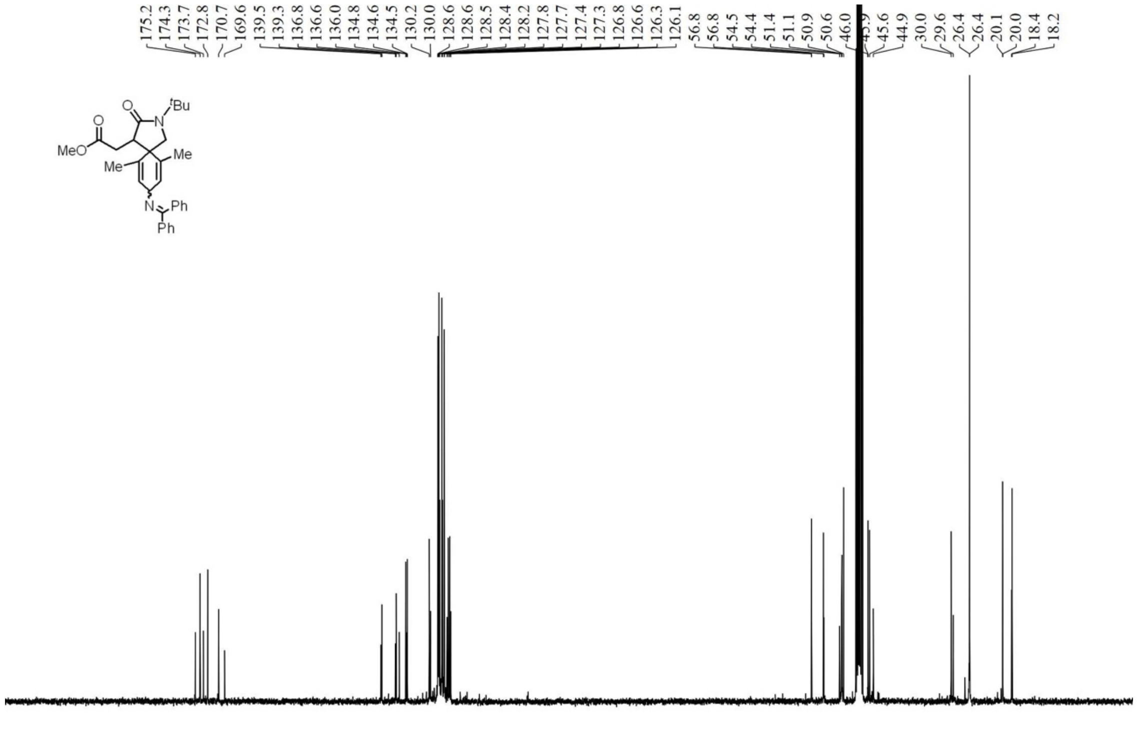

Methyl 2-(2-(*tert*-butyl)-8-((diphenylmethylene)amino)-1,3-dioxo-2-azaspiro[4.5]deca-6,9-dien-4-yl)acetate (3u)

Major product

^{1}H NMR (500 MHz, CD_2Cl_2)

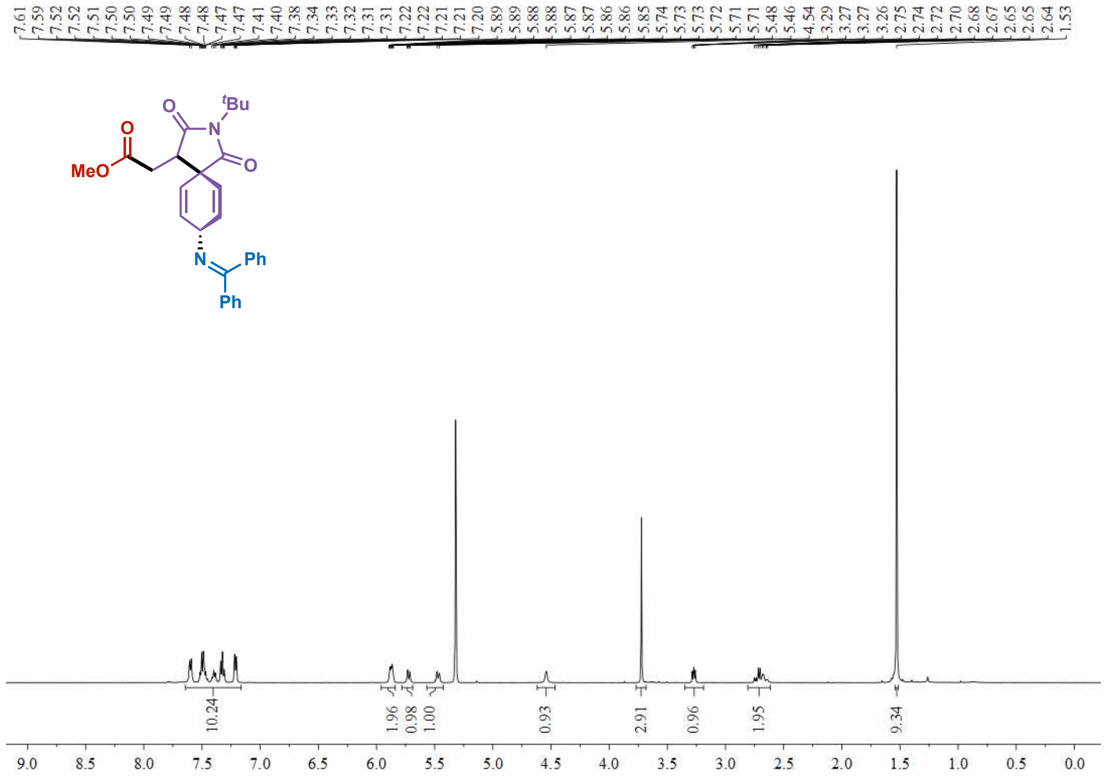

^{13}C NMR (126 MHz, CD_2Cl_2)

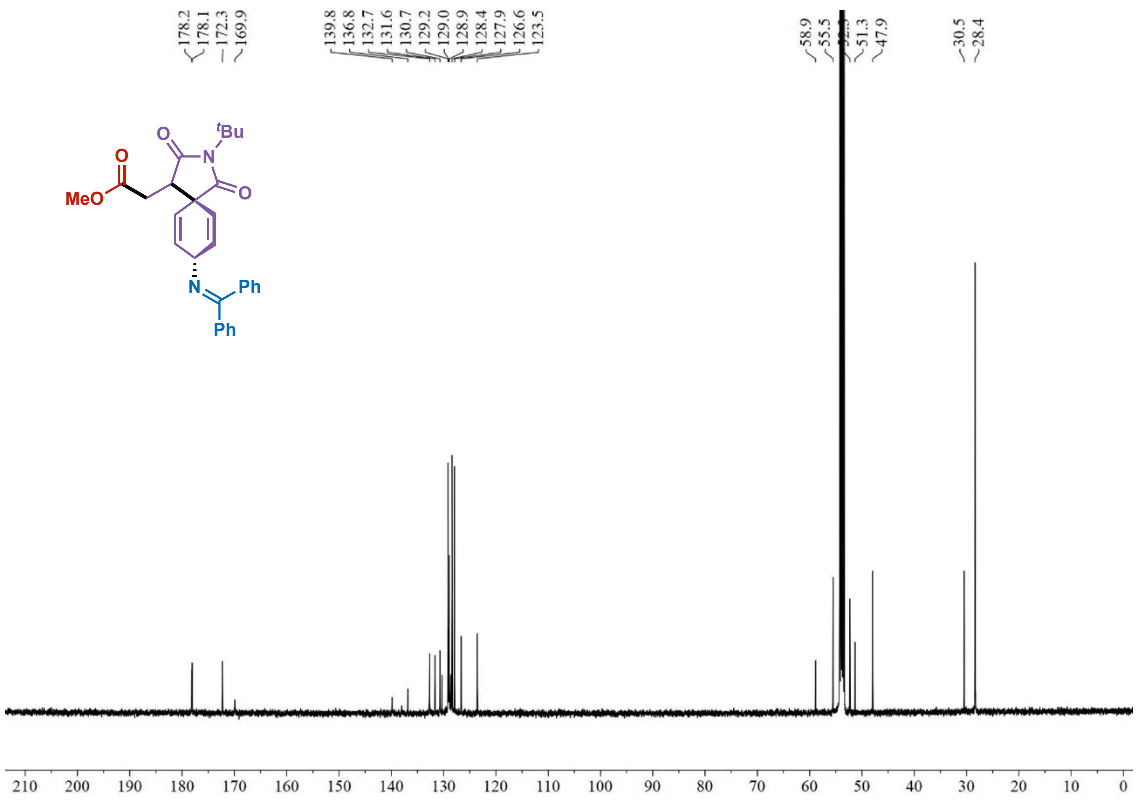

Minor product

^{1}H NMR (500 MHz, CD_3OD)

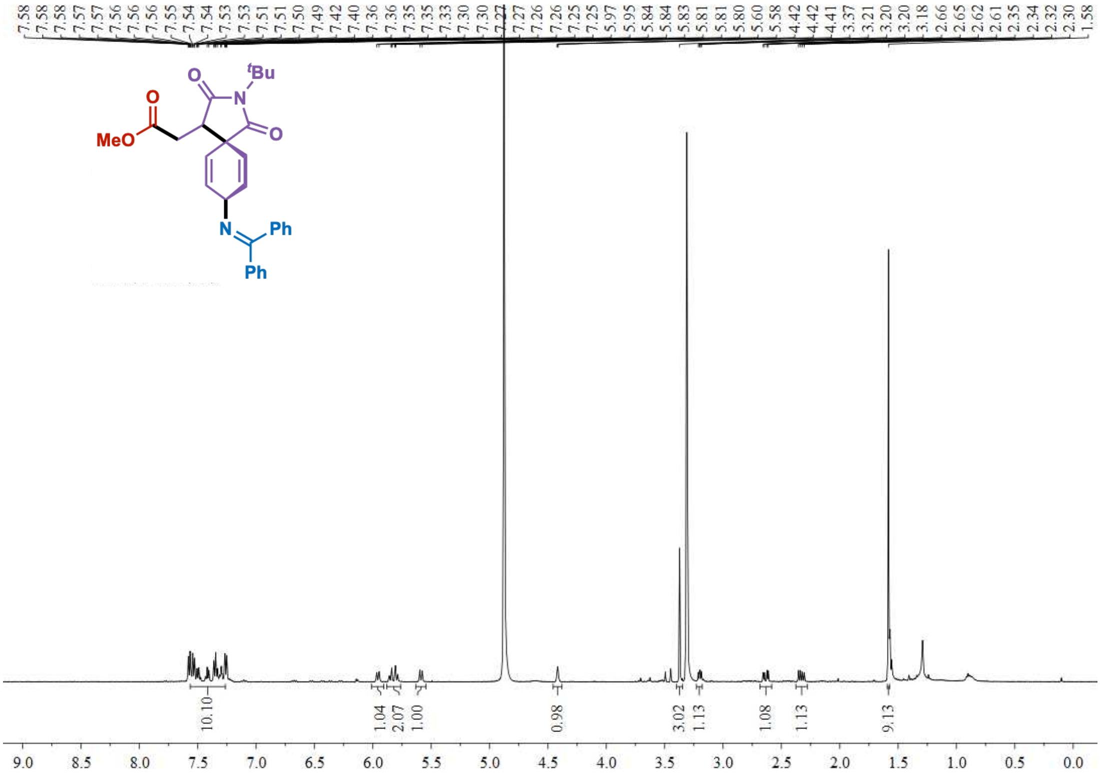

^{13}C NMR (126 MHz, CD_3OD)

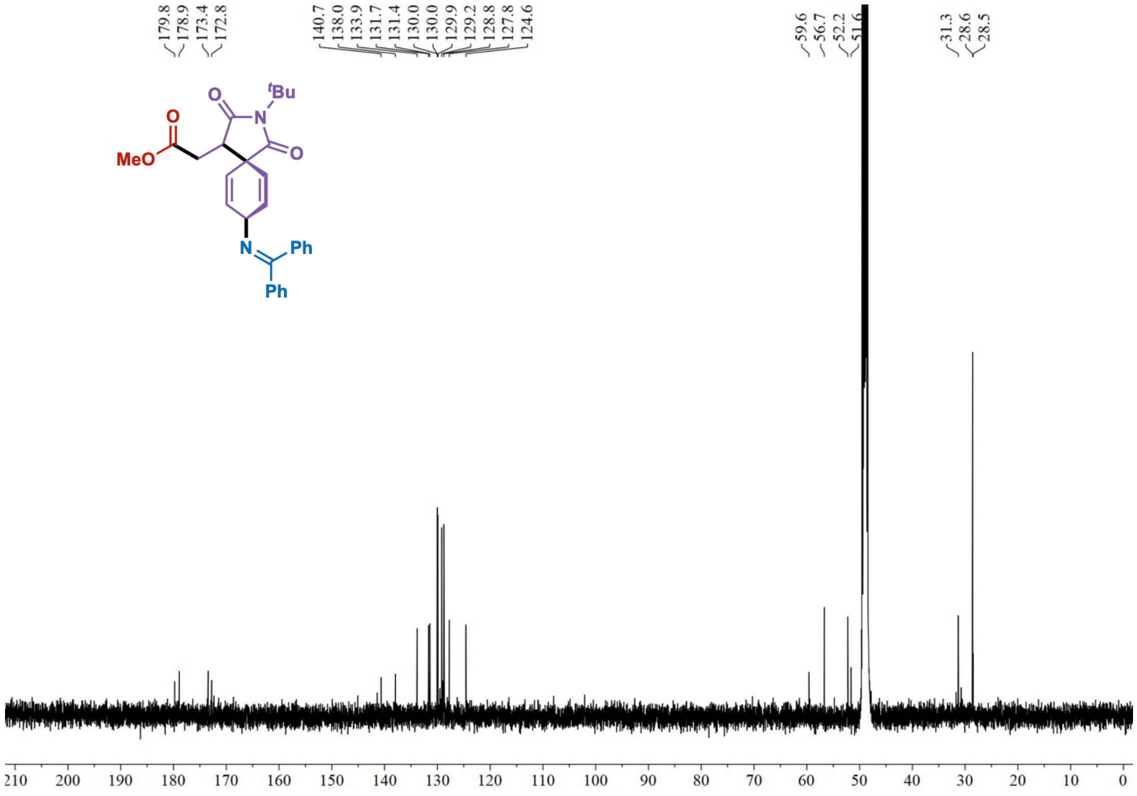

Methyl 2-(8-((diphenylmethylene)amino)-2-isopropyl-1,3-dioxo-2-azaspiro[4.5]deca-6,9-dien-4-yl)acetate (3v)

Major product

^{1}H NMR (400 MHz, CD_3OD)

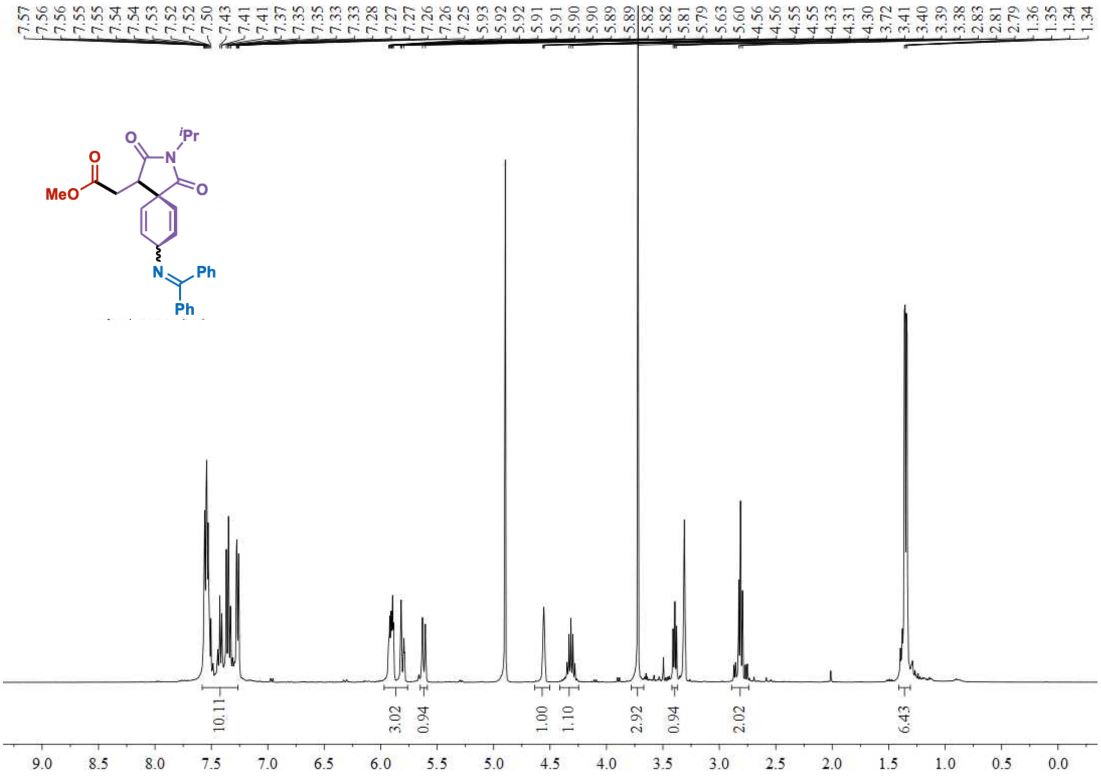

^{13}C NMR (101 MHz, CD_3OD)

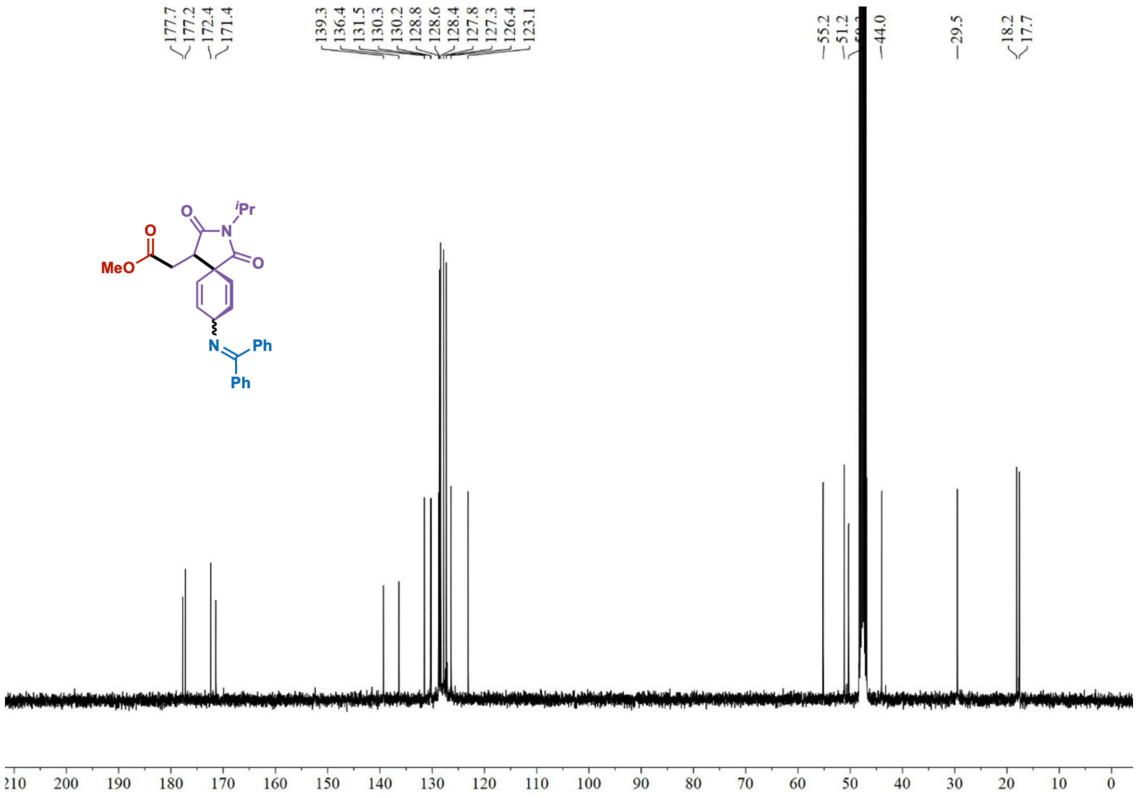

Minor product

^{1}H NMR (400 MHz, CD_3OD)

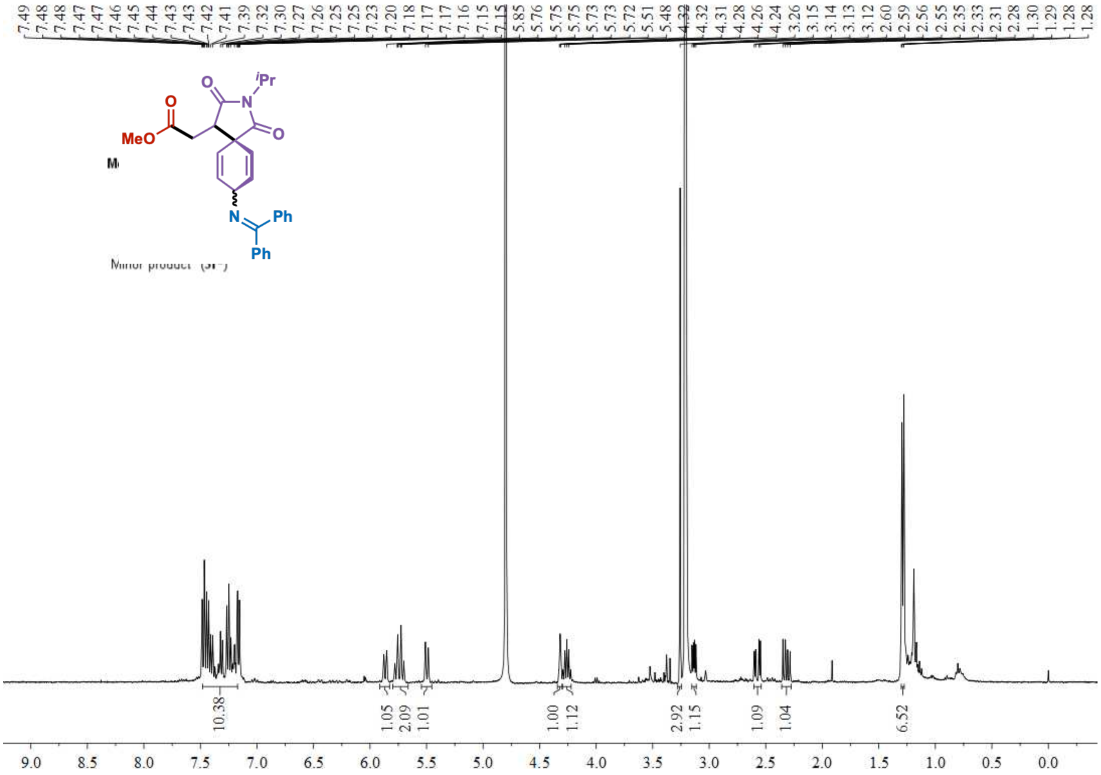

^{13}C NMR (101 MHz, CD_3OD)

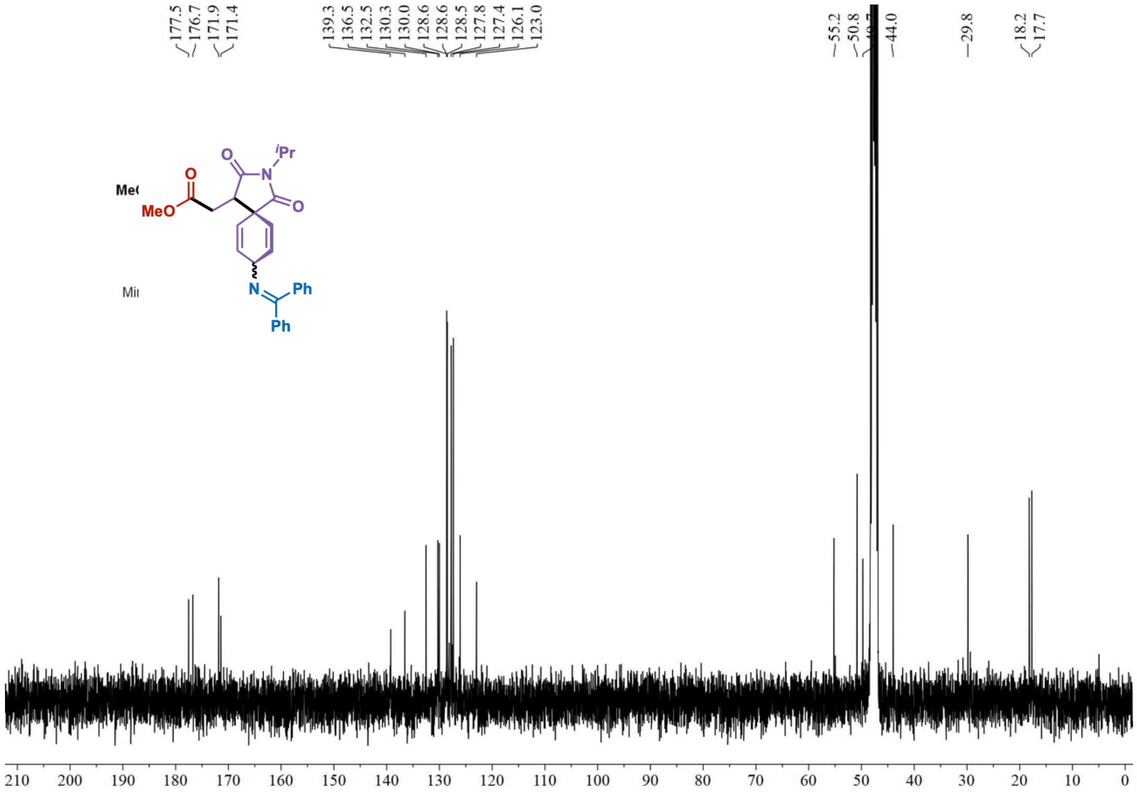

2-(*tert*-Butyl)-4-(5-(2,5-dimethylphenoxy)-2,2-dimethylpentyl)-8-((diphenylmethylene)amino)-2-azaspiro[4.5]deca-6,9-dien-3-one (3w)

^{1}H NMR (500 MHz, CD_3OD)

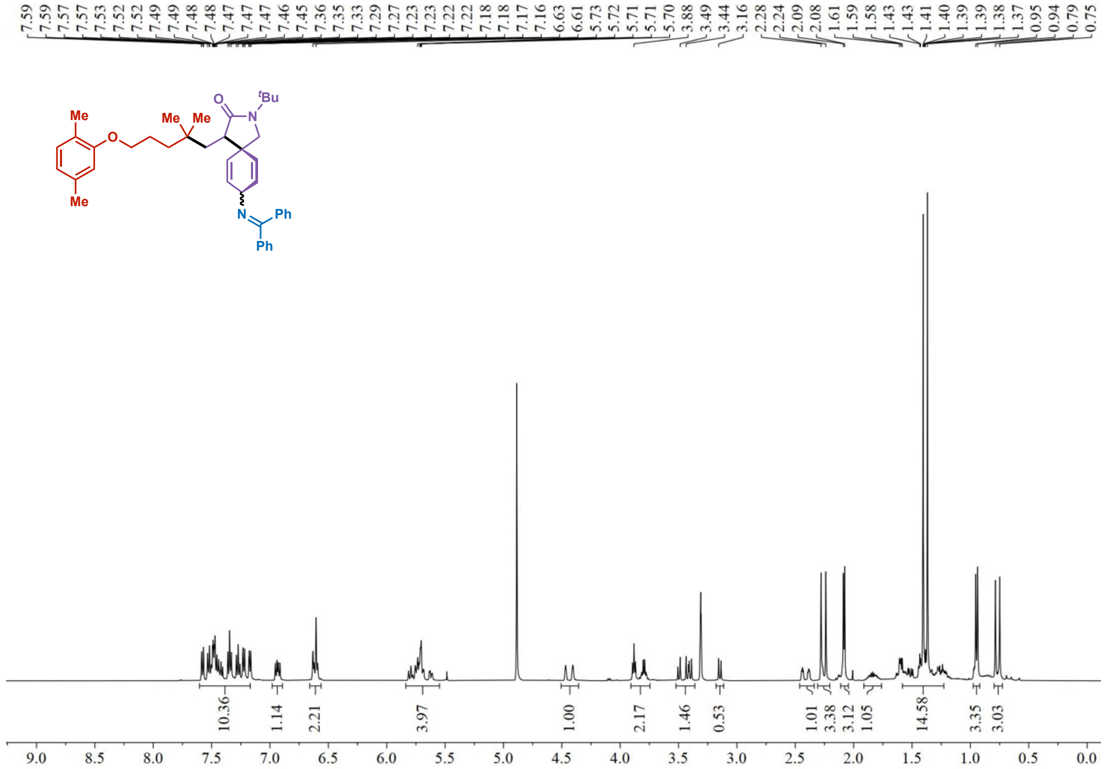

^{13}C NMR (126 MHz, CD_3OD)

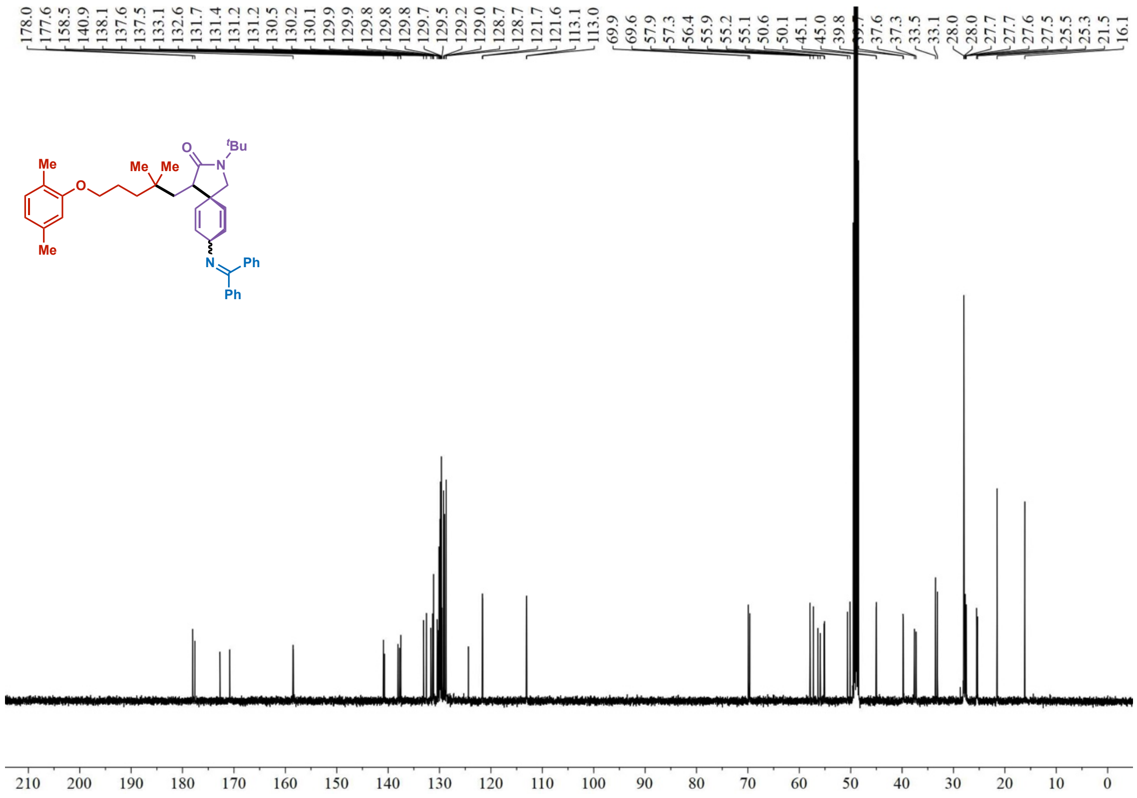

2-(tert-butyl)-4-(2-(4-(4-chlorobenzoyl)phenoxy)-2-methylpropyl)-8-((diphenylmethylene)amino)-2-azaspiro[4.5]deca-6,9-dien-3-one (3x).

Major isomer

^{1}H NMR (500 MHz, CD_3OD)

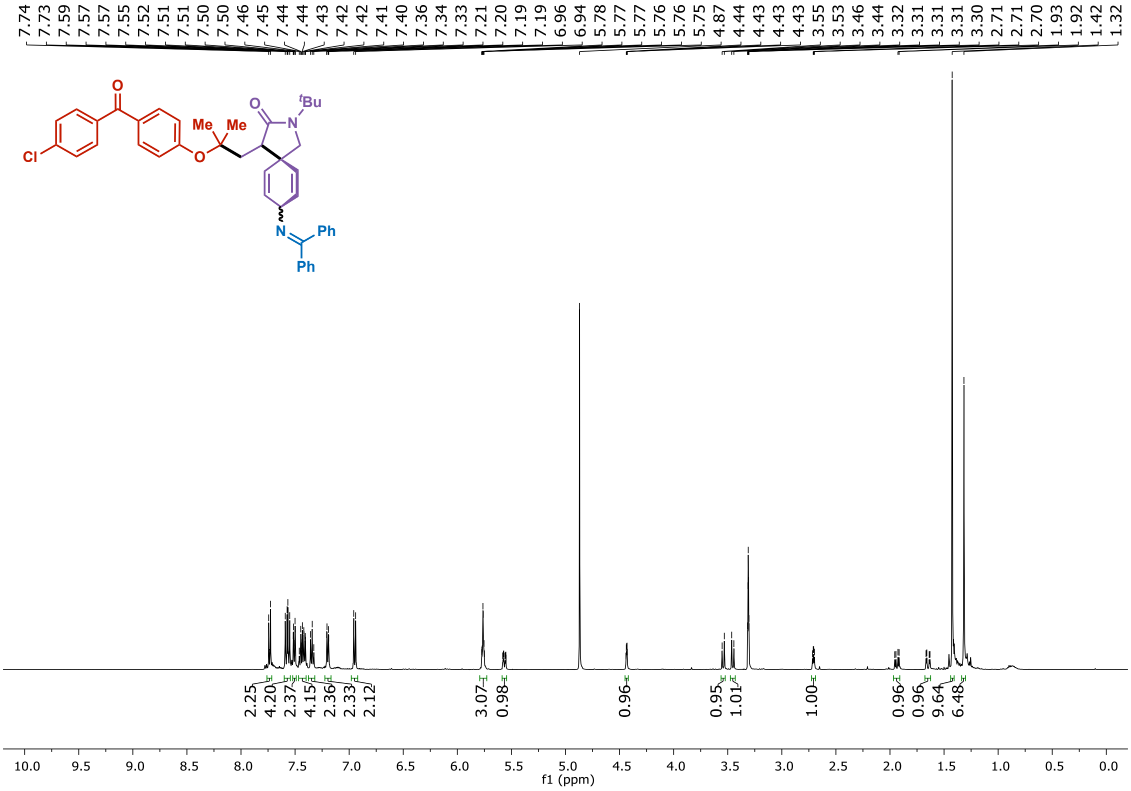

^{13}C NMR (126 MHz, CD_3OD)

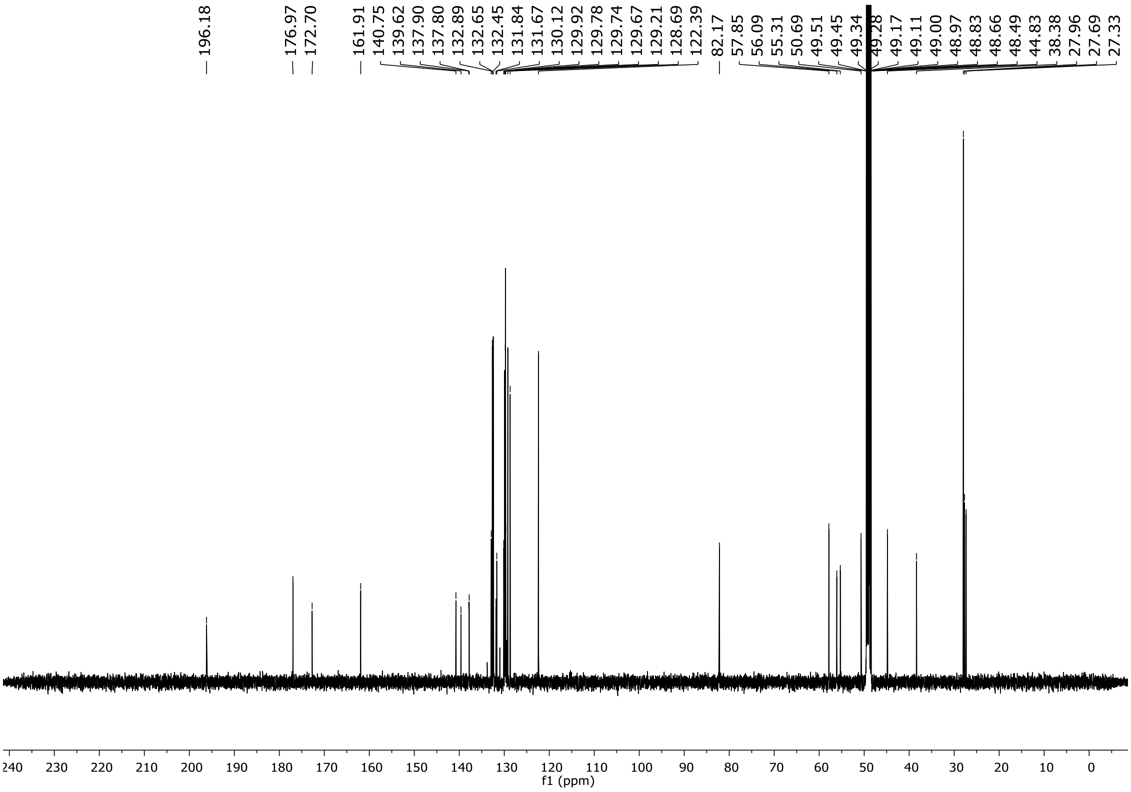

COSY

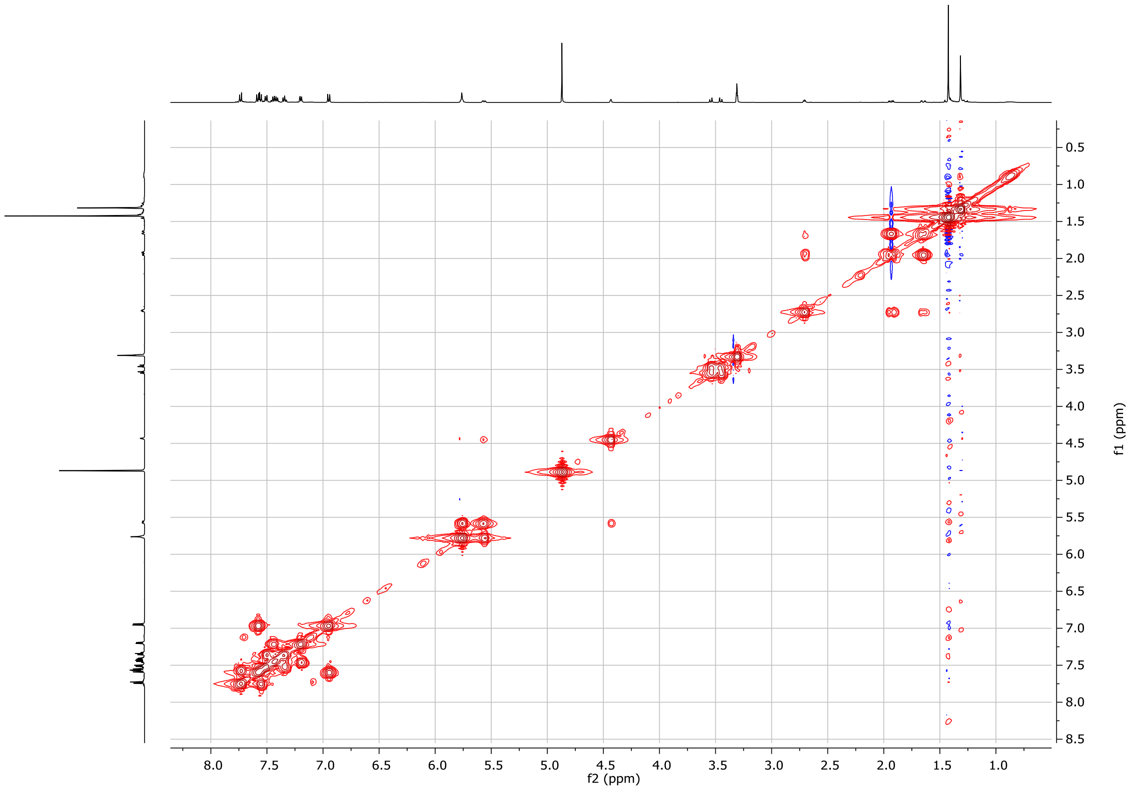

NOESY

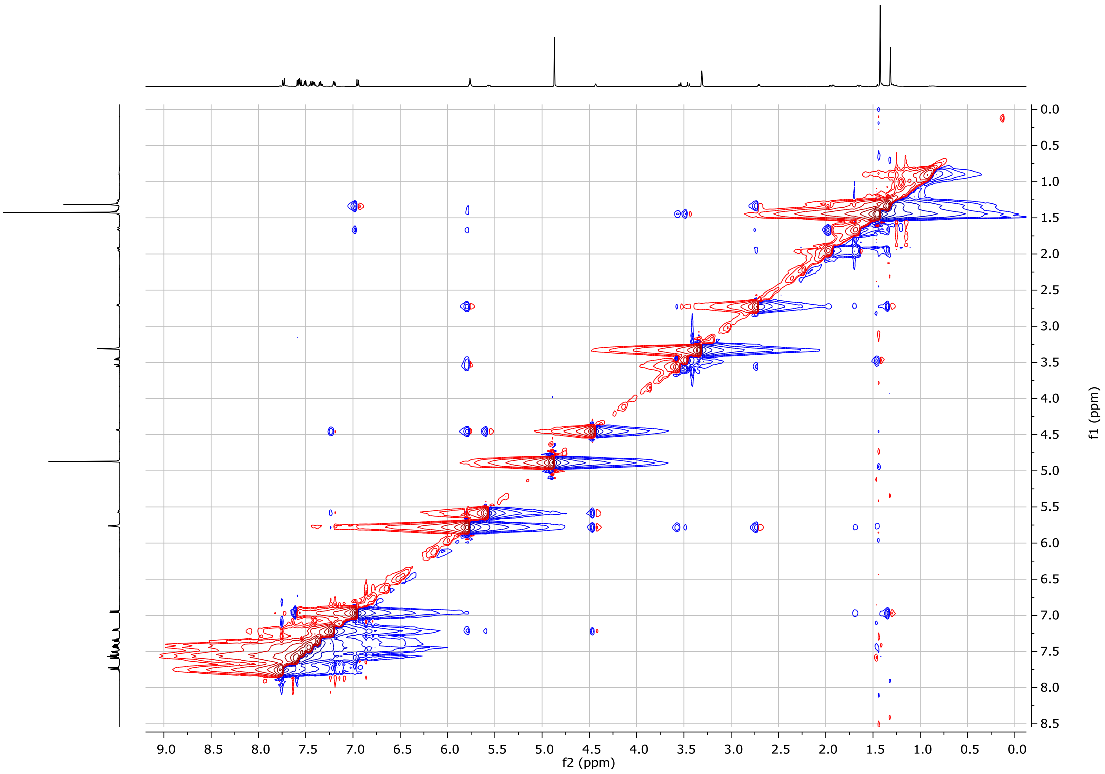

HSQC

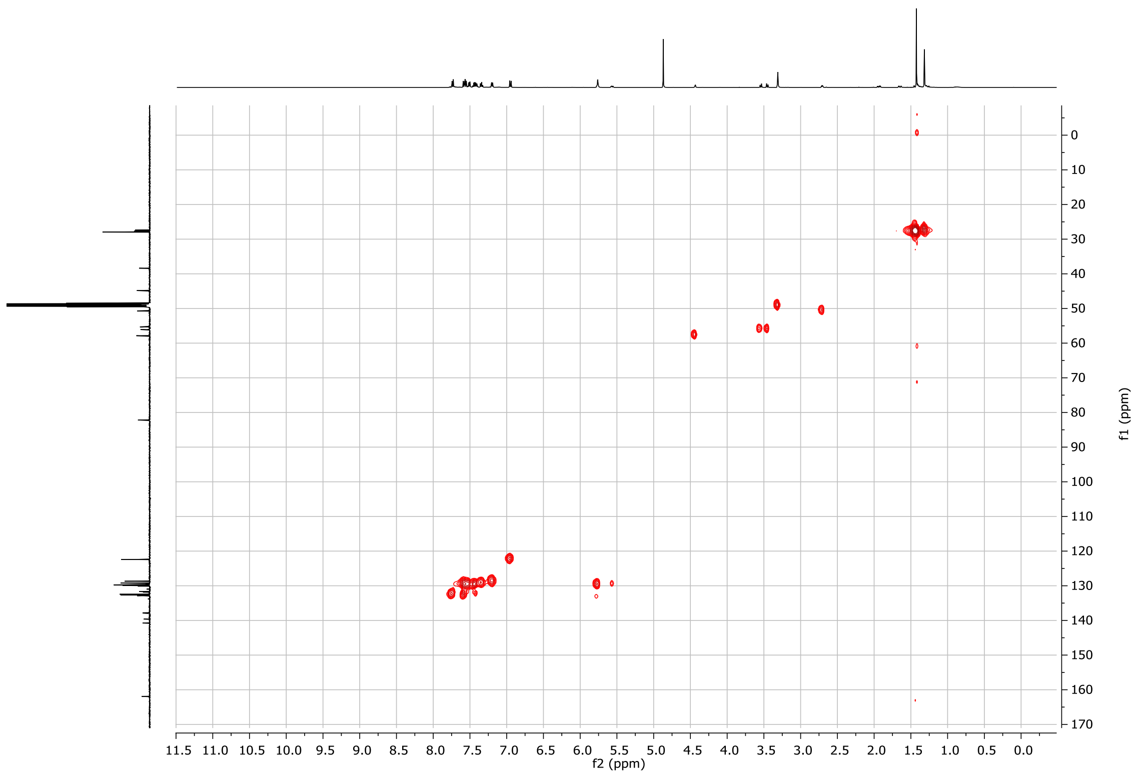

HMBC

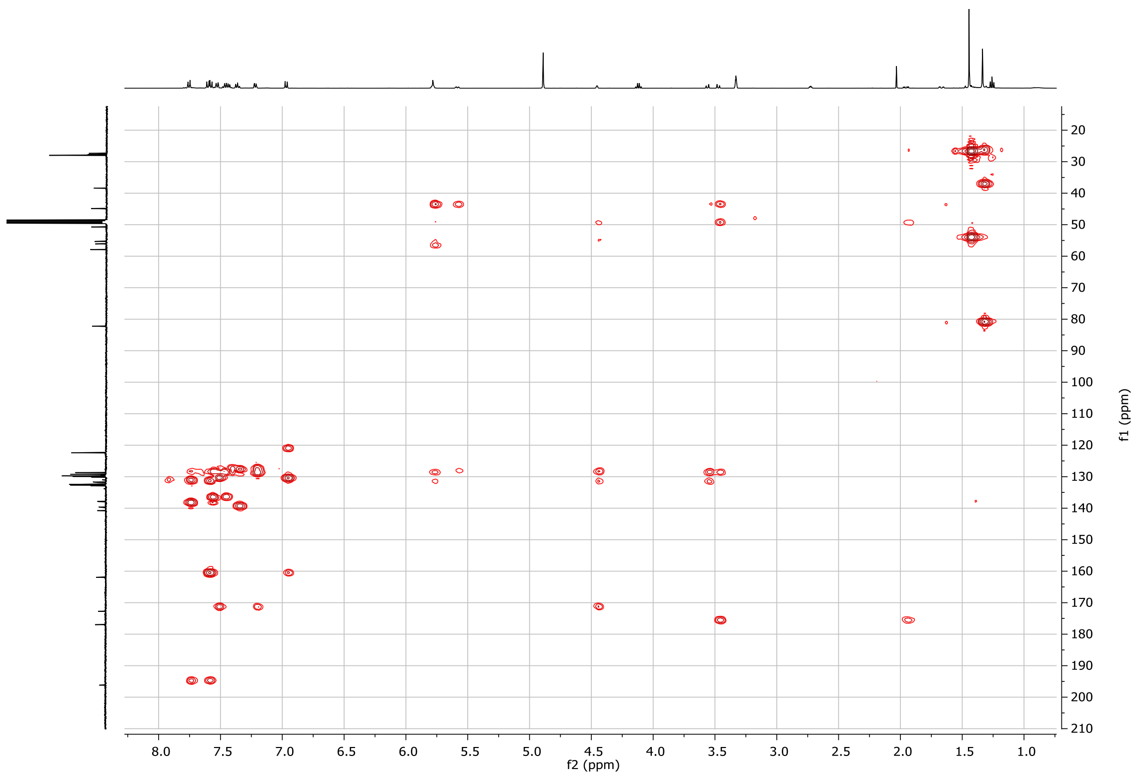

Minor isomer

^{1}H NMR (500 MHz, CD_3OD)

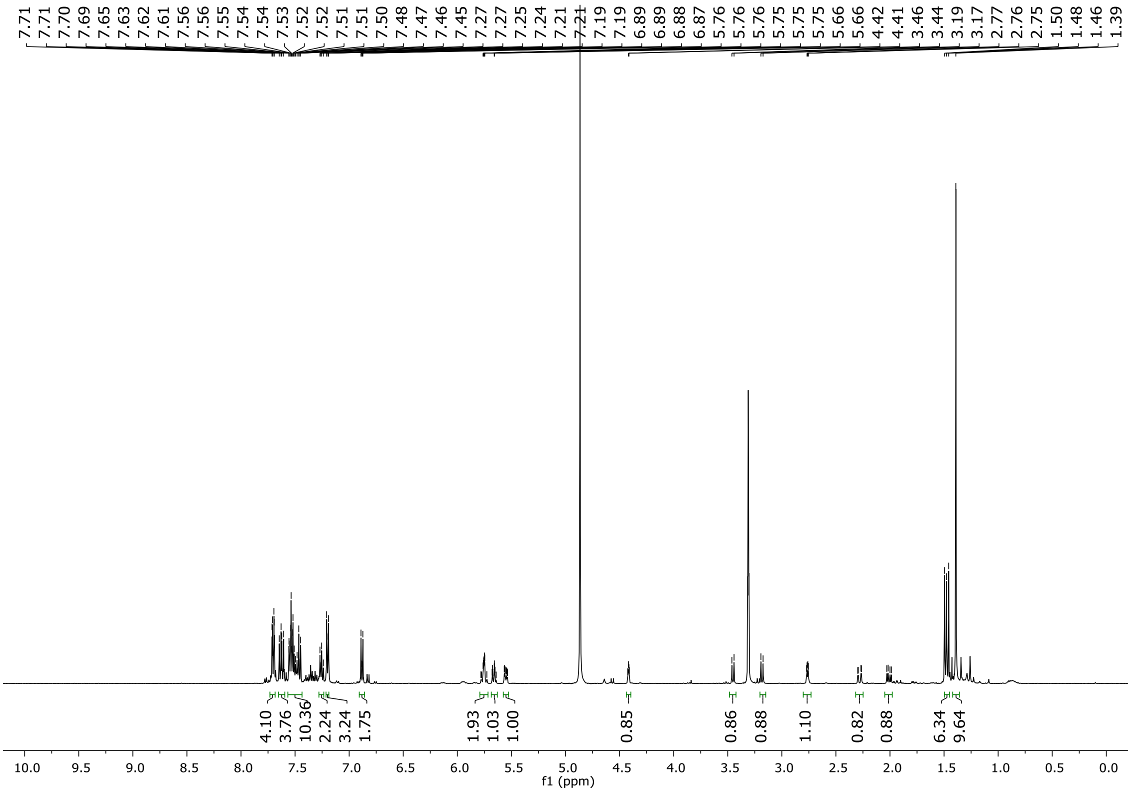

^{13}C NMR (126 MHz, CD_3OD)

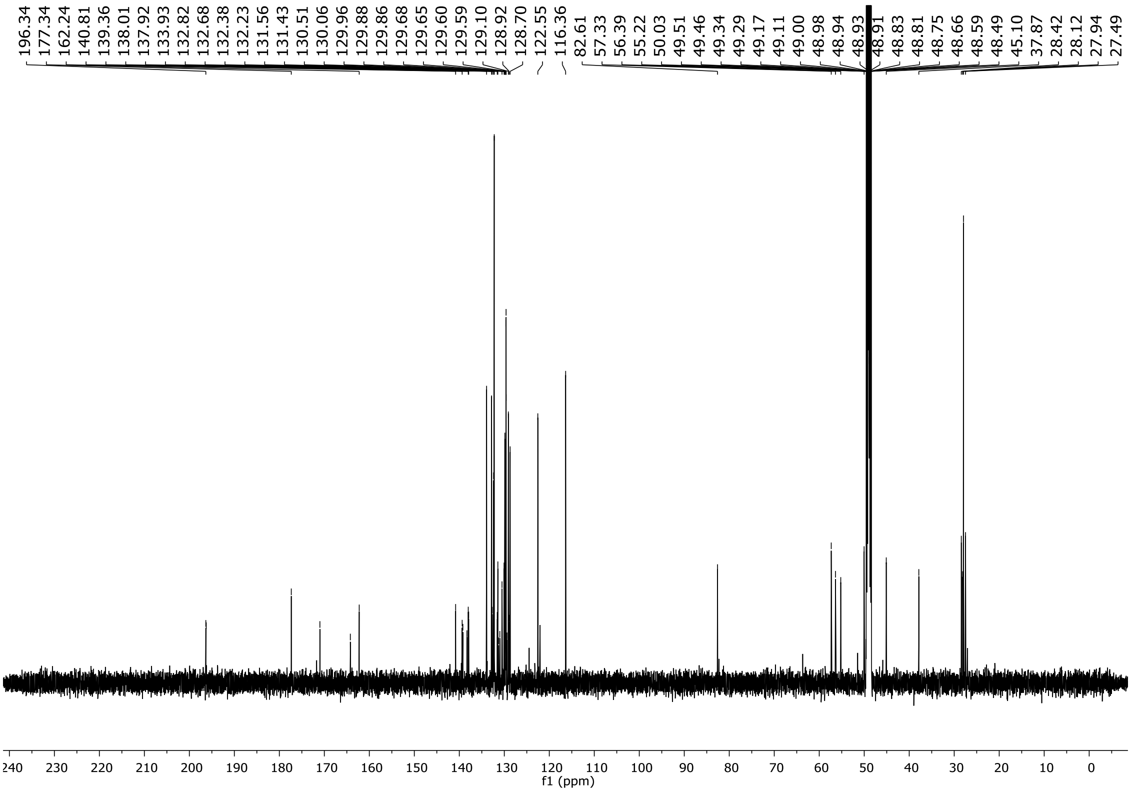

COSY

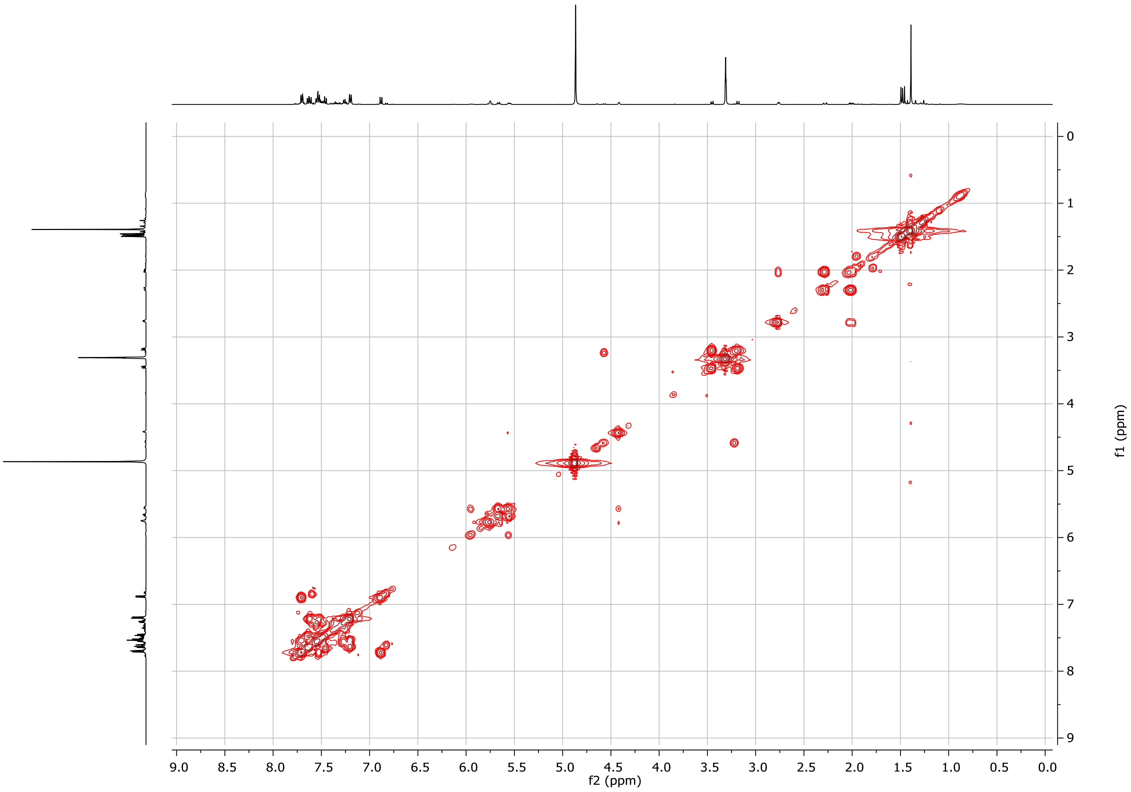

NOESY

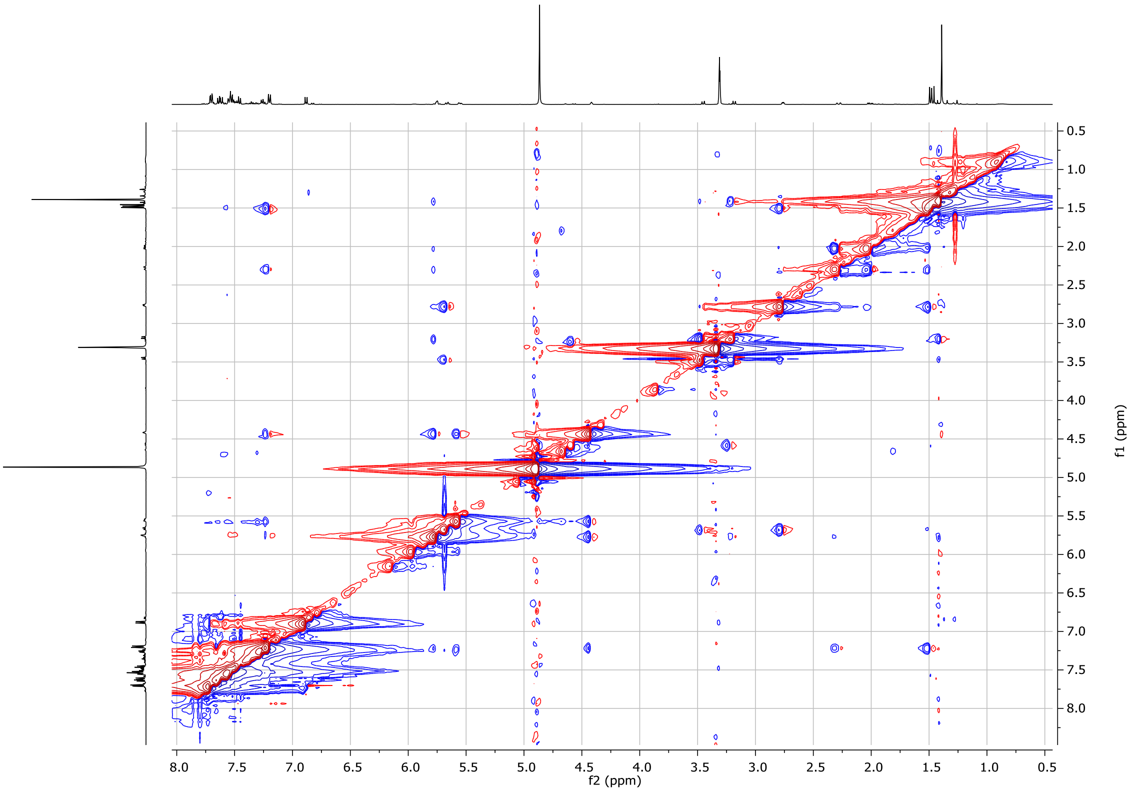

HSQC

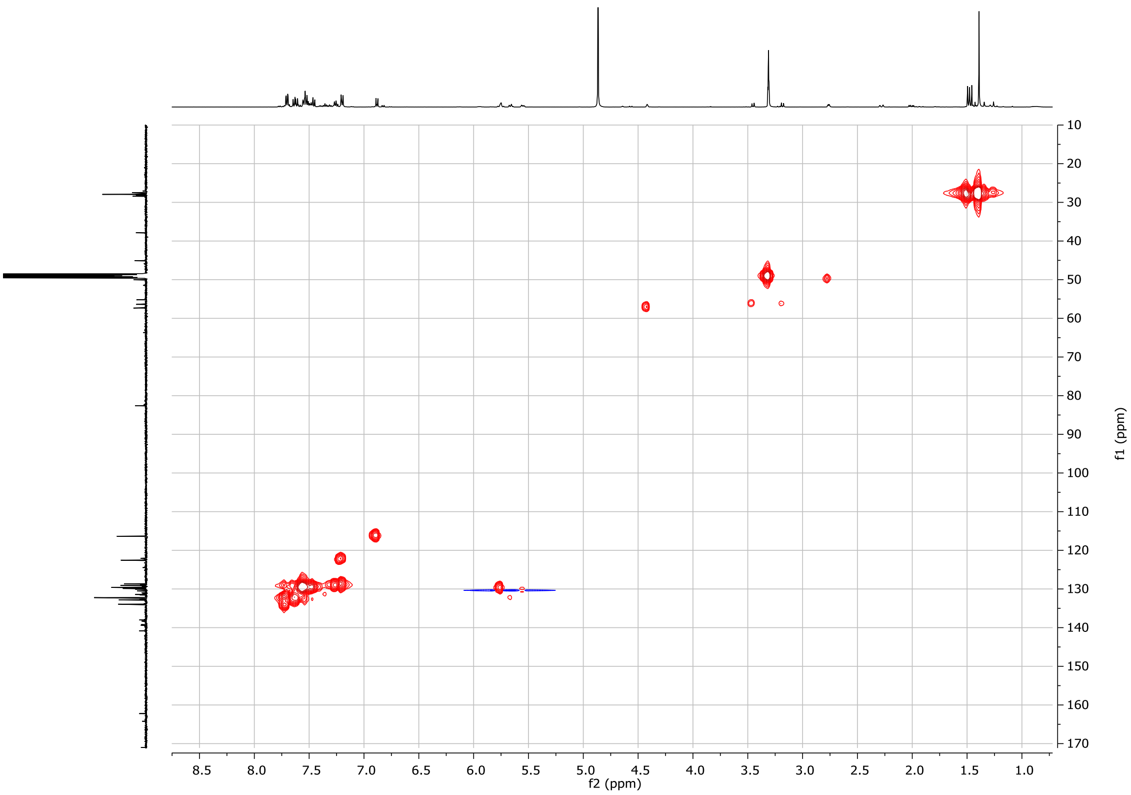

8.5
8.0
7.5
7.0
6.5
6.0
5.5
5.0
4.5
4.0
3.5
3.0
2.5
2.0
1.5
1.0
f2 (ppm)
f1 (ppm)
10
20
30
40
50
60
70
80
90
100
110
120
130
140
150
160
170

HMBC

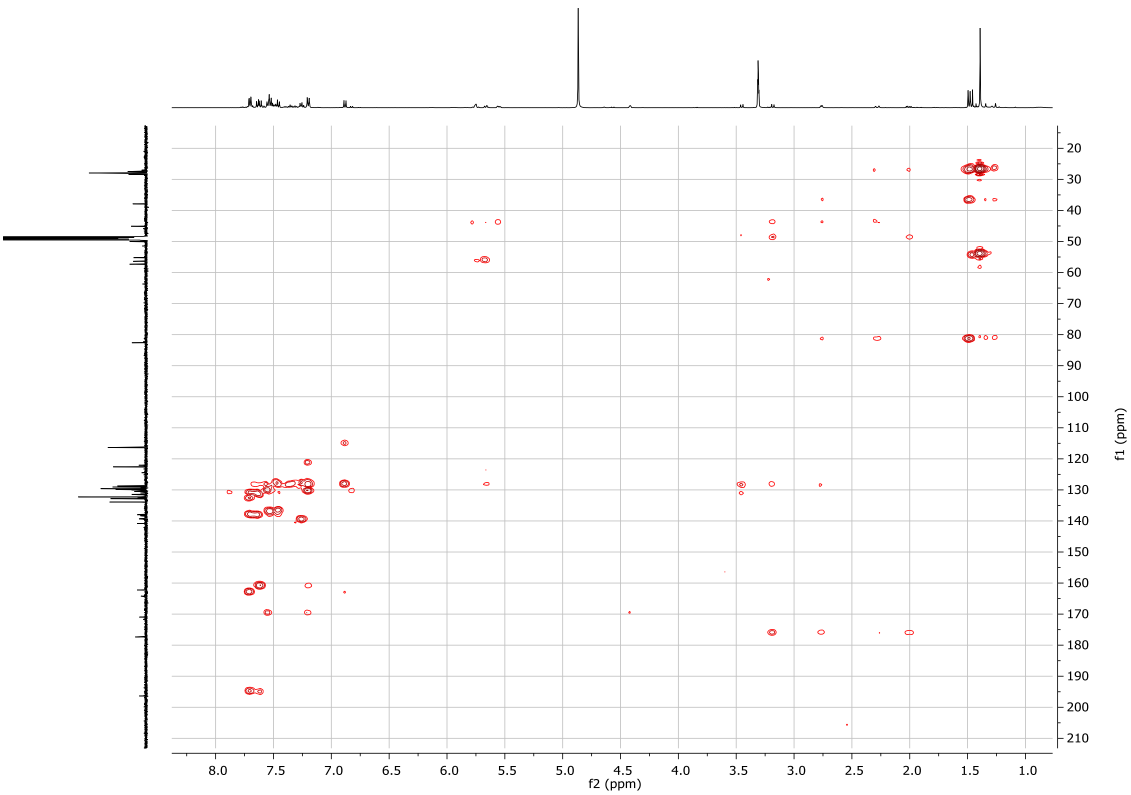

8.0
7.5
7.0
6.5
6.0
5.5
5.0
4.5
4.0
3.5
3.0
2.5
2.0
1.5
1.0
f2 (ppm)
f1 (ppm)
20
30
40
50
60
70
80
90
100
110
120
130
140
150
160
170
180
190
200
210

2-(*tert*-butyl)-4-(2-(4-(2,2-dichlorocyclopropyl)phenoxy)-2-methylpropyl)-8-((diphenylmethylene)amino)-2-azaspiro[4.5]deca-6,9-dien-3-one (3y)

^{1}H NMR (500 MHz, CD_3OD)

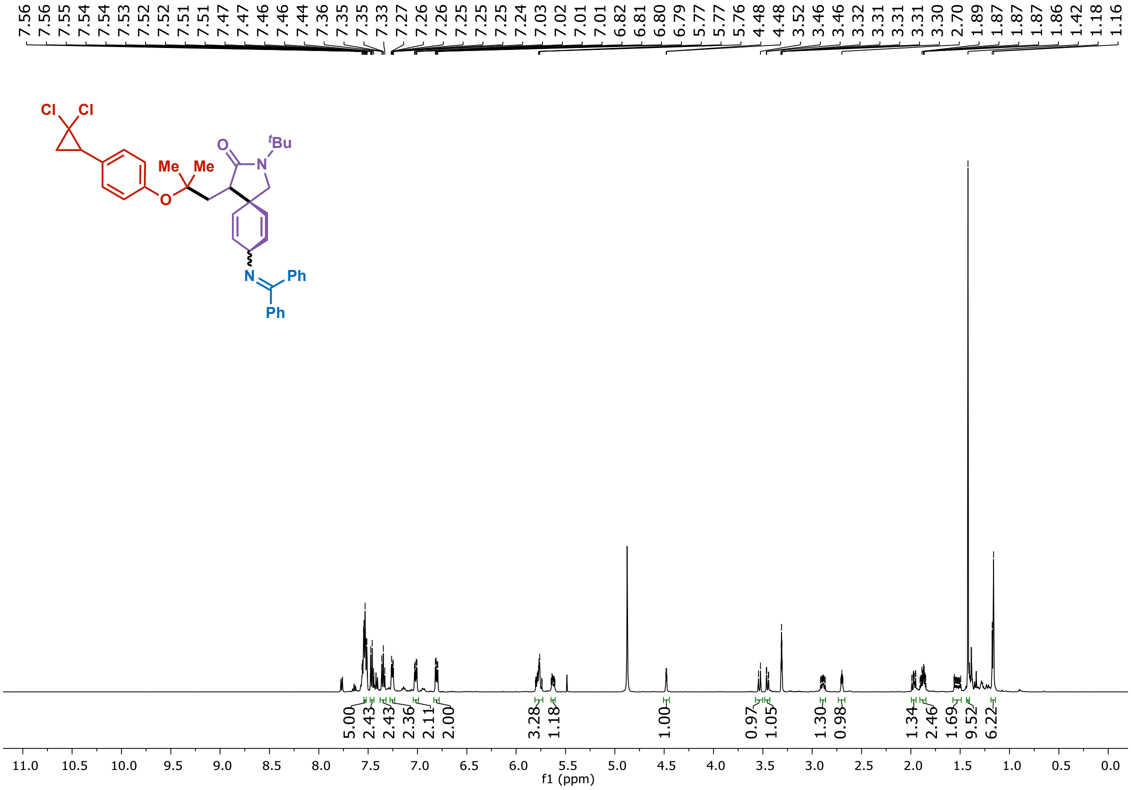

^{13}C NMR (126 MHz, CD_3OD)

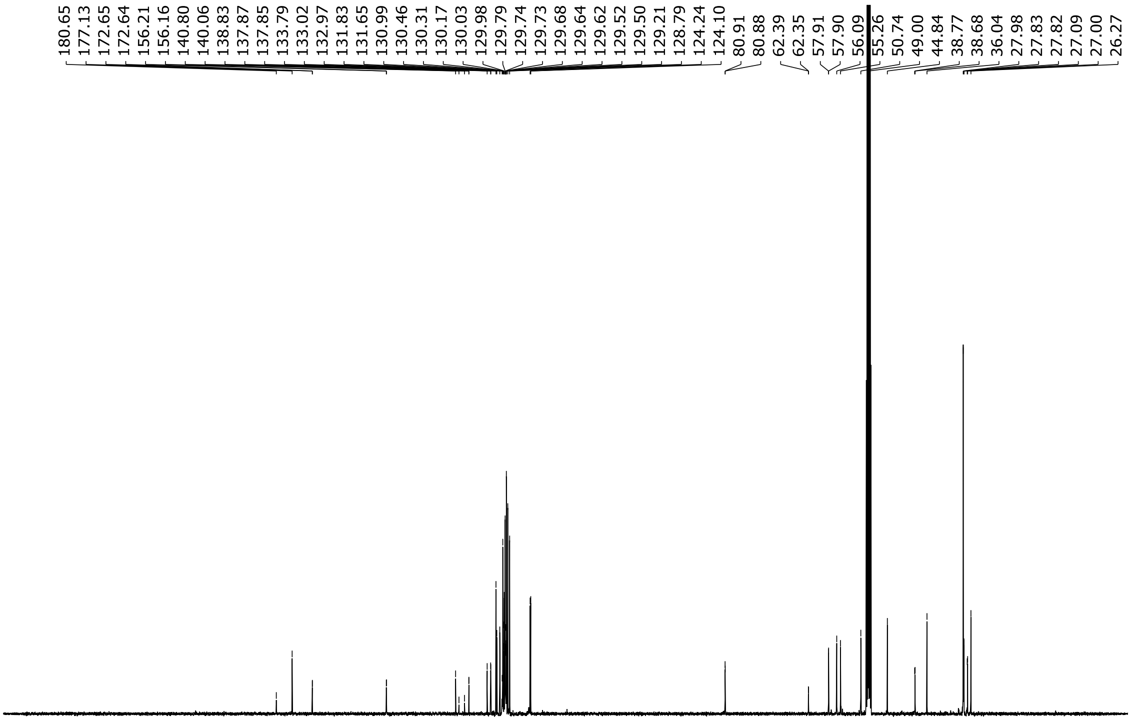

2-(*tert*-Butyl)-8-((diphenylmethylene)amino)-4-(3-(4,5-diphenyloxazol-2-yl)propyl)-2-azaspiro[4.5]deca-6,9-dien-3-one (3z)

^{1}H NMR (500 MHz, CD_3OD)

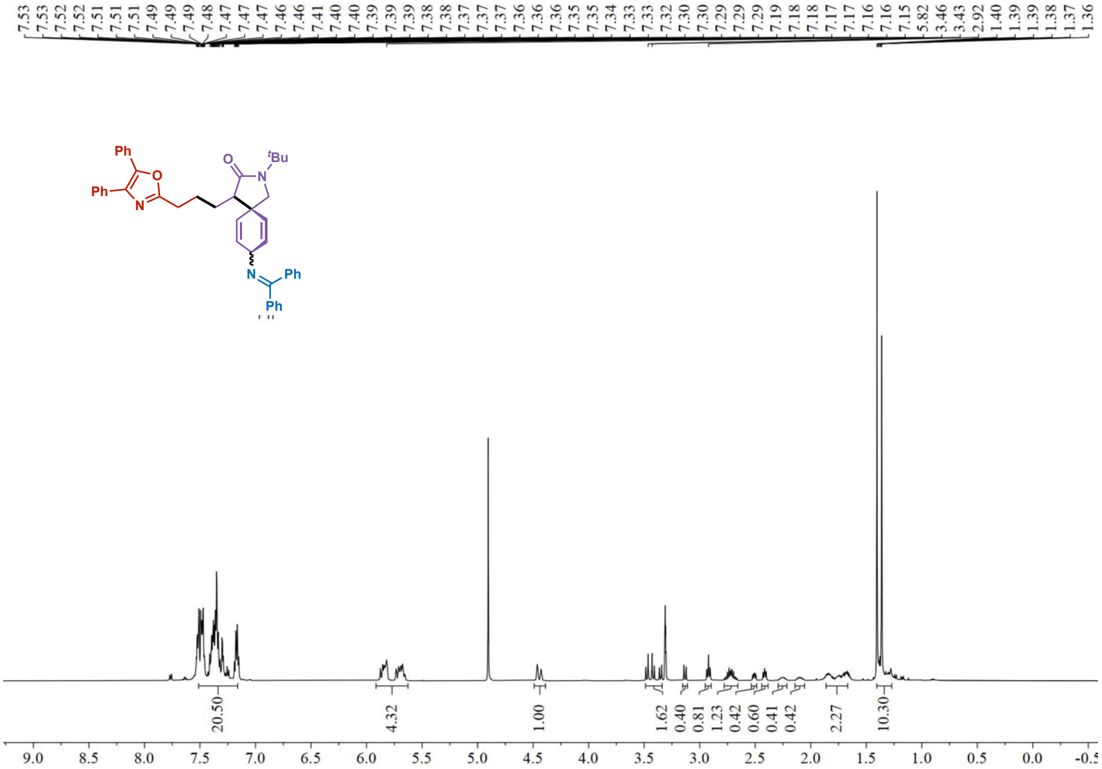

^{13}C NMR (126 MHz, CD_3OD)

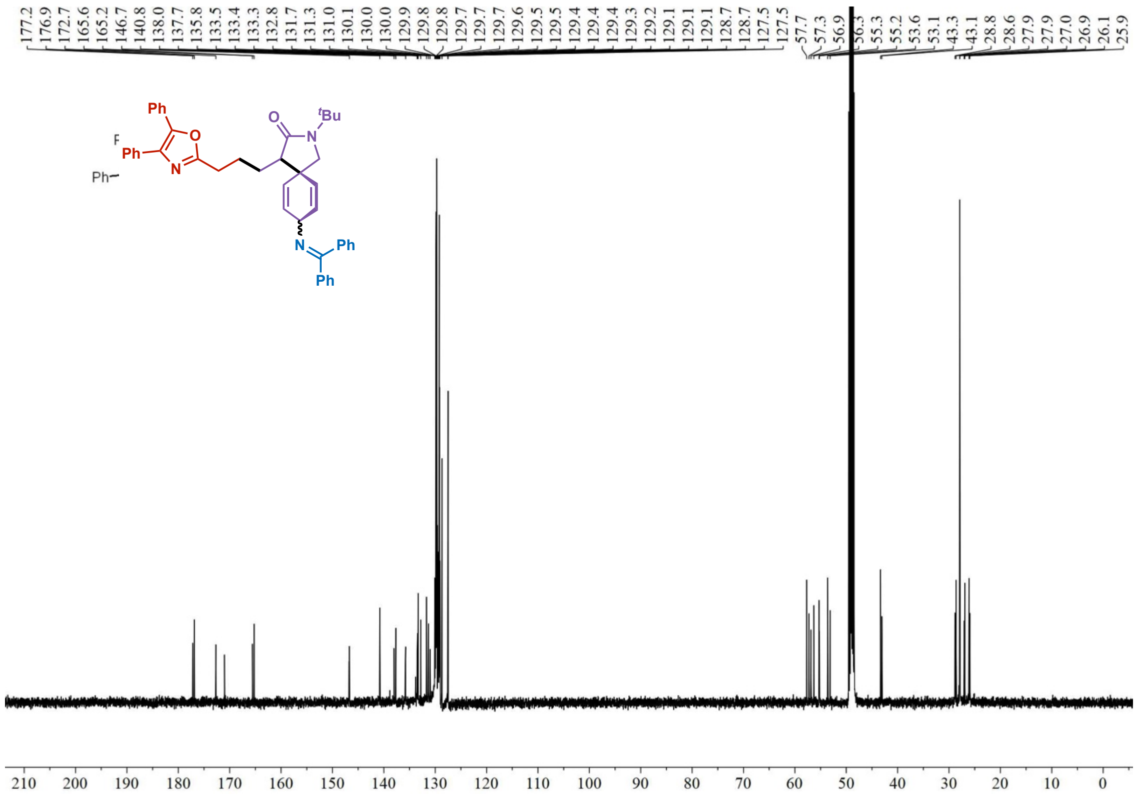

(5*R*)-2-((2-(*tert*-butyl)-8-((diphenylmethylene)amino)-3-oxo-2-azaspiro[4.5]deca-6,9-dien-4-yl)methyl)-3,3-dimethyl-4-thia-1-azabicyclo[3.2.0]heptan-7-one 4,4-dioxide (3aa).

^{1}H NMR (500 MHz, CD_3OD)

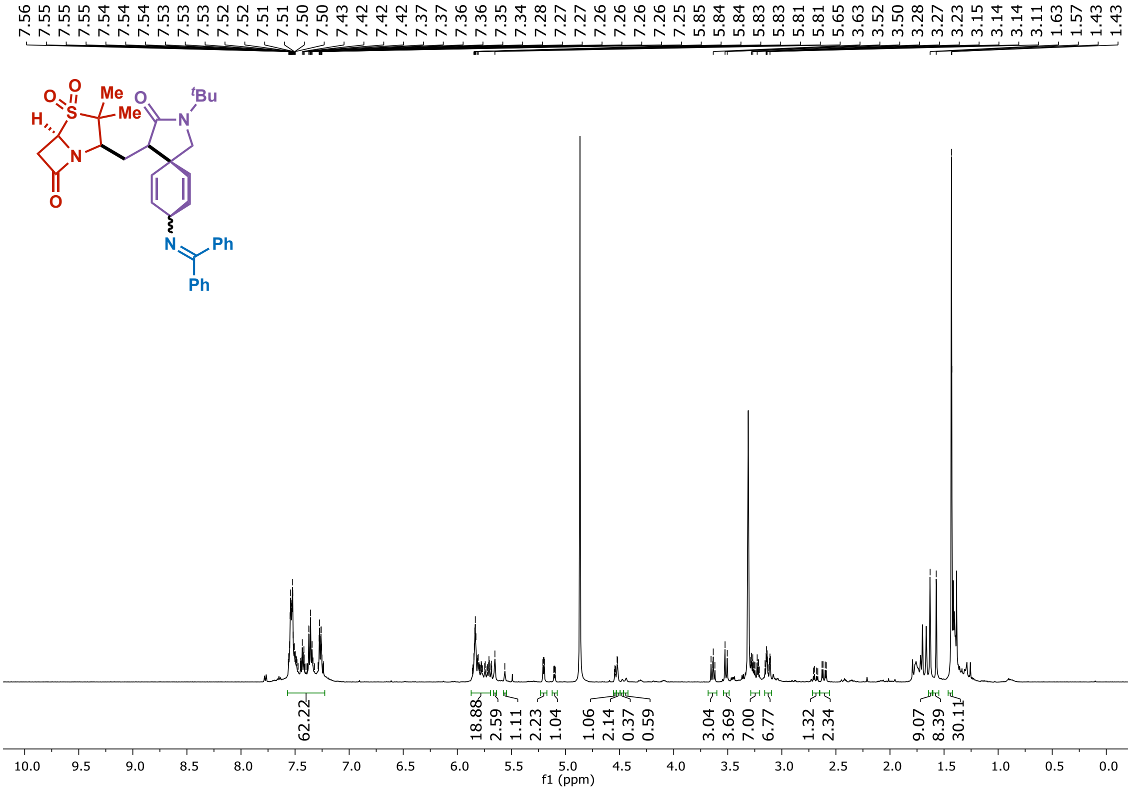

^{13}C NMR (126 MHz, CD_3OD)

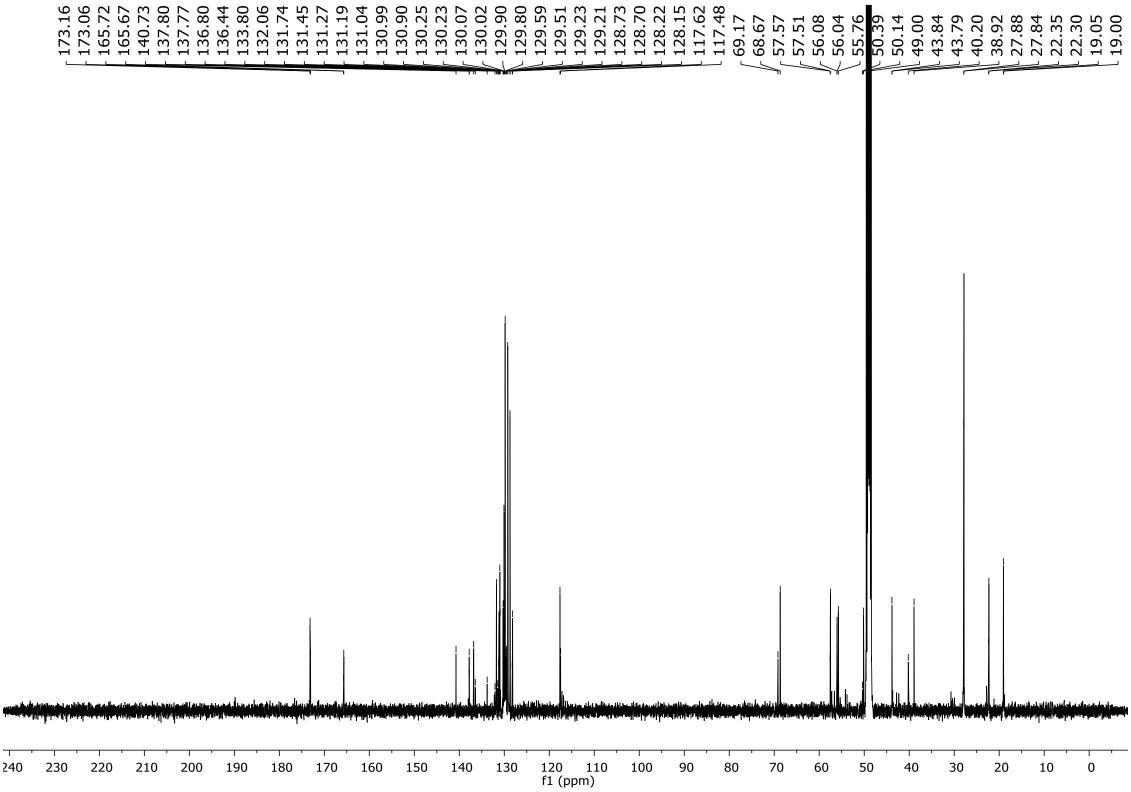

(1r,4R)-*N*-(1-((*R*)-2-(*tert*-butyl)-8-((diphenylmethylene)amino)-3-oxo-2-azaspiro[4.5]deca-6,9-dien-4-yl)-3-phenylpropan-2-yl)-4-isopropylcyclohexane-1-carboxamide (3ab).

^{1}H NMR (500 MHz, CD_3OD)

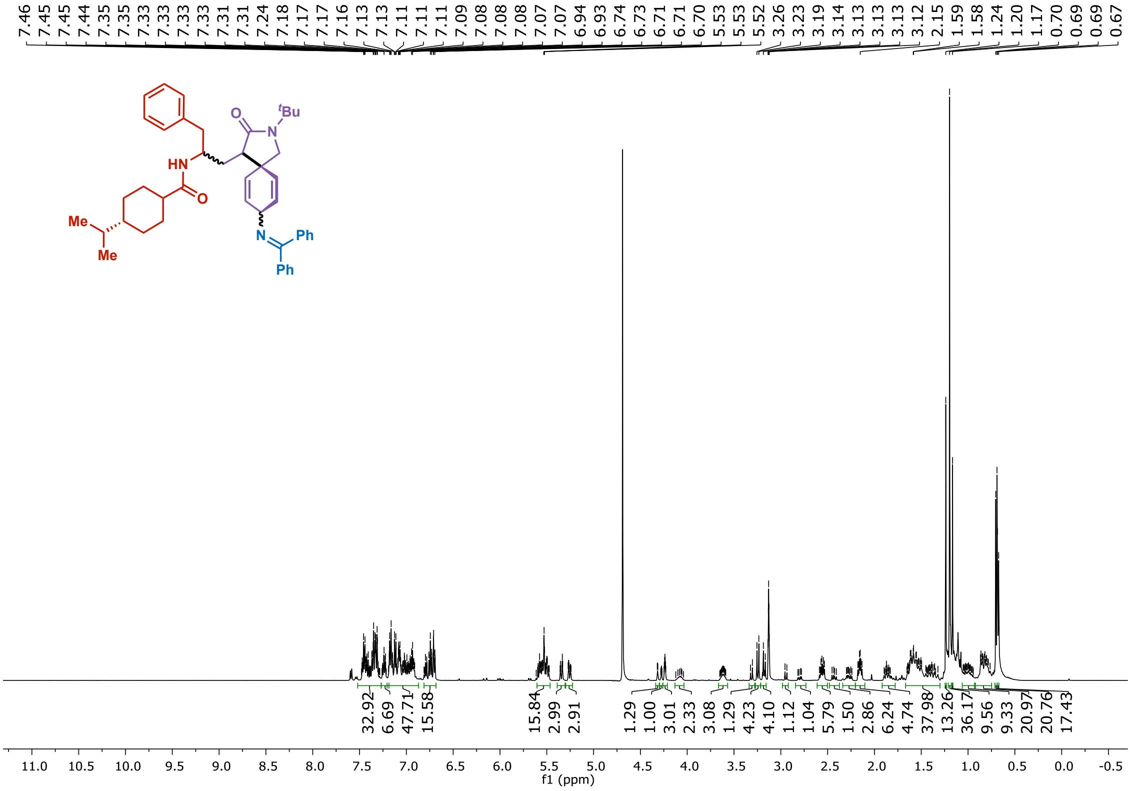

^{13}C NMR (126 MHz, CD_3OD)

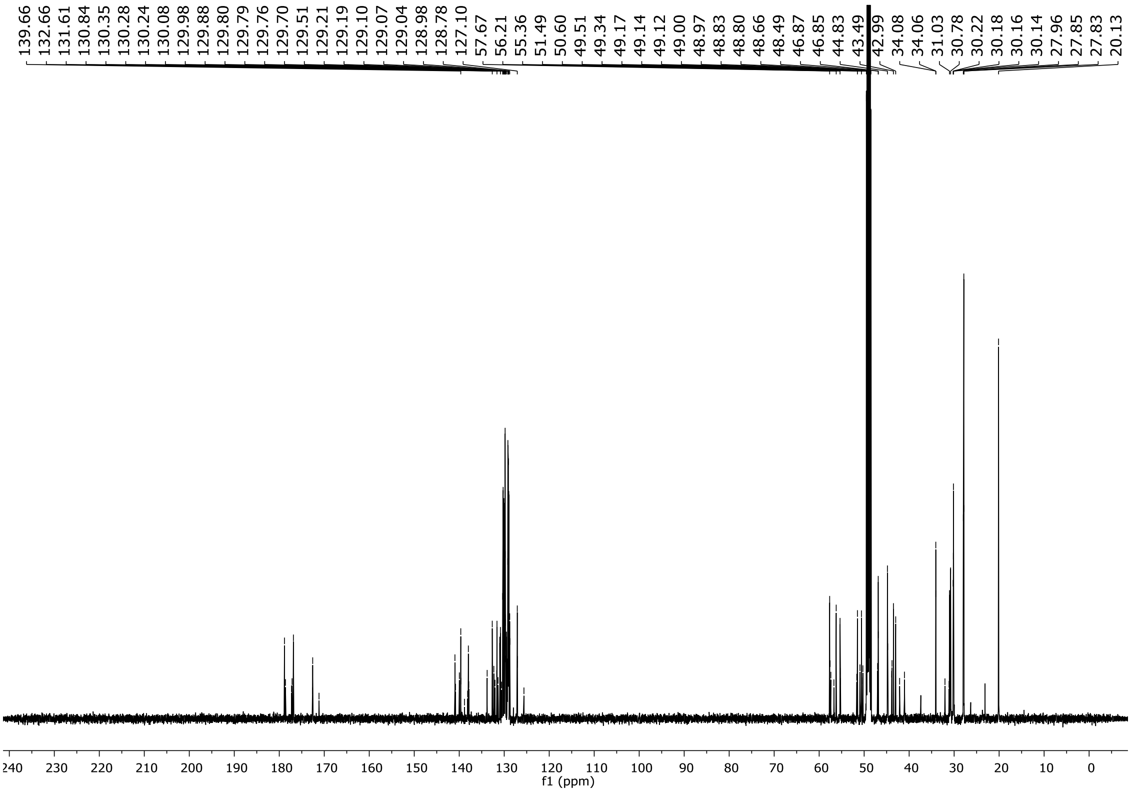

***N*-(2-(2-(tert-butyl)-8-((diphenylmethylene)amino)-3-oxo-2-azaspiro[4.5]deca-6,9-dien-4-yl)ethyl)-4-methylbenzenesulfonamide (3ac)**

Major isomer

^{1}H NMR (500 MHz, CD_3OD)

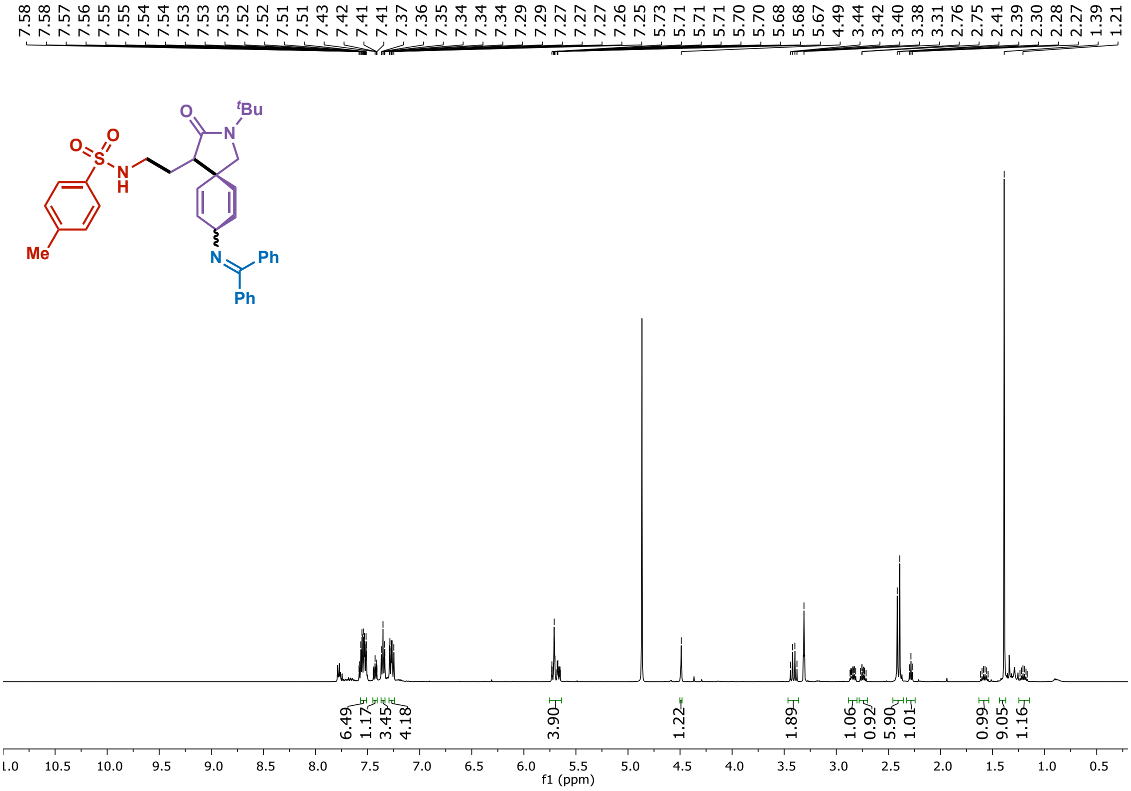

^{13}C NMR (126 MHz, CD_3OD)

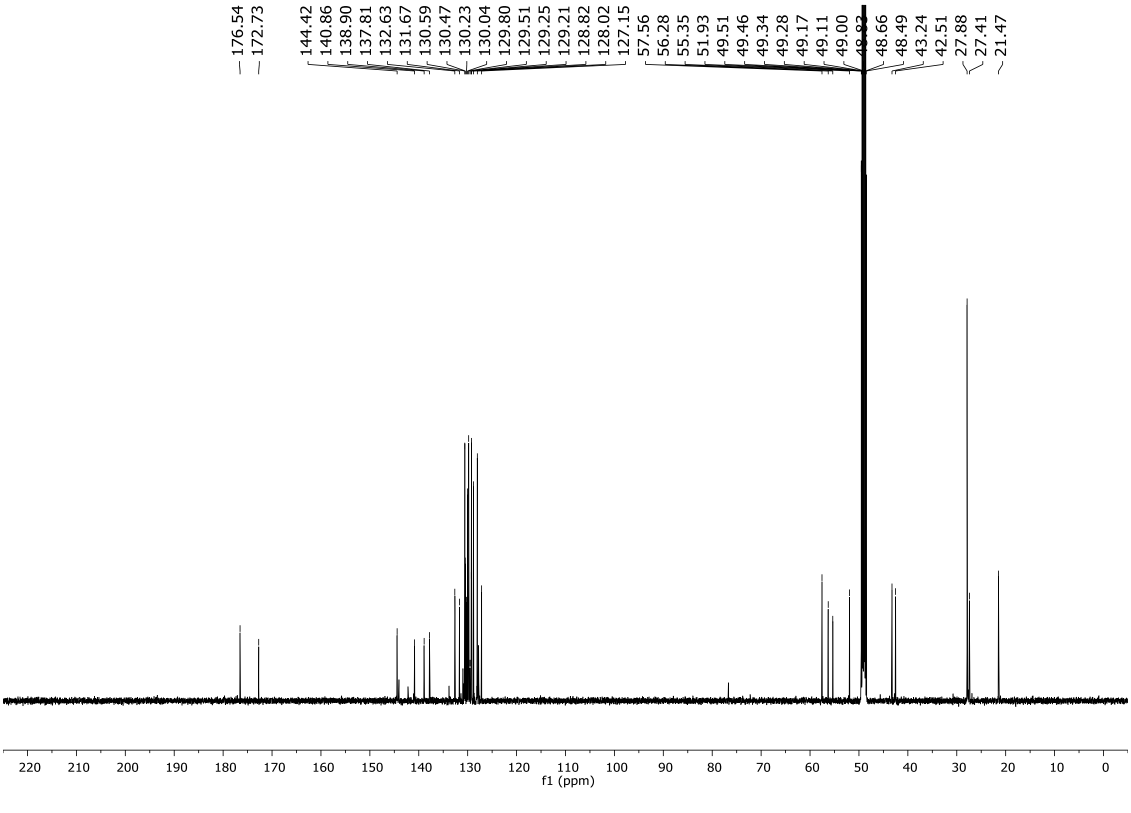

COSY

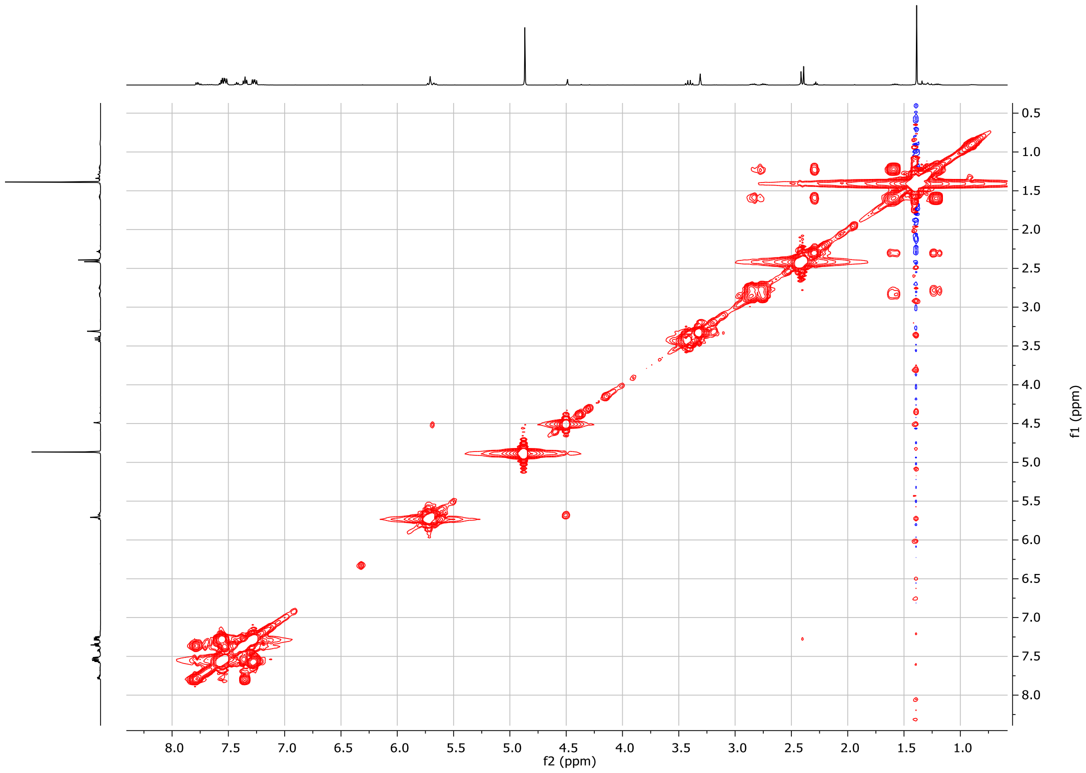

NOESY

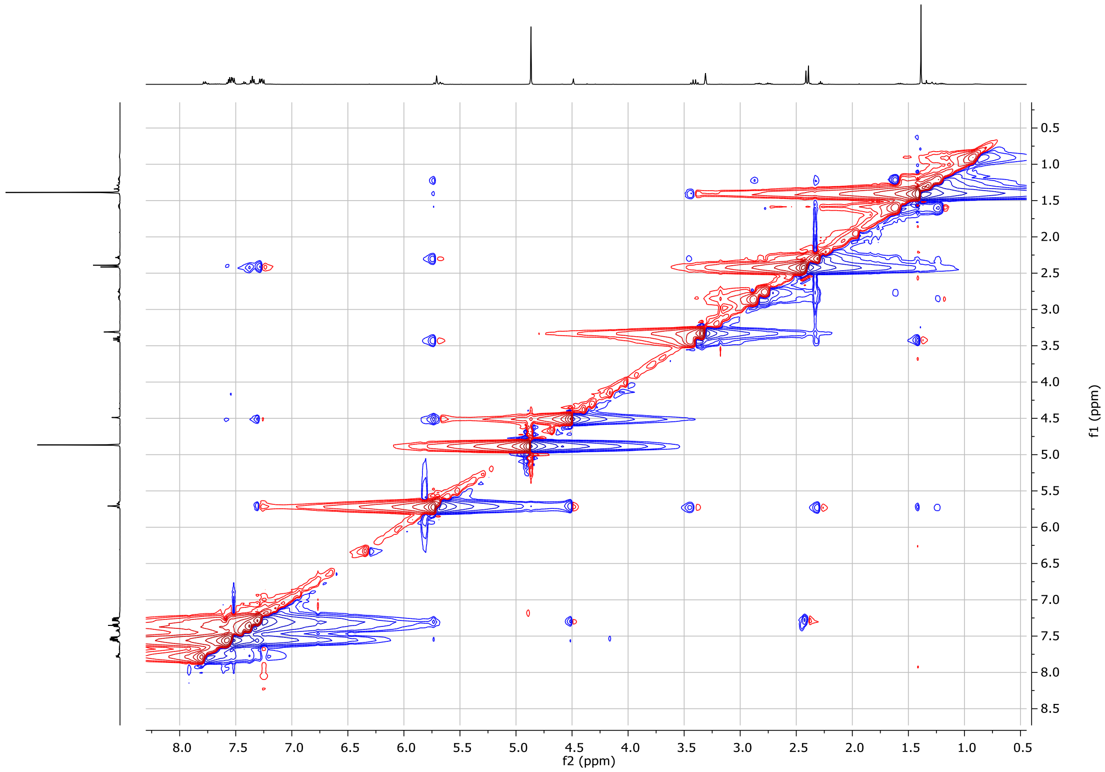

HSQC

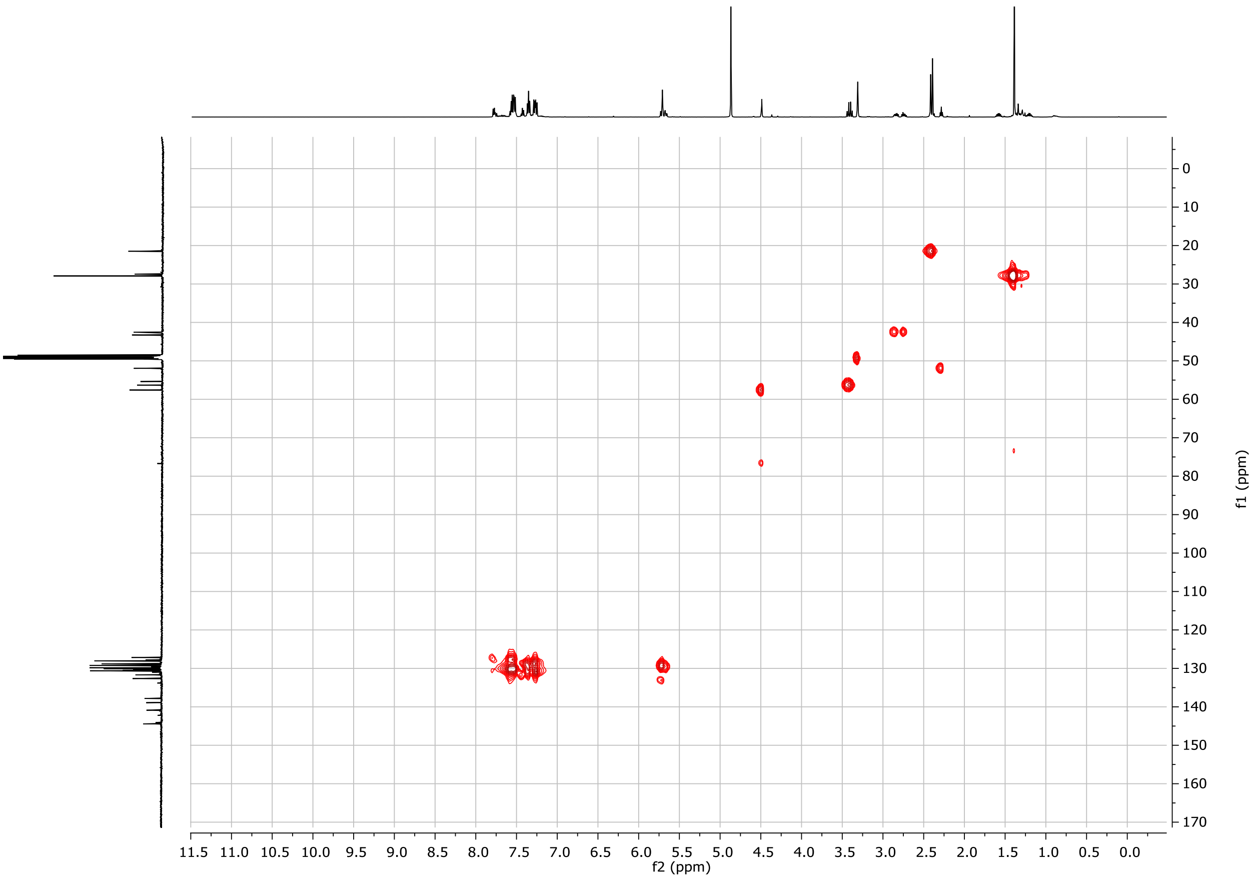

HMBC

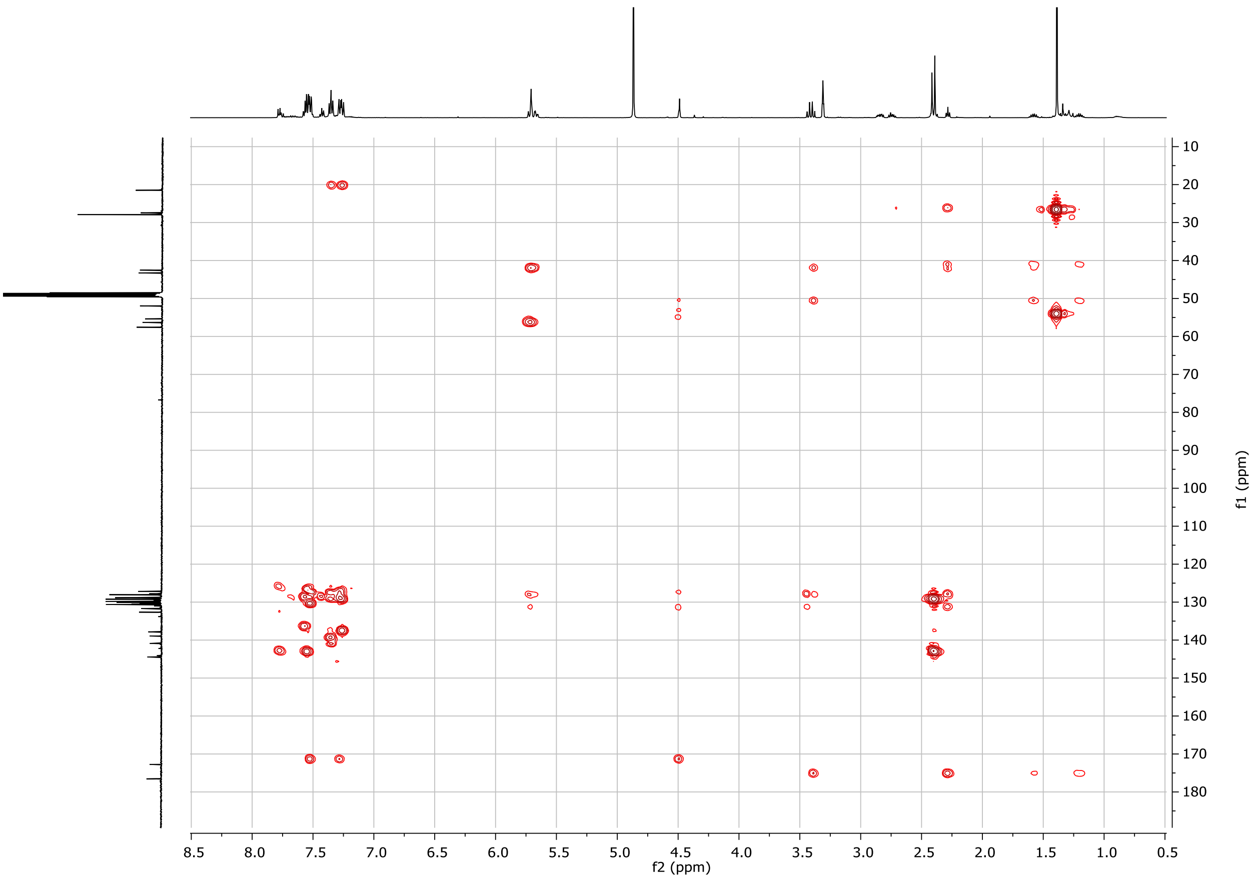

Minor isomer

^{1}H NMR (500 MHz, CD_3OD)

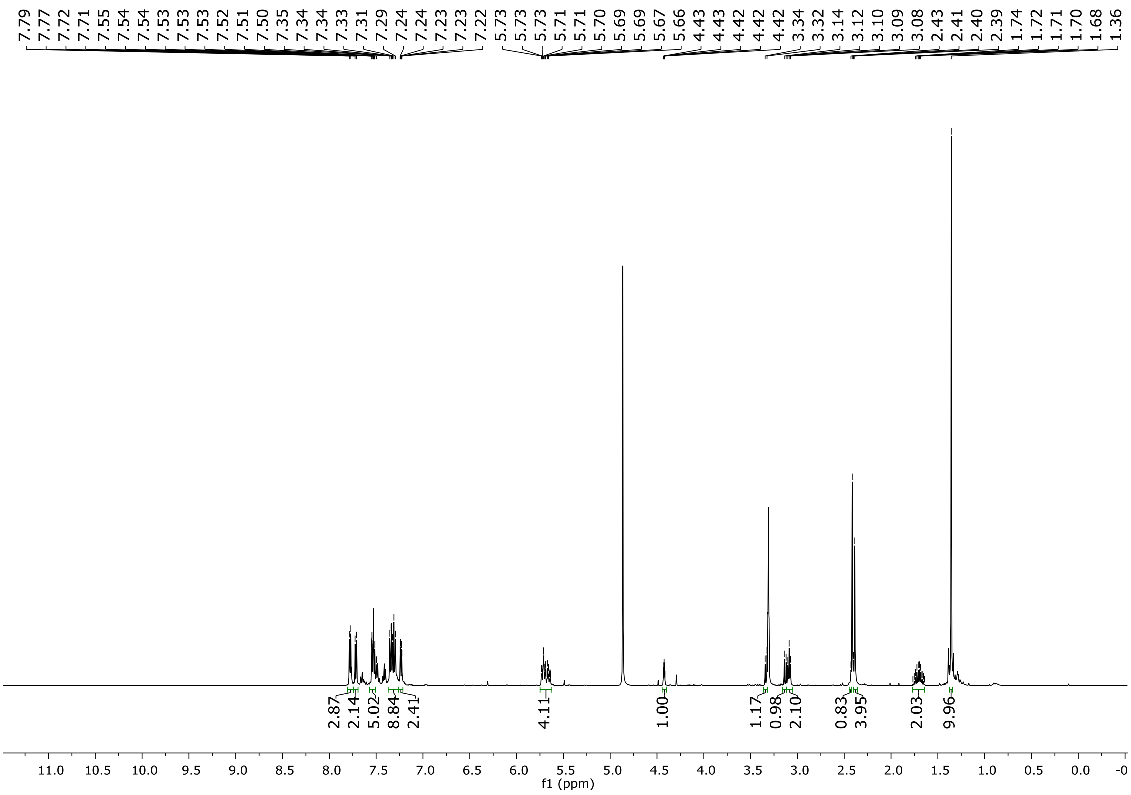

^{13}C NMR (126 MHz, CD_3OD)

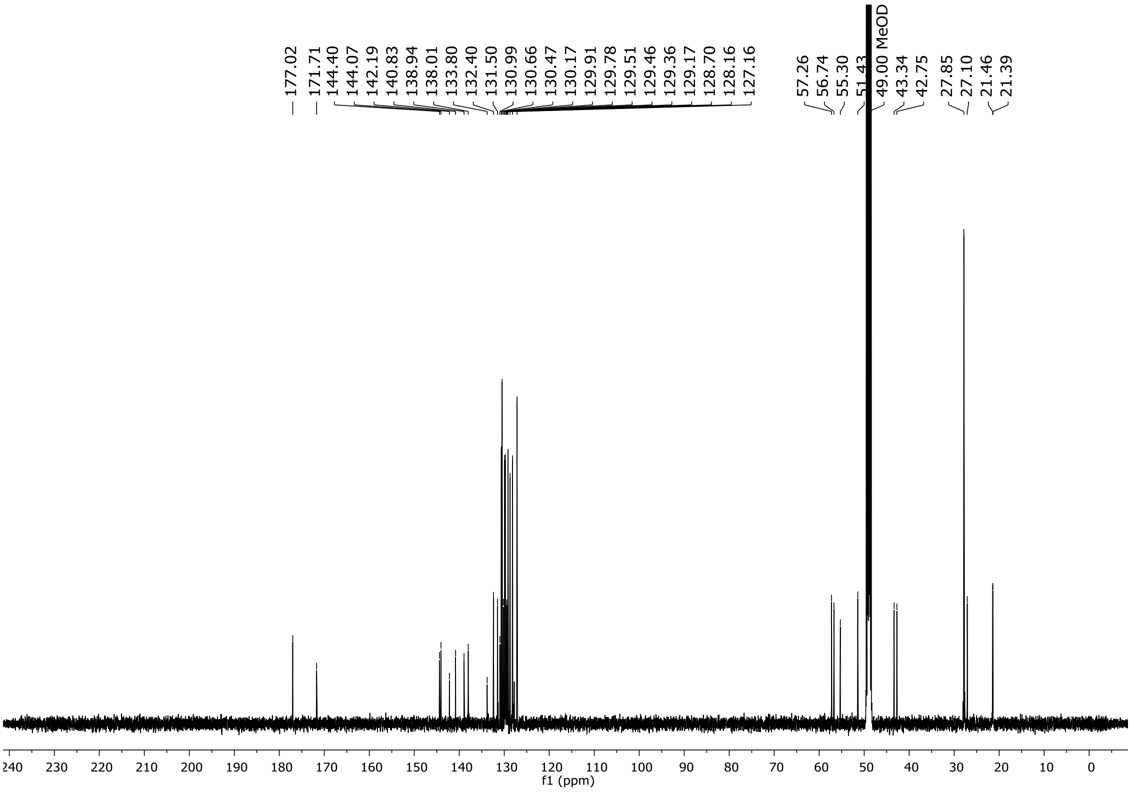

COSY

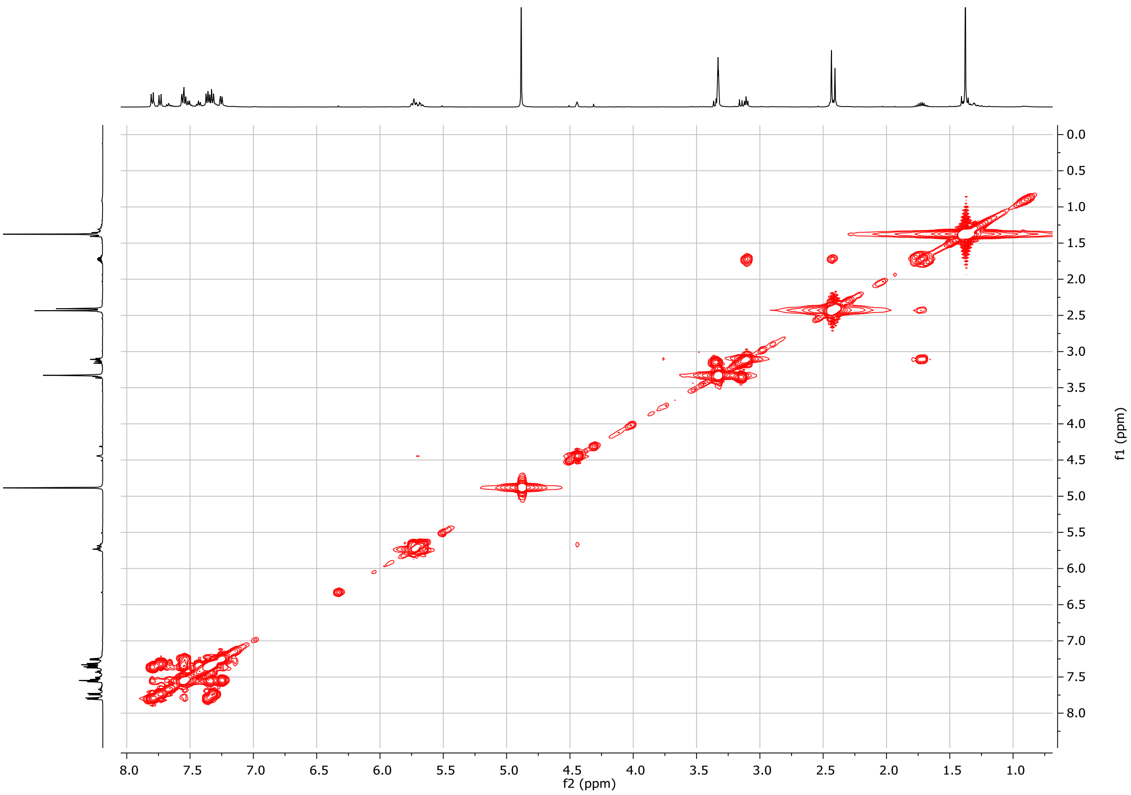

NOESY

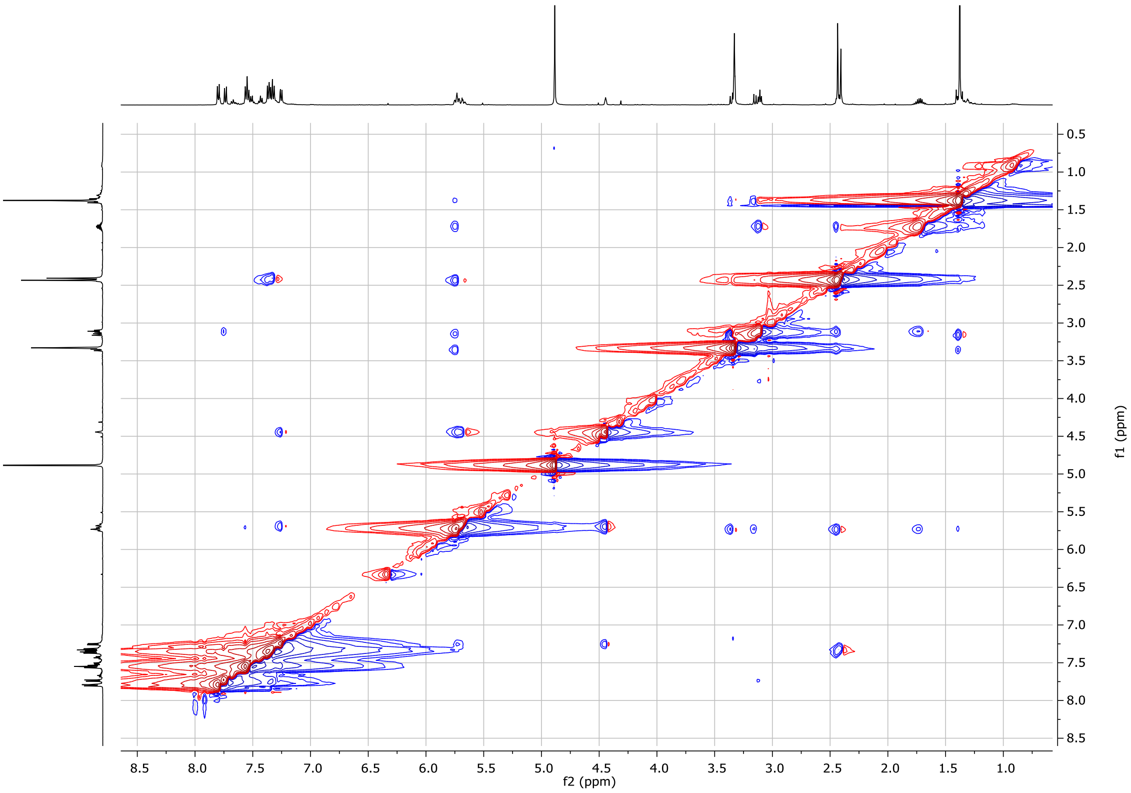

HSQC

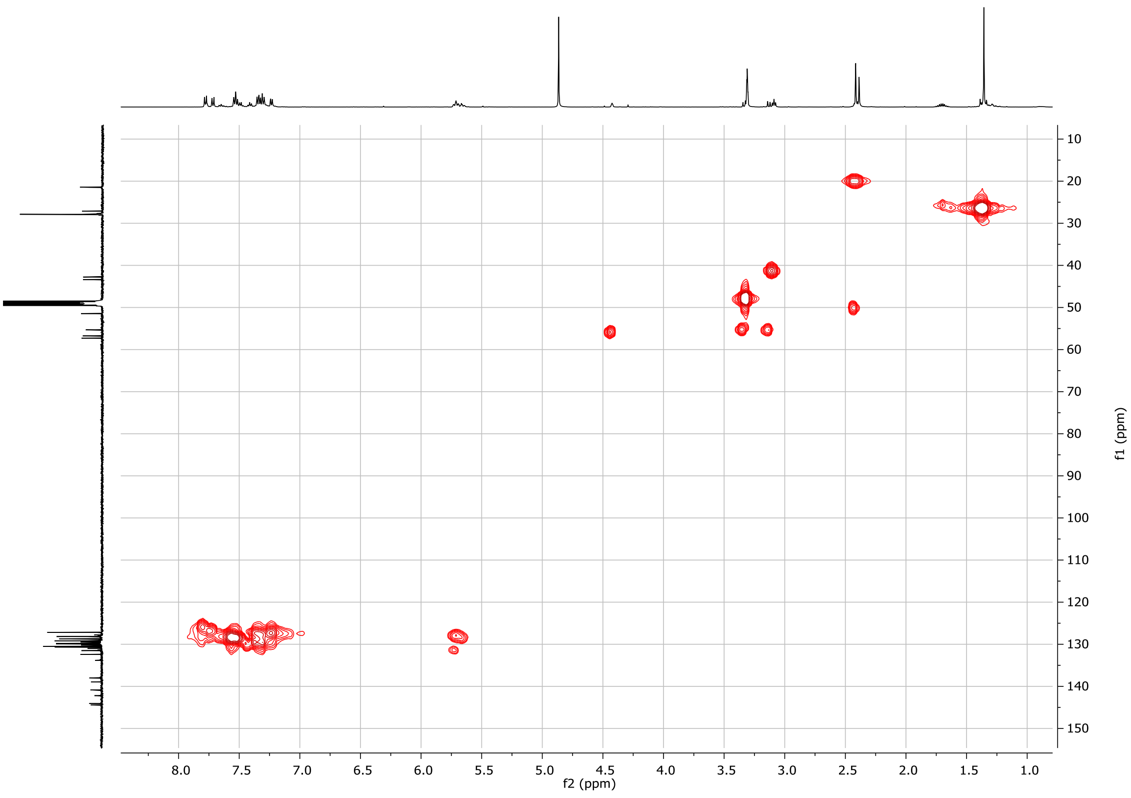

HMBC

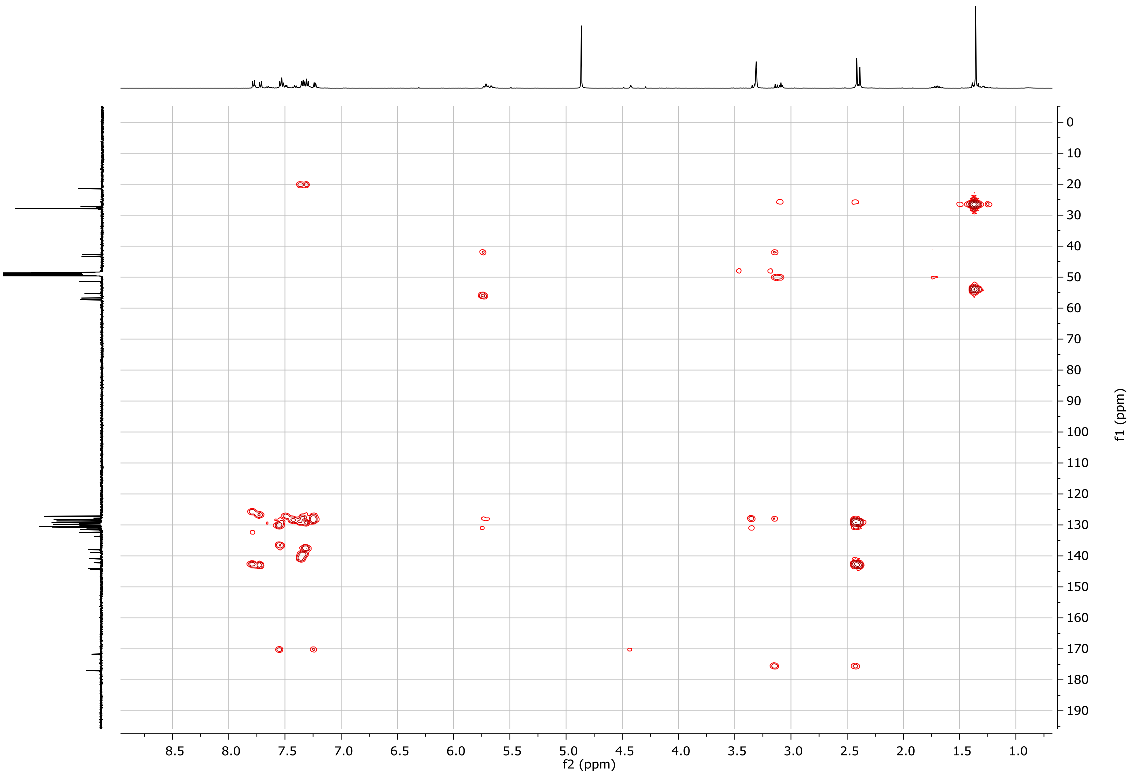

2-(*tert*-butyl)-8-((diphenylmethylene)amino)-4-((2,2,5,5-tetramethyltetrahydro-8aH-[1,3]dioxolo[4',5':4,5]furo[3,2-d][1,3]dioxin-8a-yl)methyl)-2-azaspiro[4.5]deca-6,9-dien-3-one (3ad).

^{1}H NMR (500 MHz, CD_3OD)

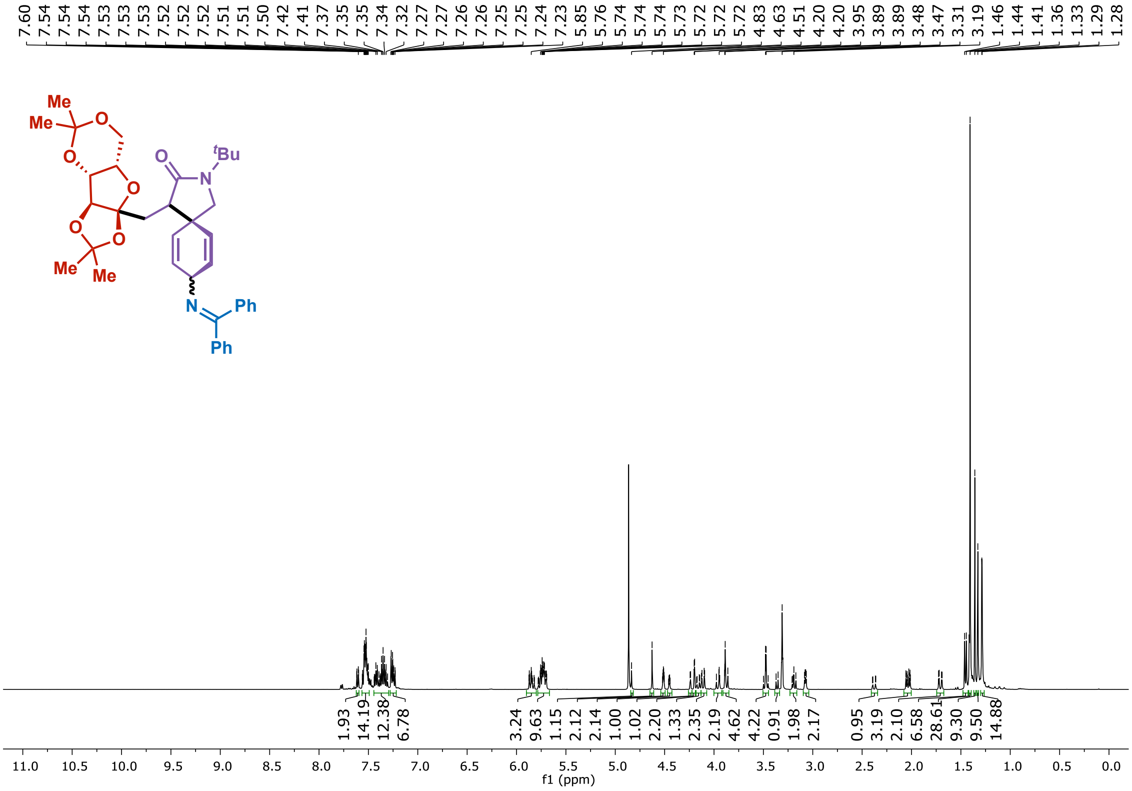

^{13}C NMR (126 MHz, CD_3OD)

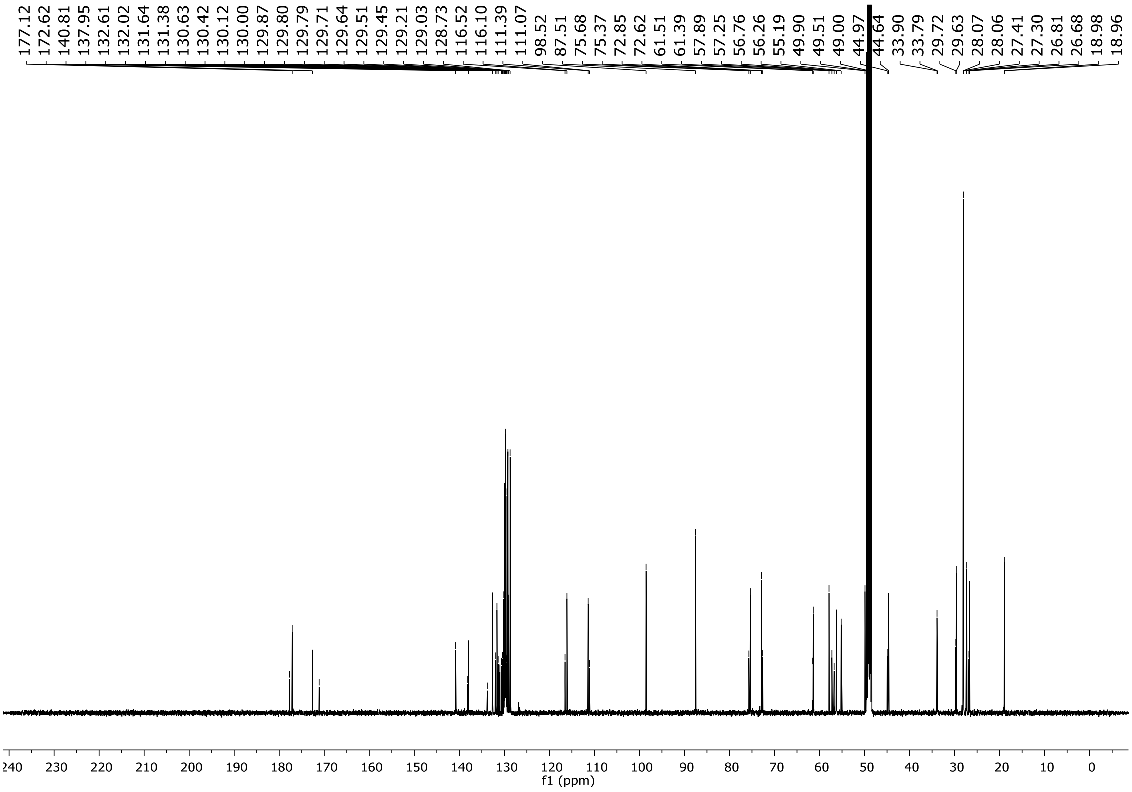

2-(tert-butyl)-8-((diphenylmethylene)amino)-2-azaspiro[4.5]deca-6,9-dien-3-one (3ae)

Isomer A

^{1}H NMR (500 MHz, CD_3OD)

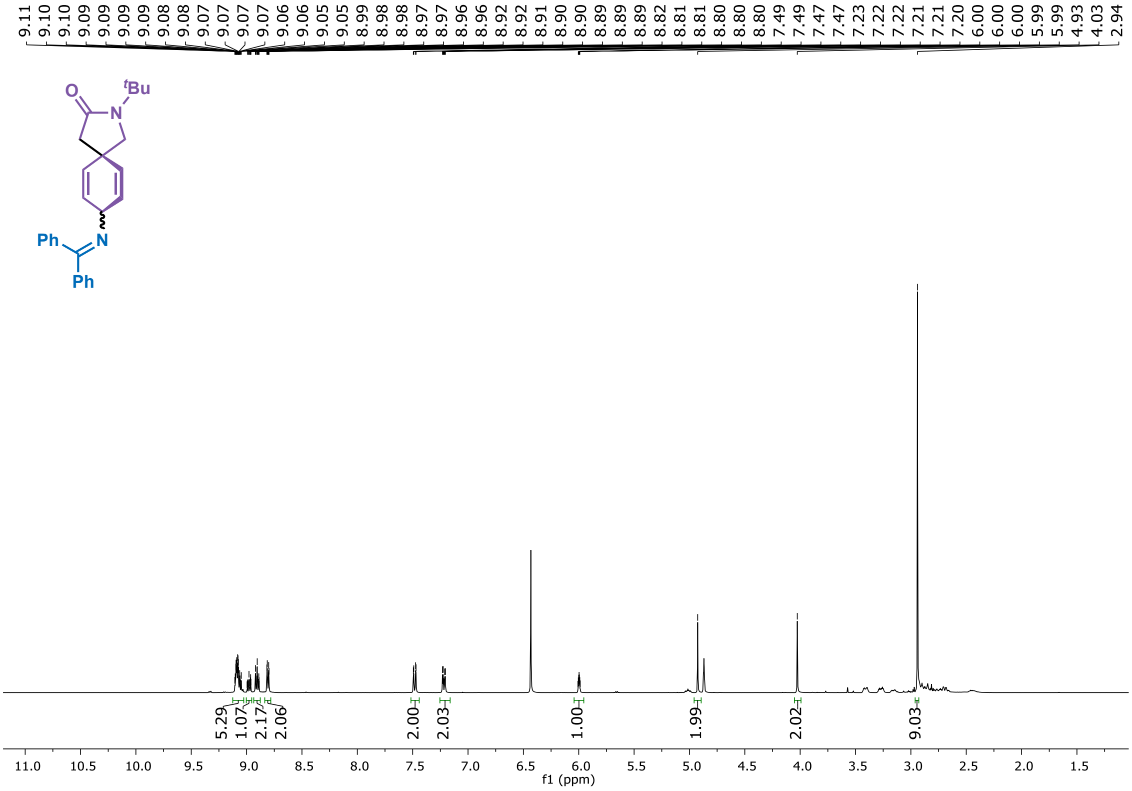

^{13}C NMR (126 MHz, CD_3OD)

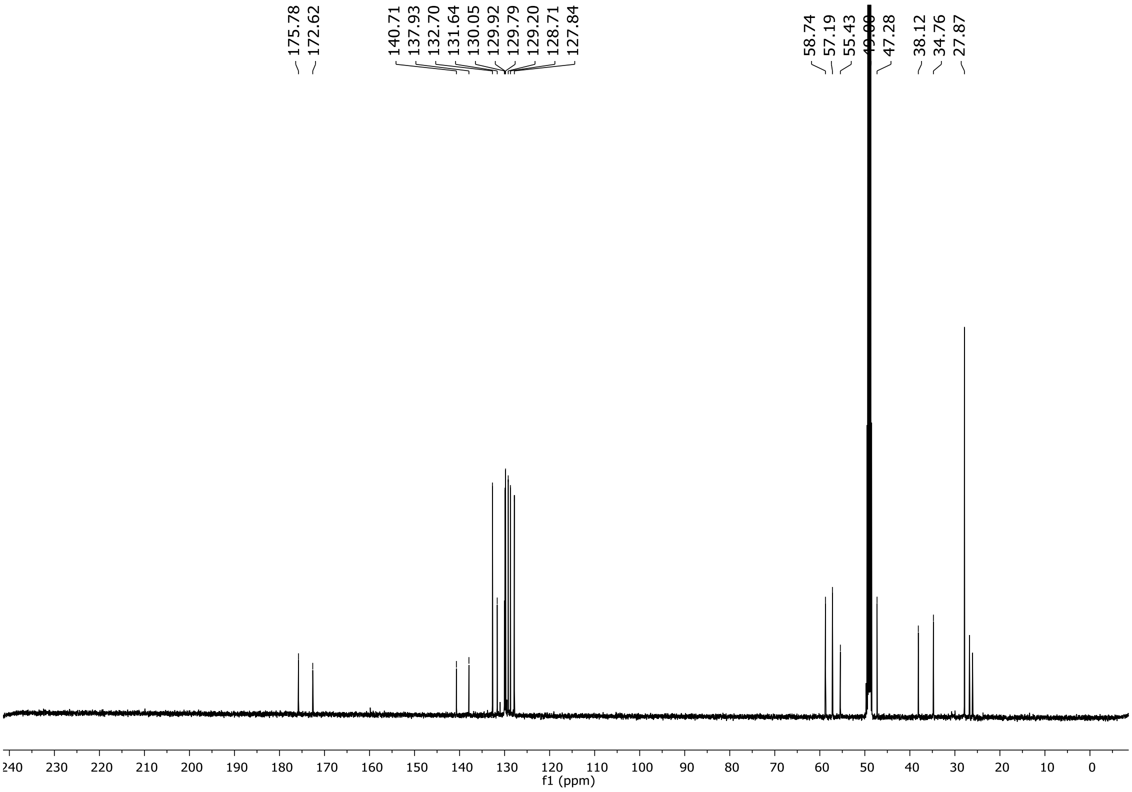

COSY

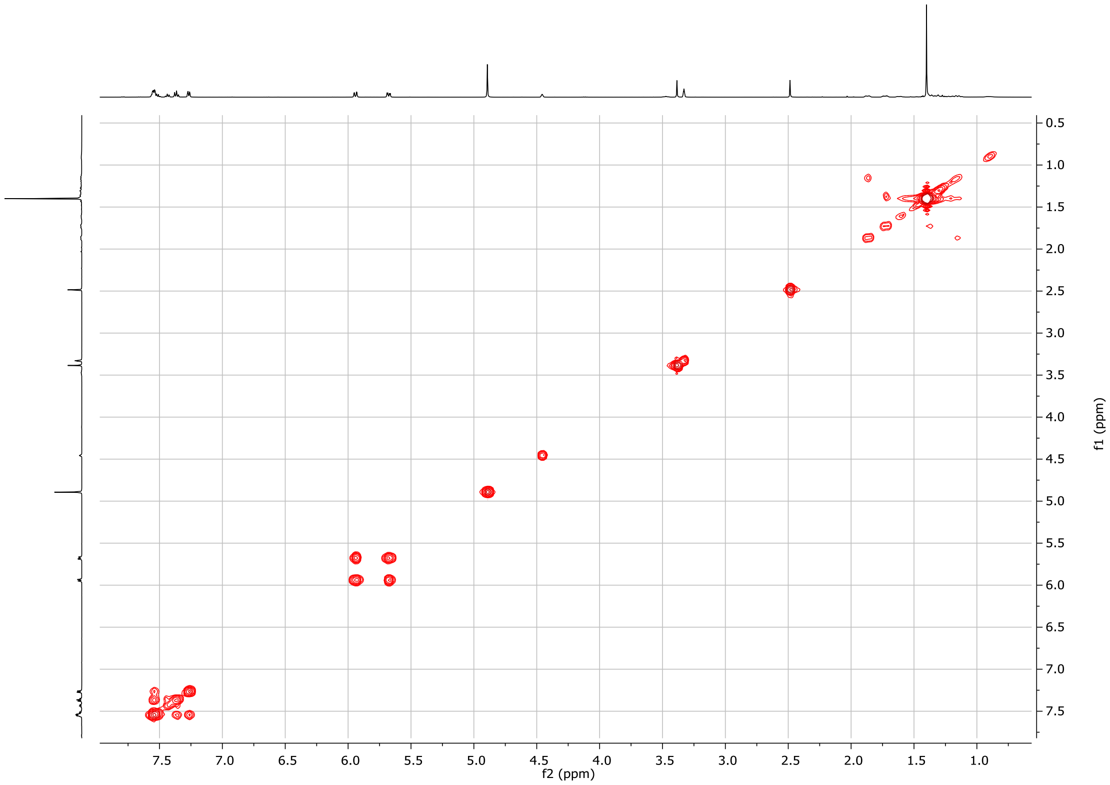

NOESY

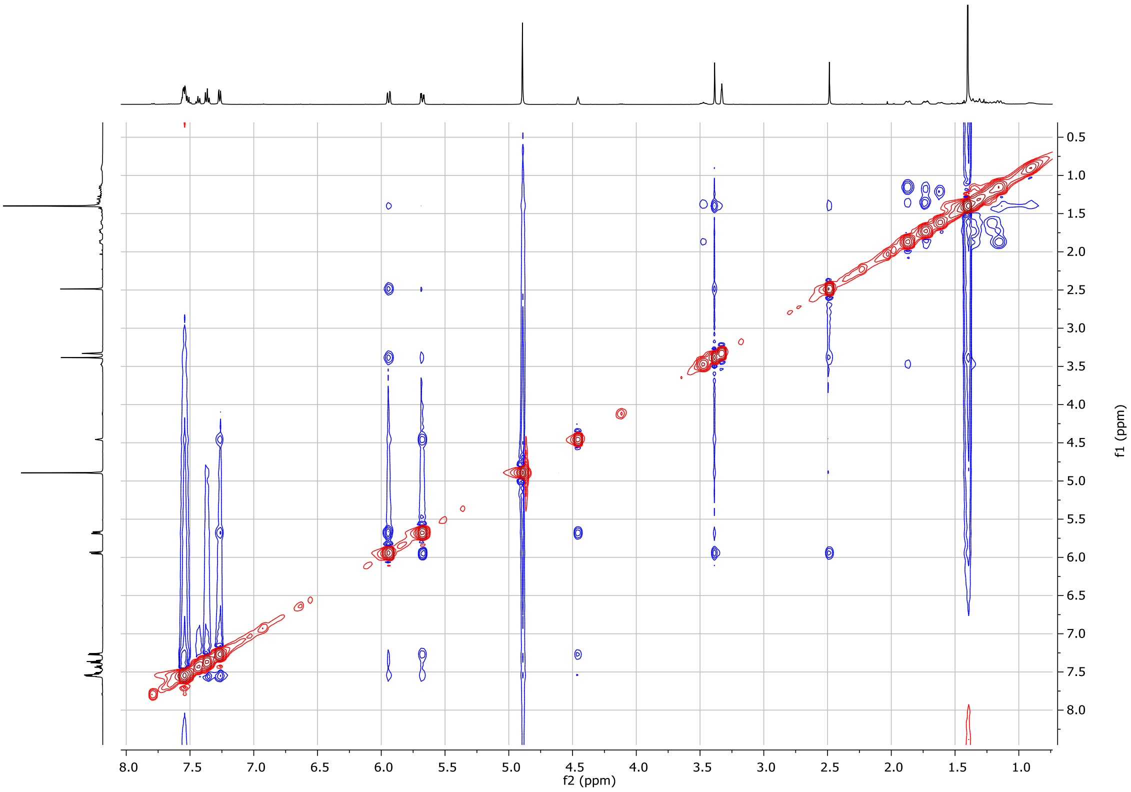

HSQC

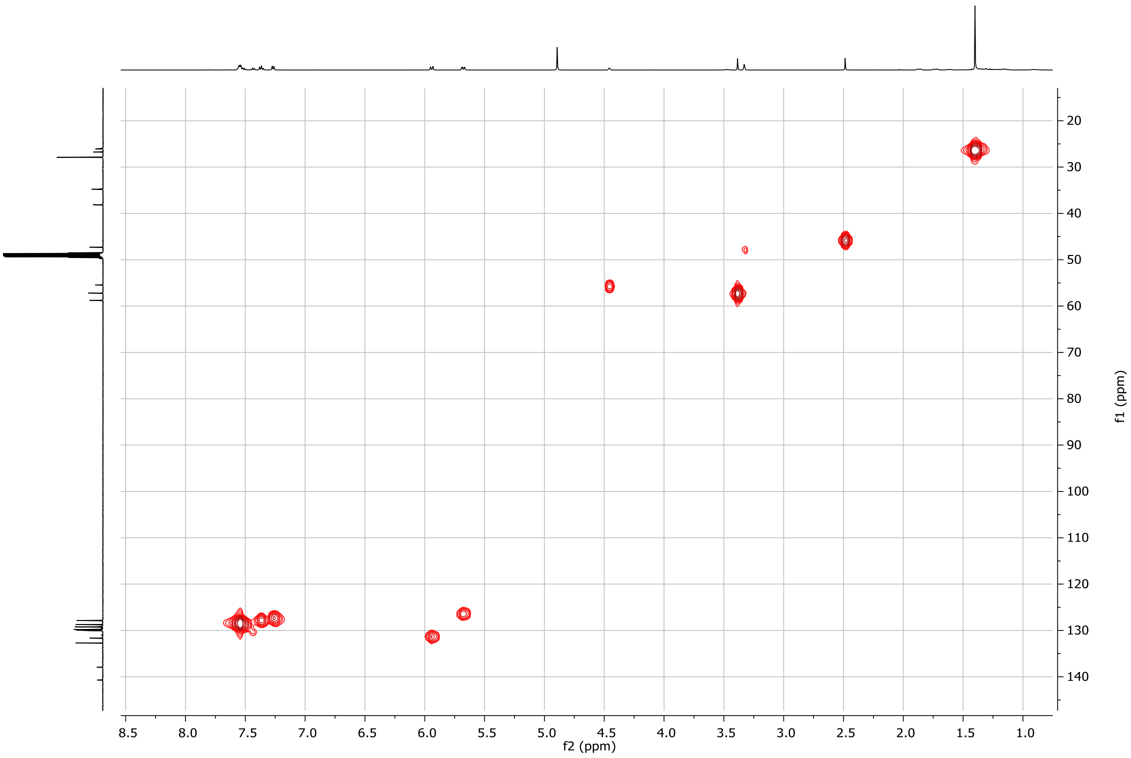

HMBC

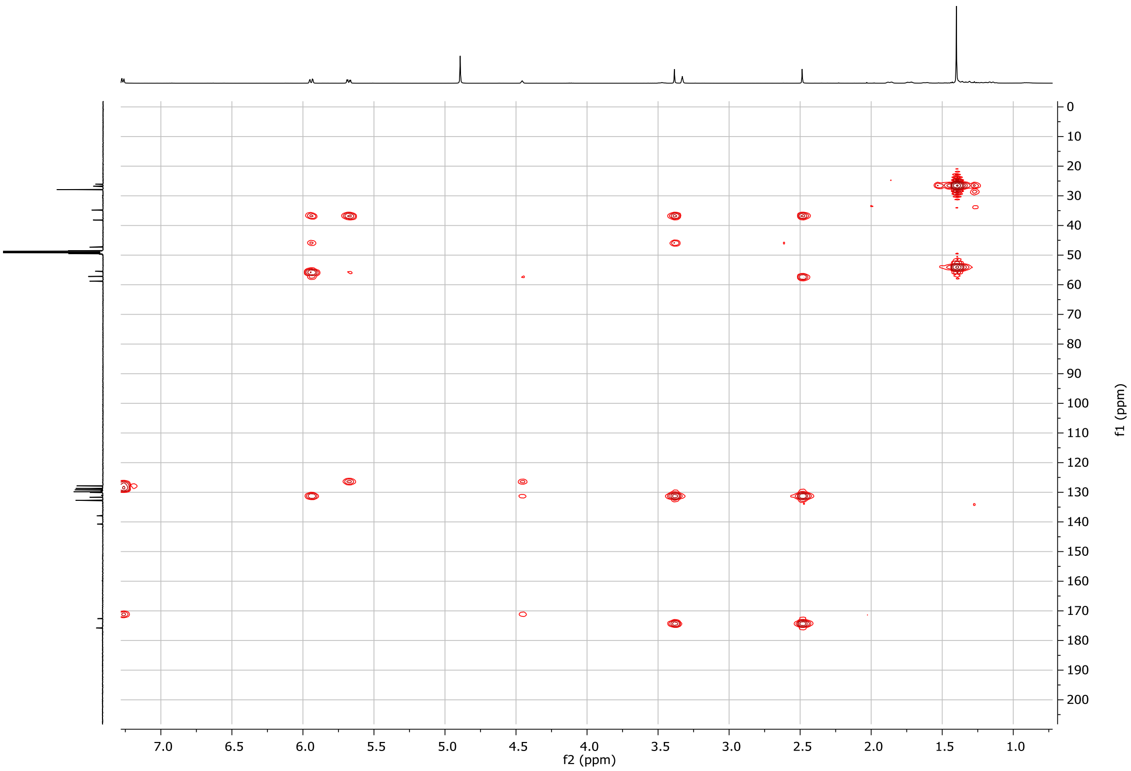

Isomer B

^{1}H NMR (500 MHz, CD_3OD)

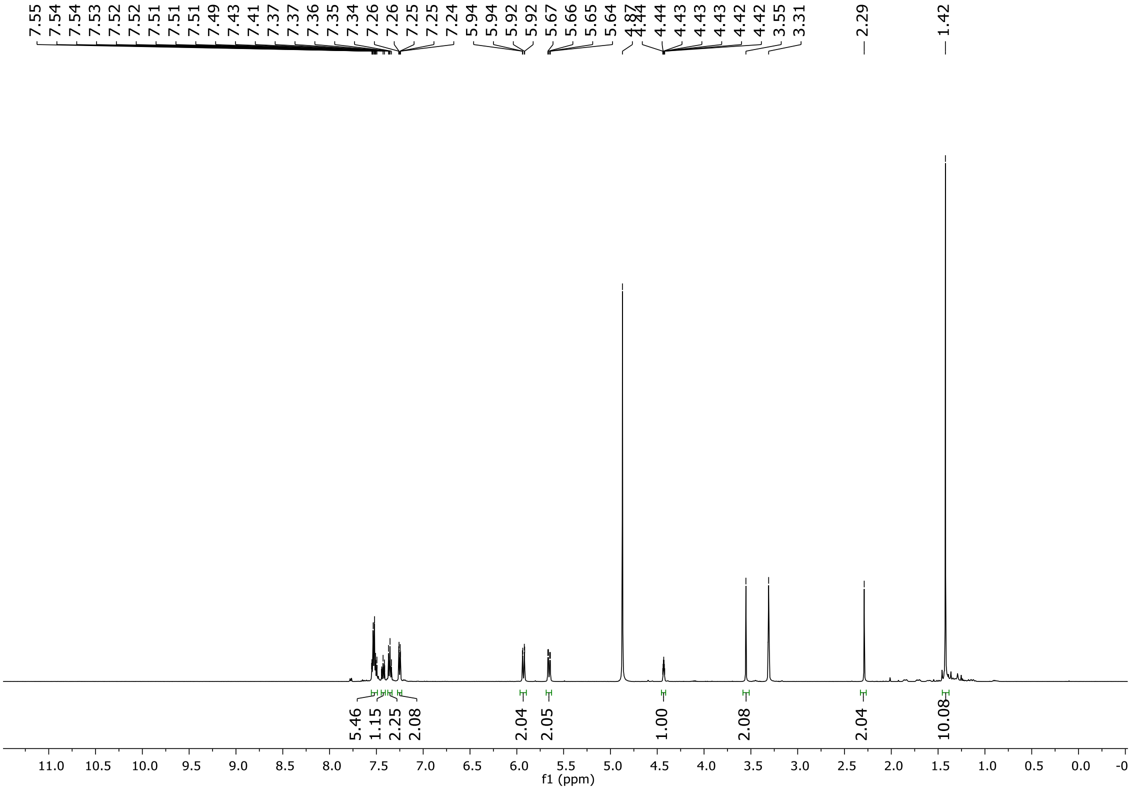

^{13}C NMR (126 MHz, CD_3OD)

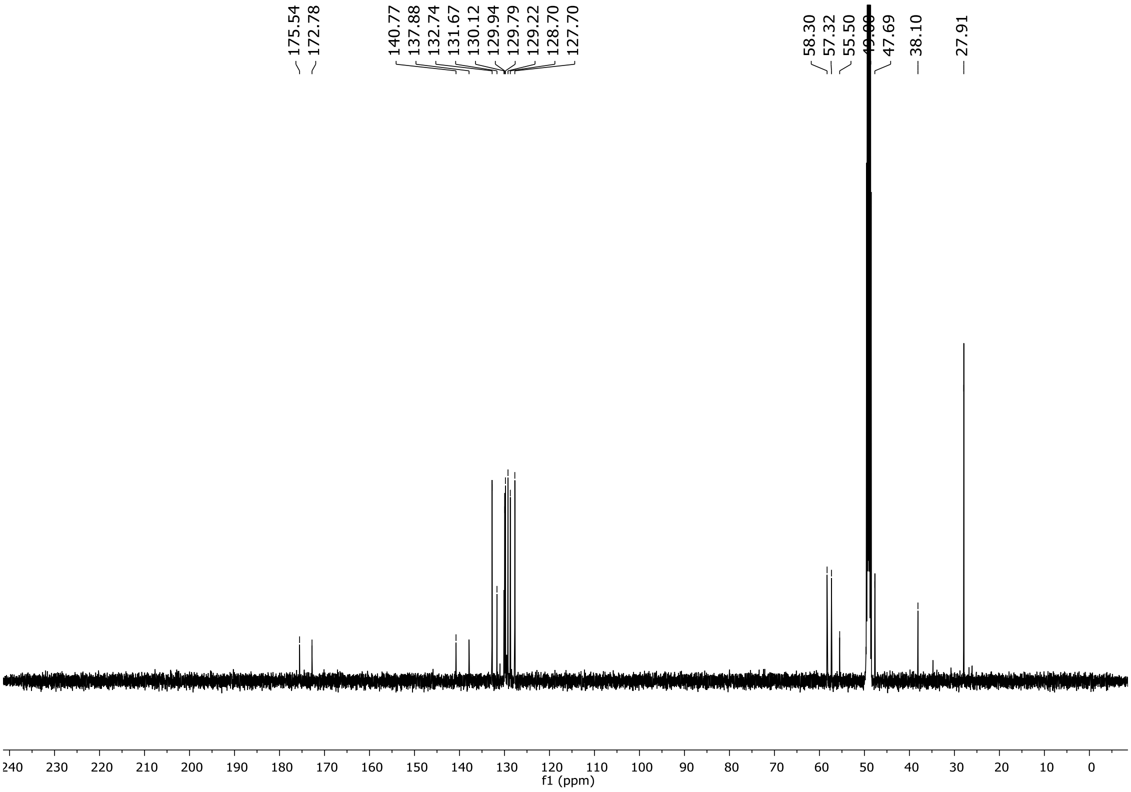

COSY

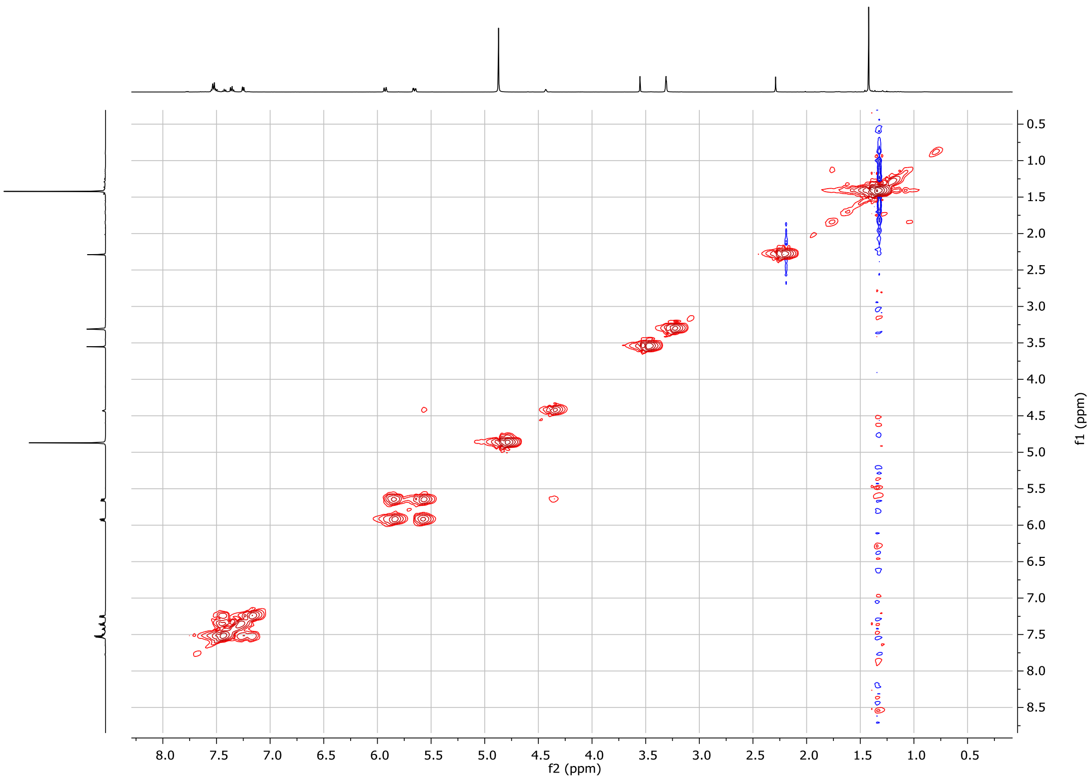

HSQC

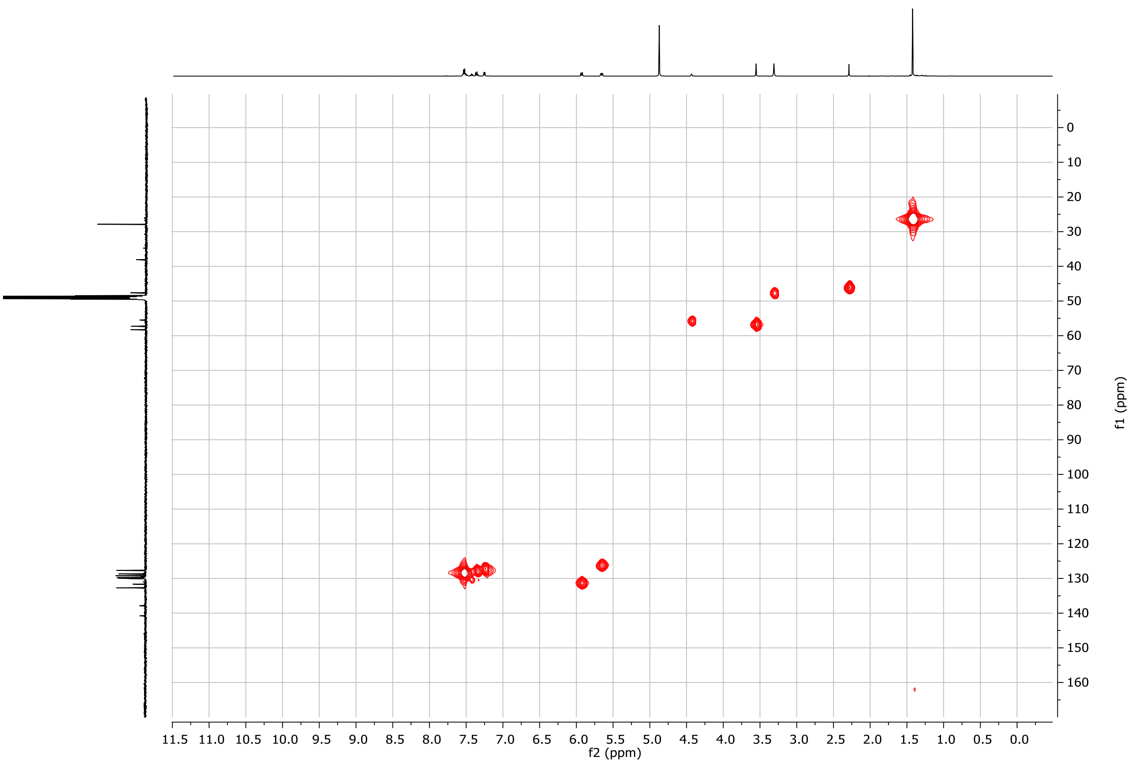

Methyl(2r,4r)-12-(tert-butyl)-8-((diphenylmethylene)amino)-13-oxo-12-azadispiro[3.0.55.34]trideca-6,9-diene-2-carboxylate (3af)

^{1}H NMR (400 MHz, CD_3OD)

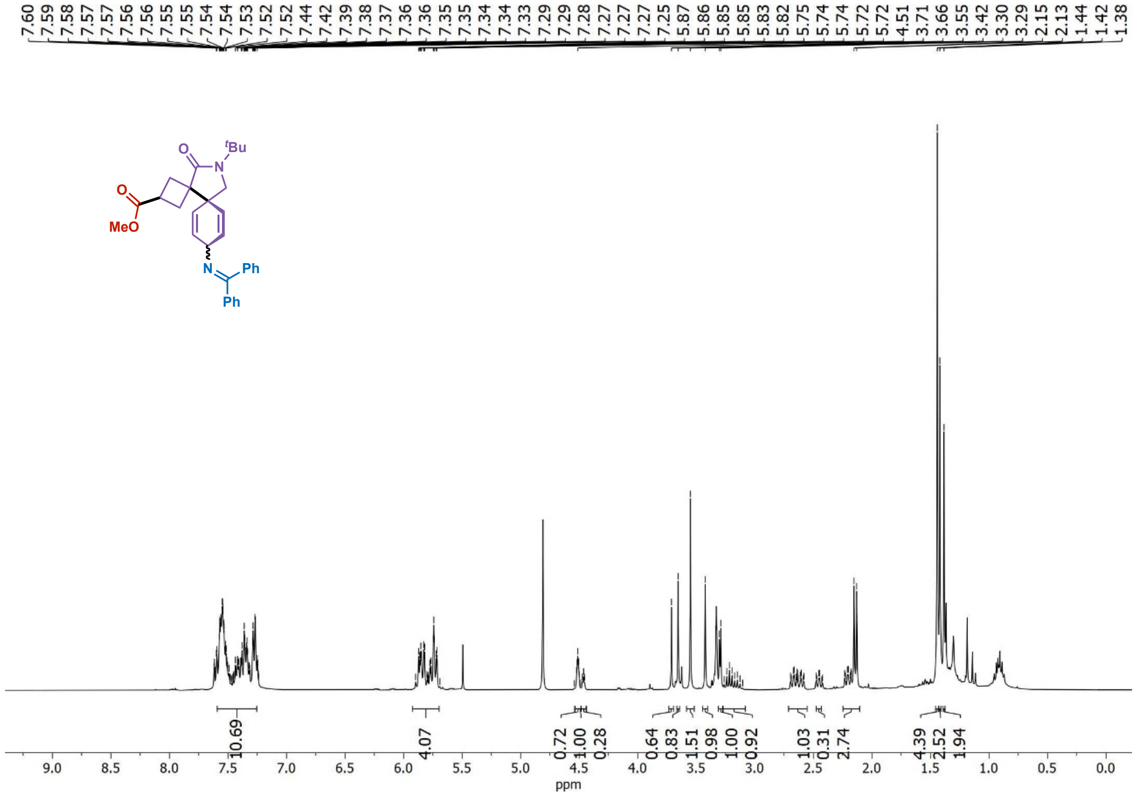

^{13}C NMR (101 MHz, CD_3OD)

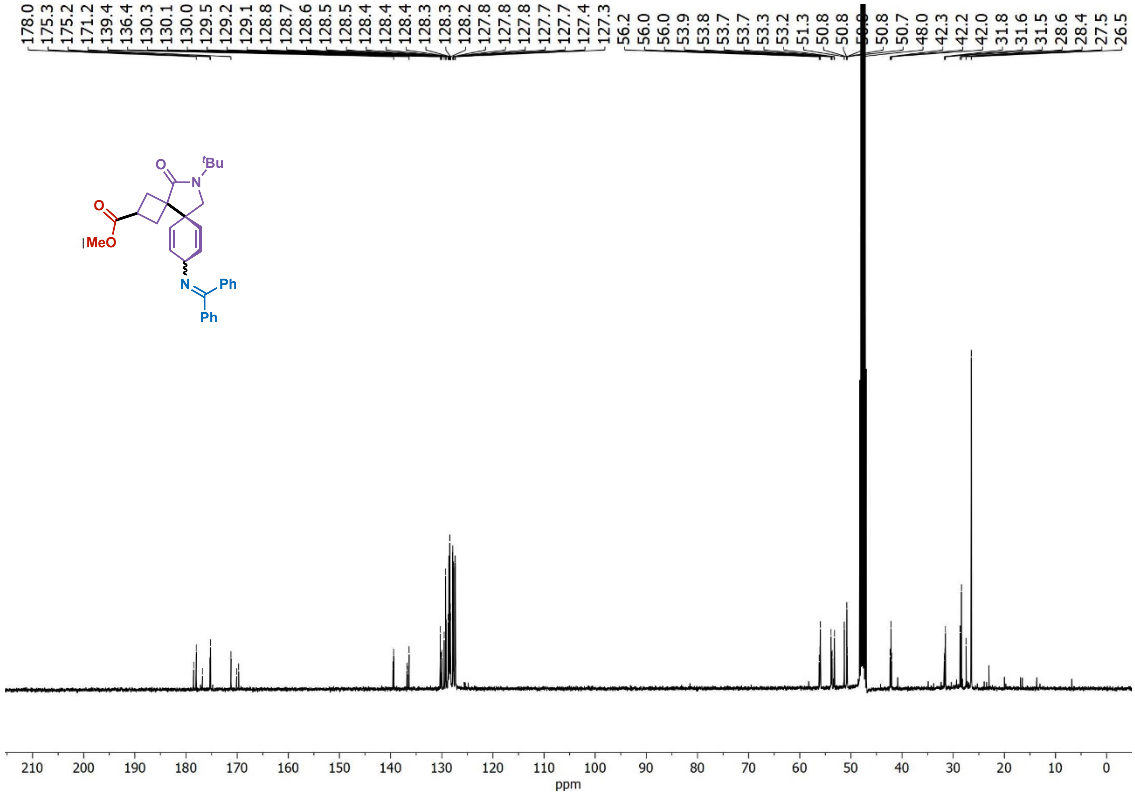

(1-((2-(tert-Butyl)-8-((diphenylmethylene)amino)-3-oxo-2-azaspiro[4.5]deca-6,9-dien-4-yl)methyl)cyclohexyl)methyl methyl carbonate (6a)

^{1}H NMR (500 MHz, CD_3OD)

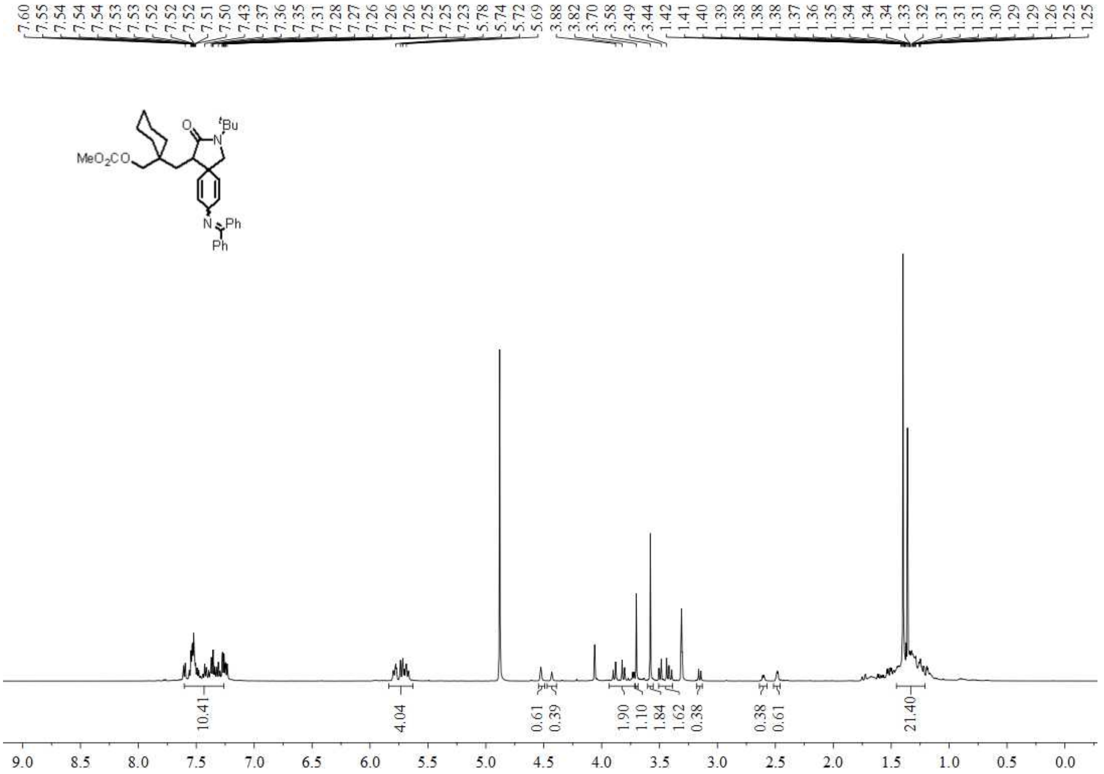

^{13}C NMR (126 MHz, CD_3OD)

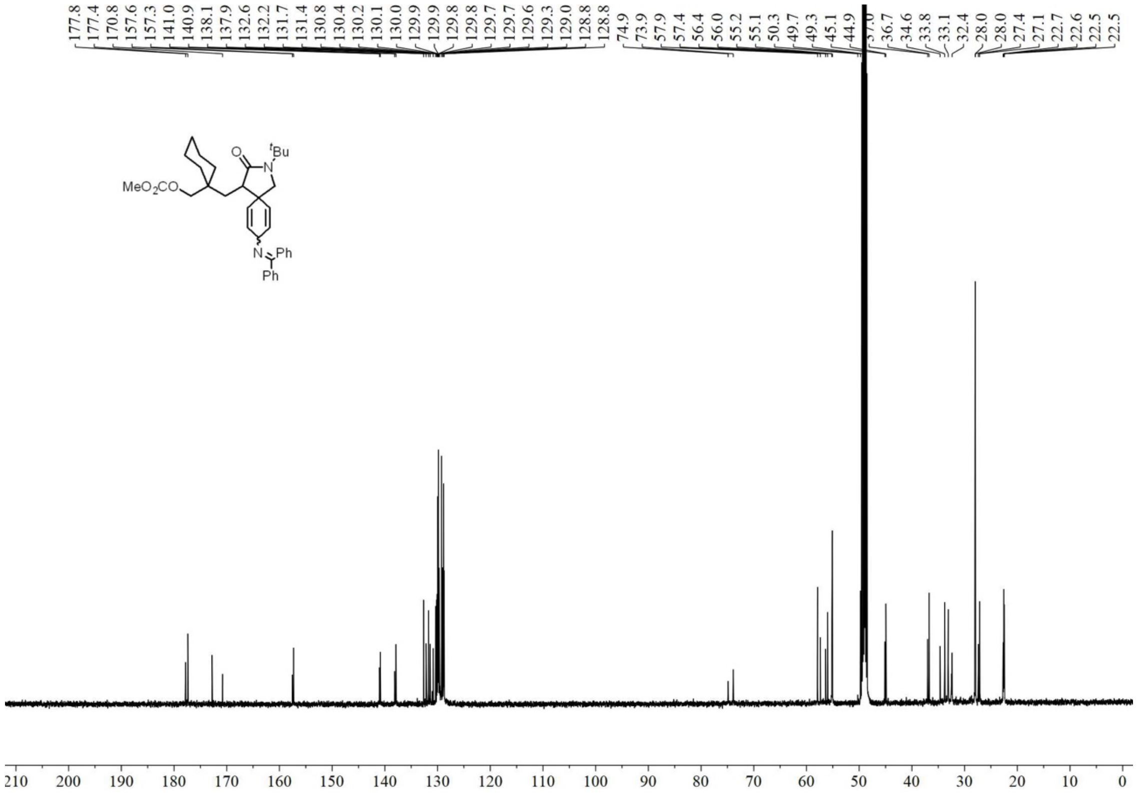

(1-((2-(*tert*-Butyl)-8-((diphenylmethylene)amino)-3-oxo-2-azaspiro[4.5]deca-6,9-dien-4-yl)methyl)cyclohexyl)methyl ethyl carbonate (6b)

^{1}H NMR (400 MHz, CD_3OD)

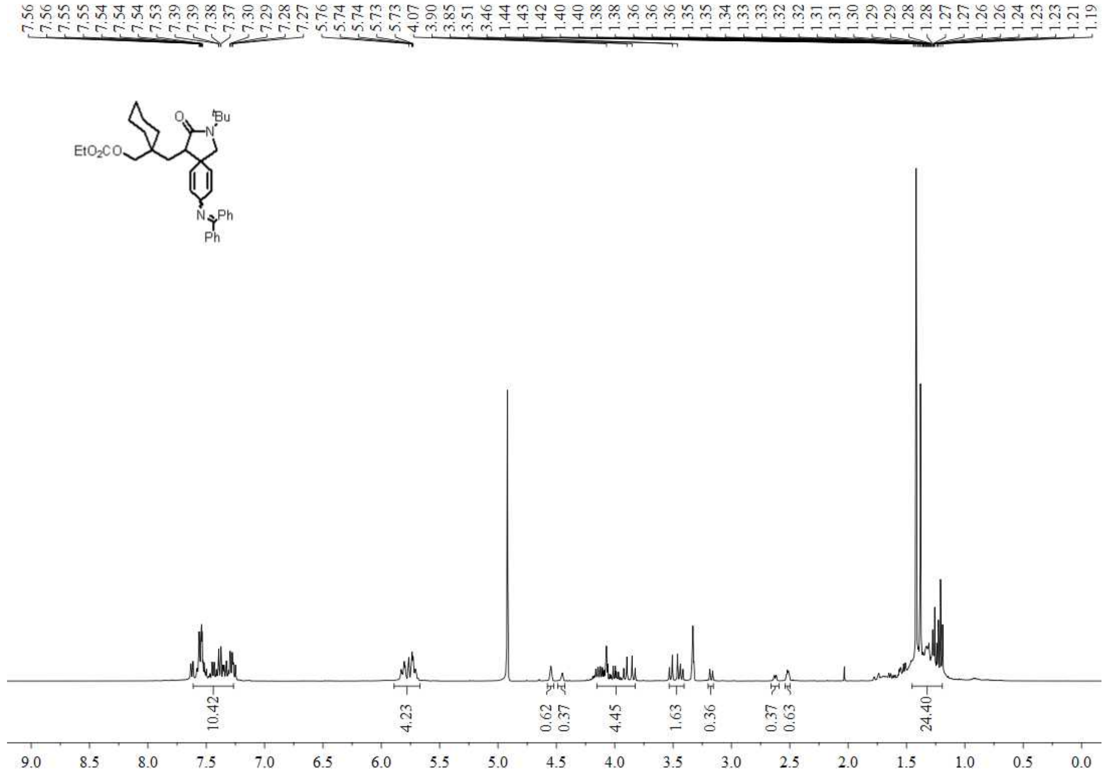

^{13}C NMR (101 MHz, CD_3OD)

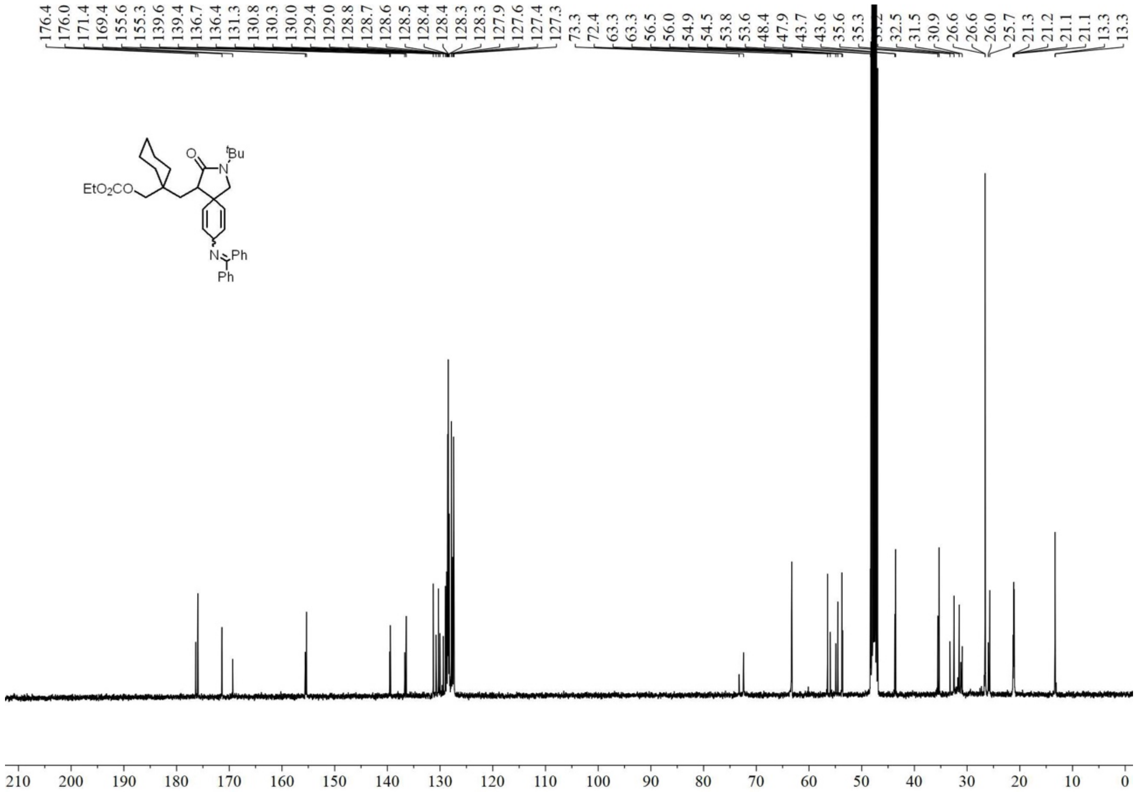

Butyl ((1-((2-(*tert*-butyl)-8-((diphenylmethylene)amino)-3-oxo-2-azaspiro[4.5]deca-6,9-dien-4-yl)methyl)cyclohexyl)methyl) carbonate (6c)

^{1}H NMR (400 MHz, CD_3OD)

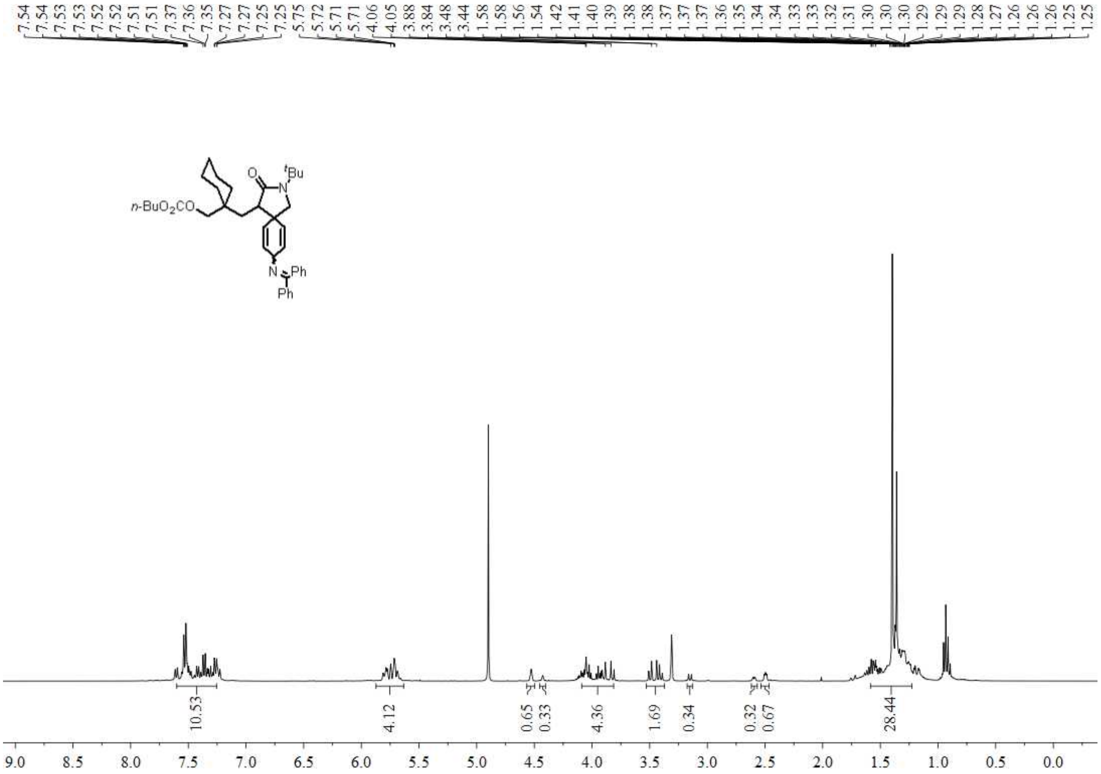

^{13}C NMR (101 MHz, CD_3OD)

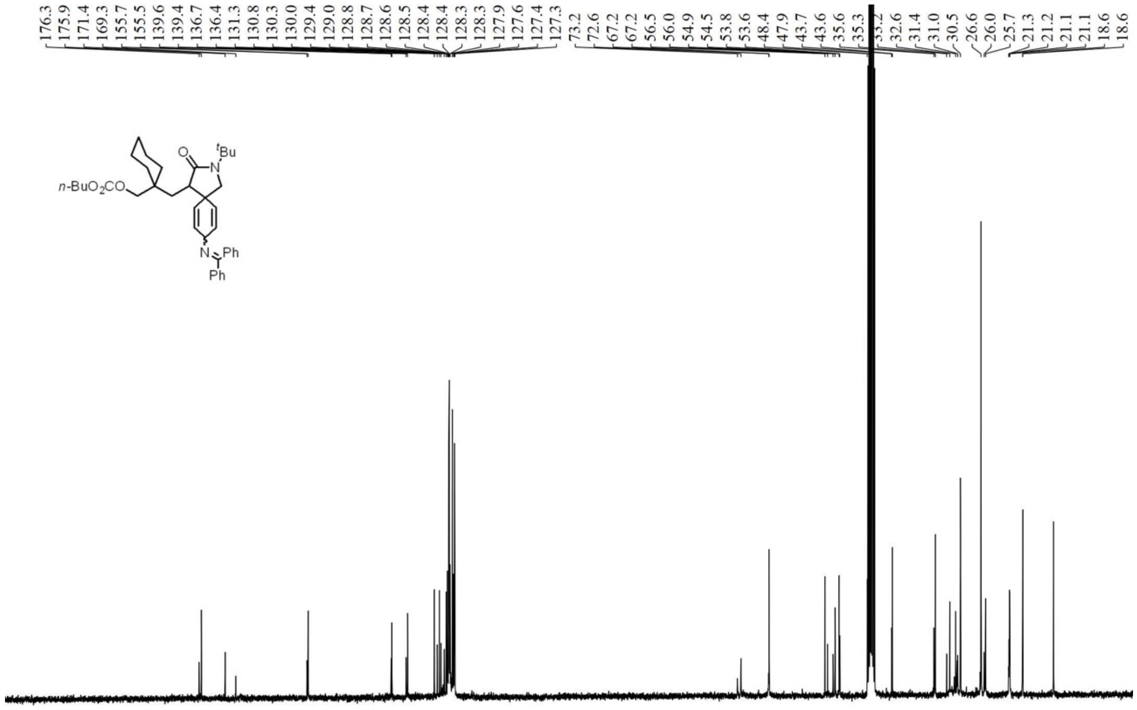

(4-((2-(*tert*-Butyl)-8-((diphenylmethylene)amino)-3-oxo-2-azaspiro[4.5]deca-6,9-dien-4-yl)methyl)tetrahydro-2H-pyran-4-yl)methyl methyl carbonate (6d)

Major product 6d

^{1}H NMR (500 MHz, CD_3OD)

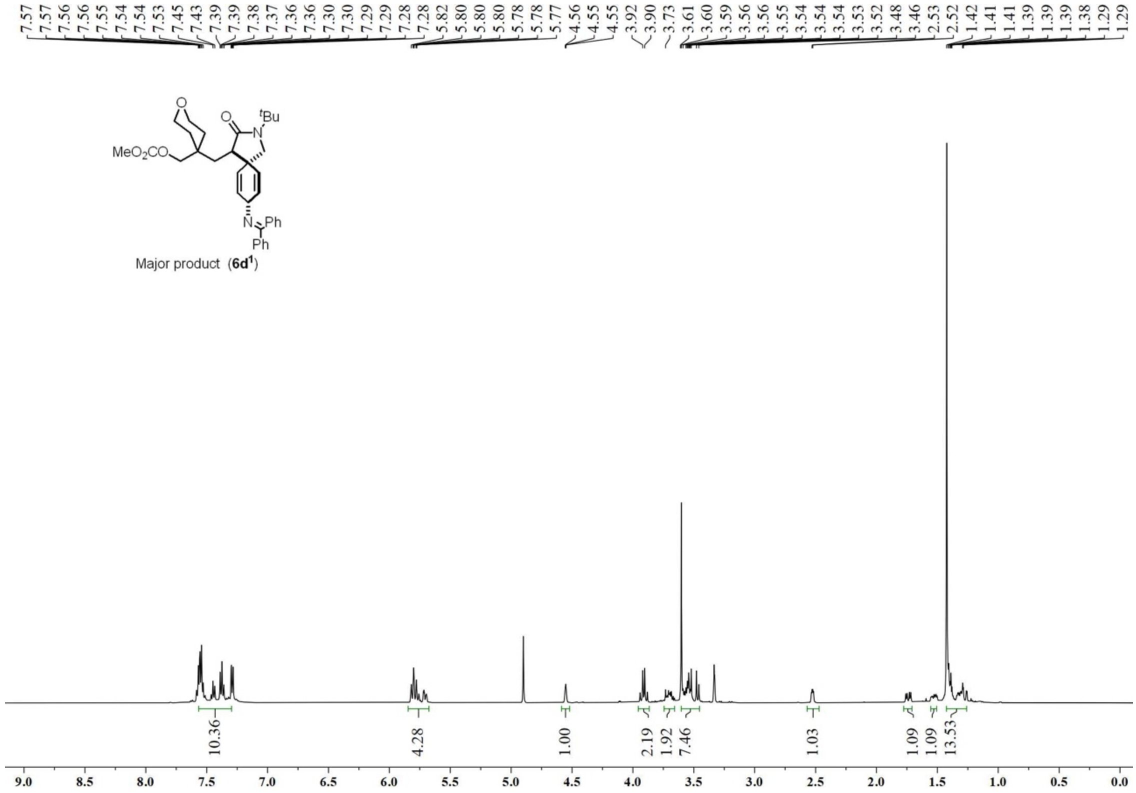

^{13}C NMR (126 MHz, CD_3OD)

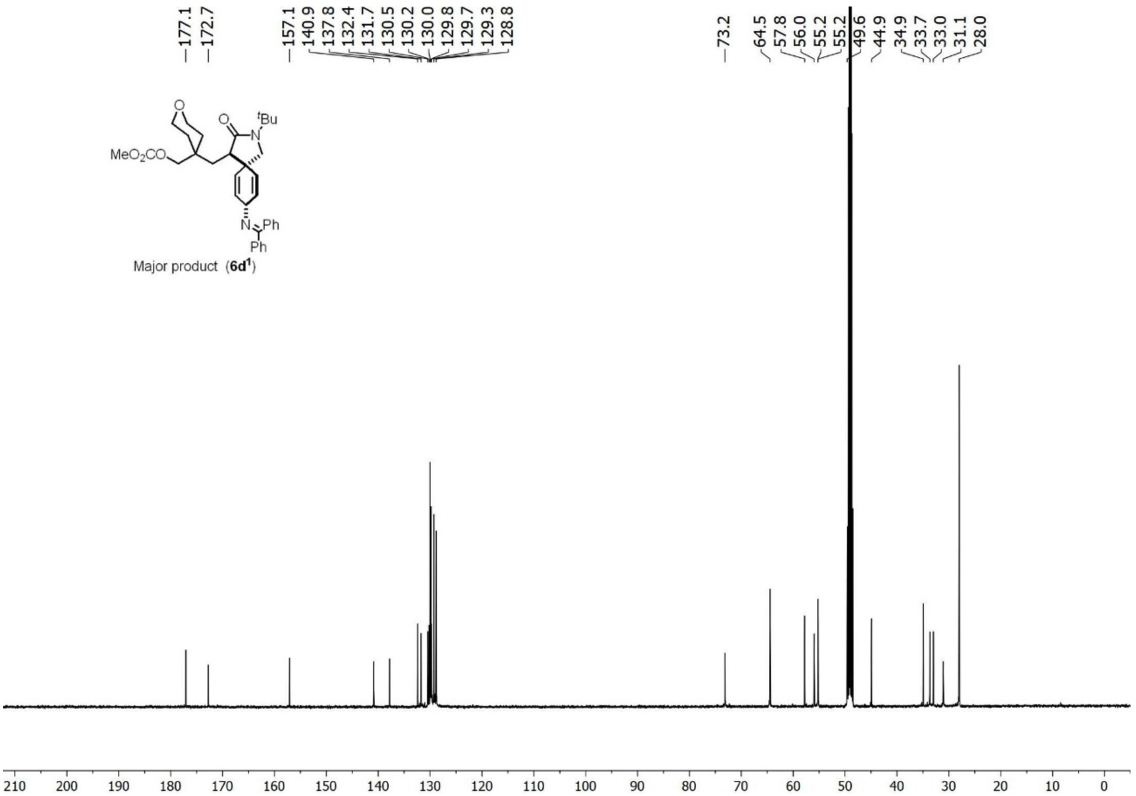

Minor product 6d

^{1}H NMR (500 MHz, Acetone-d_6)

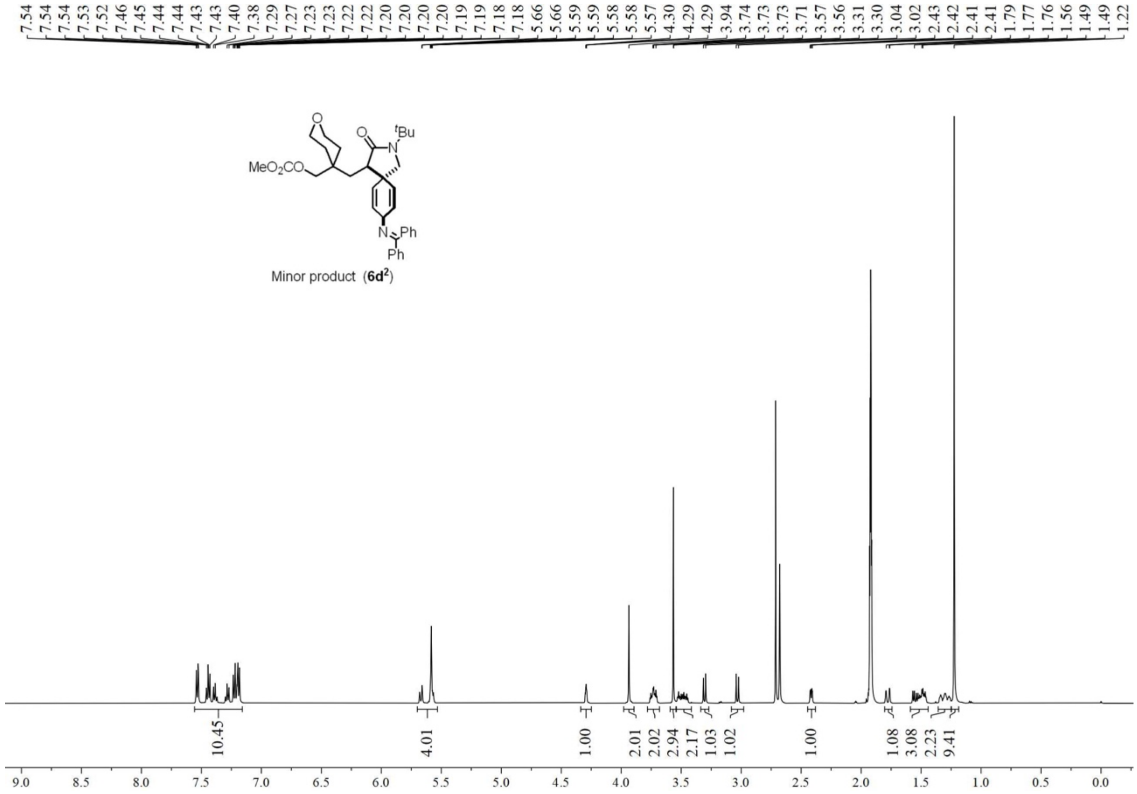

^{13}C NMR (126 MHz, Acetone-d_6)

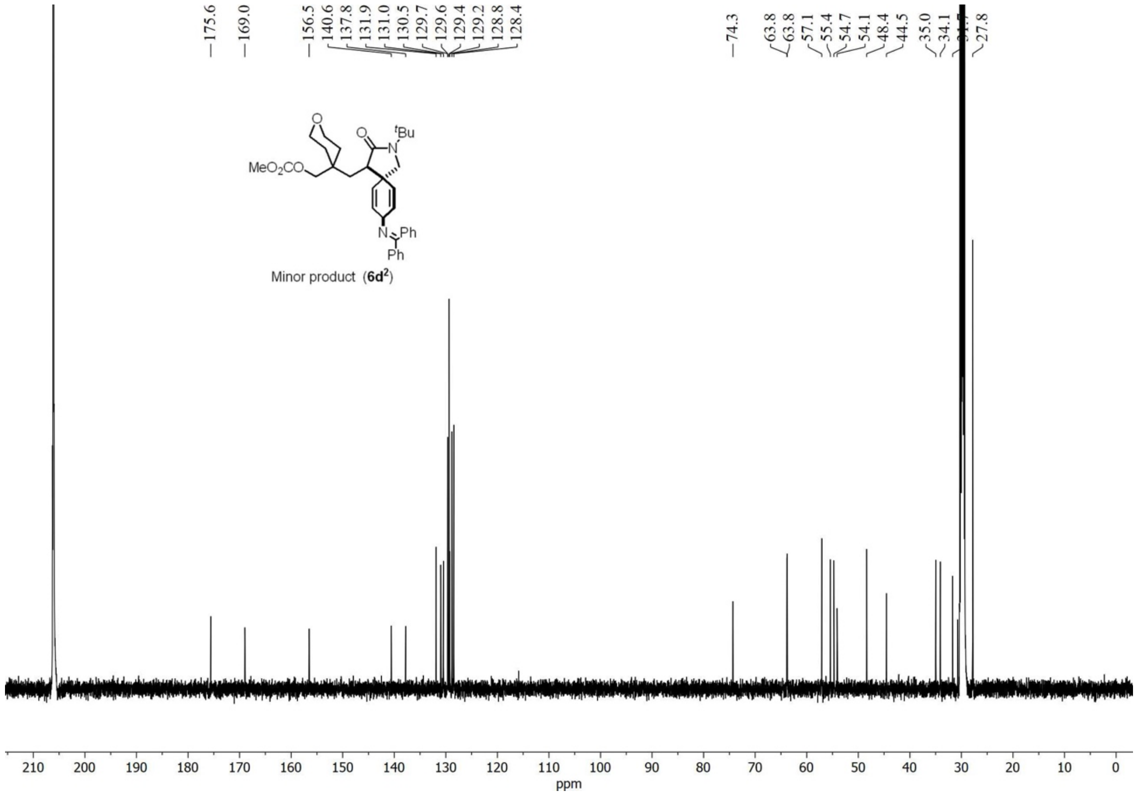

(1-((2-(*tert*-Butyl)-8-((diphenylmethylene)amino)-3-oxo-2-azaspiro[4.5]deca-6,9-dien-4-yl)methyl)cyclopentyl)methyl methyl carbonate (6e)

Major product

^{1}H NMR (500 MHz, Acetone-d_6)

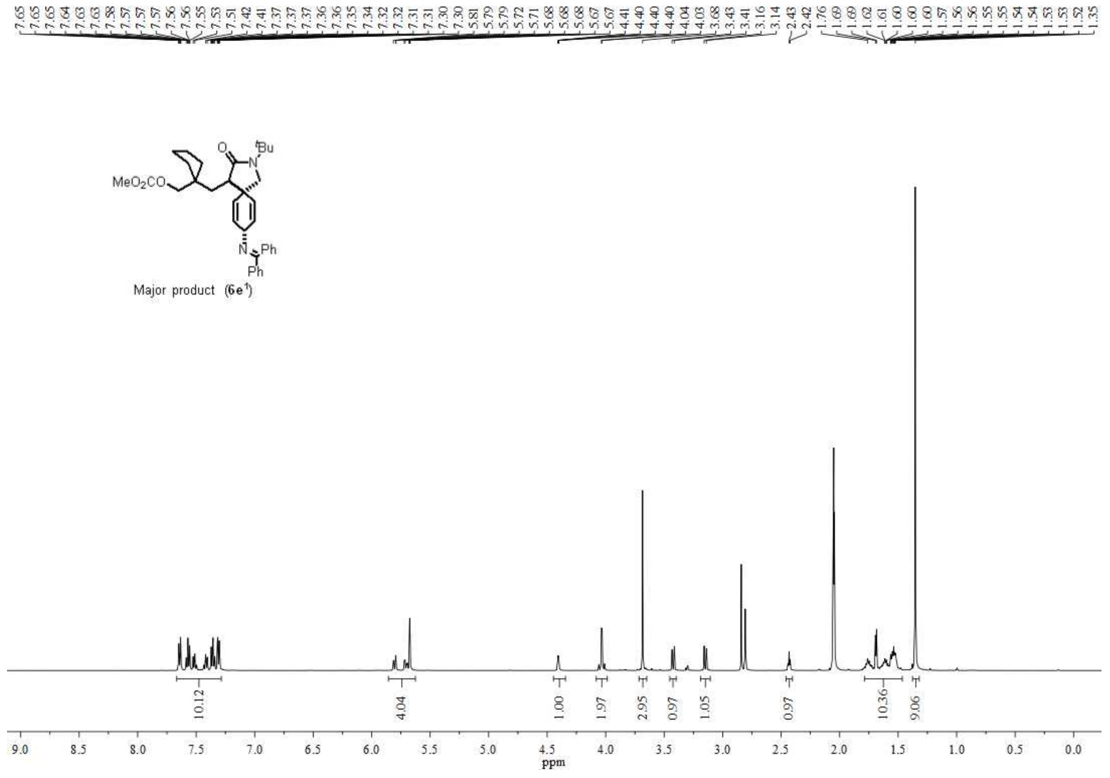

^{13}C NMR (126 MHz, Acetone-d_6)

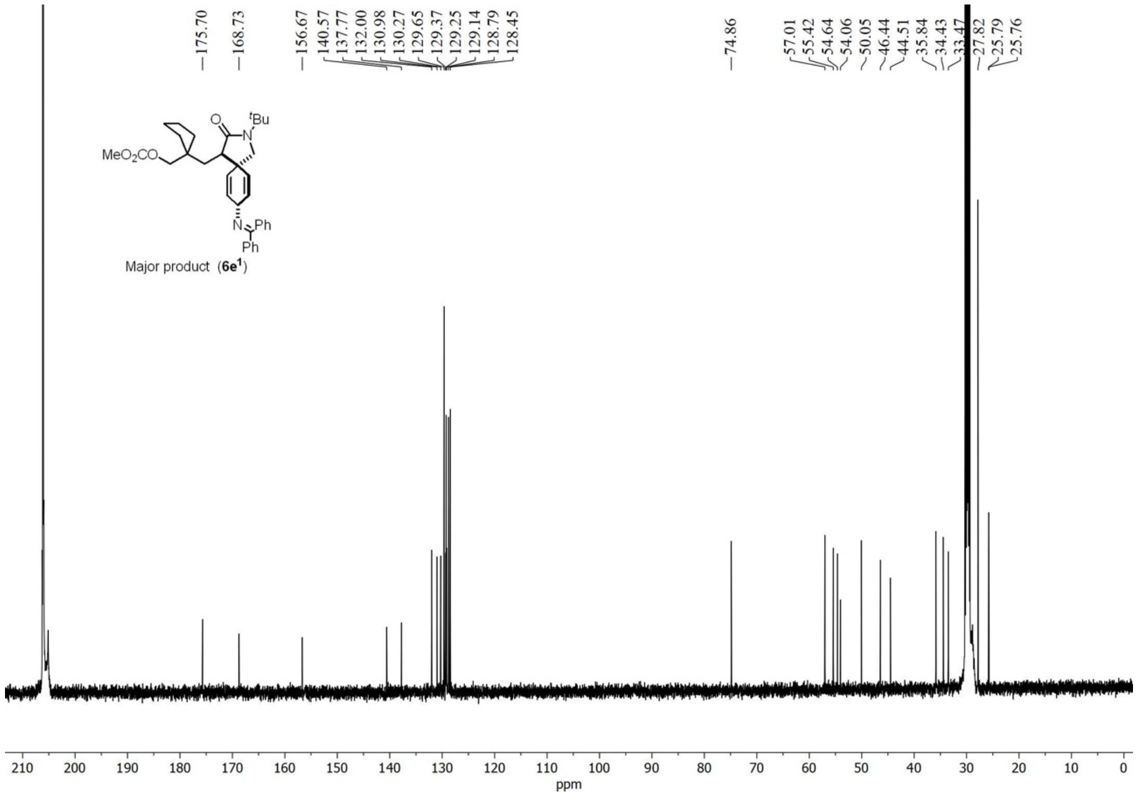

COSY

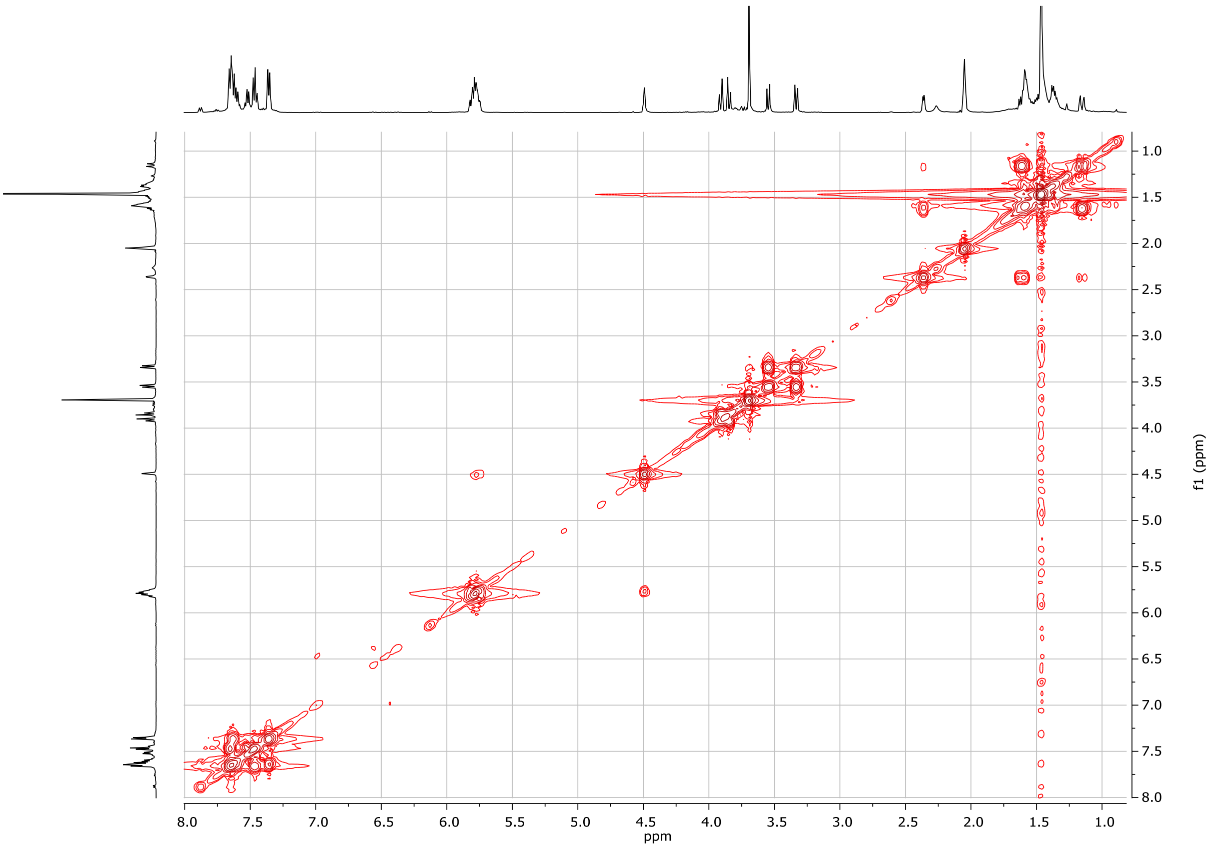

8.0
7.5
7.0
6.5
6.0
5.5
5.0
4.5
4.0
3.5
3.0
2.5
2.0
1.5
1.0
ppm
f1 (ppm)

HSQC

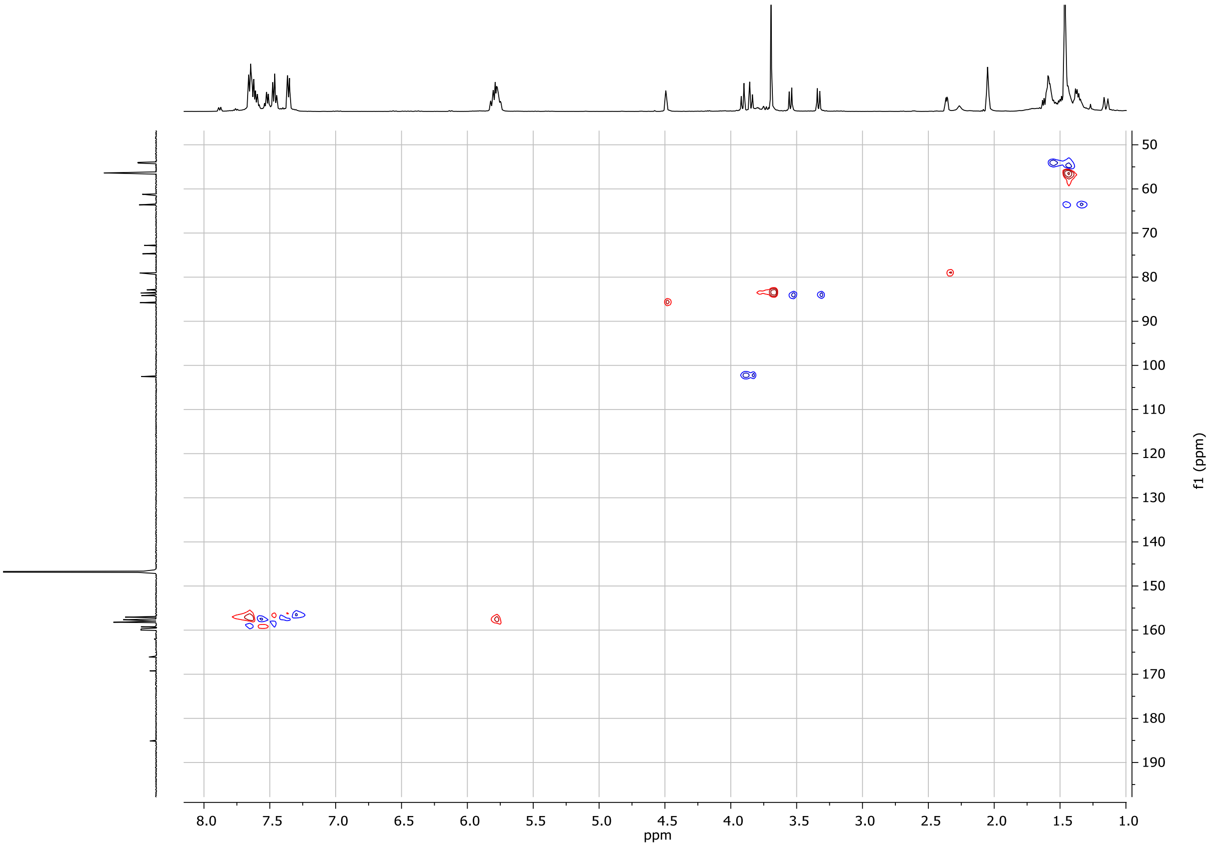

8.0
7.5
7.0
6.5
6.0
5.5
5.0
4.5
4.0
3.5
3.0
2.5
2.0
1.5
1.0
ppm
f1 (ppm)
50
60
70
80
90
100
110
120
130
140
150
160
170
180
190

HMBC

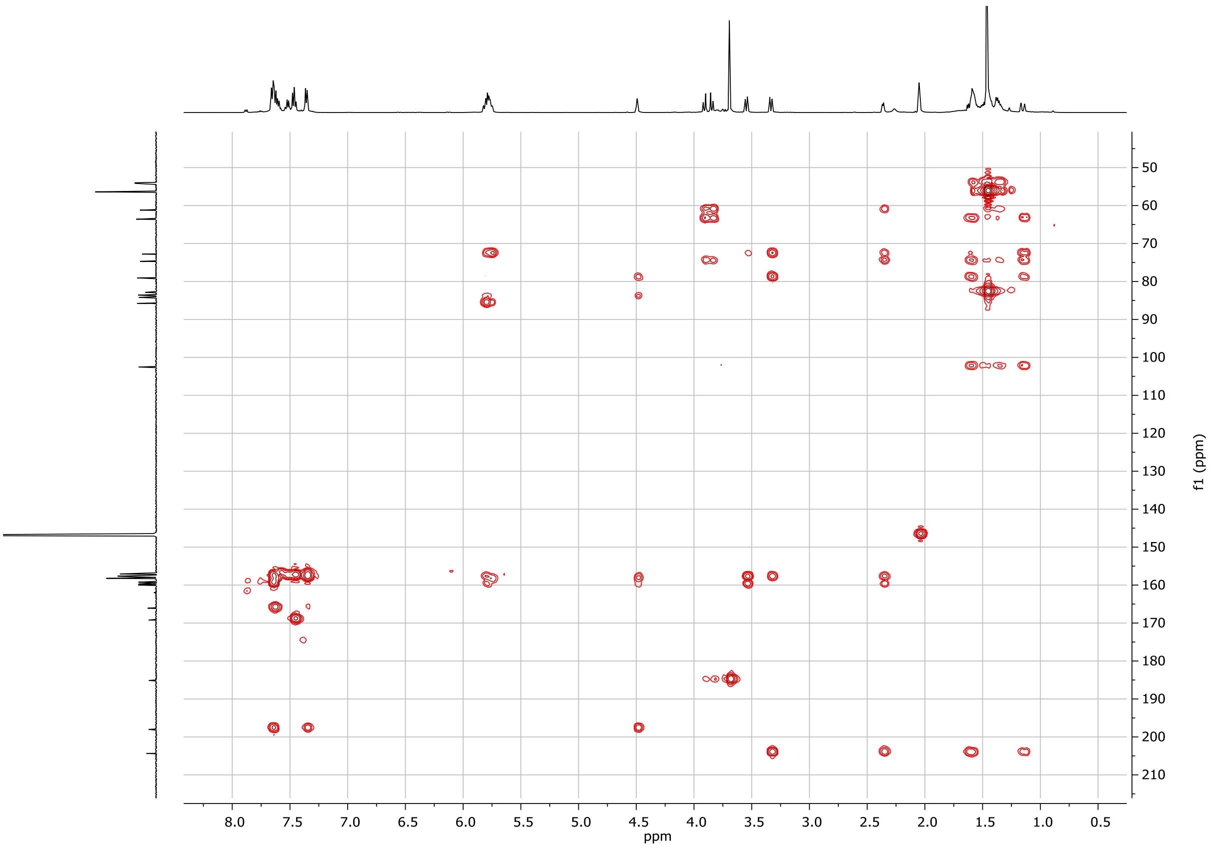

NOESY

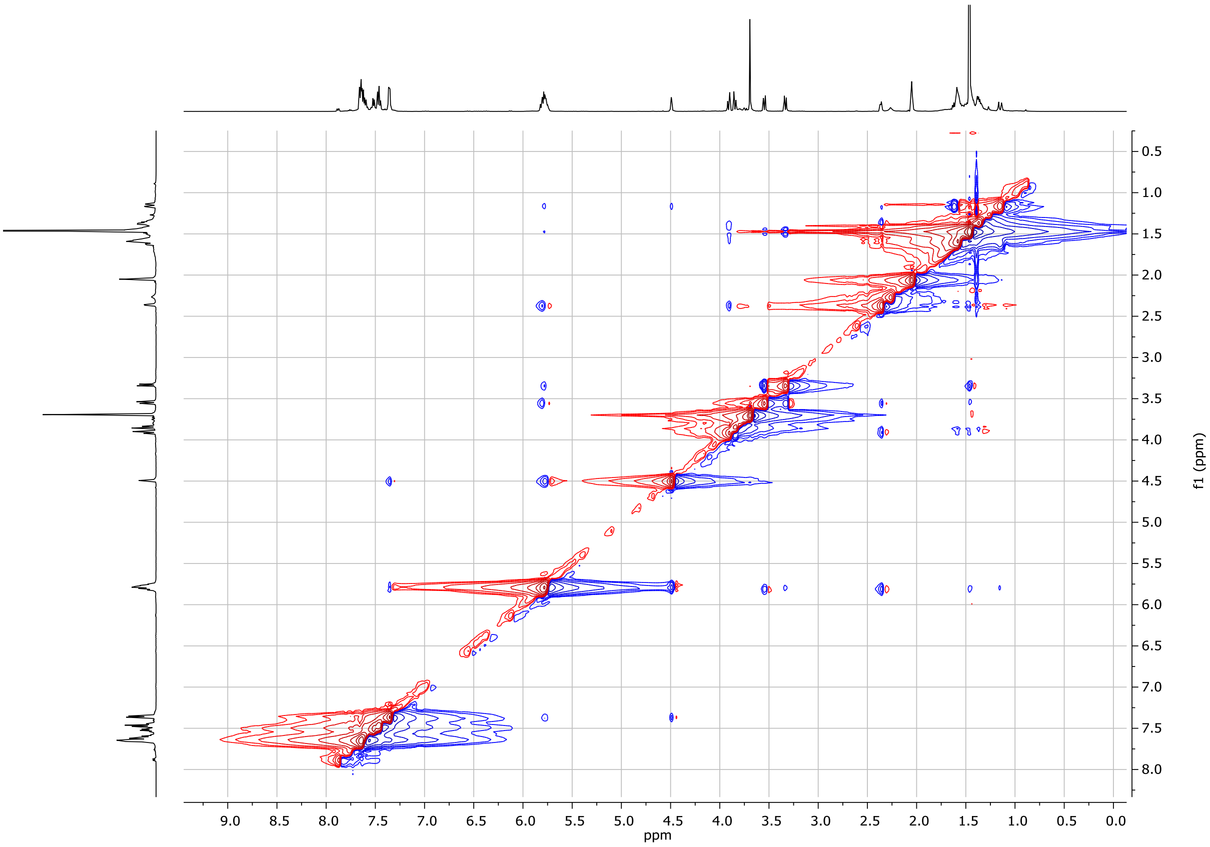

Minor product

^{1}H NMR (500 MHz, CD_3OD)

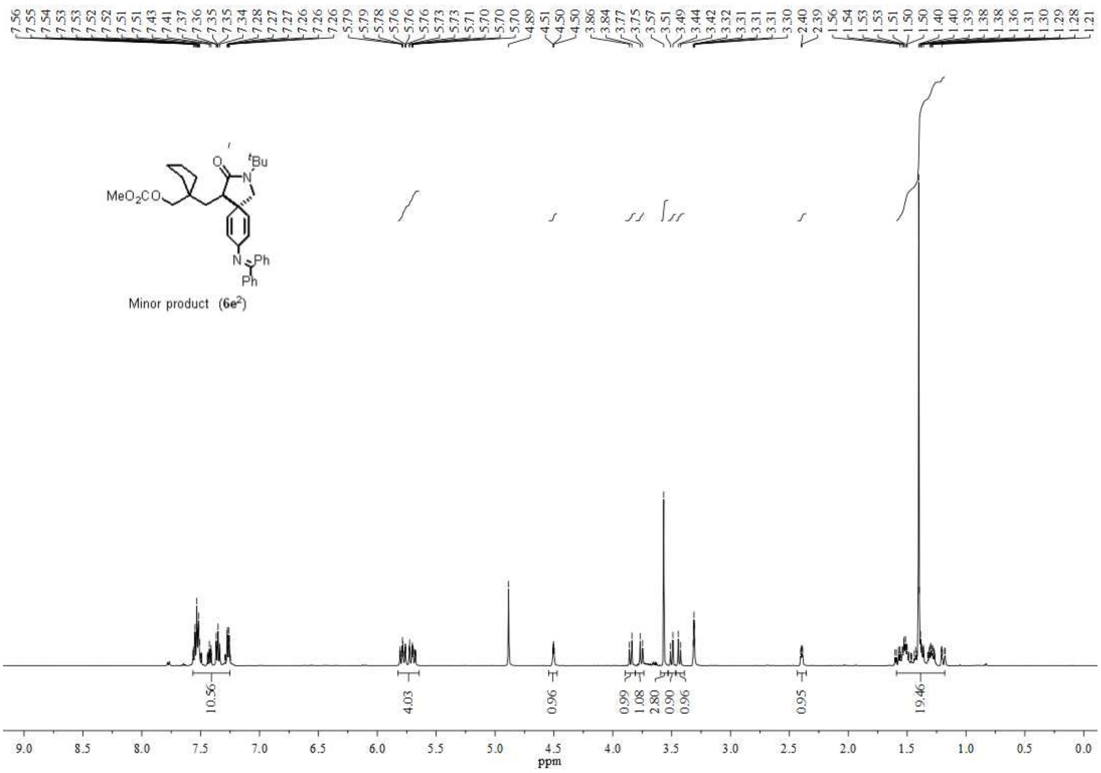

^{13}C NMR (126 MHz, CD_3OD)

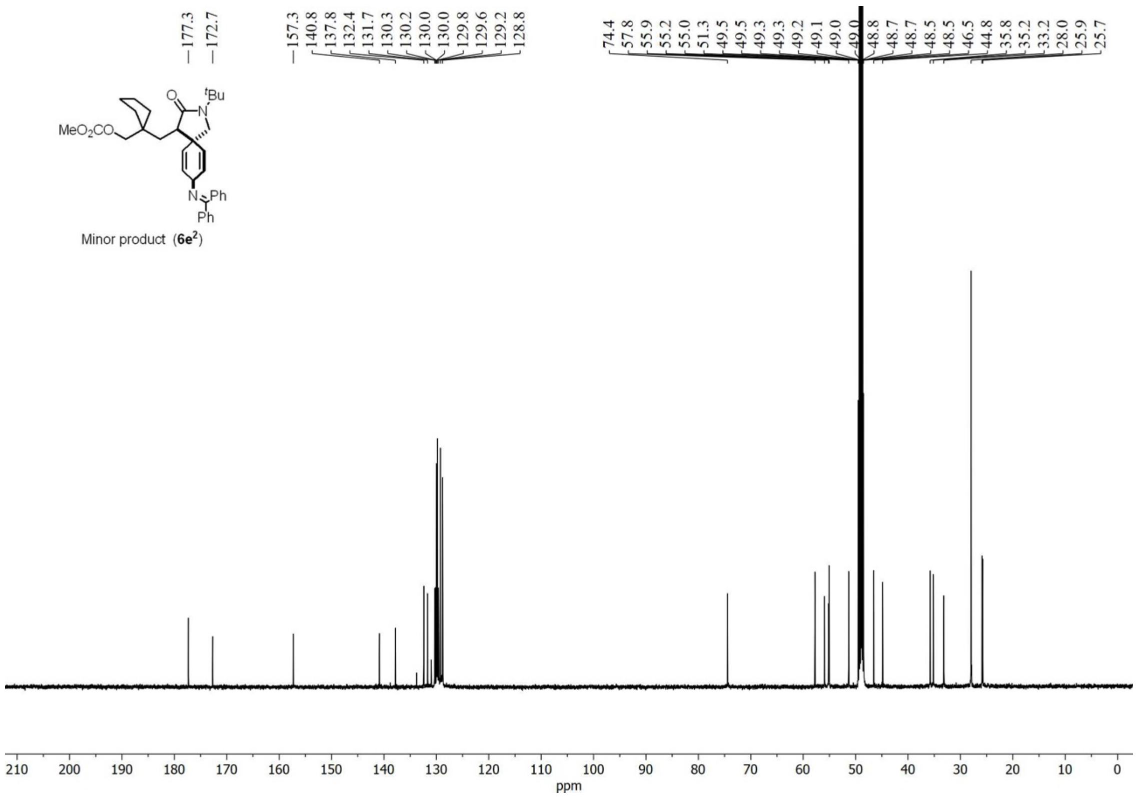

COSY

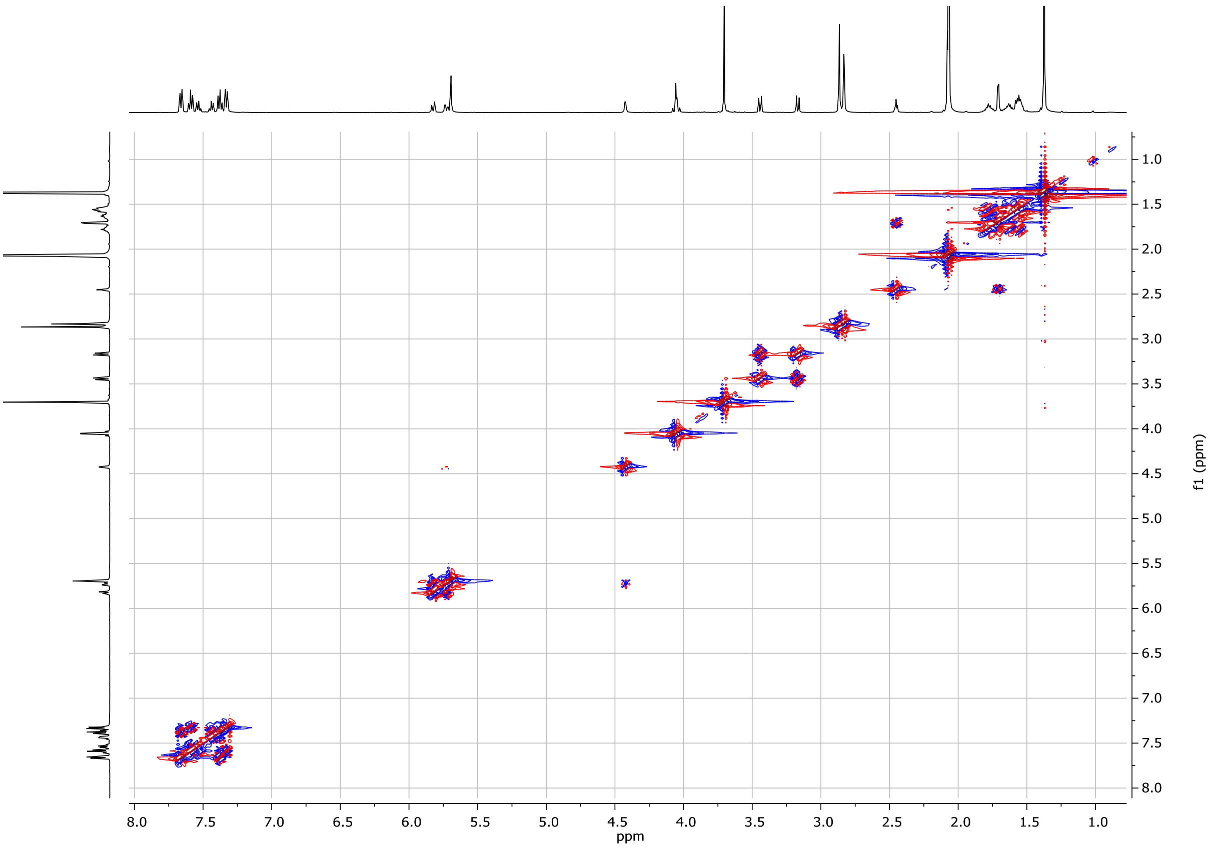

HSQC

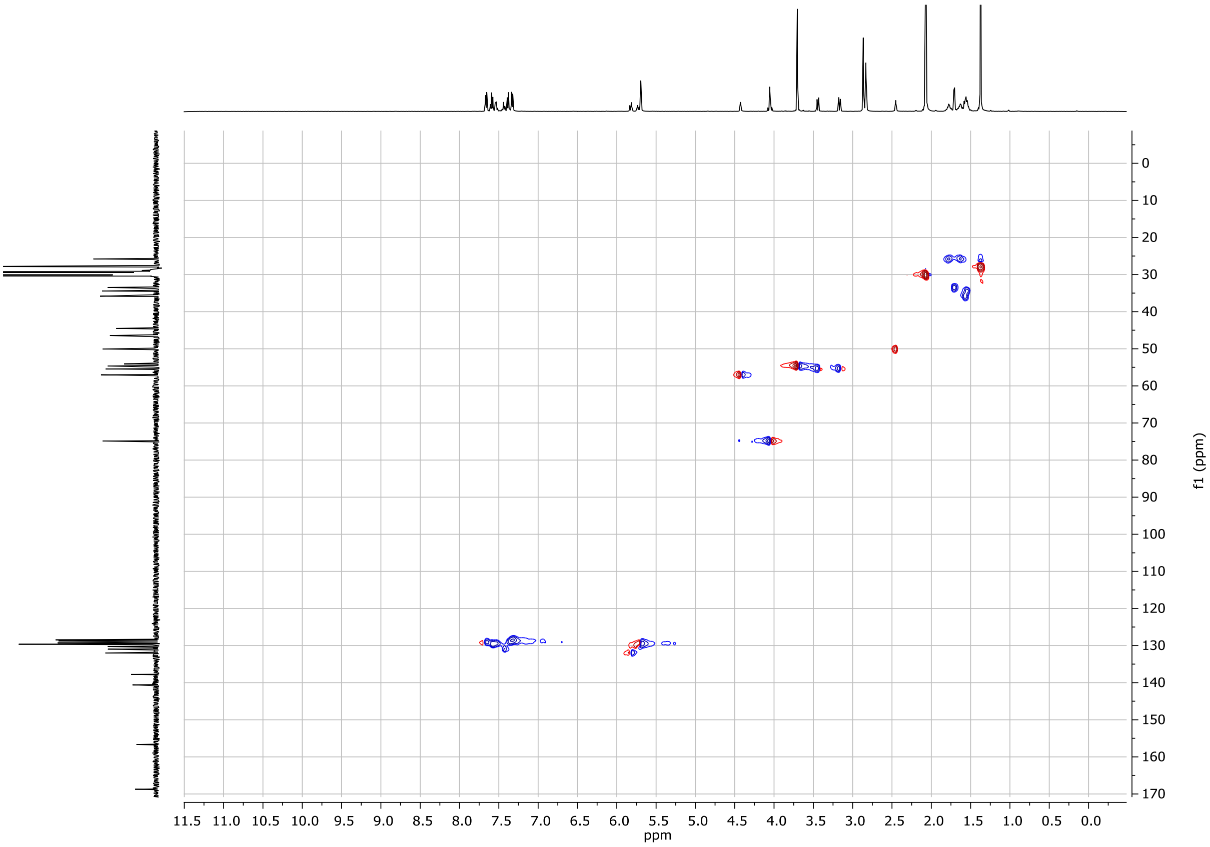

HMBC

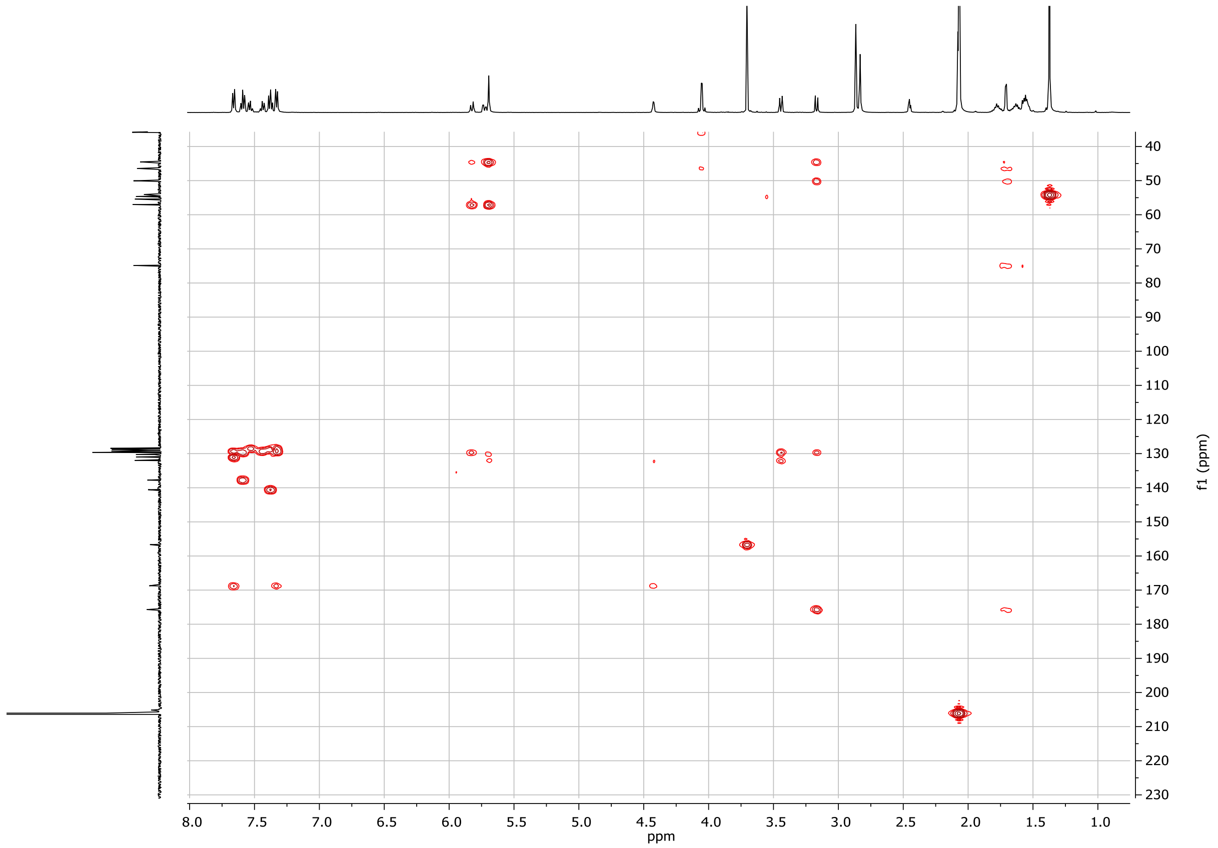
8.0
7.5
7.0
6.5
6.0
5.5
5.0
4.5
4.0
3.5
3.0
2.5
2.0
1.5
1.0
ppm
40
50
60
70
80
90
100
110
120
130
140
150
160
170
180
190
200
210
220
230
f1 (ppm)

NOESY

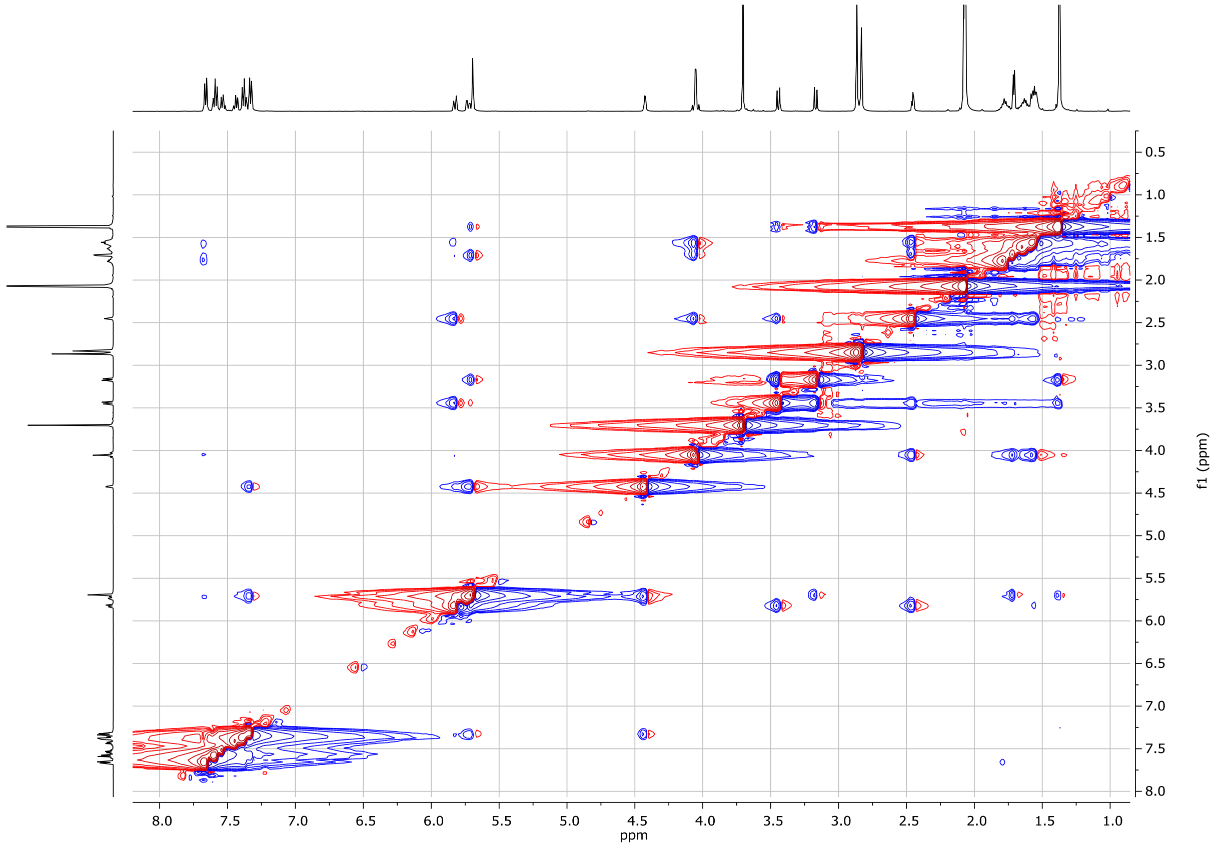
8.0
7.5
7.0
6.5
6.0
5.5
5.0
4.5
4.0
3.5
3.0
2.5
2.0
1.5
1.0
ppm
0.5
1.0
1.5
2.0
2.5
3.0
3.5
4.0
4.5
5.0
5.5
6.0
6.5
7.0
7.5
8.0
f1 (ppm)

(1-((2-(*tert*-Butyl)-8-((diphenylmethylene)amino)-3-oxo-2-azaspiro[4.5]deca-6,9-dien-4-yl)methyl)cyclobutyl)methyl methyl carbonate (6f)

^{1}H NMR (400 MHz, CD_3OD)

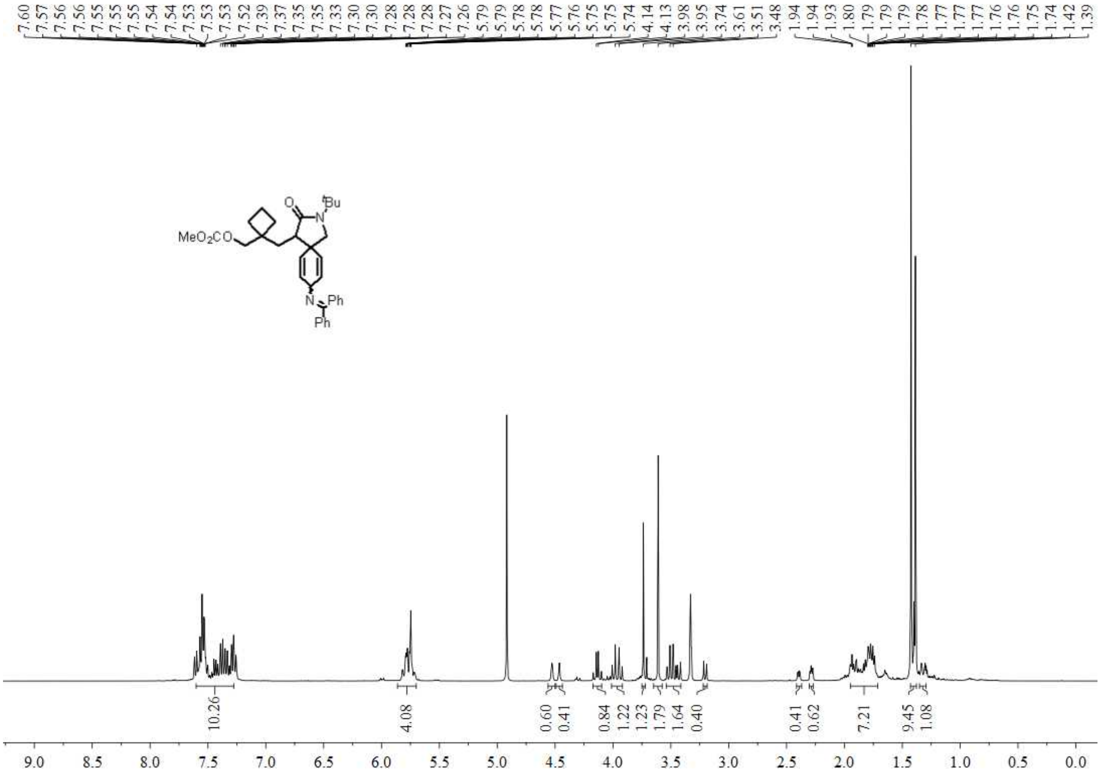

^{13}C NMR (101 MHz, CD_3OD)

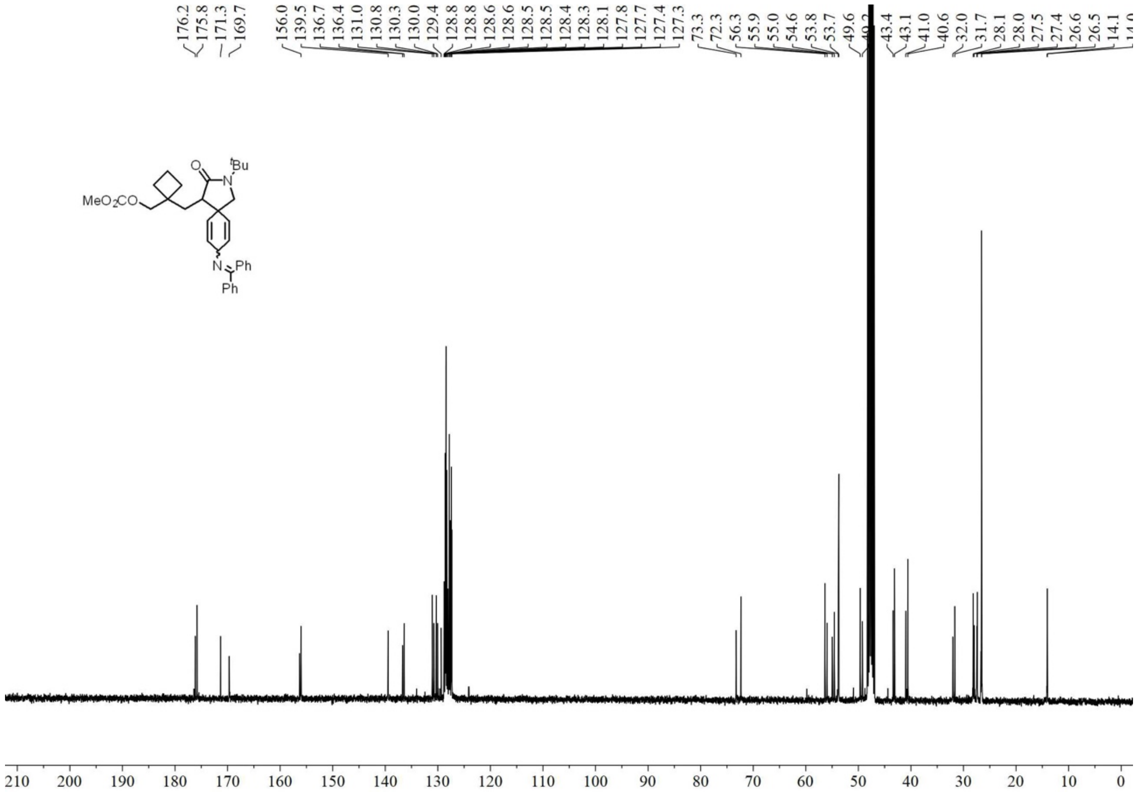

2-((2-(*tert*-Butyl)-8-((diphenylmethylene)amino)-3-oxo-2-azaspiro[4.5]deca-6,9-dien-4-yl)methyl)-2-ethylbutyl methyl carbonate (6g)

^{1}H NMR (400 MHz, CD_3OD)

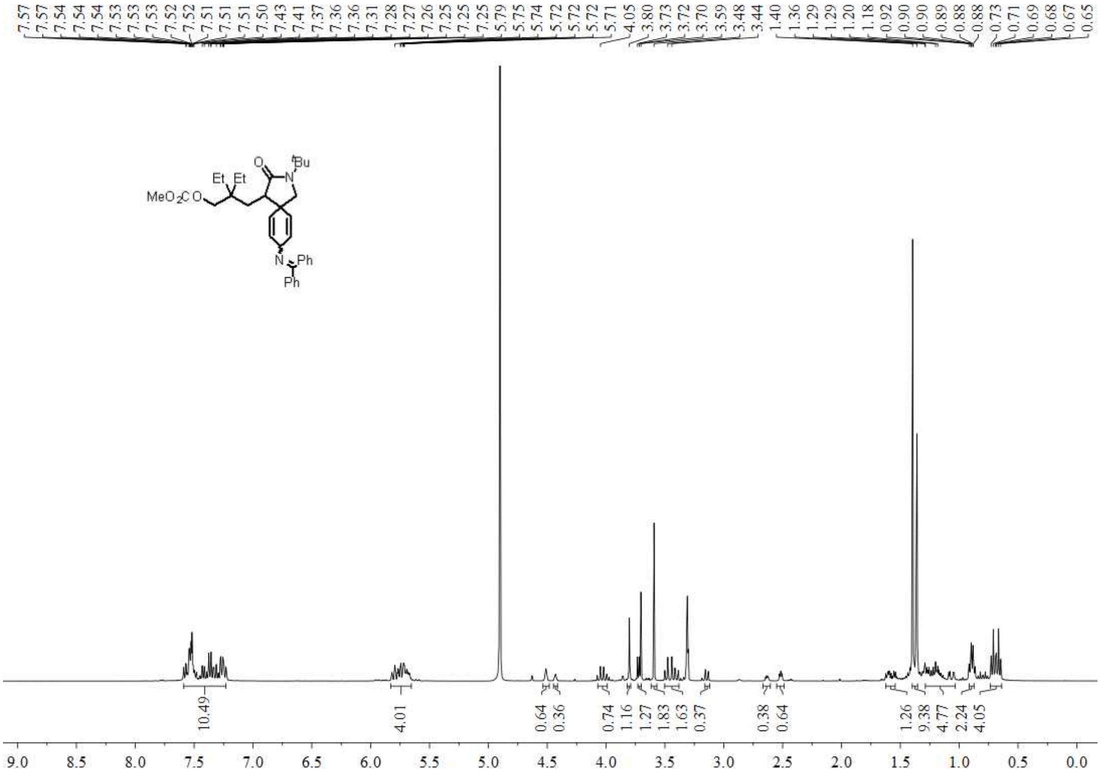

^{13}C NMR (101 MHz, CD_3OD)

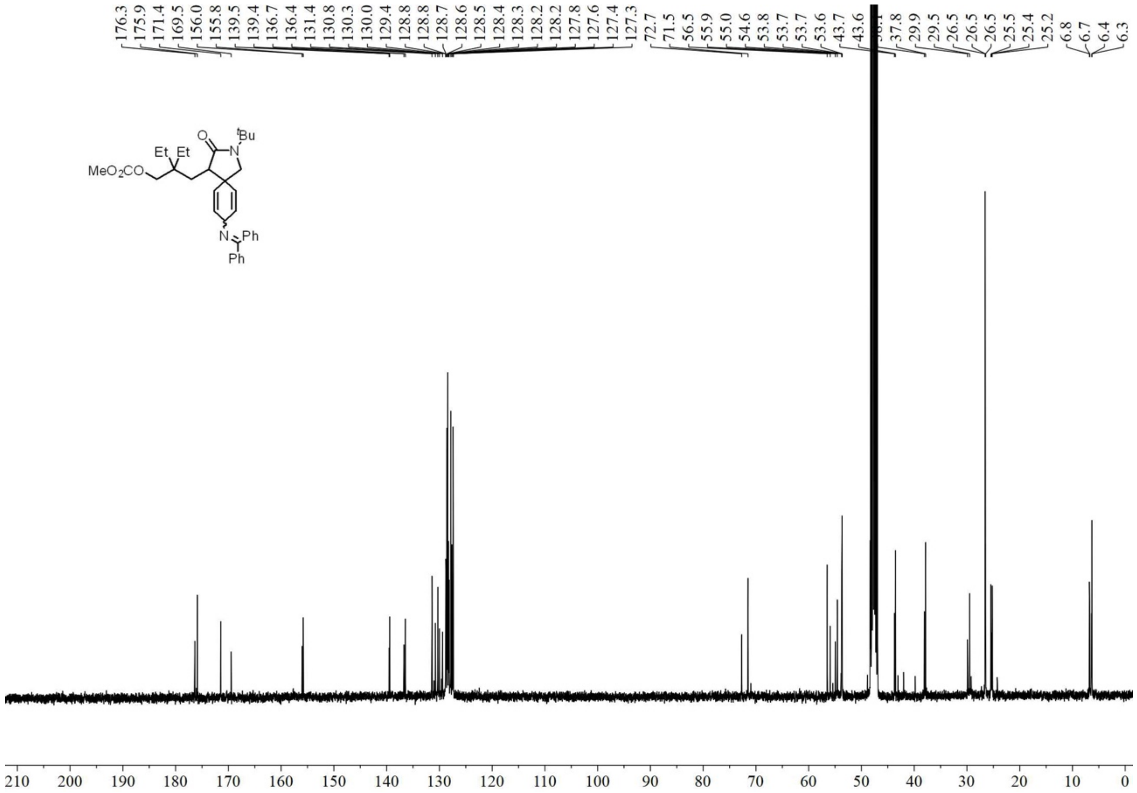

(1-((2-(*tert*-Butyl)-8-((diphenylmethylene)amino)-7,9-dimethyl-3-oxo-2-azaspiro[4.5]deca-6,9-dien-4-yl)methyl)cyclohexyl)methyl methyl carbonate (6h)

^{1}H NMR (400 MHz, CD_3OD)

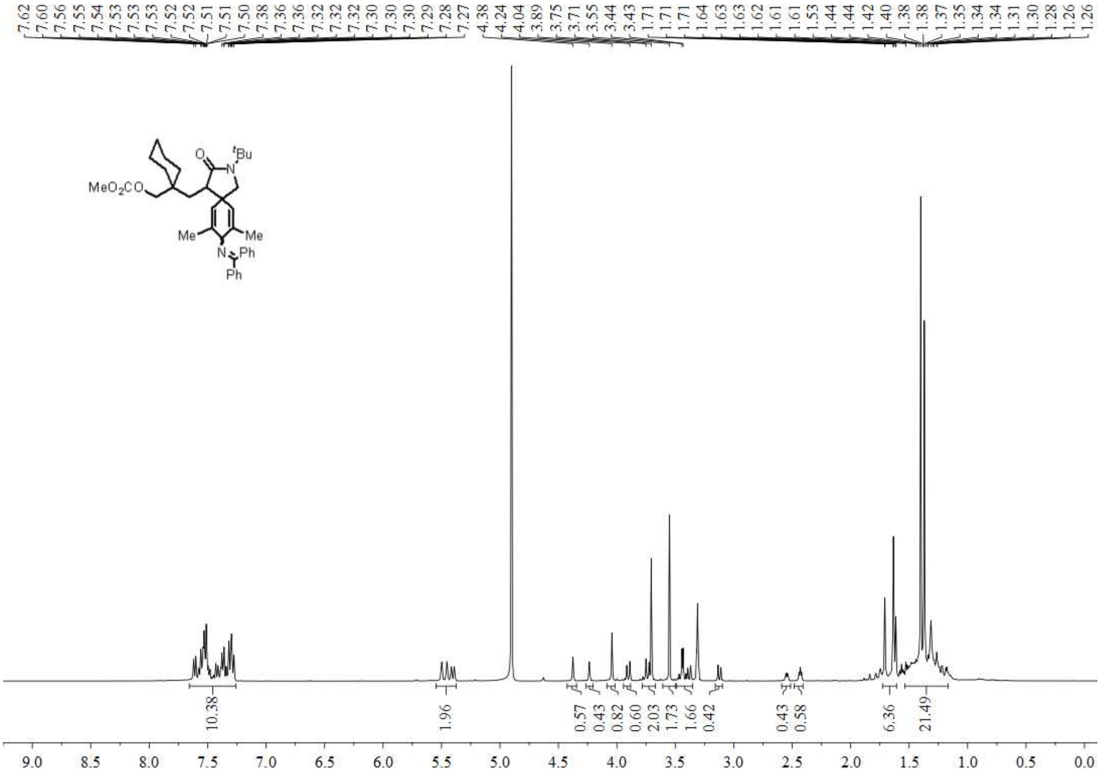

^{13}C NMR (101 MHz, CD_3OD)

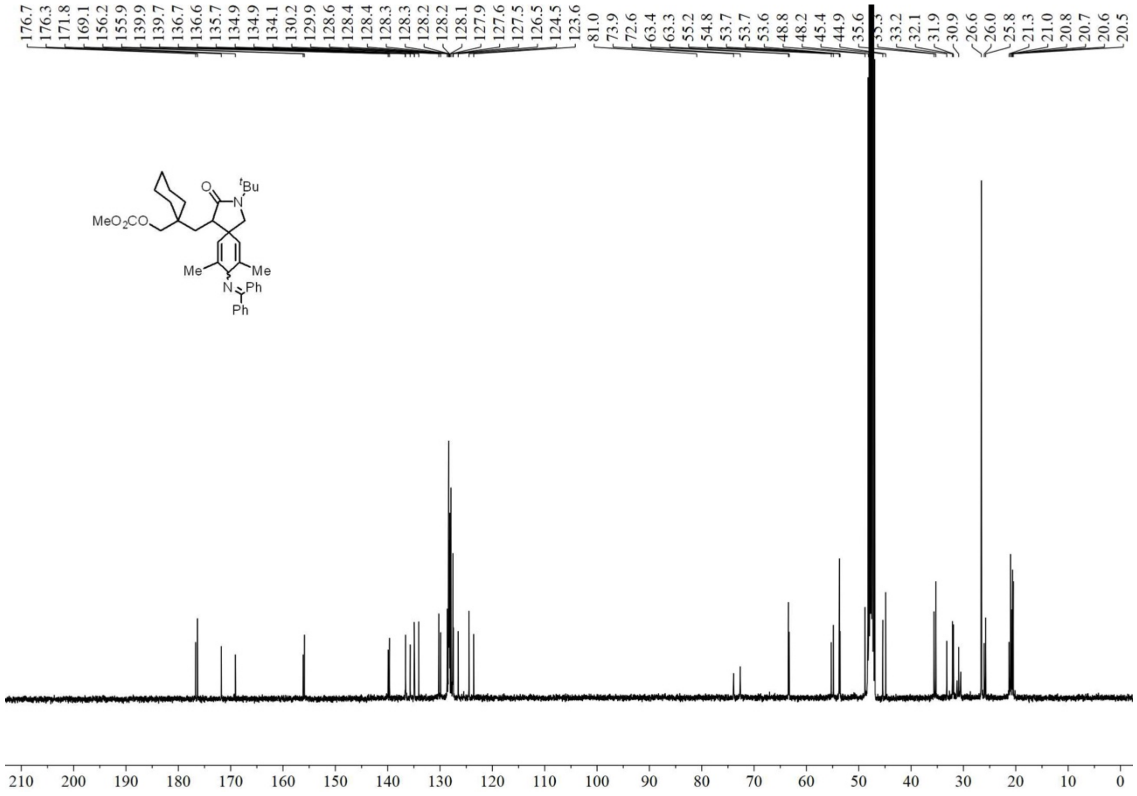

(1-((2-(*tert*-Butyl)-7,9-dichloro-8-((diphenylmethylene)amino)-3-oxo-2-azaspiro[4.5]deca-6,9-dien-4-yl)methyl)cyclohexyl)methyl methyl carbonate (6i)

^{1}H NMR (500 MHz, CD_3OD)

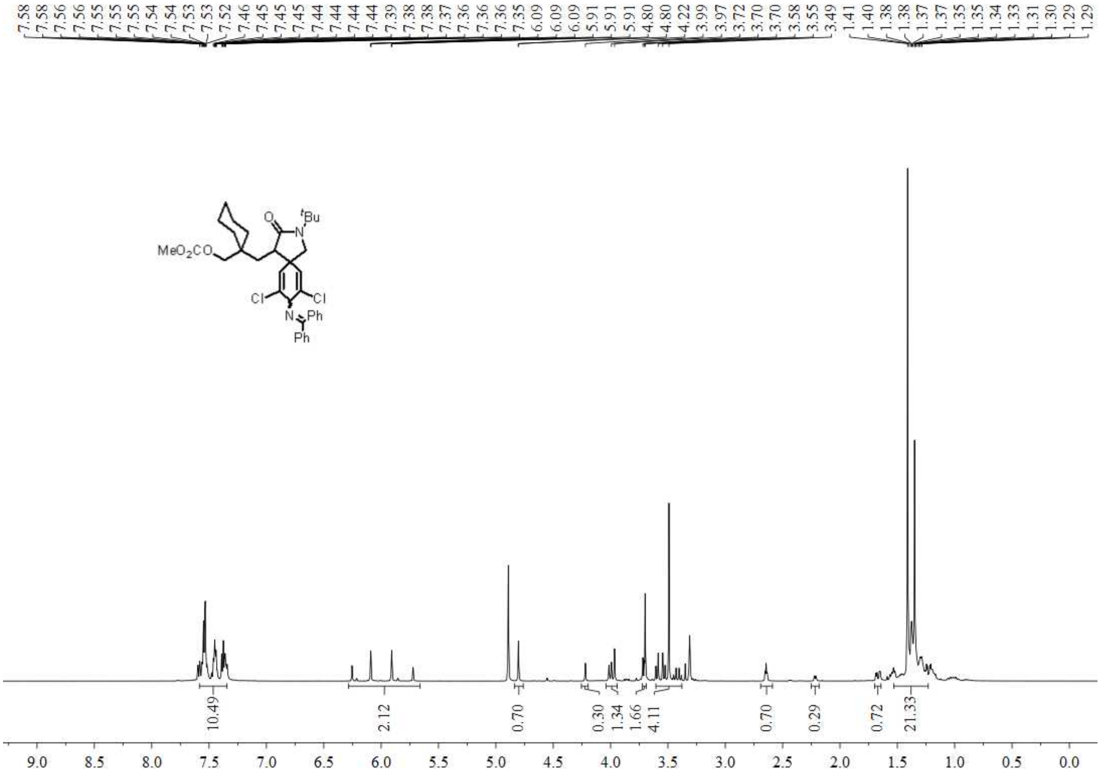

^{13}C NMR (126 MHz, CD_3OD)

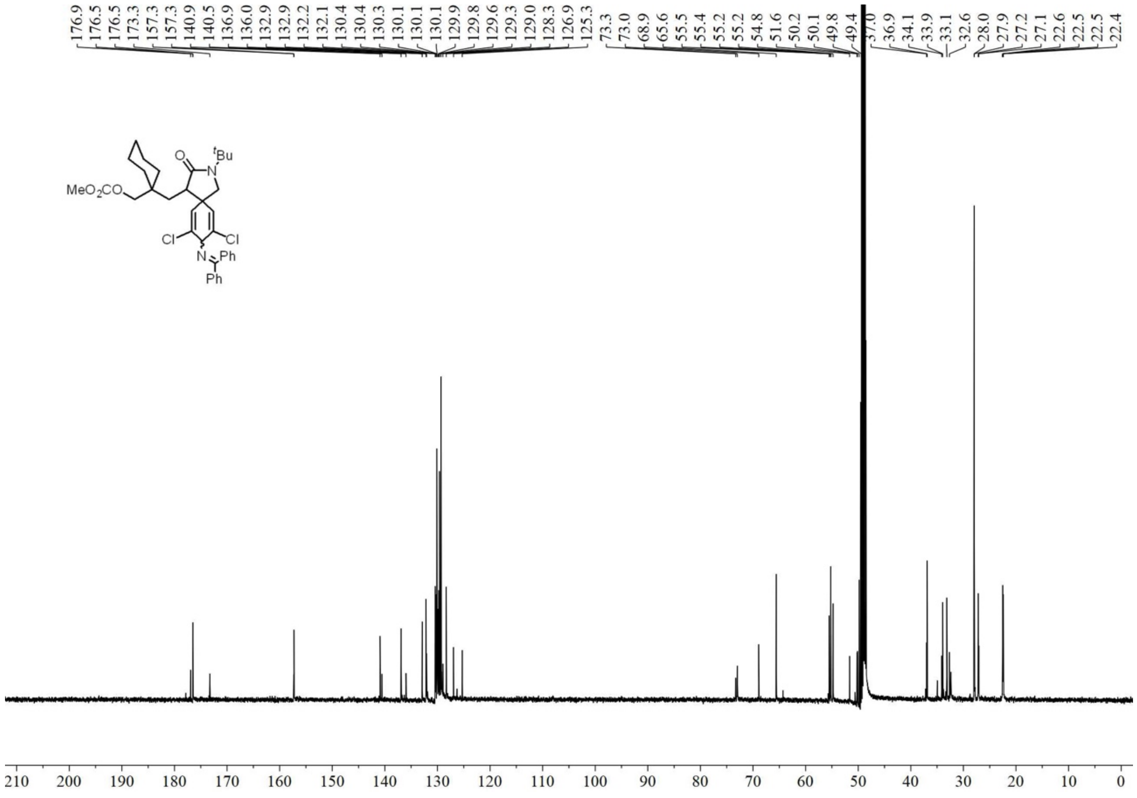

(1-((2-(*tert*-Butyl)-8-((diphenylmethylene)amino)-6,10-dimethyl-3-oxo-2-azaspiro[4.5]deca-6,9-dien-4-yl)methyl)cyclohexyl)methyl methyl carbonate (6j)

^{1}H NMR (400 MHz, CD_3OD)

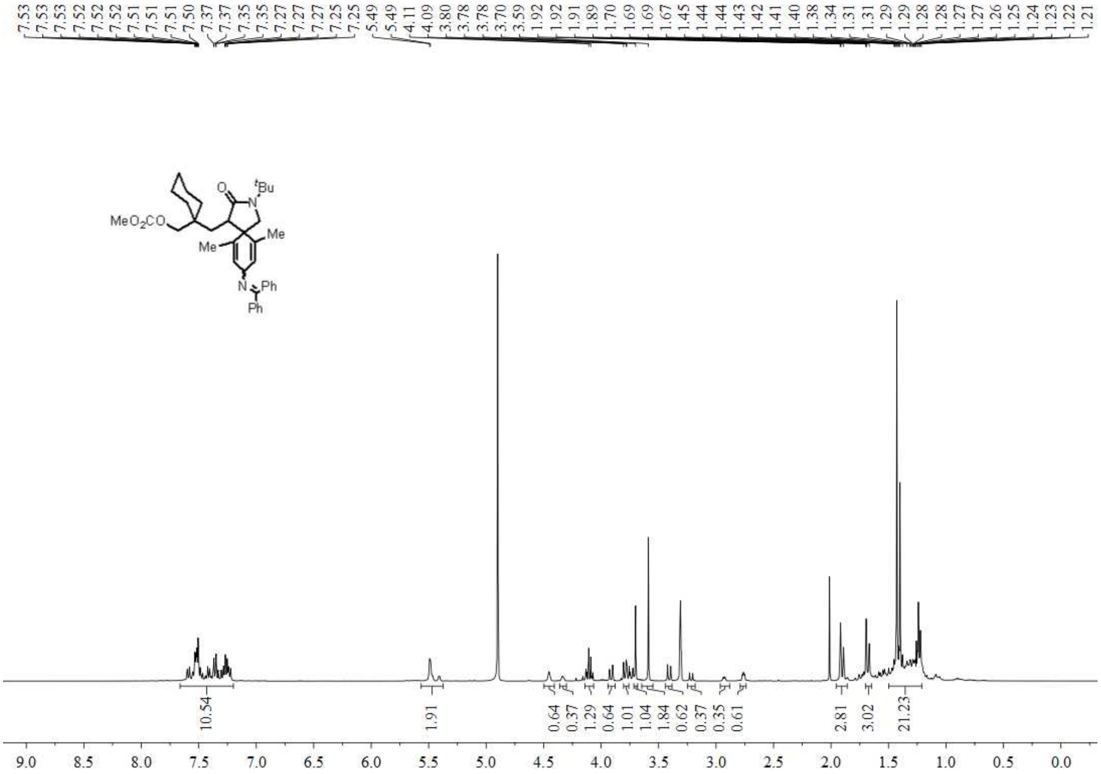

^{13}C NMR (101 MHz, CD_3OD)

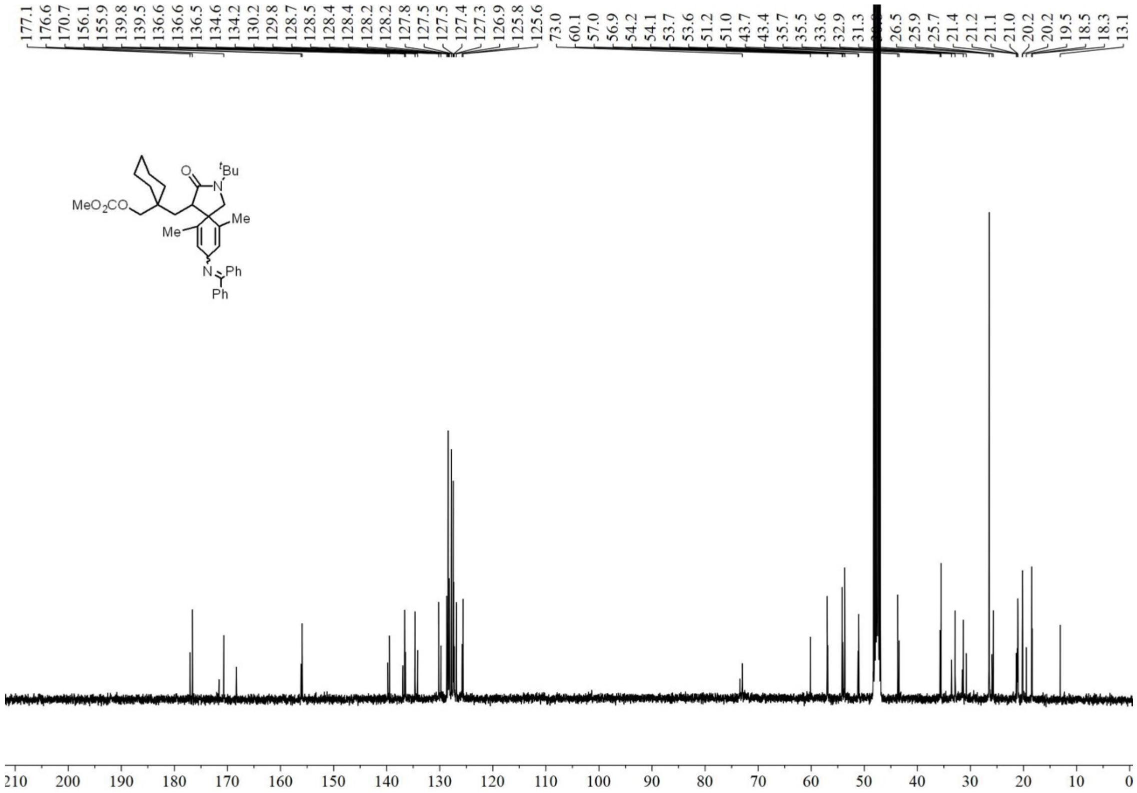

2-(*tert*-Butyl)-4-(cyclopentylmethyl)-8-((diphenylmethylene)amino)-2-azaspiro[4.5]deca-6,9-dien-3-one (8b)

^{1}H NMR (400 MHz, CD_3OD)

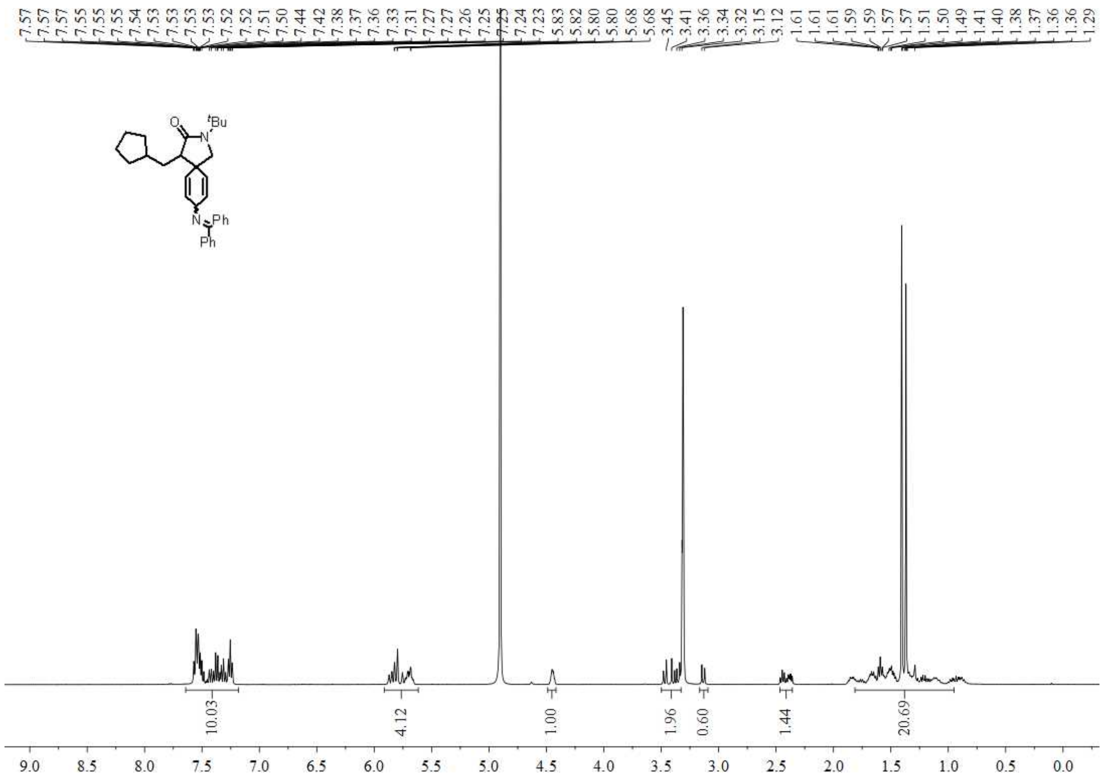

^{13}C NMR (101 MHz, CD_3OD)

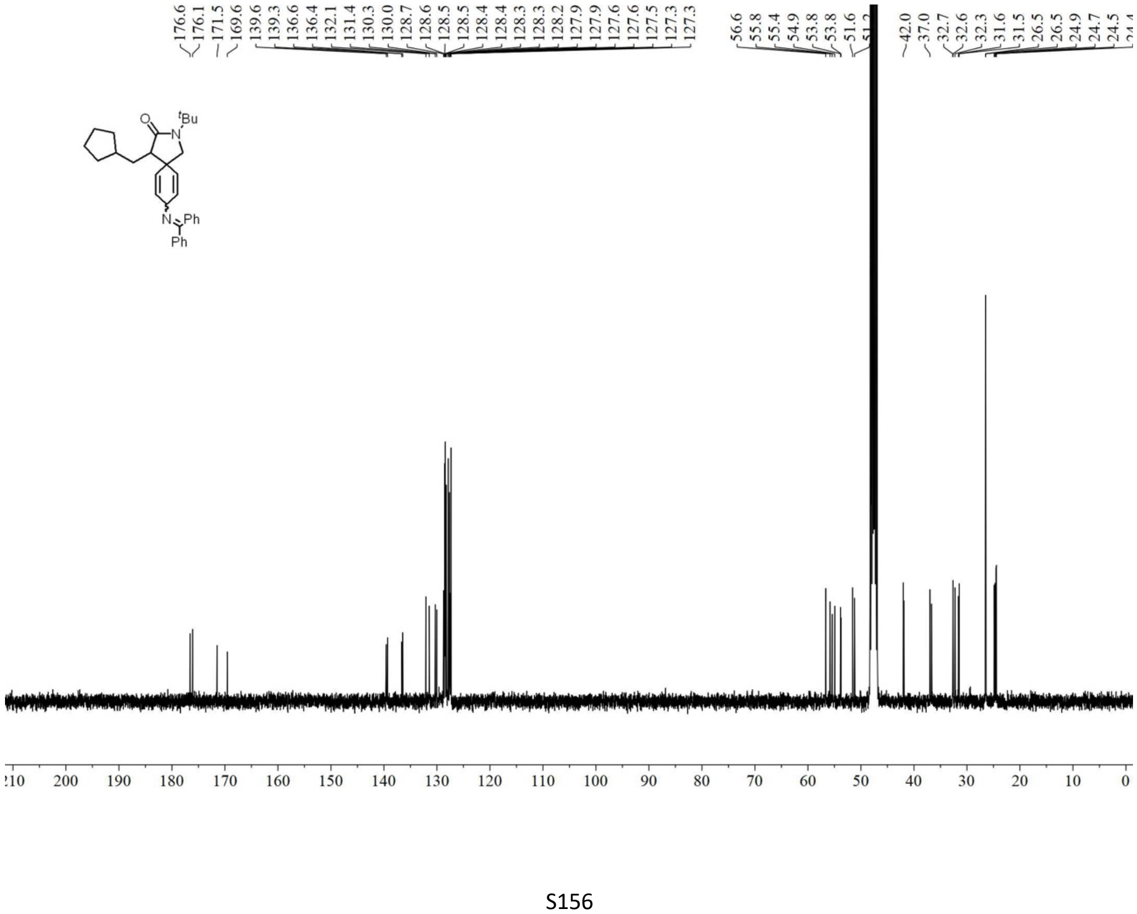

2-(*tert*-Butyl)-4-(cycloheptylmethyl)-8-((diphenylmethylene)amino)-2-azaspiro[4.5]deca-6,9-dien-3-one (8c)

Major product

^{1}H NMR (500 MHz, CD_3OD)

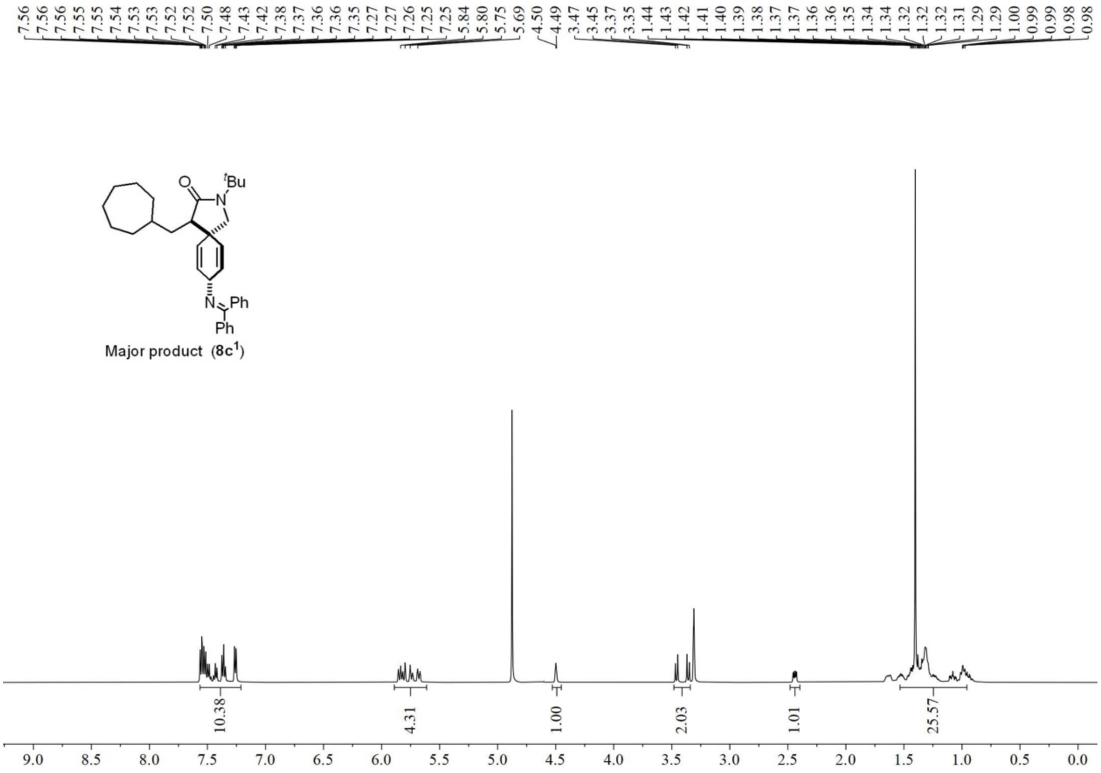

^{13}C NMR (126 MHz, CD_3OD)

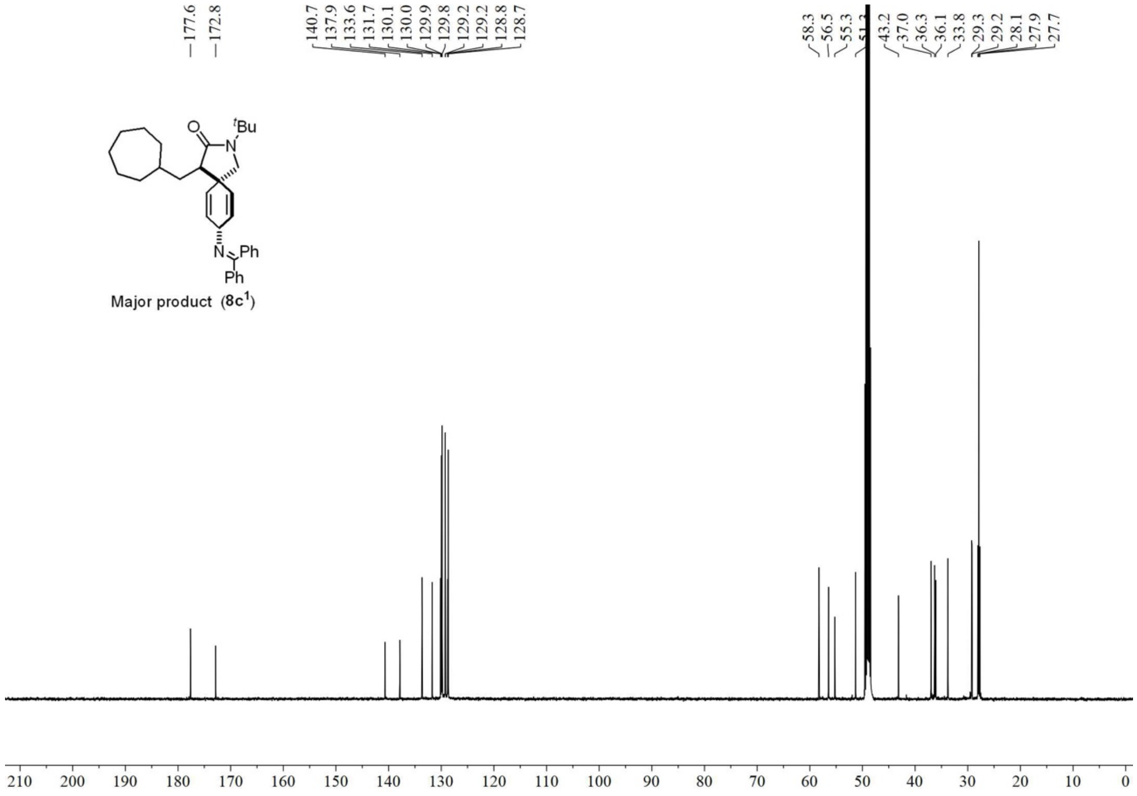

Minor product

^{1}H NMR (400 MHz, CD_3OD)

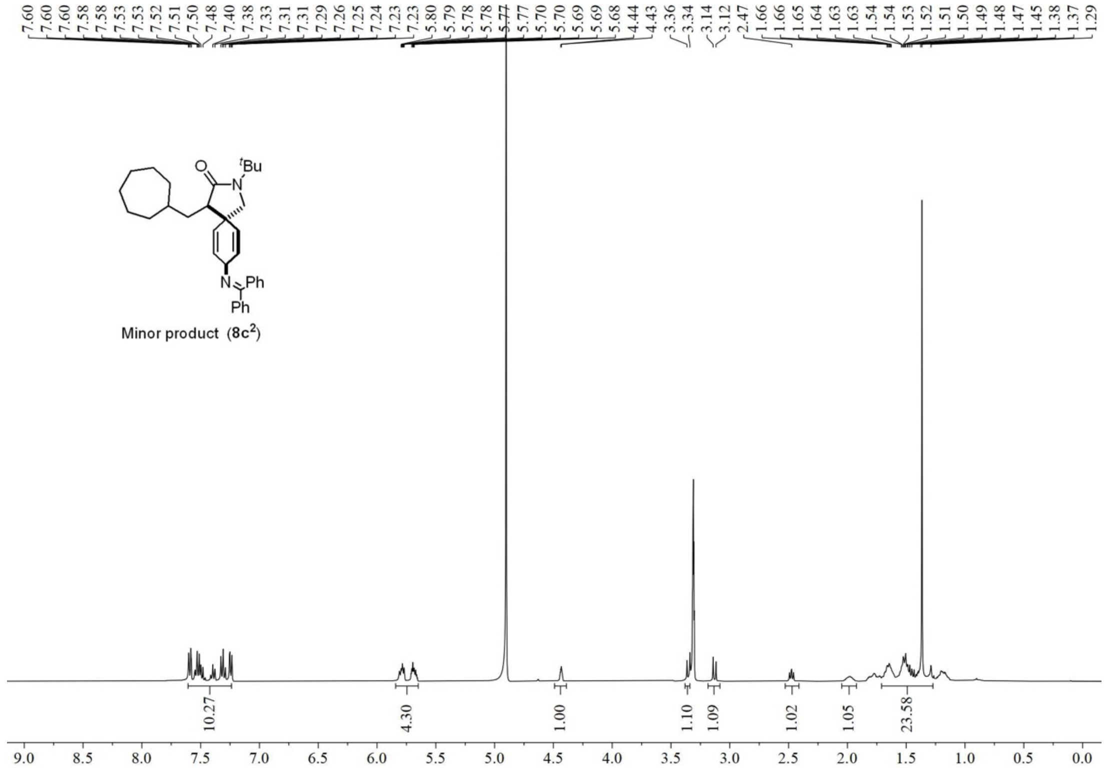

^{13}C NMR (101 MHz, CD_3OD)

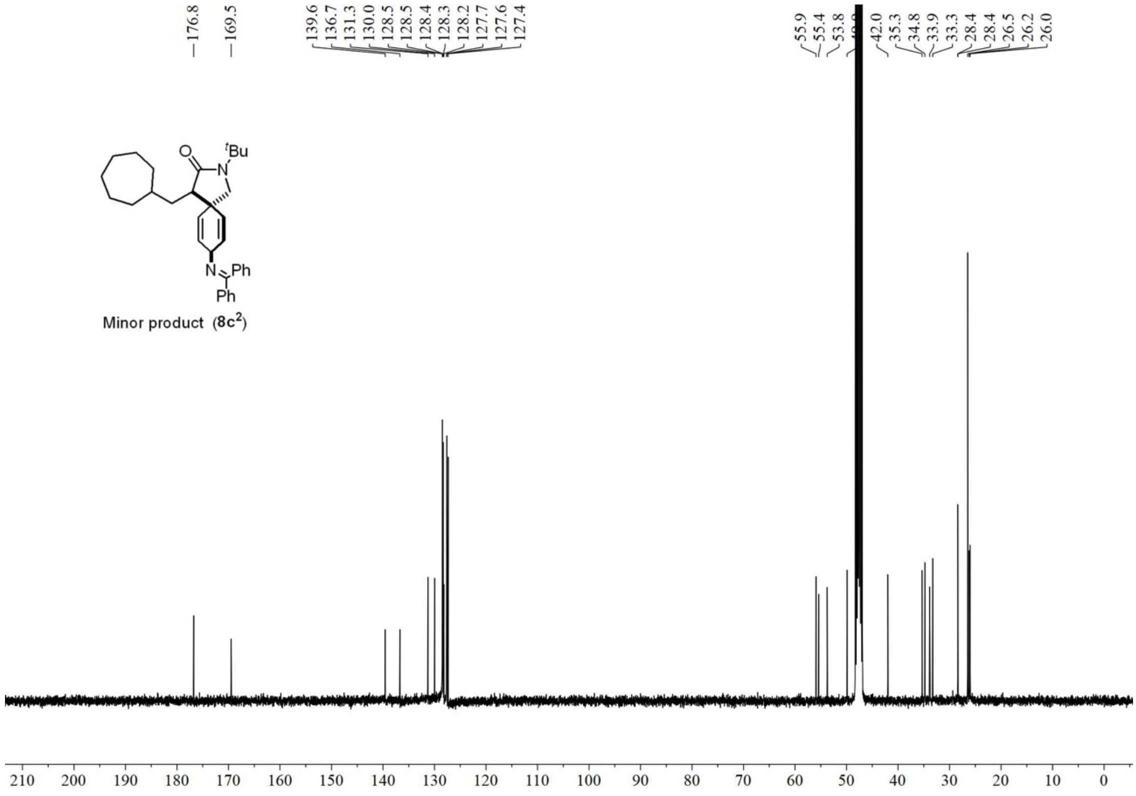

4-(2-(tert-Butoxy)ethyl)-2-(tert-butyl)-8-((diphenylmethylene)amino)-2-azaspiro[4.5]deca-6,9-dien-3-one (8d)

^{1}H NMR (400 MHz, CD_3OD)

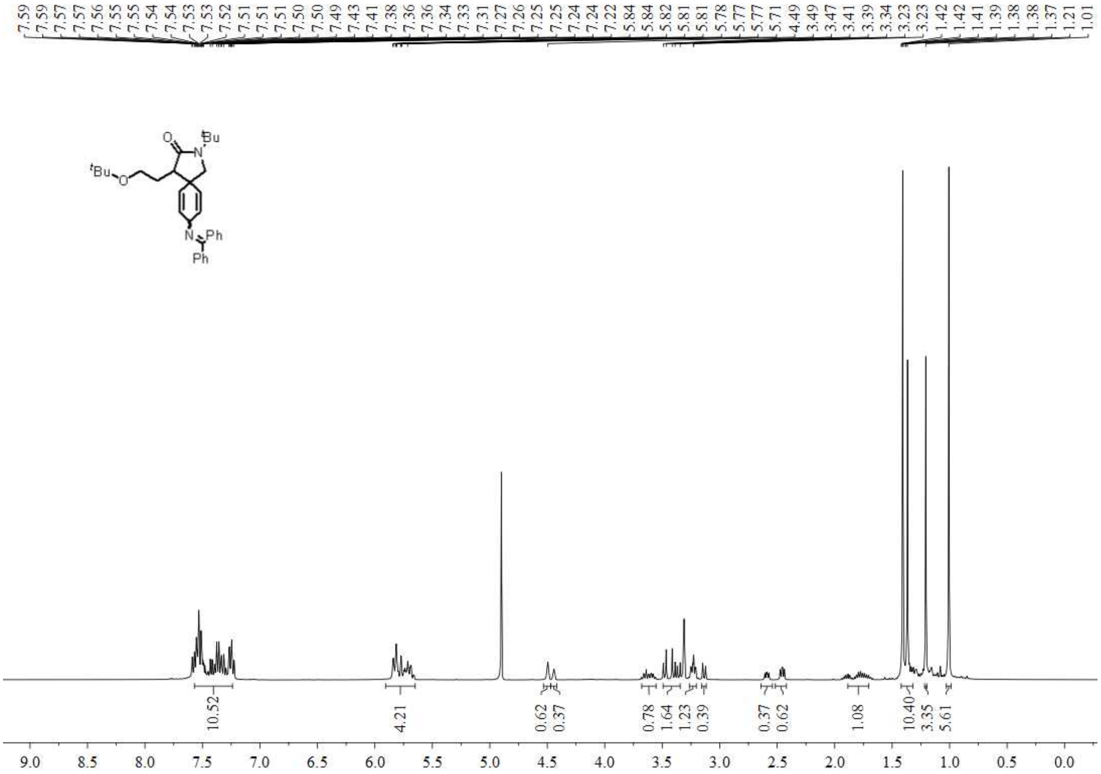

^{13}C NMR (101 MHz, CD_3OD)

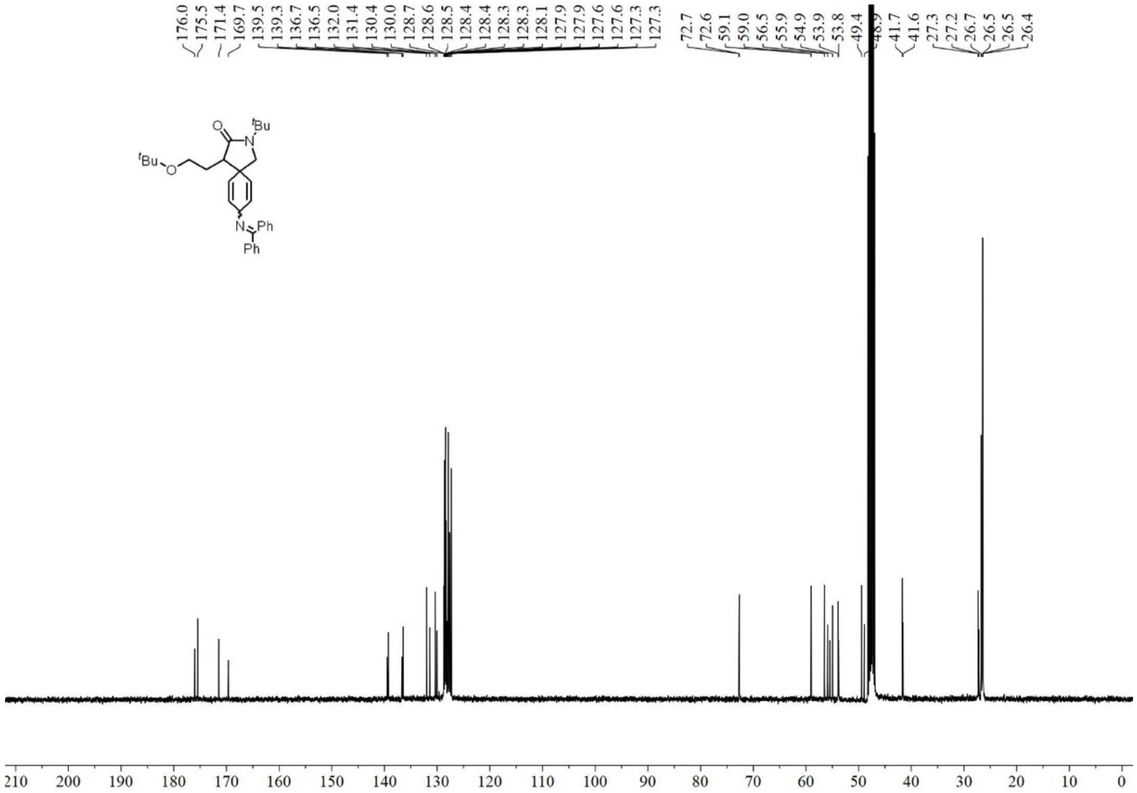

8-Amino-2-(*tert*-butyl)-4-(3-phenylpropyl)-2-azaspiro[4.5]deca-6,9-dien-3-one (9)

^1H NMR (400 MHz, CD_3OD)

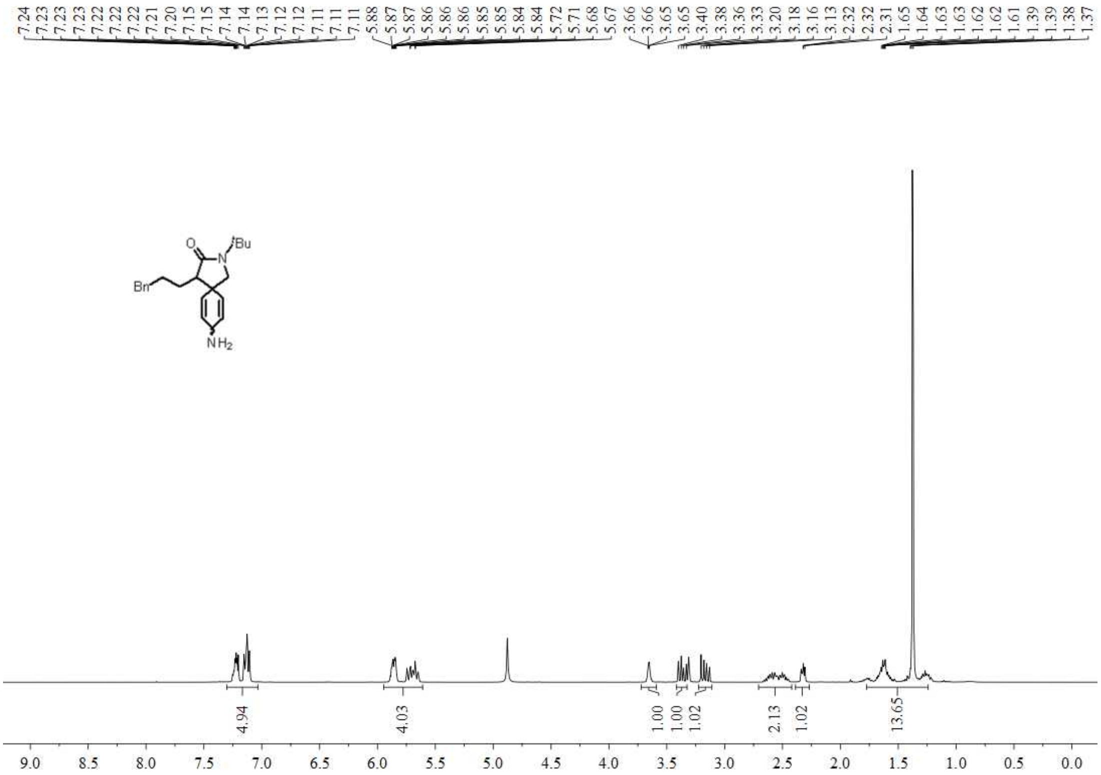

^{13}C NMR (101 MHz, CD_3OD)

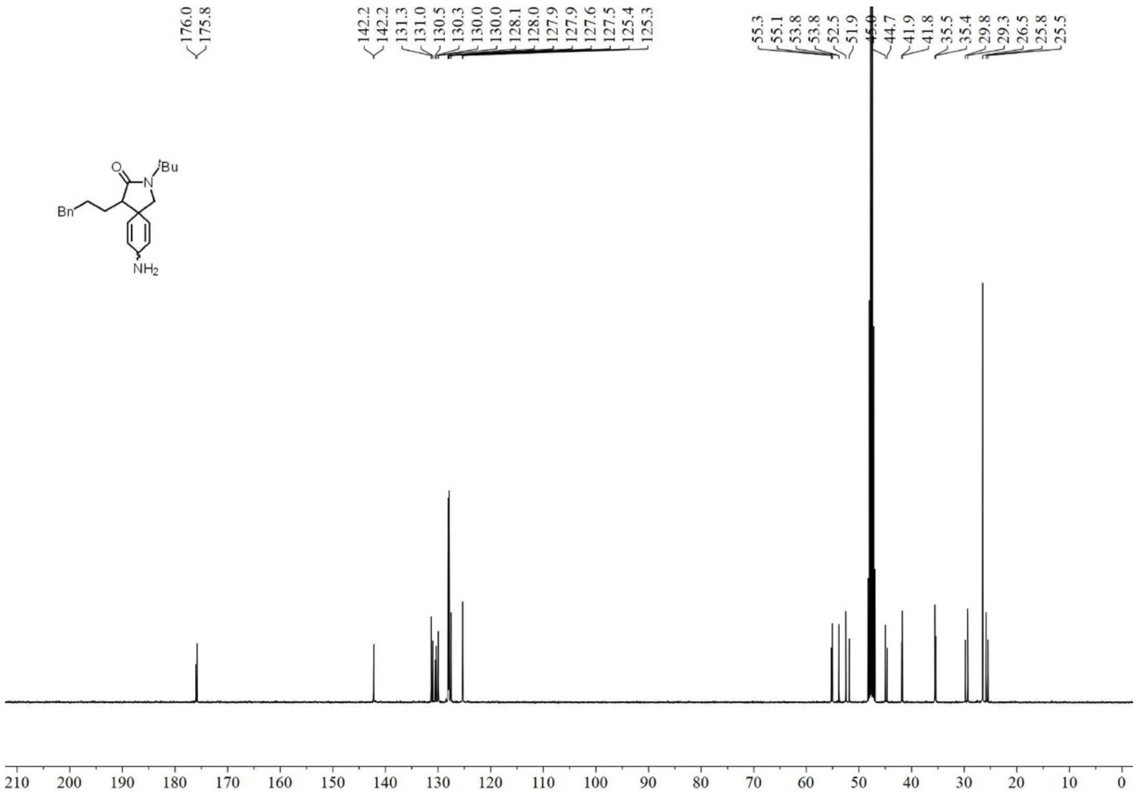

Ethyl 2-(8-amino-2-(tert-butyl)-3-oxo-2-azaspiro[4.5]deca-6,9-dien-4-yl)acetate (10).

^{1}H NMR (400 MHz, CD_3OD)

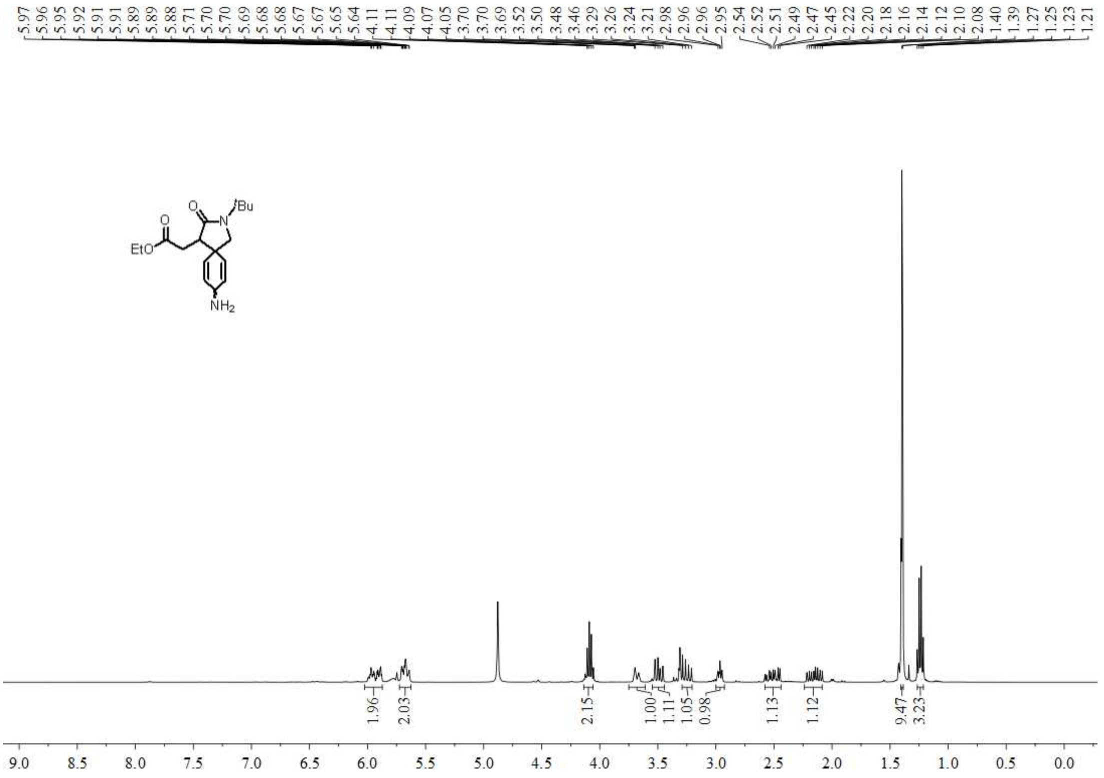

^{13}C NMR (101 MHz, CD_3OD)

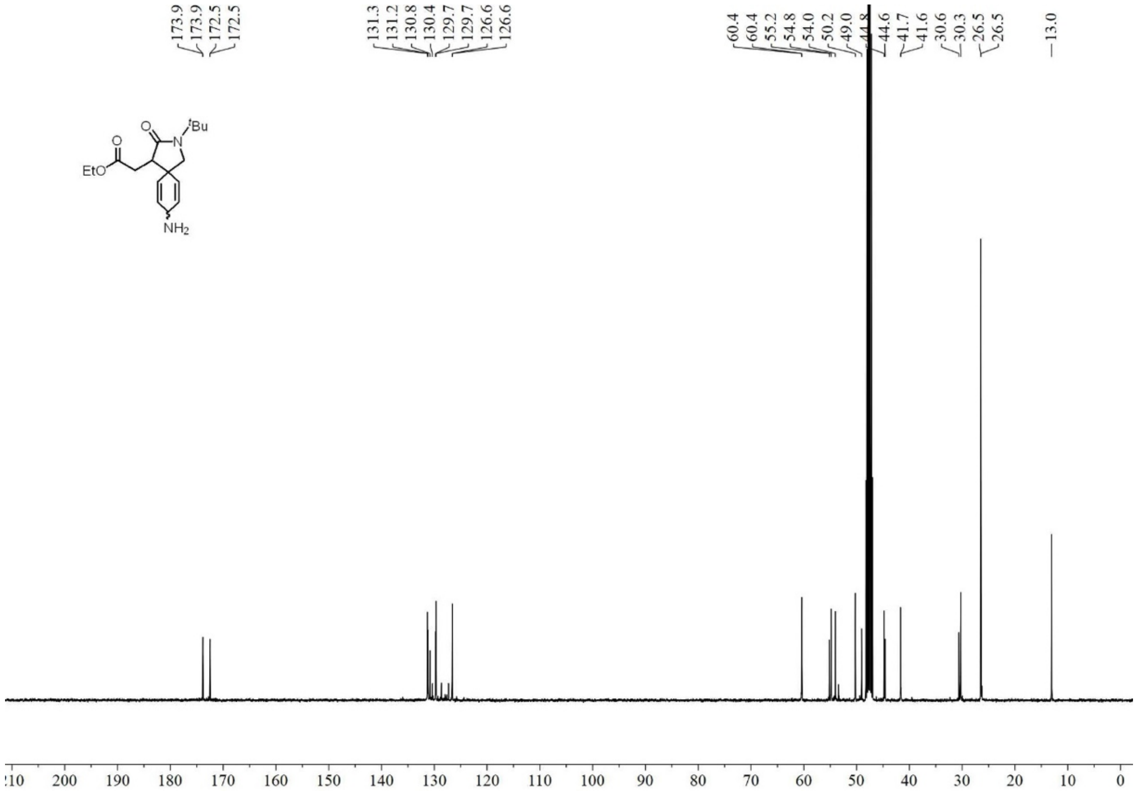

Ethyl 2-(8-(benzhydrylamino)-2-(tert-butyl)-3-oxo-2-azaspiro[4.5]deca-6,9-dien-4-yl)acetate (11)

^{1}H NMR (400 MHz, CD_3OD)

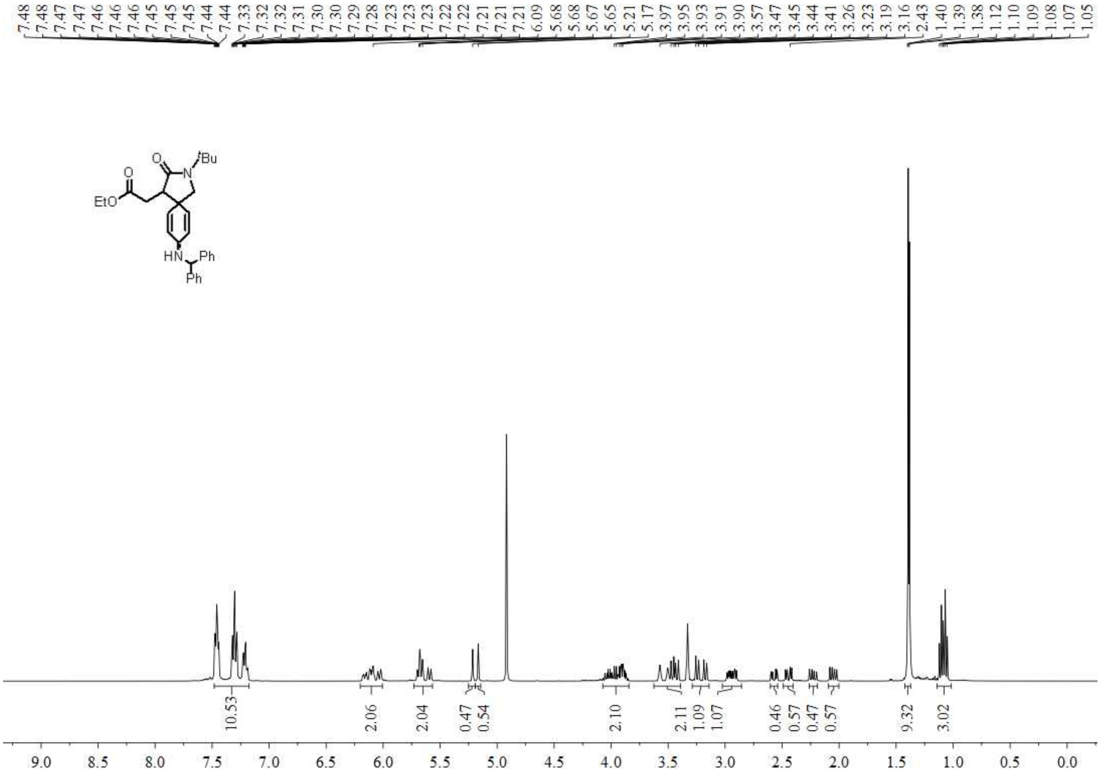

^{13}C NMR (101 MHz, CD_3OD)

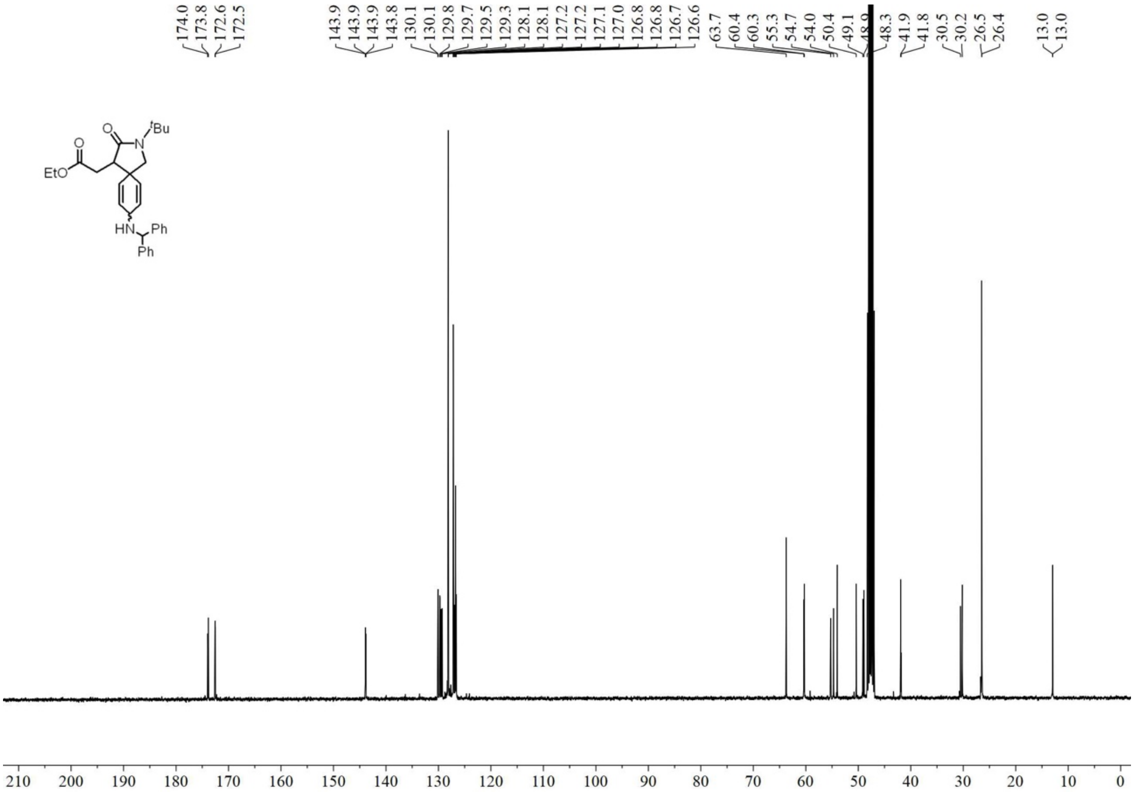

8. References

1 Rodríguez, R. I. *et al.* Taming photocatalysis in flow: easy and speedy preparation of α-aminoamide derivatives. *Green Chemistry* **24**, 6613-6618, doi:10.1039/D2GC02087D (2022).

2 Tan, G. *et al.* Highly Selective Radical Relay 1,4-Oxyimination of Two Electronically Differetiated Olefins. *J. Am. Chem. Soc.* **144**, 21664-21673, doi:10.1021/jacs.2c09244 (2022).

3 Tan, G. *et al.* Photochemical single-step synthesis of β-amino acid derivatives from alkenes and (hetero)arenes. *Nature Chemistry* **14**, 1174-1184, doi:10.1038/s41557-022-01008-w (2022).

4 Zhou, C., Shatskiy, A., Temerdashev, A. Z., Kärkäs, M. D. & Dinér, P. Highly congested spiro-compounds via photoredox-mediated dearomative annulation cascade. *Communications Chemistry* **5**, 92, doi:10.1038/s42004-022-00706-3 (2022).

5 Ma, J. *et al.* Direct Dearomatization of Pyridines via an Energy-Transfer-Catalyzed Intramolecular [4+2] Cycloaddition. *Chem* **5**, 2854-2864, doi:https://doi.org/10.1016/j.chempr.2019.10.016 (2019).

6 Wolfe, J. P., Åhman, J., Sadighi, J. P., Singer, R. A. & Buchwald, S. L. An Ammonia Equivalent for the Palladium-Catalyzed Amination of Aryl Halides and Triflates. *Tetrahedron Lett.* **38**, 6367-6370, doi:https://doi.org/10.1016/S0040-4039(97)01465-2 (1997).
